# Giving an Enzyme Scissors: Serotonin Derivatives as Potent Organocatalytic Switches for DNA Repair Enzyme OGG1

**DOI:** 10.1021/acs.jmedchem.5c01454

**Published:** 2025-10-15

**Authors:** Marek Varga, Florian Ortis, Alicia Del Prado, Alice Eddershaw, Emma Scaletti Hutchinson, Emily C. Hank, Kaixin Zhou, Natálie Rudolfová, Alessia Dodaro, Elisée Wiita, Ingrid Almlöf, Stella Karsten, Kirill Mamonov, Sara H. Ahmed, Kirsty Bentley, Olov Wallner, Evert J. Homan, Martin Scobie, Thomas Helleday, Mario Prejanò, Pål Stenmark, Miguel de Vega, Allan J. B. Watson, Maurice Michel

**Affiliations:** † EaStCHEM, School of Chemistry, 7486 University of St Andrews, St Andrews KY16 9ST, United Kingdom; ‡ 27106 Karolinska Institute, Oncology and Pathology Science for Life Laboratory, Stockholm 171 65, Sweden; § 70698 Centro de Biología Molecular Severo Ochoa, CSIC-UAM, Madrid 28049, Spain; ∥ Karolinska Institute, Oncology and Pathology, Center for Molecular Medicine, Karolinska Institute and Karolinska Hospital, L8:05, Visionsgatan 18, Stockholm 171 76, Sweden; ⊥ 7675 Stockholm University, Biochemistry and Biophysics, Stockholm 106 91, Sweden; # Dipartimento di Chimica E Tecnologie Chimiche, 18950 Università Degli Studi della, Rende, Calabria 87036, Italy

## Abstract

The base excision repair enzyme 8-oxoguanine DNA glycosylase 1 (OGG1) plays a central role in maintaining genome integrity and mediating cellular responses to oxidative stress. As such, it represents an attractive target for pharmaceutical modulation. Small-molecule organocatalytic switches (ORCAs) greatly enhance the rate of OGG1-catalyzed cleavage of DNA abasic sites, thereby accelerating DNA repair. Here, we present the discovery and hit-to-lead optimization of a novel class of highly potent serotonin-derived ORCAs with greatly improved pharmacokinetic properties. Biochemical assays, X-ray crystallography, and molecular dynamics simulations point toward a water-mediated mechanism of activation, distinct from previously proposed Brønsted base-assisted models. These findings establish serotonin-based ORCAs as promising chemical probes and potential leads for therapeutic modulation of OGG1 in oxidative stress-driven diseases.

## Graphical abstract

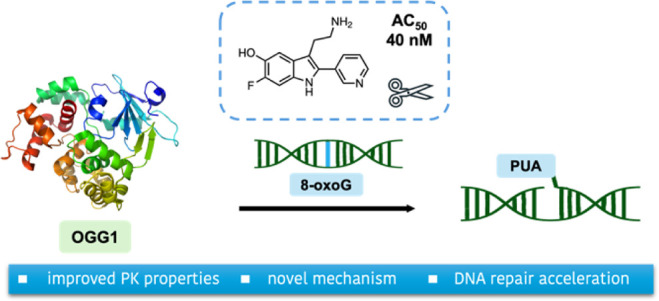

## Introduction

Enzymes represent an attractive target across many fields of science. From the medicinal chemistry perspective, 29% of all small-molecule drugs approved by the FDA in 2023 target enzymes, exclusively through enzyme inhibition.^1^ This success stems from the fact that aberrant or overactive enzymatic function is often associated with disease. However, there are many cases where enzymatic activity is beneficial, and small-molecule modulators which add or enhance an enzyme function are desirable. Reflecting this, recent years have seen a growing number of reports of “small-molecule activators”.^2,3^ The first of these are also appearing in the market, although only two, Mitapivat^4,5^ and Vericiguat,^6^ have been approved by the FDA in the past decade.^1,4,6–13^

The disparity between the number of enzyme inhibitors and activators likely reflects the inherent challenges in identifying activators. While both inhibitors and activators can bind allosterically, orthosteric activators must bind to the active site without blocking substrate binding or catalysis. Nevertheless, several classes of small-molecule activators have been successfully identified, including kinase,^14–17^ deacetylase,^18,19^ protease,^20–22^ and dehydrogenase^23^ activators, among others, with some advancing into clinical trials.^24–26^

8-Oxoguanine DNA glycosylase 1 (OGG1) is a prime example of an enzyme for which activation offers substantial therapeutic potential. OGG1 initiates the repair of 8-oxoguanine (8-oxoG),^27^ a common oxidative DNA lesion that accumulates under conditions of oxidative stress and has been linked to cancer, neurodegeneration, inflammation, aging, as well as other human conditions.^28–31^ The prevalence of 8-oxoG arises from the low redox potential of guanine compared to the other nucleobases.^32^ By excising 8-oxoG, OGG1 plays a critical role in preventing its accumulation,^33,34^ and has demonstrated protective effects in cancer,^35–37^ neurodegeneration,^38^ cardiovascular disease,^39,40^ as well as diabetes and aging.^41–44^ Furthermore, recent studies continue to uncover roles for OGG1 in regulating transcription within several signaling pathways, including those involved in inflammation,^45–52^ cancer,^53–56^ and pulmonary fibrosis.^44,57–60^

OGG1 acts as a monofunctional glycosylase, catalyzing the excision of 8-oxoG and forming a Schiff-base between Lys249 and the resulting apurinic/apyrimidinic (AP) site.^61^ Release of this covalent intermediate is rate-limiting and requires the action of apurinic/apyrimidinic endonuclease 1 (APE1) to cleave the DNA (Figure 1a).^62,63^ It has been proposed that 8-oxoG and its synthetic analogues 8-amino and 8-bromoguanine can promote proton abstraction at the C2’-position of the Schiff-base, triggering β-elimination and product release.^61,64^ However, in later studies free 8-oxoG acted as an inhibitor of both glycosylase and AP lyase activities of OGG1,^65^ and this mechanism appears to have a negligible effect in cells.^66,67^ Coenzyme-Q_10_ has been shown to increase OGG1 glycosylase activity while inhibiting the AP-lyase activity.^68^ In contrast, we and others have disclosed small-molecule *organocatalytic switches* (ORCAs) that increase OGG1 turnover by 10- to 20-fold, dramatically accelerating DNA repair.^55,67,69–71^ We postulated that ORCAs bind within the OGG1 active site, enabling either β,δ-elimination (Figure 1b), or β-elimination (Figure 1c) through Brønsted-base catalysis.^67,70^ Allosteric activators have also been suggested.^72^

**Figure 1:**
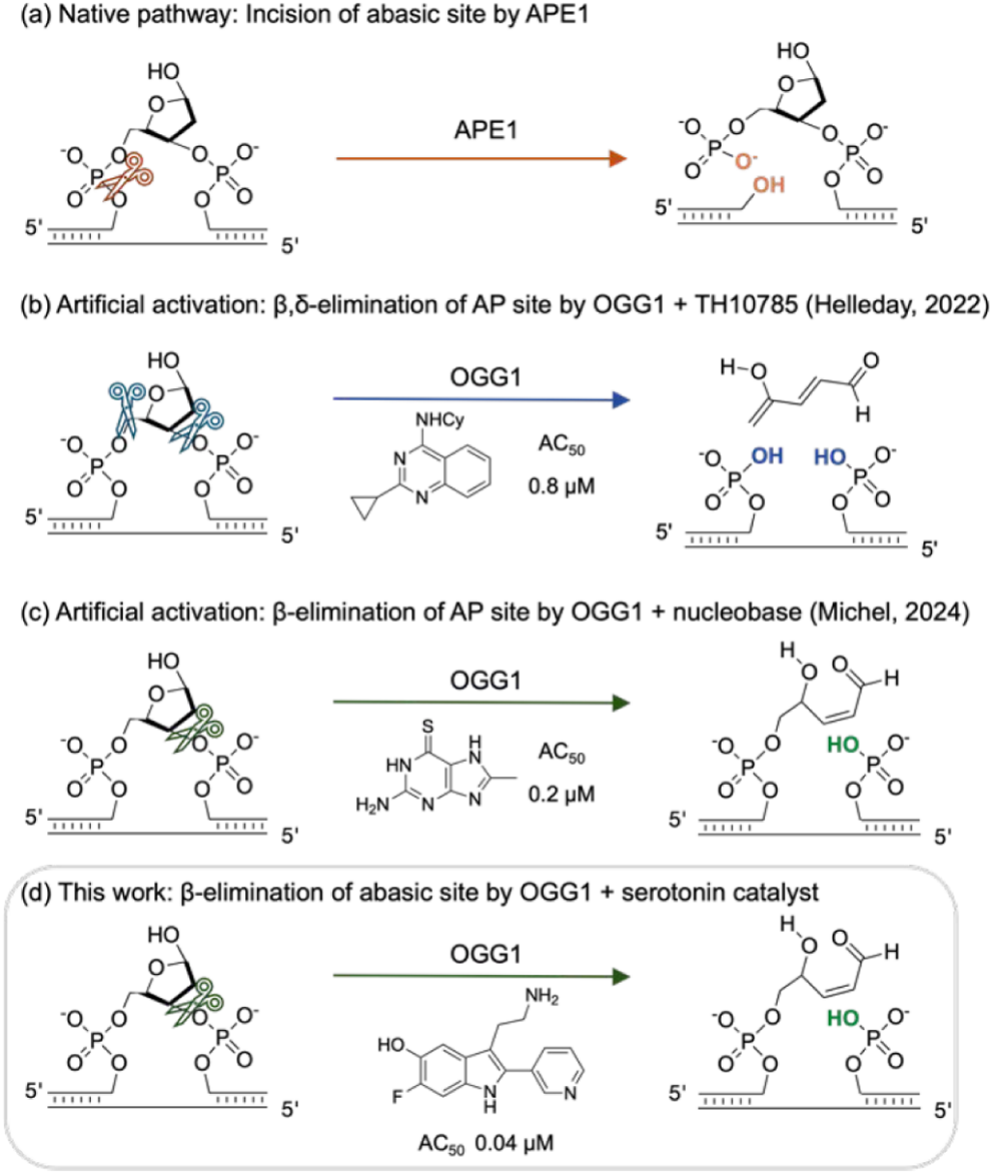
(a) Native incision pathway for AP sites generated by OGG1. (b) β,δ-elimination of AP sites by OGG1 in the presence of TH10785. (c) β-elimination of AP sites by OGG1 in the presence of nucleobase catalysts. (d) This work: β-elimination of AP sites by OGG1 in the presence of serotonin catalysts.

The therapeutic potential of OGG1 ORCAs has been demonstrated *in vitro*. For example, ORCAs protect DNA against KBrO_3_-induced damage at telomeres,^67^ and against paraquat-induced damage to mitochondrial DNA, metabolism, and membrane stability.^69,72^ Certain ORCAs can even restore activity to the OGG1 Ser326Cys variant, commonly found in Alzheimer’s disease and cancer patients.^69^ Given their mechanism, ORCAs hold the greatest promise in diseases characterized by elevated oxidative damage.

Notably, ORCAs have been shown to reduce fibrosis in a patient-derived 3D model of metabolic dysfunction-associated steatohepatitis (MASH),^71,73^ a leading cause of chronic liver-disease with limited therapeutic options.^74,75^

In this work, we sought to expand beyond established nucleobase and quinazoline-derived ORCAs to develop a new series of ORCAs with improved potency and pharmacological properties. Through the identification of a novel ORCA scaffold (Figure 1d), we also aimed to gain a deeper insight into the molecular mechanism of OGG1 activation.

## Results and Discussion

### Hit Identification

To identify novel ORCAs, an in-house small molecule library and a filtered selection of the National Cancer Institute (NCI) and National Institutes of Health (NIH) compound libraries were computationally screened. Of these, 40 were selected by docking score, structural appeal, and availability for screening (Table S1). A previously disclosed fluorescence assay featuring an 8-oxoA-containing oligonucleotide with one strand ligated to a quencher and the other to a fluorophore was used to measure the rate of incision (Figure 2).^67^ 8-oxoA was used in place of 8-oxoG due to increased stability while remaining a good substrate for OGG1. Compound potency was measured by the AC_50_, defined as the concentration of compound at which OGG1 achieved 50% of the incision rate observed with OGG1 in the presence of APE1. In this way, the 2-pyridyltryptamine derivative, **1**, was identified as an ORCA with an AC_50_ of 4.1 μM, while the *N*,*N*-dimethyl 2-phenyltryptamine derivative, TH12166, was identified as an inhibitor with an IC_50_ of 15.5 μM.

**Figure 2:**
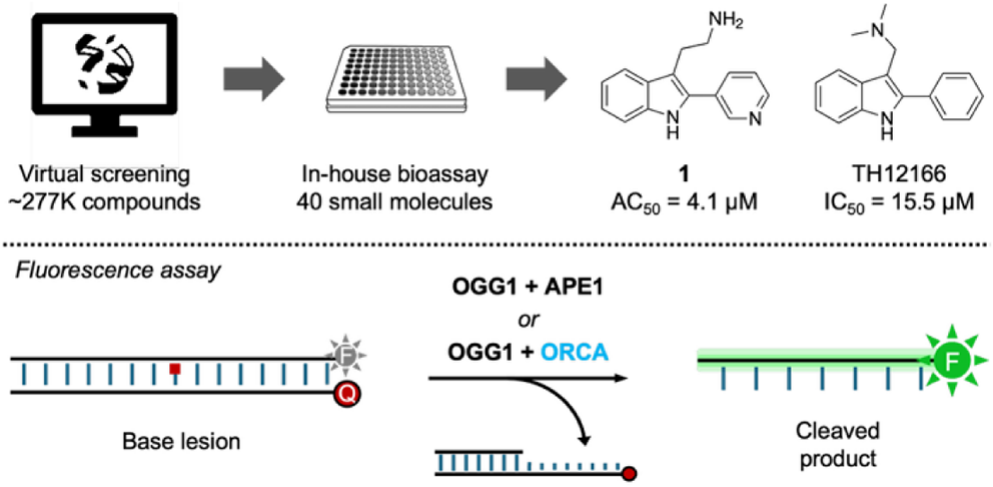
Identification of OGG1 ORCA **1** and inhibitor TH12166 (top). Illustration of fluorescence assay (bottom). *F* = 6-carboxyfluoresceine, Q = dabcyl.

### Synthesis and SAR of 2-Aryltryptamines

For initial structure–activity relationship (SAR) exploration, 2-pyridyltryptamines were synthesized via a Pd-catalyzed C2-arylation (Scheme 1A). The RuPhos ligand was essential for reactivity; however, conversions were generally poor. An initial library of 2-pyridyltryptamine derivatives was prepared (Table 1, **1**–**6**). Substitution on the indole scaffold was not tolerated (**2**–**5**), and conversion of the tryptamine amine chain to a tryptophan amino acid (**6**) abolished activity as well. Further SAR development was hindered by the requirement of prefunctionalized tryptamines and the low reliability of the C2-arylation methodology.

**Table 1:**
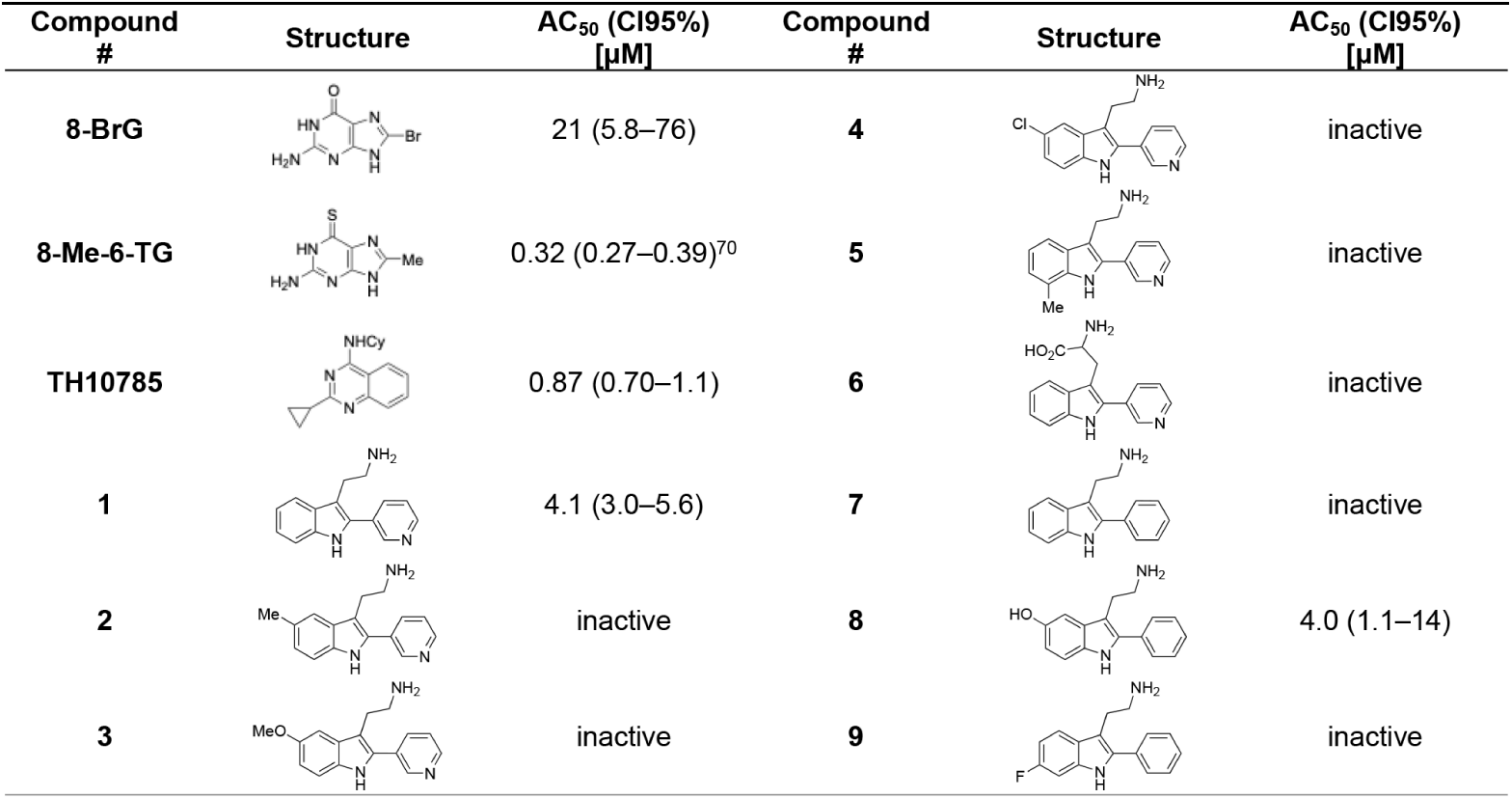
Initial *In Vitro* Evaluation of 2-Pyridyl and 2-Phenyltryptamines with Previously Disclosed ORCAs as Positive Controls

**Scheme 1:**
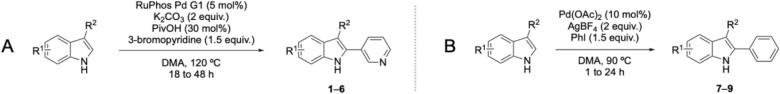
(A) Pd-Catalyzed Coupling of Tryptamines with 3-Bromopyridine. (B) Pd-Catalyzed Coupling of Tryptamines with Iodobenzene

In parallel, 2-phenyltryptamines, were evaluated for OGG1 activation (Table 1, **7**–**9**). These were accessible via a previously reported method for the C2-arylation of tryptophans (Scheme 1B), which was, however, incompatible with pyridine substrates.^76^ To our surprise, although 2-phenyltryptamine was inactive, the 5-hydroxy derivative, **8**, showed potency despite lacking the pyridyl motif. This observation suggested that the primary role of the pyridyl group may be to promote active site binding, rather than direct participation in Brønsted base catalysis of β-elimination.

### Synthesis and SAR Analysis of 2-Pyridylindoles

To further explore the SAR of ORCAs, we turned our attention to the more synthetically accessible 3-unsubstituted 2-pyridylindoles (Table 2). These compounds were primarily prepared using either the Fischer indole synthesis, or the Suzuki-Miyaura cross-coupling of *N*-Boc-indole-2-boronic acid (see Experimental).

**Table 2:**
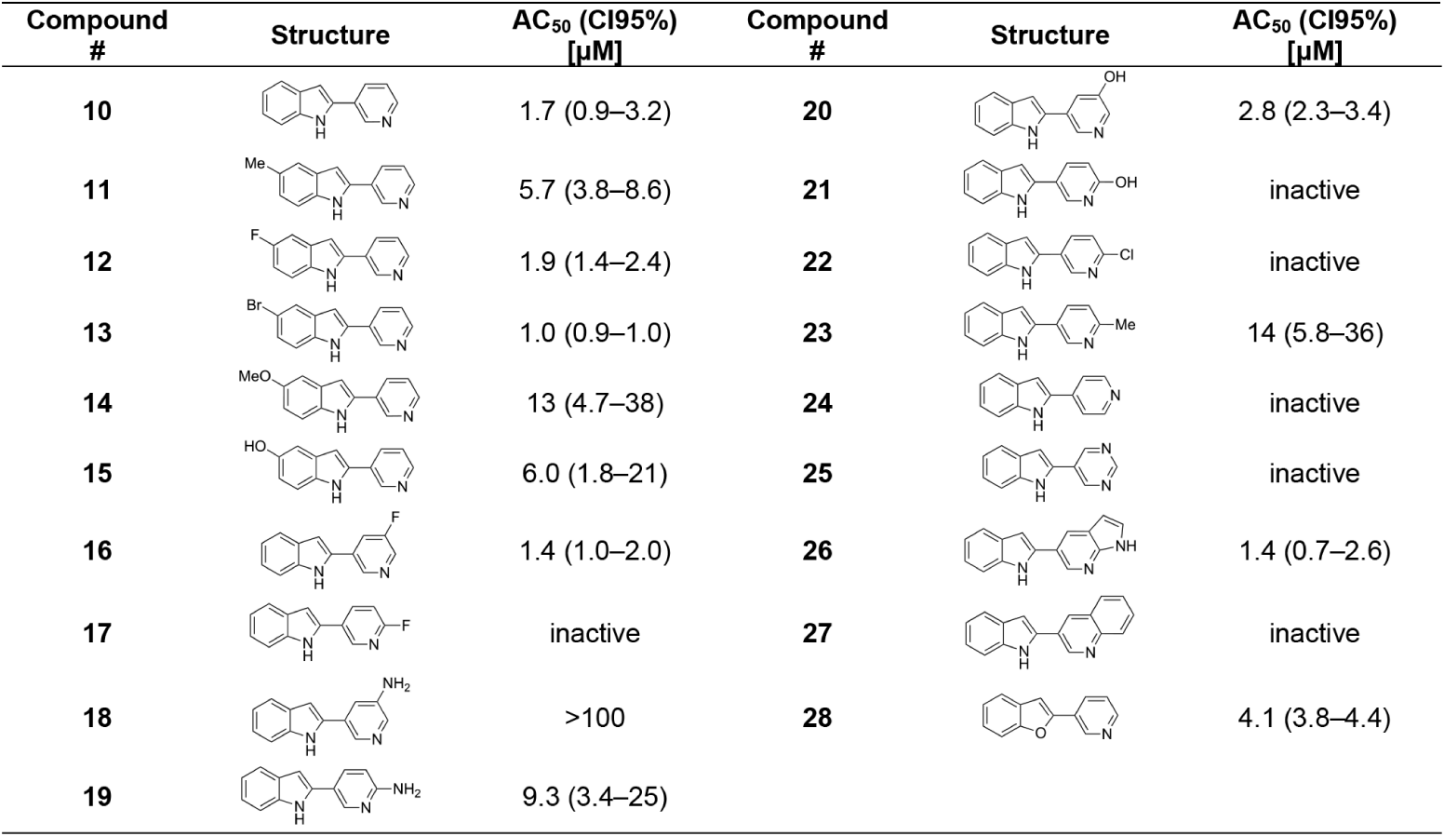
*In Vitro* Evaluation of 2-Arylindoles

Interestingly, removal of the ethylamine chain, present in the tryptamine series, led to an increase in potency, with the 2-pyrid-3-ylindole, **10**, having an AC_50_ of 1.7 μM. However, despite extensive SAR efforts, further improvements in potency proved challenging. Substitutions at the 5-position of the indole ring had limited effects (**11**–**15**), whereas substitution on the pyridine was generally detrimental (**16**–**23**). Replacement of the pyridin-3-yl motif with other heterocycles, pyridin-4-yl (**24**), pyrimidin-5-yl (**25**), or quinoline (**27**) was not tolerated, with the exception of the azaindole (**26**). Similarly, substitution of the indole core for a benzofuran scaffold (**28**) did not improve potency.

### Cocrystal of OGG1 and 1

To rationalize the observed SAR and guide further efforts, we solved the cocrystal structure of mouse OGG1 (mOGG1) in complex with **1** (Figure 3A and Table S2). mOGG1 was utilized for structural studies as it has an identical active site structure to human OGG1 but is significantly more amenable to crystallization. The crystal structure was obtained in the absence of a DNA substrate, which may not fully represent the active catalytic conformation. However, we have previously observed correlations between binary OGG1-ORCA complex structures and catalytic potency.^67,70,71^ Furthermore, we noted significant overlap between the active site structure of the NaBH_4_-trapped Schiff-base ternary complex with 8-oxoG (PDB ID: 1HU0) and the binary complexes of nucleobase ORCAs (PDB IDs: 8BQ7, 9F8U, 9F8 V, 9F8Z),^70^ supporting the use of these structures as reasonable approximations of the catalytic conformation. The crystal structure unambiguously placed **1** within the OGG1 active site, engaging in a binding mode consistent with previously reported ORCAs.^67,70,71^ Key interactions included H-bonds to Gly42, Gln315, a salt bridge to Asp268, π-stacking with Phe319, and a water-mediated H-bonding network. Analogous to previous ligands where the Gly42 H-bond was mediated via an aniline or a secondary amine, the indole NH formed this crucial interaction.

**Figure 3:**
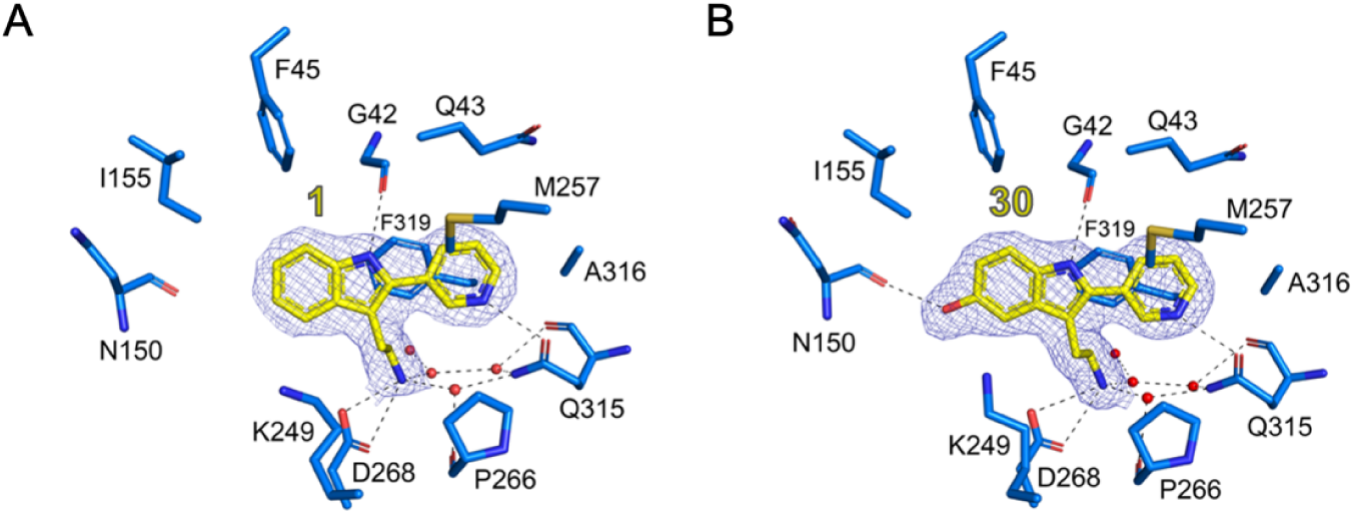
(A) Recognition of **1** by mOGG1 (PDB ID: 9FNV). (B) Recognition of **30** by mOGG1 (PDB ID: 9FNU). Amino acids contributing to ligand binding are depicted as sticks: C atoms are colored blue, O atoms red, and N atoms dark blue. Ligands are depicted as stick models: C atoms colored yellow, N atoms dark blue, and O atoms red. Hydrogen bond interactions are shown as dashed lines. Water molecules are shown as red spheres. The 2Fo-Fc electron density maps around the ligands are contoured at 1.0 σ (blue) and the Fo-Fc electron density maps are contoured at +3.5 s (green) and −3.5 s (red). Figure produced using PyMOL (v.2.3.3, Schrödinger).

As hypothesized, the pyridyl moiety was involved in bonding interactions and was found deep in the OGG1 catalytic pocket, forming a H-bond to Gln315. Importantly, the H5 of **1** was found to reside within 3.2 Å of Asn150 (Figure S2), providing a possible explanation for the potency of **8** via a tentative H-bonding interaction between its 5-OH substituent and Asn150. This insight provided the rationale to develop an alternative synthetic strategy that would enable the incorporation of the 5-OH onto **1**, as well as further SAR exploration of the tryptamine scaffold.

### Larock Indole Synthesis of 2-Pyridyltryptamines

The new synthetic route centered around the Pd-catalyzed Larock indole synthesis, which allowed for far greater modularity (Scheme 2).^77,78^ This approach allowed for broad variation of starting materials: 2-iodoacetanilides (S1a–l) could often be prepared from commercially available 2-iodoanilines or 2-iodonitrobenzenes, while Boc-protected aminoalkynes (S2a–j) were prepared through Sonogashira coupling reactions. Following Larock cyclization, intermediates bearing methyl or benzyl ethers were treated with BBr_3_ to afford the corresponding phenols (**30**–**33**, **38**–**45**, **52**–**53**). Other derivatives were deprotected under a variety of acidic conditions to afford the desired tryptamines (**1, 34**–**37, 46**–**47, 50**).

**Scheme 2:**
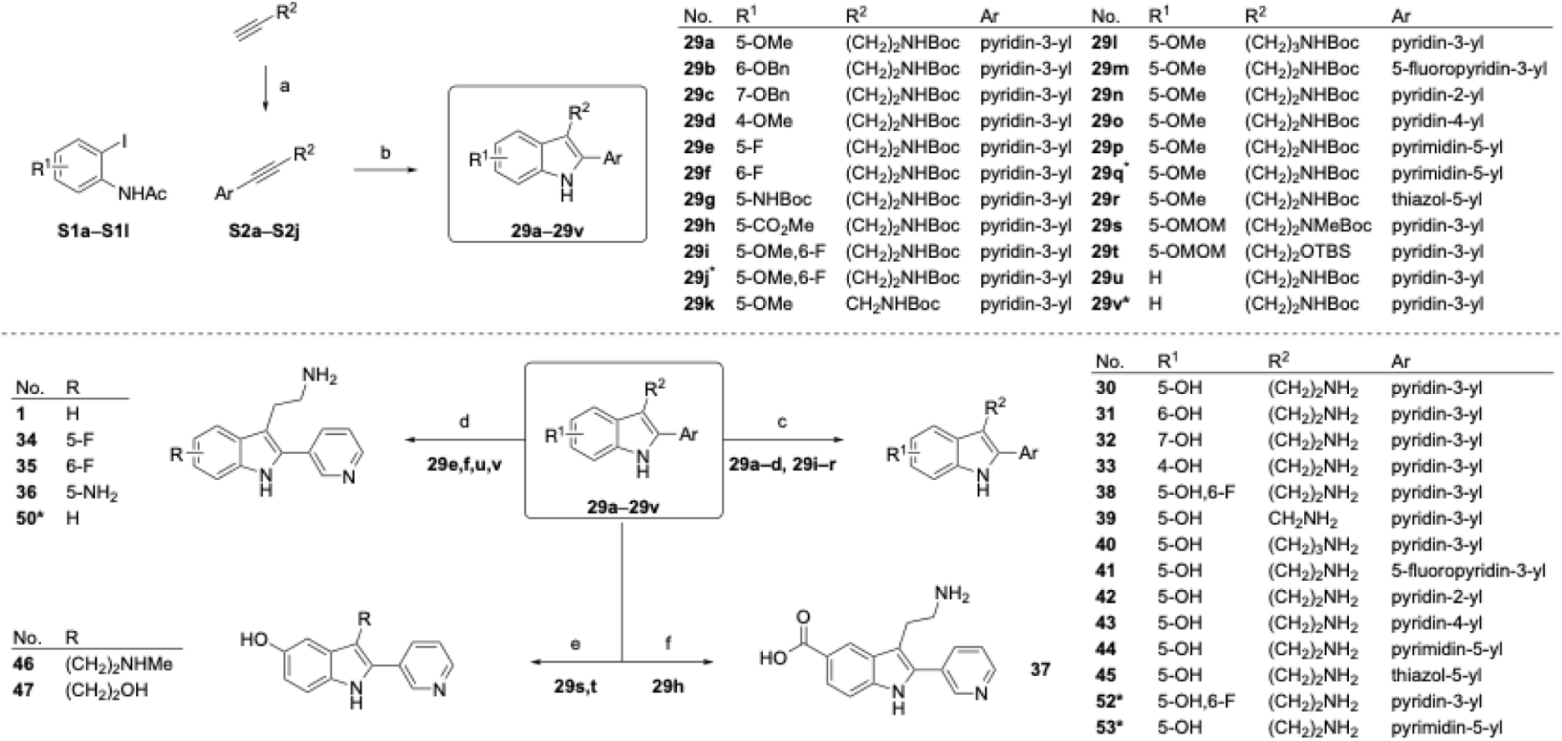
Larock Indole Synthesis Route to Substituted 2-Aryltryptamines.^a^

a (a) PdCl_2_(PPh_3_)_2_ (3 mol %), CuI (9 mol %), ArX (1.5 equiv), RT for X = I, 90 °C for X = Br, NEt_3_, 3 h; (b) 1. Pd­(^t^Bu_3_)_2_ (5 mol %), S1a–S1l (1.3 equiv), Cy_2_NMe (2 equiv), DMF, 80 °C, 24 h 2. LiOH (5 equiv), THF/H_2_O (3:1), 45 °C, 3 h (c) BBr_3_ (8 equiv), 1,2,4,5-tetramethylbenzene (8 equiv) CH_2_Cl_2_, – 84 °C to RT, overnight; (d) TFA (20% in CH_2_Cl_2_), 0 °C to RT, 2 h; (e) MeOH/3 M HCl (3:1), reflux, 2 h; (f) AcOH/conc. HCl (3:1), reflux, 18 h. *Tryptamine regioisomers with ethylamine chain in the C2-position and aryl group in the C3-position.

### Second Round of 2-Pyridyltryptamine Optimization

This new synthetic route enabled the preparation of a second library of 2-pyridyltryptamines (Table 3). Notably, we found that introduction of a 5-OH substituent in **30** improved potency 10-fold over **1**, consistent with the activity of the 2-phenyl analogue, **8**. Evaluation of other hydroxy-substitution patterns revealed that the 5-OH position was essential for activity as the 4-OH, 6-OH, and 7-OH derivatives (**31**–**33**) were universally inactive. 5-F (**34**), and 6-F (**35**) derivatives were tolerated but neither improved on the potency of **1**. Replacement of the 5-OH with other H-bond donors, such as aniline (**36**) or carboxylic acid (**37**) analogues, was not tolerated. To elaborate on the enhanced potency of **30**, a cocrystal structure with mOGG1 was obtained (Figure 3B). As expected, the 5-OH formed a new H-bond to Asn150 while maintaining all key interactions observed for **1**. In contrast, the 4-, 6-, and 7-OH derivatives were likely positioned too far to Asn150 to enable this interaction.

**Table 3:**
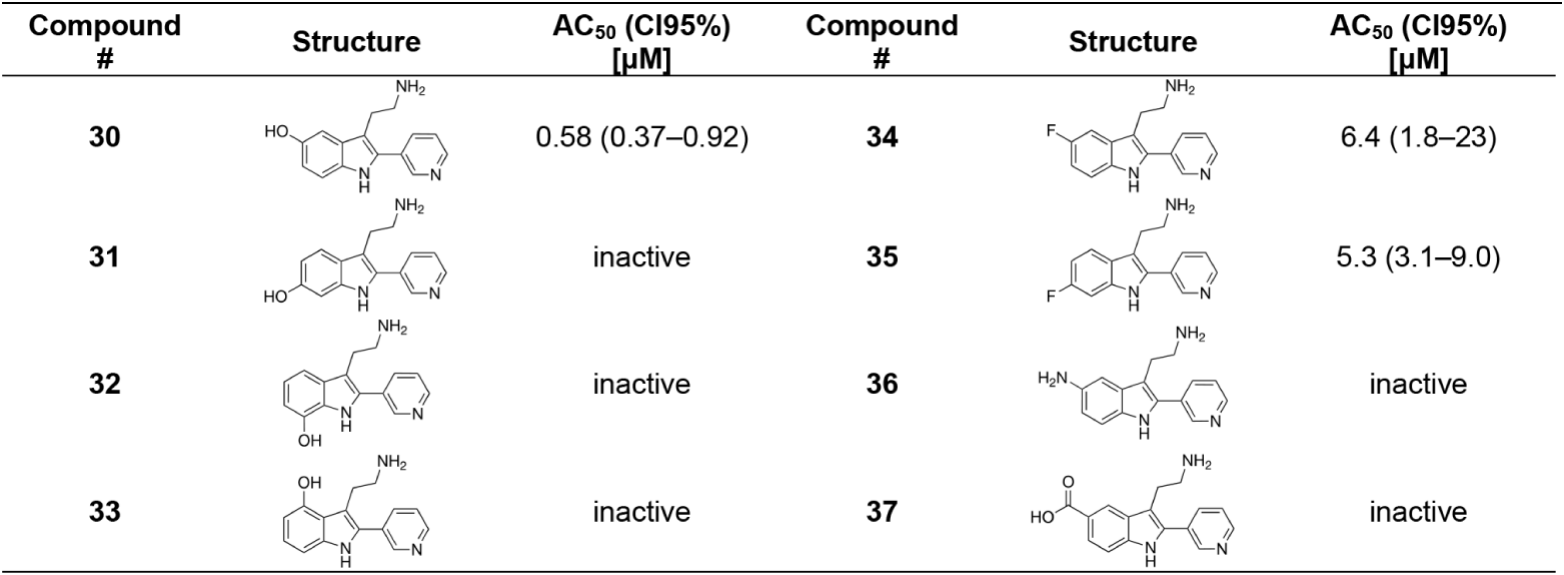
Second Round *In Vitro* Evaluation of 2-Pyridyltryptamines

### Computational Analysis

A computational docking screen of the tryptamines synthesized was performed to guide further SAR exploration (Table S3). This was conducted using the mOGG1-**30** cocrystal structure. Interestingly, the highest-scoring ligand was the 6-F tryptamine **35**, despite its moderate activity in the fluorescence assay. A hydrophobic interaction with Ile155 was identified as the possible origin of this affinity (Figure S3A). Another novel interaction was observed for **32** where the 7-OH moiety formed a H-bond to Gly42 (Figure S3B). These insights prompted the synthesis of 5-OH-6-F, and 5,7-diOH hybrid compounds. The synthesis of the latter was unsuccessful, likely due to product instability. In addition, a series of further serotonin-derived analogues was prepared (Table 4).

**Table 4:**
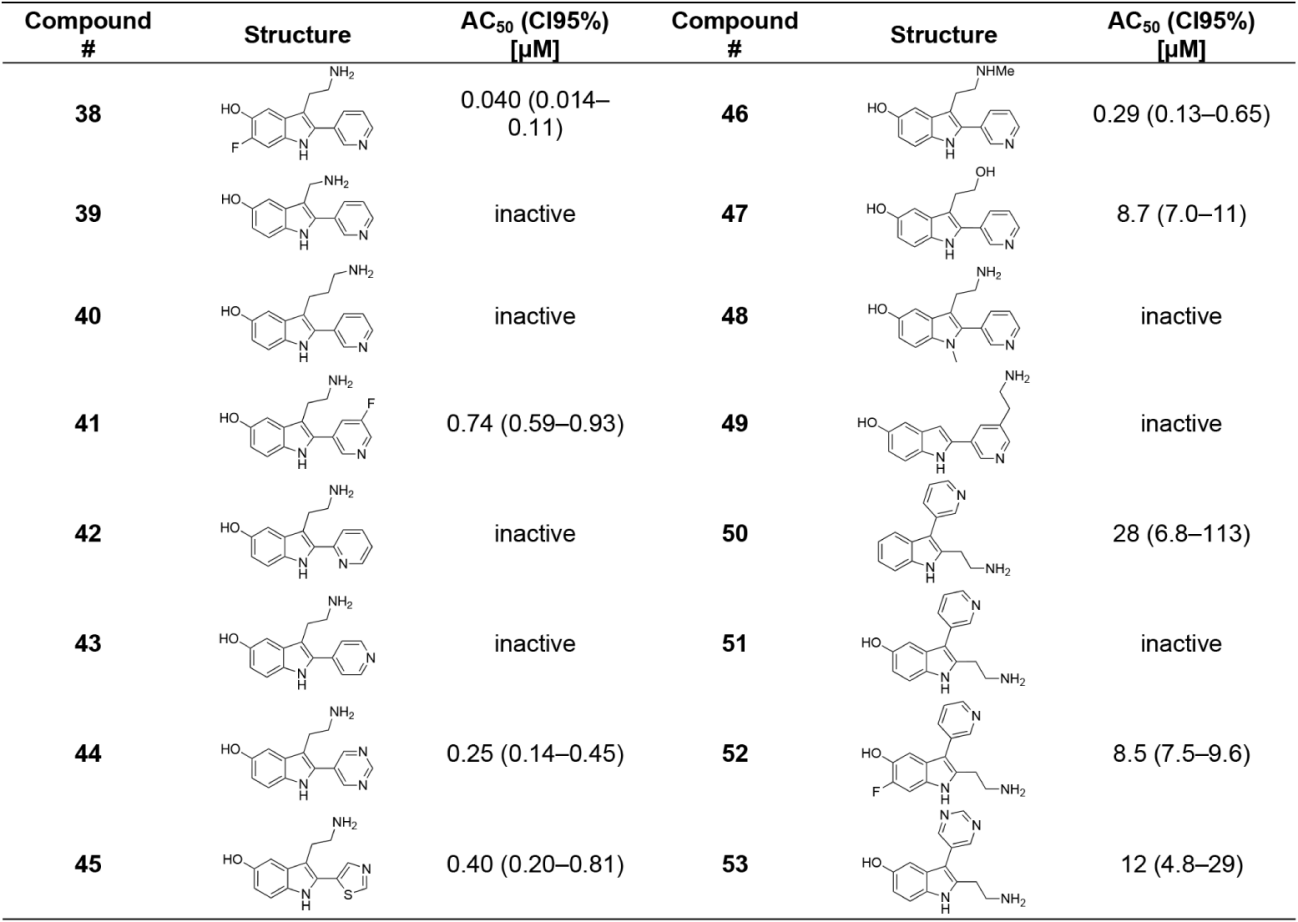
Third Round *In Vitro* Evaluation of 2-Pyridyltryptamines

### Third Round of 2-Pyridyltryptamine Optimization

Importantly, the 5-OH-6-F hybrid, **38**, exhibited the highest potency identified in this series with an AC_50_ of 0.040 μM, consistent with novel hydrophobic interactions or increased H-bond donating ability of the phenol. Shortening (**39**) or lengthening (**40**) of the ethylamine chain of **30** was not tolerated.

Substitution on the pyridin-3-yl group was also explored. Fluorination did not significantly affect potency (**41**), whereas the position of the pyridine N was essential as the pyridin-2-yl (**42**) and pyridin-4-yl (**43**) derivatives lost activity. However, the pyridine could be substituted with either pyrimidine (**44**) or thiazole (**45**) heterocycles to achieve a slight increase in activity. Amine monomethylation produced a similar increase in potency (**46**), unlike N1-methylation of the indole (**48**), which attenuated activity, presumably by interrupting H-bonding to Gly42. These results were consistent with previous work where interaction with Gly42 was essential for activation.^67,70^ The ethanol derivative **47** decreased activity ∼10-fold, demonstrating the importance of the ionic interactions of the amino group. An isomer placing the ethylamine on the pyridine ring rather than the indole (**49**) was inactive.

Finally, regioisomeric side products of the Larock cyclization, in which the positions of the ethylamine chain and the aryl group were reversed, were isolated and evaluated (**50**–**53**). Despite some residual activity for **52** and **53**, these isomers were generally less potent or inactive compared to their tryptamine analogues.

### Selectivity and Toxicity

To ascertain the selectivity of the tryptamine ORCAs, **30** was screened against a panel of DNA glycosylases and nucleotide-binding NUDIX hydrolases. **30** had no effect on NUDIX hydrolases or APE1, and a >150-fold selectivity for OGG1 activation over inhibition of other glycosylases (Table S4). This represents a slight improvement over the already high (>120-fold) selectivity of the established ORCA, TH10785. Compound **30** also improves on the toxicity of TH10785 which shows some toxicity in the immortalized BJ-TERT and oncogene-driven BJ-RAS,^79,80^ and inhibits proliferation in activated peripheral blood mononuclear cells (PBMCs, Figure S4). In contrast, **30** showed no detectable toxicity when incubated with the BJ-derived cells at 100 μM over several days (Figure S5).

### Pharmacokinetic Analysis

The primary goal of this series was to improve on the physicochemical properties of known ORCAs. For example, TH10785 has a relatively high *c*logP of 4.54, a low aqueous solubility of 6 μM and a suboptimal efflux ratio of 3.3 (Table 5). In addition to improved toxicity, **30** also exhibits a 58-fold higher aqueous solubility, and a 5-fold lower *c*logP compared to TH10785. The efflux ratio (4.1) remains at an acceptable level but should be addressed for further clinical development. The substantial decrease in efflux (1.7 vs. 4.1) in the presence of a P-glycoproptein (P-gp) efflux protein inhibitor, verapamil, suggests that **30** is a P-gp substrate and subject to active efflux.

**Table 5:** Comparison of **30** and TH10785

	**30**	**TH10785**
AC_50_ [μM]	0.58	0.78 ^67^
incision mode	β	β,δ
selectivity^a^	>150-fold	>120-fold^70^
toxicity [μM]^b^	>100	>18 (20)
*c*logP^c^	0.84	4.54
AcLE^d^	0.456	0.419
AcLLE^e^	5.49	1.57
aq. solubility (μM)^f^	345	6
Cl_int_ (μL/min/mg)^g^	8	18 (347)
*t*_1/2_ (min)^g^	213	-
*k*_el_ (min^–1^)^g^	0.003	-
*P*_app_ (AB) (nm/s)^h^	5.3 (10.7)	15.0
*P*_app_ (BA) (nm/s)^h^	21.5 (18.7)	49.0
Efflux ratio^i^	4.1 (1.7)	3.3

a Calculated as lowest IC_50_ measured against a panel of DNA glycosylases and NUDIX nucleotide binding proteins/OGG1 AC_50_.

b Incubation in BJ-RAS and BJ-TERT cell lines. Value in parentheses obtained using PBMCs.

c Calculated using InstantJChem version 23.16.

d AcLE = (1.37/#heavy atoms) × pAC_50_.

e AcLLE = pAC_50_ – *c*logP.

f Kinetic solubility in PBS buffer (pH 7.4).

g Metabolic stability evaluated in human (mouse) liver microsomes.

h Using Caco-2 cell line. Value in parentheses obtained in the presence of P-gp inhibitor (verapamil).

i Calculated by dividing *P*_app_ (B to A) by *P*_app_ (A to B).

Surprisingly, **30** was remarkably stable when exposed to liver microsomes, despite the presence of phenol^81^ and amine^82^ functionalities, both typically liable to rapid metabolism, with serotonin itself being a natural substrate of monoamine oxidase.^83^ Here, **30** showed less than half the clearance rate of TH10785 with a microsomal half-life exceeding 3.5 h.

Finally, to evaluate the drug-likeness of ORCAs we propose the metrics “AcLE” and “AcLLE”, modified forms of the ligand efficiency (LE) ligand-lipophilicity efficiency (LLE) metrics, where pIC_50_ is substituted by the pAC_50_ in the standard calculations of these measures.^84^ Using these metrics, **30** showed a slightly better AcLE than TH10785 and a markedly higher AcLLE, attributable to its significantly lower lipophilicity.

### Activity of OGG1 in the Presence of 2-Pyridyltryptamines

Different ORCAs have been shown to have distinct effects on the OGG1-catalyzed incision of apurinic/apyrimidinic (AP) sites. Nucleobase-derived ORCAs facilitate β-elimination of the Schiff-base intermediate, generating 3′-phosphate unsaturated aldehyde (3′-PUA) products that are further processed by APE1.^70^ Other ORCAs, such as TH10785, enable β,δ-elimination, which produces 3′-phosphate (3′-P) termini that cannot be processed by APE1, thereby redirecting the repair pathway toward polynucleotide kinase phosphatase (PNKP1).

To investigate the molecular mechanism of OGG1 activation by 2-pyridyltryptamines, a PAGE assay was performed. A [^32^P]-labeled double-stranded DNA substrate containing 8-oxoG (Figure 4A) was incubated in the presence (+) or absence (−) of hOGG1 and either ORCAs **30**, **38**, or TH10785, or the OGG1 inhibitor TH5487. The resulting products were analyzed by urea-PAGE (Figure 4B). Stimulation of OGG1 AP lyase activity was observed for TH10785 and **38**, whereas the effect of **30** was not significant (Figure 4C). This lack of effect may be attributed to differences in assay conditions relative to the fluorescence assay, which contains 2 mM MgCl_2_, a known inhibitor of OGG1 AP lyase activity.^65^ As previously reported,^67^ TH10785 generated both β and β,δ-elimination products (3′-PUA and 3′-P, respectively), whereas **30** and **38** primarily induced β-elimination.

**Figure 4:**
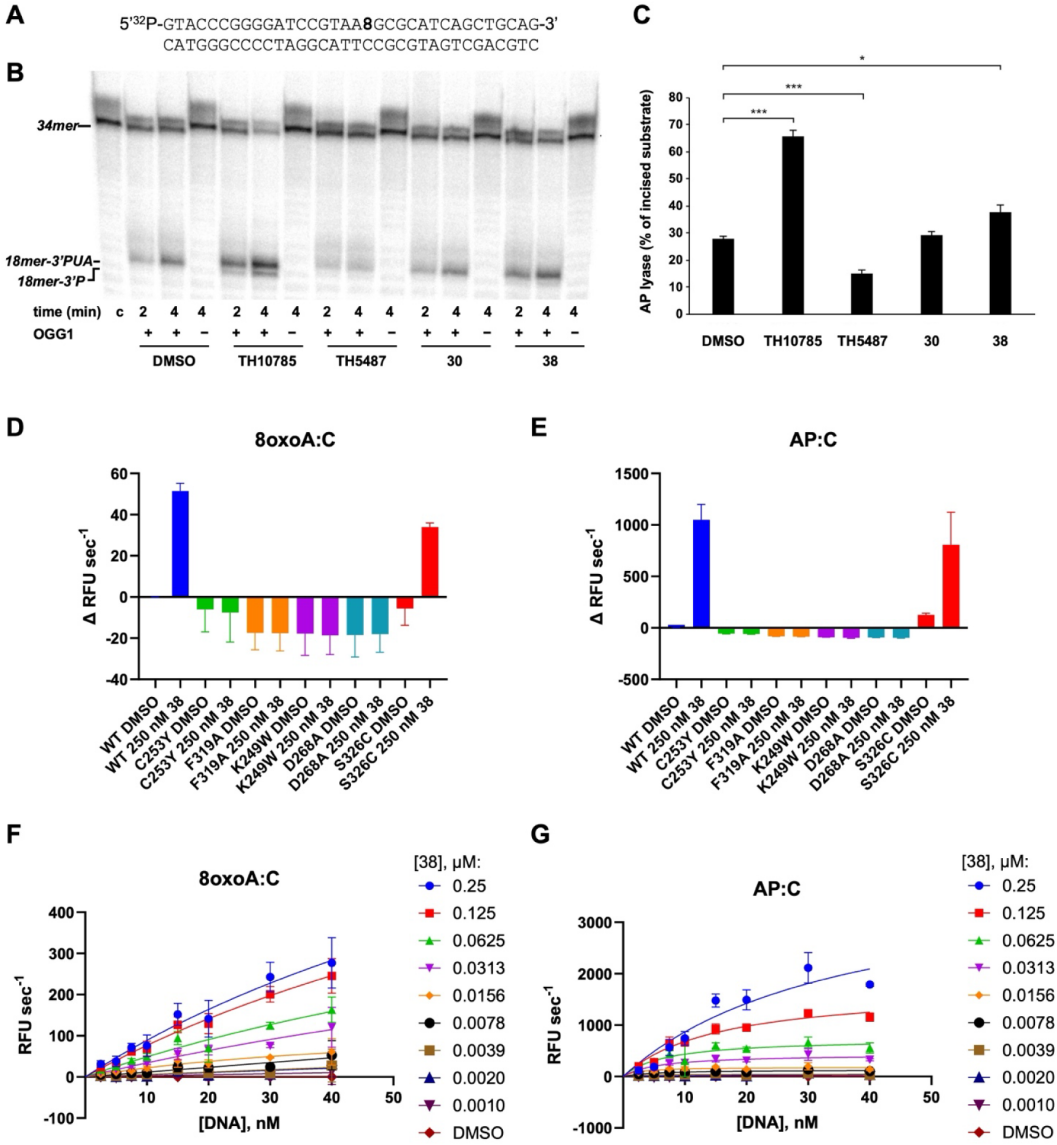
Effect of tryptamine ORCAs on the catalytic activity of OGG1. (A) [^32^P]­5′-labeled 8-oxoG-containing DNA substrate used during the reaction (8 stands for 8-oxoG). (B) 2 nM of the 8-oxoG-containing DNA substrate was incubated with either 10% DMSO, 6.25 μM TH10785, 50 μM TH5487, 6.25 μM **30**, or 10 μM **38**, as indicated, and in the presence (+) or absence (−) of 10 nM hOGG1. After incubation for the indicated times at 37 °C, reactions were stopped, the products were resolved by 7 M urea-20% PAGE and visualized by autoradiography. Position of products is indicated. (C) Bar chart shows the percentage of incised substrate obtained by hOGG1 after 4 min of reaction in the presence of the indicated compound (*n* = 3 each; means ± SEM). Significance of results was determined with a two-tailed paired *t*-test. *p < 0.05; **p < 0.01; ***p < 0.001. (D) and (E) Assessment of wt and mutant OGG1 activation by **38**. Initial rates measured in the standard fluorophore-quencher assay using 10 nM 8-oxoA:C (D) or AP:C (E) substrate, 10 nM of wt, C253Y, F319A, K249W or D268A hOGG1, or 50 nM S326C hOGG1 and 0.25 μM of **38**; Δ*R*FU s^–1^ = change of relative fluorescence units per second relative to wt hOGG1 DMSO. (F) and (G) Initial slope measured in the standard fluorophore-quencher assay using either the 8oxoA:C (F) or AP:C (G) DNA substrate at indicated concentrations in the presence of 10 nM hOGG1 and either DMSO or **38** at indicated concentrations. AP-sites were generated from U:C substrate using UDG.

Because the assay in Figure 4B reflects the combined outcome of OGG1 glycosylase and AP-lyase activity, we next assessed the effect of serotonin ORCAs on each reaction individually. No significant effect on OGG1 glycosylase activity was detected (Figure S6), whereas cleavage of an AP-substrate was accelerated (Figure S7). The requirement for OGG1-ORCA cocatalysis was demonstrated by the lack of reaction in the absence of hOGG1.

Compound **38** was further evaluated against a panel of OGG1 mutants including mutations within and outside the active site, using the established fluorescence assay with either an 8-oxoA or AP substrate (Figure 4D,E). Promisingly, **38** accelerated the AP-lyase activity of the disease-associated mutant Ser326Cys. In contrast, **38** did not restore activity in active site mutants, consistent with the active site binding mode observed in the mOGG1-**38** crystal structure.

### Kinetic Analysis

Saturation kinetic studies where the rate of DNA cleavage was measured at increasing concentrations of DNA substrate revealed a faster resolution of AP-sites (Figure 4G) than 8-oxo sites (Figure 4F). This preference is consistent with previous observations for TH10785,^67^ nucleobase,^70^ and pyridine ORCAs.^71^ The increased rate likely stems from a lack of competition for active-site binding between the ORCA and the 8-oxo substrate, and the hydrolysis of the glycosidic bond being the rate-determining step in the presence of ORCAs. Another consequence of this acceleration was that a plateau was not achieved at 40 nM of the AP:C substrate, the highest concentration achievable within the assay.

### Mechanistic Discussion

Various mechanisms have been postulated for the acceleration of OGG1 AP-lyase activity by small-molecules.^61,67,70,72^ It has been suggested that the excised 8-oxoG may remain in the active site and catalyze β-elimination, although with negligible activity.^61,64,65,67^ Structurally similar, but significantly more potent nucleobase ORCAs seem to follow the same mechanism, binding in a similar manner to 8-oxoG.^61,70^ This hypothesis is further supported by the pH-dependence of nucleobase and pyridine ORCA activity, with compounds performing best at pH levels close to the p*K*_a_ of their basic nitrogen.^70,71^ Similarly, the quinazoline ORCA TH10785 has been shown to bind in the active site and seems to rely on the basicity of its nitrogen (p*K*_a_ ≈ 6.55) for activity.^67^

### Base-Assisted Catalysis

Our initial screening results were consistent with a base-assisted catalysis mechanism of activation. While the 2-pyridyltryptamine ORCA **1** enhanced activity, the 2-phenyltryptamine derivative TH12166 bound to OGG1, but inhibited activity. This was further supported by the complete loss of activity in the phenyl analogue of **1**, compound **7**. Therefore, we hypothesized that a basic nitrogen able to participate in proton exchange was required for activation. However, the activity of the 2-phenyltryptamine **8** was not consistent with base-mediated activation. Although the activity of **8** is lower compared to its pyridyl analogue, the acceleration of AP-lyase activity was significant and implied the pyridine moiety in binding interactions rather than directly in activation.

### Allosteric Activation

These results prompted us to investigate other possible modes of activation, including allosteric modulation. An allosteric mode of action has previously been proposed for the 8-oxoG analogue 8-bromoguanine as well as other undisclosed ORCAs.^72^ This was based on the argument that active site binding would result in competition between the 8-oxoG substrate and the ORCA. In contrast, initial rate analysis suggested that 8-bromoguanine binds with a similar affinity to the free enzyme and to the enzyme–substrate complex, consistent with a noncompetitive binding model. However, this could alternatively reflect the low affinity of the enzyme to 8-oxoG following base excision, as previously reported,^64,85^ which would enable a rapid displacement of 8-oxoG by an ORCA following base excision.

In principle, an ideal ORCA would selectively bind to the Schiff-base DNA-enzyme complex without interfering with substrate binding. Nonetheless, partial inhibition of substrate binding can still result in net rate acceleration, since OGG1-catalyzed base excision is orders of magnitude faster than AP-lyase cleavage in the absence of an ORCA.^64^ This balancing act between activation and inhibition is often visible in the form of a bell-shaped dose–response curve for TH10785 as well as ORCAs identified here and previously.^67,70,71^ In these cases, rate acceleration peaks at a certain concentration, and then declines at higher loadings, consistent with active-site inhibition. These observations, along with several OGG1-ORCA crystal structures presenting clear active-site binding and extensive SAR relationships with regards to active-site interactions, further supported by active-site mutant studies, argue against an allosteric mechanism. Nevertheless, allostery remains a possible mechanism of activation for other ORCAs, and putative allosteric binding sites have been identified computationally.^72,86^

### Schiff-Base Surrogate

DNA glycosylases can be broadly classified as monofunctional or bifunctional, depending on whether they possess AP-lyase activity in addition to base excision. Structurally, bifunctional glycosylases are distinguished by the presence of an N-terminal valine or proline α-NH_2_ or a catalytic lysine residue in the active site — Lys249 in OGG1 — which traps the S_N_1 oxocarbenium intermediate generated during base excision (Figure S1).^87,88^ This results in the formation of a Schiff-base intermediate. In contrast, monofunctional glycosylases rely on a nucleophilic water molecule within the active site to trap the oxocarbenium species.^89^ Given the lysine-like ethylamine tail present in the tryptamine ORCAs, we considered whether this moiety might act as a surrogate for Lys249. For this purpose, we expressed the Lys249Ala and Lys249Trp mutants of OGG1, which lack glycosylase and AP-lyase activity. However, both **30** and **41** failed to restore AP-lyase activity of the Lys249Ala mutant in a PAGE assay (Figure S8), while **38** failed to restore activity of the Lys249Trp mutant in the standard fluorescence assay, suggesting 2-pyridyltryptamines cannot replace Lys249 in the active site.

Additionally, we confirmed that ORCAs do not exhibit their effect through accelerating the rate of Schiff-base formation, a key step for AP-lyase activity of bifunctional glycosylases. To test this, we trapped the Schiff base intermediate using NaBH_4_ to generate an OGG1-DNA cross-link. As shown in Figure 5B, ORCAs TH10785, **30** and **38** had no discernible effect on the level of cross-linked product formed with either AP or 8-oxoG DNA substrates. These findings are consistent with rapid Schiff-base formation during/immediately after base excision, followed by ORCA binding to facilitate its cleavage.

**Figure 5:**
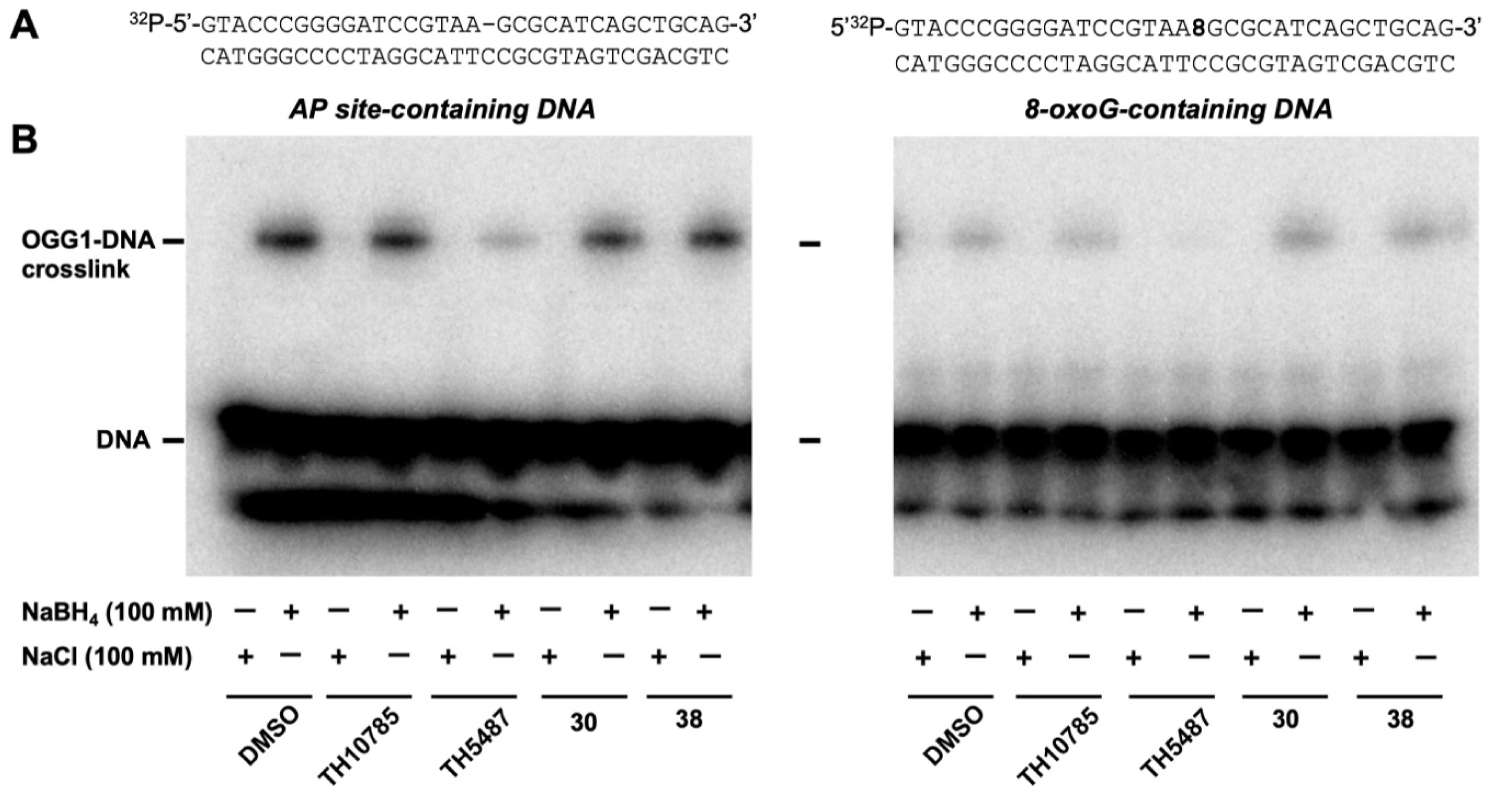
(A) The [^32^P]­5′-labeled DNA substrates used. The AP-containing DNA substrate was prepared by treating an uracil-containing substrate with *E*. *coli* UDG. (B) Schiff-base trapping assay. 78 pM hOGG1 was incubated with either 1 nM of the [^32^P]­5′-labeled AP-site-containing substrate (*left panel*), or 2 nM of the [^32^P]­5′-labeled 8oxoG-containing substrate (*right panel*), and 100 mM of either NaBH_4_ or NaCl (as indicated), in the presence of 6.25 μM TH10785, 50 μM TH5487, 6.25 μM compound **30** or 10 μM compound **38**. Products were resolved by SDS–PAGE and visualized by autoradiography.

### Changes in Active Site Structure

The precise mechanism by which bifunctional glycosylases catalyze β-elimination is not completely understood. Nevertheless, it is agreed that deprotonation at the C2’ of the Schiff-base intermediate is a key step. In addition to Lys249, another key catalytic residue shared by bifunctional glycosylases is an aspartic acid — Asp268 in OGG1. This residue likely stabilizes the developing negative charge on 8-oxoG during base excision, but may play a role in the ensuing elimination.^87^

Comparison of the cocrystal structures of mOGG1 with **30**, TH10785 (Figure S9A,B), the inhibitor TH5487 (Figure S9C,D), and **1** (Figure S10) revealed little overall change in the active site structure with most amino acids occupying the same position. However, the absence of the DNA substrate presumed to influence active-site structure prevents more concrete conclusions.

### Conserved Water Molecule

A conserved water molecule held in place by Asp45, has been proposed as the base responsible for C2’ deprotonation in a related glycosylase, endonuclease III (Nth).^88^ Notably, Nth performs β-elimination orders of magnitude faster than OGG1, and does not retain the excised nucleobase in the active site. These mechanistic features raise the possibility of a similar catalytic strategy in tryptamine ORCA-mediated β-elimination by OGG1 facilitated by a network of water molecules stabilized by the ORCA as observed in the mOGG1-**30** crystal structure (Figure 3B).

Consistent with this hypothesis, the water network lies in close proximity to the catalytic residues Lys249 and Asp268. Further support includes the loss of activity observed when the ethylamine chain was removed (**15**), shortened (**39**), extended (**40**) or replaced by an alcohol (**47**). In contrast, amine monomethylation (**46**) increased potency. Together, these results suggest that both the charged nature, and precise positioning of the ammonium moiety are critical for activation, potentially by maintaining the position of the catalytic water molecule within the active site.

### Molecular Dynamics

OGG1-ORCA cocrystal structures were instrumental in guiding virtual docking studies during hit identification and lead optimization. However, their relevance with regards to the catalytic mechanism is limited by the absence of DNA, which may alter the catalytic pocket and ligand binding interactions. To obtain a more representative model of the OGG1-ORCA-DNA complex, we computationally replaced 8-oxoG with ligands **30** or **38** in the NaBH_4_-trapped Schiff-base OGG1–DNA–8-oxoG complex (PDB: 1HU0). We then performed molecular dynamics (MD) simulations, over 200 ns in duplicate using AMBER20.^90^

The clustered pose of the OGG1–DNA–**30** complex following MD simulations revealed a “flipped” ligand orientation (Figure 6A) compared to the OGG1-**30** crystal structure (Figure 3B). The hydroxy group was directed away from Asn150, while the amine–Asp268 salt bridge persisted (Figure 3B). This challenges the SAR rationale that led to compounds **30** and **38**.

**Figure 6:**
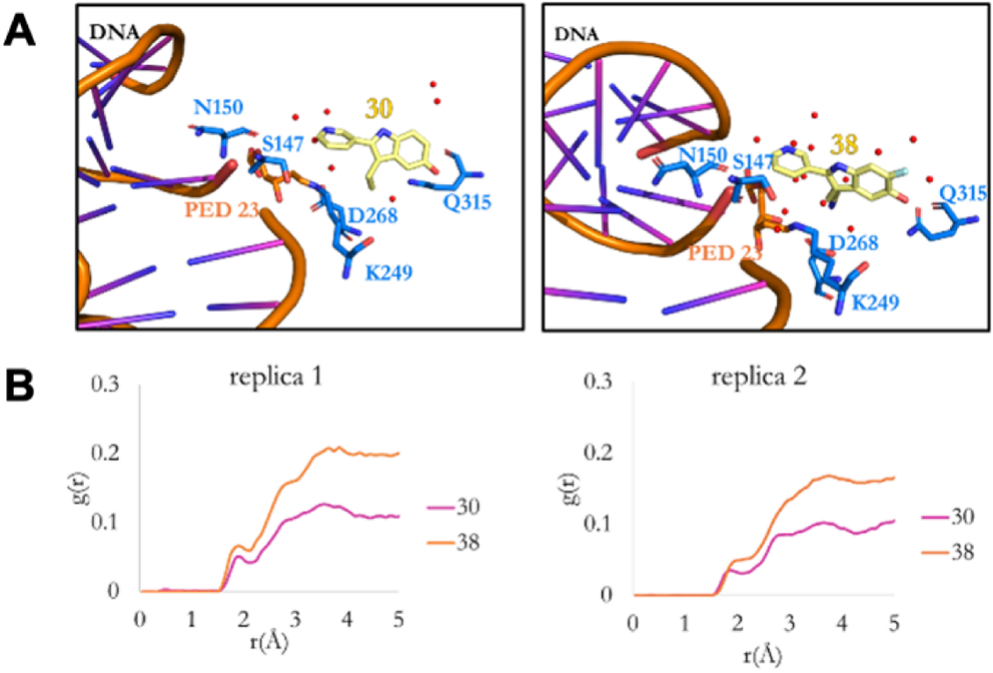
(A) Binding modes of ligands **30** and **38** obtained from clustering analysis of MD trajectories of OGG1:DNA:**30** and OGG1:DNA:**38** systems. Water molecules are colored in red. (B) Calculated RDF profiles calculated for ligand-O_water_ pairs.

Root mean square deviation (RMSD) values for DNA and protein backbones remained within 2.5 Å (Figure S11), and no secondary-structure changes were apparent by clustered geometry inspection. Root mean square fluctuation (RMSF) analysis showed most active-site residues were flexible except the ligand-binding residues Ser147, Asn150, Lys249, Asp268, and Gln315, which were more rigid (Figure S12). Both ligands retained conformations close to their docked poses (Figure S13), and RMSF profiles were similar, indicating that residue flexibility differences are unlikely to explain catalytic variation.

As in the cocrystal structures, multiple water molecules occupied the active site. Radial distribution function (RDF) analysis revealed greater hydration for **38** than for **30**, with peaks at ∼ 2 Å and ∼ 3 Å corresponding to the first and second hydration shells (Figure 6B). Waters within 5 Å of the ligand averaged up to 17 for **38** versus 12 for **30** (Table S5), enabling more extensive water-mediated hydrogen-bond networks with Ser147, Asn150, Lys249, Asp268, and Gln315 (Figure 6A). This enhanced hydration correlates with **38**’s superior β-elimination potency and supports a water-mediated catalytic mechanism.

## Conclusion

We identified a novel class of serotonin-based small-molecule organocatalytic switches of OGG1-catalyzed cleavage of AP sites with improved potency, pharmacokinetic properties, and a distinct mechanism compared to previously reported ORCAs. Computational screening revealed 2-pyrid-3-yltryptamine (**1**) as a hit compound (AC_50_ = 4.1 μM). Guided by X-ray crystallography, molecular modeling, and enabled by Pd-catalyzed Larock chemistry, hit-to-lead optimization yielded the 5-OH derivative **30** (AC_50_ = 0.58 μM) and 5-OH-6-F derivative **38** (AC_50_ = 0.040 μM) over three rounds of optimization. 3-Unsubstituted 2-pyridylindoles also showed ORCA activity but remained less potent than optimized serotonin derivatives. Notably, compound **30** displayed improved pharmacokinetic properties over the benchmark ORCA TH10785, including enhanced solubility (>300 μM), metabolic stability (>3.5 h in human microsomes), and superior lipophilic efficiency, although active efflux remains a limitation for future optimization.

Mechanistically, serotonin ORCAs promote β-elimination of AP-sites by OGG1. Yet, experimental data suggest a distinct activation mode from the previously proposed base-assisted catalysis mechanism. Lack of activity rescue in Lys249 mutants and lack of influence on native Schiff-base formation argue against tryptamine/AP-site Schiff-base formation. Competitive binding as well as crystallographic, SAR studies and MD simulations likewise disfavor an allosteric mechanism. Instead, the data support active-site binding of ORCAs in the 8-oxoG pocket. Once bound, ORCAs stabilize a network of water molecules to trigger β-elimination in an analogous manner to the bifunctional glycosylase Nth.

While this study advances understanding of OGG1 activation by serotonin-derived ORCAs, further mechanistic investigations will be required to fully elucidate their mode of action. Nevertheless, given the growing evidence for the therapeutic potential of ORCAs in reducing oxidative DNA damage,^67,72^ and attenuating fibrosis in MASH,^71^ the compounds reported herein provide valuable chemical tools for further investigations of OGG1 activation in a range of oxidative stress-driven diseases.

## Experimental Section

### Chemistry General Methods

Reagents and solvents were obtained from commercial suppliers and were not purified further unless specified. Dry solvents (THF, CH_2_Cl_2_, Et_2_O, DMF) were provided by a PureSolv SPS-400–5 solvent purification system. Reactions were carried out in standard borosilicate glassware, 2 mL microwave vials with septum caps, or Pyrex culture tubes sealed with a phenolic cap with a PTFE liner. Room temperature was approximately 18–20 °C. Reactions at high temperature were heated using either a DrySyn metal heating bath, a sand bath, or a silicone oil bath. Reactions at 0 °C were performed using and ice/water bath, reactions at – 5 °C were performed using an ice/brine mixture, reactions at – 78 °C were performed using dry ice/acetone baths, reactions at – 84 °C were performed using liquid nitrogen/ethyl acetate baths.

^1^H, ^13^C, and ^19^F (with ^1^H decoupling) NMR spectra recorded at 400, 101, and 376 MHz, respectively, were recorded on a Bruker AVII 400 (BBFO probe). ^1^H, ^13^C, and ^19^F (with ^1^H decoupling) NMR spectra recorded at 500, 126, and 377 MHz, respectively, were recorded on either a Bruker AVIII-HD 500 (BBFO probe) or a Bruker AVIII 500 (BBFO+ probe). ^1^H, and ^13^C NMR spectra recorded at 600 and 151 MHz, respectively, were recorded on a Bruker AVIII 600 (TXI probe). ^1^H, and ^13^C NMR spectra recorded at 700 and 176 MHz, respectively, were recorded on a Bruker AVIII-HD 700 (Prodigy TCI probe). All spectra were recorded at room temperature with the deuterated solvents used as a lock for spectra and internal reference (CDCl_3_: ^1^H, 7.26 ppm; ^13^C, 77.16 ppm; (CD_3_)_2_CO: ^1^H, 2.05 ppm, ^13^C, 29.8 ppm; (CD_3_)_2_SO: ^1^H 2.50 ppm, ^13^C 39.5 ppm; CD_3_OD: ^1^H 3.31 ppm, ^13^C 49.0 ppm). NMR spectra are reported as follows: chemical shift/ppm (multiplicity, coupling constant(s), number of nuclei). Multiplicity given as bs (broad singlet), s (singlet), d (doublet), t (triplet), q (quartet), p (pentet), h (hextet), m (multiplet), and combinations thereof. Signals which overlap with one another are described as multiplets. Note that rotameric behavior was observed in some ^1^H and ^13^C NMR spectra.

IR spectra were recorded using a Shimadzu IT Affinity-1 Fourier transform IR spectrophotometer with a Specac Quest ATR (diamond puck). The spectra were recorded as specified in the procedure as films, solids, or as neat liquids. Transmittance was recorded with maximal absorption wavenumbers given as cm^–1^.

High resolution mass spectrometry (HRMS) spectra were recorded on a Bruker micrOTOF benchtop ESI with either positive or negative electrospray ionization or EI using a Thermo Mat 900XP, Double Focusing Hi-resolution mass spectrometer at the University of Edinburgh mass spectrometry facility (SIRCAMS).

TLC was carried out using Merck aluminum-backed silica plates coated with F254 fluorescent indicator, analyzed under UV light, and developed using aqueous KMnO_4_, ethanolic vanillin, or *n*-butanol ninhydrin solutions where appropriate. Column chromatography was performed using silica gel (40–62 μm, Fluorochem), or in a Biotage SP1 MPLC system using Fisher Chemical silica gel 60 Å. Preparative high-performance liquid chromatography (HPLC) was performed on a Gilson system using Waters C18 OBD 5 μm column (30 × 75 mm) with water buffer (either 0.1% aq. TFA, or 50 mM aq. NH_4_HCO_3_) and acetonitrile as mobile phases using a flow rate of 45 mL/min. Compound purity was measured by HPLC analysis performed on the Agilent 1260 Infinity II LC system coupled to single quadrupole MS Agilent LC/MSD XT. The separation was achieved with either an Avantor ACE 3 C8 (3.0 × 50 mm, 3.0 μm), an Avantor ACE Phenyl (3.0 × 50 mm, 3.0 μm), or a Waters XBridge C18 (3.0 × 50 mm, 3.5 μm) column with a flow rate of 1 mL/min with a linear gradient of MeCN in either 0.1% aqueous TFA or 10 mM NH_4_CO_3_. All compounds used in biological assays had a > 95% purity as determined by HPLC.

### General Procedure for the Preparation of Compounds 2–6

A Pyrex reaction vial was charged with tryptamine derivative (1.0 equiv), RuPhos Pd G1 methyl *t*-butyl ether adduct (5.0 mol %), K_2_CO_3_ (2.0 equiv), 3-bromopyridine (1.5 equiv) and DMA (400 μL per 0.10 mmol of tryptamine). The vial was flushed with N_2_, sealed, and heated to 120 °C for 24 h. After cooling to RT, the reaction mixture was diluted with EtOAc (5 mL per 0.10 mmol of tryptamine) and filtered through a plug of Celite. The volatiles were removed under reduced pressure, and the residue was purified by preparative HPLC.

#### 2-(5-Methyl-2-(pyridin-3-yl)-1H-indol-3-yl)­ethan-1-amine (**2**)

Prepared according to General Procedure A from 5-methyltryptamine hydrochloride (21.1 mg, 1.00 equiv., 0.100 mmol) and K_2_CO_3_ (41.5 mg, 3.00 equiv., 0.300 mmol). Purified by preparative HPLC (10 to 50% MeCN in 50 mM aq. NH_4_CO_3_) to afford the product as a colorless solid (5.5 mg, 22%). ^1^H NMR (600 MHz, (CD_3_)_2_SO with H_2_SO_4_) δ 11.20 (s, 1H), 8.88 (s, 1H), 8.55 (d, *J* = 5.2 Hz, 1H), 8.12 – 7.99 (m, 1H), 7.53 – 7.48 (m, 1H), 7.40 (d, *J* = 5.2 Hz, 1H), 7.27 (d, *J* = 8.5 Hz, 1H), 6.96 (d, *J* = 8.7 Hz, 1H), 3.25 – 3.18 (m, 1H), 2.96 – 2.85 (m, 3H), 2.40 (s, 3H).^13^C NMR (151 MHz, (CD_3_)_2_SO with H_2_SO_4_) δ 144.8, 140.4, 140.3, 135.8, 132.2, 129.0, 128.7, 128.7, 128.2, 125.9, 119.1, 112.4, 111.0, 39.0, 22.8, 21.8. HRMS (ESI) *m*/*z* calcd. for [M + H]^+^ (C_16_H_18_N_3_): 252.1495, found: 252.1495.

#### 2-(5-Methoxy-2-(pyridin-3-yl)-1H-indol-3-yl)­ethan-1-amine, TFA Salt (**3**)

Prepared according to General Procedure A from 5-methoxytryptamine (19.0 mg, 1.00 equiv., 0.100 mmol). Purified by preparative HPLC (0 to 50% MeCN in 0.1% aq. TFA) to afford the product as a beige solid (6.4 mg, 13%). ^1^H NMR (600 MHz, (CD_3_)_2_SO) δ 11.36 (s, 1H), 8.87 (s, 1H), 8.63 (d, *J* = 5.0 Hz, 1H), 8.07 (dt, *J* = 8.3, 2.0 Hz, 1H), 7.86 (s, 3H), 7.60 (dd, *J* = 8.2, 5.0 Hz, 1H), 7.31 (d, *J* = 9.1 Hz, 1H), 7.14 (d, *J* = 2.5 Hz, 1H), 6.83 (dd, *J* = 9.1, 2.1 Hz, 1H), 3.81 (s, 3H), 3.14 – 3.04 (m, 4H).^13^C NMR (151 MHz, (CD_3_)_2_SO) δ 153.6, 147.6, 135.9, 132.2, 131.5, 128.8, 128.5, 124.1, 112.6, 112.3, 107.9, 100.3, 55.5, 40.0, 22.7. HRMS (ESI) *m*/*z* calcd. for [M + H]^+^ (C_16_H_18_N_3_O_1_): 268.1444, found: 268.1460.

#### 2-(5-Chloro-2-(pyridin-3-yl)-1H-indol-3-yl)­ethan-1-amine (**4**)

Prepared according to General Procedure A from 5-chlorotryptamine hydrochloride (57.8 mg, 1.00 equiv., 0.250 mmol). Purified by preparative HPLC (0 to 50% MeCN in 50 mM aq. NH_4_CO_3_) to afford the product as a beige solid (1.7 mg, 2.5%).^1^H NMR (600 MHz, (CD_3_)_2_SO) δ 11.56 (s, 1H), 8.89 (d, *J* = 2.4 Hz, 1H), 8.59 (d, *J* = 4.5 Hz, 1H), 8.08 (d, *J* = 8.7 Hz, 1H), 7.67 (d, *J* = 2.1 Hz, 1H), 7.54 (dd, *J* = 8.2, 5.0 Hz, 1H), 7.39 (d, *J* = 8.7 Hz, 1H), 7.13 (dd, *J* = 8.7, 2.1 Hz, 1H), 2.97 – 2.78 (m, 4H).^13^C NMR (151 MHz, (CD_3_)_2_SO) δ 148.4 (2C), 135.2, 134.7, 133.0, 129.8, 128.5, 123.8, 123.5, 121.9, 118.2, 112.8, 111.1, 42.7, 28.4. HRMS (ESI) *m*/*z* calcd. for [M + H]^+^ (C_15_H_15_^35^Cl_1_N_3_): 272.0949, found: 272.0939.

#### 2-(7-Methyl-2-(pyridin-3-yl)-1H-indol-3-yl)­ethan-1-amine, TFA salt (**5**)

Prepared according to General Procedure A from 7-methyltryptamine (17.4 mg, 1.00 equiv., 0.100 mmol). Purified by preparative HPLC (10 to 50% MeCN in 0.1% aq. TFA) to afford the product as an off-white solid (11.4 mg, 22%). ^1^H NMR (600 MHz, (CD_3_)_2_SO) δ 11.26 (s, 1H), 8.92 (s, 1H), 8.68 (d, *J* = 5.2 Hz, 1H), 8.16 (dt, *J* = 8.2, 2.0 Hz, 1H), 7.89 (s, 3H), 7.66 (dd, *J* = 8.2, 5.2 Hz, 1H), 7.49 (d, *J* = 8.2 Hz, 1H), 7.06 – 6.91 (m, 2H), 3.16 – 3.00 (m, 4H), 2.51 (s, 3H). ^13^C NMR (151 MHz, (CD_3_)_2_SO) δ 147.7, 147.2, 137.3, 136.0, 131.6, 128.9, 127.7, 124.2, 122.9, 121.1, 119.6, 116.1, 108.7, 22.7, 16.9. Note: overlap of *C*H_2_NH_2_ signal with residual solvent signal at 39.5 ppm. HRMS (ESI) *m*/*z* calcd. for [M + H]^+^ (C_16_H_18_N_3_): 252.1495, found: 252.1503.

#### 2-Amino-3-(2-(pyridin-3-yl)-1H-indol-3-yl)­propanoic Acid, TFA Salt (**6**)

Prepared according to General Procedure A from *DL*-tryptophan (20.4 mg, 1.00 equiv., 0.100 mmol). Purified by preparative HPLC (0 to 40% MeCN in 0.1% aq. TFA) to afford the product as a beige solid (1.0 mg, 2%). ^1^H NMR (600 MHz, (CD_3_)_2_SO with H_2_SO_4_) δ 9.06 (s, 1H), 8.90 (d, *J* = 6.2 Hz, 1H), 8.74 (d, *J* = 8.6 Hz, 1H), 8.19 (dd, *J* = 8.4, 6.2 Hz, 1H), 8.13 (d, *J* = 5.5 Hz, 3H), 7.66 (d, *J* = 8.3 Hz, 1H), 7.46 (d, *J* = 8.3 Hz, 1H), 7.19 (t, *J* = 7.5 Hz, 1H), 7.07 (t, *J* = 7.6 Hz, 1H), 3.99 – 3.93 (m, 1H), 3.45 (dd, *J* = 14.9, 7.5 Hz, 1H), 3.38 (dd, *J* = 15.1, 7.4 Hz, 1H).^13^C NMR (151 MHz, (CD_3_)_2_SO with H_2_SO_4_) δ 171.0, 145.9, 141.2, 140.8, 137.3, 132.3, 130.3, 128.2, 128.1, 123.8, 120.4, 119.7, 112.6, 108.6, 53.1, 25.9. HRMS (ESI) *m*/*z* calcd. for [M + H]^+^ (C_16_H_16_N_3_O_2_): 282.1237, found: 282.1254.

### General Procedure B for the Preparation of Compounds 7–9

A Pyrex reaction vial was charged with tryptamine derivative (1.0 equiv), Pd­(OAc)_2_ (15 mol %), AgBF_4_ (2.0 equiv), iodobenzene (1.5 equiv) and DMA (600 μL per 0.10 mmol of tryptamine). The vial was flushed with N_2_, sealed, and heated to 90 °C for 24 h. After cooling to RT, the reaction mixture was diluted with EtOAc (5 mL per 0.10 mmol of tryptamine) and filtered through a plug of Celite. Volatiles were removed under reduced pressure, and the residue was purified by column chromatography (silica gel) or preparative HPLC.

#### 2-Phenyltryptamine (**7**)

Prepared according to General Procedure B from tryptamine (34 mg, 1.0 equiv., 0.21 mmol). Product was purified by column chromatography (0 to 15% MeOH in CH_2_Cl_2_ + 1% NH_4_OH 37% aq. solution) to afford the product as a brown solid (2.9 mg, 6%).^1^H NMR (500 MHz, (CD_3_)_2_SO) δ 11.17 (s, 1H), 7.67 (d, *J* = 6.9 Hz, 2H), 7.59 (d, *J* = 7.7 Hz, 1H), 7.51 (t, *J* = 7.7 Hz, 2H), 7.40 – 7.33 (m, 2H), 7.10 (t, *J* = 7.5 Hz, 1H), 7.01 (t, *J* = 7.3 Hz, 1H), 2.99 – 2.93 (m, 2H), 2.92 – 2.81 (m, 2H). ^13^C NMR (126 MHz, (CD_3_)_2_SO) δ 136.0, 134.5, 132.9, 128.8, 128.7, 127.8, 127.3, 121.5, 118.7, 118.7, 111.2, 109.4, 42.4, 28.1. Spectral data were consistent with those reported in the literature.^91^

#### 5-Hydroxy-2-phenyltryptamine (**8**)

Prepared according to General Procedure B from 5-hydroxytryptamine (17.6 mg, 1.00 equiv., 0.100 mmol). Product was purified by preparative HPLC (30 to 80% MeCN in 50 mM aq. NH_4_CO_3_) to afford the product as a dark brown solid (2.8 mg, 11%). ^1^H NMR (500 MHz, CD_3_OD) δ 7.63 – 7.57 (m, 2H), 7.47 (t, *J* = 7.8 Hz, 2H), 7.38 – 7.31 (m, 1H), 7.21 (d, *J* = 8.7 Hz, 1H), 6.97 (d, *J* = 2.3 Hz, 1H), 6.71 (dd, *J* = 8.6, 2.3 Hz, 1H), 3.11 – 3.05 (m, 2H), 3.02 – 2.96 (m, 2H). ^13^C NMR (126 MHz, CD_3_OD) δ 151.6, 137.5, 134.8, 132.7, 130.9, 129.8, 129.1, 128.5, 112.9, 112.7, 108.4, 103.5, 42.4, 27.2. HRMS (ESI) *m*/*z* calcd. for [M + H]^+^ (C_16_H_17_N_2_O_1_): 253.1335, found: 253.1348.

#### 6-Fluoro-2-phenyltryptamine TFA Salt (**9**)

Prepared according to General Procedure B from 6-fluorotryptamine hydrochloride (21.5 mg, 1.00 equiv., 0.100 mmol). Product was purified by preparative HPLC (30 to 70% MeCN in 0.1% aq. TFA) to afford the product as a brown solid (13.9 mg, 39%). ^1^H NMR (600 MHz, (CD_3_)_2_SO) δ 11.48 (s, 1H), 8.00 (s, 3H), 7.64 – 7.61 (m, 3H), 7.52 (t, *J* = 7.5 Hz, 2H), 7.42 (t, *J* = 7.4 Hz, 1H), 7.14 (dd, *J* = 9.9, 2.4 Hz, 1H), 6.93 (td, *J* = 9.4, 2.4 Hz, 1H), 3.17 – 3.11 (m, 2H), 3.09 – 3.03 (m, 2H). ^13^C NMR (151 MHz, (CD_3_)_2_SO) δ, 159.2 (d, ^1^ *J*_*CF*_ = 235.5 Hz), 135.9 (d, ^3^ *J*_*CF*_ = 12.8 Hz),135.7, 132.2, 128.9, 127.8, 127.7, 125.2, 119.4 (d, ^3^ *J*_*CF*_ = 10.2 Hz), 107.5 (d, ^2^ *J*_*CF*_ = 24.2 Hz), 106.8, 97.3 (d, ^2^ *J*_*CF*_ = 25.6 Hz), 39.3, 22.8. HRMS (ESI) *m*/*z* calcd. for [M + H]^+^ (C_16_H_16_F_1_N_2_): 255.1292, found: 255.1304.

### General Procedure C for the Preparation of Compounds 10–13, 24

A microwave vial under air was charged with substituted phenylhydrazine hydrochloride (1.50 equiv., 0.750 mmol), KOAc (73.6 mg, 1.50 equiv., 0.750 mmol), 3-acetylpyridine (67 μL, 1.0 equiv., 0.50 mmol), and EtOH (0.75 mL). The vial was sealed, and the mixture was stirred at 80 °C in a microwave reactor for 5 min. The mixture was then directly transferred to a Pyrex reaction tube containing preheated polyphosphoric acid (approximately 1 to 1.5 g) at 110 °C. The mixture was stirred at this temperature for 90 min. After cooling to RT, the mixture was partitioned between water (5 mL) and CH_2_Cl_2_ (5 mL). The aqueous phase was further extracted with CH_2_Cl_2_ (3 × 15 mL). The combined organic phases were dried over anhydrous MgSO_4_, filtered, and volatiles were removed under reduced pressure. The residue was purified by preparative HPLC or column chromatography (silica gel).

#### 2-(Pyridin-3-yl)-1H-indole (**10**)

Prepared according to General Procedure C from 3-acetylpyridine (110 μL, 1.00 equiv., 1.00 mmol) and phenylhydrazine (108 μL, 1.10 equiv., 1.10 mmol). Product was purified by column chromatography (30 to 55% EtOAc in hexane) to afford the product as a beige solid (21.7 mg, 11%).^1^H NMR (500 MHz, CDCl_3_) δ 8.98 (d, *J* = 2.3 Hz, 1H), 8.79 (s, 1H), 8.56 (dd, *J* = 4.8, 1.6 Hz, 1H), 7.95 (dt, *J* = 8.1, 2.0 Hz, 1H), 7.66 (d, *J* = 7.9 Hz, 1H), 7.42 (d, *J* = 8.1 Hz, 1H), 7.37 (dd, *J* = 8.0, 4.8 Hz, 1H), 7.23 (t, *J* = 7.5 Hz, 1H), 7.15 (t, *J* = 7.4 Hz, 1H), 6.90 (d, *J* = 2.1 Hz, 1H).^13^C NMR (126 MHz, CDCl_3_) δ 148.5, 146.5, 137.4, 134.6, 132.6, 129.1, 128.7, 124.0, 123.2, 121.0, 120.7, 111.3, 101.4. Spectral data were consistent with those reported in literature.^92^

#### 5-Methyl-2-(pyridin-3-yl)-1H-indole, TFA Salt (**11**)

Prepared according to General Procedure C from 4-methylphenylhydrazine hydrochloride (119 mg, 1.50 equiv., 0.750 mmol). Product was purified by preparative HPLC (20 to 50% MeCN in 0.1% aq. TFA) to afford the product as a light brown solid (22.4 mg, 14%).^1^H NMR (600 MHz, (CD_3_)_2_SO) δ 9.25 (s, 1H), 8.67 (d, *J* = 5.8 Hz, 1H), 8.63 (dt, *J* = 8.6, 1.6 Hz, 1H), 7.84 (dd, *J* = 8.6, 5.6 Hz, 1H), 7.36 (s, 1H), 7.35 (d, *J* = 8.7 Hz, 1H), 7.11 (d, *J* = 2.2 Hz, 1H), 7.01 (dd, *J* = 8.5, 1.6 Hz, 1H), 2.37 (s, 3H). ^13^C NMR (151 MHz, (CD_3_)_2_SO) δ 143.0, 141.3, 136.7, 136.1, 132.5, 130.4, 128.6, 128.5, 126.0, 124.7, 120.2, 111.4, 101.3, 21.2. HRMS (ESI) *m*/*z* calcd. for [M + H]^+^ (C_14_H_13_N_2_): 209.1073, found: 209.1070.

#### 5-Fluoro-2-(pyridin-3-yl)-1H-indole, TFA Salt (**12**)

Prepared according to General Procedure C from 4-fluorophenylhydrazine hydrochloride (122 mg, 1.50 equiv., 0.750 mmol). Product was purified by preparative HPLC (20 to 50% MeCN in 0.1% aq. TFA) to afford the product as a red-brown solid (5.2 mg, 3%). ^1^H NMR (600 MHz, (CD_3_)_2_SO) δ 11.89 (s, 1H), 9.18 (s, 1H), 8.62 (d, *J* = 5.4 Hz, 1H), 8.43 (d, *J* = 9.1 Hz, 1H), 7.69 (dd, *J* = 8.4, 5.3 Hz, 1H), 7.44 (dd, *J* = 9.2, 4.5 Hz, 1H), 7.35 (dd, *J* = 9.8, 2.6 Hz, 1H), 7.12 (d, *J* = 2.3 Hz, 1H), 7.01 (td, *J* = 9.3, 2.6 Hz, 1H).^13^C NMR (151 MHz, (CD_3_)_2_SO) δ 157.3 (d, ^1^ *J*_*CF*_ = 232.9 Hz), 146.0, 144.0, 135.4, 134.6, 134.2, 128.9, 128.5 (d, ^3^ *J*_*CF*_ = 10.6 Hz), 125.0, 112.6 (d, ^3^ *J*_*CF*_ = 9.8 Hz), 110.8 (d, ^2^ *J*_*CF*_ = 26.2 Hz), 104.9 (d, ^2^ *J*_*CF*_ = 23.3 Hz), 100.9. HRMS (ESI) *m*/*z* calcd. for [M + H]^+^ (C_13_H_10_F_1_N_2_): 213.0823, found: 213.0820.

#### 5-Bromo-2-(pyridin-3-yl)-1H-indole, TFA Salt (**13**)

Prepared according to General Procedure C from 4-bromophenylhydrazine hydrochloride (168 mg, 1.50 equiv., 0.750 mmol). Product was purified by preparative HPLC (20 to 50% MeCN in 0.1% aq. TFA) to afford the product as a brown solid (2.1 mg, 1%). ^1^H NMR (400 MHz, (CD_3_)_2_SO) δ 11.90 (s, 1H), 9.10 (d, *J* = 2.5 Hz, 1H), 8.53 (dd, *J* = 4.7, 1.6 Hz, 1H), 8.22 (ddd, *J* = 8.0, 2.4, 1.6 Hz, 1H), 7.75 (d, *J* = 1.9 Hz, 1H), 7.50 (ddd, *J* = 8.0, 4.8, 0.9 Hz, 1H), 7.39 (d, *J* = 8.6 Hz, 1H), 7.24 (dd, *J* = 8.6, 2.0 Hz, 1H), 7.03 (s, 1H). ^13^C NMR (151 MHz, (CD_3_)_2_SO) δ 148.6, 146.4, 136.1, 136.0, 132.3, 130.3, 127.6, 124.5, 124.0, 122.4, 113.4, 112.1, 99.4. HRMS (ESI) *m*/*z* calcd. for [M + H]^+^ (C_13_H_10_^79^Br_1_N_2_): 273.0022, found: 273.0044.

#### (2-Pyridin-4-yl)-1H-indole (**24**)

Prepared according to General Procedure C from 4-acetylpyridine (111 μL, 1.00 equiv., 1.00 mmol) and phenylhydrazine (108 μL, 1.10 equiv., 1.10 mmol). Product was purified by column chromatography (30 to 55% EtOAc in hexane) to afford the product as a beige solid (19.2 mg, 10%). Note: rotary evaporator bath kept at 30 °C to avoid decomposition. ^1^H NMR (500 MHz, CD_3_OD) δ 8.51 (d, *J* = 5.9 Hz, 2H), 7.80 – 7.74 (m, 2H), 7.58 (dt, *J* = 8.0, 1.0 Hz, 1H), 7.43 (dd, *J* = 8.2, 1.0 Hz, 1H), 7.18 (ddd, *J* = 8.2, 7.0, 1.1 Hz, 1H), 7.11 (d, *J* = 0.9 Hz, 1H), 7.05 (ddd, *J* = 8.0, 7.0, 1.0 Hz, 1H).^13^C NMR (126 MHz, CD_3_OD) δ 150.4, 142.4, 139.6, 135.5, 130.0, 124.4, 122.0, 121.1, 120.6, 112.5, 103.5. Spectral data were consistent with those reported in literature.^93^

#### 5-Methoxy-2-(pyridin-3-yl)-1H-indole, TFA Salt (**14**)

A Pyrex reaction tube was charged with *N*-Boc-5-methoxy-1*H*-indole-2-boronic acid (150 mg, 1.00 equiv., 0.51 mmol), 3-bromopyridine (50 μL, 1.0 equiv., 0.52 mmol,), Pd­(PPh_3_)_4_ (27.3 mg, 5.0 mol %, 0.024 mmol), Na_2_CO_3_ (133 mg, 2.50 equiv., 1.25 mmol), and a 1,4-dioxane/water mixture (2:1, 3.0 mL). The vial was flushed with N_2_, sealed, and the mixture was stirred at 60 °C for 18 h. After cooling to RT, volatiles were removed under reduced pressure, and CH_2_Cl_2_ (5.0 mL) was added, followed by TFA (2.0 mL). The mixture was stirred at RT for 2 h, after which sat. NaHCO_3_ solution (10 mL) was added. The mixture was extracted with CH_2_Cl_2_ (2 × 15 mL), the combined organic phases were dried over anhydrous MgSO_4_, filtered, and volatiles were removed under reduced pressure. The residue was purified by preparative HPLC (20 to 50% MeCN in 0.1% aq. TFA) to afford the product as an off-white solid (71 mg, 41%). ^1^H NMR (400 MHz, (CD_3_)_2_SO) δ 11.69 (s, 1H), 9.18 (d, *J* = 2.3 Hz, 1H), 8.61 (dd, *J* = 5.1, 1.5 Hz, 1H), 8.50 (dt, *J* = 8.3, 1.8 Hz, 1H), 7.75 (dd, *J* = 8.2, 5.1 Hz, 1H), 7.34 (d, *J* = 8.8 Hz, 1H), 7.10 – 7.05 (m, 2H), 6.82 (dd, *J* = 8.8, 2.4 Hz, 1H), 3.77 (s, 3H). ^13^C NMR (151 MHz, (CD_3_)_2_SO) δ 153.9, 143.5, 141.8, 136.1, 133.1, 132.9, 130.1, 128.7, 125.7, 113.5, 112.4, 101.7, 101.3, 55.3. HRMS (ESI) *m*/*z* calcd. for [M + H]^+^ (C_14_H_13_N_2_O_1_): 225.1022, found: 225.1033.

#### 2-(Pyridin-3-yl)-1H-indol-5-ol (**15**)

A round-bottom flask under air was charged with 5-methoxy-2-(pyridin-3-yl)-1*H*-indole (68 mg, 1.0 equiv., 0.20 mmol), and HBr (1.0 mL, 33% in AcOH). The solution was heated 70 °C for 2 min and was then allowed to cool to RT, and was stirred for 18 h. The volatiles were removed under reduced pressure, and the residue was purified by preparative HPLC (10 to 50% MeCN in 50 mM aq. NH_4_CO_3_) to afford the product as a light brown solid (12 mg, 29%). ^1^H NMR (600 MHz, (CD_3_)_2_SO) δ 11.36 (s, 1H), 9.05 (s, 1H), 8.74 (s, 1H), 8.46 (d, *J* = 4.9 Hz, 1H), 8.15 (d, *J* = 8.9 Hz, 1H), 7.45 (dd, *J* = 8.4, 5.0 Hz, 1H), 7.21 (d, *J* = 9.1 Hz, 1H), 6.87 – 6.83 (m, 2H), 6.66 (dd, *J* = 8.9, 2.3 Hz, 1H). ^13^C NMR (151 MHz, (CD_3_)_2_SO) δ 151.1, 147.9, 146.1, 134.7, 132.0, 131.7, 129.2, 128.3, 123.9, 112.7, 111.8, 103.9, 99.1. HRMS (ESI) *m*/*z* calcd. for [M + H]^+^ (C_13_H_11_N_2_O_1_): 211.0866, found: 211.0861.

### General Procedure D for the Preparation of Compounds 16–23

A Pyrex reaction tube was charged with *N*-Boc-1*H*-indole-2-boronic acid (52 mg, 1.0 equiv., 0.20 mmol), aryl halide (1.0 equiv., 0.20 mmol), Pd­(PPh_3_)_4_ (12 mg, 5.0 mol %, 0.010 mmol,), Na_2_CO_3_ (55 mg, 2.5 equiv., 0.50 mmol), and a 1,4-dioxane/water mixture (2:1, 1.5 mL). The vial was flushed with N_2_, sealed, and the mixture was stirred at 100 °C for 18 h. After cooling to RT, volatiles were removed under reduced pressure, and the residue was purified by MPLC (0 to 30% MeOH in CH_2_Cl_2_). The Boc-protected indole was dissolved in CH_2_Cl_2_/TFA (1:1, 2.0 mL), and the mixture was stirred at RT for 2 h. Volatiles were removed under reduced pressure to afford the product as the TFA salt.

#### 2-(5-Fluoropyridin-3-yl)-1H-indole, TFA Salt (**16**)

Prepared according to General Procedure D from 3-bromo-5-fluoropyridine (35.2 mg, 1.00 equiv., 0.200 mmol) to afford the product as a brown solid (28.1 mg, 43%).^1^H NMR (600 MHz, (CD_3_)_2_SO) δ 11.75 (s, 1H), 9.01 (s, 1H), 8.50 (d, *J* = 2.9 Hz, 1H), 8.19 (dt, *J* = 10.5, 2.3 Hz, 1H), 7.58 (d, *J* = 8.3 Hz, 1H), 7.44 (d, *J* = 8.4 Hz, 1H), 7.18 – 7.12 (m, 2H), 7.04 (t, *J* = 7.6 Hz, 1H).^13^C NMR (151 MHz, (CD_3_)_2_SO) δ 159.5 (d, ^1^ *J*_CF_= 253.5 Hz), 142.4, 137.5, 135.8 (d, ^2^ *J*_*CF*_ = 22.9 Hz), 133.1, 130.1, 128.3, 122.7, 120.6, 119.8, 118.7 (d, ^2^ *J*_*CF*_ = 19.5 Hz), 111.6, 101.3. HRMS (ESI) *m*/*z* calcd. for [M + H]^+^ (C_13_H_10_F_1_N_2_): 213.0823, found: 213.0820.

#### 2-(6-Fluoropyridin-3-yl)-1H-indole, TFA Salt (**17**)

Prepared according to General Procedure D from 5-bromo-2-fluoropyridine (35.2 mg, 1.00 equiv., 0.200 mmol) to afford the product as a brown solid (26.9 mg, 41%).^1^H NMR (600 MHz, (CD_3_)_2_SO) δ 11.69 (s, 1H), 8.75 (d, *J* = 2.7 Hz, 1H), 8.42 (td, *J* = 8.2, 2.7 Hz, 1H), 7.56 (d, *J* = 8.2 Hz, 1H), 7.43 (d, *J* = 8.3 Hz, 1H), 7.30 (dd, *J* = 8.8, 2.7 Hz, 1H), 7.14 (t, *J* = 7.7 Hz, 1H), 7.05 – 6.98 (m, 2H).^13^C NMR (151 MHz, (CD_3_)_2_SO) δ 162.2 (d, ^1^ *J*_CF_= 236.2 Hz), 143.8 (d, ^3^ *J*_*CF*_ = 15.3 Hz), 138.4 (d, ^3^ *J*_*CF*_ = 7.8 Hz), 137.3, 133.5, 128.4, 126.9 (d, ^4^ *J*_*CF*_ = 4.4 Hz), 122.2, 120.3, 119.7, 111.4, 109.90 (d, ^2^ *J*_*CF*_ = 38.0 Hz), 99.9. HRMS (ESI) *m*/*z* calcd. for [M + H]^+^ (C_13_H_10_F_1_N_2_): 213.0823, found: 213.0836.

#### (1H-Indol-2-yl)­pyridin-3-amine, TFA Salt (**18**)

Prepared according to General Procedure D from 3-bromo-5-aminopyridine (34.6 mg, 1.00 equiv., 0.200 mmol) to afford the product as a beige solid (21.9 mg, 34%). ^1^H NMR (600 MHz, (CD_3_)_2_SO) δ 8.47 (s, 1H), 7.97 (s, 1H), 7.89 (s, 1H), 7.60 (d, *J* = 8.2 Hz, 1H), 7.45 (d, *J* = 8.3 Hz, 1H), 7.18 (t, *J* = 7.7 Hz, 1H), 7.08 (d, *J* = 2.2 Hz, 1H), 7.05 (t, *J* = 7.7 Hz, 1H). ^13^C NMR (151 MHz, (CD_3_)_2_SO) δ 147.5, 137.6, 132.3, 131.5, 128.1, 126.0, 125.7, 123.0, 121.2, 120.8, 120.0, 111.8, 101.8. HRMS (ESI) *m*/*z* calcd. for [M + H]^+^ (C_13_H_12_N_3_): 210.1026, found: 210.1027.

#### 5-(1H-Indol-2-yl)­pyridin-2-amine, TFA Salt (**19**)

Prepared according to General Procedure D from 5-bromo-2-aminopyridine (34.7 mg, 1.00 equiv., 0.200 mmol). To afford the product as a light brown solid (11.0 mg, 17%). ^1^H NMR (600 MHz, (CD_3_)_2_SO) δ 11.59 (s, 1H), 8.39 (d, *J* = 2.3 Hz, 1H), 8.34 (dd, *J* = 9.4, 2.2 Hz, 1H), 7.53 (d, *J* = 8.3 Hz, 1H), 7.39 (d, *J* = 8.3 Hz, 1H), 7.12 (t, *J* = 7.7 Hz, 1H), 7.05 – 6.99 (m, 2H), 6.89 (d, *J* = 2.3 Hz, 1H).^13^C NMR (151 MHz, (CD_3_)_2_SO) δ 153.6, 140.3, 137.1, 132.9, 132.4, 128.3, 122.0, 120.1, 119.6, 117.7, 113.4, 111.2, 99.1. HRMS (ESI) *m*/*z* calcd. for [M + H]^+^ (C_13_H_11_N_3_): 210.1026, found: 210.1034.

#### 5-(1H-Indol-2-yl)­pyridin-3-ol, TFA Salt (**20**)

Prepared according to General Procedure D from 3-bromo-5-hydroxypyridine (34.9 mg, 1.00 equiv., 0.200 mmol) to afford the product as a beige solid (18.1 mg, 28%). ^1^H NMR (600 MHz, (CD_3_)_2_SO) δ 11.73 (s, 1H), 8.69 (s, 1H), 8.16 (s, 1H), 7.85 (s, 1H), 7.58 (d, *J* = 8.3 Hz, 1H), 7.43 (d, *J* = 8.4 Hz, 1H), 7.16 (t, *J* = 7.7 Hz, 1H), 7.08 (d, *J* = 2.2 Hz, 1H), 7.04 (t, *J* = 7.6 Hz, 1H). ^13^C NMR (151 MHz, (CD_3_)_2_SO) δ 154.9, 137.5, 134.4, 133.3, 133.2, 130.4, 128.2, 122.6, 121.0, 120.5, 119.8, 111.6, 101.0. HRMS (ESI) *m*/*z* calcd. for [M + H]^+^ (C_13_H_10_N_2_O_1_): 211.0866, found: 211.0879.

#### 5-(1H-Indol-2-yl)­pyridin-2-ol, TFA Salt (**21**)

Prepared according to General Procedure D from 2-hydroxy-5-iodopyridine (44.3 mg, 1.00 equiv., 0.200 mmol) to afford the product as a dark green solid (21.9 mg, 34%). ^1^H NMR (600 MHz, (CD_3_)_2_SO) δ 11.34 (s, 1H), 7.95 (dd, *J* = 9.6, 2.6 Hz, 1H), 7.91 (d, *J* = 2.7 Hz, 1H), 7.46 (d, *J* = 8.3 Hz, 1H), 7.33 (d, *J* = 8.4 Hz, 1H), 7.05 (t, *J* = 7.7 Hz, 1H), 6.96 (t, *J* = 7.6 Hz, 1H), 6.68 (d, *J* = 2.2 Hz, 1H), 6.46 (d, *J* = 9.7 Hz, 1H). ^13^C NMR (151 MHz, (CD_3_)_2_SO) δ 161.6, 139.0, 136.8, 134.6, 131.1, 128.6, 121.2, 120.4, 119.5, 119.3, 111.2, 110.9, 97.3. HRMS (ESI) *m*/*z* calcd. for [M + H]^+^ (C_13_H_10_N_2_O_1_): 211.0866, found: 211.0867.

#### 2-(6-Chloropyridin-3-yl)-1H-indole, TFA Salt (**22**)

Prepared according to General Procedure D from 5-bromo-2-chloropyridine (38.5 mg, 1.00 equiv., 0.200 mmol) to afford the product as a brown solid (8.6 mg, 13%). ^1^H NMR (600 MHz, CDCl_3_) δ 8.68 (d, *J* = 2.7 Hz, 1H), 8.54 (s, 1H), 7.89 (dd, *J* = 8.5, 2.7 Hz, 1H), 7.64 (d, *J* = 8.5 Hz, 1H), 7.41 (d, *J* = 8.3 Hz, 1H), 7.38 (d, *J* = 8.8 Hz, 1H), 7.24 (t, *J* = 7.7 Hz, 1H), 7.15 (t, *J* = 7.8 Hz, 1H), 6.87 (d, *J* = 2.3 Hz, 1H).^13^C NMR (151 MHz, CDCl_3_) δ 150.2, 146.0, 137.4, 135.3, 133.3, 129.0, 127.7, 124.7, 123.5, 121.1, 120.9, 111.3, 102.0. HRMS (ESI) *m*/*z* calcd. for [M + H]^+^ (C_13_H_10_^35^Cl_1_N_2_): 229.0527, found: 229.0541

#### 2-(6-Methylpyridin-3-yl)-1H-indole, TFA Salt (**23**)

Prepared according to General Procedure D from 5-bromo-2-methylpyridine (34.3 mg, 1.00 equiv., 0.200 mmol) to afford the product as a light brown solid (11.2 mg, 19%).^1^H NMR (600 MHz, (CD_3_)_2_SO) δ 11.80 (s, 1H), 9.09 (d, *J* = 2.5 Hz, 1H), 8.54 – 8.49 (m, 1H), 7.69 (d, *J* = 9.0 Hz, 1H), 7.58 (d, *J* = 8.4 Hz, 1H), 7.44 (d, *J* = 8.5 Hz, 1H), 7.19 – 7.12 (m, 2H), 7.04 (t, *J* = 7.6 Hz, 1H), 2.62 (s, 3H). ^13^C NMR (151 MHz, (CD_3_)_2_SO) δ 153.9, 140.9, 137.5, 136.7, 132.9, 128.3, 127.4, 125.7, 122.6, 120.5, 119.9, 111.5, 100.9, 21.3. HRMS (ESI) *m*/*z* calcd. for [M + H]^+^ (C_14_H_13_N_2_): 209.1073, found: 209.1083.

### General Procedure E for the Preparation of Compounds 25–27

A Pyrex reaction tube was charged with *N*-Boc-1*H*-indole-2-boronic acid (26 mg, 1.0 equiv., 0.10 mmol), aryl halide (1.0 equiv., 0.10 mmol), Pd­(OAc)_2_ (1.1 mg, 5.0 mol %, 5.0 μmol), XPhos (4.8 mg, 10 mol %, 10 μmol,), K_2_CO_3_ (28 mg, 2.0 equiv., 0.20 mmol), and a 1,4-dioxane/water mixture (2:1, 0.60 mL). The vial was flushed with N_2_, sealed, and the mixture was stirred at 60 °C for 18 h. After cooling to RT, the mixture was diluted with CH_2_Cl_2_ (5 mL), filtered through a plug of Celite, and volatiles were removed under reduced pressure. The residue was dissolved in CH_2_Cl_2_ (5 mL) and TFA (2 mL). The mixture was stirred at RT for 2 h, after which sat. aq. NaHCO_3_ (10 mL) was added. The mixture was extracted with CH_2_Cl_2_ (2 × 15 mL), and the combined organic phases were dried over anhydrous MgSO_4_, filtered, and volatiles were removed under reduced pressure. The residue was purified by MPLC (silica gel) or preparative HPLC.

#### 2-(Pyrimidin-5-yl)-1H-indole (**25**)

Prepared according to General Procedure E from 5-bromopyrimidine (15.9 mg, 1.00 equiv., 0.100 mmol). Purified by preparative HPLC (10 to 50% MeCN in 50 mM aq. NH_4_CO_3_) to afford the product as a tan solid (6.0 mg, 31%).^1^H NMR (600 MHz, (CD_3_)_2_SO) δ 9.29 (s, 2H), 9.10 (s, 1H), 7.59 (d, *J* = 8.3 Hz, 1H), 7.46 (d, *J* = 8.4 Hz, 1H), 7.19 – 7.15 (m, 2H), 7.05 (t, *J* = 7.6 Hz, 1H). ^13^C NMR (151 MHz, (CD_3_)_2_SO) δ 156.7, 152.9, 137.6, 131.2, 128.2, 126.4, 122.7, 120.6, 119.9, 111.6, 101.1. Spectral data were consistent with those reported in literature.^94^

#### 5-(1H-Indol-2-yl)-1H-pyrrolo­[2,3-*b*]­pyridine (**26**)

Prepared according to General Procedure E from 5-bromo-1*H*-pyrrolo-[2,3-*b*]­pyridine (19.7 mg, 1.00 equiv., 0.100 mmol). Purified by MPLC (0 to 35% MeOH in CH_2_Cl_2_) to afford the product as a light brown solid (4.4 mg, 19%).^1^H NMR (600 MHz, (CD_3_)_2_SO) δ 11.56 (s, 1H), 8.77 (d, *J* = 2.2 Hz, 1H), 8.41 (d, *J* = 2.2 Hz, 1H), 7.54 – 7.51 (m, 2H), 7.41 (d, *J* = 8.4 Hz, 1H), 7.08 (t, *J* = 7.7 Hz, 1H), 7.00 (t, *J* = 7.6 Hz, 1H), 6.89 (d, *J* = 2.2 Hz, 1H), 6.53 (dd, *J* = 3.4, 1.8 Hz, 1H).^13^C NMR (151 MHz, (CD_3_)_2_SO) δ 147.7, 140.4, 137.0, 136.9, 128.8, 127.2, 124.4, 121.1, 120.7, 119.7, 119.7, 119.3, 111.1, 100.2, 97.7. LCMS found *m*/*z* 234.

#### 3-(1H-Indol-2-yl)­quinoline (**27**)

Prepared according to General Procedure E from 3-bromoquinoline (20.8 mg, 1.00 equiv., 0.100 mmol). Purified by column chromatography (0 to 30% MeOH in CH_2_Cl_2_) to afford the product as a brown solid (6.7 mg, 27%). ^1^H NMR (400 MHz, (CD_3_)_2_SO) δ 11.85 (s, 1H), 9.48 (d, *J* = 2.3 Hz, 1H), 8.77 (d, *J* = 2.3 Hz, 1H), 8.05 (d, *J* = 8.4 Hz, 1H), 8.01 (dd, *J* = 8.1, 1.4 Hz, 1H), 7.77 (ddd, *J* = 8.5, 6.9, 1.5 Hz, 1H), 7.67 (ddd, *J* = 8.1, 6.7, 1.2 Hz, 1H), 7.61 (d, *J* = 7.9 Hz, 1H), 7.47 (dd, *J* = 8.2, 0.7 Hz, 1H), 7.23 (d, *J* = 2.1 Hz, 1H), 7.21 – 7.10 (m, 1H), 7.09 – 6.99 (m, 1H). ^13^C NMR (151 MHz, (CD_3_)_2_SO) δ 148.5, 146.6, 137.5, 134.7, 129.8, 129.3, 128.8, 128.5, 128.1, 127.6, 127.3, 125.5, 122.3, 120.4, 119.7, 111.4, 100.5. HRMS (ESI) *m*/*z* calcd. for [M + H]^+^ (C_17_H_13_N_2_): 245.1073, found: 245.1082.

#### 3-(Benzofuran-2-yl)­pyridine (**28**)

A Pyrex reaction tube was charged with benzofuran (54 μL, 1.0 equiv., 0.50 mmol), 3-iodopyridine (153 mg, 1.50 equiv., 0.750 mmol), *trans*-bis­(acetato)­bis­[*ortho*-(di-*ortho*-tolylphosphino)­benzyldipalladi-um­(II) (46 mg, 10 mol %, 0.050 mmol), AgOAc (125 mg, 1.50 equiv., 0.750 mmol,), and TFA (2.5 mL). The vial was sealed and heated to 70 °C for 24 h. After cooling to RT, the reaction mixture was diluted with CH_2_Cl_2_ (25 mL). The mixture was further cooled to 0 °C and was quenched by slow addition of aq. NaOH (5 M, 15 mL) with stirring. The phases were separated, and the aqueous phase was extracted with CH_2_Cl_2_ (2 × 25 mL). The combined organic phases were dried over anhydrous MgSO_4_, filtered, and volatiles were removed under reduced pressure. The residue was purified by column chromatography (silica gel, 0 to 10% MeCN in CH_2_Cl_2_) to afford the product as a white solid (62 mg, 64%).^1^H NMR (500 MHz, CDCl_3_) δ 9.09 (s, 1H), 8.56 (d, *J* = 4.8 Hz, 1H), 8.10 – 8.04 (m, 1H), 7.58 (d, *J* = 7.1 Hz, 1H), 7.52 (d, *J* = 8.0 Hz, 1H), 7.35 – 7.27 (m, 2H), 7.24 (t, *J* = 7.1 Hz, 1H), 7.08 – 7.03 (m, 1H). ^13^C NMR (126 MHz, CDCl_3_) δ 155.1, 152.9, 149.3, 146.4, 131.8, 128.8, 126.6, 125.0, 123.6, 123.3, 121.2, 111.3, 102.8. Spectral data were consistent with those reported in literature.^95^

### General Procedure F for the Preparation of Compounds 29a–29v

In an Ar-filled glovebox, a flame-dried Schlenk flask was charged with Pd­(P^t^Bu_3_)_2_ (5.1 mg, 5.0 mol %, 10 μmol). A separate, oven-dried microwave vial was charged with the acetamide (1.33 equiv., 0.266 mmol) and the alkyne (1.00 equiv., 0.200 mmol) after which the vial was sealed, evacuated, and refilled with N_2_ three times. Dry DMF (600 μL) and Cy_2_NMe (53.3 μL, 2.50 equiv., 0.250 mmol, purged with N_2_ for 5 min prior to use) were added to the microwave vial, after which the contents were transferred to the Schlenk vial. The mixture was then stirred at 80 °C for 24 h. After cooling to RT, volatiles were removed under reduced pressure to produce a residue which was dissolved in EtOAc (10 mL) and washed with 10% aq. LiCl (2 × 10 mL). The organic phase was dried over anhydrous Na_2_SO_4_, filtered, and volatiles were removed under reduced pressure. The residue was dissolved in THF/H_2_O (1.0 mL, 3:1), LiOH (24 mg, 5.0 equiv., 0.50 mmol), was added, and the mixture was stirred at 45 °C for 2.5 h. Volatiles were removed under reduced pressure and the residue was purified by column chromatography (silica gel).

#### *tert*-Butyl (2-(5-Methoxy-2-(pyridin-3-yl)-1H-indol-3-yl)­ethyl)­carbamate (**29a**)

Prepared according to General Procedure F from *tert*-butyl (4-(pyridin-3-yl)­but-3-yn-1-yl)­carbamate **S2a** (36.9 mg, 1.00 equiv., 0.150 mmol) and *N*-(2-iodo-4-methoxyphenyl)­acetamide **S1a** (58.1 mg, 1.33 equiv., 0.200 mmol). Purified by column chromatography (20 to 55% EtOAc in hexane) to afford the product as an orange solid (30.4 mg, 55%). ^1^H NMR (500 MHz, CDCl_3_) δ 8.77 (s, 1H), 8.75 (s, 1H), 8.54 (d, *J* = 4.9 Hz, 1H), 7.88 (d, *J* = 7.9 Hz, 1H), 7.38 – 7.32 (m, 1H), 7.29 (d, *J* = 8.7 Hz, 1H), 7.09 (s, 1H), 6.89 (dd, *J* = 8.8, 2.4 Hz, 1H), 4.76 (bs, 1H), 3.88 (s, 3H), 3.44 (q, *J* = 6.7 Hz, 2H), 3.02 (t, *J* = 7.0 Hz, 2H), 1.39 (s, 9H).^13^C NMR (126 MHz, CDCl_3_) δ 156.0, 154.5, 148.6, 148.4, 135.5, 132.7, 131.7, 129.4, 129.3, 123.9, 113.4, 112.1, 111.4, 101.1, 79.3, 56.1, 41.1, 28.5, 25.5. υ_max_ (film) 3277, 2928, 1684, 1456, 1157, 735 cm^–1^. HRMS (ESI) *m*/*z* calcd. for [M + H]^+^ (C_21_H_26_N_3_O_3_): 368.1969, found: 368.1963.

#### *tert*-Butyl (2-(6-(Benzyloxy)-2-(pyridin-3-yl)-1H-indol-3-yl)­ethyl)­carbamate (**29b**)

Prepared according to General Procedure F from *tert*-butyl (4-(pyridin-3-yl)­but-3-yn-1-yl)­carbamate **S2a** (36.9 mg, 1.00 equiv., 0.150 mmol) and *N*-(5-(benzyloxy)-2-iodophenyl)­acetamide **S1b** (73.4 mg, 1.33 equiv., 0.200 mmol). Purified by column chromatography (10 to 50% EtOAc in hexane) then repurified by column chromatography (0 to 1% MeOH in CH_2_Cl_2_) to afford the product as an orange solid (9.6 mg, 19%). ^1^H NMR (500 MHz, CDCl_3_) δ 8.78 (s, 1H), 8.55 (s, 1H), 8.38 (s, 1H), 7.87 (d, *J* = 7.7 Hz, 1H), 7.54 (d, *J* = 8.6 Hz, 1H), 7.46 (d, *J* = 7.1 Hz, 2H), 7.41 – 7.30 (m, 4H), 6.94 (d, *J* = 2.2 Hz, 1H), 6.91 (dd, *J* = 8.6, 2.2 Hz, 1H), 5.12 (s, 2H), 4.72 – 4.65 (m, 1H), 3.44 (q, *J* = 7.0 Hz, 2H), 3.04 (t, *J* = 7.2 Hz, 2H), 1.40 (s, 9H). ^13^C NMR (126 MHz, CDCl_3_) δ 156.4, 156.0, 148.5, 148.3, 137.4, 137.2, 135.2, 130.7, 129.4, 128.7, 128.0, 127.6, 123.9, 123.7, 120.3, 111.8, 111.0, 96.0, 79.3, 70.7, 41.3, 28.5, 25.5. υ_max_ (film) 2924, 1709, 1593, 1454, 1366, 1163 cm^–1^. HRMS (ESI) *m*/*z* calcd. for [M + H]^+^ (C_27_H_30_N_3_O_3_): 444.2282, found: 444.2278.

#### *tert*-Butyl (2-(7-(Benzyloxy)-2-(pyridin-3-yl)-1H-indol-3-yl)­ethyl)­carbamate (**29c**)

Prepared according to General Procedure F from *tert*-butyl (4-(pyridin-3-yl)­but-3-yn-1-yl)­carbamate **S2a** (36.9 mg, 1.00 equiv., 0.150 mmol) and *N*-(2-(benzyloxy)-6-iodophenyl)­acetamide **S1d** (73.4 mg, 1.33 equiv., 0.200 mmol). Purified by column chromatography (20 to 45% EtOAc in hexane) to afford a mixture of C2-pyridyl and C3-pyridyl products (85:15) as a light brown solid (48.2 mg, 72%). ^1^H NMR (500 MHz, CDCl_3_) δ 8.77 (s, 2H), 8.55 (s, 1H), 7.88 (d, *J* = 7.9 Hz, 1H), 7.50 – 7.46 (m, 2H), 7.43 – 7.32 (m, 4H), 7.30 (d, *J* = 8.1 Hz, 1H), 7.07 (t, *J* = 7.9 Hz, 1H), 6.78 (d, *J* = 7.7 Hz, 1H), 5.21 (s, 2H), 4.73 – 4.66 (m, 1H), 3.46 (q, *J* = 6.8 Hz, 2H), 3.05 (t, *J* = 7.1 Hz, 2H), 1.40 (s, 9H).^13^C NMR (126 MHz, CDCl_3_) δ 156.0, 149.0, 148.7, 145.4, 136.9, 135.6, 131.8, 130.4, 128.8, 128.7, 128.4, 128.1, 127.1, 123.7, 120.6, 112.5, 112.0, 104.1, 79.2, 70.5, 41.2, 28.5, 25.6. υ_max_ (film) 3262, 2974 1686, 1506, 1161, 777, 731 cm^–1^. HRMS (ESI) *m*/*z* calcd. for [M + H]^+^ (C_27_H_30_N_3_O_3_): 444.2282, found: 444.2273.

#### *tert*-Butyl (2-(4-Methoxy-2-(pyridin-3-yl)-1H-indol-3-yl)­ethyl)­carbamate (**29d**)

Prepared according to General Procedure F from *tert*-butyl (4-(pyridine-3-yl)­but-3-yn-1-yl)­carbamate **S2a** (49.3 mg, 1.00 equiv., 0.200 mmol) and *N*-(2-iodo-3-methoxyphenyl)­acetamide **S1e** (77.4 mg, 1.33 equiv., 0.266 mmol). Purified by column chromatography (40 to 60% EtOAc in hexane) to afford the product as a gray solid (9.9 mg, 13%). ^1^H NMR (500 MHz, CDCl_3_) δ 8.81 (bs, 1H), 8.60 (bs, 1H), 8.23 (s, 1H), 7.93 (d, *J* = 6.1 Hz, 1H), 7.40 (bs, 1H), 7.15 (t, *J* = 8.0 Hz, 1H), 7.02 (d, *J* = 8.1 Hz, 1H), 6.55 (d, *J* = 7.8 Hz, 1H), 4.89 (bs, 1H), 3.97 (s, 3H), 3.54 – 3.47 (m, 2H), 3.11 (t, *J* = 6.8 Hz, 2H), 1.35 (s, 9H).^13^C NMR (126 MHz, CDCl_3_) δ 156.1, 154.8, 149.0, 148.6, 138.0, 136.0, 130.9, 123.9 (2C), 118.7, 112.3, 104.5, 100.2, 78.9, 55.3, 42.4, 28.5, 26.2. υ_max_ (film) 3285, 2926, 1688, 1248, 1167, 1107, 735 cm^–1^. HRMS (ESI) *m*/*z* calcd. for [M + H]^+^ (C_21_H_26_N_3_O_3_): 368.1969, found: 368.1974.

#### *tert*-Butyl (2-(5-Fluoro-2-(pyridin-3-yl)-1H-indol-3-yl)­ethyl)­carbamate (**29e**)

Prepared according to General Procedure F from *tert*-butyl (4-(pyridin-3-yl)­but-3-yn-1-yl)­carbamate **S2a** (49.3 mg, 1.00 equiv., 0.200 mmol) and *N*-(4-fluoro-2-iodophenyl)­acetamide **S 1f** (74.2 mg, 1.33 equiv., 0.266 mmol). Purified by column chromatography (0 to 1.5% MeOH in CH_2_Cl_2_), then repurified by column chromatography (50 to 65% EtOAc in hexane) to afford the product as an off-white solid (11.4 mg, 16%). ^1^H NMR (500 MHz, CDCl_3_) δ 8.80 (s, 1H), 8.63 – 8.58 (m, 2H), 7.90 (d, *J* = 7.9 Hz, 1H), 7.39 (dd, *J* = 7.9, 4.9 Hz, 1H), 7.34 – 7.28 (m, 2H), 6.99 (td, *J* = 9.0, 2.5 Hz, 1H), 4.67 (s, 1H), 3.41 (q, *J* = 6.9 Hz, 2H), 3.01 (t, *J* = 7.1 Hz, 2H), 1.40 (s, 9H). ^13^C NMR (126 MHz, CDCl_3_) δ 158.2 (d, ^1^ *J*_*CF*_ = 235.9 Hz), 156.0, 148.9, 148.8, 135.6, 133.7, 132.9, 129.5 (d, ^3^ *J*_*CF*_ = 7.9 Hz), 129.0, 123.9, 112.0, 111.9, 111.6 (d, *J* = 26.2 Hz), 104.4 (d, ^2^ *J*_*CF*_ = 23.4 Hz), 79.4, 41.2, 28.5, 25.4. ^19^F NMR (377 MHz, CDCl_3_) δ – 123.50. υ_max_ (film) 3350, 2926, 1680, 1171, 1153, 793 cm^–1^. HRMS (ESI) *m*/*z* calcd. for [M + H]^+^ (C_20_H_23_F_1_N_3_O_2_): 356.1769, found: 356.1759.

#### *tert*-Butyl (2-(6-Fluoro-2-(pyridin-3-yl)-1H-indol-3-yl)­ethyl)­carbamate (**29f**)

Prepared according to General Procedure F from *tert*-butyl (4-(pyridin-3-yl)­but-3-yn-1-yl)­carbamate **S2a** (49.3 mg, 1.00 equiv., 0.200 mmol) and *N*-(5-fluoro-2-iodophenyl)­acetamide **S1g** (74.2 mg, 1.33 equiv., 0.266 mmol). Purified by column chromatography (0 to 1.5% MeOH in CH_2_Cl_2_) to afford the product as a light yellow solid (13.8 mg, 19%). ^1^H NMR (500 MHz, CDCl_3_) δ 8.81 (s, 1H), 8.71 (s, 1H), 8.58 (s, 1H), 7.91 (d, *J* = 7.9 Hz, 1H), 7.58 (dd, *J* = 8.8, 5.4 Hz, 1H), 7.43 – 7.37 (m, 1H), 7.09 (dd, *J* = 9.4, 2.3 Hz, 1H), 6.92 (td, *J* = 9.2, 2.3 Hz, 1H), 4.70 (t, *J* = 6.4 Hz, 1H), 3.43 (q, *J* = 6.9 Hz, 2H), 3.04 (t, *J* = 7.2 Hz, 2H), 1.40 (s, 9H). ^13^C NMR (126 MHz, CDCl_3_) δ 160.6 (d, ^1^ *J*_*CF*_ = 239.5 Hz), 156.0, 148.6, 136.5 (d, ^3^ *J*_*CF*_ = 12.6 Hz), 135.5, 132.1, 129.1, 125.6, 124.0, 120.4 (d, ^3^ *J*_*CF*_ = 10.2 Hz), 111.8, 109.0 (d, ^2^ *J*_*CF*_ = 24.6 Hz), 97.6 (d, ^2^ *J*_*CF*_ = 26.0 Hz), 79.4, 41.3, 28.5, 25.5. ^19^F NMR (470 MHz, CDCl_3_) δ – 119.40. υ_max_ (film) 3260, 2976, 1686, 1364, 1248, 1165 cm^–1^. HRMS (ESI) *m*/*z* calcd. for [M + Na]^+^ (C_20_H_22_F_1_N_3_O_2_Na_1_): 378.1588, found: 378.1588.

#### *tert*-Butyl (2-(5-((*tert*-Butoxycarbonyl)­amino)-2-(pyridin-3-yl)-1H-indol-3-yl)­ethyl)­carbamate (**29g**)

Prepared according to General Procedure F from *tert*-butyl (4-(pyridine-3-yl)­but-3-yn-1-yl)­carbamate **S2a** (36.9 mg, 1.00 equiv., 0.150 mmol) and *tert*-butyl (4-acetamido-3-iodophenyl)­carbamate **S1h** (75.0 mg, 1.33 equiv., 0.200 mmol). Purified by column chromatography (0 to 5% MeOH in CH_2_Cl_2_) to afford the product as a light brown powder (19.4 mg, 29%). ^1^H NMR (700 MHz, CDCl_3_) δ 8.83 (s, 1H), 8.77 (s, 1H), 8.58 – 8.45 (m, 1H), 7.88 (d, *J* = 7.8 Hz, 1H), 7.61 (s, 1H), 7.34 (t, *J* = 6.4 Hz, 1H), 7.25 (d, *J* = 8.7 Hz, 1H), 7.16 (s, 1H), 6.64 (s, 1H), 4.75 (s, 1H), 3.43 – 3.36 (m, 2H), 2.98 (t, *J* = 7.2 Hz, 2H), 1.53 (s, 9H), 1.38 (s, 9H). ^13^C NMR (176 MHz, CDCl_3_) δ 156.0, 153.7, 148.6, 148.5, 135.6, 133.4, 132.8, 131.2, 129.3, 129.1, 123.8, 116.8, 111.4, 111.4, 109.8, 80.2, 79.2, 41.1, 28.6, 28.5, 25.4. υ_max_ (film) 3308, 2976, 1686, 1244, 1159 cm^–1^. HRMS (ESI) *m*/*z* calcd. for [M + H]^+^ (C_25_H_33_N_4_O_4_): 453.2496, found: 453.2497.

#### Methyl 3-(2-((*tert*-Butoxycarbonyl)­amino)­ethyl)-2-(pyridin-3-yl)-1H-indole-5-carboxylate (**29h**)

Prepared according to General Procedure F from *tert*-butyl (4-(pyridin-3-yl)­but-3-yn-1-yl)­carbamate **S2a** (49.3 mg, 1.00 equiv., 0.200 mmol) and methyl 4-acetamido-3-iodobenzoate **S1i** (84.9 mg, 1.33 equiv., 0.266 mmol). Purified by column chromatography (0 to 2% MeOH in CH_2_Cl_2_) to afford the product as a light brown solid (8.9 mg, 11%). ^1^H NMR (500 MHz, (CD_3_)_2_CO) δ 10.89 (s, 1H), 8.96 (s, 1H), 8.61 (s, 1H), 8.44 (d, *J* = 1.7 Hz, 1H), 8.16 (d, *J* = 8.0 Hz, 1H), 7.87 (dd, *J* = 8.5, 1.6 Hz, 1H), 7.53 – 7.48 (m, 2H), 6.25 – 6.17 (m, 1H), 3.89 (s, 3H), 3.44 (q, *J* = 6.8 Hz, 2H), 3.18 – 3.12 (m, 2H), 1.39 (s, 9H). ^13^C NMR (126 MHz, (CD_3_)_2_CO) δ 168.2, 156.7, 149.7 (2C), 140.2, 136.1, 134.4, 129.6, 129.5, 124.5, 124.3, 122.6, 122.4, 113.2, 111.9, 78.6, 51.9, 42.1, 28.6, 26.2. υ_max_ (film) 2926, 1699, 1250, 1169, 1105, 772 cm^–1^. HRMS (ESI) *m*/*z* calcd. for [M + Na]^+^ (C_22_H_25_N_3_O_4_Na_1_): 418.1737, found: 418.1738.

#### *tert*-Butyl (2-(6-Fluoro-5-methoxy-2-(pyridin-3-yl)-1H-indol-3-yl)­ethyl)­carbamate (**29i**)

Prepared according to General Procedure F from *tert-*butyl (4-(pyridin-3-yl)­but-3-yn-1-yl)­carbamate **S2a** (49.3 mg, 1.00 equiv., 0.200 mmol) and *N*-(5-fluoro-2-iodo-4-methoxyphenyl)­acetamide **S1j** (82.2 mg, 1.33 equiv., 0.266 mmol). Purified by column chromatography (0 to 1.5% MeOH in CH_2_Cl_2_) to afford the product as a light yellow solid (25.5 mg, 33%). ^1^H NMR (500 MHz, CDCl_3_) δ 8.78 (bs, 1H), 8.64 (s, 1H), 8.57 (bs, 1H), 7.88 (d, *J* = 5.8 Hz, 1H), 7.38 (bs, 1H), 7.17 (d, *J* = 8.1 Hz, 1H), 7.13 (d, *J* = 11.1 Hz, 1H), 4.72 (s, 1H), 3.95 (s, 3H), 3.42 (q, *J* = 5.2 Hz, 2H), 3.02 (t, *J* = 7.1 Hz, 2H), 1.40 (s, 9H). ^13^C NMR (126 MHz, CDCl_3_) δ 156.0, 151.4 (d, ^1^ *J*_*CF*_ = 241.8 Hz), 148.6 (2C), 143.6 (d, ^3^ *J*_*CF*_ = 12.8 Hz), 135.3, 132.2, 130.0 (d, ^3^ *J*_*CF*_ = 11.3 Hz), 129.2, 124.6, 123.9, 111.6, 102.8, 98.9 (d, ^2^ *J*_*CF*_ = 23.4 Hz), 79.4, 57.1, 41.2, 28.5, 25.6. ^19^F NMR (377 MHz, CDCl_3_) δ – 138.32. υ_max_ (film) 3379, 2934, 1686, 1520, 1215, 1169, 1142 cm^–1^. HRMS (ESI) *m*/*z* calcd. for [M + H]^+^ (C_21_H_25_F_1_NO_3_): 386.1875, found: 386.1880.

#### *tert*-Butyl (2-(6-Fluoro-5-methoxy-3-(pyridin-3-yl)-1H-indol-2-yl)­ethyl)­carbamate (**29j**)

Prepared according to General Procedure F from *tert*-butyl (4-(pyridin-3-yl)­but-3-yn-1-yl)­carbamate **S2a** (49.3 mg, 1.00 equiv., 0.200 mmol) and *N*-(5-fluoro-2-iodo-4-methoxyphenyl)­acetamide **S1j** (82.2 mg, 1.33 equiv., 0.266 mmol). Purified by column chromatography (0 to 2% MeOH in CH_2_Cl_2_), then repurified by column chromatography (45 to 60% EtOAc in hexane) to afford the product as a white solid (6.5 mg, 8%). ^1^H NMR (500 MHz, CDCl_3_) δ 9.21 (s, 1H), 8.74 (s, 1H), 8.58 (s, 1H), 7.79 (d, *J* = 7.8 Hz, 1H), 7.43 (bs, 1H), 7.12 (d, *J* = 11.1 Hz, 1H), 7.05 (d, *J* = 8.1 Hz, 1H), 4.82 (s, 1H), 3.87 (s, 3H), 3.49 (q, *J* = 6.5 Hz, 2H), 3.04 (t, *J* = 6.6 Hz, 2H), 1.41 (s, 9H).^13^C NMR (126 MHz, CDCl_3_) δ 156.7, 150.8 (d, ^1^ *J*_*CF*_ = 240.5 Hz), 150.1, 147.1, 143.8 (d, ^2^ *J*_*CF*_ = 12.8 Hz), 136.9, 134.0, 134.0, 129.1 (d, ^3^ *J*_*CF*_ = 11.2 Hz), 124.0, 123.1, 111.4, 102.0, 98.8 (d, ^2^ *J*_*CF*_ = 23.4 Hz), 80.3, 57.1, 39.8, 28.5, 28.0. ^19^F NMR (377 MHz, CDCl_3_) δ – 140.0. υ_max_ (film) 2922, 1682, 1481, 1157, 1128 cm^–1^. HRMS (ESI) *m*/*z* calcd. for [M + H]^+^ (C_21_H_25_F_1_N_3_O_3_): 386.1875, found: 386.1878.

#### *tert*-Butyl ((5-Methoxy-2-(pyridin-3-yl)-1H-indol-3-yl)­methyl)­carbamate (**29k**)

Prepared according to General Procedure F from *tert*-butyl (3-(pyridin-3-yl)­prop-2-yn-1-yl)­carbamate **S2c** (46.5 mg, 1.00 equiv., 0.200 mmol) and *N*-(2-iodo-4-methoxyphenyl)­acetamide **S1a** (77.4 mg, 1.33 equiv., 0.266 mmol). Purified by column chromatography (0 to 2% MeOH in CH_2_Cl_2_) to afford the product as a light pink solid (26.9 mg, 38%). Note: unstable, rotary evaporator bath kept at 30 °C. ^1^H NMR (500 MHz, CDCl_3_) δ 8.79 (s, 1H), 8.63 (d, *J* = 4.8 Hz, 1H), 8.22 (s, 1H), 7.86 (dt, *J* = 7.9, 1.9 Hz, 1H), 7.42 (dd, *J* = 7.9, 4.8 Hz, 1H), 7.31 (d, *J* = 8.8 Hz, 1H), 7.18 (s, 1H), 6.93 (dd, *J* = 8.8, 2.4 Hz, 1H), 4.69 (bs, 1H), 4.56 (d, *J* = 5.1 Hz, 2H), 3.88 (s, 3H), 1.47 (s, 9H). ^13^C NMR (126 MHz, CDCl_3_) δ 155.9, 154.9, 149.2, 148.8, 135.6, 133.4, 131.4, 128.9, 128.5, 123.9, 114.0, 112.1, 111.1, 100.9, 79.5, 56.0, 35.4, 28.6. υ_max_ (film) 2922, 1699, 1506, 1497, 1223, 1163 cm^–1^. HRMS (ESI) *m*/*z* calcd. for [M + H]^+^ (C_20_H_24_N_3_O_3_): 354.1812, found: 354.1813.

#### *tert*-Butyl (3-(5-Methoxy-2-(pyridin-3-yl)-1H-indol-3-yl)­propyl)­carbamate (**29l**)

Prepared according to General Procedure F from *tert*-butyl (5-(pyridin-3-yl)­pent-4-yn-1-yl)­carbamate **S 2d** (46.6 mg, 1.00 equiv., 0.179 mmol) and *N*-(2-iodo-4-methoxyphenyl)­acetamide **S1a** (69.3 mg, 1.33 equiv., 0.238 mmol). Purified by column chromatography (0 to 2% MeOH in CH_2_Cl_2_) to afford the product as a white solid (31.0 mg, 45%). ^1^H NMR (500 MHz, CDCl_3_) δ 8.78 (s, 1H), 8.72 (s, 1H), 8.55 (d, *J* = 4.8 Hz, 1H), 7.81 (dt, *J* = 8.0, 2.0 Hz, 1H), 7.34 (dd, *J* = 8.0, 4.9 Hz, 1H), 7.28 (d, *J* = 8.7 Hz, 1H), 7.03 (d, *J* = 2.4 Hz, 1H), 6.88 (dd, *J* = 8.8, 2.4 Hz, 1H), 4.57 (bs, 1H), 3.87 (s, 3H), 3.13 (q, *J* = 6.7 Hz, 2H), 2.88 – 2.83 (m, 2H), 1.86 (p, *J* = 7.0 Hz, 2H), 1.41 (s, 9H).^13^C NMR (126 MHz, CDCl_3_) δ 156.1, 154.3, 148.8, 148.4, 135.2, 131.8, 131.8, 129.6, 129.3, 123.8, 113.9, 113.1, 112.1, 101.0, 79.2, 56.1, 40.6, 31.0, 28.5, 21.9. υ_max_ (film) 3302, 2932, 1684, 1474, 1163, 1024 cm^–1^. HRMS (ESI) *m*/*z* calcd. for [M + H]^+^ (C_22_H_28_N_3_O_3_): 382.2125, found: 382.2116.

#### *tert*-Butyl (2-(2-(5-Fluoropyridin-3-yl)-5-methoxy-1H-indol-3-yl)­ethyl)­carbamate (**29m**)

Prepared according to General Procedure F from *tert*-butyl (4-(5-fluoropyridin-3-yl)­but-3-yn-1-yl)­carbamate **S 2f** (52.9 mg, 1.00 equiv., 0.200 mmol) and *N*-(2-iodo-4-methoxyphenyl)­acetamide **S1a** (77.4 mg, 1.33 equiv., 0.266 mmol). Purified by column chromatography (0 to 1.5% MeOH in CH_2_Cl_2_) to afford the product as an orange solid (35.0 mg, 45%). ^1^H NMR (500 MHz, CDCl_3_) δ 8.63 (s, 1H), 8.45 (s, 1H), 8.27 (s, 1H), 7.63 (d, *J* = 9.3 Hz, 1H), 7.30 (d, *J* = 8.8 Hz, 1H), 7.10 (s, 1H), 6.92 (dd, *J* = 8.8, 2.4 Hz, 1H), 4.67 (s, 1H), 3.88 (s, 3H), 3.50 – 3.44 (m, 2H), 3.04 (t, *J* = 6.9 Hz, 2H), 1.40 (s, 9H).^13^C NMR (126 MHz, CDCl_3_) δ 159.6 (^1^ *J*_*CF*_ = 259.4 Hz), 156.0, 154.7, 144.5, 137.0 (d, ^2^ *J*_*CF*_ = 23.2 Hz), 131.7, 131.1, 130.7, 129.3, 121.9 (d, ^2^ *J*_*CF*_ = 18.8 Hz), 114.0, 112.5, 112.2, 101.2, 79.5, 56.1, 41.0, 28.5, 25.5. ^19^F NMR (376 MHz, CDCl_3_) δ – 125.76. υ_max_ (film) 3275, 2928, 1684, 1215, 1165 cm^–1^. HRMS (ESI) *m*/*z* calcd. for [M + H]^+^ (C_21_H_25_F_1_N_3_O_3_): 386.18745, found: 386.1880.

#### *tert*-Butyl (2-(5-Methoxy-2-(pyridin-2-yl)-1H-indol-3-yl)­ethyl)­carbamate (**29n**)

Prepared according to General Procedure F from *tert*-butyl (4-(pyridin-2-yl)­but-3-yn-1-yl)­carbamate **S2h** (36.9 mg, 1.00 equiv., 0.150 mmol) and *N*-(2-iodo-4-methoxyphenyl)­acetamide **S1a** (58.1 mg, 1.33 equiv., 0.200 mmol). Purified by column chromatography (20 to 40% EtOAc in hexane) to afford the product as a yellow solid (30.9 mg, 56%). ^1^H NMR (500 MHz, CDCl_3_) δ 9.48 (s, 1H), 8.62 (d, *J* = 4.3 Hz, 1H), 7.85 (d, *J* = 8.2 Hz, 1H), 7.76 (t, *J* = 7.8 Hz, 1H), 7.28 (d, *J* = 8.8 Hz, 1H), 7.19 (t, *J* = 6.0 Hz, 1H), 7.07 (d, *J* = 2.4 Hz, 1H), 6.92 (dd, *J* = 8.8, 2.4 Hz, 1H), 5.26 (bs, 1H), 3.90 (s, 3H), 3.54 (q, *J* = 6.2 Hz, 2H), 3.28 (t, *J* = 6.8 Hz, 2H), 1.44 (s, 9H).^13^C NMR (126 MHz, CDCl_3_) δ 156.3, 154.3, 150.8, 149.4, 137.1, 133.9, 131.0, 130.0, 121.8, 121.1, 114.1, 112.2, 111.9, 100.7, 79.1, 56.0, 41.0, 28.6, 25.6. υ_max_ (film) 3348, 2974, 1684, 1215, 1163, 787 cm^–1^. HRMS (ESI) *m*/*z* calcd. for [M + H]^+^ (C_21_H_26_N_3_O_3_): 368.1969, found:368.1977.

#### *tert*-Butyl (2-(5-Methoxy-2-(pyridin-4-yl)-1H-indol-3-yl)­ethyl)­carbamate (**29o**)

Prepared according to General Procedure F from *tert*-butyl (4-(pyridin-4-yl)­but-3-yn-1-yl)­carbamate **S2i** (52.1 mg, 1.00 equiv., 0.212 mmol) and *N*-(2-iodo-4-methoxyphenyl)­acetamide **S1a** (77.5 mg, 1.26 equiv., 0.266 mmol). Purified by column chromatography (0 to 1.5% MeOH in CH_2_Cl_2_) to afford the product as a yellow solid (38.0 mg, 49%). ^1^H NMR (400 MHz, CDCl_3_) δ 9.22 (s, 1H), 8.55 (bs, 2H), 7.52 (bs, 2H), 7.30 (d, *J* = 8.7 Hz, 1H), 7.05 (s, 1H), 6.91 – 6.87 (m, 1H), 4.83 (s, 1H), 3.85 (s, 3H), 3.47 – 3.38 (m, 2H), 3.09 (t, *J* = 7.1 Hz, 2H), 1.39 (s, 9H).^13^C NMR (101 MHz, CDCl_3_) δ 156.1, 154.5, 149.6, 141.3, 132.4, 132.1, 129.5, 122.0, 114.4, 113.2, 112.5, 100.9, 79.4, 56.0, 41.0, 28.5, 25.7. υ_max_ (film) 3308, 2932, 1684, 1217, 1163 cm^–1^. HRMS (ESI) *m*/*z* calcd. for [M + H]^+^ (C_21_H_26_N_3_O_3_): 368.1969, found: 368.1969.

#### *tert*-Butyl (2-(5-Methoxy-2-(pyrimidin-5-yl)-1H-indol-3-yl)­ethyl)­carbamate (**29p**)

Prepared according to General Procedure F from *tert*-butyl (4-(pyrimidin-5-yl)­but-3-yn-1-yl)­carbamate **S2g** (37.1 mg, 1.00 equiv., 0.150 mmol) and *N*-(2-iodo-4-methoxyphenyl)­acetamide **S1a** (58.1 mg, 1.33 equiv., 0.200 mmol). Purified by column chromatography (0 to 2% MeOH in CH_2_Cl_2_), then repurified by column chromatography (50 to 55% EtOAc in hexane) to afford the product as a brown solid (25.9 mg, 47%). ^1^H NMR (500 MHz, CDCl_3_) δ 9.21 (s, 1H), 8.96 (s, 2H), 8.13 (s, 1H), 7.32 (d, *J* = 8.8 Hz, 1H), 7.12 (s, 1H), 6.95 (dd, *J* = 8.8, 2.4 Hz, 1H), 4.65 (bs, 1H), 3.90 (s, 3H), 3.52 – 3.42 (m, 2H), 3.05 (t, *J* = 7.0 Hz, 2H), 1.40 (s, 9H). ^13^C NMR (126 MHz, CDCl_3_) δ 157.3, 156.2, 156.0, 155.4, 154.8, 132.1, 129.3, 128.9, 127.7, 114.2, 113.0, 112.3, 101.2, 79.5, 56.1, 41.1, 28.5, 25.6. υ_max_ (film) 3298, 2924, 1699, 1456, 1167 cm^–1^. HRMS (ESI) *m*/*z* calcd. for [M + Na]^+^ (C_20_H_24_N_4_O_3_Na_1_): 391.1741, found: 391.1740.

#### *tert*-Butyl (2-(5-Methoxy-3-(pyrimidin-5-yl)-1H-indol-2-yl)­ethyl)­carbamate (**29q**)

Prepared according to General Procedure F from *tert*-butyl (4-(pyrimidin-5-yl)­but-3-yn-1-yl)­carbamate **S2g** (37.1 mg, 1.00 equiv., 0.150 mmol) and *N*-(2-iodo-4-methoxyphenyl)­acetamide **S1a** (58.1 mg, 1.33 equiv., 0.200 mmol). Purified by column chromatography (0 to 2% MeOH in CH_2_Cl_2_) to afford the product as a brown solid (13.3 mg, 24%). ^1^H NMR (500 MHz, CDCl_3_) δ 9.34 (s, 1H), 9.17 (s, 1H), 8.88 (s, 2H), 7.30 (d, *J* = 8.8 Hz, 1H), 7.00 (d, *J* = 2.4 Hz, 1H), 6.88 (dd, *J* = 8.8, 2.4 Hz, 1H), 4.84 (s, 1H), 3.81 (s, 3H), 3.52 (q, *J* = 6.6 Hz, 2H), 3.06 (t, *J* = 6.5 Hz, 2H), 1.42 (s, 9H). ^13^C NMR (126 MHz, CDCl_3_) δ 156.8, 156.2, 155.2, 135.1, 130.8, 130.1, 127.6, 112.7, 112.2, 107.7, 99.8, 80.4, 56.0, 39.7, 28.4, 28.1. υ_max_ (film) 3264, 2928, 1686, 1489, 1474, 1159 cm^–1^. HRMS (ESI) *m*/*z* calcd. for [M + H]^+^ (C_20_H_25_N_4_O_3_): 369.1921, found: 369.1924.

#### *tert*-Butyl (2-(5-Methoxy-2-(thiazol-5-yl)-1H-indol-3-yl)­ethyl)­carbamate (**29r**)

Prepared according to General Procedure F from *tert*-butyl (4-(thiazol-5-yl)­but-3-yn-1-yl)­carbamate **S2j** (50.5 mg, 1.00 equiv., 0.200 mmol) and *N*-(2-iodo-4-methoxyphenyl)­acetamide **S1a** (77.5 mg, 1.26 equiv., 0.266 mmol). Purified by column chromatography (0 to 1.5% MeOH in CH_2_Cl_2_), then repurified by column chromatography (40 to 55% EtOAc in hexane) to afford the product as a brown solid (18.0 mg, 24%). ^1^H NMR (500 MHz, CDCl_3_) δ 8.79 (s, 1H), 8.44 (s, 1H), 8.03 (s, 1H), 7.28 – 7.22 (m, 1H), 7.05 (s, 1H), 6.90 (dd, *J* = 8.8, 2.4 Hz, 1H), 4.70 (s, 1H), 3.87 (s, 3H), 3.44 (q, *J* = 6.7 Hz, 2H), 3.07 (t, *J* = 6.8 Hz, 2H), 1.41 (s, 9H). ^13^C NMR (126 MHz, CDCl_3_) δ 156.0, 154.7, 152.5, 140.3, 131.6, 129.9, 129.4, 126.2, 113.9, 112.7, 111.9, 101.0, 79.4, 56.1, 40.9, 28.5, 25.8. υ_max_ (film) 3277, 2974, 1684, 1215, 1165, 1111 cm^–1^. HRMS (ESI) *m*/*z* calcd. for [M + H]^+^ (C_19_H_24_N_3_O_3_S_1_): 374.1533, found: 374.1522.

#### *tert*-Butyl (2-(5-(Methoxymethoxy)-2-(pyridin-3-yl)-1H-indol-3-yl)­ethyl)­(methyl)­carbamate (**29s**)

Prepared according to General Procedure F from *tert*-butyl methyl­(4-(pyridin-3-yl)­but-3-yn-1-yl)­carbamate **S2b** (52.1 mg, 1.00 equiv., 0.200 mmol) and *N*-(2-iodo-4-(methoxymethoxy)­phenyl)­acetamide **S1k** (85.4 mg, 1.33 equiv., 0.266 mmol). Purified by column chromatography (0 to 2% MeOH in CH_2_Cl_2_), then repurified by column chromatography (50 to 60% EtOAc in hexane) to afford the product as a yellow solid (26.3 mg, 32%). ^1^H NMR (500 MHz, CDCl_3_, mixture of rotamers) δ 8.80 (s, 1H), 8.59 (s, 1H), 8.46 (m, 1H), 8.00 – 7.94 (m, 1H)*, 7.84 – 7.80 (m, 1H)*, 7.41 – 7.35 (m, 1H), 7.32 – 7.27 (m, 2H), 7.00 (dd, *J* = 8.7, 2.3 Hz, 1H), 5.22 (s, 2H), 3.53 (s, 3H), 3.51 – 3.46 (m, 2H), 3.07 – 3.00 (m, 2H), 2.73 (s, 3H), 1.27 (s, 9H). *rotamer signal splitting. ^13^C NMR (126 MHz, CDCl_3_, mixture of rotamers) δ 155.6, 152.0, 149.0, 148.8, 135.6, 132.8, 132.6, 129.6, 129.4, 123.9, 114.5, 111.8, 111.8, 105.7, 96.0, 79.4, 56.0, 49.5, 34.3, 28.3, 23.4. υ_max_ (film) 3248, 2920, 1657, 1150, 1011, 810 cm^–1^. HRMS (ESI) *m*/*z* calcd. for [M + H]^+^ (C_23_H_30_N_3_O_4_): 412.2231, found: 412.2231.

#### 3-(2-((*tert*-Butyldimethylsilyl)­oxy)­ethyl)-5-(methoxymethoxy)-2-(pyridin-3-yl)-1H-indole (**29t**)

Prepared according to General Procedure F from 3-(4-((*tert*-butyldimethylsilyl)­oxy)­but-1-yn-1-yl)­pyridine **S2e** (52.3 mg, 1.00 equiv., 0.200 mmol) and *N*-(2-iodo-4-(methoxymethoxy)­phenyl)­acetamide **S1k** (85.4 mg, 1.33 equiv., 0.266 mmol). Purified by column chromatography (0 to 1.5% MeOH in CH_2_Cl_2_) to afford the product as a yellow solid (34.0 mg, 41%). ^1^H NMR (400 MHz, CDCl_3_) δ 8.85 (s, 1H), 8.72 (s, 1H), 8.59 – 8.52 (m, 1H), 8.03 (d, *J* = 7.9 Hz, 1H), 7.40 – 7.32 (m, 1H), 7.31 – 7.27 (m, 2H), 6.97 (d, *J* = 8.9 Hz, 1H), 5.22 (s, 2H), 3.92 (t, *J* = 7.2 Hz, 2H), 3.53 (t, *J* = 1.7 Hz, 3H), 3.07 (t, *J* = 7.2 Hz, 2H), 0.83 (s, 9H), – 0.05 (s, 6H). ^13^C NMR (176 MHz, CDCl_3_) δ 151.8, 148.7, 148.5, 135.7, 133.0, 132.5, 129.8, 129.4, 123.7, 114.6, 111.8, 111.6, 105.8, 96.0, 63.5, 56.0, 28.4, 26.1, 18.5, – 5.3. υ_max_ (film) 2926, 1464, 1152, 1080, 1005, 833 cm^–1^. HRMS (ESI) *m*/*z* calcd. for [M + Na]^+^ (C_23_H_32_N_2_O_3_Si_1_Na_1_) 435.2074, found: 435.2073.

#### *tert*-Butyl (2-(2-(pyridin-3-yl)-1H-indol-3-yl)­ethyl)­carbamate (**29u**)

Prepared according to General Procedure F from *tert*-butyl (4-(pyridin-3-yl)­but-3-yn-1-yl)­carbamate **S2a** (49.3 mg, 1.00 equiv., 0.200 mmol) and *N*-(2-iodophenyl)­acetamide (69.4 mg, 1.33 equiv., 0.266 mmol). Purified by column chromatography (0 to 1.5% MeOH in CH_2_Cl_2_), then repurified by column chromatography (40 to 55% EtOAc in hexane) to afford the product as a yellow solid (18.7 mg, 28%). ^1^H NMR (500 MHz, CDCl_3_) δ 8.82 (s, 1H), 8.77 (s, 1H), 8.59 – 8.55 (m, 1H), 7.92 (d, *J* = 7.9 Hz, 1H), 7.67 (d, *J* = 7.9 Hz, 1H), 7.41 (d, *J* = 8.1 Hz, 1H), 7.41 – 7.34 (m, 1H), 7.26 – 7.22 (m, 1H), 7.16 (ddd, *J* = 8.0, 7.0, 1.0 Hz, 1H), 4.72 (s, 1H), 3.45 (t, *J* = 7.0 Hz, 2H), 3.07 (t, *J* = 7.1 Hz, 2H), 1.40 (s, 9H). ^13^C NMR (126 MHz, CDCl_3_) δ 156.0, 148.7, 148.5, 136.5, 135.7, 131.8, 129.4, 128.9, 123.9, 123.1, 120.2, 119.5, 111.7, 111.3, 79.3, 41.2, 28.5, 25.5. υ_max_ (film) 3292, 2976, 1686, 1364, 1161 cm^–1^. HRMS (ESI) *m*/*z* calcd. for [M + H]^+^ (C_20_H_24_N_3_O_2_): 338.1863, found: 338.1852.

#### *tert*-Butyl (2-(3-(pyridin-3-yl)-1H-indol-2-yl)­ethyl)­carbamate (**29v**)

Prepared according to General Procedure F from *tert*-butyl (4-(pyridin-3-yl)­but-3-yn-1-yl)­carbamate **S2a** (49.3 mg, 1.00 equiv., 0.200 mmol) and *N*-(2-iodophenyl)­acetamide (69.4 mg, 1.33 equiv., 0.266 mmol). Purified by column chromatography (0 to 1.5% MeOH in CH_2_Cl_2_) to afford the product as a light-brown solid (27.8 mg, 41%). ^1^H NMR (500 MHz, CDCl_3_) δ 9.49 (s, 1H), 8.74 (s, 1H), 8.55 (d, *J* = 4.9 Hz, 1H), 7.82 (dt, *J* = 7.9, 1.8 Hz, 1H), 7.58 (d, *J* = 7.9 Hz, 1H), 7.43 – 7.32 (m, 2H), 7.20 (ddd, *J* = 8.2, 7.0, 1.2 Hz, 1H), 7.13 (td, *J* = 7.5, 1.1 Hz, 1H), 4.91 (s, 1H), 3.48 (t, *J* = 6.8 Hz, 2H), 3.07 (t, *J* = 6.9 Hz, 2H), 1.41 (s, 9H). ^13^C NMR (126 MHz, CDCl_3_) δ 156.5, 150.3, 147.1, 136.9, 135.8, 133.7, 131.6, 127.6, 123.7, 122.2, 120.4, 118.4, 111.5, 111.1, 80.1, 40.0, 28.4, 27.7. υ_max_ (film) 3300, 2976, 1703, 1364, 1250, 1165 cm^–1^. HRMS (ESI) *m*/*z* calcd. for [M + H]^+^ (C_20_H_24_N_3_O_2_) 338.18630, found: 338.1858.

### General Procedure G for the Preparation of Compounds 30–33, 38–45, 48–49, 52–53

A flame-dried round-bottom flask was charged with protected indole (1.00 equiv), 1,2,4,5-tetramethylbenzene (8.00 equiv), and anhydrous CH_2_Cl_2_ (2.5 mL per 0.1 mmol indole). The mixture was cooled to – 84 °C, and BBr_3_ (0.75 M in CH_2_Cl_2_, 8.00 equiv) was added portion-wise over 5 min via syringe. The mixture was allowed to gradually warm to RT and stirred for 16 h. The mixture was diluted with water (∼5 mL per 0.1 mmol of indole, added cautiously) and MeOH (∼5 mL per 0.1 mmol indole). The pH of the aqueous phase was adjusted to >10 by addition of aq. NaOH (2 M). Volatiles were removed under reduced pressure and the residue was purified by column chromatography (silica gel).

#### 3-(2-Aminoethyl)-2-(pyridin-3-yl)-1H-indol-5-ol (**30**)

Prepared according to General Procedure G from *tert*-butyl (2-(5-methoxy-2-(pyridin-3-yl)-1*H*-indol-3-yl)­ethyl)­carbamate **29a** (20.3 mg, 1.00 equiv., 55.2 μmol). Purified by column chromatography (0 to 12% MeOH in CH_2_Cl_2_ + 1% NH_4_OH 37% aq. solution) to afford the product as a dark green solid (9.6 mg, 69%). ^1^H NMR (400 MHz, CD_3_OD) δ 8.78 (s, 1H), 8.53 (d, *J* = 4.8 Hz, 1H), 8.06 (dt, *J* = 8.0, 2.0 Hz, 1H), 7.56 (dd, *J* = 7.9, 4.9 Hz, 1H), 7.25 (d, *J* = 8.7 Hz, 1H), 7.00 (d, *J* = 2.3 Hz, 1H), 6.77 (dd, *J* = 8.7, 2.3 Hz, 1H), 3.20 – 3.05 (m, 4H). ^13^C NMR (101 MHz, CD_3_OD) δ 152.1, 149.1, 148.8, 137.4, 133.6, 133.2, 131.3, 130.4, 125.5, 114.0, 113.1, 108.9, 103.5, 41.5, 25.2. υ_max_ (film) 2922, 2855, 1738, 1711, 1462 cm^–1^. HRMS (ESI) *m*/*z* calcd. for [M + H]^+^ (C_15_H_16_N_3_O_1_): 254.1288, found: 254.1291.

#### 3-(2-Aminoethyl)-2-(pyridin-3-yl)-1H-indol-6-ol (**31**)

Prepared according to General Procedure G from *tert*-butyl (2-(6-(benzyloxy)-2-(pyridin-3-yl)-1*H*-indol-3-yl)­ethyl)­carbamate **29b** (10.2 mg, 1.00 equiv., 23.0 μmol). Purified by column chromatography (8 to 12% MeOH in CH_2_Cl_2_ + 1% NH_4_OH 37% aq. solution) to afford the product as a yellow solid (2.4 mg, 41%). ^1^H NMR (700 MHz, CD_3_OD) δ 8.76 (s, 1H), 8.53 (s, 1H), 8.05 (dt, *J* = 8.0, 1.9 Hz, 1H), 7.57 (dd, *J* = 7.9, 4.9 Hz, 1H), 7.45 (d, *J* = 8.5 Hz, 1H), 6.83 (d, *J* = 2.1 Hz, 1H), 6.69 (dd, *J* = 8.6, 2.1 Hz, 1H), 3.25 – 3.21 (m, 2H), 3.21 – 3.17 (m, 2H). ^13^C NMR (176 MHz, CD_3_OD) δ 155.5, 148.9, 148.5, 139.6, 137.2, 131.5 (2C), 125.6, 123.5, 120.0, 111.3, 108.9, 97.7, 41.1, 24.0. υ_max_ (film) 3229, 2924, 1558, 1541, 1506, 1456 cm^–1^. HRMS (ESI) *m*/*z* calcd. for [M + H]^+^ (C_15_H_16_N_3_O_1_): 254.1288, found: 254.1294.

#### 3-(2-Aminoethyl)-2-(pyridin-3-yl)-1H-indol-7-ol (**32**)

Prepared according to General Procedure G from a mixture of *tert*-butyl (2-(7-(benzyloxy)-2-(pyridin-3-yl)-1*H*-indol-3-yl)­ethyl)­carbamate and *tert*-butyl (2-(7-methoxy-3-(pyridin-3-yl)-1*H*-indol-2-yl)­ethyl)­carbamate (85:15) **29c** (30.6 mg, 1.00 equiv., 69.0 μmol). Purified by column chromatography (8 to 15% MeOH in CH_2_Cl_2_ + 1% NH_4_OH 37% aq. solution) to afford the product as a red-brown solid (10.8 mg, 62%). ^1^H NMR (700 MHz, CD_3_OD) δ 8.80 (d, *J* = 2.3 Hz, 1H), 8.51 (dd, *J* = 5.0, 1.6 Hz, 1H), 8.08 (dt, *J* = 7.8, 2.0 Hz, 1H), 7.55 (dd, *J* = 7.9, 4.8 Hz, 1H), 7.12 (d, *J* = 8.0 Hz, 1H), 6.89 (t, *J* = 7.8 Hz, 1H), 6.59 (d, *J* = 7.5 Hz, 1H), 3.07 (t, *J* = 7.6 Hz, 2H), 2.98 (dd, *J* = 8.9, 6.3 Hz, 2H). ^13^C NMR (176 MHz, CD_3_OD) δ 149.4, 148.5, 145.1, 137.7, 132.2, 131.9, 131.6, 128.5, 125.4, 121.3, 112.1, 111.1, 107.8, 43.0, 28.2. υ_max_ (film) 3329, 2974, 1653, 1456, 1045, 878 cm^–1^. HRMS (ESI) *m*/*z* calcd. for [M + H]^+^ (C_15_H_16_N_3_O_1_): 254.1288, found: 254.1283.

#### 3-(2-Aminoethyl)-2-(pyridin-3-yl)-1H-indol-4-ol (**33**)

Prepared according to General Procedure G from *tert*-butyl (2-(4-methoxy-2-(pyridin-3-yl)-1*H*-indol-3-yl)­ethyl)­carbamate **29d** (8.7 mg, 1.0 equiv., 24 μmol). Purified by column chromatography (4 to 8% MeOH in CH_2_Cl_2_ + 1% NH_4_OH 37% aq. solution) to afford the product as a yellow solid (1.8 mg, 30%). ^1^H NMR (500 MHz, CD_3_OD) δ 8.74 (d, *J* = 2.4 Hz, 1H), 8.54 (dd, *J* = 4.9, 1.6 Hz, 1H), 8.03 (dt, *J* = 7.9, 2.1 Hz, 1H), 7.57 (dd, *J* = 8.0, 5.0 Hz, 1H), 6.97 (t, *J* = 7.8 Hz, 1H), 6.90 (d, *J* = 8.1 Hz, 1H), 6.43 (d, *J* = 7.5 Hz, 1H), 3.28 (s, 4H). ^13^C NMR (126 MHz, CD_3_OD) δ 153.1, 149.4, 148.7, 140.5, 137.8, 131.5, 125.4, 124.8, 110.1, 105.1, 104.2, 43.0, 30.8 note: additional signals observed by (^1^H,^13^C) HMBC at ∼ 131.7 ppm and ∼ 119.5 ppm. υ_max_ (film) 3335, 2924, 2855, 1593, 1456, 1254 cm^–1^. HRMS (ESI) *m*/*z* calcd. for [M + H]^+^ (C_15_H_16_N_3_O_1_): 254.1288, found: 254.1282.

#### 3-(2-Aminoethyl)-6-fluoro-2-(pyridin-3-yl)-1H-indol-5-ol (**38**)

Prepared according to General Procedure G from *tert*-butyl (2-(6-fluoro-5-methoxy-2-(pyridin-3-yl)-1*H*-indol-3-yl)­ethyl)­carbamate **29i** (13.9 mg, 1.00 equiv., 36.1 μmol). Purified by column chromatography (8 to 12% MeOH in CH_2_Cl_2_ + 1% NH_4_OH 37% aq. solution). Further purified by dissolving in minimum volume of DMSO followed by addition of toluene (10 mL). The solution was cooled to – 94 °C in a liquid nitrogen/acetone bath and the precipitate was collected and dried to afford the product as a yellow solid (5.4 mg, 55%). ^1^H NMR (500 MHz, CD_3_OD) δ 8.81 (bs, 1H), 8.60 (bs, 1H), 8.12 (d, *J* = 7.9 Hz, 1H), 7.69 – 7.59 (m, 1H), 7.15 (d, *J* = 8.4 Hz, 1H), 7.14 (d, *J* = 11.0 Hz, 1H), 3.26 – 3.20 (m, 2H), 3.20 – 3.14 (m, 2H).^13^C NMR (126 MHz, CD_3_OD) δ 152.2 (d, ^1^ *J*_*CF*_ = 236.8 Hz), 148.5, 148.4, 141.0 (d, ^2^ *J*_*CF*_ = 15.7 Hz), 138.1, 133.4, 133.3, 131.8 (d, ^3^ *J*_*CF*_ = 11.1 Hz), 125.9, 125.7, 108.5, 106.0 (d, ^3^ *J*_CF_ = 2.5 Hz), 99.3 (d, ^2^ *J*_*CF*_ = 23.6 Hz), 40.9, 23.9.^19^F NMR (377 MHz, CD_3_OD) δ – 142.16. υ_max_ (solid) 3119, 2924, 1466, 1402, 1275, 1028, 806 cm^–1^. HRMS (ESI) *m*/*z* calcd. for [M + H]^+^ (C_15_H_15_F_1_N_3_O_1_): 272.1194, found: 272.1197.

#### 2-(2-Aminoethyl)-6-fluoro-3-(pyridin-3-yl)-1H-indol-5-ol (**52**)

Prepared according to General Procedure G from *tert*-butyl (2-(6-fluoro-5-methoxy-3-(pyridin-3-yl)-1*H*-indol-2-yl)­ethyl)­carbamate **29j** (6.3 mg, 1.0 equiv., 16 μmol). Purified by column chromatography (8 to 10% MeOH in CH_2_Cl_2_ + 1% NH_4_OH 37% aq. solution) to afford the product as a yellow solid (3.0 mg, 68%). ^1^H NMR (500 MHz, CD_3_OD) δ 8.61 (d, *J* = 2.2 Hz, 1H), 8.47 (dd, *J* = 5.0, 1.6 Hz, 1H), 7.93 (dt, *J* = 7.9, 2.0 Hz, 1H), 7.54 (dd, *J* = 7.9, 4.9 Hz, 1H), 7.09 (d, *J* = 11.2 Hz, 1H), 6.98 (d, *J* = 8.4 Hz, 1H), 3.10 – 2.98 (m, 4H). ^13^C NMR (126 MHz, CD_3_OD) δ 151.4 (d, ^1^ *J*_*CF*_ = 235.9 Hz), 150.5, 147.3, 141.2 (d, ^2^ *J* = 15.0 Hz), 138.9, 134.6, 133.7, 130.8 (d, ^3^ *J*_*CF*_ = 10.9 Hz), 125.5, 124.6, 111.4, 105.5 (d, ^3^ *J*_*CF*_ = 2.2 Hz), 99.0 (d, ^2^ *J*_*CF*_ = 23.2 Hz), 41.5, 28.6. ^19^F NMR (376 MHz, CD_3_OD) δ – 143.46. υ_max_ (solid) 2920, 2851, 1472, 1098, 1026, 804 cm^–1^. HRMS (ESI) *m*/*z* calcd. for [M + H]^+^ (C_15_H_15_F_1_N_3_O_1_): 272.1194, found: 272.1195.

#### 3-(Aminomethyl)-2-(pyridin-3-yl)-1H-indol-5-ol (**39**)

Prepared according to General Procedure G from *tert*-butyl ((5-methoxy-2-(pyridin-3-yl)-1*H*-indol-3-yl)­methyl)­carbamate **29k** (10.0 mg, 1.00 equiv., 28.3 μmol). Purified by column chromatography (0 to 14% MeOH in CH_2_Cl_2_ + 1% NH_4_OH 37% aq. solution) to afford the product as a yellow solid (2.7 mg, 40%). ^1^H NMR (700 MHz, CD_3_OD) δ 9.28 – 8.50 (m, 2H), 8.12 (d, *J* = 7.7 Hz, 1H), 7.82 – 7.62 (m, 1H), 7.31 (d, *J* = 8.6 Hz, 1H), 7.11 (d, *J* = 2.2 Hz, 1H), 6.84 (dd, *J* = 8.7, 2.2 Hz, 1H), 4.36 (s, 2H). ^13^C NMR (176 MHz, CD_3_OD) δ 152.8, 149.6, 137.8, 136.3, 132.9, 129.7, 127.2, 123.5, 114.6, 113.3, 105.4, 103.4, 35.1. υ_max_ (film) 3211, 2922, 2856, 1628, 1456, 1217 cm^–1^. HRMS (ESI): [M + H]^+^ *m*/*z* calcd. for (C_14_H_14_N_3_O_1_): 240.1131, found: 240.1132.

#### 3-(3-Aminopropyl)-2-(pyridin-3-yl)-1H-indol-5-ol (**40**)

Prepared according to General Procedure G from *tert*-butyl (3-(5-methoxy-2-(pyridin-3-yl)-1*H*-indol-3-yl)­propyl)­carbamate **29l** (15.0 mg, 1.00 equiv., 39.3 μmol). Purified by column chromatography (8 to 15% MeOH in CH_2_Cl_2_ + 1% NH_4_OH 37% aq. solution) to afford the product as a yellow solid (3.6 mg, 34%). ^1^H NMR (500 MHz, CD_3_OD) δ 8.77 (s, 1H), 8.53 (s, 1H), 8.06 (dt, *J* = 8.0, 1.8 Hz, 1H), 7.57 (dd, *J* = 8.0, 4.8 Hz, 1H), 7.24 (d, *J* = 8.7 Hz, 1H), 6.97 (d, *J* = 2.2 Hz, 1H), 6.76 (dd, *J* = 8.6, 2.3 Hz, 1H), 2.97 (t, *J* = 7.7 Hz, 2H), 2.95 – 2.91 (m, 2H), 2.01 (p, *J* = 7.8 Hz, 2H). ^13^C NMR (126 MHz, CD_3_OD) δ 151.8, 148.9, 148.5, 137.2, 133.2, 132.6, 130.9, 130.6, 125.4, 113.9, 112.9, 112.5, 103.7, 40.7, 29.9, 22.5. υ_max_ (film) 3215, 2924, 2853, 1456, 1206, 802 cm^–1^. HRMS (ESI) *m*/*z* calcd. for [M + H]^+^ (C_16_H_18_N_3_O_1_):268.1444, found: 268.1453.

#### 3-(2-Aminoethyl)-2-(5-fluoropyridin-3-yl)-1H-indol-5-ol (**41**)

Prepared according to General Procedure G from *tert*-butyl (2-(2-(5-fluoropyridin-3-yl)-5-methoxy-1*H*-indol-3-yl)­ethyl)­carbamate **29m** (9.5 mg, 1.0 equiv., 25 μmol). Purified by column chromatography (0 to 12% MeOH in CH_2_Cl_2_ + 1% NH_4_OH 37% aq. solution) to afford the product as a dark green solid (5.3 mg, 55%). ^1^H NMR (700 MHz, CD_3_OD) δ 8.66 (s, 1H), 8.45 (d, *J* = 2.6 Hz, 1H), 7.85 (dt, *J* = 9.8, 2.4 Hz, 1H), 7.25 (d, *J* = 8.6 Hz, 1H), 7.00 (d, *J* = 2.3 Hz, 1H), 6.78 (dd, *J* = 8.7, 2.3 Hz, 1H), 3.13 – 3.09 (m, 2H), 3.03 (t, *J* = 7.7 Hz, 2H).^13^C NMR (176 MHz, CD_3_OD) δ 161.2 (d, ^1^ *J*_*CF*_ = 254.9 Hz), 152.1, 145.4, 136.8 (d, ^2^ *J*_*CF*_ = 23.7 Hz), 133.3, 132.9, 131.9, 130.6, 123.3 (d, ^2^ *J*_*CF*_ = 18.8 Hz), 114.4, 113.1, 111.0, 103.7, 42.2, 27.0. ^19^F NMR (470 MHz, CD_3_OD) δ – 127.95. υ_max_ (film) 3219, 2924, 1558, 1541, 1506, 1456 cm^–1^. HRMS (ESI) *m*/*z* calcd. for [M + Na]^+^ (C_15_H_14_F_1_N_3_O_1_Na_1_): 294.1013, found: 294.1010.

#### 3-(2-Aminoethyl)-2-(pyridin-2-yl)-1H-indol-5-ol (**42**)

Prepared according to General Procedure G from *tert*-butyl (2-(5-methoxy-2-(pyridin-2-yl)-1*H*-indol-3-yl)­ethyl)­carbamate **29n** (20.0 mg, 1.00 equiv., 54.4 μmol). Purified by column chromatography (8 to 12% MeOH in CH_2_Cl_2_ + 1% NH_4_OH 37% aq. solution) to afford the product as a dark yellow solid (8.7 mg, 63%). ^1^H NMR (500 MHz, CD_3_OD) δ 8.62 (ddd, *J* = 5.0, 1.9, 0.9 Hz, 1H), 7.88 (td, *J* = 7.8, 1.9 Hz, 1H), 7.77 (dt, *J* = 8.1, 1.1 Hz, 1H), 7.29 (ddd, *J* = 7.5, 4.9, 1.1 Hz, 1H); H, 7.26 (d, *J* = 8.7 Hz, 1H), 6.99 (d, *J* = 2.3 Hz, 1H), 6.77 (dd, *J* = 8.7, 2.3 Hz, 1H), 3.26 (t, *J* = 7.0 Hz, 2H), 3.09 (t, *J* = 7.0 Hz, 2H).^13^C NMR (126 MHz, CD_3_OD) δ 153.0, 151.9, 150.2, 138.5, 135.2, 133.0, 131.0, 123.0, 122.7, 114.6, 113.1, 111.9, 103.6, 42.5, 27.1. υ_max_ (film) 3242, 2922, 1589, 1456, 1207, 1020, 787 cm^–1^. HRMS (ESI) *m*/*z* calcd. for [M + H]^+^ (C_15_H_16_N_3_O_1_): 254.1288, found: 254.1284.

#### 3-(2-Aminoethyl)-2-(pyridin-4-yl)-1H-indol-5-ol (**43**)

Prepared according to General Procedure G from *tert*-butyl (2-(5-methoxy-2-(pyridin-4-yl)-1*H*-indol-3-yl)­ethyl)­carbamate **29o** (16.9 mg, 1.00 equiv., 46.0 μmol). Purified by column chromatography (5 to 10% MeOH in CH_2_Cl_2_ + 1% NH_4_OH 37% aq. solution) to afford a residue which was further purified by dissolving in the minimum volume of MeOH (approximately 0.2 mL) followed by addition of Et_2_O (2 mL). The precipitate was collected and dried under vacuum to afford the product as a yellow-green solid (5.9 mg, 50%). ^1^H NMR (400 MHz, CD_3_OD) δ 8.59 (d, *J* = 5.0 Hz, 2H), 7.71 – 7.60 (m, 2H), 7.26 (d, *J* = 8.7 Hz, 1H), 7.00 (d, *J* = 2.2 Hz, 1H), 6.79 (dd, *J* = 8.7, 2.1 Hz, 1H), 3.18 (t, *J* = 7.6 Hz, 2H), 3.10 – 3.01 (m, 2H). ^13^C NMR (126 MHz, CD_3_OD) δ 152.1, 150.5, 143.2, 133.4, 133.2, 130.9, 123.3, 115.0, 113.2, 112.0, 103.7, 42.1, 27.0. υ_max_ (film) 3244, 2924, 2855, 1503, 1558, 1456, 1211 cm^–1^. HRMS (ESI) *m*/*z* calcd. for [M + H]^+^ (C_15_H_16_N_3_O_1_): 254.1288, found: 254.1277.

#### 3-(2-Aminoethyl)-2-(pyrimidin-5-yl)-1H-indol-5-ol (**44**)

Prepared according to General Procedure G from *tert*-butyl (2-(5-methoxy-2-(pyrimidin-5-yl)-1*H*-indol-3-yl)­ethyl)­carbamate **29p** (15.4 mg, 1.00 equiv., 41.8 μmol). Purified by column chromatography (5 to 10% MeOH in CH_2_Cl_2_ + 1% NH_4_OH 37% aq. solution) to afford the product as a dark green solid (6.1 mg, 57%). ^1^H NMR (500 MHz, CD_3_OD) δ 9.13 (s, 1H), 9.02 (s, 2H), 7.27 (d, *J* = 8.7 Hz, 1H), 7.01 (d, *J* = 2.3 Hz, 1H), 6.79 (dd, *J* = 8.7, 2.3 Hz, 1H), 3.19 – 3.07 (m, 4H).^13^C NMR (126 MHz, CD_3_OD) δ 157.5, 156.6, 152.2, 133.6, 130.4, 130.0, 129.4, 114.7, 113.2, 110.7, 103.7, 41.9, 25.8. υ_max_ (film) 3244, 2922, 2853, 1624, 1458, 1412, 1015 cm^–1^. HRMS (ESI): *m*/*z* calcd. for [M + H]^+^ (C_14_H_15_N_4_O_1_): 255.1240, found: 255.1236.

#### 2-(2-Aminoethyl)-3-(pyrimidin-5-yl)-1H-indol-5-ol (**53**)

Prepared according to General Procedure G from *tert*-butyl (2-(5-methoxy-3-(pyrimidin-5-yl)-1*H*-indol-2-yl)­ethyl)­carbamate **29q** (9.2 mg, 1.00 equiv., 25 μmol). Purified by column chromatography (5 to 10% MeOH in CH_2_Cl_2_ + 1% NH_4_OH 37% aq. solution) to afford the product as a dark green solid (5.8 mg, 91%). ^1^H NMR (500 MHz, CD_3_OD) δ 9.06 (s, 1H), 8.90 (s, 2H), 7.23 (d, *J* = 8.7 Hz, 1H), 6.91 (d, *J* = 2.3 Hz, 1H), 6.73 (dd, *J* = 8.7, 2.3 Hz, 1H), 3.04 (s, 4H). ^13^C NMR (126 MHz, CD_3_OD) δ 157.8, 156.1, 152.8, 136.6, 132.3, 132.3, 128.9, 113.1, 112.8, 107.4, 102.6, 42.0, 29.7. υ_max_ (film) 3217, 2924, 1558, 1412, 1163, 802 cm^–1^. HRMS (ESI) *m*/*z* calcd. for [M + H]^+^ (C_14_H_15_N_4_O_1_): 255.1240, found: 255.1246.

#### 3-(2-Aminoethyl)-2-(thiazol-5-yl)-1H-indol-5-ol (**45**)

Prepared according to General Procedure G from *tert*-butyl (2-(5-methoxy-2-(thiazol-5-yl)-1*H*-indol-3-yl)­ethyl)­carbamate **29r** (8.6 mg, 1.0 equiv., 23 μmol). Purified by column chromatography (8 to 12% MeOH in CH_2_Cl_2_ + 1% NH_4_OH 37% aq. solution) to afford the product as a yellow solid (4.1 mg, 69%). ^1^H NMR (500 MHz, CD_3_OD) δ 9.03 (s, 1H;), 8.13 (s, 1H), 7.21 (d, *J* = 8.7 Hz, 1H), 6.96 (d, *J* = 2.3 Hz, 1H), 6.76 (dd, *J* = 8.7, 2.3 Hz, 1H), 3.15 – 3.10 (m, 2H), 3.03 – 2.99 (m, 2H). ^13^C NMR (126 MHz, CD_3_OD) δ 154.4, 152.1, 140.6, 133.2, 131.7, 130.6, 127.2, 114.4, 112.9, 111.2, 103.5, 42.1, 27.2. υ_max_ (film) 3210, 2920, 1456, 1217, 837, 802 cm^–1^. HRMS (ESI): [M + H]^+^ (C_13_H_14_N_3_O_1_S_1_): 260.0852, found: 260.0854.

#### 3-(2-Aminoethyl)-1-methyl-2-(pyridin-3-yl)-1H-indol-5-ol (**48**)

Prepared according to General Procedure G from 2-(5-(benzyloxy)-1-methyl-2-(pyridin-3-yl)-1*H*-indol-3-yl)­ethan-1-amine **S19** (6.5 mg, 1.0 equiv., 18 μmol). Purified by column chromatography (5 to 10% MeOH in CH_2_Cl_2_ + 1% NH_4_OH 37% aq. solution) to afford the product as a brown solid (2.4 mg, 50%). ^1^H NMR (500 MHz, CD_3_OD) δ 8.67 – 8.63 (m, 1H), 8.62 (d, *J* = 1.8 Hz, 1H), 7.94 (dt, *J* = 7.9, 1.9 Hz, 1H), 7.62 (ddd, *J* = 7.8, 4.9, 0.9 Hz, 1H), 7.28 (d, *J* = 8.8 Hz, 1H), 7.01 (d, *J* = 2.3 Hz, 1H), 6.89 – 6.78 (m, 1H), 3.56 (s, 3H), 2.98 – 2.94 (m, 2H), 2.93 – 2.89 (m, 2H). ^13^C NMR (126 MHz, CD_3_OD) δ 152.3, 151.4, 149.8, 140.3, 136.4, 134.3, 130.0, 129.2, 125.3, 113.7, 111.5, 110.2, 103.8, 42.1, 31.2, 26.2. υ_max_ (solid) 3354, 2922, 2490, 1456, 1020, 797 cm^–1^. HRMS (ESI) *m*/*z* calcd. for [M + H]^+^ (C_16_H_18_N_3_O_1_): 268.1444, found: 268.1449.

#### 2-(5-(2-Aminoethyl)­pyridin-3-yl)-1H-indol-5-ol (**49**)

Prepared according to General Procedure G from *tert*-butyl (2-(5-(5-methoxy-1*H*-indol-2-yl)­pyridin-3-yl)­ethyl)­carbamate **S25** (12.2 mg, 1.00 equiv., 33.2 μmol). Purified by column chromatography (8 to 12% MeOH in CH_2_Cl_2_ + 1% NH_4_OH 37% aq. solution) to afford the product as a brown solid (7.7 mg, 92%). ^1^H NMR (500 MHz, CD_3_OD) δ 8.82 (d, *J* = 2.2 Hz, 1H), 8.31 (d, *J* = 2.0 Hz, 1H), 8.08 (t, *J* = 2.1 Hz, 1H), 7.24 (d, *J* = 8.7 Hz, 1H), 6.94 (dd, *J* = 2.4, 0.6 Hz, 1H), 6.81 (d, *J* = 0.9 Hz, 1H), 6.72 (dd, *J* = 8.7, 2.4 Hz, 1H), 3.06 (t, *J* = 7.4 Hz, 2H), 2.91 (t, *J* = 7.4 Hz, 2H). ^13^C NMR (126 MHz, CD_3_OD) δ 152.1, 148.4, 144.8, 136.7, 135.8, 134.3, 134.2, 131.1, 131.0, 113.9, 112.8, 105.4, 100.9, 43.1, 35.6. υ_max_ (solid) 2918, 2851, 1458, 1192, 785 cm^–1^. HRMS (ESI) *m*/*z* calcd. for [M + H]^+^ (C_15_H_16_N_3_O_1_): 254.1288, found: 254.1293.

### General Procedure H for the Preparation of Compounds 1, 34–36, 50

A round-bottom flask under air was charged with *N*-Boc-amide (1.00 equiv) and CH_2_Cl_2_ (1.20 mL per 0.10 mmol amide). The mixture was cooled to 0 °C, and TFA (300 μL per 0.10 mmol amide) was added. The mixture was stirred at 0 °C for 40 min, after which it was allowed to warm to RT and was stirred for a further 80 min. Volatiles were removed under reduced pressure to afford the product. Where purification was necessary, this was achieved by column chromatography (silica gel).

#### 2-(2-(Pyridin-3-yl)-1H-indol-3-yl)­ethan-1-amine, TFA salt (**1**)

Prepared according to General Procedure H from *tert*-butyl (2-(2-(pyridin-3-yl)-1*H*-indol-3-yl)­ethyl)­carbamate **29u** (11.5 mg, 1.00 equiv., 34.1 μmol) to afford the product as a yellow solid (16.3 mg, quant.). ^1^H NMR (500 MHz, CD_3_OD) δ 8.99 (s, 1H), 8.73 (s, 1H), 8.45 (d, *J* = 8.3 Hz), 7.93 – 7.86 (m, 1H), 7.69 (dt, *J* = 8.1, 1.0 Hz, 1H), 7.46 (d, *J* = 8.2 Hz, 1H), 7.25 (ddd, *J* = 8.2, 7.0, 1.1 Hz, 1H), 7.15 (ddd, *J* = 8.0, 7.1, 1.0 Hz, 1H), 3.34 – 3.31 (m, 2H), 3.27 – 3.21 (m, 2H). ^13^C NMR (126 MHz, CD_3_OD) δ 145.5, 145.1, 141.6, 138.6, 132.7, 131.3, 129.4, 127.2, 124.6, 121.2, 119.7, 112.8, 110.3, 40.9, 23.8. υ_max_ (film) 2922, 1682, 1566, 1188, 1134, 835, 802 cm^–1^. HRMS (ESI) *m*/*z* calcd. for [M + H]^+^ (C_15_H_16_N_3_): 238.1339, found: 238.1342.

#### 2-(5-Fluoro-2-(pyridin-3-yl)-1H-indol-3-yl)­ethan-1-amine (**34**)

Prepared according to General Procedure H from *tert*-butyl (2-(5-fluoro-2-(pyridin-3-yl)-1*H*-indol-3-yl)­ethyl)­carbamate **29e** (10.6 mg, 1.00 equiv., 29.8 μmol). Purified by column chromatography (4 to 8% MeOH in CH_2_Cl_2_ + 1% NH_4_OH 37% aq. solution) to afford the product as a light brown solid (5.2 mg, 68%). ^1^H NMR (500 MHz, (CD_3_)_2_O) δ 9.03 (d, *J* = 0.9 Hz, 1H), 8.57 (dd, *J* = 4.8, 1.7 Hz, 1H), 8.27 (ddd, *J* = 7.9, 2.3, 1.6 Hz, 1H), 7.48 (ddd, *J* = 7.9, 4.7, 0.9 Hz, 1H), 7.43 – 7.37 (m, 2H), 6.95 (ddd, *J* = 9.5, 8.8, 2.5 Hz, 1H), 3.60 (t, *J* = 7.0 Hz, 2H), 3.15 (t, *J* = 7.0 Hz, 2H).^13^C NMR (126 MHz, (CD_3_)_2_O) δ 158.5 (d, ^1^ *J*_*CF*_ = 232.1 Hz), 149.9, 149.4, 136.2, 134.7, 134.1, 130.3 (d, ^3^ *J* = 9.7 Hz), 129.9, 124.3, 113.3 (d, ^4^ *J*_*CF*_ = 5.0 Hz), 112.9 (d, ^3^ *J*_*CF*_ = 9.7 Hz), 111.02 (d, ^2^ *J*_*CF*_ = 26.5 Hz), 104.7 (d,^2^ *J*_*CF*_ = 23.5 Hz), 52.5, 26.7. ^19^F NMR (377 MHz, (CD_3_)_2_O) δ – 126.16. υ_max_ (film) 3167, 2922, 1447, 1177, 797 cm^–1^. HRMS (ESI) *m*/*z* calcd. for [M + H]^+^ (C_15_H_15_F_1_N_3_): 256.1245, found: 256.1252.

#### 2-(6-Fluoro-2-(pyridin-3-yl)-1H-indol-3-yl)­ethan-1-amine, TFA Salt (**35**)

Prepared according to General Procedure H from *tert*-butyl (2-(6-fluoro-2-(pyridin-3-yl)-1*H*-indol-3-yl)­ethyl)­carbamate **29f** (8.2 mg, 1.00 equiv., 23 μmol) to afford the product as a yellow solid (11.3 mg, quant.). ^1^H NMR (500 MHz, CD_3_OD) δ 8.93 (s, 1H), 8.70 (s, 1H), 8.34 (dt, *J* = 8.1, 1.6 Hz, 1H, 7.86 – 7.79 (m, 1H), 7.65 (dd, *J* = 8.8, 5.1 Hz, 1H), 7.15 (dd, *J* = 9.7, 2.3 Hz, 1H), 6.94 (ddd, *J* = 9.6, 8.7, 2.3 Hz, 1H), 3.29 – 3.26 (m, 2H), 3.24 – 3.19 (m, 2H). ^13^C NMR (126 MHz, CD_3_OD) δ 162.1 (d, ^1^ *J*_*CF*_ = 238.4 Hz), 146.5, 146.3, 140.5, 138.6 (d, ^3^ *J*_*CF*_ = 12.7 Hz), 132.5, 132.0, 126.8, 126.2, 120.8 (d, ^3^ *J*_*CF*_ = 10.2 Hz), 110.2, 109.8 (d, ^2^ *J*_*CF*_ = 25.1 Hz), 98.6 (d, ^2^ *J*_*CF*_ = 26.3 Hz), 40.9, 23.8. ^19^F NMR (377 MHz, CD_3_OD) δ – 77.15 (TFA), – 121.28. υ_max_ (solid) 2924, 1668, 1188, 1140, 1126, 839, 804 cm^–1^. HRMS (ESI) *m*/*z* calcd. for [M + H]^+^ (C_15_H_15_F_1_N_3_): 256.1245, found: 256.1250.

#### 3-(2-Aminoethyl)-2-(pyridin-3-yl)-1H-indol-5-amine TFA Salt (**36**)

Prepared according to General Procedure H from *tert*-butyl (2-(5-((*tert*-butoxycarbonyl)­amino)-2-(pyridin-3-yl)-1*H*-indol-3-yl)­ethyl)­carbamate **29g** (10.0 mg, 1.00 equiv., 22.1 μmol) to afford the product as a brown solid (11.0 mg, quant.). ^1^H NMR (700 MHz, CD_3_OD) δ 9.05 – 8.76 (m, 2H), 8.42 (d, *J* = 7.9 Hz, 1H), 7.91 (s, 1H), 7.78 (d, *J* = 2.1 Hz, 1H), 7.60 (d, *J* = 8.6 Hz, 1H), 7.24 (dd, *J* = 8.6, 2.1 Hz, 1H), 3.33 – 3.31 (m, 2H), 3.25 – 3.21 (m, 2H).^13^C NMR (176 MHz, CD_3_OD) δ 146.6, 146.6, 143.2, 141.0, 137.8, 134.6, 131.7, 129.7, 124.4, 118.6, 114.3, 114.2, 110.5, 40.8, 23.6. υ_max_ (film) 2926, 1667, 1431, 1180, 1125, 837, 799, 721 cm^–1^. HRMS (ESI) *m*/*z* calcd. for [M + Na]^+^ (C_15_H_16_N_4_Na_1_): 275.1267, found: 275.1271.

#### 2-(3-(Pyridin-3-yl)-1H-indol-2-yl)­ethan-1-amine, TFA Salt (**50**)

Prepared according to General Procedure H from *tert*-butyl (2-(3-(pyridin-3-yl)-1*H*-indol-2-yl)­ethyl)­carbamate **29v** (11.3 mg, 1.00 equiv., 33.5 μmol) to afford the product as an orange solid (15.8 mg, quant.). ^1^H NMR (500 MHz, CD_3_OD) δ 8.92 (s, 1H), 8.73 (s, 1H), 8.56 (d, *J* = 7.9 Hz, 1H), 8.03 (bs, 1H), 7.59 (dt, *J* = 8.0, 1.0 Hz, 1H), 7.46 (dt, *J* = 8.2, 0.9 Hz, 1H), 7.23 (ddd, *J* = 8.2, 7.1, 1.1 Hz, 1H), 7.14 (ddd, *J* = 8.1, 7.1, 1.0 Hz, 1H), 3.36 – 3.32 (m, 2H), 3.30 – 3.27 (m, 2H). ^13^C NMR (126 MHz, CD_3_OD) δ 145.5, 144.2, 141.6, 137.8, 136.3, 133.8, 128.0, 127.8, 124.0, 122.0, 118.6, 112.6, 110.3, 40.0, 25.4. υ_max_ (film) 2924, 1668, 1568, 1177, 1126, 835, 799 cm^–1^. HRMS (ESI) *m*/*z* calcd. for [M + H]^+^ (C_15_H_16_N_3_): 238.1339, found: 238.1339.

#### 3-(2-Aminoethyl)-2-(pyridin-3-yl)-1H-indole-5-carboxylic Acid Hydrochloride (**37**)

An oven-dried microwave vial was charged with methyl 3-(2-((*tert*-butoxycarbonyl)­amino)­ethyl)-2-(pyridin-3-yl)-1*H*-indole-5-carboxylate **29h** (8.4 mg, 1.0 equiv., 21 μmol), AcOH (0.40 mL) and conc. HCl (0.10 mL). The vial was sealed, and the mixture was stirred at 100 °C for 18 h. After cooling to RT, volatiles were removed under reduced pressure to afford a solid which was dissolved in the minimum volume of MeOH (approximately 0.2 mL) followed by addition of Et_2_O (2 mL). The precipitate was collected and dried under vacuum to afford the product as a brown solid (6.0 mg, 80%). ^1^H NMR (400 MHz, CD_3_OD) δ 9.18 – 9.02 (m, 1H), 8.93 – 8.78 (m, 1H), 8.65 (d, *J* = 8.1 Hz, 1H), 8.49 (dd, *J* = 1.6, 0.7 Hz, 1H), 8.12 – 8.07 (m, 1H), 7.97 (dd, *J* = 8.6, 1.6 Hz, 1H), 7.54 (dd, *J* = 8.6, 0.7 Hz, 1H), 3.40 – 3.34 (m, 2H), 3.29 – 3.23 (m, 2H). ^13^C NMR (126 MHz, CD_3_OD) δ 170.9, 145.6, 142.0, 141.8, 141.1, 131.4, 130.6, 129.3, 129.0, 125.9, 123.6, 123.0, 112.6, 111.7, 41.0, 23.6. υ_max_ (solid) 2920, 2853, 1665, 1560, 1221, 814 cm^–1^. HRMS (ESI) *m*/*z* calcd. for [M + Na]^+^ (C_16_H_15_N_3_O_2_Na_1_): 304.1057, found: 304.1055.

#### 3-(2-(Methylamino)­ethyl)-2-(pyridin-3-yl)-1H-indol-5-ol Hydrochloride (**46**)

A round-bottom flask fitted with a reflux condenser was charged with *tert*-butyl (2-(5-(methoxymethoxy)-2-(pyridin-3-yl)-1*H*-indol-3-yl)­ethyl)­(methyl)­carbamate **29s** (12.4 mg, 1.00 equiv., 30.1 μmol), MeOH (1.20 mL), and 3 M HCl (0.400 mL). The mixture was heated to reflux for 2 h, after which volatiles were removed under reduced pressure to afford the product as a yellow solid (10.3 mg, quant.). ^1^H NMR (500 MHz, CD_3_OD) δ 9.13 (s, 1H), 8.87 – 8.73 (m, 2H), 8.22 (s, 1H), 7.32 (d, *J* = 8.7 Hz, 1H), 7.07 (d, *J* = 2.2 Hz, 1H), 6.85 (d, *J* = 8.7 Hz, 1H), 3.35 – 3.32 (m, 2H), 3.30 – 3.27 (m, 2H), 2.72 (s, 3H). ^13^C NMR (126 MHz, CD_3_OD) δ 152.7, 145.9, 141.3, 140.7, 133.8, 130.1, 130.1, 129.8, 129.0, 115.9, 113.6, 110.7, 103.7, 50.2, 33.8, 22.7. υ_max_ (film) 3201, 2920, 2851, 1560, 1464, 797 cm^–1^. HRMS (ESI) *m*/*z* calcd. for [M + H]^+^ (C_16_H_18_N_3_O_1_): 268.1444, found: 268.1436.

#### 3-(2-Hydroxyethyl)-2-(pyridin-3-yl)-1H-indol-5-ol (**47**)

A round-bottom flask fitted with a reflux condenser was charged with 3-(2-((*tert*-butyldimethylsilyl)­oxy)­ethyl)-5-(methoxymethoxy)-2-(pyridin-3-yl)-1*H*-indole **29t** (11.1 mg, 1.00 equiv., 26.9 μmol), MeOH (1.20 mL), and 3 M HCl (400 μL). The mixture was heated to 80 °C for 2 h, after which volatiles were removed under reduced pressure. The residue was purified by column chromatography (silica gel, 0 to 6% MeOH in CH_2_Cl_2_) to afford the product as a yellow solid (2.5 mg, 32%). ^1^H NMR (500 MHz, CD_3_OD) δ 8.85 (s, 1H), 8.50 (d, *J* = 4.9 Hz, 1H), 8.14 (dt, *J* = 8.0, 2.0 Hz, 1H), 7.54 (dd, *J* = 8.0, 4.9 Hz, 1H), 7.22 (d, *J* = 8.6 Hz, 1H), 6.97 (d, *J* = 2.2 Hz, 1H), 6.73 (dd, *J* = 8.5, 1.7 Hz, 1H), 3.84 (t, *J* = 7.3 Hz, 2H), 3.06 (t, *J* = 7.3 Hz, 2H). ^13^C NMR (126 MHz, CD_3_OD) δ 151.7, 149.0, 148.3, 137.3, 133.3, 133.3, 131.8, 131.0, 125.3, 113.7, 112.8, 110.8, 103.9, 63.2, 29.3. υ_max_ (solid) 3215, 2920, 1454, 1204, 1042 cm^–1^. HRMS (ESI) *m*/*z* calcd. for [M + H]^+^ (C_15_H_15_N_2_O_2_): 255.1128, found: 255.1120.

#### 2-(2-Aminoethyl)-3-(pyridin-3-yl)-1H-indol-5-ol (**51**)

A round-bottom flask was charged with Pd/C (8.6 mg, 20 mol %, 10 wt % on carbon). The flask was sealed, evacuated, and refilled with N_2_ three times. *N*,*N*-Dibenzyl-2-(5-(benzyloxy)-3-(pyridin-3-yl)-1*H*-indol-2-yl)­ethan-1-amine **S28 (**21.3 mg, 1.00 equiv., 40.7 μmol) in MeOH (1.0 mL) was added, followed by AcOH (47 μL, 20 equiv., 0.81 mmol). H_2_ gas was bubbled through the mixture for 5 min, after which the reaction was heated to 60 °C under H_2_ atmosphere (1 atm) for 24 h. After cooling to RT, the mixture was filtered through Celite, volatiles were removed under reduced pressure, and the residue was purified by column chromatography (silica gel, 10 to 12% MeOH in CH_2_Cl_2_ + 1% NH_4_OH 37% aq. solution) to obtain the product as a green-brown solid (6.8 mg, 66%). ^1^H NMR (400 MHz, CD_3_OD) δ 8.63 (s, 1H), 8.46 (d, *J* = 3.8 Hz, 1H), 7.97 – 7.90 (m, 1H), 7.54 (dd, *J* = 8.0, 5.0 Hz, 1H), 7.21 (d, *J* = 8.6 Hz, 1H), 6.89 (d, *J* = 2.4 Hz, 1H), 6.71 (dd, *J* = 8.6, 2.3 Hz, 1H), 3.08 (m, 4H). ^13^C NMR (126 MHz, CD_3_OD) δ 152.5, 150.3, 147.0, 138.9, 135.0, 134.0, 132.3, 129.3, 125.4, 112.9, 112.6, 111.2, 103.1, 41.6, 28.8. υ_max_ (film) 3210, 2968, 2922, 1458, 1406, 1051 cm^–1^. HRMS (ESI) *m*/*z* calcd. for [M + H]^+^ (C_15_H_16_N_3_O_1_): 254.1288, found: 254.1285.

### Biochemical Fluorescence Assay

Enzymes were purified as below and the biochemical assay including materials used was based on the publication by Visnes et al.^51^ and “EUbOPEN” protocols (https://www.eubopen.org/protocols-reagents) and adapted where mentioned. OGG1 activation was monitored in a kinetic mode and a time-resolved curve was obtained for each compound concentration in triplicates except where stated otherwise. Initial slopes were taken of the linear part of these curves to determine rates and kinetics. Fluorescence values of each compound concentration were normalized by the DMSO control values and calculated as % activation of the full turnover fluorescence of the APE1 (2 nM) control. The median activation concentration (AC_50_) for each compound was calculated from the % activation of all tested concentrations and refers to the compound concentration which activates the reaction to 50% of the substrate turnover reached in the assay control coupled to APE1 (2 nM) as described before.^67^ In the screening, primary hits were defined as compounds with an AC_50_ of below 100 μM.

### Proteins and Reagents

Enzymes (APE1, UDG, TDG, SMUG1, MPG, NEIL1, NTHL1, NUDT15, NUDT22, NUDT5) and mutants were produced as reported previously.^51,67,96,97^ 8-oxoG-containing oligonucleotide (see sequence below) was radiolabeled at the 5′ end with [γ^32^P]-ATP (PerkinElmer Life Sciences) and T4 polynucleotide kinase (T4PNK) from New England Biolabs.

### Oligonucleotides

#### For PAGE Analysis

Oligonucleotide 5́GTACCCGGGGATCCGTAA8GCGCATCAGCTGCAG (Integrated DNA Technologies), where 8 stands for 8oxodG, was 5′-labeled and further hybridized to the complementary oligonucleotide 5′-CAGCAGCTGATGCGCCTTACGGATCCCCGGGTAC in the presence of 60 mM Tris-HCl (pH 7.5) and 0.2 M NaCl by heating to 80 °C for 5 min before slowly cooling to room temperature overnight. For standard biochemical assay: Complementary strands containing FAM and DAB were ordered from ATD BIO. Sequences were 5′-FAM-TCTGCCA8CACTGCGTCGACCTG-3′ and 5′-CAGGTCGACGCAGTGYTGGCAGT-Dab-3′ where 8 is a uracil or 8-oxoA, and Y is the corresponding required complementary base.

### hOGG1 Activity Assay on Radiolabeled 8-OxoG-Containing Oligonucleotide

To analyze the activity of hOGG1 on 8-oxoG containing substrates, 2 nM of the indicated 34mer [^32^P]­5′-labeled 8oxoG-containing substrate was incubated with 10 nM wildtype hOGG1 and either 10% DMSO, 6.25 μM TH10785, 6.25 μM **30**, 10 μM **38**, or 50 μM TH5487 in the presence of 30 mM Hepes, pH 7.5, 4% glycerol, 20 mM EDTA, 16 mM NaCl, and 0.01% Tween-20 in a final volume of 12.5 μL. Samples were incubated at 37 °C for the indicated times. The reactions were stopped by addition of 12.5 μL of formamide buffer (20 mM EDTA, 95% formamide, 0.05% bromophenol blue and 0.05% xylencyanol blue) and further heating at 90 °C for 2 min. DNA products were analyzed by 7 M urea-20% PAGE and visualized with an Amersham Typhoon scanner.

### NaBH_4_ Trapping Assay

The reactions were carried out in a final volume of 12.5 μL in the presence of either 10% DMSO or 6.25 μM compound **30** or 10 μM compound **38**, in the presence of 30 mM HEPES, pH 7.5, 4% glycerol, 20 mM EDTA, 16 mM NaCl and 0.01% Tween-20. In the trapping assay on an AP-containing DNA, 1 nM of the 34mer [^32^P]­5′-labeled uridine-containing substrate was treated with 0.2 U *E. coli* UDG (NEB) for 15 min at 37 °C to leave an intact AP site. 78 pM hOGG1 was added and after 30 s, the Schiff base intermediate was trapped by adding 100 mM NaBH_4_ or 100 mM NaCl, as indicated. After incubation for 20 min on ice, samples were analyzed by 12% SDS-PAGE and the reaction products visualized with an Amersham Typhoon scanner. In the trapping assay on an 8oxoG-containing DNA, 2 nM of the indicated 34 mer [^32^P]­5′-labeled 8oxoG-containing substrate was used. 625 pM *h*OGG1 was added and after 1 min, the Schiff base intermediate was trapped by adding 100 mM NaBH_4_ or 100 mM NaCl, as indicated, and products were processed as indicated above.

### Kinetic Solubility Assay

Performed according to Enamine’s aqueous solubility standard operating procedure. Briefly, using a 17.5 mM stock solution of **30** in 100% DMSO dilutions were prepared to a theoretical concentration of 350 μM in duplicates in phosphate-buffered saline pH 7.4 (138 mM NaCl, 2.7 mM KCl, 10 mM K-phosphate) with 2% final DMSO. The experimental compound dilutions in PBS were further allowed to equilibrate at 25 °C on a Thermomixer R Block, 1.5 mL (Eppendorf, Germany; Cat # 5355) for 2 h and then filtered through HTS filter plates using a vacuum manifold. The filtrates of test compounds were diluted 2-fold with acetonitrile with 2% DMSO before measuring.

In parallel, using a 17.5 mM stock solution of **30** in 100% DMSO dilutions were prepared to theoretical concentrations of 0 μM (blank), 8.75 μM, 21.875 μM, 43.75 μM, 87.5 μM, and 175 μM in 50% acetonitrile/PBS with 2% final DMSO to generate calibration curves. Ondansetron was used as a reference compound to control proper assay performance. 200 μL of each sample was transferred to a 96-well plate and measured in the 230–550 nm range with a 5 nm step on a SpectraMax Paradigm Reader (Multi-Mode Detection Platform, Product # 33270–1279) using SoftMax Pro v.5.4 (Molecular Devices) software.

The concentrations of compounds in PBS filtrate were calculated using a dedicated Microsoft Excel calculation script. Proper absorbance wavelengths for calculations are selected for each compound manually based on absorbance maximums (absolute absorbance unit values for the minimum and maximum concentration points within the 0–3 OD range). Each final data set is visually evaluated by the operator, and goodness-of-fit (R^2^) is calculated for each calibration curve. The effective range of this assay is approximately 2–350 μM and the compounds returning values close to the upper limit of the range may have higher actual solubility (e.g., 5′-deoxy-5-fluorouridine).

### Microsomal Stability Assay

Microsomal incubations were carried out in 96-well plates in 5 aliquots of 30 μL each (one for each time point). Liver microsomal incubation medium comprised of phosphate buffer (100 mM, pH 7.4), MgCl_2_ (3.3 mM), NADPH (3 mM), glucose-6-phosphate (5.3 mM), glucose-6-phosphate dehydrogenase (0.67 units/mL) with 0.42 mg of liver microsomal protein per mL. In the control reactions, the NADPH-cofactor system was substituted with phosphate buffer. Test compounds (2 μM, final acetonitrile concentration 1.6%) were incubated with microsomes at 37 °C, shaking at 100 rpm in a Shaker Innova 4330 (New Brunswick Scientific). Five time points over 40 min were analyzed. The reactions were stopped by adding 5 volumes of acetonitrile with internal standard to incubation aliquots, followed by protein sedimentation by centrifuging at 5500 rpm for 5 min. Each reaction was performed in duplicates. Supernatants were analyzed using a gradient HPLC system (Agilent Technologies) coupled to a triple quadrupole mass-detector API 3000 with TurboIonSpray Ion Source (AB Sciex, Canada). The elimination rate constant (k_el_), half-life (*t*_1/2_), and intrinsic clearance (Cl_int_) were determined in a plot of ln­(AUC) versus time, using linear regression analysis.

### Caco-2 Permeability Assay

Caco-2 cells were cultured in 75 mL flasks to 80–90% confluence according to the ATCC and Millipore recommendations^98^ in a humidified atmosphere at 37 °C and 5% CO_2_. Cells were detached with Trypsin/EDTA solution and resuspended in the complete medium containing DMEM high glucose (4500 mg/L) with l-glutamine (4 mM) supplemented with 10% heat-inactivated Fetal Bovine Serum, 1% nonessential amino acids, and 730 nM puromycin and seeded at a density 5 × 105 cells in 75 mL flask.^99^ After 5 days, cells were trypsinized and resuspended in the complete medium to a final concentration of 600 × 103 cells/mL; 400 μL of the cell suspension was added to each well of the HTS 24-Multiwell Insert System, and 25 mL of prewarmed complete medium was added to the feeder tray. Caco-2 cells were incubated in Multiwell Insert System for 6–10 days before the transport experiments. The medium in the filter plate and feeder tray was refreshed every other day. Prior to the transport experiment, the integrity of the monolayer was verified by measuring the transepithelial electrical resistance (TEER) for every well using the Millicell-ERS system ohm meter. The final TEER values were within the range of 150–600 Ω × cm^2^ as required for the assay conditions.^100^ The 24-well insert plate was removed from its feeder plate and placed in a new sterile 24-well transport analysis plate. The inserts were washed with PBS after medium aspiration. Ketoprofen, Atenolol, and Digoxin were used as reference compounds.

To determine the rate of compound transport in apical (A)-to-basolateral (B) direction, 300 μL of the test compound dissolved in transport buffer (Hanks’ BSS (9.5 g/L) and NaHCO_3_ (0.35 g/L) with MgSO_4_ to final concentration 0.81 mM, CaCl_2_ to final concentration 1.26 mM, HEPES to final concentration 25 mM. pH adjusted to 7.4) was added into the filter wells; 1000 μL of transport buffer was added to transport analysis plate wells.

To determine transport rates in the basolateral (B)-to-apical (A) direction, 1000 μL of the test compound solutions was added into the wells of the transport analysis plate, the wells in the filter plate were filled with 300 μL of buffer (apical compartment).

The effect of the inhibitor on the P-gp-mediated transport of the tested compounds was assessed by determining the bidirectional transport in the presence or absence of verapamil. The Caco-2 cells were preincubated for 30 min at 37 °C with 100 μM of verapamil in both apical and basolateral compartments. After removal of the preincubation medium, the test compounds with verapamil (100 μM) in transport buffer were added to donor wells, while the receiver wells were filled with the appropriate volume of transport buffer with 100 μM of verapamil. The final amount of test and reference compounds was 10 μM.

The plates were incubated for 90 min at 37 °C under continuous shaking at 100 rpm. 75 μL aliquots were taken from the donor and receiver compartments for LC-MS/MS analysis. All samples were mixed with 2 volumes of acetonitrile followed by protein sedimentation by centrifuging at 10000 rpm for 10 min. Supernatants were analyzed using the HPLC system coupled with a tandem mass spectrometer.

All solutions of test and reference compounds were prepared manually, and further manipulations with the solutions were performed with automation using Opentrons. The apparent permeability (*P*_app_) was calculated for the Caco-2 permeability assay using the following equation:

$$P_{\mathrm{app}}=\frac{V_{A}}{\mathrm{Area}\times \mathrm{Time}}\times \frac{{[\mathrm{drug}]}_{\mathrm{acc}}}{{[\mathrm{drug}]}_{\mathrm{initial},d}}$$

Where *V*_*A*_ is the volume of transport buffer in acceptor well, *Area* is the surface area of the inset (equals to the effective growth area of the inset – 0.7 cm^2^), *Time* is the time of the assay, [*drug*]_acc_ is the amount of test compound in acceptor well, [*drug*]_initial, d_ is the initial amount of test compound in a donor well.

Efflux ratio (*P*_app_(BA)/*P*_app_(AB)) reveals the difference in *P*_app_ as a result of active transport. If the efflux ratio is greater than 2, this indicates the occurred active efflux. To identify P-gp substrates, the P-gp inhibitor verapamil was added to the incubation medium. A decrease of the efflux ratio in the presence of verapamil indicates that the compound is a P-gp substrate.

### Virtual Screening of the NCI DTP Collection

#### Ligand Preparation

the PubChem version of the NCI Developmental Therapeutics Program (DTP) database,^101^ amounting to ∼ 277 K structures in January 2017, was imported into InstantJChem version 18.22.5 for structure database management.^102^ Compounds overlapping with our in-house collection and those previously indicated to be unavailable from NCI were annotated as such. A workflow was built in KNIME version 4.0.1 ^103^ to clean the database from unwanted structures before initiating virtual screening. Briefly, SMILES strings were desalted using the Speedy SMILES Desalt node, keeping only the first unique component and the longest SMILES string, canonicalized using the RDKit Canon SMILES node, and then grouped on identical structures to remove duplicates. RDKit Functional Group Filter nodes were applied to remove structures matching REOS^104^ and PAINS.^105^ The RDKit Descriptor Calculation node was used to calculate molecular weight and logP, and numeric outliers for both descriptors were removed. After applying these filters ∼ 99K structures remained and were prepared for docking using LigPrep (Schrödinger Suite 2019–3).^106^ Epik was used to assign possible protonation states at pH 7.4 ± 2.0, possible tautomers were enumerated and a maximum of 4 stereoisomers was allowed for unassigned stereocenters. Using these settings, LigPrep generated ∼ 200 K structural species for docking.

#### Protein Preparation

The crystal structure of human OGG1 in complex with the activator TH10785 (7AYY.pdb)^67^ was used for docking and prepared using the Protein Preparation workflow (Schrödinger Suite 2019–3).^106^ Chain A was chosen for preparation, and two versions of the protein were prepared: one with Asp268 in neutral form, donating a H-bond to the quinazoline N1 nitrogen of TH10785, and one with Asp268 deprotonated, accepting an H-bond from the protonated quinazoline N1 of TH10785. Ligands other than TH10785 were deleted, as well as any crystal waters. Structural defects such as bond orders were automatically fixed and protonation states at pH 7.4 assigned using Epik. Hydroxyl group, Asn, Gln and His states were automatically optimized using ProtAssign. Finally, an OPLS4-based all-atom restrained minimization to 0.30 Å RMSD was performed to remove any clashes and/or strain. Glide docking grids were centered on TH10785, setting the enclosing box size to be comparable to the size of the bound ligand.

#### Virtual Screening

The Virtual Screening Workflow as implemented in Schrödinger Suite 2019–3 was used, providing a 3-stage screening funnel employing the Glide docking module with 3 different levels of speed vs. accuracy, namely Glide HTVS, SP, and XP, where the top 10% scoring poses were passed on to the next stage. The prepared ligands were docked to both prepared protein structures without any constraints. Epik state penalties were added to the docking scores and enhanced planarity of conjugated pi groups was enforced. After Glide XP docking 803 unique ligands remained which were further manually triaged to 669 structures. Further triaging was done based on ligand efficiency (LE), calculated as – (docking_score)/# heavy atoms, and applying a threshold of 0.40, which left 406 compounds. From these, 40 structures were selected and acquired from NCI, in part based on structural appeal and in part by their availability. These 40 structures covered a Glide XP Gscore range of – 11.14 to – 9.12 kcal/mol (LE: 0.40–0.78). Compound **1** had a Gscore of – 9.91 kcal/mol and LE of 0.55, and TH12166 had a Gscore of – 9.86 kcal/mol and LE of 0.52.

### Docking Analysis of Tryptamine Library

#### Ligand Preparation

Structures were exported as sdf from ChemDraw, InstantJChem or other sources and imported into the Maestro Suite. Using the OPLS3e force field, possible protonation states at pH 7.0 ± 2.0 were generated using Epik. Specific chiralities were retained and a maximum of 32 species per ligand were kept.

#### Protein Preparation

PDB files were imported to Maestro Suite (Schrödinger 2020–1/2024–1) and prepared using the Protein Preparation Wizard/Workflow. In brief, bond orders were assigned, hydrogens were added, disulfide bonds were generated, missing side chains and loops were filled using Prime and het states were generated using Epik for pH 7.0 ± 2.0. Afterward, the structure was manually fixed upon problem identification. H-bond assignments were performed, waters removed beyond a 3.0 Å radius of het groups and a restrained minimization was performed using the OPLS3e force field, converging the heavy atoms to an RMSD of 0.30 Å.

#### Ligand Docking

The docking grid was generated using the prepared structure of **mOGG1–1 (TH12163)**. The ligand was chosen as the center of a 10 Å × 10 Å × 10 Å box for ligand docking. No other restrictions were made. The compound was docked using the standard docking protocol (Glide SP) without restrictions. Individual poses were inspected and compound prioritized upon selection.

### Molecular Dynamics

Molecular dynamics simulations were performed on OGG1-DNA-**30** and OGG1-DNA-**38** models, starting from crystallographic structure of hOGG1 complexed with excised DNA, covalently bound to Lys249 Schiff’s base, and 8-oxoG (PDB code 1HU0).^61^ In the investigated systems, 8-oxoG was replaced by **30** or **38** via docking calculations performed with AutoDock vina 1.1.2 suite,^107^ in agreement with protocol adopted in previous investigations.^108–110^ The grid box’s center was positioned at coordinates x = 18.483 Å, y = 19.538 Å, z = 34.466 Å, with dimension box of 20 × 20 × 20 Å^3^. The lowest energy docked pose was used for subsequent MD simulations. Full geometry optimization of species **30** and **38** was carried out at HF/6–31G* level of theory, to generate parameters. General amber force field (GAFF)^111^ and restrained electrostatic potential (RESP)^112^ methods were adopted to obtain nonbonding parameters and charges. The same protocol was used to extrapolate parameters for the excised DNA-Lys249 system, in proximity to the covalent bond between the excised base and the Schiff base. A list of parameters is provided in Supporting Information. OGG1:DNA-**30** and OGG1-DNA-**38** were placed in an orthorhombic box (10 Å from the protein), adding TIP3P water molecules and 29 Na^+^. Overall, the models consisted of 54647 atoms. Protein and DNA were treated with ff14SB^113^ and OL15 ^114^ force fields, respectively. After an initial minimization, each system was progressively heated from 0 to 298 K over 5 ns and then equilibrated for further 5 ns, using Langevin thermostat in NVT ensemble (*T* = 298 K). The production phase consisted of 2 × 200 ns of molecular dynamics simulations for OGG1-DNA-**30** and OGG1-DNA-**38** systems, selecting 2 fs integration steps, using the SHAKE algorithm, Berendsen barostat in NPT ensemble (*p* = 1 bar) and a time constant τp = 2.0 ps. In all simulations, electrostatic potential calculations were managed under Particle Mesh Ewald summation method, with long-range electrostatic interactions computed using a 12 Å cutoff distance. Amber 2020 software package was adopted to perform all the simulations.^90^ To identify 10 representative conformations during the molecular dynamics simulations, RMSD-based clustering of entire trajectories was performed, by RMS-fitting of the positions of the Cα atoms and adopting average linkage clustering algorithm as implemented in *cpptraj* module.^115^

## Supplementary Material















## Data Availability

The research data supporting this publication can be accessed at 10.17630/6bca1a78-19d6-4567-a7ee-c99900865cfb. The atomic coordinates and structure factors (codes 9FNV, 9FNU) have been deposited in the Protein Data Bank (www.wwpdb.org/).

## Notes

Supplementary figures and tables with associated biochemistry, computational, and crystallographic methods, chemistry experimental for starting material synthesis, LCMS traces for compounds used in biological assays, and NMR spectra for all compounds (PDF)
Molecular formular strings and activities (CSV)
Parameters in AMBER format for OGG1-DNA-30 and OGG1-DNA-38 (PDF)
Ligand-bound molecular docking structures of OGG1–32 and OGG1–35 (PDB)
Ligand-bound clustered molecular dynamics structures of OGG1–30 and OGG1–38 (PDB)
Crystal structure of an HOGG1-DNA borohydride trapped intermediate complex (PDB)
Ligand-bound molecular docking structures (PDB)

## Abbreviations

A: adenine
AB: apical-basolateral
Ac: acetyl
AC_50_: half-maximal activation concentration
AP: apurinic/apyrimidinic
APE1: apurinic/apyrimidinic endonuclease 1
aq: aqueous
Ar: aryl
ATR: attenuated total reflectance
AUC: area under curve
BA: basolateral-apical
Bn: benzyl
Boc: *tert*-butoxycarbonyl
C: cytosine
CI: confidence interval
Cl_int_: intrinsic clearance
clogP: calculated logarithm of partition coefficient
Cy: cyclohexyl
DAB: dabcyl quencher-3′
DMEM: Dulbecco’s Modified Eagle Medium
DMF: dimethylformamide
DMSO: dimethyl sulfoxide
DNA: DNA
DTP: Developmental Therapeutics Program
E1: elimination unimolecular
EDTA: ethylenediaminetetraacetic acid
EI: electron impact
EMSA: electrophoretic mobility shift assay
ESI: electrospray ionization
FAM: 6-carboxyfluorescein
G: guanine
GAFF: general amber force field
h: human
HMBC: heteronuclear multiple bond correlation
HPLC: high-performance liquid chromatography
HRMS: high resolution mass spectrometry
IC_50_: half-maximal inhibitory concentration
IR: infrared
*K*_a_: acid dissociation constant
*k*_el_: elimination rate constant
LCMS: liquid chromatography mass spectrometry
LE: ligand efficiency
LLE: ligand-lipophilicity efficiency
M: molar
m: murine
MASH: metabolic dysfunction-associate steatohepatitis
MD: molecular dynamics
Me: methyl
MPLC: medium pressure liquid chromatography
NADPH: nicotinamide adenine dinucleotide phosphate
NCI: National Cancer Institute
NIH: National Institutes of Health
NMR: nuclear magnetic resonance
NPT: isothermal–isobaric ensemble
Nth: endonuclease III
NVT: canonical ensemble
OGG1: 8-oxoguanine DNA glycosylase 1
ORCA: organocatalytic switch
P-gp: P-glycoprotein
PAGE: polyacrylamide gel electrophoresis
PAINS: pan-assay interference compounds
P_app_: apparent permeability
PBS: phosphate-buffered saline
PDB: Protein Data Bank
Ph: phenyl
PivOH: pivalic acid
PBMC: peripheral blood mononuclear cell
PNKP1: polynucleotide kinase-phosphatase 1
ppm: parts per million
PTFE: polytetrafluoroethylene
PUA: phospho-α,β-unsaturated aldehyde
REOS: rapid elimination of swill
RESP: restrained electrostatic potential
RDF: radial distribution function
RMSD: root-mean-square deviation
RMSF: root-mean-square fluctuation
RPM: revolutions per minute
RT: room temperature
SAR: structure–activity relationship
SEM: standard error of the mean
SMILES: Simplified Molecular Input Line Entry System
T4 PNK: T4 polynucleotide kinase
T: thymine
t 1/2: half-life
*t*-Bu: *tert*-butyl
TEER: transepithelial electrical resistance
TFA: trifluoroacetic acid
THF: tetrahydrofuran
TLC: thin-layer chromatography
U: uracil
UDG: uracil DNA glycosylase
UV: ultraviolet
wt: wildtype.
