# Supplementary material for: Giving an Enzyme Scissors: Serotonin Derivatives as Potent Organocatalytic Switches for DNA Repair Enzyme OGG1
Source: J Med Chem. 2025 Oct 15;68(21):22455–83. doi: 10.1021/acs.jmedchem.5c01454 (PMC12621200; doi:10.1021/acs.jmedchem.5c01454)

Supporting Information

Giving an Enzyme Scissors: Serotonin Derivatives as Potent Organocatalytic Switches of DNA Repair Enzyme OGG1.

Marek Varga,* Florian Ortis, Alicia del Prado, Alice Eddershaw, Emma Scaletti Hutchinson, Emily C. Hank, Kaixin Zhou, Natálie Rudolfová, Alessia Dodaro, Elisée Wiita, Ingrid Almlöf, Stella Karsten, Kirill Mamonov, Sara H. Ahmed, Kirsty Bentley, Olov Wallner, Evert J. Homan, Martin Scobie, Thomas Helleday, Pål Stenmark, Mario Prejanò, Miguel de Vega, Allan J. B. Watson,* and Maurice Michel*

Contents

S1. SUPPLEMENTARY FIGURES 2
S2. SUPPLEMENTARY TABLES 11
S3. BIOCHEMISTRY METHODS 19
S4. CHEMISTRY METHODS AND CHARACTERIZATION 22
S5. REFERENCES 52
S6. LCMS TRACES 54
S7. NMR SPECTRA 77

S1. Supplementary Figures

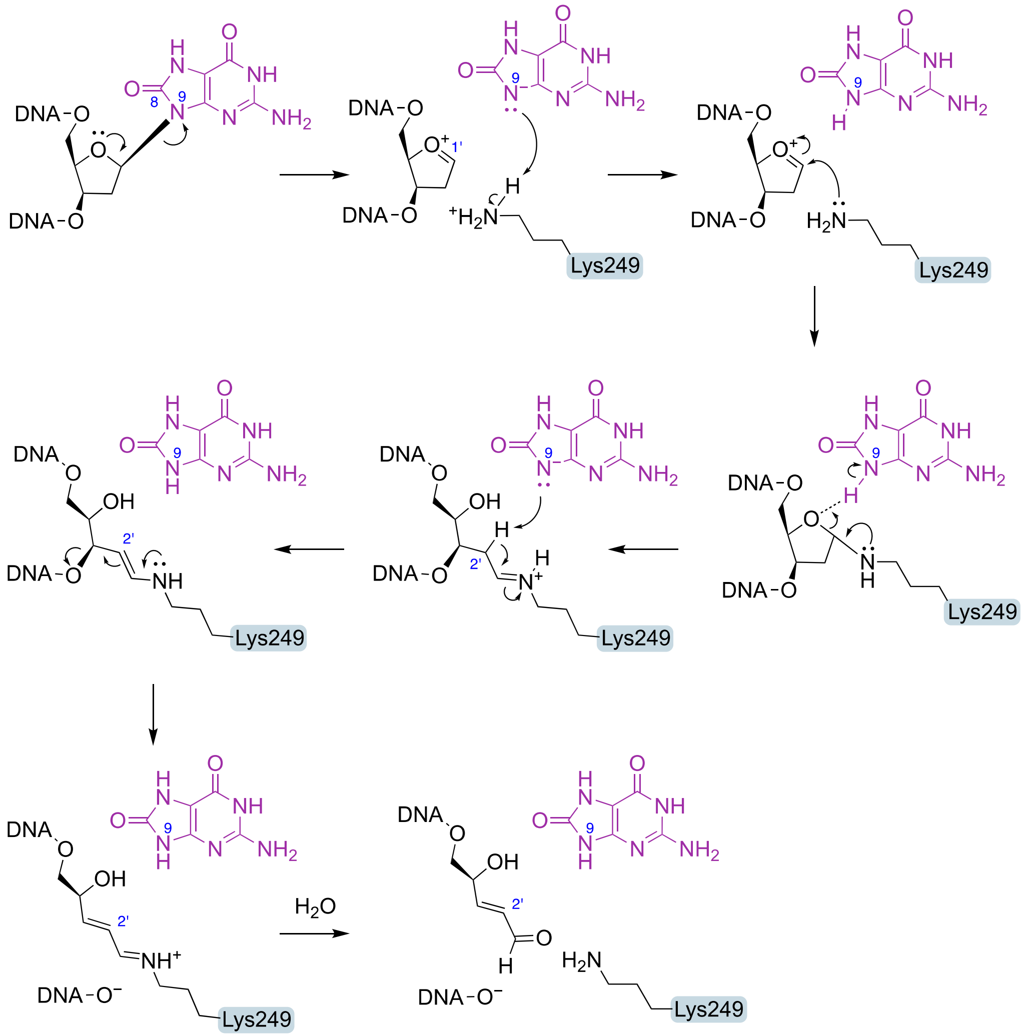

Figure S1. Plausible mechanism of 8-oxoG/OGG1 catalyzed cleavage of AP sites.

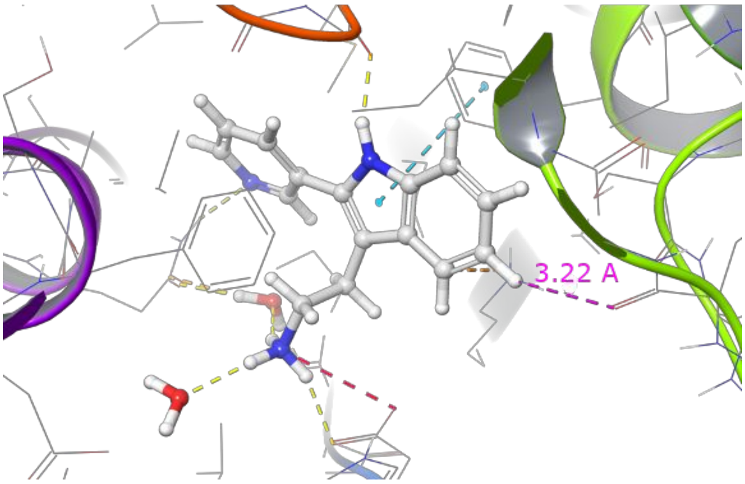

Figure S2. Close-up of binding site in the mOGG1-**1** co-crystal structure. **1** and water molecules are depicted as stick models, with C atoms grey, N blue, H white, O red. mOGG1 is depicted as a cartoon model with amino acids shown as thin sticks. H-bonding indicated by dashed yellow lines, ionic interaction by red dashed line, π-stacking by a light blue dashed line. The distance between H5 of **1** and N150 of mOGG1 is depicted by a pink dashed line.

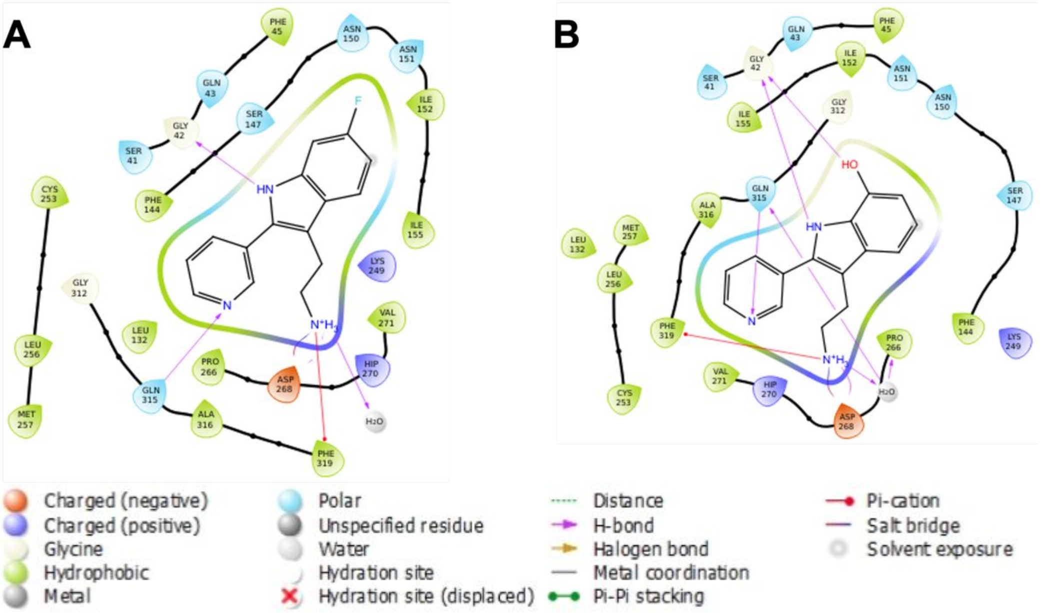

Figure S3. 2D interaction diagrams of ligands **35** (**A**) and **32** (**B**) in complex with mOGG1 generated by molecular docking using mOGG1-**30** co-crystal structure (PDB ID: 9FNU).

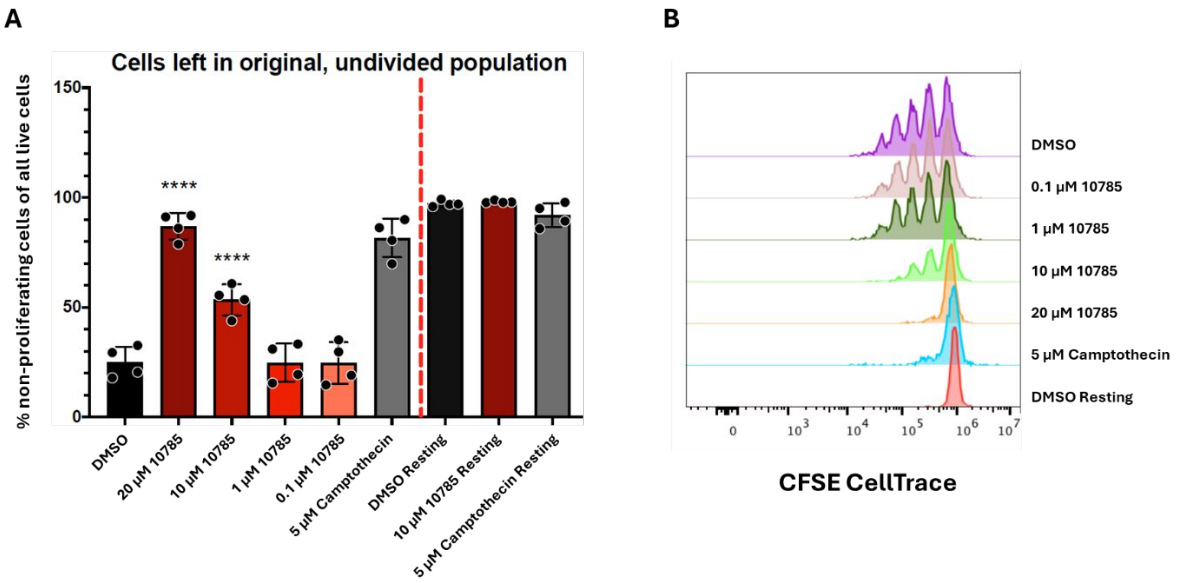

Figure S4. TH10785 does not compromise CD3/CD28/IL-2 activation and proliferation of human T cells at up to 1 μM but suppresses proliferation in a dose-dependent manner at 10 and 20 μM. (**A**) Percentage of cells left in the original, undivided population after 96 h of simultaneous activation and treatment. Only live cells analyzed, selected as Sytox blue negative cells. (**B**) Example of the proliferation plots quantified in (A), where the right-most peak (or single red for DMSO Resting) represents the undivided population. Significance in (A) shown as compared to DMSO with one-way ANOVA. Data from 2 donors with duplicates; n = 4.

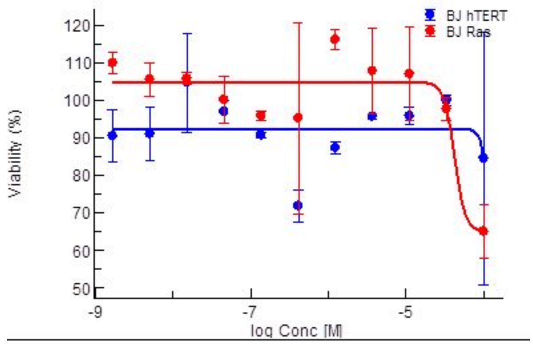

Figure S5. Viability of BJ hTERT (blue) and BJRas (red) cell lines *vs*. log [**30**]. Method described in section S3.

A

5'[32]P-GTACCCGGGGATCCGTAA**8**GCGCATCAGCTGCAG-3'
CATGGGCCCCTAGGCATTCCGCGTAGTCGACGTC

B

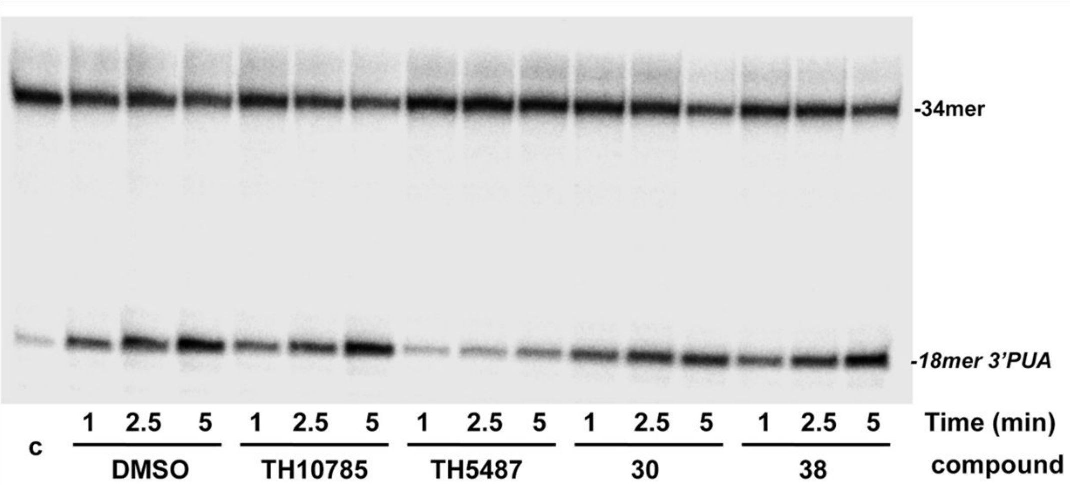

Figure S6. Analysis of the effect of ORCAs on the glycosylase activity of *h*OGG1. (**A**) The [^{32}P]5'-labeled 8-oxoG-containing DNA substrate used. (**B**) 2 nM of the DNA substrate was incubated with 500 pM *h*OGG1 and either 10% DMSO, 6.25 μM TH10785, 50 μM TH5487, 6.25 μM **30** or 10 μM **38**, as specified. After incubation for the indicated times, the reaction was stopped by addition of 0.1 M NaOH and further incubation at 90 ºC for 5 min. Samples were analyzed by 7M urea-20% PAGE and visualized with an Amersham Typhoon scanner. Position of products is indicated. *c:* control without protein and 10% DMSO.

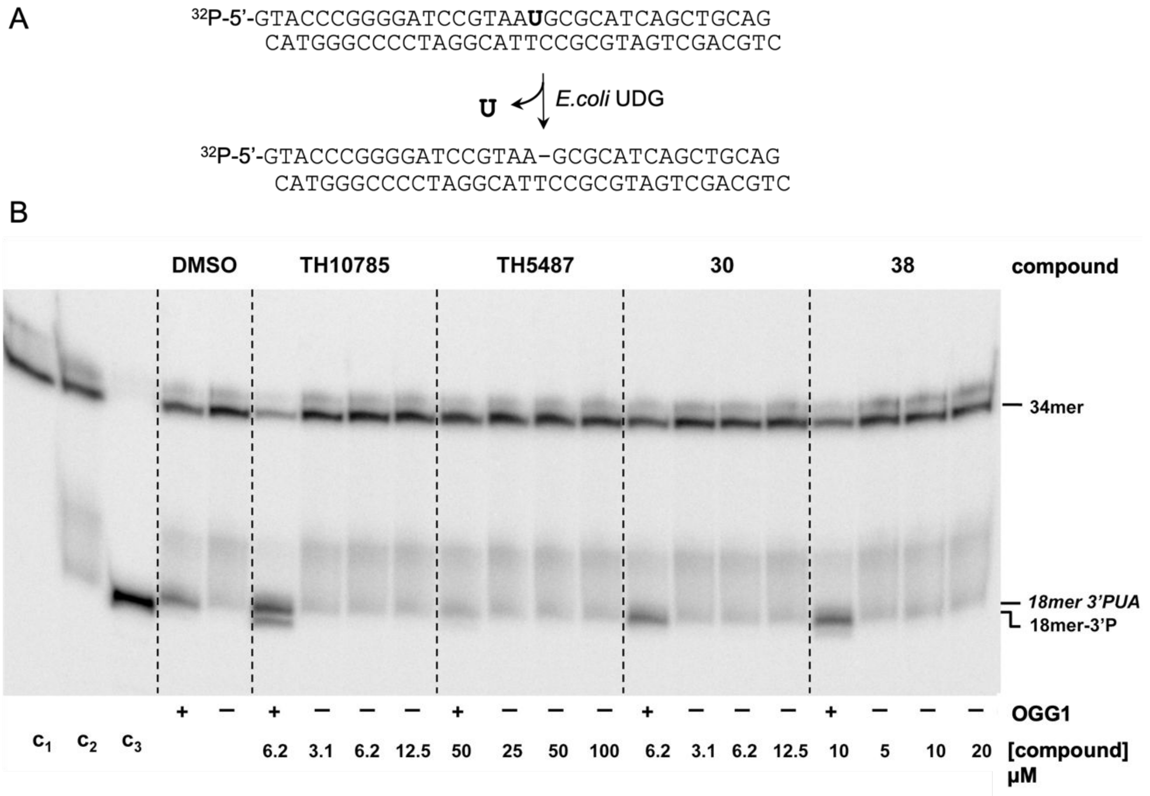

Figure S7. Analysis of the effect of ORCAs on the cleavage of AP sites. (**A**) The [^{32}P]5'-labeled AP-containing DNA substrate used, generated from a uracil-containing substrate treated with *E.coli* UDG to leave an intact AP site. (**B**) 1 nM of the [^{32}P]5'-labeled substrate was incubated in the absence (-) or presence (+) of 75 nM *h*OGG with either 10% DMSO or the specified concentration of the indicated compound. After incubation for 4 min at 37 ºC, reactions were stopped and the products resolved by 7M urea-20% PAGE and visualized with an Amersham Typhoon scanner. Position of products is indicated. c1: DNA control without UDG; c2: DNA control treated with UDG; c3: alkaline hydrolysis of the DNA control treated with UDG.

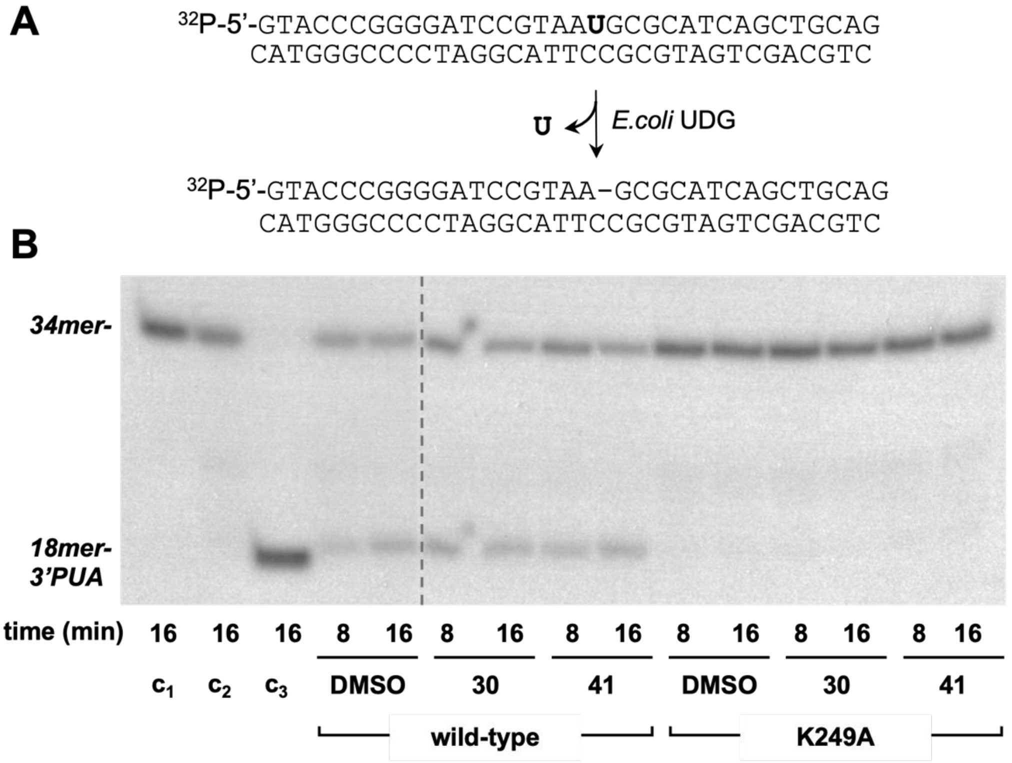

Figure S8. Effect of tryptamine ORCAs on the AP-lyase activity of OGG1. (**A**) The [^{32}P]5'-labeled uridine-containing substrate was pre-incubated with 0.2 U of *E. coli* UDG to obtain a natural AP site. (**B**) 1 nM of the AP-site-containing DNA substrate was incubated with 75 nM of either the wildtype or K249A mutant OGG1 and either 10% DMSO or 6 µM of the indicated compounds at 37 °C for the indicated times. Products were resolved by 7M urea-20% PAGE and visualized by autoradiography. Position of products is indicated. c1: control without UDG; c2: control with UDG; c3: control with UDG and NaOH.

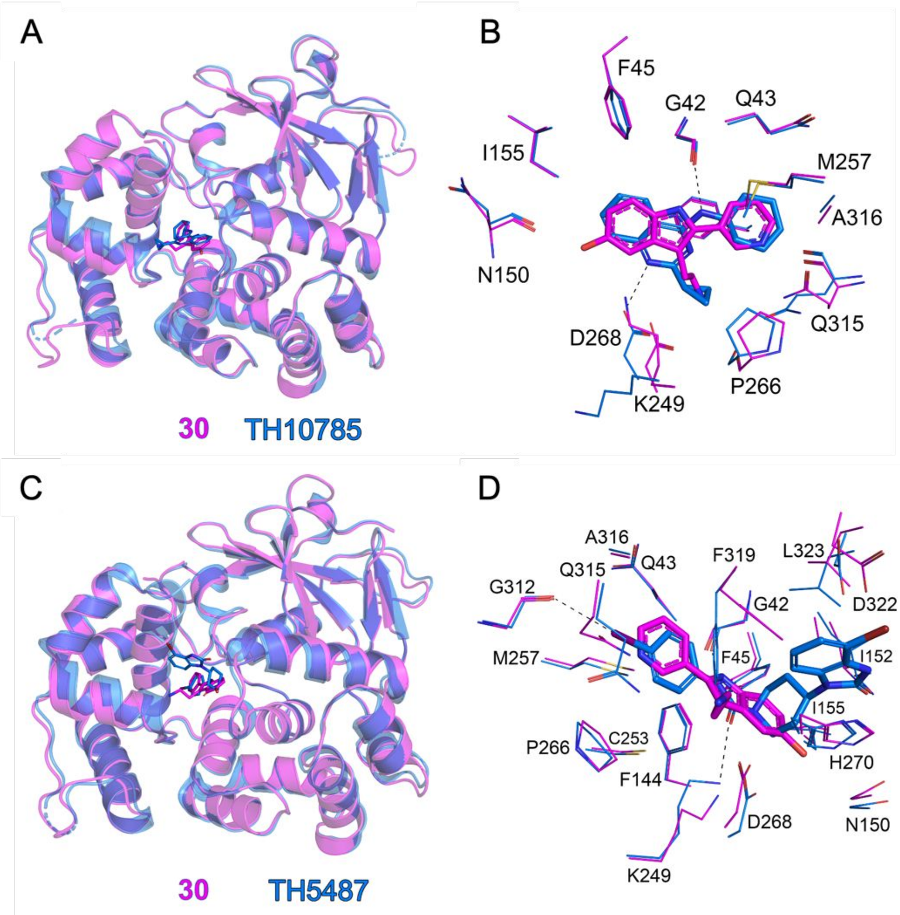

Figure S9. (**A**) Superposition of mOGG1-**30** (magenta) and hOGG1-TH10785 (blue, PDB ID: 7AYY) monomers. (**B**) Close-up comparison of ligand binding between the structures in (A). (**C**) Superposition of mOGG1-**30** (magenta) and hOGG1-TH5487 (blue, PDB ID: 6RLW) monomers. (**D**) Close-up comparison of ligand binding between the structures in (C). Ligands are depicted as sticks; C atoms are colored magenta (**30**) or blue (TH10785, TH5487), O atoms red, N atoms dark blue, and I atoms purple. Amino acids which contribute to ligand positioning are depicted as thin sticks. The hydrogen bond networks for hOGG1-TH10785 and hOGG1-TH5487 are indicated with dashed lines. Figure produced using PyMOL (v.2.3.3, Schrödinger).

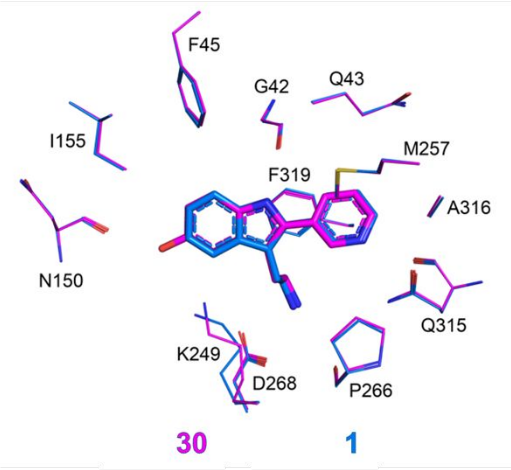

Figure S10. Active site comparison of mOGG1-**30** (magenta) and mOGG1-**1** (blue). Ligands are depicted as sticks; C atoms are colored magenta (**30**), or blue (**1**), O atoms red and N atoms dark blue. Amino acids which contribute to ligand positioning are depicted as thin sticks. Figure produced using PyMOL (v.2.3.3, Schrödinger).

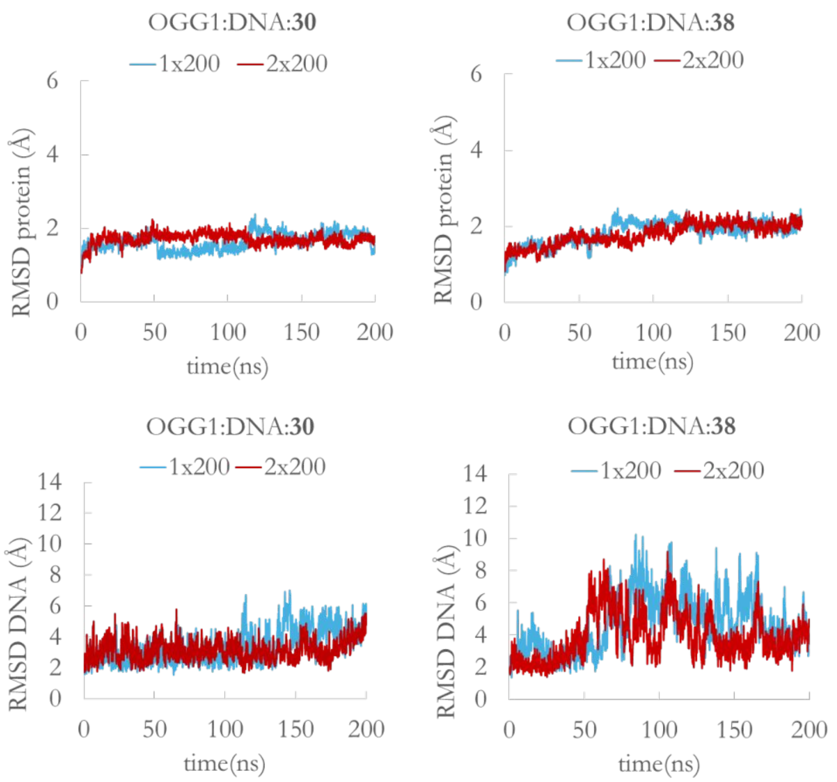

Figure S11. RMSD analysis of protein backbone and DNA, obtained from MD simulations of OGG1-DNA-**30** and OGG1-DNA-**38** systems.

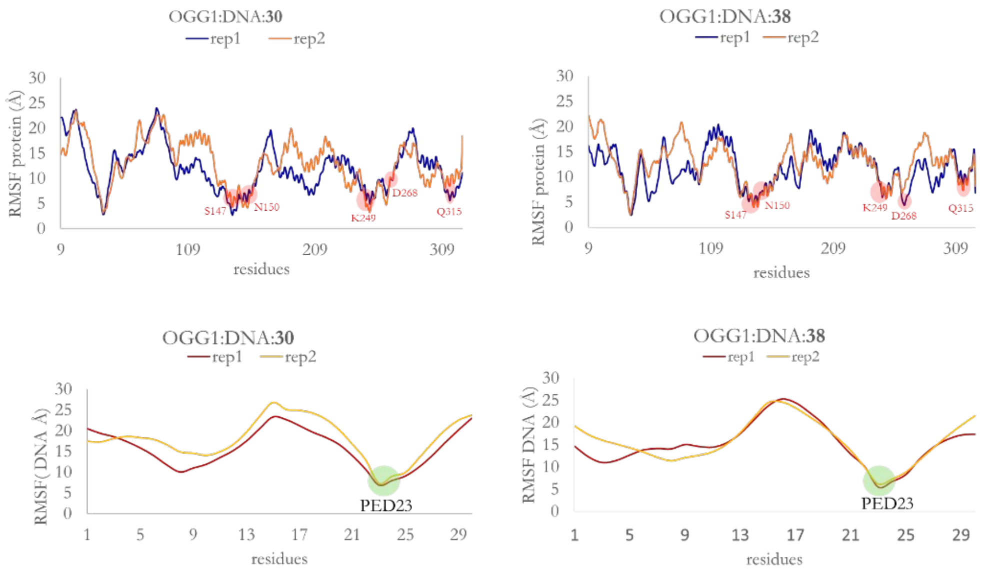

Figure S12. RMSF analysis of protein backbone and DNA, obtained from MD simulations of OGG1-DNA-**30** and OGG1-DNA-**38** systems.

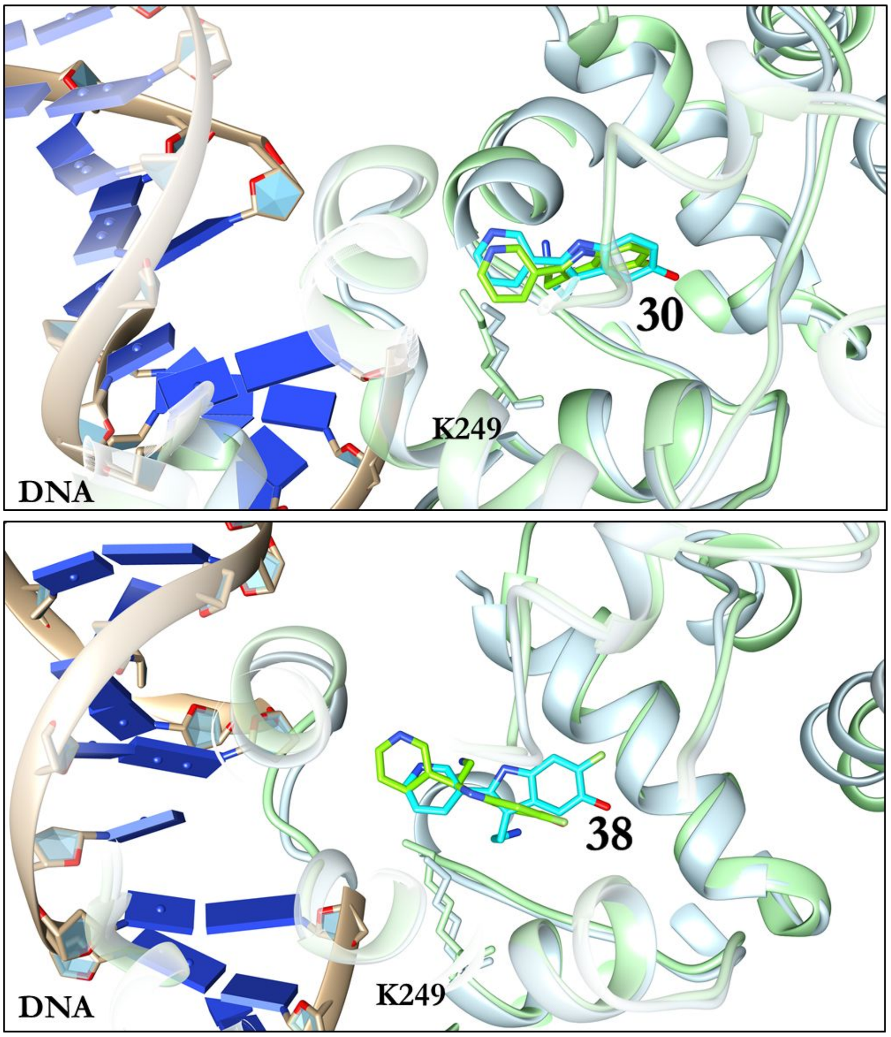

Figure S13. Superposition of initial docked structures (ligand in cyan) and most representative clustered structures (ligand in green) obtained from MD trajectories of OGG1-DNA-**30** (*top*) and OGG1-DNA-**38** (*top*) systems.

S2. Supplementary Tables

Table S1. Results of biochemical assay of virtual screening hits.

NSC ID	SMILES	Structure	Glide XP score (kcal/mol)	AC_{50} (μM)	# HA	LE
186216	OC=1CC(C)(C)C=C(C12)NC=3C(=CC(Cl)=CC3)N[C@@H]2C4=CC=CC=C4	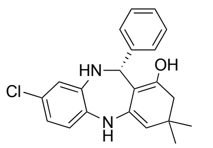	-11.14	inactive	25	0.45
4232	[O]C(=O)C1=CC=CC(=C1)N[C@H](C2=CC=CC=C2)C(=O)C3=CC=CC=C3	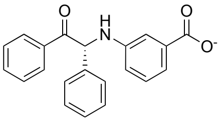	-10.96	inactive	25	0.44
34924	C1=CC=CC=C1C(=O)[C@@H](C2=CC=CC=C2)NC(=CC=3)C=C(C34)C=CC=C4	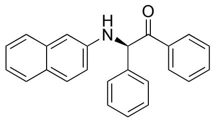	-10.90	inactive	26	0.42
674639	FC(F)(F)C(=O)NCCC(C(C1=2)=CC=CC2)=C([NH]1)C3=CC=CC=C3	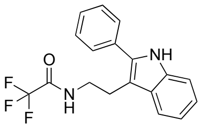	-10.58	inactive	24	0.44
210448	N1=C(N)[NH]C(=O)C(=C1C)CCCNC2=CC=CC(=C2)C(F)(F)F	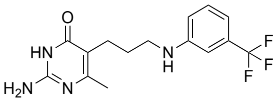	-10.45	inactive	23	0.45
247010	COC1=CC(OC)=C(C=C1Cl)NC(=O)C[C@@H]2SC(N)=NC2=O	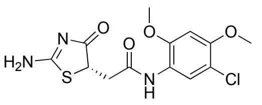	-10.37	inactive	22	0.47
263759	CC(=C1)C(=O)N([C@H]12)C3C(=CC=CC=3)N[C@@H]2C4=CC=CC=C4	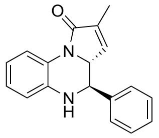	-10.37	inactive	21	0.49
123043	O=C1CCCC[C@H]1[C@H](C2=CC=CC=C2)NC3=CC=CC=C3	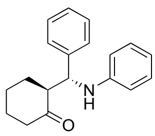	-10.10	inactive	21	0.48

655348	COC1=CC=CC=C1N2[C@@H](SCC2=O)C(=C[NH]3)C(C3=4)=CC=CC4	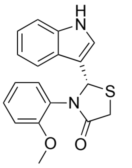	-10.07	inactive	23	0.44
202376	C1C=CC=C(C=12)C(=C[NH]2)C[C@@H](N)C(=O)OCC3=CC=CC=C3	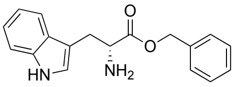	-9.99	inactive	22	0.45
13240	C1C=CC=C(C=12)C(CC[NH3+])=C([NH]2)C3=CC=CN=C3	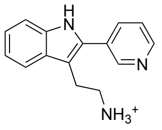	-9.91	4.7 uM	18	0.55
29454	C1C=CC=C(C=12)C(C[NH+](C)C)=C([NH]2)C3=CC=CC=C3	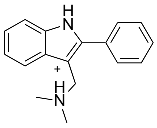	-9.86	inactive	19	0.52
210777	O=C1[NH]C=C(C(=O)[NH]1)CC2=CC=CC(=C2)OCC3=CC=CC=C3	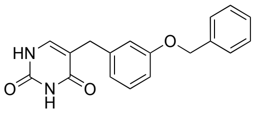	-9.86	inactive	23	0.43
42087	C1C=CC=C(C=12)C(=O)O[C@H]2C(=C[NH]3)C(C3=4)=CC=CC4	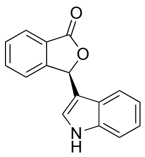	-9.78	inactive	19	0.51
136042	C1=CC=CC(C12)=C(C(=O)N=2)NCCC(=C[NH]3)C(C3=4)=CC=CC4	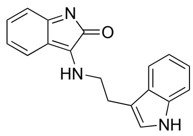	-9.70	inactive	22	0.44
338155	C1C=CC=C(C=12)[C@@H](O)N(C2=O)CCC(=C[NH]3)C(C3=4)=CC=CC4	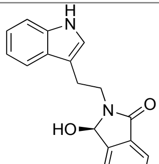	-9.69	inactive	22	0.44
669300	C1CCCCC1NC(=O)[C@@H](N2C(=O)C)CS[C@H]2C3=CC=CC=C3	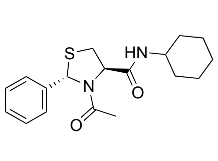	-9.68	inactive	23	0.42

364164	C1C=CC=C(C=12)N([C@H](NC)NC2=O)CC3=CC=C(F)C=C3	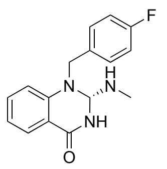	-9.66	inactive	21	0.46
645796	C1=CC=CC=C1[C@H]([C@H]([C@H]23)CCCO2)NC=4C3=CC=CC4	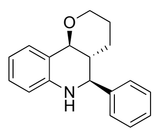	-9.53	inactive	20	0.48
202050	C=1C=C(C)C=C(C12)NC3C(C(=O)N2)=CC=CC=3	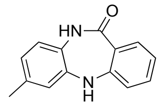	-9.45	inactive	17	0.56
637579	C1=CC=CC=C1[C@H](C(=C23)C(=O)O[C@@H](C)C2)NC=4C(N3)=CC=CC4	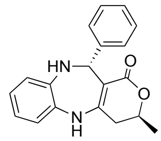	-9.41	inactive	23	0.41
158389	CCOC1=CC=CC=C1[C@@H](NC2=O)NC(C2=3)=CC=CC3	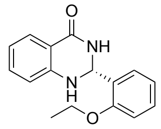	-9.40	inactive	20	0.47
522258	[NH3+]CCC(=C[NH]1)C(C1=2)=CC=CC2	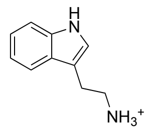	-9.39	inactive	12	0.78
26933	CC1=CC=C(C=C1)N[C@H](C2=CC=CC=C2)C(=O)C3=CC=CC=C3	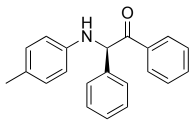	-9.35	inactive	23	0.41
56385	N1=C[NH]C(C12)=NC(C(F)(F)F)=NC=2NC3=CC=CC=C3	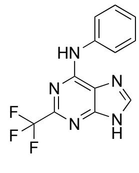	-9.34	inactive	20	0.47
175633	ClC1=CC=CC=C1[C@H](NC2=O)NC(C2=3)=CC=CC3	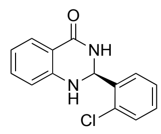	-9.33	inactive	18	0.52

364889	CCOC(=O)C(=CC=1)C=C(C12)NCC3=C(O2)C(OC)=CC=C3	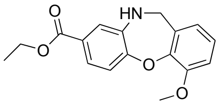	-9.33	inactive	22	0.42
128331	NC(=O)[C@@H](O)C(=C[NH]1)C(C1=2)=CC=CC2	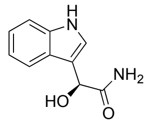	-9.33	inactive	14	0.67
97069	C[C@@H]([NH3+])CC(=C[NH]1)C(C1=2)=CC=CC2	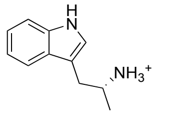	-9.33	inactive	13	0.72
147943	C1=CC=CC=C1[C@@H](C2)[C@@](O)(C(C2=3)=CC=CC3)CC4=CC=CC=N4	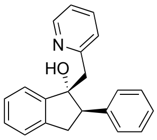	-9.30	inactive	23	0.40
213798	NC(=O)[C@@H]1CCCN(C1)C(=O)NC2=CC=CC=C2F	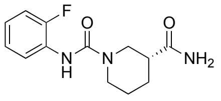	-9.29	inactive	19	0.49
642717	C1C=C(C)C[C@@H](C(=O)C)[C@]12C3C(NC2=O)=CC=CC=3	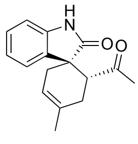	-9.28	inactive	19	0.49
62573	ClC1=CC=C(C=C1Cl)NC2=CC(=O)[NH]C(=O)[NH]2	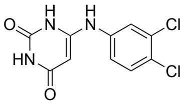	-9.26	inactive	17	0.54
144033	C=1C=CC=C(C12)N=C([NH]2)C(=CC=C3)C(C3=4)=CC=CC4	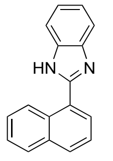	-9.25	inactive	19	0.49
186218	C1=CN=CC=C1[C@@H](C(C23)=C(O)CCC=2)NC=4C(N3)=CC=CC4	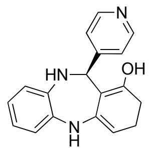	-9.22	inactive	22	0.42

133478	C1C=CC=C(C=12)C(=O)C(=C(C)[NH]2)C[NH2+]CC3=CC=CC=C3	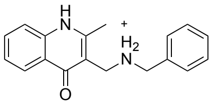	-9.22	inactive	21	0.44
32474	NC(=O)[C@@H]1CCC[N@@H+](C1)CC(=C[NH]2)C(C2=3)=CC=CC3	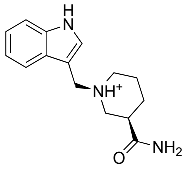	-9.21	inactive	19	0.48
14383	CN1C(=O)N(C)C(=O)C(=C12)[NH]C(=N2)C3CCCCC3	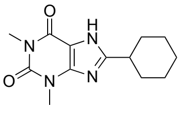	-9.17	inactive	19	0.48
50457	C[C@@H]([NH3+])CC(=C[NH]1)C(C1=2)=CC(Cl)=CC2	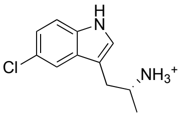	-9.16	inactive	14	0.65
643518	C=1C=CC=C(NC2=O)C1[C@]23CC[NH2+][C@@H]3CC4=CC=CC=C4	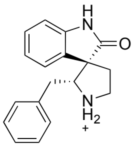	-9.12	weak	21	0.43

Table S2. X-ray crystallography data collection and refinement statistics

	**mOGG1-1 (TH12163)**	**mOGG1-30 (TH13579)**
**Data collection**		
PDB code	9FNV	9FNU
Station	MAXIV-BioMAX	MAXIV-BioMAX
Space group	$P2_12_12_1$	$P2_12_12_1$
Cell dimensions:		
a, b, c (Å)	81.4, 81.4, 170.1	80.8, 81.1, 170.1
α, β, γ (°)	90, 90, 90	90, 90, 90
Resolution (Å)	73.4-2.25 (2.29-2.25)	73.2-2.00 (2.04-2.00)
Total reflections	731699 (37264)	1024395 (60869)
Unique reflections	54738 (2676)	76437 (4487)
R_{merge}	10.7 (137.6)	9.1 (178.2)
R_{pim}	4.2 (52.9)	3.7 (72.1)
$CC_{1/2}$ (%)	99.9 (73.6)	99.7 (72.7)
I/σ	15.2 (2.1)	13.7 (1.8)
Completeness	100 (100)	100 (99.9)
Redundancy	13.4 (13.9)	13.4 (13.6)
**Refinement**		
R_{work}/R_{free} (%)	20.0/25.2	21.7/25.6
B-factors:		
Protein (all atoms)[a]	47.5/67.0/66.1	46.9/63.8/68.2
Ligand [a]	36.9/43.0/50.7	38.0/51.3/54.3
Water	47.4	45.9
R.m.s. deviations:		
Bond lengths (Å)	0.010	0.010
Bond angles (°)	1.57	1.40
Ramachandran statistics:		
Favoured (%)	98.6	99.3
Outliers (%)	1.4	0.7

Values in parentheses are for the highest-resolution shell. [a] Values for each monomer (A, B and C) of the asymmetric unit. An x indicates that a ligand is not bound in that specific monomer.

Table S3. Docking scores of selected compounds.

Compound #	Structure	Docking Score	Compound #	Structure	Docking Score
**35**	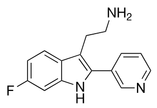	-10.62	**30**	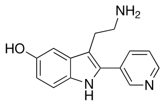	-10.04
**9**	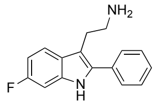	-10.53	**4**	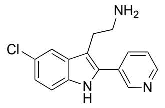	-10.04
**36**	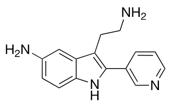	-10.53	**32**	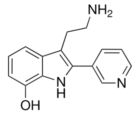	-9.68
**33**	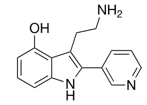	-10.46	**3**	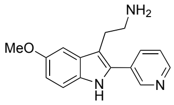	-9.68
**2**	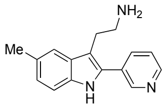	-10.45	**7**	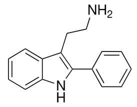	-9.67
**8**	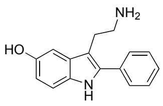	-10.39	**1**	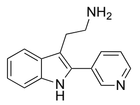	-9.66
**31**	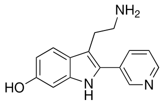	-10.22			

Table S4. Selectivity experiments on other DNA glycosylases and NUDIX protein family members. The assay was performed as described in the Experimental section using compound **30** at a concentration of 99 µM. X* is an AP site targeting PAINS (pan-assay interference compound), that stalls AP sites. Selectivity assays were performed alongside compounds reported in Hank *et al.*[1] and Kehler *et al.*[2] Inhibition reference values are thus identical to the ones reported in these earlier publications.

Protein ID	Compound 30 [inhibition]	Reference [inhibition]	Reference
**NUDT5**	No effect	99%	TH5427
**NUDT22**	No effect	93%	TH8525
**NUDT15**	No effect	96%	TH1760
**MTH1**	34%	91%	TH1579
**NEIL1**	13%	90%	X*
**APE1**	No effect	93%	X*
**UNG**	31%	95%	X*
**NTHL1**	25%	71%	X*
**MPG**	17%	88%	X*
**TDG**	55%	83%	X*

Table S5. Number of water molecules within 5 Å of ligands **30** and **38** during the molecular dynamic simulation. Water molecules were counted for each structure obtained by clustering analysis.

OGG1:DNA:**30**

**1x200**			**2x200**		
**Cluster**	**Frequency %**	**N of H_2O**	**Cluster**	**Frequency %**	**N of H_2O**
1	49.1	7	1	31.8	7
2	18.4	9	2	27.4	7
3	10.5	5	3	15.5	7
4	4.7	12	4	14.2	7
5	4.6	7	5	3.3	7
6	4.6	6	6	3.2	10
7	4.1	12	7	2.3	7
8	2.3	6	8	1.3	8
9	1.7	6	9	0.9	9
10	0.3	8	10	0.4	6

OGG1:DNA:**38**

**1x200**			**2x200**		
**Cluster**	**Frequency %**	**N of H_2O**	**Cluster**	**Frequency %**	**N of H_2O**
1	37.7	14	1	44.8	16
2	24.2	15	2	38.6	8
3	16.2	15	3	5.5	7
4	6.9	14	4	2.8	9
5	6	13	5	1.9	9
6	3.9	17	6	1.9	9
7	2.2	13	7	1.7	9
8	1.6	12	8	1.1	7
9	0.8	9	9	0.9	11
10	0.6	16	10	0.9	12

S3. Biochemistry Methods

Crystallization

Purified mOGG1 (22 mg/mL) was pre-incubated with 6.25 mM ligand (TH12163 or TH13579) and crystallized via sitting drop vapor diffusion in conditions from the Morpheus Screen (Molecular Dimensions). This included 0.12 M Alcohols, 0.1 M imidazole/MES pH 6.5, 50 % EDO_P8K (mOGG1-TH12163) or 0.12 M Ethylene Glycols, 0.1 M sodium HEPES/MOPS pH 7.5, 50 % GOL_P4K (mOGG1-TH13579) at 18 °C. Protein crystals were fished without additional cryoprotectant and flash frozen in liquid nitrogen.

Data Collection, Structure Determination, and Refinement

X-ray diffraction data were collected at BioMAX (Lund, Sweden) equipped with an EIGER X 16M detector. Complete datasets were collected on single crystals at 100 K. The datasets were processed and scaled with XDS[3] and AIMLESS[4] within the CCP4 suite.[5] Molecular replacement was performed using MOLREP[6] using the structure of mouse OGG1 (PDB ID: 6G3Y) with all ligands and waters removed, as the search model. Several rounds of manual model building and refinement were performed using COOT[7] and REFMAC5[8] during which waters and ligands were incorporated into the structures. The coordinates and structure factors for mOGG1-**1** (mOGG1-TH12163) and mOGG1-**30** (mOGG1-TH13579) were deposited in the Protein Data Bank (PDB) under the accession codes 9FNV and 9FNU, respectively.

Selectivity Screen Against Other Glycosylases and NUDIX Enzymes

The compounds were tested at a concentration of 99 µM in biochemical assays following a similar protocol as for the OGG1 biochemical readout, using the respective enzyme and the labelled enzyme substrate. Glycosylase inhibition was monitored in a kinetic mode and calculated in percent in relation to the DMSO control. The experiment was performed in duplicates for each compound. For NUDT5, NUDT15 and NUDT22, a malachite green assay was conducted, using a previously described protocol.[9–11]

Activated Peripheral Blood Mononuclear Cell Proliferation Assay

Human T cells were isolated fresh from the buffy coats of healthy donors via the Karolinska Hospital in Stockholm, Sweden. They were enriched using RosetteSep™ Human T Cell Enrichment Cocktail (15028, StemCell technologies), SepMate™-50 separation tubes (85458, StemCell technologies) and density media Ficoll-Paque Plus (17-1440-03, Ge Healthcare), and stained with 1:1000 CFSE CellTrace (C34554, Thermo Fisher Scientific) in PBS, according to the manufacturer's instructions. The T cells were cultivated in RPMI 1640 + Glutamax medium (61870-044, Thermo Fisher Scientific)

supplemented with 10 % FBS (10500064, Thermo Fisher Scientific) and 50 U/mL penicillin/streptomycin (15070063, Thermo Fisher Scientific) at 37 °C and 5% CO_2. Cells were typically activated within 24 h of separation, with Dynabeads™ Human T-Activator CD3/CD28 (11131D, StemCell technologies) using 0.75 beads per cell, and 25 ng/µL IL-2 (PHC0027, Thermo Fisher Scientific) and simultaneously treated with either TH10785 at indicated concentrations, 5 µM camptothecin, or DMSO. Activated T cells were seeded at a concentration of 0.8 million/mL cells into round-bottomed 96 well plates (83.3925.500, Sarstedt). Resting T cells were treated with either DMSO, 10 µM TH10785, or 5 µM camptothecin and were kept in culture at 1–2 million/mL. Cells were then washed with CellWASH (349524, Becton Dickinson) and stained with Sytox blue (S34857, Thermo Fisher Scientific) according to the manufacturer's instructions. Cells were counted using a TC20™ Automated Cell Counter (Bio-Rad) and 0.4% Trypan blue (1450021, Bio-Rad). Flow cytometry was analyzed using FlowJo™ (Becton Dickinson, Franklin Lakes, NJ, United States).

Resazurin Cell Viability Assay with BJ-hTERT and BJ-hTERT SV40RAS Cells for Toxicity Testing in a Dose-Response Manner

Prior to cell-seeding, compound **30** was dispensed in 384-well cell culture plates using an Echo acoustic liquid handler. Dose response curves were dispensed in duplicates from 100 µM to 0.09 µM. BJ-hTERT and BJ-hTERT SV40RAS cells were cultured in DMEM Glutamax + 10% FBS + 50U/50 µg/mL Penicillin/Streptomycin and were seeded in the cell culture plates containing the nano-dispensed compounds to concentrations of 1200 cells/well (BJ-hTERT) and 600 cells/well (BJ-hTERT SV40RAS) using a Multidrop Combi reagent dispenser. After incubation for 72 hours at 37°C, Resazurin (59 µg/mL in PBS/ 10 µL/well) was added and the plates were incubated for a further 4-7 hours at 37 °C before performing a fluorescence readout on a Hidex Sense microplate reader.

Glycosylase Activity on 8-oxoG-Containing Substrates

2 nM of the indicated 34mer [^{32}P]5'-labeled 8oxoG-containing substrate was incubated with 0.5 nM OGG1 and either 10% DMSO, 50 µM TH5487, 6.25 µM TH10785, 6.25 µM **30** or 10 µM **38** in the presence of 30 mM HEPES, pH 7.5, 4% glycerol, 20 mM EDTA, 16 mM NaCl and 0.01% Tween-20. Samples were incubated at 37 ºC for the indicated times and stopped by adding 0.1 N NaOH. After incubating at 90 ºC for 5 min, 12.5 µL of formamide buffer was added to the samples and DNA products were resolved by 7 M urea-20% PAGE and visualized with an Amersham Typhoon scanner.

AP-Lyase Assay on Radiolabeled AP-Site-Containing Oligonucleotide

The assay was performed by incubating 1 nM of the indicated 34-mer [^{32}P]5'-labeled uridine-containing substrate, previously treated with 0.2 U of *E. coli* UDG (NEB) to obtain a natural AP site, with 75 nM of either the wildtype hOGG1 or K249A mutant OGG1, and either 10% DMSO or 6.25 µM of **30** or

41, in the presence of 30 mM HEPES, pH 7.5, 4% glycerol, 20 mM EDTA, 16 mM NaCl, 0.01% Tween-20 in a final reaction volume of 12.5 μL. Samples were incubated at 37 ºC for the indicated times. The reaction was stopped by adding of 12.5 μL of formamide buffer (20 mM EDTA, 95% formamide, 0.05% bromophenol blue and 0.05% xylencyanol blue) and further heating for 2 min at 90 ºC. Products were resolved by 7 M urea-20% PAGE and visualized with an Amersham Typhoon scanner.

S4. Chemistry Methods and Characterization

General Procedure A. Nitro Reduction

A round-bottom flask was charged with the nitrobenzene (1.00 equiv.), iron (4.00 equiv.), and EtOH/AcOH (1:1, 5 mL per mmol of nitrobenzene). The flask was equipped with a reflux-condenser, and the mixture was heated to reflux for 3.5 h. After cooling to RT, the mixture was diluted with water (7.5 mL per mmol of nitrobenzene) and basified by addition of solid Na_2CO_3 until effervescence ceased. The mixture was extracted with CH_2Cl_2 (2 × 7.5 mL per mmol of nitrobenzene). The combined organic phases were dried over anhydrous Na_2SO_4, filtered, and volatiles were removed under reduced pressure. The crude aniline was either used directly in the next step or purified by column chromatography (silica gel).

General Procedure B. Aniline Acetylation

A round-bottom flask was charged with the aniline (1.00 equiv.), CH_2Cl_2 (3.5 mL per mmol of aniline), and NEt_3 (1.10 equiv.). The mixture was cooled to 0 °C, and acetyl chloride (2.00 equiv.) was added. The resulting mixture was stirred at this temperature for 30 min, then allowed to warm to RT for 17 h. Water (5 mL per mmol of aniline) was added, and the separated aqueous phase was extracted with CH_2Cl_2 (2 × 5 mL per mmol of aniline). The combined organic phases were washed with brine (2 mL per mmol aniline), dried over anhydrous Na_2SO_4, filtered, and volatiles were removed under reduced pressure. The crude acetanilide was either used directly in the next step or purified by column chromatography (silica gel).

General Procedure C. Sonogashira Coupling

An oven-dried microwave vial was charged with CuI (9 mol%) and $PdCl_2(PPh_3)_2$ (3 mol%). The vial was sealed, evacuated, and re-filled with N_2, after which NEt_3 (2.0 mL per mmol of alkyne) (purged with N_2 for 10 minutes prior to use), aryl halide (1.50 equiv.), and alkyne (1.00 equiv.) were added to the vial. Reactions with aryl bromides were heated to 90 °C for 3 h. Reactions with aryl iodides were stirred at RT for 3 h. The vial was decapped, the crude mixture was diluted with EtOAc (10 mL per mmol of alkyne), filtered through celite, and volatiles were removed under reduced pressure. The residue was purified by column chromatography (silica gel, EtOAc in hexane).

General Procedure D. Benzylation

A round-bottom flask equipped with a reflux condenser was charged with the phenol (1.00 equiv.), K_2CO_3 (1.20 equiv.), acetone (2.5 mL per mmol of phenol), and benzyl bromide (1.10 equiv.). The mixture was heated to reflux for 3 h. The hot suspension was filtered, and the filter cake was washed with acetone (10 mL per mmol of phenol). The filtrates were collected, and volatiles were removed

under reduced pressure. The residue was purified either by column chromatography (silica gel, EtOAc in hexane) or by washing with Et_2O/hexane.

2-Iodo-4-methoxyaniline (**S3**)

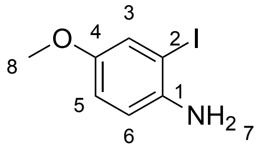

Prepared according to General Procedure A from 2-iodo-4-methoxy-1-nitrobenzene (1.12 g, 1.00 equiv., 4.00 mmol). Product was collected as a brown solid (914 mg, 92%) and was used in the next step without further purification.

^1H NMR (500 MHz, $CDCl_3$) δ 7.21 (d, *J* = 2.8 Hz, 1H), 6.77 (dd, *J* = 8.7, 2.8 Hz, 1H), 6.70 (d, *J* = 8.7 Hz, 1H), 4.30 (bs, 2H), 3.72 (s, 3H).

^{13}C NMR (126 MHz, $CDCl_3$) δ 152.9, 140.9, 123.6, 116.2, 115.6, 84.5, 56.1.

The spectral data were consistent with those previously reported in the literature.[12]

N-(2-Iodo-4-methoxyphenyl)acetamide (**S1a**)

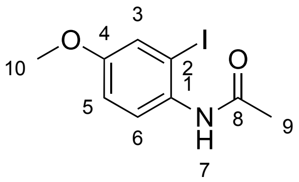

Prepared according to General Procedure B from 2-iodo-4-methoxyaniline (802 mg, 1.00 equiv., 3.22 mmol). Purified by column chromatography (5% MeOH in CH_2Cl_2) to afford the product as an off-white solid (836 mg, 89%).

^1H NMR (500 MHz, $CDCl_3$) δ 7.92 (d, *J* = 9.0 Hz, 1H), 7.31 (d, *J* = 2.8 Hz, 1H), 7.21 (s, 1H), 6.90 (dd, *J* = 9.0, 2.8 Hz, 1H), 3.77 (s, 3H), 2.21 (s, 3H).

^{13}C NMR (126 MHz, $CDCl_3$) δ 168.4, 156.9, 131.8, 123.9, 123.8, 114.9, 91.6, 55.8, 24.6.

The spectral data were consistent with those previously reported in the literature.[13]

tert-Butyl (4-(pyridin-3-yl)but-3-yn-1-yl)carbamate (**S2a**)

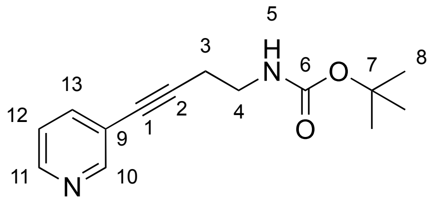

Prepared according to General Procedure C from *tert*-butyl but-3-yn-1-ylcarbamate (182 µL, 1.00 equiv., 1.00 mmol) and 3-bromopyridine (147 µL, 1.50 equiv., 1.50 mmol). Purified by column chromatography (10 to 25% EtOAc in hexane) to afford the product as a brown oil (196 mg, 80%).

^{1}H NMR (500 MHz, $CDCl_3$) δ 8.64 (s, 1H), 8.51 (d, *J* = 5.0 Hz, 1H), 7.68 (dt, *J* = 7.9, 1.9 Hz, 1H), 7.23 (dd, *J* = 7.9, 4.9 Hz, 1H), 4.86 (bs, 1H), 3.38 (q, *J* = 6.5 Hz, 2H), 2.63 (t, *J* = 6.5 Hz, 2H), 1.45 (s, 9H).

^{13}C NMR (126 MHz, $CDCl_3$) δ 155.8, 152.0, 147.9, 138.4, 123.4, 121.0, 91.0, 79.4, 78.7, 39.4, 28.4, 21.0.

υ_{max} (neat) 3325, 2974, 1694, 1516, 1250, 1167 cm^{-1}.

HRMS (ESI) *m/z* calcd. for $[M+H]^+$ ($C_{14}H_{19}N_2O_2$): 247.1441, found: 247.1436.

N-(3-(Benzyloxy)phenyl)acetamide (**S4**)

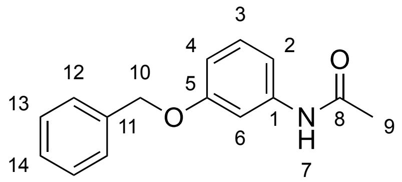

Prepared according to General Procedure D from 3-acetamidophenol (2.27 g, 1.00 equiv., 15.0 mmol). Purified by washing with Et_2O/hexane (1:1, 2 × 30 mL; then 2:1 2 × 30 mL) to afford the product as an off-white solid (3.20 g, 88%).

^{1}H NMR (400 MHz, $CDCl_3$) δ 7.46 (bs, 1H), 7.44 – 7.35 (m, 5H), 7.34 – 7.29 (m, 1H), 7.19 (t, *J* = 8.1 Hz, 1H), 6.98 (dd, *J* = 8.0, 2.0 Hz, 1H), 6.72 (dd, *J* = 8.2, 2.7 Hz, 1H), 5.04 (s, 2H), 2.15 (s, 3H).
^{13}C NMR (101 MHz, $CDCl_3$) δ 168.6, 159.4, 139.2, 137.0, 129.8, 128.7, 128.1, 127.6, 112.3, 111.1, 106.7, 70.1, 24.8.

The spectral data were consistent with those previously reported in the literature.[14]

N-(5-(Benzyloxy)-2-iodophenyl)acetamide (**S1b**)

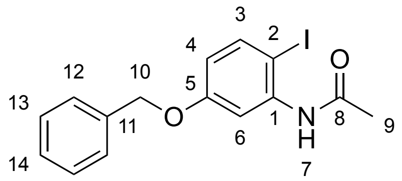

A round-bottom flask under air was charged with *N*-(3-(benzyloxy)phenyl)acetamide (1.93 g, 8.00 mmol, 1.00 equiv.), *N*-iodosuccinimide (1.89 g, 8.40 mmol, 1.05 equiv.), *p*-TsOH·H_2O (761 mg, 4.00 mmol, 0.500 equiv.), and $Pd(OAc)_2$ (89.8 mg, 0.400 mmol, 5.00 mol%). Toluene (32.0 mL) was added, and mixture was stirred at 40 °C for 2 h. After cooling to RT, the mixture was diluted with Et_2O (25 mL) and washed with aq. sat. $NaHCO_3$ solution (3 × 30 mL). The organic phase was dried over Na_2SO_4, filtered, and volatiles were removed under reduced pressure. The residue was purified by column chromatography (silica gel, 10 to 20% EtOAc in toluene) to afford the product as a white solid (2.02 g, 69%).

^{1}H NMR (400 MHz, $CDCl_3$) δ 8.12 – 8.06 (m, 1H), 7.60 (d, *J* = 8.8 Hz, 1H), 7.45 – 7.41 (m, 2H), 7.40 – 7.36 (m, 2H), 7.35 – 7.30 (m, 1H), 6.54 (dd, *J* = 8.8, 3.0 Hz, 1H), 5.06 (s, 2H), 2.24 (s, 3H).

^{13}C NMR (101 MHz, $CDCl_3$) δ 168.5, 159.8, 139.1, 138.8, 136.6, 128.7, 128.2, 127.7, 113.7, 108.1, 78.2, 70.3, 25.1.

υ_{max} (film) 3269, 2980, 1676, 1580, 1518, 1047 cm^{-1}.

HRMS (ESI) *m/z* calcd. for $[M+Na]^+$ ($C_{15}H_{14}I_1N_1Na_1O_2$): 389.9956, found: 389.9962.

<u>*tert*-Butyl (2-methoxyphenyl)carbamate (**S5**)</u>

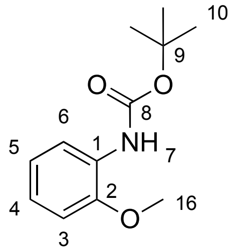

A round-bottom flask equipped with a reflux condenser was charged with Boc_2O (5.02 g, 1.15 equiv., 23.0 mmol), 2-methoxyaniline (2.46 g, 2.26 mL, 1.00 equiv., 20.0 mmol), and THF (30 mL). The mixture was heated to reflux for 17 h. Water (20 mL) was added, and the mixture was extracted with Et_2O (3 × 20 mL). The combined organic phases were dried over anhydrous Na_2SO_4, filtered, and volatiles were removed under reduced pressure. The residue was purified by column chromatography (silica gel, 10% EtOAc in hexane) to give a clear oil contaminated with Boc_2O.
The oil was dissolved in CH_2Cl_2 (10 mL), to which was added imidazole (516 mg, 7.5 mmol) and the mixture was stirred at RT for 30 min. The solution was diluted with CH_2Cl_2 (15 mL) and was washed

with aq. HCl solution (0.5 M, 3 × 15 mL). The organic phase was dried over anhydrous Na_2SO_4, filtered, and volatiles were removed under reduced pressure to afford the product as a clear oil (3.309 g, 74%), which was used in the next step without further purification.

^{1}H NMR (500 MHz, $CDCl_3$) δ 8.08 (bs, 1H), 7.10 (s, 1H), 6.95 (m, 2H), 6.85 (dd, *J* = 7.4, 2.1 Hz, 1H), 3.86 (s, 3H), 1.53 (s, 9H).

^{13}C NMR (126 MHz, $CDCl_3$) δ 152.9, 147.6, 128.2, 122.4, 121.2, 118.1, 110.0, 80.4, 55.7, 28.5.

The spectral data were consistent with those previously reported in the literature.[15]

tert-Butyl (2-iodo-6-methoxyphenyl)carbamate (**S1c**)

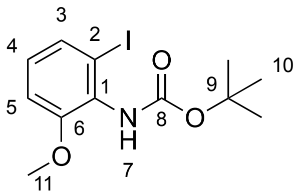

A flame-dried Schlenk flask was charged with *tert*-butyl (2-methoxyphenyl)carbamate (893 mg, 1.00 equiv., 4.00 mmol). The flask was sealed, evacuated, and re-filled with N_2 three times. Anhydrous Et_2O (10 ml) was added, and the mixture was cooled to −25 °C (liquid N_2/EtOAc). To this was added, dropwise, *tert*-butyllithium (4.94 mL, 1.7 M in pentane, 2.1 equiv., 8.40 mmol). The solution was stirred at this temperature for 3 h, after which it was cooled to −84 °C, and a solution of 1,2-diiodoethane (1.13 g, 1.00 equiv., 4.00 mmol) in dry Et_2O (4.0 mL) was added. The mixture was allowed to gradually warm to −40 °C over 1 h, before being allowed to warm to RT and stirring for further 18 h. The mixture was diluted with Et_2O (50 ml), washed with water (1 × 50 ml), and then brine (2 × 50 ml). The organic phase was dried over anhydrous Na_2SO_4, filtered, and volatiles were removed under reduced pressure. The residue was washed with hexane to afford the product as a pale-yellow powder (754 mg, 54%).

^{1}H NMR (500 MHz, $CDCl_3$) δ 7.43 (dd, *J* = 7.9, 1.4 Hz, 1H), 6.93 (t, *J* = 8.1 Hz, 1H), 6.87 (dd, *J* = 8.3, 1.4 Hz, 1H), 5.99 (bs, 1H), 3.82 (s, 3H), 1.50 (s, 9H).

^{13}C NMR (101 MHz, $CDCl_3$) δ 155.5, 153.6, 130.9, 129.1, 128.6, 111.5, 100.4, 80.6, 56.1, 28.4.

The spectral data were consistent with those previously reported in the literature.[16]

2-Amino-3-iodophenol (**S6**)

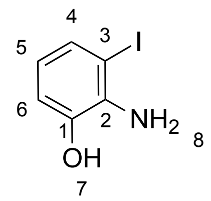

A flame-dried Schlenk flask was charged with *tert*-butyl (2-iodo-6-methoxyphenyl)carbamate (524 mg, 1.00 equiv., 1.50 mmol), sealed, and evacuated and re-filled with N_2 three times. Dry CH_2Cl_2 (3.0 mL) was added, and the mixture was cooled to −84 °C. BBr_3 (3.30 mL, 1.00 M in CH_2Cl_2, 2.2 equiv., 3.30 mmol) was added dropwise *via* syringe over 5 min. The mixture was allowed to warm to RT and was stirred for 16 h. To the mixture was added water (10 mL, added cautiously) and CH_2Cl_2 (10 mL). The pH of the aq. phase was adjusted to >7 with sat. $NaHCO_3$ solution and the mixture was extracted with CH_2Cl_2 (2 × 30 mL). The combined organic phases were washed with brine (20 mL), dried over anhydrous Na_2SO_4, filtered, and volatiles were removed under reduced pressure. The residue was purified by column chromatography (silica gel, 10 to 15% EtOAc in hexane) to afford the product as an orange solid (281 mg, 80%).

^1H NMR (500 MHz, $CDCl_3$) δ 7.25 (dd, *J* = 8.0, 1.3 Hz, 1H), 6.69 (dd, *J* = 7.9, 1.3 Hz, 1H), 6.41 (t, *J* = 8.0 Hz, 1H), 5.11 (bs, 1H), 4.12 (bs, 2H).

^{13}C NMR (126 MHz, $CDCl_3$) δ 143.0, 136.3, 131.2, 120.3, 115.0, 85.5.

The spectral data were consistent with those previously reported in the literature.[16]

N-(2-Hydroxy-6-iodophenyl)acetamide (**S7**)

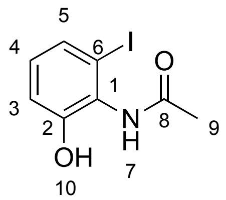

A round-bottom flask was charged with 2-amino-3-iodophenol (235 mg, 1.00 equiv., 1.00 mmol) and EtOAc (2.0 mL). The mixture was cooled to 0 °C, and acetic anhydride (100 μL, 1.06 equiv., 1.06 mmol) was added dropwise. The resulting mixture was allowed to warm to RT, stirred for 24 h and then stirred at 35 °C for 1 h. The volatiles were removed under reduced pressure and the solid residue was dried under vacuum to afford the product as a light-brown solid (264 mg, 95%), which was used in the next step without further purification.

^1H NMR (500 MHz, $CDCl_3$) δ 8.83 (s, 1H), 7.51 (bs, 1H), 7.40 (dd, *J* = 7.9, 1.4 Hz, 1H), 7.04 (dd, *J* = 8.2, 1.3 Hz, 1H), 6.88 (t, *J* = 8.0 Hz, 1H), 2.36 (s, 3H).

^{13}C NMR (126 MHz, $CDCl_3$) δ 171.2, 150.2, 131.0, 129.1, 127.0, 121.4, 93.2, 24.0.

υ_{max} (film) 3244, 2926, 1636, 1443, 1366, 1288 cm^{-1}.

HRMS (ESI) *m/z* calcd. for $[M+Na]^+$ ($C_8H_8I_1N_1O_2Na_1$): 299.9492, found: 299.9491.

N-(2-(Benzyloxy)-6-iodophenyl)acetamide (**S1d**)

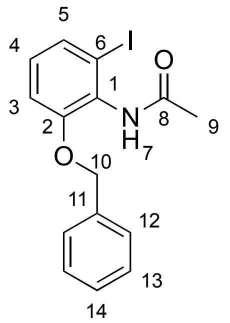

Prepared according to General Procedure D from *N*-(2-hydroxy-6-iodophenyl)acetamide (249 mg, 1.00 equiv., 0.900 mmol). Purified by column chromatography (10 to 15% EtOAc in hexane) to afford the product as a light brown solid (253 mg, 77%).

^1H NMR (500 MHz, $CDCl_3$) δ 7.49 – 7.41 (m, 1H), 7.39 – 7.28 (m, 5H), 7.09 (s, 1H), 7.01 – 6.86 (m, 2H), 5.04 (s, 2H), 2.14 (s, 3H).

^{13}C NMR (126 MHz, $CDCl_3$) δ 168.9, 154.7, 136.4, 131.2, 129.7, 128.7, 128.1, 127.3, 120.9, 113.3, 100.3, 70.8, 23.5.

υ_{max} (film) 3252, 3011, 1668, 1522, 1508, 1464 cm^{-1}.

HRMS (ESI) *m/z* calcd. for $[M+H]^+$ ($C_{15}H_{15}I_1N_1O_2$): 368.0142, found: 368.0141.

2-Iodo-3-methoxyaniline (**S8**)

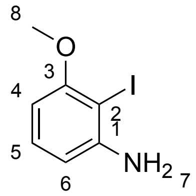

Prepared according to General Procedure A from 2-iodo-1-methoxy-3-nitrobenzene (279 mg, 1.00 equiv., 1.00 mmol). Product was collected as a dark green oil (244 mg, 98%) and was used in the next step without further purification.

^{1}H NMR (500 MHz, $CDCl_3$) δ 7.07 (t, *J* = 8.0 Hz, 1H), 6.41 (d, *J* = 8.0 Hz, 1H), 6.22 (d, *J* = 8.1 Hz, 1H), 4.22 (s, 2H), 3.85 (s, 3H).

^{13}C NMR (126 MHz, $CDCl_3$) δ 158.9, 148.5, 129.6, 107.9, 100.6, 76.0, 56.5.

The spectral data were consistent with those previously reported in the literature.[17]

<u>*N*-(2-Iodo-3-methoxyphenyl)acetamide (**S1e**)</u>

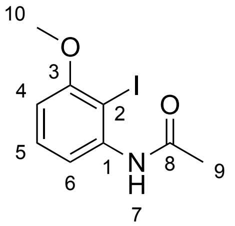

Prepared according to General Procedure B from 2-iodo-3-methoxyaniline (174 mg, 1.00 equiv., 0.700 mmol). Purified by column chromatography (20 to 30% EtOAc in hexane) to afford the product as a white solid (134 mg, 66%).

^{1}H NMR (400 MHz, $CDCl_3$) δ 7.85 (d, *J* = 8.2 Hz, 1H), 7.63 (bs, 1H), 7.26 (t, *J* = 8.2 Hz, 1H), 6.64 – 6.54 (d, *J* = 8.2 Hz, 1H), 3.86 (s, 3H), 2.22 (s, 3H).

^{13}C NMR (101 MHz, $CDCl_3$) δ 168.4, 158.3, 139.7, 129.8, 114.6, 106.8, 82.5, 56.6, 25.0.

υ_{max} (film) 3235, 3013, 1653, 1456, 1256, 789, 691 cm^{-1}.

HRMS (ESI) *m/z* calcd. for $[M+H]^+$ ($C_9H_{11}I_1N_1O_2$): 291.9829, found: 291.9828.

<u>*N*-(4-Fluoro-2-iodophenyl)acetamide (**S1f**)</u>

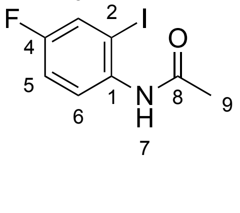

Prepared according to General Procedure B from 4-fluoro-2-iodoaniline (237 mg, 1.00 equiv., 1.00 mmol). Purified by column chromatography (10 to 25% EtOAc in hexane) to afford the product as a white solid (262 mg, 94%).

^{1}H NMR (400 MHz, $CDCl_3$) δ 8.11 (dd, J = 9.1, 5.5 Hz, 1H), 7.50 (dd, J = 7.7, 2.9 Hz, 1H), 7.09 (ddd, J = 9.1, 7.8, 2.9 Hz, 1H), 2.23 (s, 3H).

^{13}C NMR (126 MHz, $CDCl_3$) δ 168.4, 158.8 (d, $^1J_{CF}$ = 249.1 Hz), 134.9, 125.5 (d, $^2J_{CF}$ = 24.7 Hz), 123.3 (d, $^3J_{CF}$ = 7.7 Hz), 116.2 (d, $^2J_{CF}$ = 21.8 Hz), 89.9 (d, $^3J_{CF}$ = 8.3 Hz), 24.8.

^{19}F NMR (377 MHz, $CDCl_3$) δ −116.05.

The spectral data were consistent with those previously reported in the literature.[18]

N-(5-Fluoro-2-iodophenyl)acetamide (**S1g**)

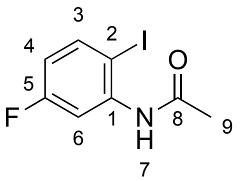

Prepared according to General Procedure B from 5-fluoro-2-iodoaniline (190 mg, 1.00 equiv., 0.800 mmol). Purified by column chromatography (5 to 15% EtOAc in hexane) to afford the product as a white solid (118 mg, 53%).

^{1}H NMR (500 MHz, $CDCl_3$) δ 8.16 (dd, J = 11.2, 3.0 Hz, 1H), 7.70 (dd, J = 8.8, 6.0 Hz, 1H), 7.47 (bs, 1H), 6.63 (ddd, J = 8.7, 7.7, 3.0 Hz, 1H), 2.25 (s, 3H).

^{13}C NMR (126 MHz, $CDCl_3$) δ 168.4, 163.4 (d, $^1J_{CF}$ = 246.6 Hz), 139.6 (d, $^3J_{CF}$ = 11.8 Hz), 139.2 (d, ^3J = 9.0 Hz), 113.1 (d, $^2J_{CF}$ = 22.5 Hz), 109.3 (d, $^2J_{CF}$ = 28.6 Hz), 81.9, 25.1.

^{19}F NMR (377 MHz, $CDCl_3$) δ −110.46.

The spectral data were consistent with those previously reported in the literature.[19]

N-(2-Iodo-4-nitrophenyl)acetamide (**S9**)

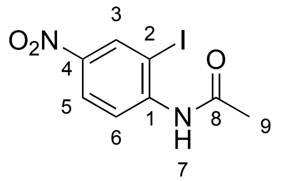

Prepared according to General Procedure B from 2-iodo-4-nitroaniline (1.32 g, 1.00 equiv., 5.00 mmol). Purified by column chromatography (10 to 20% EtOAc in hexane) to afford the product as a yellow solid (870 mg, 57%).

^{1}H NMR (400 MHz, $CDCl_3$) δ 8.61 (d, J = 2.6 Hz, 1H), 8.49 (d, J = 9.2 Hz, 1H), 8.18 (dd, J = 9.2, 2.6 Hz, 1H), 7.74 (bs, 1H), 2.29 (s, 3H).

^{13}C NMR (101 MHz, $CDCl_3$) δ 168.6, 143.9, 143.4, 134.3, 124.9, 119.8, 87.4, 25.2.

The spectral data were consistent with those previously reported in the literature.[20]

N-(4-Amino-2-iodophenyl)acetamide (**S10**)

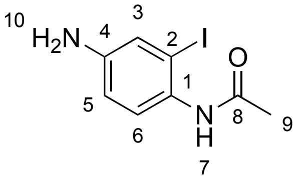

Prepared according to General Procedure A from *N*-(2-iodo-4-nitrophenyl)acetamide (306 mg, 1.00 equiv., 1.00 mmol). Purified by column chromatography (2% MeOH in CH_2Cl_2), followed by trituration with $CHCl_3$ to afford the product as a tan solid (154 mg, 56%).

^{1}H NMR (500 MHz, $(CD_3)_2CO$) δ 8.28 (s, 1H), 7.24 – 7.19 (m, 1H), 7.18 (d, J = 2.6 Hz, 1H), 6.65 (dd, J = 8.6, 2.6 Hz, 1H), 4.78 (s, 2H), 2.07 (s, 3H).

^{13}C NMR (126 MHz, $(CD_3)_2CO$) δ 168.9, 148.5, 129.8, 128.2, 124.2, 115.3, 96.9, 23.5.

υ_{max} (film) 3410, 3258, 1651, 1632, 1599, 1521, 1490 cm^{-1}.
1489.05

HRMS (ESI) *m/z* calcd. for $[M+H]^+$ ($C_8H_{10}I_1N_2O_1$): 276.9832, found: 276.9831.

tert-Butyl (4-acetamido-3-iodophenyl)carbamate (**S1h**)

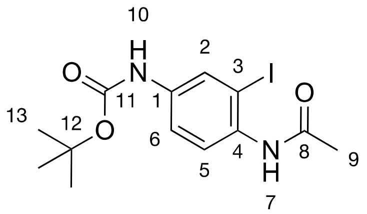

A round-bottom flask was charged with *N*-(4-amino-2-iodophenyl)acetamide (138 mg, 1.00 equiv., 0.500 mmol), and THF (1.2 mL). Boc_2O (115 mg, 1.05 equiv., 0.525 mmol) was added, the flask was equipped with a reflux condenser, and the mixture was heated to reflux for 17 h. Water (5 mL) was added, and the mixture was extracted with Et_2O (3 × 5 mL). The combined organic phases were dried over anhydrous Na_2SO_4, filtered, and volatiles were removed under reduced pressure. The residue was purified by column chromatography (silica gel, 1% MeOH in CH_2Cl_2) to afford the product as a light pink solid (176 mg, 93%).

^{1}H NMR (400 MHz, $CDCl_3$) δ 8.05 (bs, 1H), 8.01 (d, *J* = 8.8 Hz, 1H), 7.31 (bs, 1H), 7.13 (dd, *J* = 8.9, 2.5 Hz, 1H), 6.50 (bs, 1H), 2.22 (s, 3H), 1.51 (s, 9H).

^{13}C NMR (101 MHz, $CDCl_3$) δ 168.3, 152.6, 135.9, 133.7, 128.3, 122.6, 119.3, 90.8, 81.1, 28.4, 24.8.

υ_{max} (film) 3312, 1690, 1665, 1539, 1240, 1151 cm^{-1}.

HRMS (ESI) *m/z* calcd. for $[M+H]^+$ ($C_{13}H_{18}I_1N_2O_3$): 377.0357, found: 377.0363.

<u>Methyl 4-acetamido-3-iodobenzoate **(S1i)**</u>

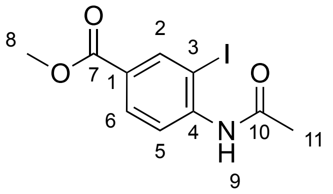

Prepared according to General Procedure B from methyl 4-amino-3-iodobenzoate (389 mg, 1.00 equiv., 1.40 mmol). Purified by column chromatography (20 to 25% EtOAc in hexane) to afford the product as a white solid (380 mg, 85%).

^{1}H NMR (500 MHz, $CDCl_3$) δ 8.43 (d, *J* = 1.9 Hz, 1H), 8.36 (d, *J* = 8.7 Hz, 1H), 7.97 (dd, *J* = 8.7, 2.0 Hz, 1H), 7.63 (s, 1H), 3.88 (s, 3H), 2.26 (s, 3H).

^{13}C NMR (126 MHz, $CDCl_3$) δ 168.5, 165.3, 142.1, 140.3, 130.9, 127.1, 120.3, 88.4, 52.4, 25.2.

The spectral data were consistent with those previously reported in the literature.[20]

N-(3-Fluoro-4-methoxyphenyl)acetamide (**S11**)

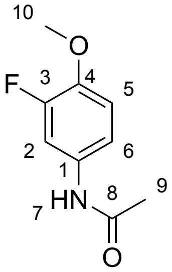

Prepared according to General Procedure B from 3-fluoro-4-methoxyaniline (706 mg, 1.00 equiv., 5.00 mmol). Purified by column chromatography (40 to 55% EtOAc in hexane) to afford the product as a white solid (894 mg, 98%).

^{1}H NMR (500 MHz, $CDCl_3$) δ 7.82 (bs, 1H), 7.39 (dd, J = 12.9, 2.4 Hz, 1H), 7.11 (ddd, J = 8.8, 2.6, 1.5 Hz, 1H), 6.90 – 6.82 (m, 1H), 3.87 – 3.83 (m, 3H), 2.16 – 2.11 (m, 3H).

^{13}C NMR (126 MHz, $CDCl_3$) δ 168.9, 152.1 (d, $^{1}J_{CF}$ = 245.0 Hz), 144.5 (d, $^{2}J_{CF}$ = 10.3 Hz), 132.2 – 130.7 (m; C^{1}), 116.0, 113., 109.5 (d, $^{2}J_{CF}$ = 22.1 Hz), 56.7, 24.4.

^{19}F NMR (470 MHz, $CDCl_3$) δ −133.34.

The spectral data were consistent with those previously reported in the literature.[21]

N-(5-Fluoro-2-iodo-4-methoxyphenyl)acetamide (**S1j**)

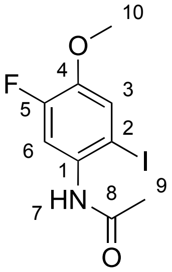

A round-bottom flask was charged with *N*-(3-fluoro-4-methoxyphenyl)acetamide (458 mg, 1.00 equiv., 2.50 mmol), *N*-iodosuccinimide (602 mg, 1.07 equiv., 2.68 mmol), *p*-TsOH·H_2O (238 mg, 0.500 equiv., 1.25 mmol), and $Pd(OAc)_2$ (28.1 mg, 5.00 mol%, 0.125 mmol). Toluene (10.0 mL) was added, and mixture was stirred at 40 °C for 3 h. After cooling to RT, the mixture was diluted with EtOAc (30 mL) and washed with sat. $NaHCO_3$ (3 × 30 mL). The organic phase was dried over anhydrous Na_2SO_4, filtered, and volatiles were removed under reduced pressure. The residue was purified by column chromatography (silica gel, 30 to 50% EtOAc in hexane) to afford the product as a white solid (403 mg, 52%).

^1H NMR (500 MHz, $CDCl_3$) δ 8.00 (d, J = 13.2 Hz, 1H), 7.29 (d, J = 8.7 Hz, 1H), 7.25 (bs, 1H), 3.85 (s, 3H), 2.21 (s, 3H).

^{13}C NMR (126 MHz, $CDCl_3$) δ 168.3, 152.4 (d, $^1J_{CF}$ = 247.1 Hz), 145.1 (d, $^2J_{CF}$ = 11.6 Hz), 132.2 (d, $^3J_{CF}$ = 9.8 Hz), 122.8 (d, $^3J_{CF}$ = 2.6 Hz), 111.0 (d, $^2J_{CF}$ = 24.4 Hz), 82.3 (d, $^4J_{CF}$ = 3.8 Hz), 56.9, 24.8.

^{19}F NMR (376 MHz, $CDCl_3$) δ −131.18.

υ_{max} (solid) 3244, 2995, 1651, 1531, 1207, 1020 cm^{-1}.

HRMS (ESI) *m/z* calcd. for $[M+H]^+$ ($C_9H_{10}F_1I_1N_1O_2$): 309.9735, found: 309.9740.

N-Hydroxy-*N*-(2-iodophenyl)acetamide (**S12**)

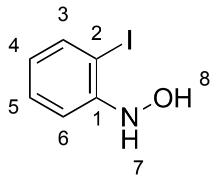

A round-bottom flask was charged with 1-iodo-2-nitrobenzene (2.49 g, 1.00 equiv., 10.0 mmol). The flask was sealed, evacuated, and re-filled with N_2. THF (30 mL) was added, and the mixture was cooled to 0 °C, after which 5 wt% Rh/C (61.7 mg, 0.30 mol%), and, dropwise, hydrazine hydrate (584 µL, 1.20 equiv., 12.0 mmol) were added. The flask was placed behind a blast shield and was stirred at 0 °C for 2.5 h. The mixture was filtered through a short pad of celite, and volatiles were removed under reduced pressure to afford the crude hydroxylamine which was used in the next step without further purification.

^1H NMR (500 MHz, $CDCl_3$) δ 7.68 (dd, J = 7.8, 1.3 Hz, 1H), 7.33 (t, J = 7.7 Hz, 1H), 7.24 (dd, J = 8.1, 1.6 Hz, 1H), 6.87 (s, 1H), 6.72 (td, J = 7.5, 1.6 Hz, 1H).

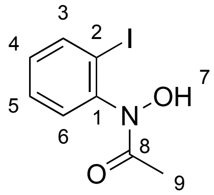

A flame-dried round-bottom flask was charged with crude *N*-(2-iodophenyl)hydroxylamine (2.35 g, 1.00 equiv., 10.0 mmol), and $NaHCO_3$ (1.01 g, 1.20 equiv., 12.0 mmol). The flask was sealed, evacuated, and re-filled with N_2. Anhydrous Et_2O (25 mL) was added, the mixture was cooled to 0 °C, and acetyl chloride was added (853 µL, 1.20 equiv., 12.0 mmol). The resulting mixture was allowed to warm to RT and was stirred for 7 h. Solids were filtered off, washed with Et_2O, and the combined filtrates were concentrated under reduced pressure. The residue was purified by column

chromatography (silica gel, 20 to 40% EtOAc in hexane) to afford the product as a light yellow solid (1.54 g, 56% over two steps).

^{1}H NMR (500 MHz, $CDCl_3$) δ 8.95 (bs, 1H), 7.95 (d, J = 8.0 Hz, 1H), 7.46 (d, J = 4.3 Hz, 2H), 7.17 (dt, J = 8.8, 4.6 Hz, 1H), 1.94 (s, 3H).

^{13}C NMR (126 MHz, $CDCl_3$) δ 166.1, 141.4, 140.4, 131.8, 130.5, 129.8, 99.6, 20.3.

$υ_{max}$ (film) 3161, 2901, 1643, 1468, 1435, 1398 cm^{-1}.

HRMS (ESI) *m/z* calcd. for $[M+H]^+$ ($C_8H_9I_1N_1O_2$): 277.9673, found: 277.9671.

N-(4-Hydroxy-2-iodophenyl)acetamide (**S13**)

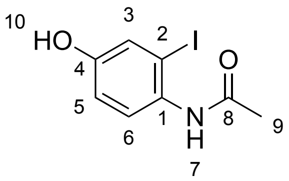

A round-bottom flask under air was charged with *N*-hydroxy-*N*-(2-iodophenyl)acetamide (471 mg, 1.00 equiv., 1.70 mmol), and anhydrous 1,4-dioxane (6.0 mL, dried by stirring over CaH_2 for 24 h followed by distillation). To this was added phenylselenyl bromide (40.1 mg, 10 mol%, 0.170 mmol), and the mixture was stirred at RT for 3 h. Volatiles were removed under reduced pressure and the residue was purified by column chromatography (silica gel, 20 to 50% EtOAc in hexane) to afford the product as a light brown solid (311 mg, 66%).

^{1}H NMR (400 MHz, $(CD_3)_2CO$) δ 8.68 (s, 1H), 8.37 (s, 1H), 7.42 (d, J = 8.7 Hz, 1H), 7.33 (d, J = 2.7 Hz, 1H), 6.85 (dd, J = 8.7, 2.8 Hz, 1H), 2.10 (s, 3H).

^{13}C NMR (101 MHz, $(CD_3)_2CO$) δ 169.0, 156.5, 132.8, 128.0, 125.9, 116.5, 95.7, 23.6.

The spectral data were consistent with those previously reported in the literature.[22]

N-(2-Iodo-4-(methoxymethoxy)phenyl)acetamide (**S1k**)

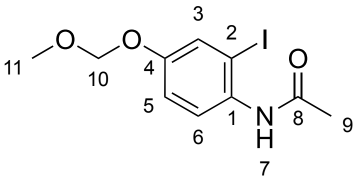

A flame-dried round-bottom flask was charged with *N*-(4-hydroxy-2-iodophenyl)acetamide (90.4 mg, 1.00 equiv., 0.326 mmol). The flask was sealed, evacuated, and re-filled with N_2 three times. Anhydrous THF (1.5 mL), and DBU (73.8 μL, 1.50 equiv., 0.489 mmol) were added, and the mixture was stirred for 2 min at RT. Bromo(methoxy)methane (40.0 μL, 1.50 equiv., 0.489 mmol) was added and the

mixture was stirred at RT for 18 h. The reaction was quenched by addition of water (5 mL), and the mixture was extracted with Et_2O (3 × 10 mL). The combined organic phases were dried over anhydrous Na_2SO_4, filtered, and volatiles were removed under reduced pressure. The residue was purified by column chromatography (silica gel, 30 to 40% EtOAc in hexane) to afford the product as a white solid (53.2 mg, 51%).

^1H NMR (500 MHz, $CDCl_3$) δ 7.95 (d, *J* = 9.0 Hz, 1H), 7.47 (d, *J* = 2.8 Hz, 1H), 7.24 (bs, 1H), 7.04 (dd, *J* = 9.0, 2.7 Hz, 1H), 5.12 (s, 2H), 3.46 (s, 3H), 2.21 (s, 3H).

^{13}C NMR (126 MHz, $CDCl_3$) δ 168.4, 154.5, 132.8, 126.3, 123.6, 117.3, 94.8, 91.2, 56.2, 24.7.

υ_{max} (film) 3260, 2924, 1653, 1526, 1155, 1001 cm^{-1}.

HRMS (ESI) *m/z* calcd. for $[M+H]^+$ ($C_{10}H_{13}I_1N_1O_3$): 321.9935, found: 321.9946.

tert-Butyl but-3-yn-1-yl(methyl)carbamate (**S14**)

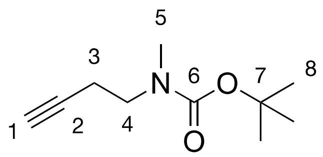

To a flame-dried round-bottom flask under N_2 was added *tert*-butyl but-3-yn-1-ylcarbamate (364 µL, 1.00 equiv., 2.00 mmol) and anhydrous THF (7.0 mL). The mixture was cooled to 0 °C and NaH (120 mg, 60% wt. dispersion in oil, 1.50 equiv., 3.00 mmol) was added. The reaction mixture was stirred at 0 °C for 0.5 h, then MeI (188 µL, 1.50 equiv., 3.00 mmol) was added. The reaction mixture was allowed to warm to RT and stirred for 20 h. The reaction mixture was quenched with sat. aq. NH_4Cl (10 mL) and extracted with EtOAc (2 × 20 mL). The combined organic phases were washed with brine (10 mL), dried over anhydrous Na_2SO_4, and filtered. Volatiles were removed under reduced pressure and The residue was purified by column chromatography (silica gel, 10% EtOAc in hexane) to afford the product as a colorless oil (285 mg, 78%).

^1H NMR (400 MHz, $CDCl_3$, mixture of rotamers) δ 3.36 – 3.27 (m, 2H), 2.87 (s, 3H), 2.35 (t, *J* = 7.3 Hz, 2H), 1.93 (t, *J* = 2.7 Hz, 1H), 1.41 (s, 9H).

^{13}C NMR (101 MHz, $CDCl_3$, mixture of rotamers) δ 155.5, 82.0 (and 81.7, C^2*), 79.6, 69.7 (and 69.6, C^1*), 48.0 (and 47.8, C^4*), 35.2 (and 34.8, C^5*), 28.5 (C^8), 18.1 (and 17.9, C^3*). *rotamer signal splitting.

The spectral data were consistent with those previously reported in the literature.[23]

tert-Butyl methyl(4-(pyridin-3-yl)but-3-yn-1-yl)carbamate (**S2b**)

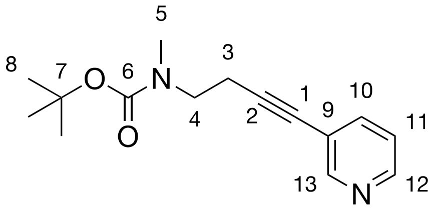

Prepared according to General Procedure D from *tert*-butyl but-3-yn-1-yl(methyl)carbamate (183 mg, 1.00 equiv., 1.00 mmol) and 3-bromopyridine (147 µL, 1.50 equiv., 1.50 mmol). Purified by column chromatography (10 to 25% EtOAc in hexane) to afford the product as an orange oil (150 mg, 58%).

^{1}H NMR (500 MHz, $CDCl_3$, mixture of rotamers) δ 8.56 (s, 1H), 8.43 (d, J = 5.0 Hz, 1H), 7.61 (dt, J = 8.0, 2.0 Hz, 1H), 7.15 (dd, J = 7.8, 4.9 Hz, 1H), 3.41 (m, 2H), 2.90 (s, 3H), 2.59 (t, J = 6.9 Hz, 2H), 1.40 (s, 9H).

^{13}C NMR (126 MHz, $CDCl_3$, mixture of rotamers) δ 155.6 (and 155.5, C^{6}*), 152.3, 148.2, 138.5, 123.0, 120.7, 91.3 (and 90.9, C^{2*}), 79.7, 78.5 (and 78.4, C^{1}*), 48.0 (and 47.7, C^{4}*), 35.1 (and 34.8 C^{5}*), 28.4, 19.2 (and 18.8 C^{3}*). *rotamer signal splitting.

υ_{max} (film) 2974, 1688, 1391, 1364, 1169, 1142 cm^{-1}.

HRMS (ESI) exact mass calculated for $[M+H]^+$ ($C_{15}H_{21}N_2O_2$): 261.1598, found: 261.1607.

tert-Butyl (3-(pyridin-3-yl)prop-2-yn-1-yl)carbamate (**S2c**)

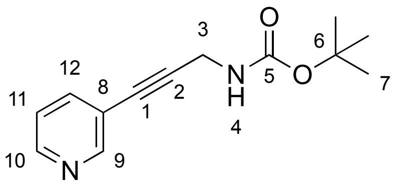

Prepared according to General Procedure B from *tert*-butyl prop-2-yn-1-ylcarbamate (310 mg, 1.00 equiv., 2.00 mmol), and 3-bromopyridine (293 µL, 1.50 equiv., 3.00 mmol). Purified by column chromatography (10 to 30% EtOAc in hexane) to afford the product as an orange oil (234 mg, 50%).

^{1}H NMR (400 MHz, $CDCl_3$) δ 8.66 (bs, 1H), 8.53 (bs, 1H), 7.70 (d, J = 8.1 Hz, 1H), 7.26 – 7.22 (m, 1H), 4.88 (s, 1H), 4.17 (d, J = 5.6 Hz, 2H), 1.46 (s, 9H).

^{13}C NMR (126 MHz, $CDCl_3$) δ 155.5, 152.2, 148.5, 138.9, 123.2, 120.1, 89.4, 80.2, 79.7, 31.2, 28.4.

The spectral data were consistent with those previously reported in the literature.[24]

tert-Butyl pent-4-yn-1-ylcarbamate (**S15**)

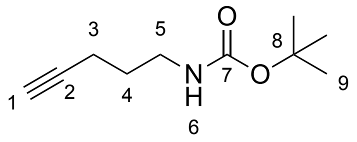

A flame-dried round-bottom flask was charged with *t*-BuOH (4.0 mL), hex-5-ynoic acid (435 µL, 1.00 equiv., 4.00 mmol), diphenyl phosphorazidate (1.02 mL, 1.18 equiv., 4.72 mmol), and NEt_3 (1.12 mL, 2.00 equiv., 8.00 mmol). The flask was equipped with a reflux condenser, and the mixture was heated to 90 °C for 48 h. After cooling to RT, the mixture was diluted with Et_2O (30 mL), filtered through celite, and volatiles were removed under reduced pressure. The residue was purified by column chromatography (silica gel, 5 to 10% EtOAc in hexane) to afford the product as a yellow oil (501 mg, 68%).

^{1}H NMR (400 MHz, $CDCl_3$) δ 4.75 (bs, 1H), 3.21 – 3.13 (m, 2H), 2.19 (td, *J* = 7.1, 2.7 Hz, 2H), 1.93 (t, *J* = 2.7 Hz, 1H), 1.67 (p, *J* = 7.0 Hz, 2H), 1.39 (s, 9H).

^{13}C NMR (101 MHz, $CDCl_3$) δ 156.0, 83.5, 79.2, 69.0, 39.6, 28.7, 28.4, 15.9.

The spectral data were consistent with those previously reported in the literature.[25]

tert-Butyl (5-(pyridin-3-yl)pent-4-yn-1-yl)carbamate (**S2d**)

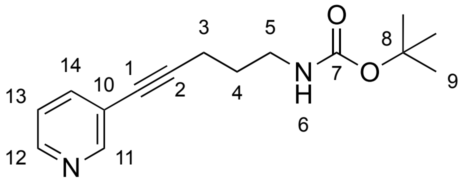

Prepared according to General Procedure C from *tert*-butyl pent-4-yn-1-ylcarbamate (190 µL, 1.00 equiv., 1.00 mmol) and 3-bromopyridine (147 µL, 1.50 equiv., 1.50 mmol). Purified by column chromatography (10 to 30% EtOAc in hexane) to afford the product as a yellow oil (178 mg, 68%).

^{1}H NMR (500 MHz, $CDCl_3$) δ 8.61 (s, 1H), 8.48 (s, 1H), 7.65 (dt, *J* = 7.9, 1.8 Hz, 1H), 7.19 (dd, *J* = 7.9, 4.8 Hz, 1H), 4.82 (s, 1H), 3.26 (q, *J* = 6.6 Hz, 2H), 2.46 (t, *J* = 7.0 Hz, 2H), 1.78 (p, *J* = 6.9 Hz, 2H), 1.42 (s, 9H).

^{13}C NMR (126 MHz, $CDCl_3$) δ 156.1, 152.4, 148.1, 138.6, 123.1, 121.0, 92.9, 79.3, 78.1, 39.9, 28.8, 28.5, 17.1.

υ_{max} (film) 3325, 2974, 2230, 1686, 1165, 706 cm^{-1}.

HRMS (ESI) *m/z* calcd. for $[M+H]^+$ ($C_{15}H_{21}N_2O_2$): 261.1598, found: 261.1595.

3-(4-((*tert*-Butyldimethylsilyl)oxy)but-1-yn-1-yl)pyridine (**S2e**)

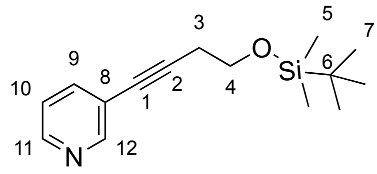

Prepared according to General Procedure C from (but-3-yn-1-yloxy)(*tert*-butyl)dimethylsilane (369 mg, 1.00 equiv., 2.00 mmol) and 3-bromopyridine (293 µL, 1.50 equiv., 3.00 mmol). Purified by column chromatography (5 to 7.5% EtOAc in hexane) to afford the product as an orange oil (318 mg, 61%).

^{1}H NMR (500 MHz, $CDCl_3$) δ 8.64 – 8.61 (m, 1H), 8.49 (dd, *J* = 4.9, 1.7 Hz, 1H), 7.66 (dt, *J* = 7.9, 1.9 Hz, 1H), 7.20 (ddd, *J* = 7.9, 4.9, 0.9 Hz, 1H), 3.82 (t, *J* = 6.9 Hz, 2H), 2.64 (t, *J* = 6.9 Hz, 2H), 0.91 (s, 9H), 0.09 (s, 6H).

^{13}C NMR (126 MHz, $CDCl_3$) δ 152.5, 148.2, 138.6, 123.1, 121.1, 91.2, 78.4, 61.8, 26.0, 24.0, 18.5, −5.1.

υ_{max} (film) 2928, 1252, 1101, 835, 775 cm^{-1}.

HRMS (ESI) *m/z* calcd. for $[M+H]^+$ ($C_{15}H_{24}N_1O_1Si_1$): 262.1622, found: 262.1627.

tert-Butyl (4-(5-fluoropyridin-3-yl)but-3-yn-1-yl)carbamate (**S2f**)

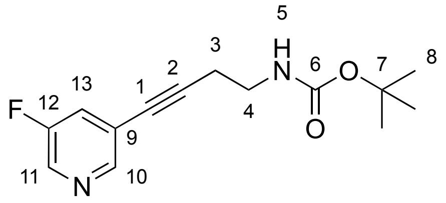

Prepared according to General Procedure C from *tert*-butyl but-3-yn-1-ylcarbamate (109 µL, 1.00 equiv., 0.600 mmol), and 3-bromo-5-fluoropyridine (158 mg, 1.50 equiv., 0.900 mmol). Purified by column chromatography (10 to 25% EtOAc in hexane) to afford the product as a yellow oil (120 mg, 76%).

^{1}H NMR (500 MHz, $CDCl_3$) δ 8.44 – 8.39 (m, 1H), 8.36 (d, J = 2.8 Hz, 1H), 7.37 (ddd, J = 9.0, 2.8, 1.6 Hz, 1H), 4.95 (bs, 1H), 3.36 (q, J = 6.5 Hz, 2H), 2.61 (t, J = 6.6 Hz, 2H), 1.43 (s, 9H).

^{13}C NMR (126 MHz, $CDCl_3$) δ 158.8 (d, $^{1}J_{CF}$ = 257.3 Hz), 155.8, 148.4 (d, $^{4}J_{CF}$ = 4.1 Hz), 137.1 (d, $^{2}J_{CF}$ = 23.2 Hz), 125.3 (d, $^{2}J_{CF}$ = 19.2 Hz), 121.8 (d, $^{3}J_{CF}$ = 5.2 Hz), 92.3, 79.7, 77.5, 39.4, 28.5, 21.2.

^{19}F NMR (377 MHz, $CDCl_3$) δ −126.94.

υ_{max} (film) 3335, 2976, 2237, 1694, 1414, 1160 cm^{-1}.

HRMS (ESI) *m/z* calcd. for $[M+H]^+$ ($C_{14}H_{18}F_1N_2O_2$): 265.1347, found: 265.1342.

tert-Butyl (4-(pyrimidin-5-yl)but-3-yn-1-yl)carbamate (**S2g**)

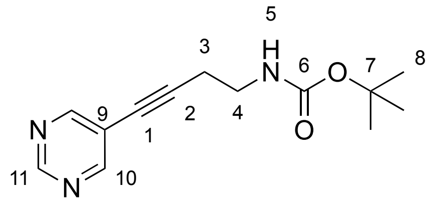

Prepared according to General Procedure C from *tert*-butyl but-3-yn-1-ylcarbamate (91 μL, 1.0 equiv., 0.50 mmol) and 5-bromopyrimidine (119 mg, 1.50 equiv., 0.750 mmol). Purified by column chromatography (15 to 35% EtOAc in hexane) to afford the product as a yellow oil (113 mg, 91%).

^{1}H NMR (400 MHz, $CDCl_3$) δ 9.08 (s, 1H), 8.71 (s, 2H), 4.99 (s, 1H), 3.36 (q, J = 6.5 Hz, 2H), 2.64 (t, J = 6.6 Hz, 2H), 1.42 (s, 9H).

^{13}C NMR (101 MHz, $CDCl_3$) δ 158.9, 156.5, 155.8, 120.2, 95.1, 79.7, 75.3, 39.3, 28.5, 21.3.

υ_{max} (film) 3319, 2976, 1694, 1524, 1412, 1165 cm^{-1}.

HRMS (ESI) *m/z* calcd. for $[M+Na]^+$ ($C_{13}H_{17}N_3O_2Na_1$): 270.1213, found: 270.1204.

tert-Butyl (4-(pyridin-2-yl)but-3-yn-1-yl)carbamate (**S2h**)

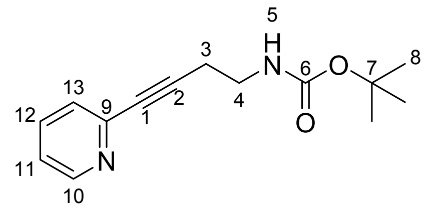

Prepared according to General Procedure C from *tert*-butyl but-3-yn-1-ylcarbamate (91 μL, 1.0 equiv., 0.50 mmol), and 2-bromopyridine (73 μL, 1.5 equiv., 0.75 mmol). Purified by column chromatography (10 to 25% EtOAc in hexane) to afford the product as an orange solid (119 mg, 97%).

^{1}H NMR (400 MHz, $CDCl_3$) δ 8.52 (d, *J* = 5.3 Hz, 1H), 7.60 (td, *J* = 7.7, 1.7 Hz, 1H), 7.36 (d, *J* = 7.9 Hz, 1H), 7.22 – 7.14 (m, 1H), 5.08 (s, 1H), 3.36 (q, *J* = 6.4 Hz, 2H), 2.62 (t, *J* = 6.5 Hz, 2H), 1.42 (s, 9H).

^{13}C NMR (101 MHz, $CDCl_3$) δ 155.9, 149.9, 143.5, 136.2, 127.1, 122.7, 88.0, 81.6, 79.5, 39.3, 28.5, 21.0.

υ_{max} (film) 3335, 2976, 1692, 1250, 1167, 777 cm^{-1}.

HRMS (ESI) *m/z* calcd. for $[M+H]^+$ ($C_{14}H_{19}N_2O_2$): 247.1441, found: 247.1440.

<u>*tert*-Butyl (4-(pyridin-4-yl)but-3-yn-1-yl)carbamate (**S2i**)</u>

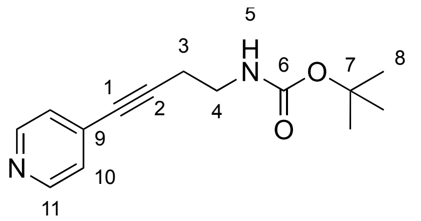

Prepared according to General Procedure C from *tert*-butyl but-3-yn-1-ylcarbamate (91 μL, 1.0 equiv., 0.50 mmol) and 4-iodopyridine (154 mg, 1.50 equiv., 0.750 mmol). Purified by column chromatography (10 to 30% EtOAc in hexane) to afford product as light brown solid (94 mg, 76%).
Note: Rotary evaporator bath was kept at 20 °C to avoid decomposition.

^{1}H NMR (400 MHz, $CDCl_3$) δ 9.23 (bs, 2H), 7.56 (bs, 2H), 4.88 (s, 1H), 3.37 (q, *J* = 6.5 Hz, 2H), 2.60 (t, *J* = 6.5 Hz, 2H), 1.45 (s, 9H).

^{13}C NMR (101 MHz, $CDCl_3$) δ 155.8, 148.4, 131.2, 131.1, 92.7, 80.1, 79.8, 39.3, 28.5, 21.3.

υ_{max} (film) 3348, 2976, 2228, 1697, 1595, 1169 cm^{-1}.

HRMS (ESI) *m/z* calcd. for $[M+H]^+$ ($C_{14}H_{19}N_2O_2$): 247.1441, found: 247.1442.

<u>*tert*-Butyl (4-(thiazol-5-yl)but-3-yn-1-yl)carbamate (**S2j**)</u>

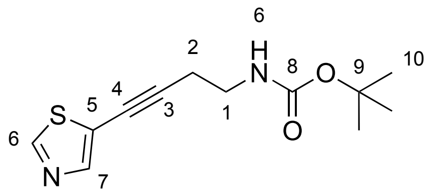

Prepared according to General Procedure C from *tert*-butyl but-3-yn-1-ylcarbamate (182 µL, 1.00 equiv., 1.00 mmol), and 5-bromothiazole (134 µL, 1.50 equiv., 1.50 mmol) at 90 °C for 3 h. Purified by column chromatography (10 to 25% EtOAc in hexane) to afford the product as a yellow oil (182 mg, 72%).

^{1}H NMR (400 MHz, $CDCl_3$) δ 8.64 (d, *J* = 0.7 Hz, 1H), 7.88 (s, 1H), 4.94 (bs, 1H), 3.34 (q, *J* = 6.4 Hz, 2H; H^1), 2.62 (t, *J* = 6.5 Hz, 2H), 1.43 (s, 9H).

^{13}C NMR (101 MHz, $CDCl_3$) δ 155.8, 152.9, 146.8, 119.4, 95.3, 79.7, 71.3, 39.3, 28.5, 21.4.

υ_{max} (neat) 3335, 2976, 1686, 1506, 1248, 1163 cm^{-1}.

HRMS (ESI) *m/z* calcd. for $[M+Na]^+$ ($C_{12}H_{16}N_2Na_1O_2S_1$): 275.0825, found: 275.0828.

N-(4-(Benzyloxy)phenyl)acetamide (**S16**)

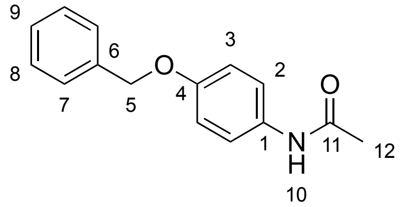

Prepared according to General Procedure B from 4-acetamidophenol (6.00 g, 1.00 equiv., 39.7 mmol). Purified by washing with Et_2O/hexane (1:1, 2 × 30 mL; then 2:1, 2 × 30 mL) to afford the product as a white solid (7.95 g, 83%).

^{1}H NMR (400 MHz, $CDCl_3$) δ 7.44 – 7.29 (m, 8H), 6.92 (d, *J* = 9.1 Hz, 2H), 5.03 (s, 2H), 2.14 (s, 3H).

^{13}C NMR (101 MHz, $CDCl_3$) δ 168.4, 155.7, 137.0, 131.3, 128.7, 128.1, 127.6, 122.0, 115.3, 70.4, 24.5.

The spectral data were consistent with those previously reported in the literature.[26]

N-(4-(Benzyloxy)-2-iodophenyl)acetamide (**S1l**)

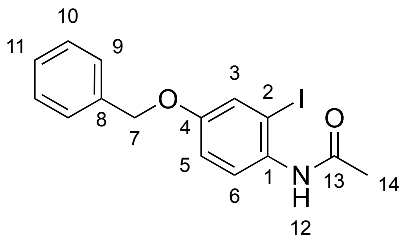

A round-bottom flask was charged with *N*-(4-(benzyloxy)phenyl)acetamide (1.45 g, 1.00 equiv., 6.00 mmol), *N*-iodosuccinimide (1.41 g, 1.05 equiv., 6.27 mmol), *p*-TsOH·H_2O (570 mg, 50 mol%, 3.00 mmol), and $Pd(OAc)_2$ (67.3 mg, 5.00 mol%, 0.30 mmol). Toluene (24 mL) was added, the flask was stoppered, and the mixture was stirred at 40 °C for 2 h. After cooling to RT, the mixture was diluted with Et_2O (25 mL) and washed with aq. sat. $NaHCO_3$ solution (20 mL) and H_2O (2 × 20 mL). The organic phase was dried over anhydrous Na_2SO_4, filtered, and volatiles were removed under reduced pressure. The residue was purified by column chromatography (10 to 20% EtOAc in toluene) to afford the product as a light pink solid (812 mg, 37%).

^{1}H NMR (400 MHz, $CDCl_3$) δ 7.94 (d, *J* = 9.0 Hz, 1H), 7.42 – 7.30 (m, 6H), 7.22 (s, 1H), 6.97 (dd, *J* = 9.0, 2.8 Hz, 1H), 5.01 (s, 2H), 2.21 (s, 3H).

^{13}C NMR (101 MHz, $CDCl_3$) δ 168.3, 156.1, 136.5, 132.2, 128.8, 128.3, 127.6, 125.0, 123.7, 115.7, 91.5, 70.6, 24.6.

υ_{max} (film) 3264, 1651, 1522, 1007, 746, 694 cm^{-1}.

HRMS (ESI) *m/z* calcd. for $[M+H]^+$ ($C_{15}H_{15}I_1N_1O_2$): 368.0142, found: 368.0134.

<u>*tert*-Butyl (2-(5-(benzyloxy)-2-(pyridin-3-yl)-1*H*-indol-3-yl)ethyl)carbamate (**S17**)</u>

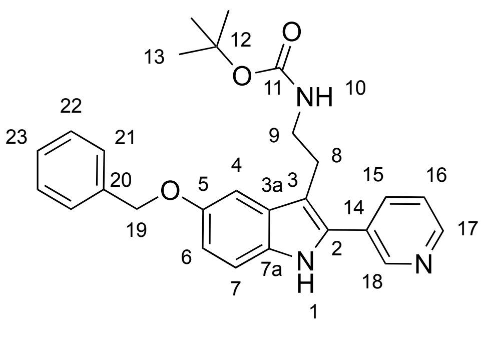

An oven-dried microwave vial was charged with *N*-(4-(benzyloxy)-2-iodophenyl)acetamide (73.4 mg, 1.33 equiv., 0.200 mmol), *tert*-butyl (4-(pyridin-3-yl)but-3-yn-1-yl)carbamate (36.9 mg, 1.00 equiv., 0.150 mmol), $Pd(OAc)_2$ (0.4 mg, 1 mol%, 1.5 µmol), 1-(4-methoxyphenyl)urea (1.0 mg, 4.0 mol%, 6.0 µmol) and K_2CO_3 (62.2 mg, 3.00 equiv., 0.450 mmol). The vial was sealed, evacuated and re-filled with N_2 three times prior to the addition of anhydrous DMF (400 µL). The solution was heated for 20 h at 130 °C. After cooling to RT, the mixture was diluted with EtOAc (10 mL) and was washed with 10%

LiCl solution (2 × 10 mL). The organic phase was dried over anhydrous Na_2SO_4, filtered, and volatiles were removed under reduced pressure. The residue was purified by column chromatography (10 to 40% EtOAc in hexane) to afford the product as an orange solid (30.5 mg, 46%).

^{1}H NMR (500 MHz, $CDCl_3$) δ 8.80 (s, 1H), 8.54 (s, 2H), 7.92 (d, J = 6.0 Hz, 1H), 7.50 (d, J = 7.5 Hz, 2H), 7.40 (dd, J = 8.3, 6.7 Hz, 3H), 7.35 – 7.29 (m, 2H), 7.19 (bs, 1H), 6.98 (dd, J = 8.8, 2.5 Hz, 1H), 5.13 (s, 2H), 4.72 – 4.67 (m, 1H), 3.43 (m, 2H), 3.02 (t, J = 7.1 Hz, 2H), 1.40 (s, 9H).

^{13}C NMR (101 MHz, $CDCl_3$) δ 156.0, 153.7, 148.5, 148.3, 137.6, 135.7, 132.7, 131.9, 129.4, 128.7 (2C), 128.0, 127.8, 123.9, 114.1, 112.1, 111.6, 102.9, 79.3, 71.1, 41.1, 28.5, 25.5.

υ_{max} (film) 3302, 2974, 1690, 1454, 1389, 1163 cm^{-1}.

HRMS (ESI) *m/z* calcd. for $[M+H]^+$ ($C_{27}H_{30}N_3O_3$): 444.2282, found: 444.2278.

2-(5-(Benzyloxy)-2-(pyridin-3-yl)-1*H*-indol-3-yl)ethan-1-amine (**S18**)

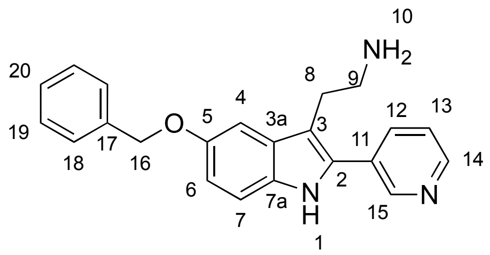

A round-bottom flask was charged with *tert*-butyl (2-(5-(benzyloxy)-2-(pyridin-3-yl)-1*H*-indol-3-yl)ethyl)carbamate (25.0 mg, 1.00 equiv., 56.4 μmol), and CH_2Cl_2 (0.40 mL). The mixture was cooled to 0 °C, TFA (0.10 mL) was added, and the mixture was stirred at 0 °C for 1 h. The mixture was allowed to warm to RT and was stirred for a further 1 h. Volatiles were removed under reduced pressure to afford a residue that was dissolved in CH_2Cl_2 (5 mL) and washed with NH_4OH (37 % aq. solution, 5 mL). The aqueous phase was further extracted with CH_2Cl_2 (2 × 10 mL). The combined organic phases were dried over anhydrous Na_2SO_4, filtered, and volatiles were removed under reduced pressure. The residue was re-dissolved in CH_2Cl_2 (5 mL) and washed with 2M NaOH (5 mL). The aqueous phase was further extracted with CH_2Cl_2 (2 × 10 mL). The combined organic phases were dried over anhydrous Na_2SO_4, filtered, and volatiles were removed under reduced pressure to afford the product as a dark green solid (21.3 mg, quant.).

^{1}H NMR (500 MHz, $CDCl_3$) δ 8.82 (d, J = 2.3 Hz, 1H), 8.57 (dd, J = 4.9, 1.6 Hz, 1H), 7.90 (dt, J = 7.9, 2.0 Hz, 1H), 7.49 (d, J = 7.4 Hz, 2H), 7.42 – 7.30 (m, 5H), 7.28 (d, J = 8.7 Hz, 1H), 7.17 (d, J = 2.4 Hz, 1H), 6.98 (dd, J = 8.8, 2.5 Hz, 1H), 5.12 (s, 2H), 3.02 – 2.96 (m, 4H).

^{13}C NMR (126 MHz, $CDCl_3$) δ 153.5, 148.9, 148.6, 137.6, 135.4, 132.5, 131.8, 129.5, 129.5, 128.7, 128.0, 127.7, 123.8, 113.9, 112.1, 111.9, 103.0, 71.1, 42.7, 28.8.

υ_{max} (film) 2920, 1452, 1200, 1186, 1022, 800 cm^{-1}.

HRMS (ESI) *m/z* calcd. for $[M+H]^+$ ($C_{22}H_{22}N_3O_1$): 344.1757, found: 344.1759.

2-(5-(Benzyloxy)-1-methyl-2-(pyridin-3-yl)-1*H*-indol-3-yl)ethan-1-amine (**S19**)

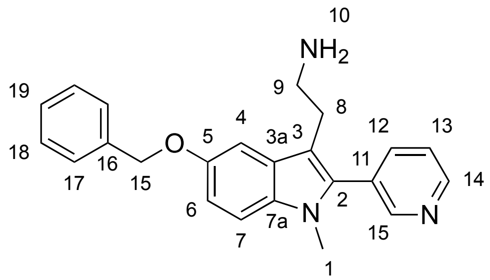

To a flame-dried round-bottom flask was added 2-(5-(benzyloxy)-2-(pyridin-3-yl)-1*H*-indol-3-yl)ethan-1-amine (15 mg, 1.0 equiv., 44 μmol), and NaH (2.9 mg, 60 wt% dispersion in mineral oil, 1.7 equiv., 72 μmol). The flask was evacuated and re-filled N_2 three times. Anhydrous DMF (0.60 mL) was added, and the mixture was cooled to 0 °C. The mixture was stirred at this temperature for 20 min, after which iodomethane (3.0 μL, 1.1 equiv., 48 μmol) was added in one portion. After stirring at 0 °C for 1 h, the mixture was then allowed to gradually warm to RT over 3 h. The reaction mixture was quenched with 2M NaOH (1 mL), diluted with CH_2Cl_2 (10 mL), and washed with aq. 10% LiCl (4 × 10 mL). The organic phase was dried over anhydrous Na_2SO_4, filtered, and volatiles were removed under reduced pressure. The residue was purified by column chromatography (silica gel, 3 to 5% MeOH in CH_2Cl_2 + 0.5 to 1% NH_4OH 37% aq. solution) to afford the product as a light brown solid (6.5 mg, 42%).

^1H NMR (500 MHz, $CDCl_3$) δ 8.69 – 8.66 (m, 2H), 7.74 (dt, *J* = 7.8, 2.1 Hz, 1H), 7.50 (d, *J* = 7.5 Hz, 2H), 7.43 (dd, *J* = 7.8, 4.9 Hz, 1H), 7.39 (t, *J* = 7.6 Hz, 2H), 7.35 – 7.30 (m, 1H), 7.26 (d, *J* = 8.8 Hz, 1H), 7.19 (d, *J* = 2.4 Hz, 1H), 7.04 (dd, *J* = 8.8, 2.4 Hz, 1H), 5.15 (s, 2H), 3.56 (s, 3H), 2.93 – 2.86 (m, 2H), 2.81 (t, *J* = 7.0 Hz, 2H), 1.67 (bs, 2H).

^{13}C NMR (126 MHz, $CDCl_3$) δ 153.5, 151.3, 149.4, 138.0, 137.7, 135.6, 133.2, 128.7, 128.3, 128.0, 128.0, 127.7, 123.5, 113.4, 111.9, 110.4, 103.1, 71.3, 43.2, 31.2, 29.0.

υ_{max} (film) 2924, 1483, 1454, 1217, 1204 cm^{-1}.

HRMS (ESI) *m/z* calcd. for $[M+H]^+$ ($C_{23}H_{24}N_3O_1$): 358.1914, found: 358.1922.

2,2,2-Trifluoro-*N*-(2-iodo-4-methoxyphenyl)acetamide (**S20**)

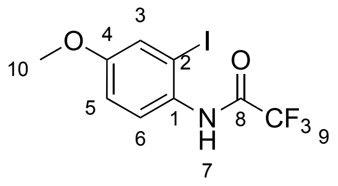

A flame-dried round-bottom flask, under air, was charged with 2-iodo-4-methoxyaniline (385 mg, 1.00 equiv., 1.55 mmol), dry THF (4.0 mL), and NEt_3 (259 µL, 1.20 equiv., 1.86 mmol). The resulting solution was cooled down to 0 ˚C, and trifluoroacetic anhydride (259 µL, 1.20 equiv., 1.86 mmol) was added dropwise. The reaction was then allowed to warm to RT and stir for 23 h. The reaction was diluted with water (10 mL) and the pH was adjusted to approx. 8 by addition of solid $NaHCO_3$. The mixture was cooled to 0 °C and was then filtered and dried under suction to afford the product as a white solid (518 mg, 97%).

^{1}H NMR (500 MHz, $CDCl_3$) δ 8.08 (bs, 1H), 7.99 (d, J = 9.0 Hz, 1H), 7.36 (d, J = 2.8 Hz, 1H), 6.95 (dd, J = 9.0, 2.8 Hz, 1H), 3.80 (s, 3H).

^{13}C NMR (126 MHz, $CDCl_3$) δ 158.1, 154.9 (q, $^2J_{CF}$ = 37.6 Hz), 129.0, 124.4, 123.4, 115.9 (q, $^1J_{CF}$ = 288.6 Hz), 115.1, 91.4, 55.9.

^{19}F NMR (377 MHz, $CDCl_3$) δ −75.75.

υ_{max} (solid) 3275, 1703, 1541, 1481, 1184, 1159 cm^{-1}.

HRMS (ESI) *m/z* calcd. for $[M-H]^-$ ($C_9H_6F_3I_1N_1O_2$): 343.9401, found: 343.9400.

2-(5-Bromopyridin-3-yl)ethan-1-amine (**S21**)

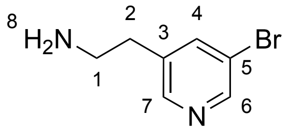

To a round-bottom flask was added 2-(5-bromopyridin-3-yl)acetonitrile (261 mg, 1.00 equiv., 1.32 mmol) and Raney nickel (155 mg, 50 wt% suspension in water, 1.00 equiv., 1.32 mmol). The flask was sealed, and EtOH (10 mL) and NH_4OH (285 µL, 37% in water, 2.00 equiv., 2.65 mmol) were added. The mixture was bubbled through with N_2 for 5 min. Then, H_2 gas was bubbled through the mixture for 10 min. The mixture was stirred at 70 °C under H_2 atmosphere for 16 h. After reaction completion, the mixture was filtered through celite, washed with EtOH (10 mL) and volatiles were removed under

reduced pressure. The residue was purified by column chromatography (silica gel, 5 to 8% MeOH in CH_2Cl_2 + 1% NH_4OH 37% aq. solution) to afford the product as an off-white solid (64.4 mg, 24%).

^{1}H NMR (400 MHz, $CDCl_3$) δ 8.54 (s, 1H), 8.39 (s, 1H), 7.70 (s, 1H), 3.00 (t, J = 7.0 Hz, 2H), 2.74 (t, J = 6.9 Hz, 2H), 1.65 (bs, 2H).

^{13}C NMR (126 MHz, $CDCl_3$) δ 149.0, 148.5, 139.0, 137.2, 120.8, 43.1, 36.7.

The spectral data were consistent with those previously reported in the literature.[27]

tert-Butyl (2-(5-bromopyridin-3-yl)ethyl)carbamate (**S22**)

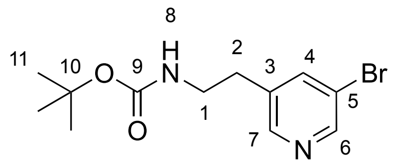

To a round-bottom flask, under air, was added 2-(5-bromopyridin-3-yl)ethan-1-amine (63 mg, 1.0 equiv., 0.30 mmol), 1,4-dioxane (1.0 mL), water (0.50 mL), and $NaHCO_3$ (26 mg, 1.1 equiv., 0.31 mmol). The mixture was cooled to 0 °C, and a solution of Boc_2O (68 mg, 1.1 equiv., 0.31 mmol) in 1,4-dioxane (0.30 mL) was slowly added. The mixture was allowed to warm to RT and stir for 3 h. The mixture was diluted with water (3 mL) and extracted with EtOAc (3 × 5 mL). The combined organic phases were dried over anhydrous Na_2SO_4, filtered, and volatiles were removed under reduced pressure. The residue was purified by column chromatography (silica gel, 25 to 30% EtOAc in hexane) to afford the product as an amber oil which solidified into an orange solid (69 mg, 77%).

^{1}H NMR (500 MHz, $CDCl_3$) δ 8.54 (d, J = 1.5 Hz, 1H), 8.36 (d, J = 1.8 Hz, 1H), 7.68 (s, 1H), 4.61 (s, 1H), 3.37 (q, J = 6.8 Hz, 2H), 2.80 (t, J = 7.0 Hz, 2H), 1.43 (s, 9H).

^{13}C NMR (126 MHz, $CDCl_3$) δ 155.9, 149.2, 148.5, 139.1, 136.5, 120.9, 79.8, 41.4, 33.2, 28.5.

υ_{max} (film) 3335, 2976, 1688, 1522, 1250, 1163 cm^{-1}.

HRMS (ESI) *m/z* calcd. for $[M+H]^+$ ($C_{12}H_{18}Br_1N_2O_2$): 301.0546, found: 301.0541.

tert-Butyl (2-(5-((trimethylsilyl)ethynyl)pyridin-3-yl)ethyl)carbamate (**S23**)

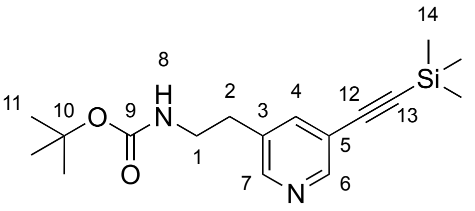

Prepared according to General Procedure C from *tert*-butyl (2-(5-bromopyridin-3-yl)ethyl)carbamate (133 mg, 1.00 equiv., 0.443 mmol) and ethynyltrimethylsilane (0.61 mL, 10 equiv., 4.4 mmol) at 90 °C for 3 h. Purified by column chromatography (10 to 25% EtOAc in hexane) to afford the product as a clear oil (141 mg, 97%).

^{1}H NMR (400 MHz, $CDCl_3$) δ 8.53 (s, 1H), 8.37 – 8.27 (m, 1H), 7.57 (s, 1H), 4.69 (s, 1H), 3.33 (q, J = 6.7 Hz, 2H), 2.76 (t, J = 7.0 Hz, 2H), 1.41 (m, 9H), 0.36 – 0.03 (m, 9H).

^{13}C NMR (101 MHz, $CDCl_3$) δ 155.9, 150.7, 149.3, 139.2, 134.0, 120.2, 101.5, 98.4, 79.6, 41.4, 33.2, 28.5, −0.1.

υ_{max} (film) 3323, 2926, 2160, 1694, 1250, 843 cm^{-1}.

HRMS (ESI) *m/z* calcd. for $[M+Na]^+$ ($C_{17}H_{26}N_2O_2Si_1Na_1$): 341.1661, found: 341.1656.

tert-Butyl (2-(5-ethynylpyridin-3-yl)ethyl)carbamate (**S24**)

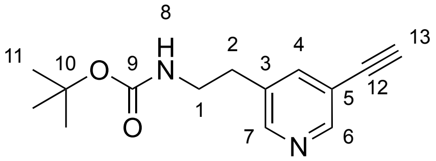

To a vial, under air, was added *tert*-butyl (2-(5-((trimethylsilyl)ethynyl)pyridin-3-yl)ethyl)carbamate (130 mg, 1.00 equiv., 0.408 mmol), MeOH (2.0 mL), and K_2CO_3 (113 mg, 2.00 equiv., 0.816 mmol). The mixture was stirred at RT for 4.5 h. The mixture was then diluted with water (10 mL) and extracted with CH_2Cl_2 (3 × 15 mL). The combined organic phases were dried over anhydrous Na_2SO_4, filtered, and volatiles were removed under reduced pressure. The residue was purified by column chromatography (silica gel, 10 to 30% EtOAc in hexane) to afford the product as a brown oil (74.2 mg, 74%).

^{1}H NMR (500 MHz, $CDCl_3$) δ 8.56 (bs, 1H), 8.39 (bs, 1H), 7.59 (s, 1H), 4.76 (s, 1H), 3.34 (q, J = 6.7 Hz, 2H), 3.19 (s, 1H), 2.78 (t, J = 7.0 Hz, 2H), 1.40 (s, 9H).

^{13}C NMR (126 MHz, $CDCl_3$) δ 155.9, 150.9, 149.7, 139.4, 134.2, 119.2, 80.8, 80.4, 79.6, 41.4, 33.2, 28.4.

υ_{max} (film) 3294, 2976, 1690, 1518, 1366, 1273, 1248, 1163, 1026 cm^{-1}.

HRMS (ESI) *m/z* calcd. for $[M+Na]^+$ ($C_{14}H_{18}N_2O_2Na_1$): 269.1266, found: 269.1262.

tert-Butyl (2-(5-(5-methoxy-1*H*-indol-2-yl)pyridin-3-yl)ethyl)carbamate (S25)

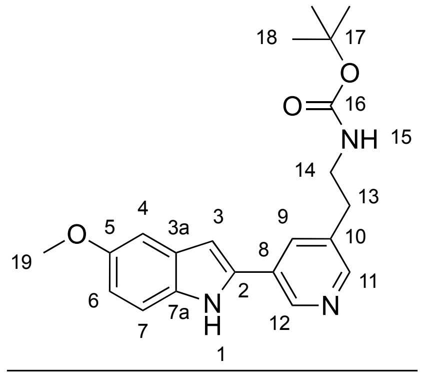

An oven-dried microwave vial was charged with 2,2,2-trifluoro-*N*-(2-iodo-4-methoxyphenyl)acetamide (82.7 mg, 1.20 equiv., 0.240 mmol), 2-(5-(2-((*tert*-butoxycarbonyl)amino)ethyl)pyridin-3-yl)ethyn-1-ylium (49.0 mg, 1.00 equiv., 0.200 mmol), (1,10-phenanthroline)bis(triphenylphosphine)copper(I) nitrate dichloromethane adduct (18.3 mg, 0.100 equiv., 20.0 μmol), and K_3PO_4 (84.8 mg, 2.00 equiv., 0.400 mmol). The vial was sealed, evacuated and re-filled with N_2 three times. Toluene (1.00 mL) was added, and the mixture was heated to 110 °C for 24 h. After cooling to RT, the mixture was diluted with EtOAc (10 mL), washed with water (10 mL), dried over anhydrous Na_2SO_4, filtered, and volatiles were removed under reduced pressure. The crude residue was purified by column chromatography (silica gel, 20 to 50% EtOAc in hexane), then re-purified by column chromatography (silica gel, Et_2O) to afford the product as a yellow solid (22.2 mg, 30%).

^{1}H NMR (500 MHz, $CDCl_3$) δ 8.93 (s, 1H), 8.82 (s, 1H), 8.35 (s, 1H), 7.81 (s, 1H), 7.30 (d, *J* = 8.8 Hz, 1H), 7.09 (d, *J* = 2.4 Hz, 1H), 6.88 (dd, *J* = 8.8, 2.4 Hz, 1H), 6.80 (s, 1H), 4.73 (bs, 1H), 3.87 (s, 3H), 3.49 – 3.41 (m, 2H), 2.86 (t, *J* = 7.0 Hz, 2H), 1.42 (s, 9H).

^{13}C NMR (126 MHz, $CDCl_3$) δ 156.2, 154.7, 148.8, 144.8, 135.2, 134.7, 132.6, 132.4, 131.0, 129.6, 113.5, 112.1, 102.3, 101.0, 79.8, 56.0, 41.2, 33.5, 28.5.

υ_{max} (film): 3161, 2930, 1688, 1165, 779 cm^{-1}.

HRMS (ESI) m/z calcd. for $[M+H]^+$ ($C_{21}H_{26}N_3O_3$): 368.19687, found: 368.1970.

<u>*N,N*-Dibenzyl-3-butyn-1-amine (**S26**)</u>

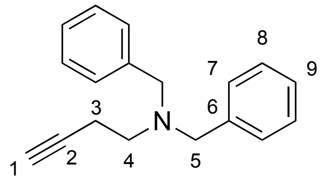

A round-bottom flask under air was charged with *O*-tosyl-butyn-1-ol (5.65 g, 1.00 equiv., 25.2 mmol), dibenzylamine (25 mL, 5.2 equiv. 130 mmol), K_2CO_3 (3.46 g, 1.0 equiv., 25.0 mmol), and KI (415 mg, 0.10 equiv., 2.50 mmol). The mixture was heated to 80 °C for 24 h. After cooling to RT, the reaction mixture was diluted with water/brine (1:1, 40 mL), and extracted with EtOAc (2 × 60 mL). The combined organic phases were washed with brine (20 mL), dried over anhydrous Na_2SO_4, filtered, and volatiles were removed under reduced pressure. The residue was purified by column chromatography (silica gel, 0 to 4% EtOAc in hexane) to afford the product as a clear oil (4.97 g, 79%).

^{1}H NMR (400 MHz, $CDCl_3$) δ 7.43 (d, J = 7.4 Hz, 4H), 7.36 (t, J = 7.5 Hz, 4H), 7.33 – 7.24 (m, 2H), 3.67 (s, 4H), 2.75 (t, J = 7.4 Hz, 2H), 2.41 (td, J = 7.4, 2.7 Hz, 2H), 1.99 (t, J = 2.7 Hz, 1H).

^{13}C NMR (126 MHz, $CDCl_3$) δ 139.6, 128.9, 128.4, 127.1, 83.1, 69.1, 58.2, 52.2, 17.2.

The spectral data were consistent with those previously reported in the literature.[28]

<u>*N,N*-Dibenzyl-4-(pyridin-3-yl)but-3-yn-1-amine (**S27**)</u>

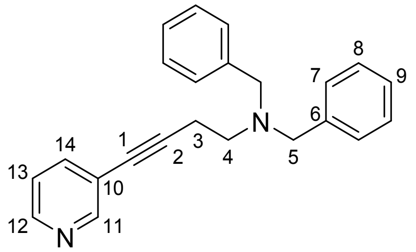

Prepared according to General Procedure C from *N,N*-dibenzyl-3-butyn-1-amine (499 mg, 1.00 equiv., 2.00 mmol) and 3-bromopyridine (293 µL, 1.50 equiv., 3.00 mmol) at 90 °C for 2.5 h. Purified by column chromatography (10 to 20% EtOAc in hexane) to afford the product as a yellow solid (497 mg, 76%).

^{1}H NMR (400 MHz, $CDCl_3$) δ 8.64 (bs, 1H), 8.51 (bs, 1H), 7.65 (dt, J = 7.9, 1.8 Hz, 1H), 7.42 (d, J = 6.8 Hz, 4H), 7.32 (t, J = 7.2 Hz, 4H), 7.27 – 7.20 (m, 3H), 3.68 (s, 4H), 2.79 (t, J = 7.3 Hz, 2H), 2.61 (t, J = 7.0 Hz, 2H).

^{13}C NMR (101 MHz, $CDCl_3$) δ 152.5, 148.1, 139.6, 138.5, 128.8, 128.5, 128.4, 127.1, 123.1, 92.7, 78.2, 58.3, 52.1, 18.3.

υ_{max} (film) 3026, 1450, 1406, 733, 696 cm^{-1}.

HRMS (ESI) *m/z* calcd. for $[M+H]^+$ ($C_{23}H_{23}N_2$): 327.1856, found: 327.1848.

N,N-Dibenzyl-2-(5-(benzyloxy)-3-(pyridin-3-yl)-1*H*-indol-2-yl)ethan-1-amine (**S28**)

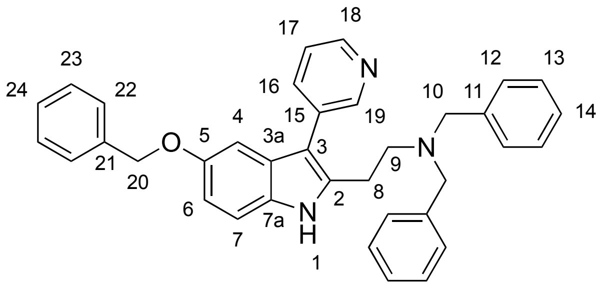

An oven-dried microwave vial was charged with *N*-(4-(benzyloxy)-2-iodophenyl)acetamide (73.3 mg, 1.33 equiv., 0.200 mmol), *N,N*-dibenzyl-4-(pyridin-3-yl)but-3-yn-1-amine (49.0 mg, 1.00 equiv., 0.150 mmol), $Pd(OAc)_2$ (0.4 mg, 1 mol%, 2 μmol), 1-(4-methoxyphenyl)urea (1.0 mg, 4.0 mol%, 6.0 μmol), and K_2CO_3 (62.2 mg, 3.00 equiv., 0.450 mmol). The vial was sealed, evacuated and re-filled with N_2 three times. Anhydrous DMF (0.50 mL) was added, and the mixture was stirred at 110 °C for 20 h. After cooling to RT, the mixture was diluted with EtOAc (12 mL) and washed with 10% LiCl solution (2 × 10 mL). The organic phase was dried over anhydrous Na_2SO_4, filtered, and volatiles were removed under reduced pressure. The residue was purified by column chromatography (silica gel, 10 to 40% EtOAc in hexane) to obtain the product as a brown solid (21.8 mg, 28%).

^{1}H NMR (400 MHz, $CDCl_3$) δ 9.81 (s, 1H), 8.64 – 8.59 (m, 1H), 8.45 (dd, J = 4.8, 1.7 Hz, 1H), 7.62 (dt, J = 7.9, 1.9 Hz, 1H), 7.40 (d, J = 7.3 Hz, 2H), 7.36 – 7.13 (m, 15H), 7.08 (d, J = 2.4 Hz, 1H), 6.88 (dd, J = 8.7, 2.4 Hz, 1H), 5.00 (s, 2H), 3.63 (s, 4H), 3.01 (t, J = 6.0 Hz, 2H), 2.81 (t, J = 6.0 Hz, 2H).

^{13}C NMR (101 MHz, $CDCl_3$) δ 153.9, 150.2, 146.8, 138.0, 137.7, 136.9, 136.4, 131.7, 130.6, 129.4, 128.7, 128.6, 127.9, 127.7, 127.7, 127.5, 123.6, 112.4, 111.7, 110.0, 102.1, 71.1, 58.4, 52.6, 23.2.

υ_{max} (film) 1493, 1481, 1452, 1152, 1026, 799, 733, 696 cm^{-1}.

HRMS (ESI) *m/z* calcd. for $[M+Na]^+$ ($C_{36}H_{33}N_3O_1Na_1$): 546.2516, found: 546.2517.

S5. References

(1) Hank, E. C.; D'Arcy-Evans, N.; Scaletti, E. R.; Benítez-Buelga, C.; Wallner, O.; Ortis, F.; Zhou, K.; Meng, L.; Calvo, P.; Almlöf, I.; Wiita, E.; Kosenina, S.; Krämer, A.; Long, M.; Jemth, A.-S.; Dawson, H.; Stewart, J.; Dickey, A.; Astorga, M. E.; Varga, M.; Homan, E. J.; Scobie, M.; Knapp, S.; Vega, M. de; Sastre, L.; Stenmark, P.; Helleday, T.; Michel, M. Nucleobase Catalysts for the Enzymatic Activation of 8-Oxoguanine DNA Glycosylase 1. ChemRxiv January 2, 2025. https://doi.org/10.26434/chemrxiv-2023-70gws-v4.

(2) Kehler, M.; Zhou, K.; Kemas, A. M.; del Prado, A.; Hutchinson, E. S.; Nairn, E. H.; Varga, M.; Plattner, Y.; Zhong, Y.; Purewal-Sidhu, O.; Haslam, J.; Wiita, E.; Gildie, H.; Singerova, K.; Szaruga, Z.; Almlöf, I.; Hormann, F. M.; Liu, K.-C.; Wallner, O.; Ortis, F.; Homan, E. J.; Gileadi, O.; Rudd, S. G.; Stenmark, P.; de Vega, M.; Helleday, T.; D'Arcy-Evans, N. D.; Lauschke, V. M.; Michel, M. Organocatalytic Switches of DNA Glycosylase OGG1 Catalyze a Highly Efficient AP-Lyase Function. *Chem. – Eur. J. n/a* (n/a), e202500382. https://doi.org/10.1002/chem.202500382.

(3) Kabsch, W. XDS. *Acta Crystallogr. D Biol. Crystallogr.* **2010**, *66* (2), 125–132. https://doi.org/10.1107/S0907444909047337.

(4) Evans, P. Scaling and Assessment of Data Quality. *Acta Crystallogr. D Biol. Crystallogr.* **2006**, *62* (1), 72–82. https://doi.org/10.1107/S0907444905036693.

(5) Project, C. C.; Number 4. The CCP4 Suite: Programs for Protein Crystallography. *Acta Crystallogr. D Biol. Crystallogr.* **1994**, *50* (5), 760–763. https://doi.org/10.1107/S0907444994003112.

(6) Vagin, A.; Teplyakov, A. MOLREP: An Automated Program for Molecular Replacement. *J. Appl. Crystallogr.* **1997**, *30* (6), 1022–1025. https://doi.org/10.1107/S0021889897006766.

(7) Emsley, P.; Cowtan, K. Coot: Model-Building Tools for Molecular Graphics. *Acta Crystallogr. D Biol. Crystallogr.* **2004**, *60* (12), 2126–2132. https://doi.org/10.1107/S0907444904019158.

(8) Murshudov, G. N.; Skubák, P.; Lebedev, A. A.; Pannu, N. S.; Steiner, R. A.; Nicholls, R. A.; Winn, M. D.; Long, F.; Vagin, A. A. REFMAC5 for the Refinement of Macromolecular Crystal Structures. *Acta Crystallogr. D Biol. Crystallogr.* **2011**, *67* (4), 355–367. https://doi.org/10.1107/S0907444911001314.

(9) Michel, M.; Homan, E. J.; Wiita, E.; Pedersen, K.; Almlöf, I.; Gustavsson, A.-L.; Lundbäck, T.; Helleday, T.; Warpman Berglund, U. In Silico Druggability Assessment of the NUDIX Hydrolase Protein Family as a Workflow for Target Prioritization. *Front. Chem.* **2020**, *8*. https://doi.org/10.3389/fchem.2020.00443.

(10) Page, B. D. G.; Valerie, N. C. K.; Wright, R. H. G.; Wallner, O.; Isaksson, R.; Carter, M.; Rudd, S. G.; Loseva, O.; Jemth, A.-S.; Almlöf, I.; Font-Mateu, J.; Llona-Minguez, S.; Baranczewski, P.; Jeppsson, F.; Homan, E.; Almqvist, H.; Axelsson, H.; Regmi, S.; Gustavsson, A.-L.; Lundbäck, T.; Scobie, M.; Strömberg, K.; Stenmark, P.; Beato, M.; Helleday, T. Targeted NUDT5 Inhibitors Block Hormone Signaling in Breast Cancer Cells. *Nat. Commun.* **2018**, *9* (1), 250. https://doi.org/10.1038/s41467-017-02293-7.

(11) Zhang, S. M.; Desroses, M.; Hagenkort, A.; Valerie, N. C. K.; Rehling, D.; Carter, M.; Wallner, O.; Koolmeister, T.; Throup, A.; Jemth, A.-S.; Almlöf, I.; Loseva, O.; Lundbäck, T.; Axelsson, H.; Regmi, S.; Sarno, A.; Krämer, A.; Pudelko, L.; Bräutigam, L.; Rasti, A.; Göttmann, M.; Wiita, E.; Kutzner, J.; Schaller, T.; Kalderén, C.; Cázares-Körner, A.; Page, B. D. G.; Krimpenfort, R.; Eshtad, S.; Altun, M.; Rudd, S. G.; Knapp, S.; Scobie, M.; Homan, E. J.; Berglund, U. W.; Stenmark, P.; Helleday, T. Development of a Chemical Probe against NUDT15. *Nat. Chem. Biol.* **2020**, *16* (10), 1120–1128. https://doi.org/10.1038/s41589-020-0592-z.

(12) Lizos, D. E.; Murphy, J. A. Concise Synthesis of (±)-Horsfiline and (±)-Coerulescine by Tandem Cyclisation of Iodoaryl Alkenyl Azides. *Org. Biomol. Chem.* **2003**, *1* (1), 117–122. https://doi.org/10.1039/B208114H.

(13) Li, Z.; Sun, K.; Cai, C. Cobalt(II)-Catalyzed Regioselective C–H Halogenation of Anilides. *Org. Biomol. Chem.* **2018**, *16* (30), 5433–5440. https://doi.org/10.1039/C8OB01448E.

(14) Zhou, F.; Han, X.; Lu, X. Synthesis of Indoles via Palladium-Catalyzed C–H Activation of *N*-Aryl Amides Followed by Coupling with Alkynes. *Tetrahedron Lett.* **2011**, *52* (36), 4681–4685. https://doi.org/10.1016/j.tetlet.2011.07.009.

(15) Ma, F.; Xie, X.; Zhang, L.; Peng, Z.; Ding, L.; Fu, L.; Zhang, Z. Palladium-Catalyzed Amidation of Aryl Halides Using 2-Dialkylphosphino-2′-Alkoxyl-1,1′-Binaphthyl as Ligands. *J. Org. Chem.* **2012**, *77* (12), 5279–5285. https://doi.org/10.1021/jo3005827.

(16) Dumez, E.; Snaith, J. S.; Jackson, R. F. W.; McElroy, A. B.; Overington, J.; Wythes, M. J.; Withka, J. M.; McLellan, T. J. Synthesis of Macrocyclic, Potential Protease Inhibitors Using a Generic Scaffold. *J. Org. Chem.* **2002**, *67* (14), 4882–4892. https://doi.org/10.1021/jo025615o.

(17) Wu, W.; Li, Z.; Zhou, G.; Jiang, S. Total Synthesis of Argyrins A and E. *Tetrahedron Lett.* **2011**, *52* (19), 2488–2491. https://doi.org/10.1016/j.tetlet.2011.03.021.

(18) Bell, G. E.; Fyfe, J. W. B.; Israel, E. M.; Slawin, A. M. Z.; Campbell, M.; Watson, A. J. B. Synthesis of 2-BMIDA Indoles via Heteroannulation: Applications in Drug Scaffold and Natural Product Synthesis. *Org. Lett.* **2022**, *24* (16), 3024–3027. https://doi.org/10.1021/acs.orglett.2c00959.

(19) Tasker, S. Z.; Jamison, T. F. Highly Regioselective Indoline Synthesis under Nickel/Photoredox Dual Catalysis. *J. Am. Chem. Soc.* **2015**, *137* (30), 9531–9534. https://doi.org/10.1021/jacs.5b05597.

(20) Kathiravan, S.; Nicholls, I. A. Monoprotected L-Amino Acid (l-MPAA), Accelerated Bromination, Chlorination, and Iodination of C(Sp2)−H Bonds by Iridium(III) Catalysis. *Chem. – Eur. J.* **2017**, *23* (29), 7031–7036. https://doi.org/10.1002/chem.201700280.

(21) Mahajan, P. S.; Humne, V. T.; Tanpure, S. D.; Mhaske, S. B. Radical Beckmann Rearrangement and Its Application in the Formal Total Synthesis of Antimalarial Natural Product Isocryptolepine via C–H Activation. *Org. Lett.* **2016**, *18* (14), 3450–3453. https://doi.org/10.1021/acs.orglett.6b01634.

(22) Chuang, H.-Y.; Schupp, M.; Meyrelles, R.; Maryasin, B.; Maulide, N. Redox-Neutral Selenium-Catalysed Isomerisation of Para-Hydroxamic Acids into Para-Aminophenols. *Angew. Chem. Int. Ed.* **2021**, *60* (25), 13778–13782. https://doi.org/10.1002/anie.202100801.

(23) Liddon, J. T. R.; James, M. J.; Clarke, A. K.; O'Brien, P.; Taylor, R. J. K.; Unsworth, W. P. Catalyst-Driven Scaffold Diversity: Selective Synthesis of Spirocycles, Carbazoles and Quinolines from Indolyl Ynones. *Chem. – Eur. J.* **2016**, *22* (26), 8777–8780. https://doi.org/10.1002/chem.201601836.

(24) Russo, O.; Messaoudi, S.; Hamze, A.; Olivi, N.; Peyrat, J.-F.; Brion, J.-D.; Sicsic, S.; Berque-Bestel, I.; Alami, M. Three-Component One-Pot Process to Propargylic Amines and Related Amide and Sulfonamide Compounds: Application to the Construction of 2-(Aminomethyl)Benzofurans and Indoles. *Tetrahedron* **2007**, *63* (43), 10671–10683. https://doi.org/10.1016/j.tet.2007.07.096.

(25) Westley, E.; Sowden, M. J.; Magann, N. L.; Horvath, K. L.; Connor, K. P. E.; Sherburn, M. S. Substituted Tetraethynylethylene–Tetravinylethylene Hybrids. *J. Am. Chem. Soc.* **2022**, *144* (2), 977–986. https://doi.org/10.1021/jacs.1c11598.

(26) Schmidt, B.; Berger, R.; Hölter, F. Functionalized Alkoxy Arene Diazonium Salts from Paracetamol. *Org. Biomol. Chem.* **2010**, *8* (6), 1406–1414. https://doi.org/10.1039/B924619C.

(27) Kauffmann, T.; Fischer, H. Hetarine, XVI. Seitenkettencyclisierung Bei 3,4-Dehydropyridin-Derivaten. *Chem. Ber.* **1973**, *106* (1), 220–227. https://doi.org/10.1002/cber.19731060127.

(28) Quinodoz, P.; Wright, K.; Drouillat, B.; Kletskii, M. E.; Burov, O. N.; Lisovin, Anton. V.; Couty, F. α-Hydroxy-Tetrazoles as Latent Ethynyl Moieties: A Mechanistic Investigation. *Eur. J. Org. Chem.* **2019**, *2019* (2–3), 342–350. https://doi.org/10.1002/ejoc.201800143.

S6. LCMS Traces

Compound 2

Method Info : X-bridge
10-97% MeCN-3min, ACE C8, 50x3.0 mm, 3u, 1ml/min, 215-395 & 254 nm,C:
NH4HCO3 10mM, B: MeCN

Sample Info : Walkup method: 'X1097-3'
Target:

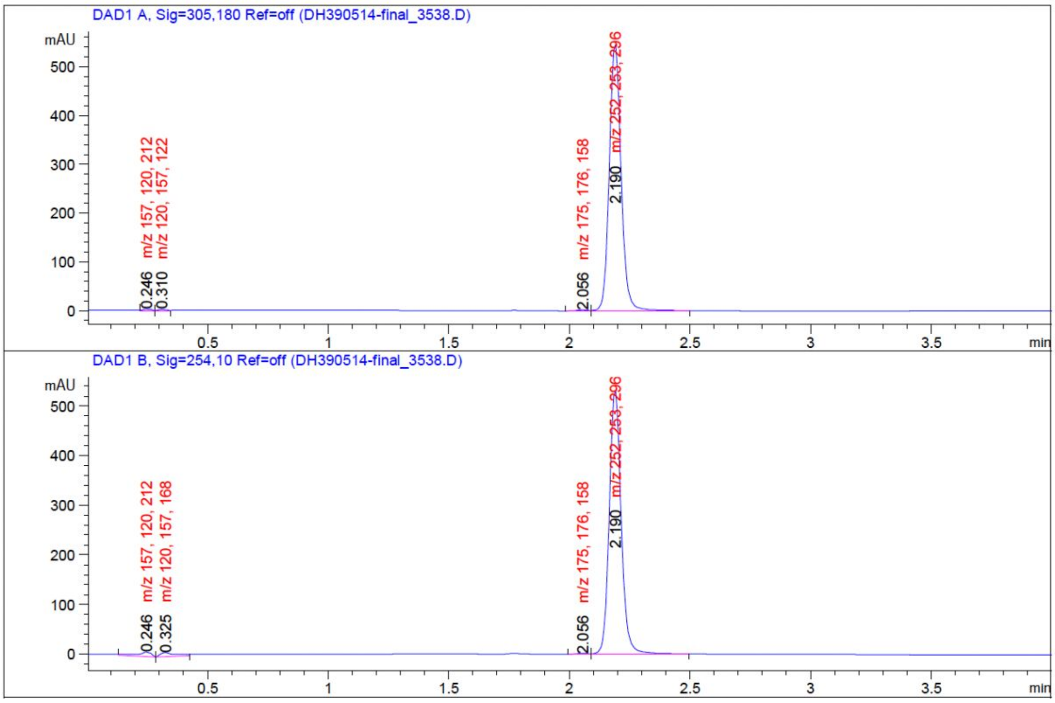

Signal 1: DAD1 A, Sig=305,180 Ref=off

Peak #	RetTime [min]	Type	Width [min]	Area [mAU*s]	Height [mAU]	Area %
1	0.246	BV	0.0355	7.17669	3.28555	0.3844
2	0.310	VV	0.0367	5.33570	2.32750	0.2858
3	2.056	BV	0.0482	6.23684	2.00938	0.3341
4	2.190	VV	0.0515	1848.15576	546.35205	98.9957

Totals : 1866.90499 553.97448

Signal 2: DAD1 B, Sig=254,10 Ref=off

Peak #	RetTime [min]	Type	Width [min]	Area [mAU*s]	Height [mAU]	Area %
1	0.246	BV	0.0597	37.80506	9.25353	1.9992
2	0.325	VB	0.0633	35.55917	8.09341	1.8804
3	2.056	BV	0.0473	5.71184	1.89017	0.3020
4	2.190	VV	0.0517	1811.97375	532.63269	95.8184

Totals : 1891.04982 551.86980

Compound 3

[illegible]
STANDARD METHOD FOR REGISTRATION INTO CHEMSPEC
ACE 3 C8 50x3.0 mm, 10-97% acetonitrile in 3 min, 1 ml/min
215-395 & 254 nm

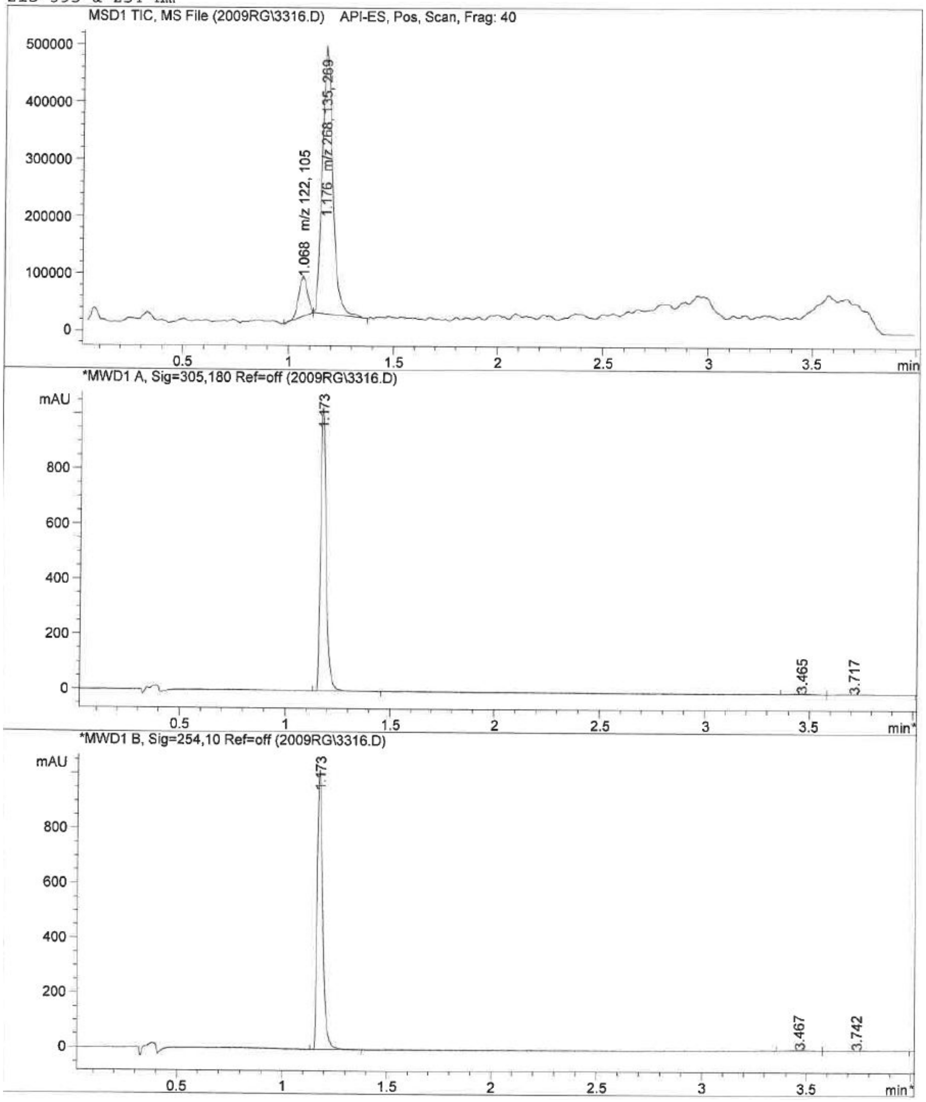

Signal 2: MWD1 A, Sig=305,180 Ref=off
Signal has been modified after loading from rawdata file!

Peak #	RetTime [min]	Type	Width [min]	Area [mAU*s]	Height [mAU]	Area %
1	1.173	BBA	0.0288	1908.82935	977.69812	97.5750
2	3.465	BB	0.0984	20.07162	2.91194	1.0260
3	3.717	BB	0.1839	27.36876	1.90121	1.3990
Totals :				1956.26973	982.51127	

Signal 3: MWD1 B, Sig=254,10 Ref=off
Signal has been modified after loading from rawdata file!

Peak #	RetTime [min]	Type	Width [min]	Area [mAU*s]	Height [mAU]	Area %
1	1.173	BBA	0.0286	1858.11804	959.82458	97.4000
2	3.467	BB	0.0884	20.14934	2.92386	1.0562
3	3.742	BB	0.1609	29.45149	2.19061	1.5438
Totals :				1907.71888	964.93906	

Compound 4

Method Info : X-bridge C18, 50x3.0 mm, 3.5u, 10-97% MeCN 3min,1ml/min, B: MeCN C: NH4HCO3

Sample Info : Walkup method: 'X1097-3'
Target:

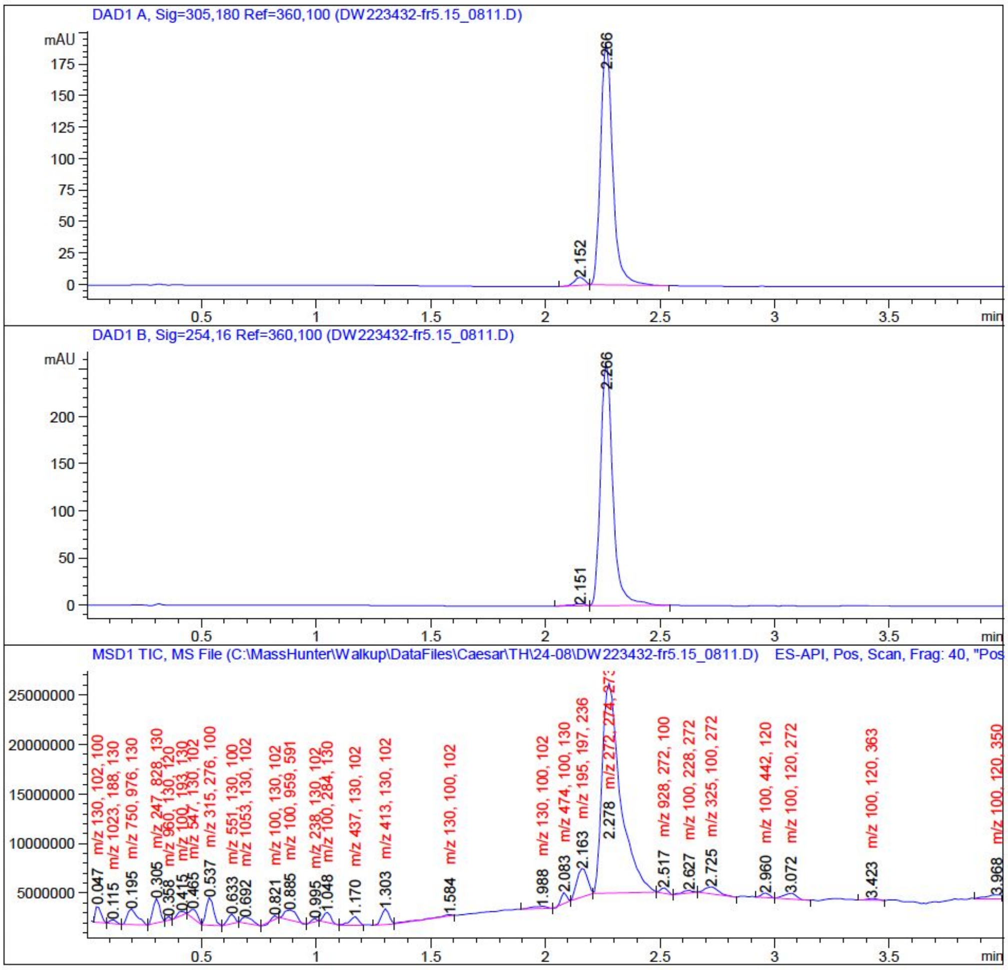

Signal 1: DAD1 A, Sig=305,180 Ref=360,100

Peak #	RetTime [min]	Type	Width [min]	Area [mAU*s]	Height [mAU]	Area %
1	2.152	BB	0.0497	18.87810	6.16346	2.6062
2	2.266	BB	0.0570	705.47223	191.43184	97.3938
Totals :				724.35033	197.59530	

Signal 2: DAD1 B, Sig=254,16 Ref=360,100

Peak #	RetTime [min]	Type	Width [min]	Area [mAU*s]	Height [mAU]	Area %
1	2.151	BB	0.0562	8.20109	2.16569	0.8482
2	2.266	BB	0.0576	958.71301	256.60294	99.1518
Totals :				966.91411	258.76863	

Compound 5

[illegible]
ACE 3 C8 50x3.0 mm, 10-97% acetonitrile in 3 min, 1 ml/min
215-395 & 254 nm

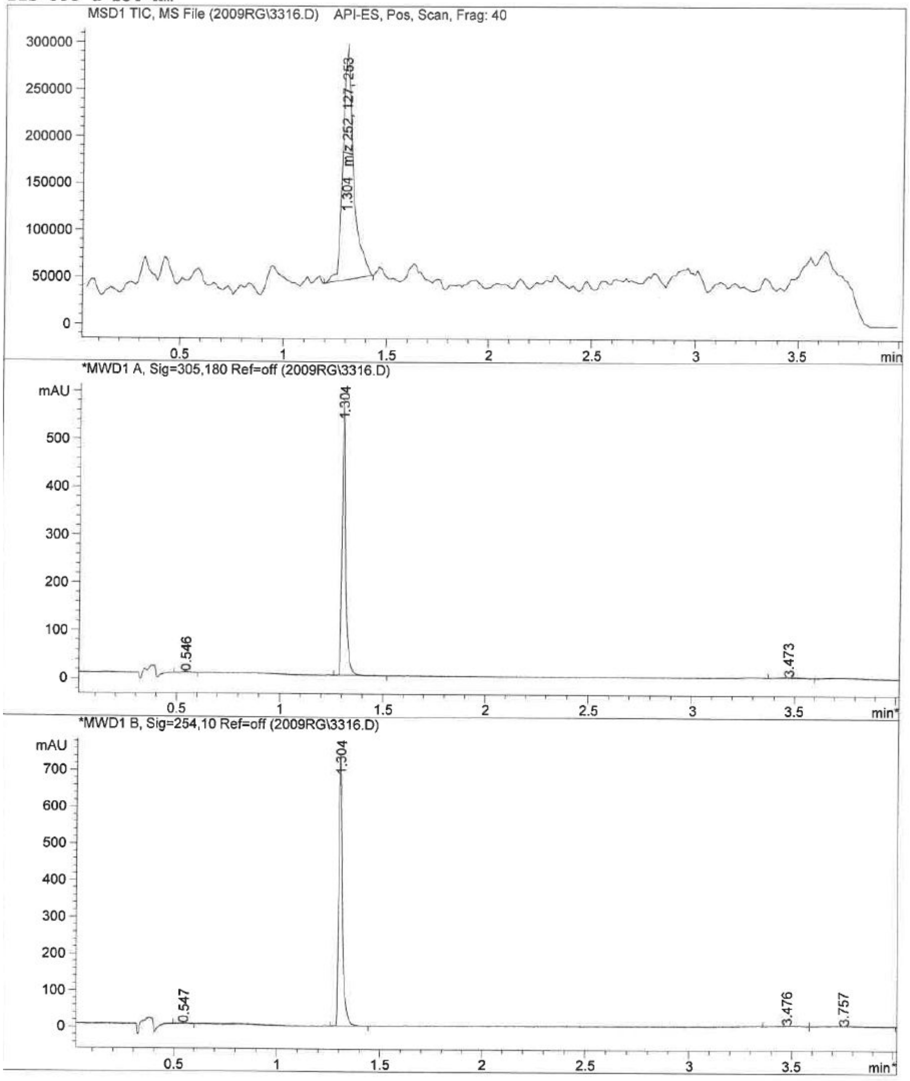

Signal 2: MWD1 A, Sig=305,180 Ref=off
Signal has been modified after loading from rawdata file!

Peak #	RetTime [min]	Type	Width [min]	Area [mAU*s]	Height [mAU]	Area %
1	0.546	BB	0.0348	5.38551	2.34247	0.7426
2	1.304	BB	0.0206	701.26166	559.08569	96.7006
3	3.473	BB	0.0987	18.54117	2.74939	2.5567
Totals :				725.18833	564.17755	

Signal 3: MWD1 B, Sig=254,10 Ref=off
Signal has been modified after loading from rawdata file!

Peak #	RetTime [min]	Type	Width [min]	Area [mAU*s]	Height [mAU]	Area %
1	0.547	BB	0.0313	6.02004	2.77888	0.6281
2	1.304	BB	0.0206	902.85547	722.67120	94.1989
3	3.476	BB	0.0889	20.79367	3.07278	2.1695
4	3.757	BB	0.1734	28.78713	2.13429	3.0035
Totals :				958.45631	730.65715	

Compound 6

Method Info : X-bridge C18, 50x3.0 mm, 3.5u, 5-60% MeCN 3min,1ml/min, B: MeCN C: NH4HCO3

Sample Info : Walkup method: 'X0560-3'
Target:

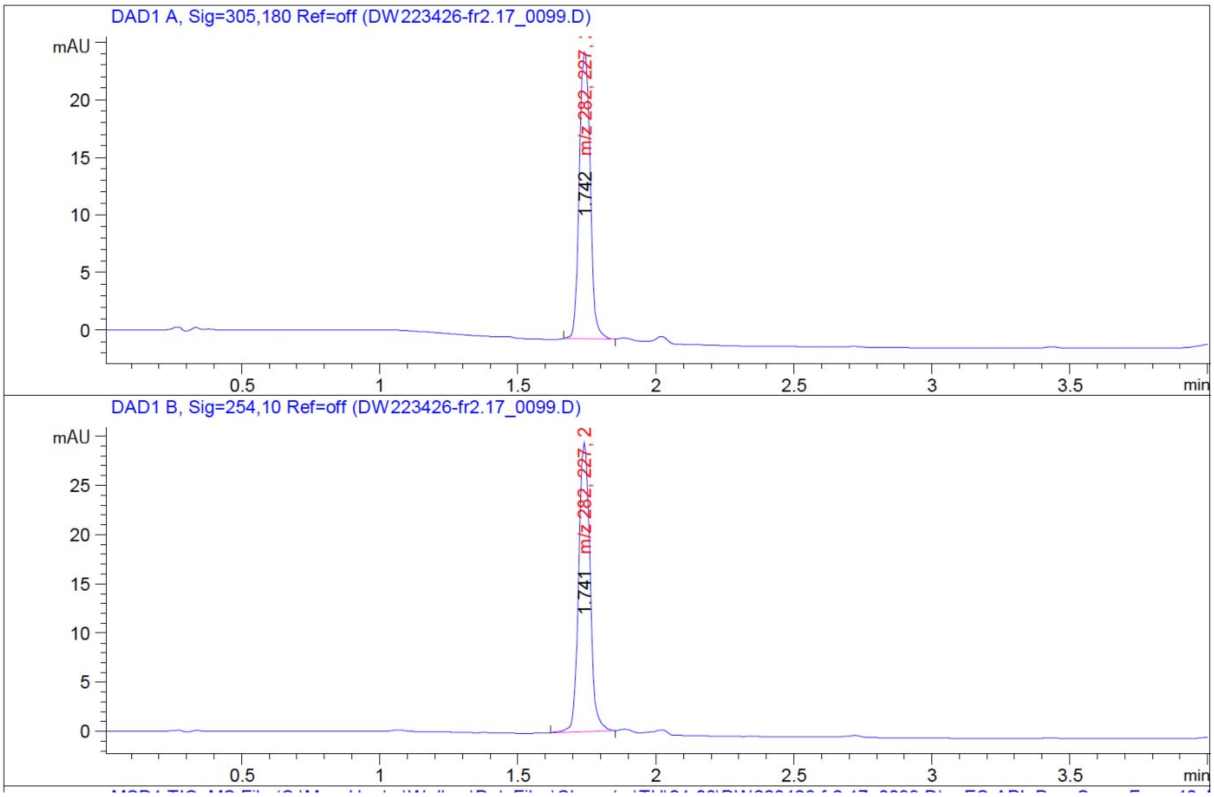

Signal 1: DAD1 A, Sig=305,180 Ref=off

Peak #	RetTime [min]	Type	Width [min]	Area [mAU*s]	Height [mAU]	Area %
1	1.742	BB	0.0435	67.67715	25.07649	100.0000
Totals :				67.67715	25.07649	

Signal 2: DAD1 B, Sig=254,10 Ref=off

Peak #	RetTime [min]	Type	Width [min]	Area [mAU*s]	Height [mAU]	Area %
1	1.741	BB	0.0450	83.27351	29.41354	100.0000
Totals :				83.27351	29.41354	

Compound 8

Method Info : X-bridge C18, 50x3.0 mm, 3.5u, 10-97% MeCN 3min,1ml/min, B: MeCN C: NH4HCO3

Sample Info : Walkup method: 'X1097-3'
Target:

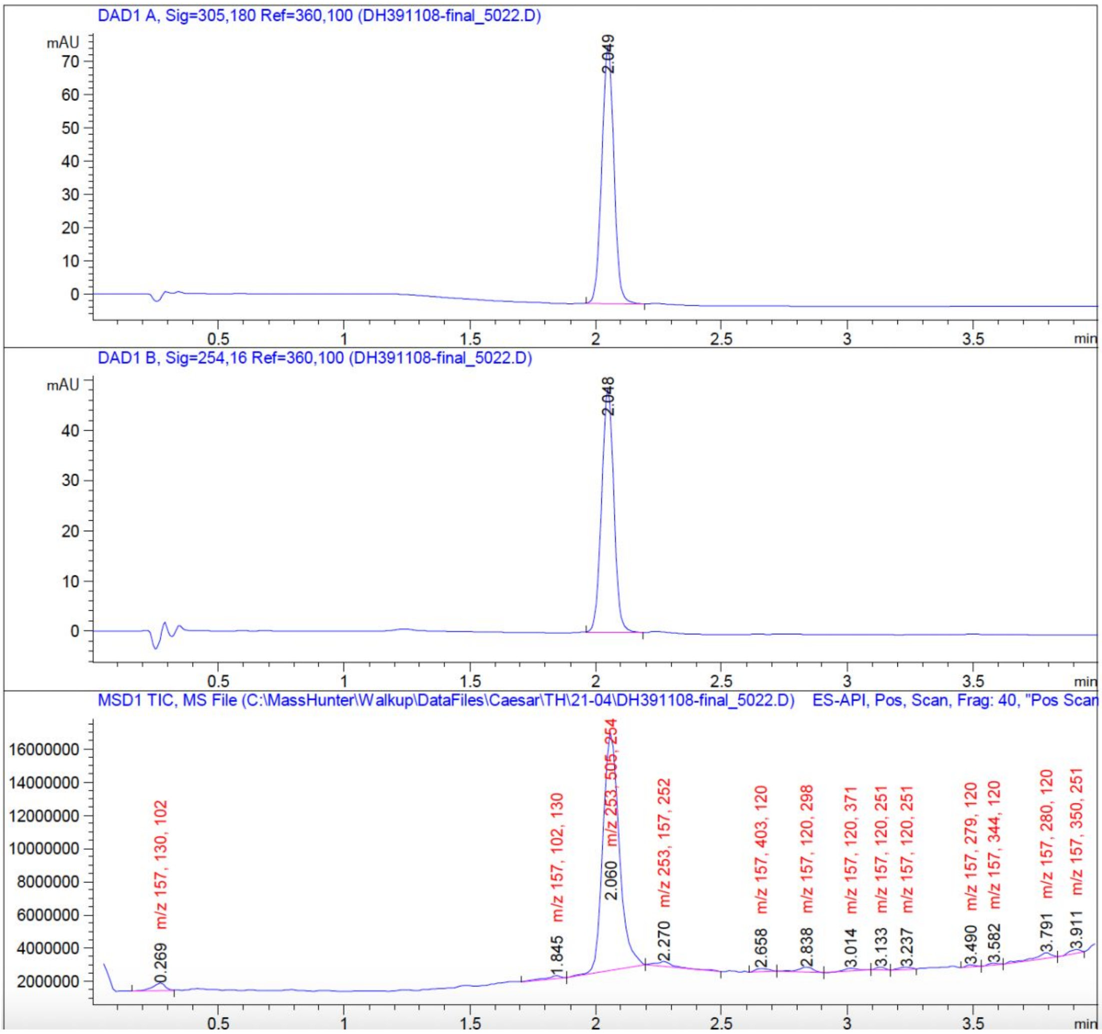

Signal 1: DAD1 A, Sig=305,180 Ref=360,100

Peak #	RetTime [min]	Type	Width [min]	Area [mAU*s]	Height [mAU]	Area %
1	2.049	BB	0.0528	257.83832	77.45622	100.0000

Totals : 257.83832 77.45622

Signal 2: DAD1 B, Sig=254,16 Ref=360,100

Peak #	RetTime [min]	Type	Width [min]	Area [mAU*s]	Height [mAU]	Area %
1	2.048	BB	0.0535	164.55159	48.61157	100.0000

Totals : 164.55159 48.61157

Compound 9

ACE 3 C8 50x3.0 mm, 10-97% acetonitrile in 3 min, 1 ml/min

215-395 & 254 nm

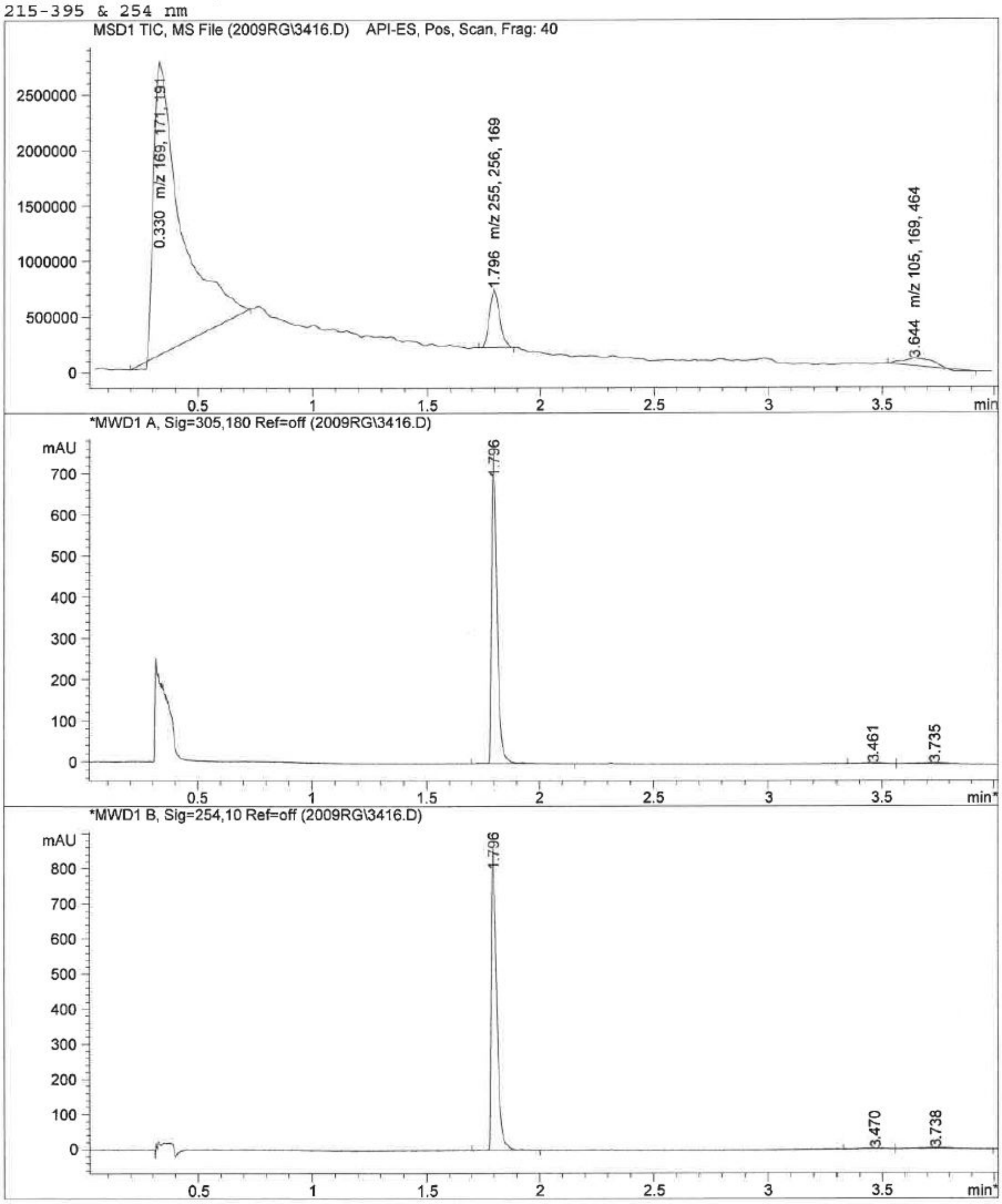

Signal 2: MWD1 A, Sig=305,180 Ref=off

Peak #	RetTime [min]	Type	Width [min]	Area [mAU*s]	Height [mAU]	Area %
1	1.776	MM	0.0278	1321.03687	792.33887	100.0000
Totals :				1321.03687	792.33887	

Signal 3: MWD1 B, Sig=254,10 Ref=off

Peak #	RetTime [min]	Type	Width [min]	Area [mAU*s]	Height [mAU]	Area %
1	1.778	BB	0.0259	1441.01038	844.63019	100.0000
Totals :				1441.01038	844.63019	

Compound 11

Method Info : ACE Phenyl, 50x3mm, 3μ,10-97% MeCN, 3min; 1ml/min, A: 0.1% TFA, B:MeCN.

Sample Info : Walkup method: 'Ph1097-3'
Target:

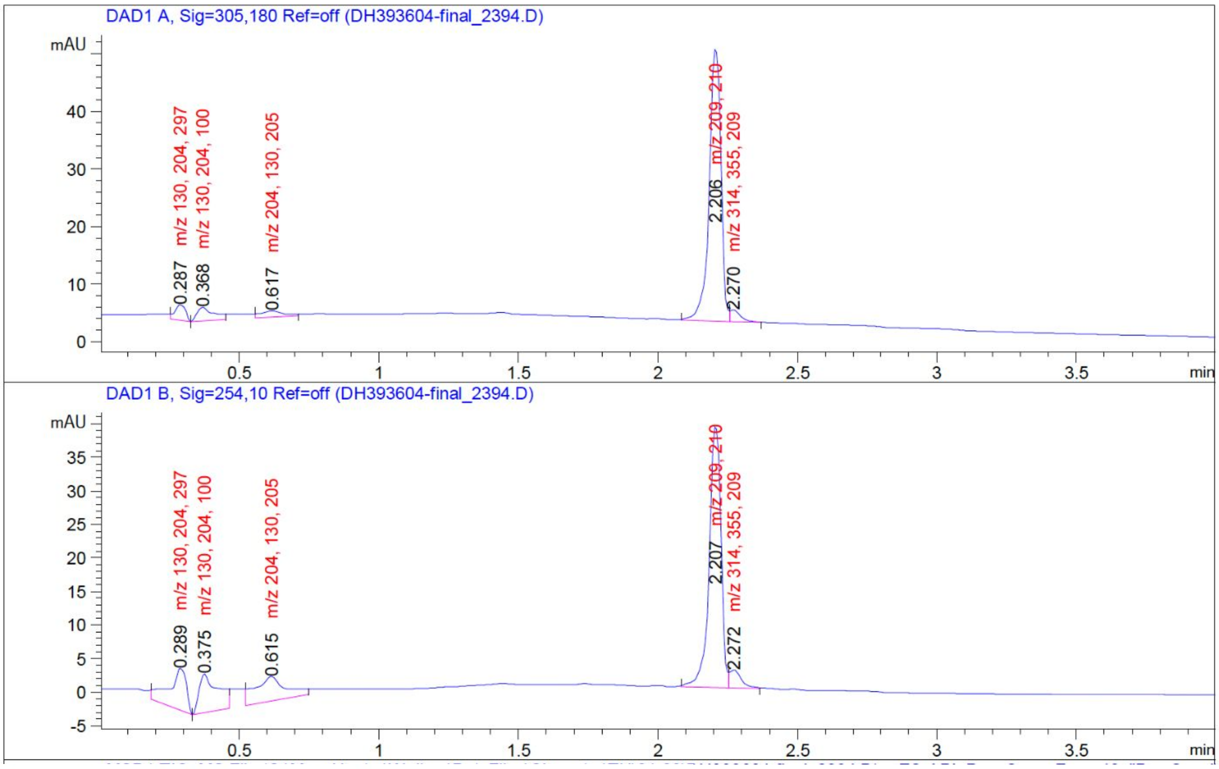

Signal 1: DAD1 A, Sig=305,180 Ref=off

Peak #	RetTime [min]	Type	Width [min]	Area [mAU*s]	Height [mAU]	Area %
1	0.287	BV	0.0424	6.96528	2.66902	4.2031
2	0.368	VB	0.0584	9.72721	2.34337	5.8698
3	0.617	BB	0.0783	6.03885	1.09638	3.6441
4	2.206	BV	0.0459	137.89867	47.49380	83.2138
5	2.270	VB	0.0399	5.08608	1.98490	3.0692

Totals : 165.71608 55.58748

Signal 2: DAD1 B, Sig=254,10 Ref=off

Peak #	RetTime [min]	Type	Width [min]	Area [mAU*s]	Height [mAU]	Area %
1	0.289	BV	0.0625	26.71750	6.17302	13.0969
2	0.375	VB	0.0642	26.84147	5.77319	13.1577
3	0.615	BB	0.1023	27.93004	3.60885	13.6913
4	2.207	BV	0.0462	114.57673	39.09018	56.1655
5	2.272	VB	0.0444	7.93262	2.69296	3.8886

Totals : 203.99836 57.33821

Compound 12

Method Info : ACE Phenyl, 50x3mm, 3μ,10-97% MeCN, 3min; 1ml/min, A: 0.1% TFA, B:MeCN.

Sample Info : Walkup method: 'Ph1097-3'
Target:

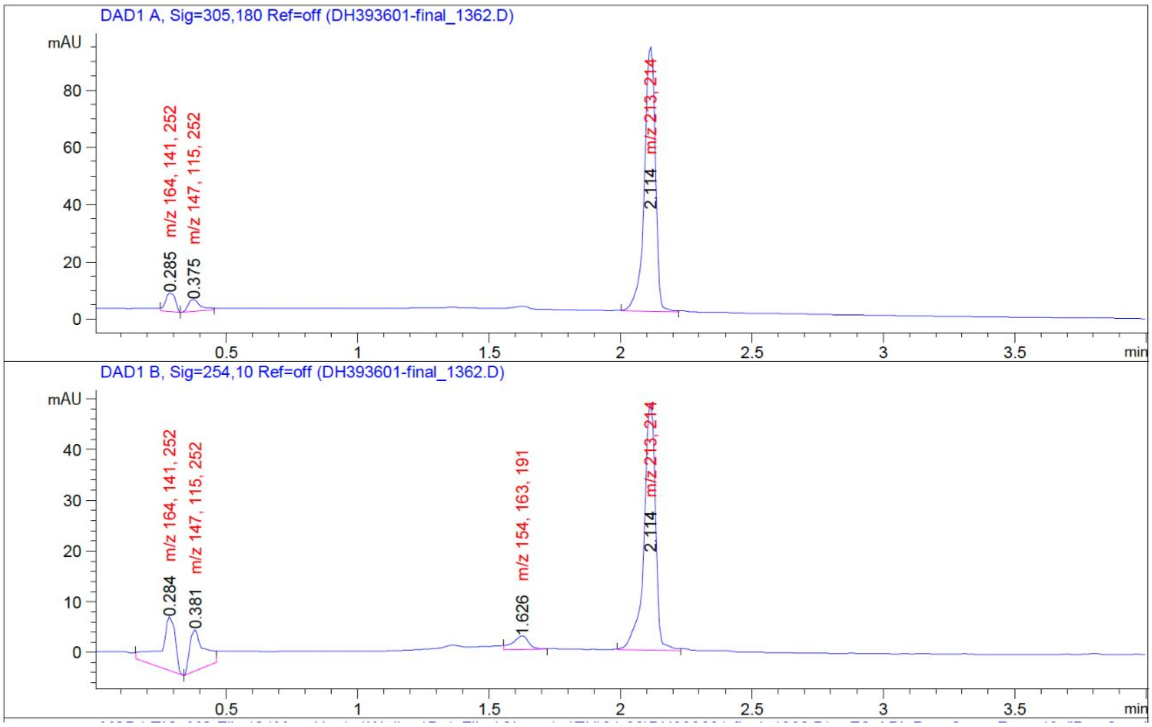

Signal 1: DAD1 A, Sig=305,180 Ref=off

Peak #	RetTime [min]	Type	Width [min]	Area [mAU*s]	Height [mAU]	Area %
1	0.285	BV	0.0413	16.44697	6.55455	5.4632
2	0.375	VB	0.0511	13.85804	4.13718	4.6033
3	2.114	BB	0.0461	270.74255	92.67313	89.9335

Totals : 301.04757 103.36486

Signal 2: DAD1 B, Sig=254,10 Ref=off

Peak #	RetTime [min]	Type	Width [min]	Area [mAU*s]	Height [mAU]	Area %
1	0.284	BV	0.0605	42.26677	10.61275	18.0424
2	0.381	VB	0.0536	30.56600	8.17175	13.0477
3	1.626	BB	0.0634	11.48133	2.71078	4.9010
4	2.114	BB	0.0479	149.94983	48.74969	64.0089

Compound 13

```
Method Info      : X-bridge
                   10-97% MeCN-3min, ACE C8, 50x3.0 mm, 3u, 1ml/min, 215-395 & 254 nm,C:
                   NH4HCO3 10mM, B: MeCN

Sample Info      : Walkup method: 'X1097-3'
                   Target:
```

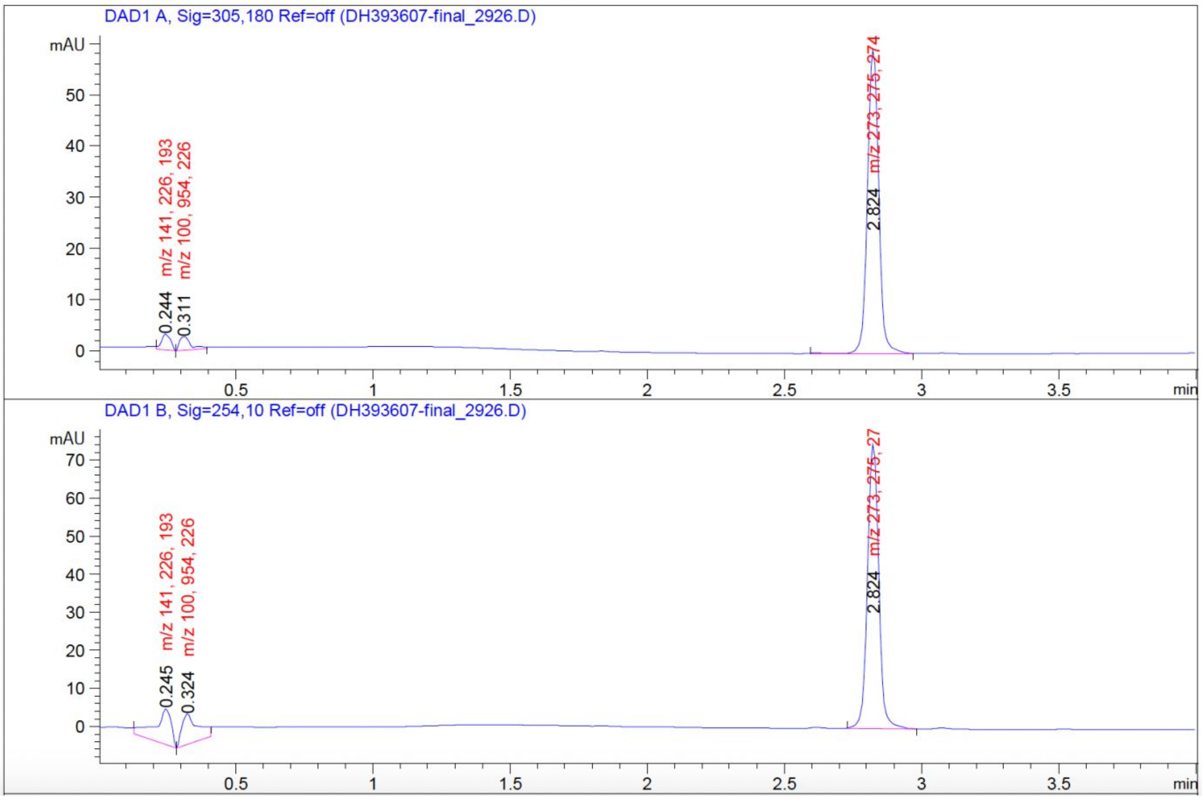

Signal 1: DAD1 A, Sig=305,180 Ref=off

Peak #	RetTime [min]	Type	Width [min]	Area [mAU*s]	Height [mAU]	Area %
1	0.244	BV	0.0325	7.12731	3.13492	3.8551
2	0.311	VB	0.0461	7.70558	2.63291	4.1679
3	2.824	BB	0.0455	170.04674	59.13942	91.9770
Totals :				184.87963	64.90725	

Signal 2: DAD1 B, Sig=254,10 Ref=off

Peak #	RetTime [min]	Type	Width [min]	Area [mAU*s]	Height [mAU]	Area %
1	0.245	BV	0.0596	38.37722	9.40057	13.3935
2	0.324	VB	0.0587	32.42729	8.10413	11.3170
3	2.824	BB	0.0457	215.73215	74.62402	75.2895
Totals :				286.53666	92.12872	

Compound 14

Method Info : X-bridge
10-97% MeCN-3min, ACE C8, 50x3.0 mm, 3u, 1ml/min, 215-395 & 254 nm,C: NH4HCO3 10mM, B: MeCN

Sample Info : Walkup method: 'X1097-3'
Target:

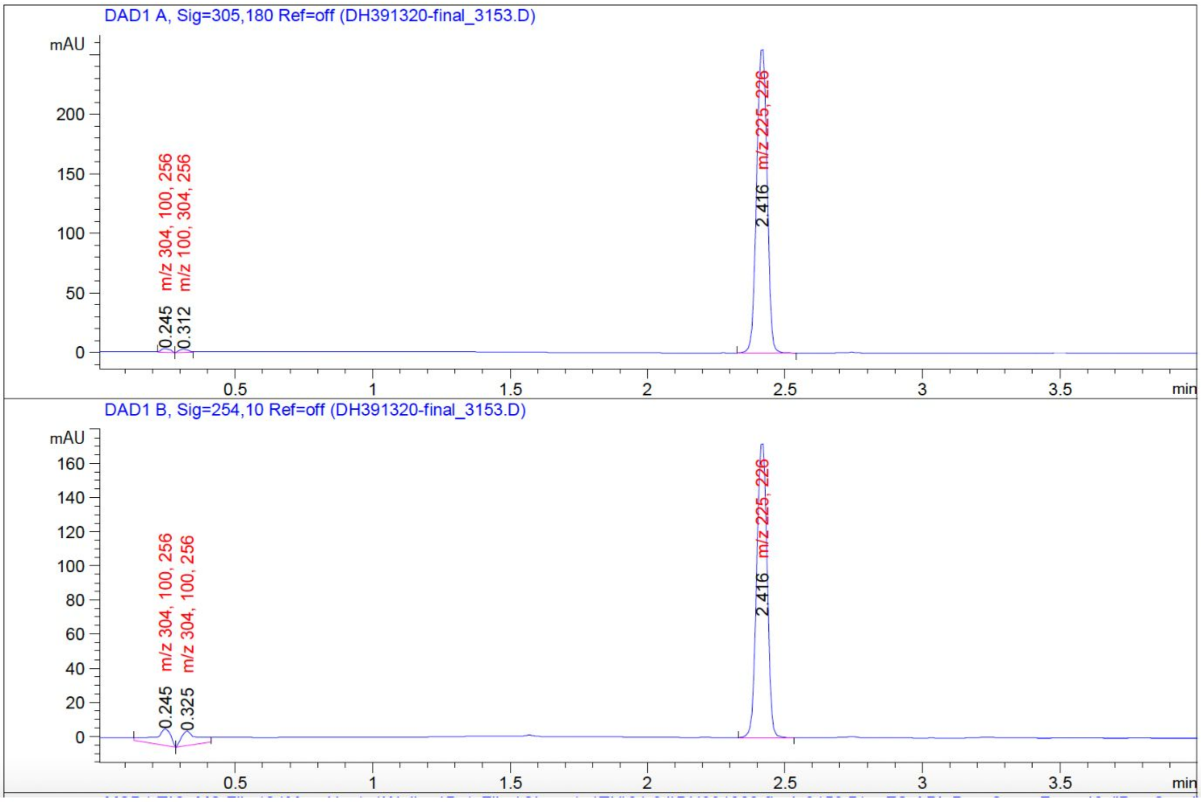

Signal 1: DAD1 A, Sig=305,180 Ref=off

Peak #	RetTime [min]	Type	Width [min]	Area [mAU*s]	Height [mAU]	Area %
1	0.245	BV	0.0321	7.41221	3.31402	1.0663
2	0.312	VV	0.0405	6.68819	2.73832	0.9621
3	2.416	BB	0.0428	681.04382	257.71622	97.9716

Totals : 695.14422 263.76856

Signal 2: DAD1 B, Sig=254,10 Ref=off

Peak #	RetTime [min]	Type	Width [min]	Area [mAU*s]	Height [mAU]	Area %
1	0.245	BV	0.0591	39.29856	9.73271	7.3585
2	0.325	VB	0.0591	34.43008	8.52735	6.4469
3	2.416	BB	0.0428	460.33035	174.26717	86.1947

Compound 16

Method Info : ACE Phenyl, 50x3mm, 3µ,10-97% MeCN, 3min; 1ml/min, A: 0.1% TFA, B:MeCN.

Sample Info : Walkup method: 'Ph1097-3'
Target:

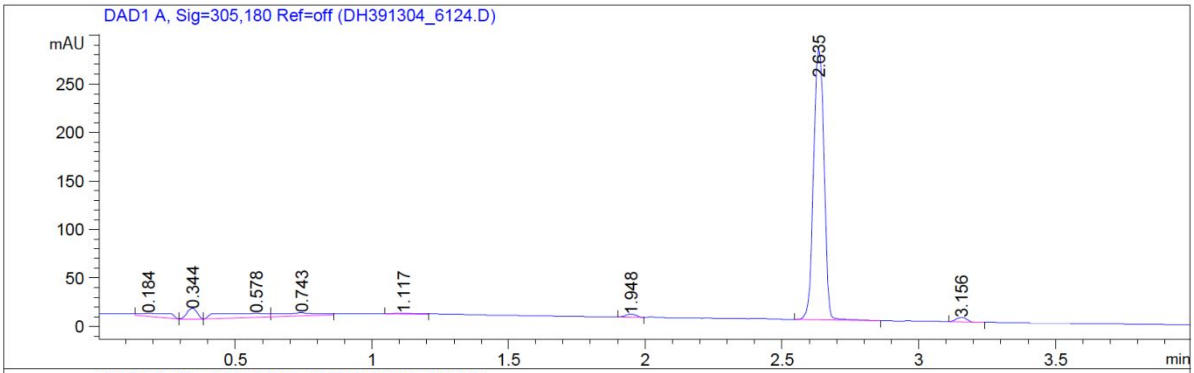

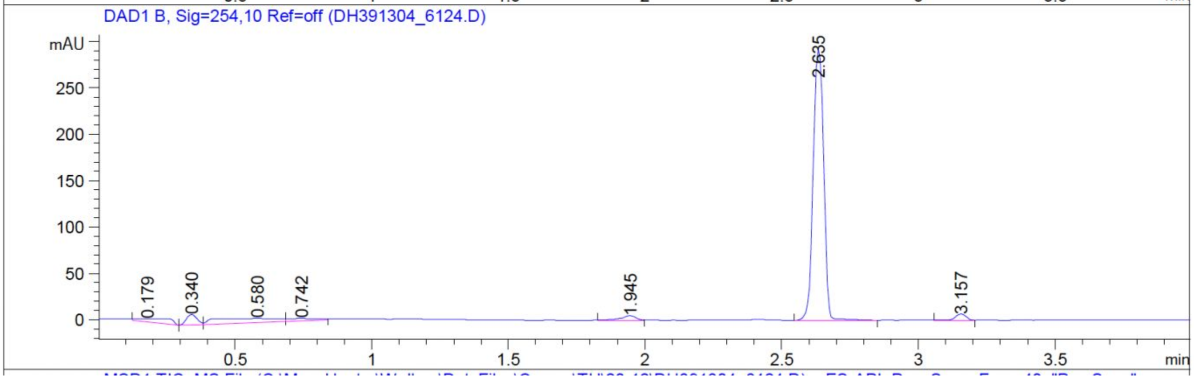

Signal 1: DAD1 A, Sig=305,180 Ref=off

Peak #	RetTime [min]	Type	Width [min]	Area [mAU*s]	Height [mAU]	Area %
1	0.184	BV	0.1202	29.12253	3.08451	3.1474
2	0.344	VV	0.0422	28.15196	10.88069	3.0425
3	0.578	VV	0.1896	61.34666	4.02969	6.6301
4	0.743	VB	0.1383	32.55095	3.00617	3.5180
5	1.117	BB	0.0762	6.25343	1.13578	0.6758
6	1.948	BV	0.0441	8.94260	3.24529	0.9665
7	2.635	VB	0.0431	746.24512	280.29010	80.6509
8	3.156	BB	0.0431	12.66475	4.75393	1.3688

Totals : 925.27801 310.42614

Signal 2: DAD1 B, Sig=254,10 Ref=off

Peak #	RetTime [min]	Type	Width [min]	Area [mAU*s]	Height [mAU]	Area %
1	0.179	BV	0.1347	36.47401	3.46893	3.7167
2	0.340	VV	0.0437	30.18376	11.10066	3.0757
3	0.580	VV	0.2131	77.42226	4.49260	7.8893
4	0.742	VB	0.0985	19.84925	2.74158	2.0226
5	1.945	BV	0.0593	19.25263	4.95961	1.9618
6	2.635	VV	0.0430	779.21570	293.09503	79.4022
7	3.157	VV	0.0454	18.95466	6.61550	1.9315

Totals : 981.35228 326.47392

Compound 17

Method Info : X-bridge
10-97% MeCN-3min, ACE C8, 50x3.0 mm, 3u, 1ml/min, 215-395 & 254 nm,C:
NH4HCO3 10mM, B: MeCN

Sample Info : Walkup method: 'X1097-3'
Target:

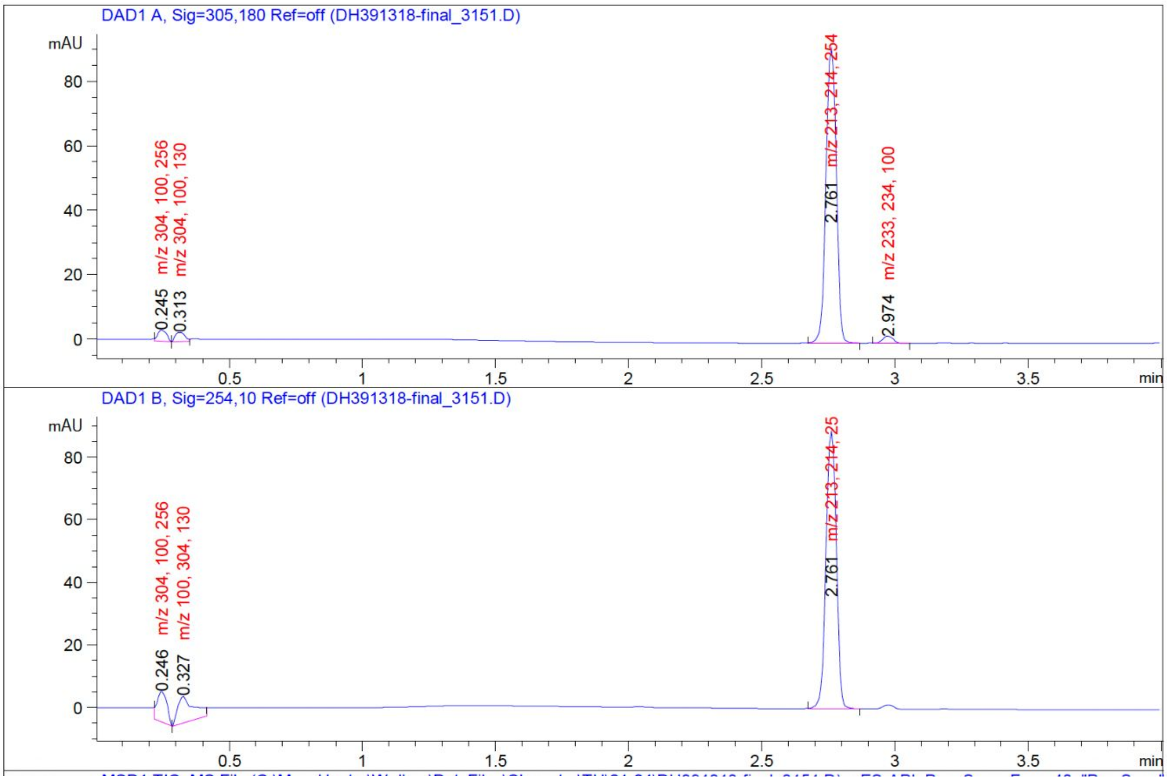

Signal 1: DAD1 A, Sig=305,180 Ref=off

Peak #	RetTime [min]	Type	Width [min]	Area [mAU*s]	Height [mAU]	Area %
1	0.245	BV	0.0326	8.08826	3.55292	3.0274
2	0.313	VV	0.0414	7.27388	2.88241	2.7225
3	2.761	BB	0.0454	245.80779	91.42844	92.0035
4	2.974	BB	0.0431	6.00240	2.25223	2.2466

Totals : 267.17233 100.11599

Signal 2: DAD1 B, Sig=254,10 Ref=off

Peak #	RetTime [min]	Type	Width [min]	Area [mAU*s]	Height [mAU]	Area %
1	0.246	BV	0.0414	24.58535	9.74640	8.2543
2	0.327	VB	0.0599	35.21101	8.57728	11.8218
3	2.761	BB	0.0454	238.05255	88.52896	79.9239

Totals : 297.84891 106.85264

Compound 18

Method Info : ACE Phenyl, 50x3mm, 3μ,10-97% MeCN, 3min; 1ml/min, A: 0.1% TFA, B:MeCN.

Sample Info : Walkup method: 'Ph1097-3'
Target:

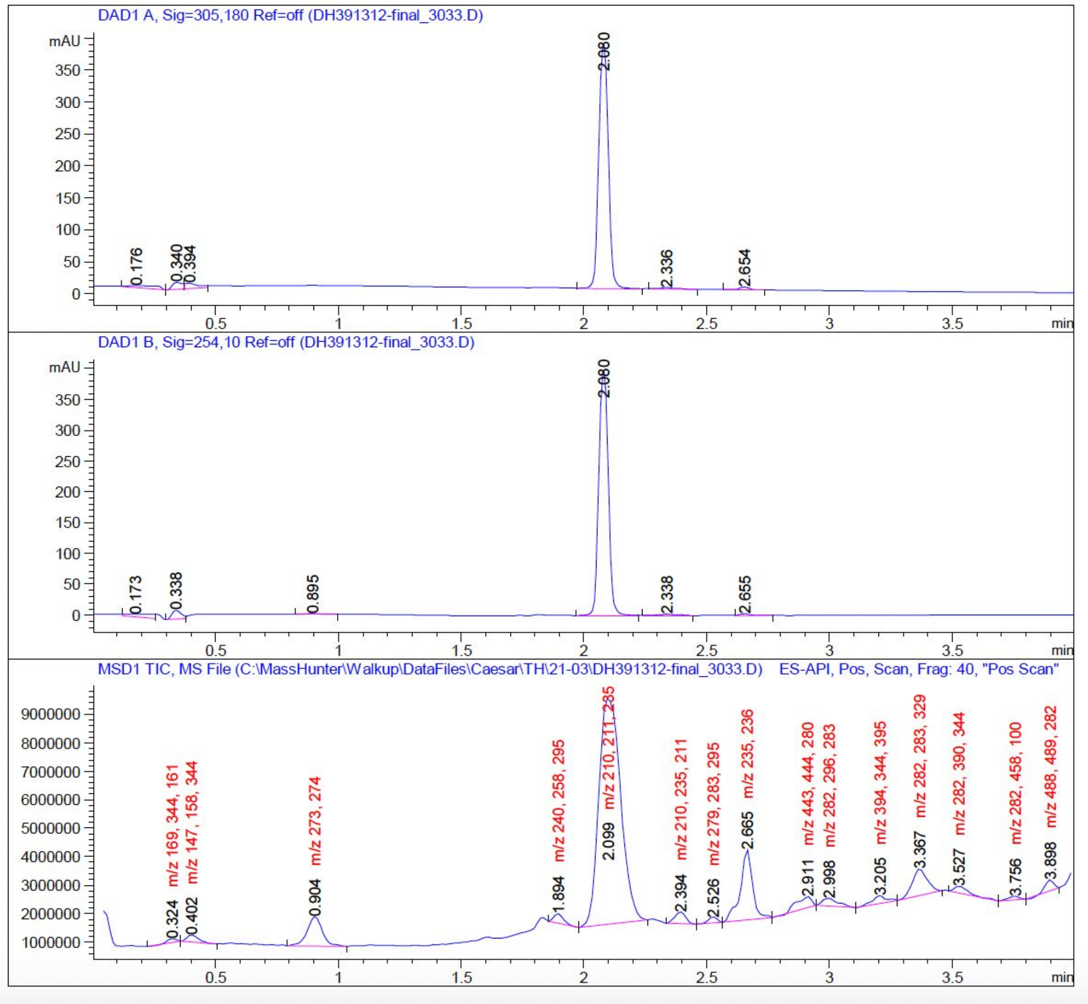

Signal 1: DAD1 A, Sig=305,180 Ref=off

Peak #	RetTime [min]	Type	Width [min]	Area [mAU*s]	Height [mAU]	Area %
1	0.176	BV	0.1366	37.99562	3.55876	3.2972
2	0.340	VV	0.0427	30.67876	10.94223	2.6622
3	0.394	VB	0.0495	30.71274	8.24038	2.6652
4	2.080	VB	0.0433	1027.73584	383.61264	89.1841
5	2.336	BB	0.0712	11.89410	2.26759	1.0321
6	2.654	BB	0.0490	13.35893	4.20801	1.1593

Totals : 1152.37599 412.82961

Signal 2: DAD1 B, Sig=254,10 Ref=off

Peak #	RetTime [min]	Type	Width [min]	Area [mAU*s]	Height [mAU]	Area %
1	0.173	BB	0.1115	36.79247	4.31612	3.1496
2	0.338	BV	0.0432	38.62285	14.42277	3.3063
3	0.895	BB	0.0552	5.73535	1.62311	0.4910
4	2.080	VB	0.0432	1065.62305	398.15375	91.2225
5	2.338	BV	0.0741	13.39012	2.43819	1.1463
6	2.655	VB	0.0462	7.99383	2.72727	0.6843

Totals : 1168.15766 423.68121

Compound 19

Method Info : ACE Phenyl, 50x3mm, 3µ,10-97% MeCN, 3min; 1ml/min, A: 0.1% TFA, B:MeCN.

Sample Info : Walkup method: 'Ph1097-3'
Target:

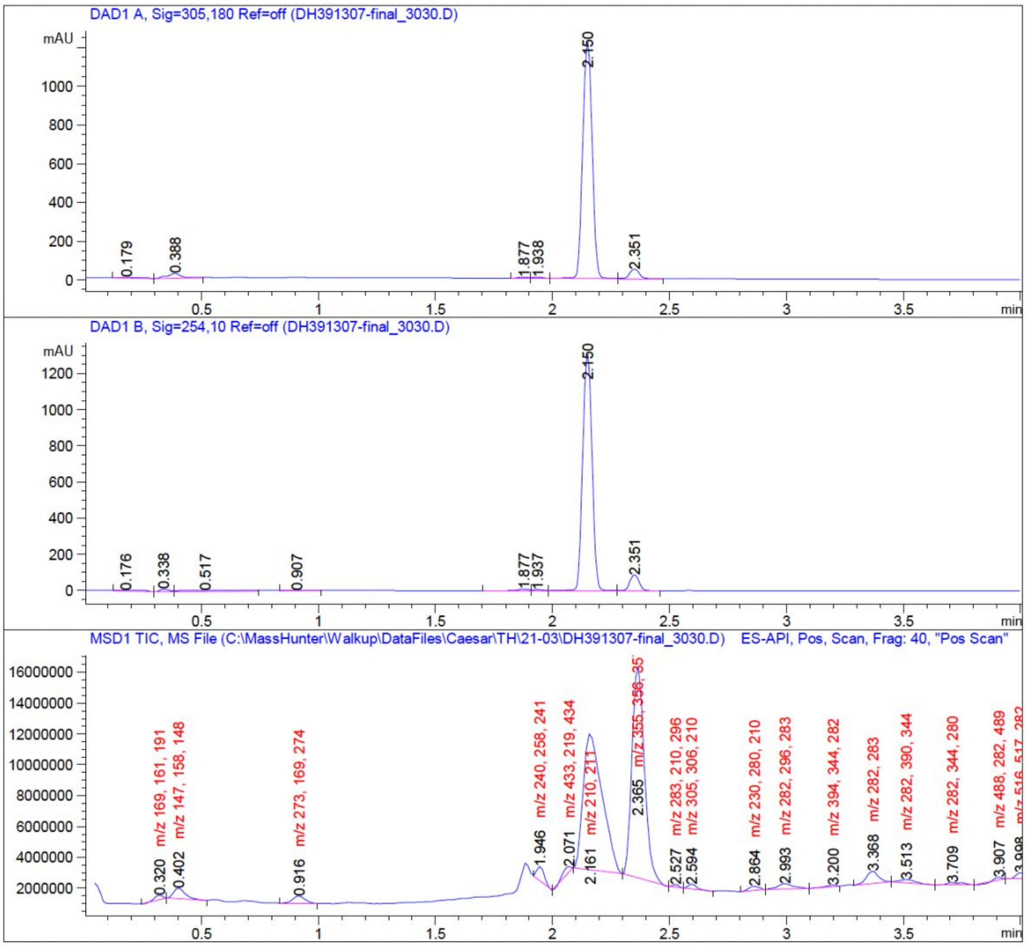

Signal 1: DAD1 A, Sig=305,180 Ref=off

Peak #	RetTime [min]	Type	Width [min]	Area [mAU*s]	Height [mAU]	Area %
1	0.179	BV	0.1350	33.68972	3.19530	0.9066
2	0.388	VB	0.0653	117.12455	25.61568	3.1518
3	1.877	BV	0.0447	14.91725	5.32977	0.4014
4	1.938	VV	0.0450	16.96758	6.00235	0.4566
5	2.150	VV	0.0444	3397.01733	1223.23975	91.4138
6	2.351	VB	0.0446	136.37157	48.74049	3.6698

Totals : 3716.08799 1312.12334

Signal 2: DAD1 B, Sig=254,10 Ref=off

Peak #	RetTime [min]	Type	Width [min]	Area [mAU*s]	Height [mAU]	Area %
1	0.176	BV	0.1379	43.22313	4.00482	1.0457
2	0.338	VV	0.0453	40.47015	14.16477	0.9791
3	0.517	VB	0.2378	114.51796	5.91632	2.7706
4	0.907	BB	0.1094	20.93940	2.56057	0.5066
5	1.877	VV	0.0496	34.18800	10.61545	0.8271
6	1.937	VV	0.0417	20.57878	7.56749	0.4979
7	2.150	VV	0.0441	3618.46631	1313.71667	87.5423
8	2.351	VB	0.0441	241.00584	87.56169	5.8307

Totals : 4133.38957 1446.10777

Compound 20

Method Info : ACE Phenyl, 50x3mm, 3µ,10-97% MeCN, 3min; 1ml/min, A: 0.1% TFA, B:MeCN.

Sample Info : Walkup method: 'Ph1097-3'
Target:

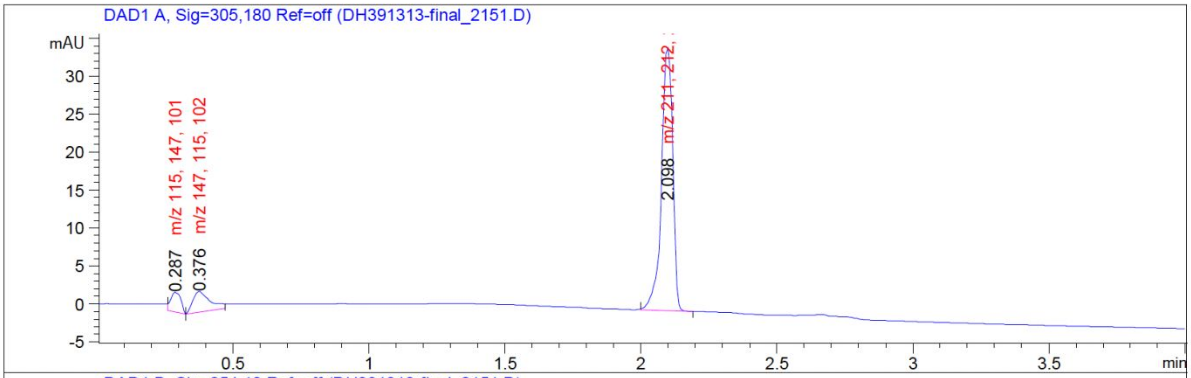

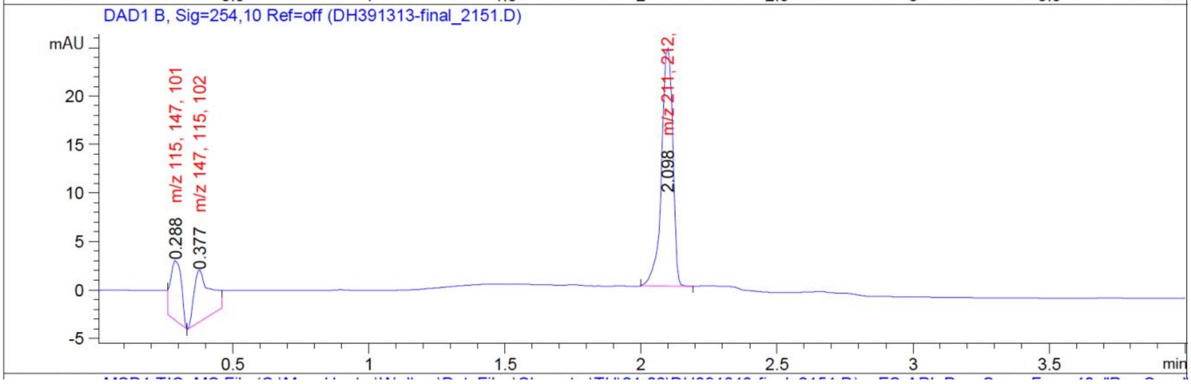

Signal 1: DAD1 A, Sig=305,180 Ref=off

Peak #	RetTime [min]	Type	Width [min]	Area [mAU*s]	Height [mAU]	Area %
1	0.287	BV	0.0412	6.56382	2.62572	5.5746
2	0.376	VB	0.0618	11.66640	2.73075	9.9081
3	2.098	BB	0.0475	99.51537	34.68898	84.5173
Totals :				117.74558	40.04545	

Signal 2: DAD1 B, Sig=254,10 Ref=off

Peak #	RetTime [min]	Type	Width [min]	Area [mAU*s]	Height [mAU]	Area %
1	0.288	BV	0.0439	16.93386	6.19249	15.4954
2	0.377	VB	0.0572	21.93119	5.42243	20.0682
3	2.098	BB	0.0474	70.41835	24.58055	64.4365
Totals :				109.28340	36.19546	

Compound 21

Method Info : ACE Phenyl, 50x3mm, 3μ,10-97% MeCN, 3min; 1ml/min, A: 0.1% TFA, B:MeCN.

Sample Info : Walkup method: 'Ph1097-3'
Target:

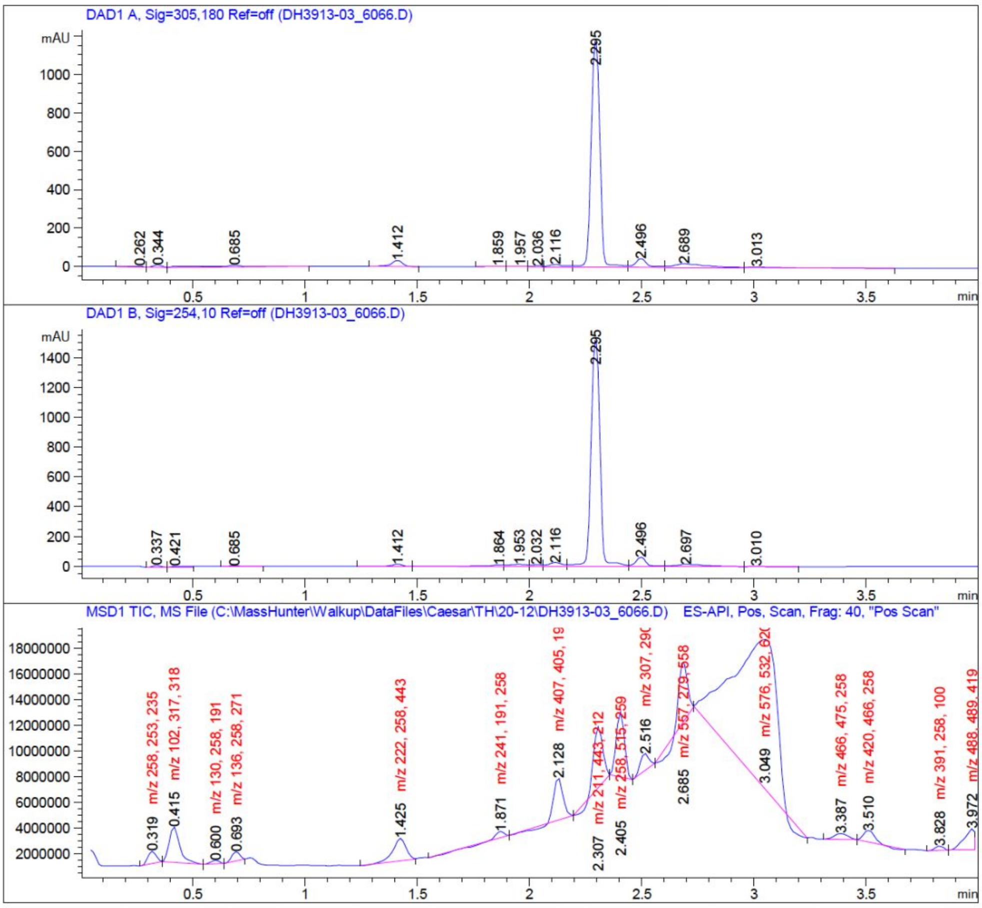

Signal 1: DAD1 A, Sig=305,180 Ref=off

Peak #	RetTime [min]	Type	Width [min]	Area [mAU*s]	Height [mAU]	Area %
1	0.262	BV	0.0649	21.00848	4.15860	0.5354
2	0.344	VV	0.0435	30.90947	11.46229	0.7878
3	0.685	VB	0.2004	136.00417	8.42385	3.4663
4	1.412	BB	0.0545	107.50926	30.95361	2.7401
5	1.859	BV	0.0654	16.19926	3.53968	0.4129
6	1.957	VV	0.0717	20.59014	4.02451	0.5248
7	2.036	VV	0.0603	19.15570	4.62624	0.4882
8	2.116	VV	0.0671	58.71376	12.42372	1.4964
9	2.295	VV	0.0430	3144.02759	1182.41333	80.1317
10	2.496	VV	0.0546	160.41209	43.90444	4.0884
11	2.689	VV	0.1188	186.72516	20.40225	4.7591
12	3.013	VB	0.0781	22.32187	3.93858	0.5689

Totals : 3923.57695 1330.27112

Signal 2: DAD1 B, Sig=254,10 Ref=off

Peak #	RetTime [min]	Type	Width [min]	Area [mAU*s]	Height [mAU]	Area %
1	0.337	BV	0.0436	31.18483	11.49594	0.6189
2	0.421	VB	0.0787	28.11426	4.77464	0.5580
3	0.685	BB	0.0738	34.75055	6.55853	0.6897
4	1.412	BV	0.0557	60.31281	16.87309	1.1970
5	1.864	VV	0.0997	78.64321	10.46486	1.5608
6	1.953	VV	0.0814	87.93873	15.21492	1.7453
7	2.032	VV	0.0503	45.07929	12.42085	0.8947
8	2.116	VV	0.0625	118.27374	27.30942	2.3473
9	2.295	VV	0.0437	4155.71582	1530.45703	82.4769
10	2.496	VV	0.0520	214.15002	62.47823	4.2502
11	2.697	VV	0.1177	167.33771	18.46362	3.3211
12	3.010	VB	0.0822	17.13993	2.93297	0.3402

Totals : 5038.64090 1719.44409

Compound 22

Method Info : ACE Phenyl, 50x3mm, 3μ,10-97% MeCN, 3min; 1ml/min, A: 0.1% TFA, B:MeCN.

Sample Info : Walkup method: 'Ph1097-3'
Target:

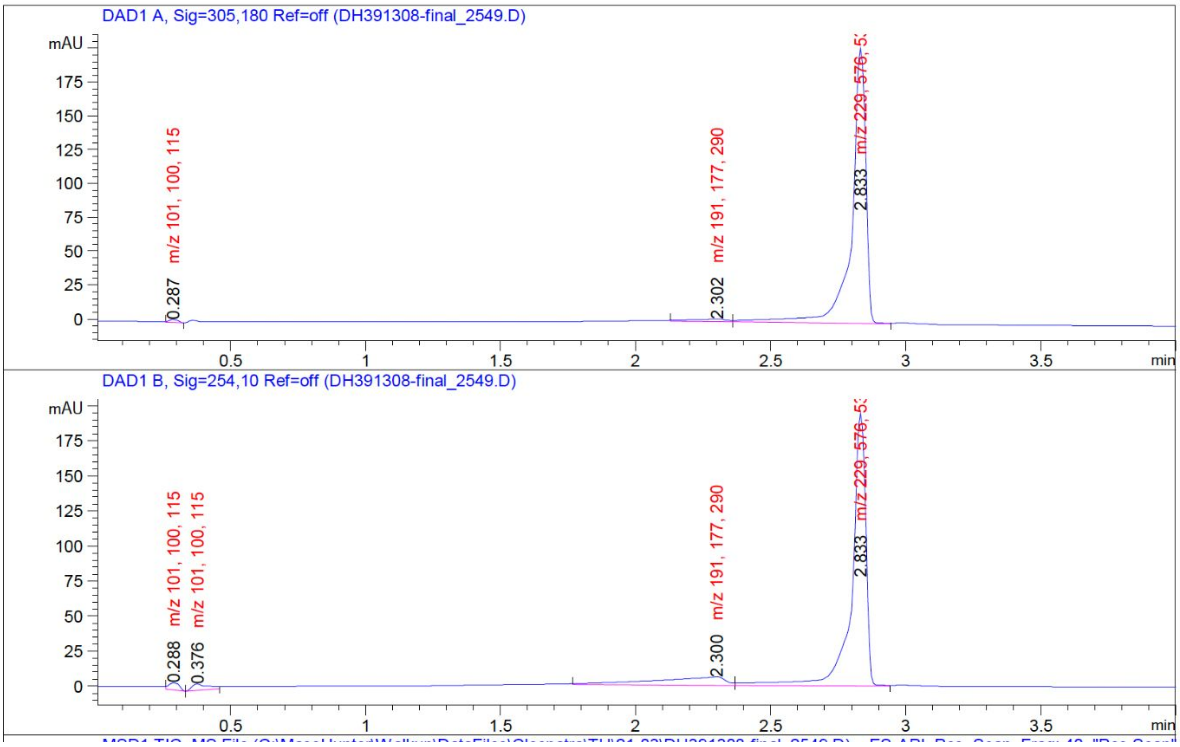

Signal 1: DAD1 A, Sig=305,180 Ref=off

Peak #	RetTime [min]	Type	Width [min]	Area [mAU*s]	Height [mAU]	Area %
1	0.287	BV	0.0352	5.58633	2.23408	0.7300
2	2.302	BV	0.1162	17.60665	1.97113	2.3007
3	2.833	VV	0.0546	742.08850	203.18533	96.9694

Totals : 765.28148 207.39055

Signal 2: DAD1 B, Sig=254,10 Ref=off

Peak #	RetTime [min]	Type	Width [min]	Area [mAU*s]	Height [mAU]	Area %
1	0.288	BV	0.0463	14.18001	5.11902	1.6675
2	0.376	VB	0.0590	18.62401	4.62217	2.1901
3	2.300	BV	0.2166	108.37270	6.17886	12.7439
4	2.833	VV	0.0546	709.21484	194.45630	83.3986

Totals : 850.39156 210.37635

Compound 23

```
Method Info      : ACE Phenyl, 50x3mm, 3μ,10-97% MeCN, 3min; 1ml/min, A: 0.1% TFA, B:MeCN.

Sample Info      : Walkup method: 'Ph1097-3'
                   Target:
```

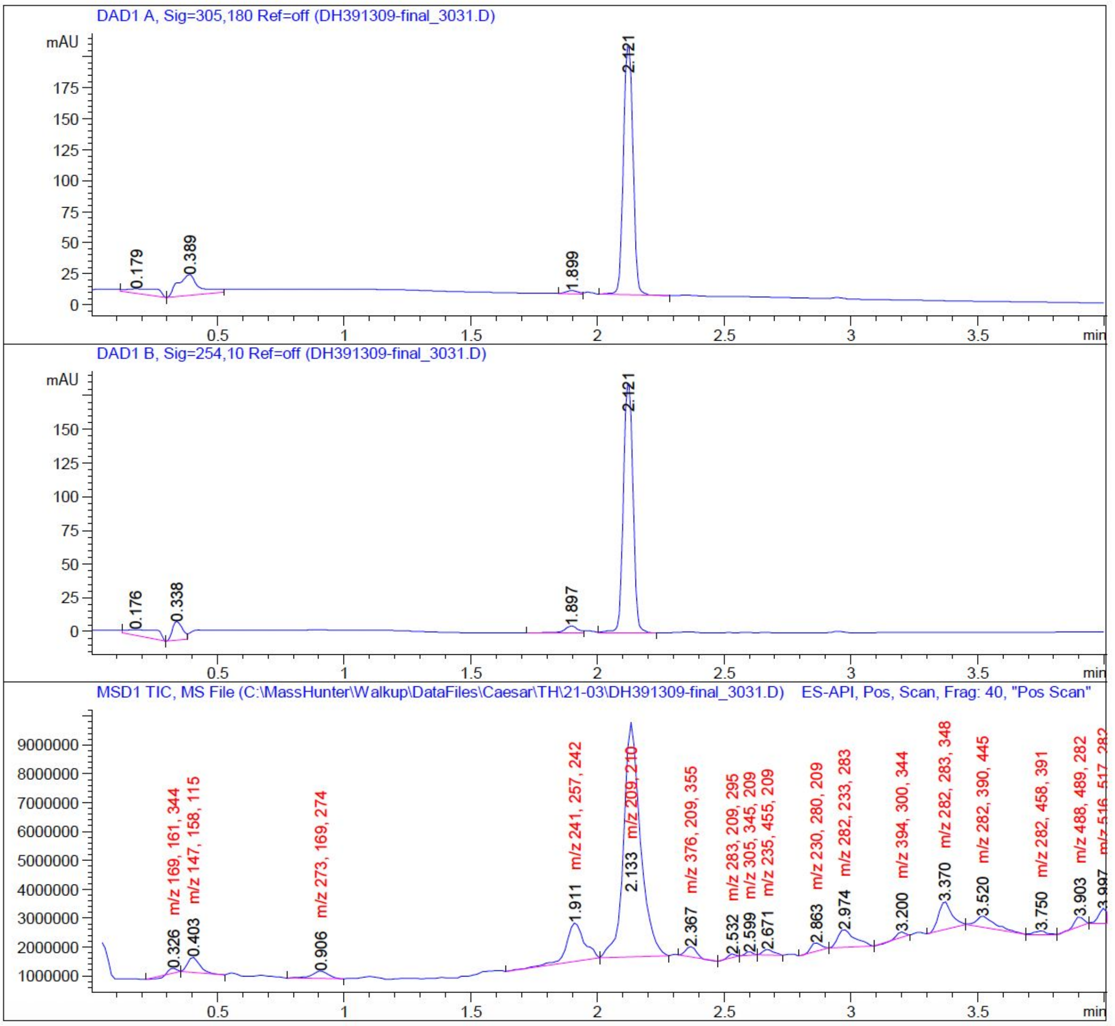

Signal 1: DAD1 A, Sig=305,180 Ref=off

Peak #	RetTime [min]	Type	Width [min]	Area [mAU*s]	Height [mAU]	Area %
1	0.179	BV	0.1344	36.57708	3.48736	5.4287
2	0.389	VB	0.0793	96.17725	16.67042	14.2745
3	1.899	BV	0.0522	9.00744	2.61346	1.3369
4	2.121	VB	0.0427	532.00903	202.45041	78.9599

Totals : 673.77081 225.22166

Signal 2: DAD1 B, Sig=254,10 Ref=off

Peak #	RetTime [min]	Type	Width [min]	Area [mAU*s]	Height [mAU]	Area %
1	0.176	BV	0.1375	45.20253	4.20248	7.6123
2	0.338	VV	0.0436	37.96859	13.99845	6.3941
3	1.897	BV	0.0568	19.16974	4.98955	3.2283
4	2.121	VB	0.0428	491.46957	185.96498	82.7654

Totals : 593.81043 209.15546

Compound 26

Method Info : X-bridge C18, 50x3.0 mm, 3.5u, 10-97% MeCN 3min,1ml/min, B: MeCN C: NH4HCO3

Sample Info : Walkup method: 'X1097-3'
Target:

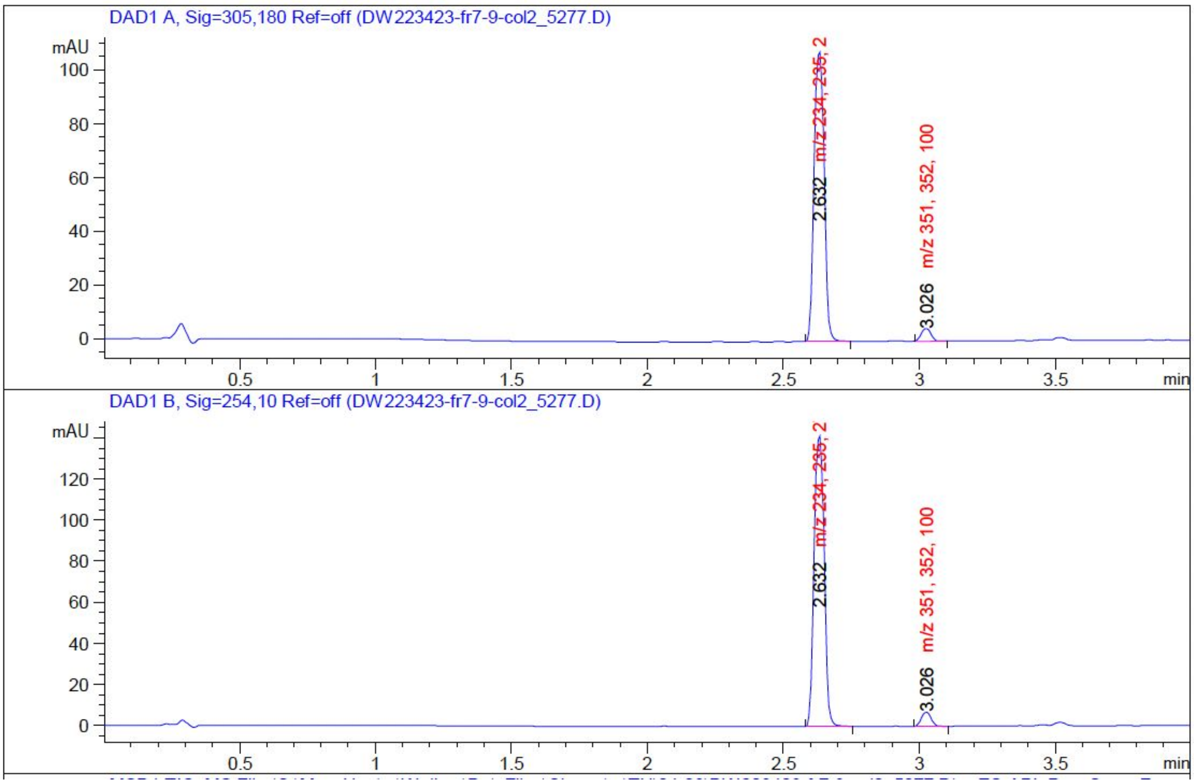

Signal 1: DAD1 A, Sig=305,180 Ref=off

Peak #	RetTime [min]	Type	Width [min]	Area [mAU*s]	Height [mAU]	Area %
1	2.632	BB	0.0416	275.81659	108.65607	95.8376
2	3.026	BB	0.0418	11.97932	4.69501	4.1624

Totals : 287.79591 113.35108

Signal 2: DAD1 B, Sig=254,10 Ref=off

Peak #	RetTime [min]	Type	Width [min]	Area [mAU*s]	Height [mAU]	Area %
1	2.632	BB	0.0416	362.12228	142.58075	95.3646
2	3.026	BB	0.0419	17.60165	6.87366	4.6354

Totals : 379.72394 149.45441

Compound 29

[illegible]

Method Info : X-bridge
10-97% MeCN-3min, ACE C8, 50x3.0 mm, 3u, 1ml/min, 215-395 & 254 nm,C: NH4HCO3 10mM, B: MeCN

Sample Info : Walkup method: 'X1097-3'
Target:

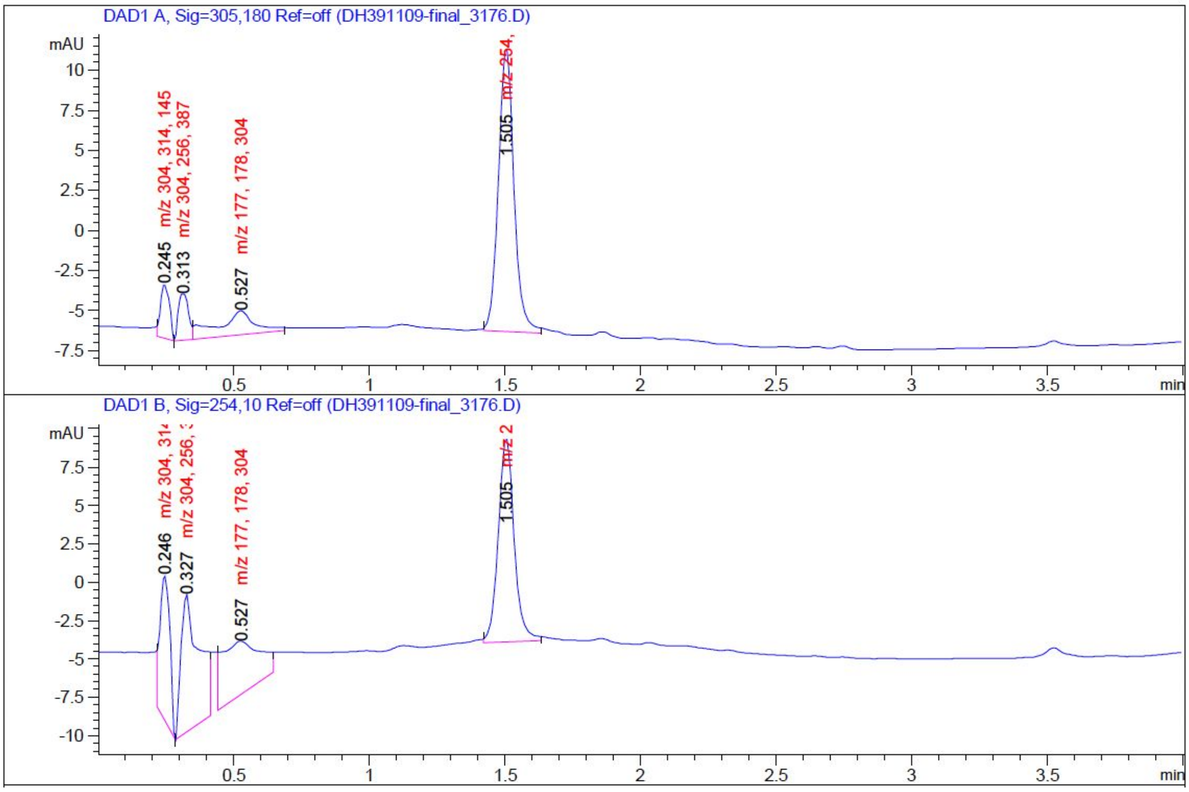

Signal 1: DAD1 A, Sig=305,180 Ref=off

Peak #	RetTime [min]	Type	Width [min]	Area [mAU*s]	Height [mAU]	Area %
1	0.245	BV	0.0324	7.64435	3.38382	7.6471
2	0.313	VV	0.0421	7.54561	2.92266	7.5483
3	0.527	VB	0.1247	14.25377	1.50218	14.2589
4	1.505	BB	0.0625	70.52007	17.70461	70.5456

Totals : 99.96380 25.51327

Signal 2: DAD1 B, Sig=254,10 Ref=off

Peak #	RetTime [min]	Type	Width [min]	Area [mAU*s]	Height [mAU]	Area %
1	0.246	BV	0.0414	23.76030	9.41421	15.5959
2	0.327	VB	0.0647	40.49784	8.97010	26.5822
3	0.527	BB	0.1280	34.14115	3.49276	22.4098
4	1.505	BB	0.0635	53.94992	13.26095	35.4120

Totals : 152.34922 35.13801

Compound 30

Method Info : X-bridge C18, 50x3.0 mm, 3.5u, 10-97% MeCN 3min,1ml/min, B: MeCN C: NH4HCO3

Sample Info : Walkup method: 'X1097-3'
Target:

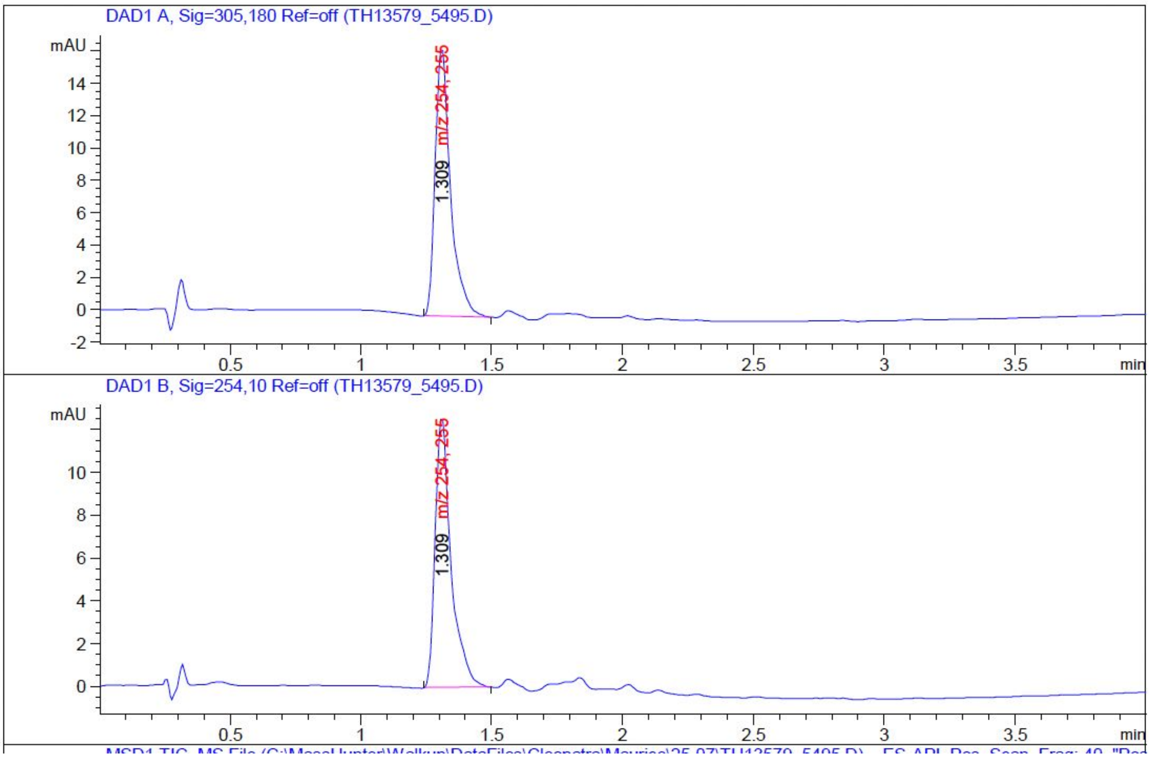

Signal 1: DAD1 A, Sig=305,180 Ref=off

Peak #	RetTime [min]	Type	Width [min]	Area [mAU*s]	Height [mAU]	Area %
1	1.309	BB	0.0614	67.49475	16.60168	100.0000

Totals : 67.49475 16.60168

Signal 2: DAD1 B, Sig=254,10 Ref=off

Peak #	RetTime [min]	Type	Width [min]	Area [mAU*s]	Height [mAU]	Area %
1	1.309	BB	0.0630	53.12283	12.65660	100.0000

Totals : 53.12283 12.65660

Compound 38

Method Info : X-bridge C18, 50x3.0 mm, 3.5u, 10-97% MeCN 3min,1ml/min, B: MeCN C: NH4HCO3

Sample Info : Walkup method: 'X1097-3'
Target:

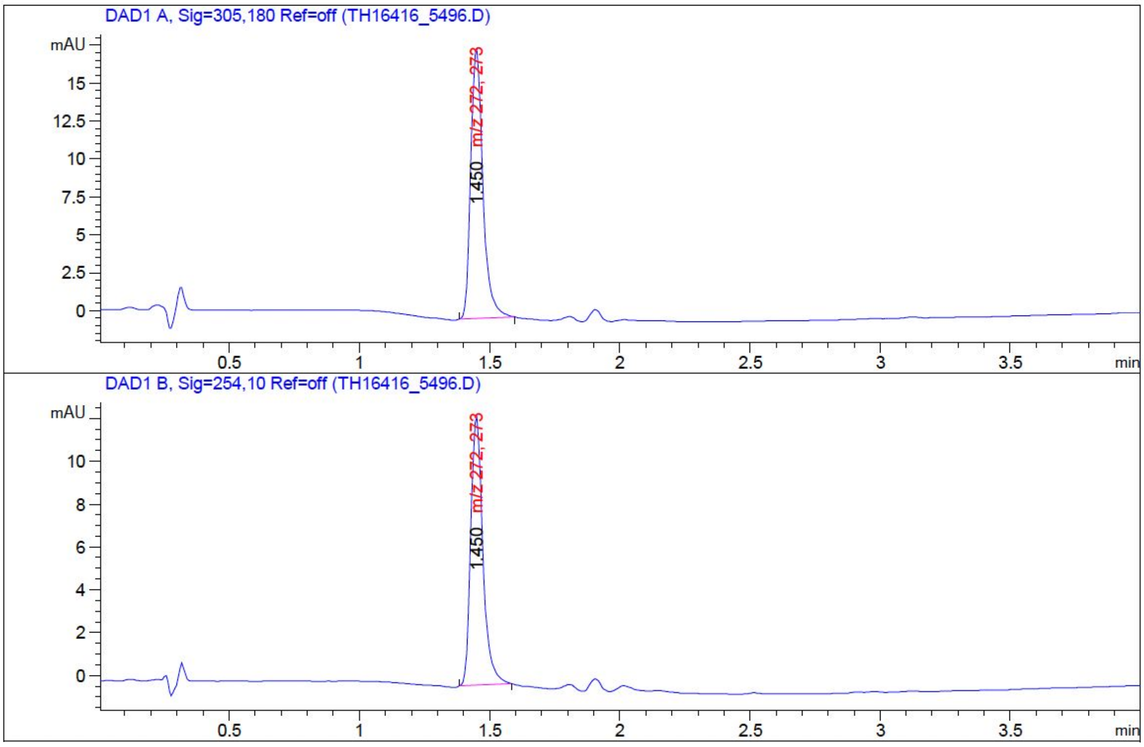

Signal 1: DAD1 A, Sig=305,180 Ref=off

Peak #	RetTime [min]	Type	Width [min]	Area [mAU*s]	Height [mAU]	Area %
1	1.450	BB	0.0499	57.89118	17.83881	100.0000
Totals :				57.89118	17.83881	

Signal 2: DAD1 B, Sig=254,10 Ref=off

Peak #	RetTime [min]	Type	Width [min]	Area [mAU*s]	Height [mAU]	Area %
1	1.450	BB	0.0497	40.70349	12.58718	100.0000
Totals :				40.70349	12.58718	

S7. NMR Spectra

Compound 1 ^{1}H

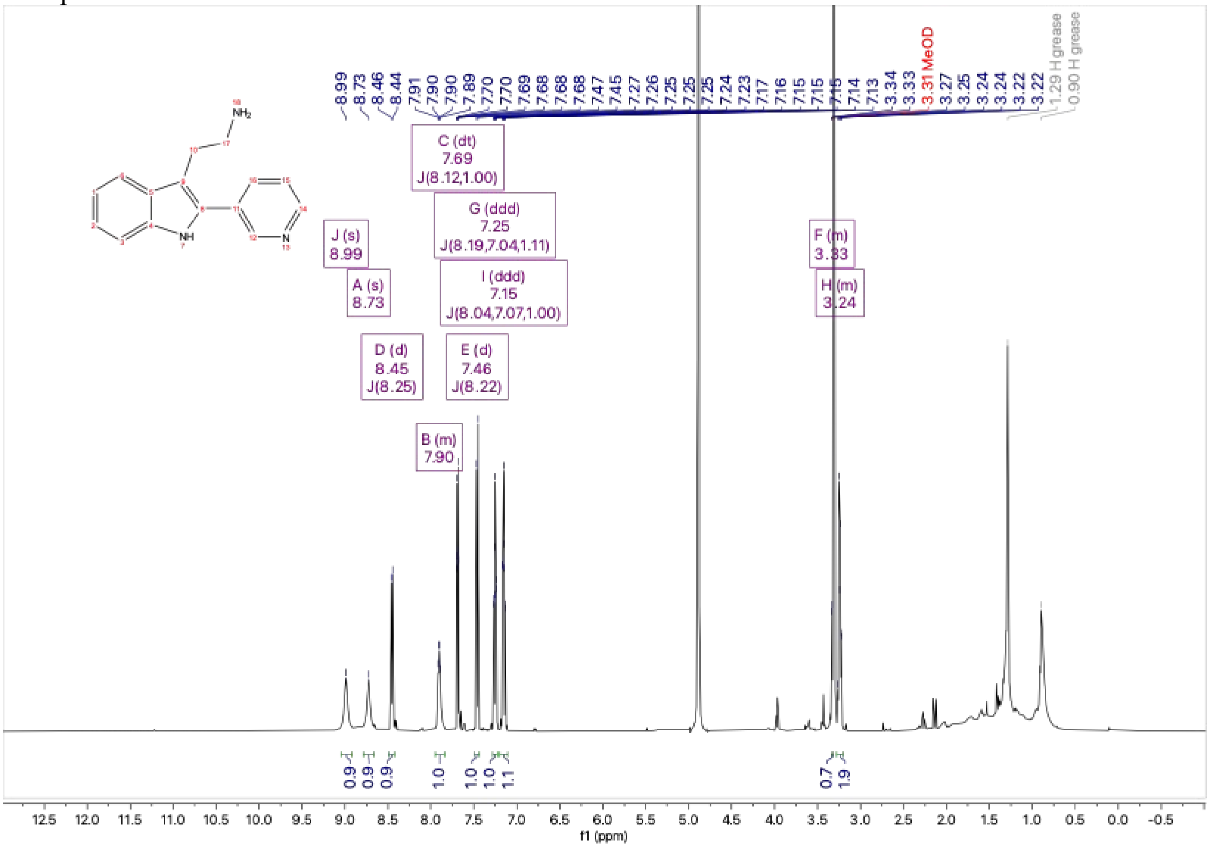

Compound 1 ^{13}C

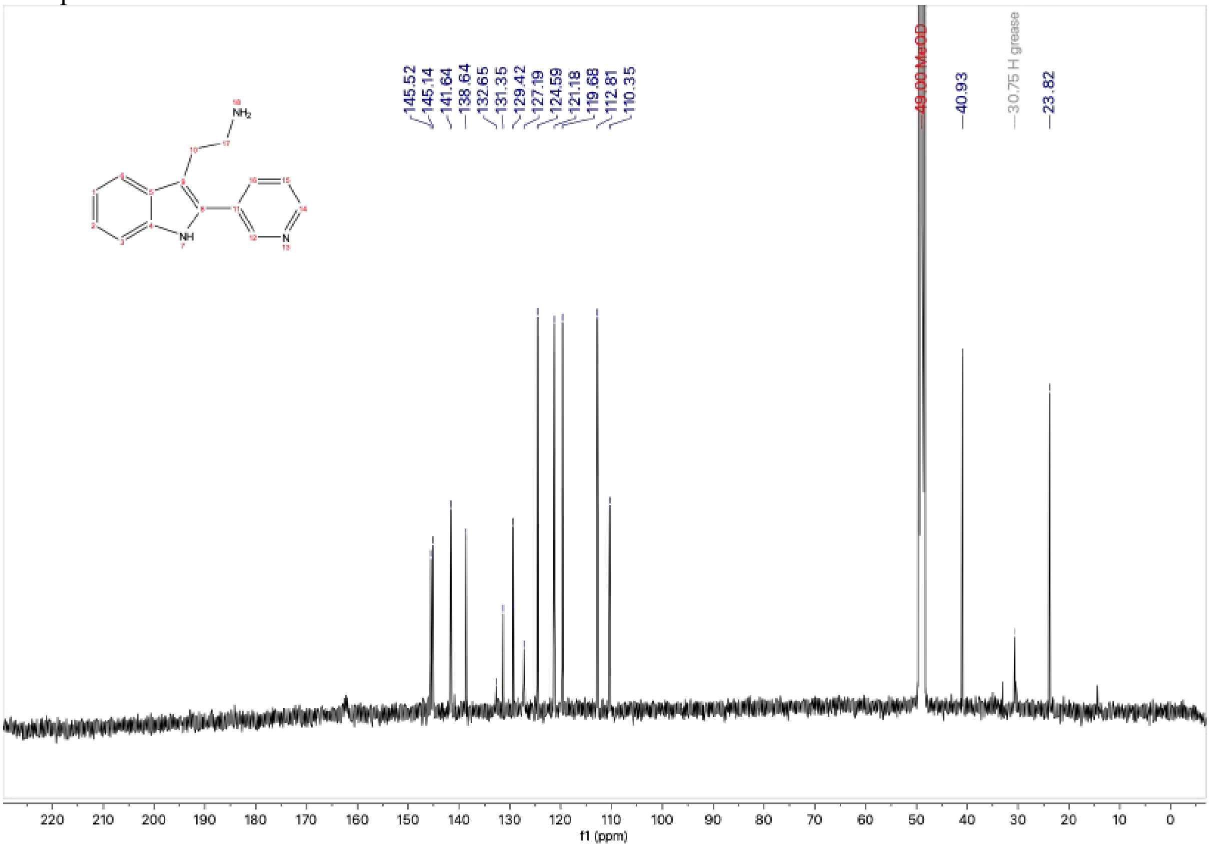

Compound 2 ^{1}H

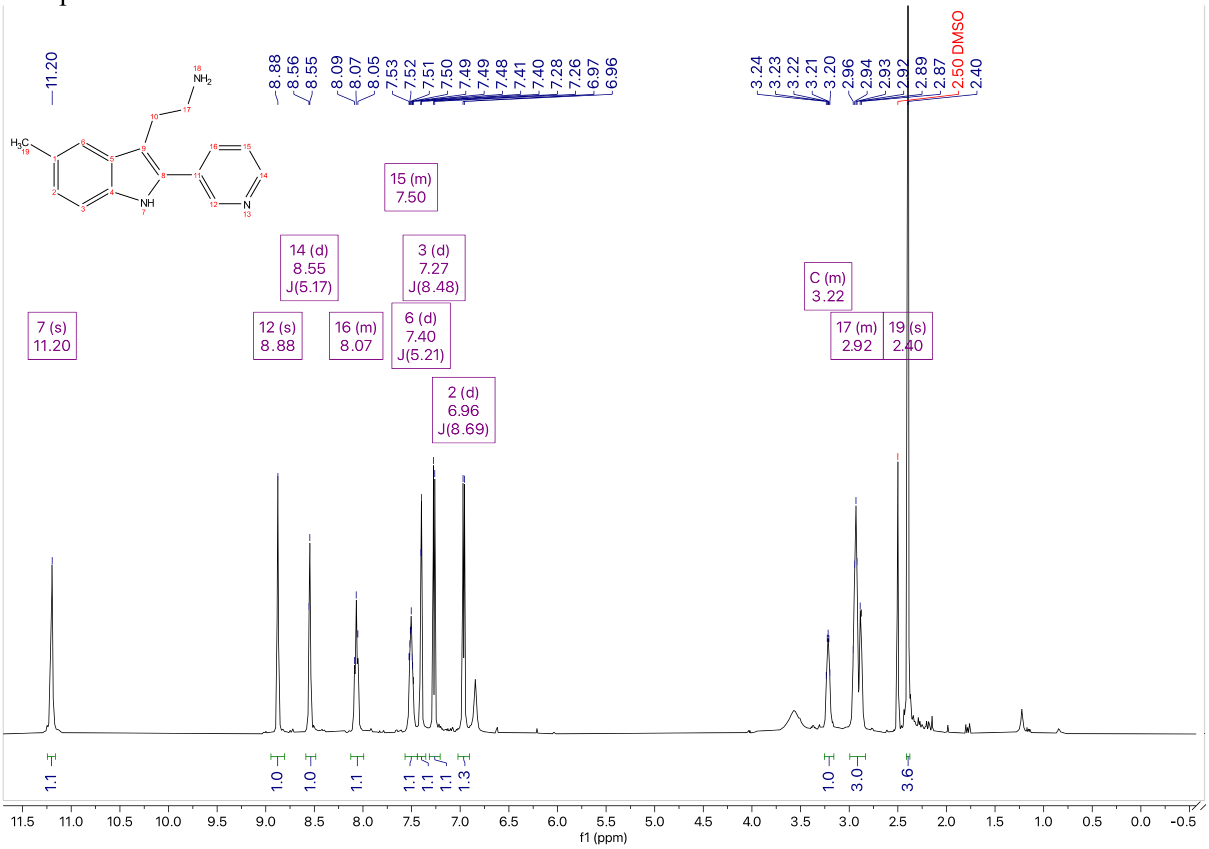

Compound 2 ^{13}C

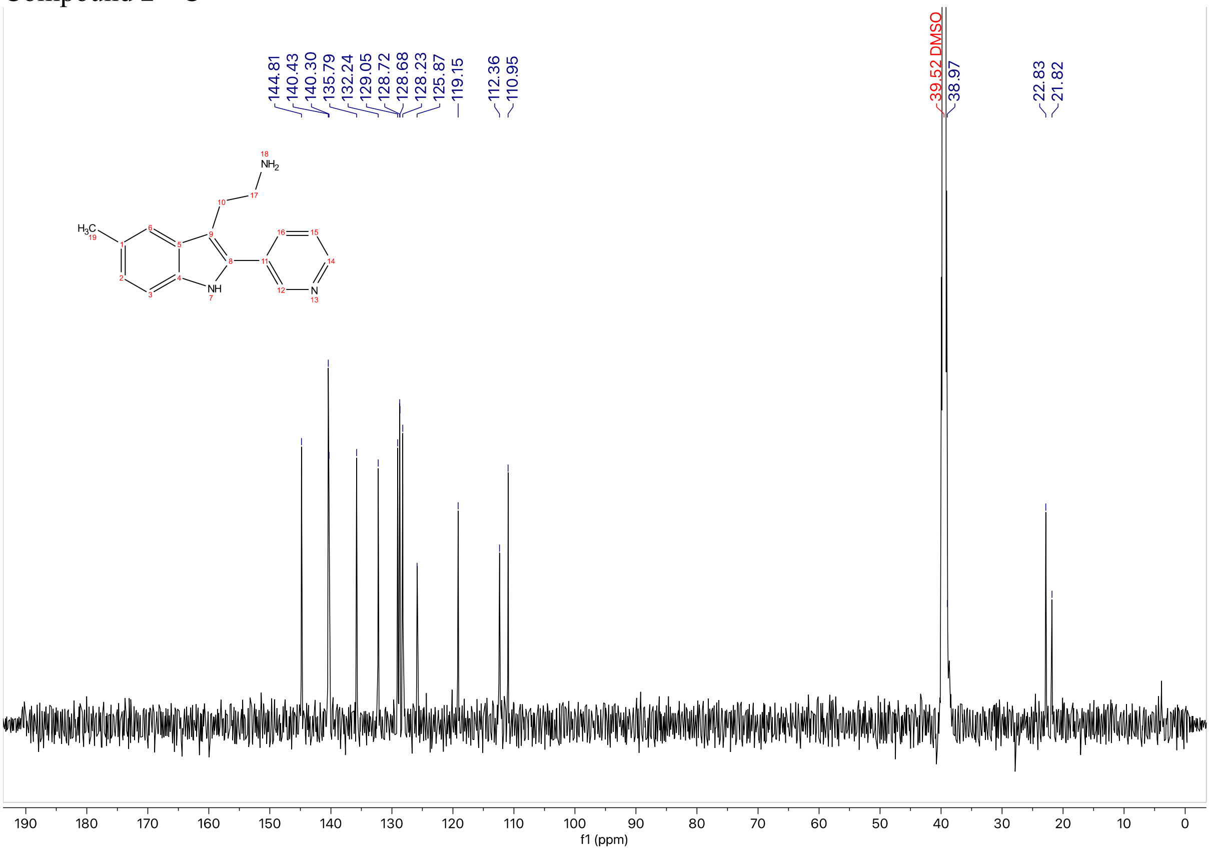

Compound 3 ^{1}H

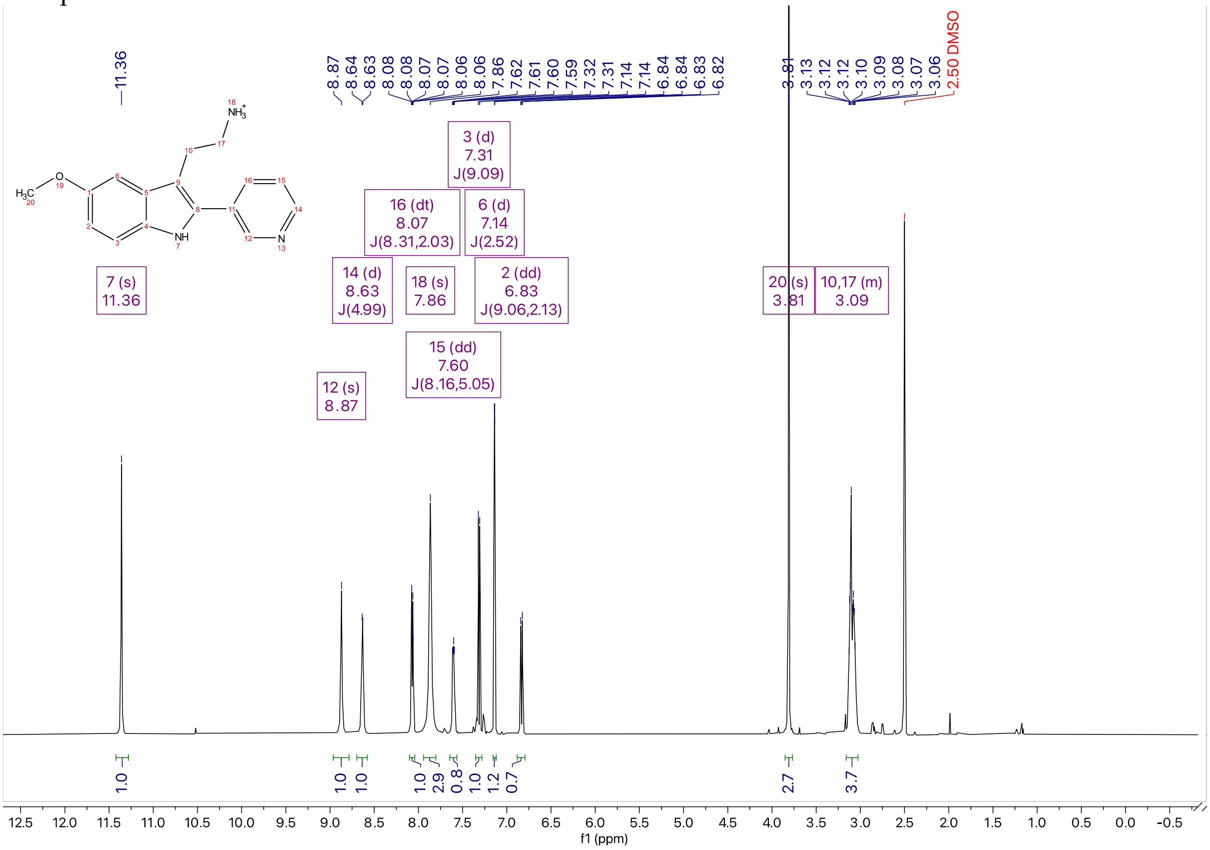

Compound 3 ^{13}C

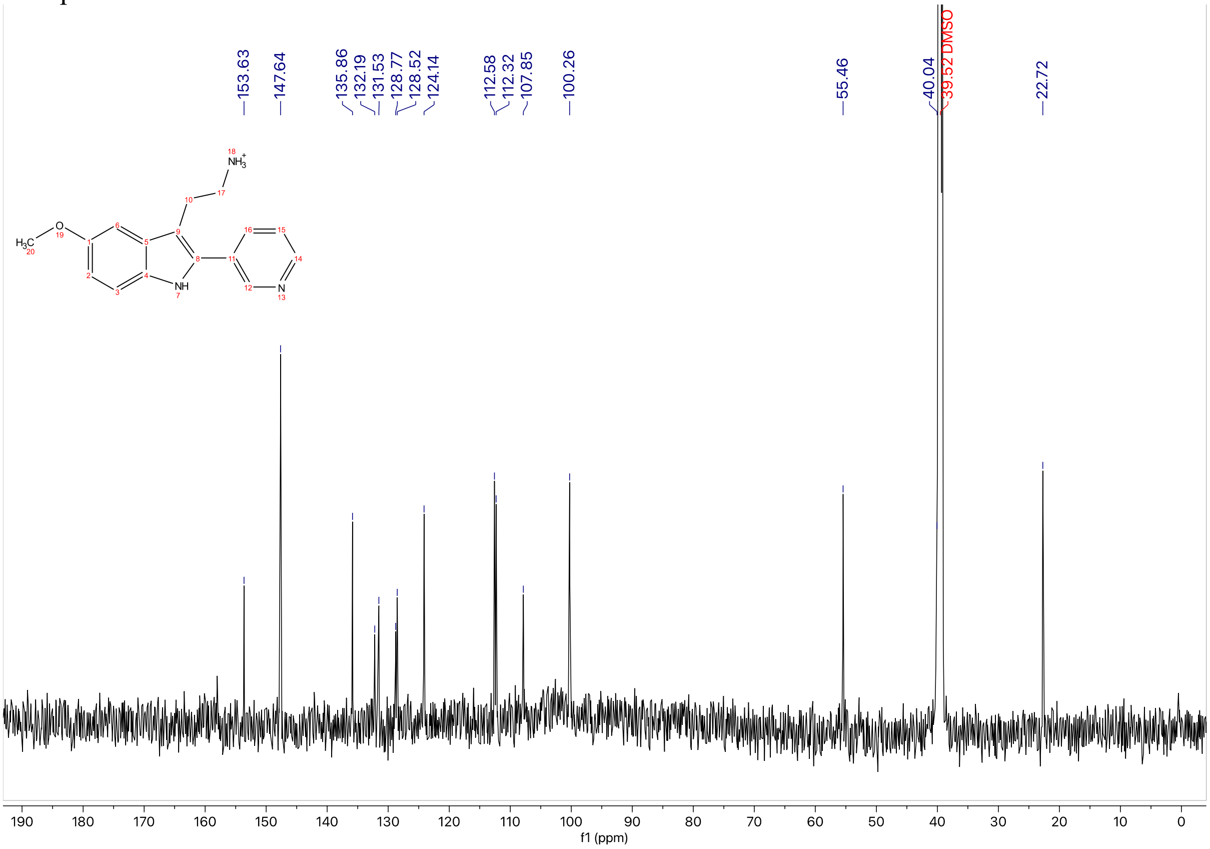

Compound 4 ^{1}H

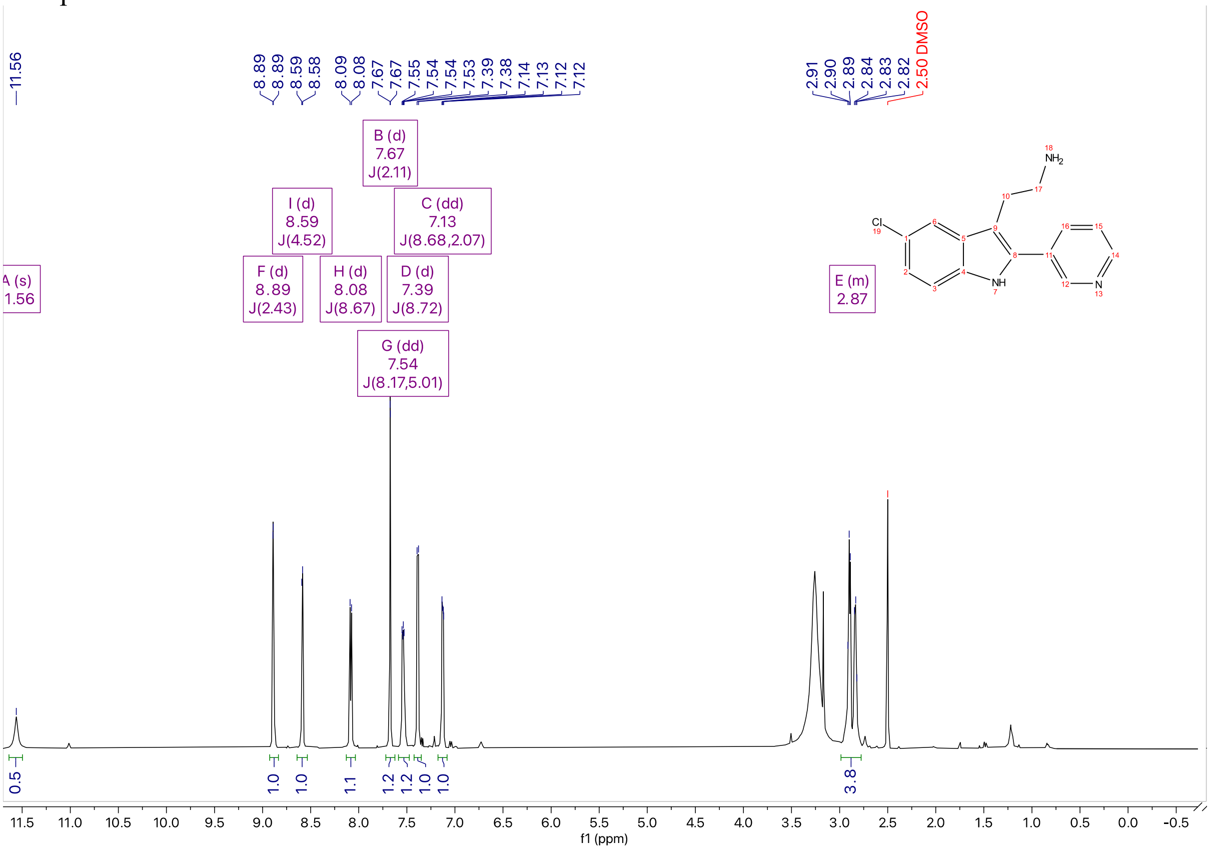

Compound 4 ^{13}C

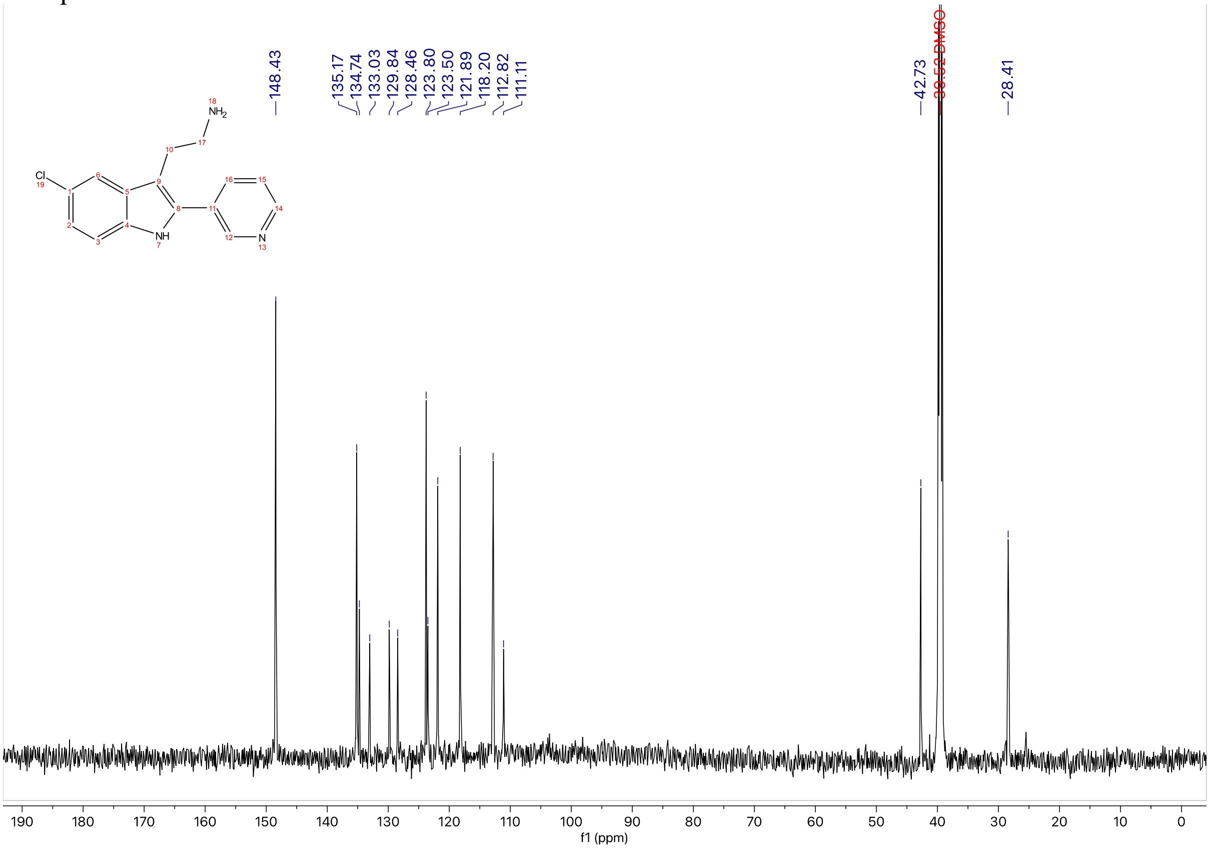

Compound 5 ^{1}H

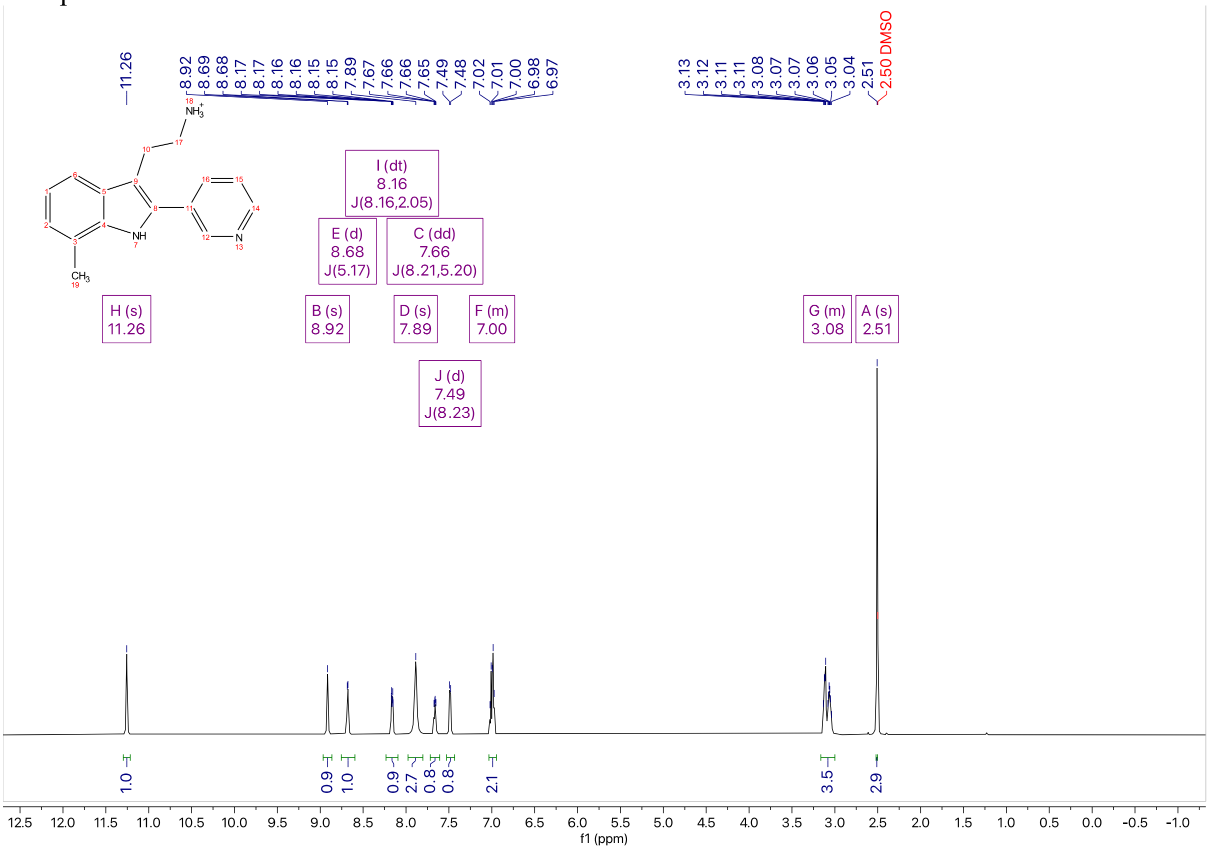

Compound 5 ^{13}C

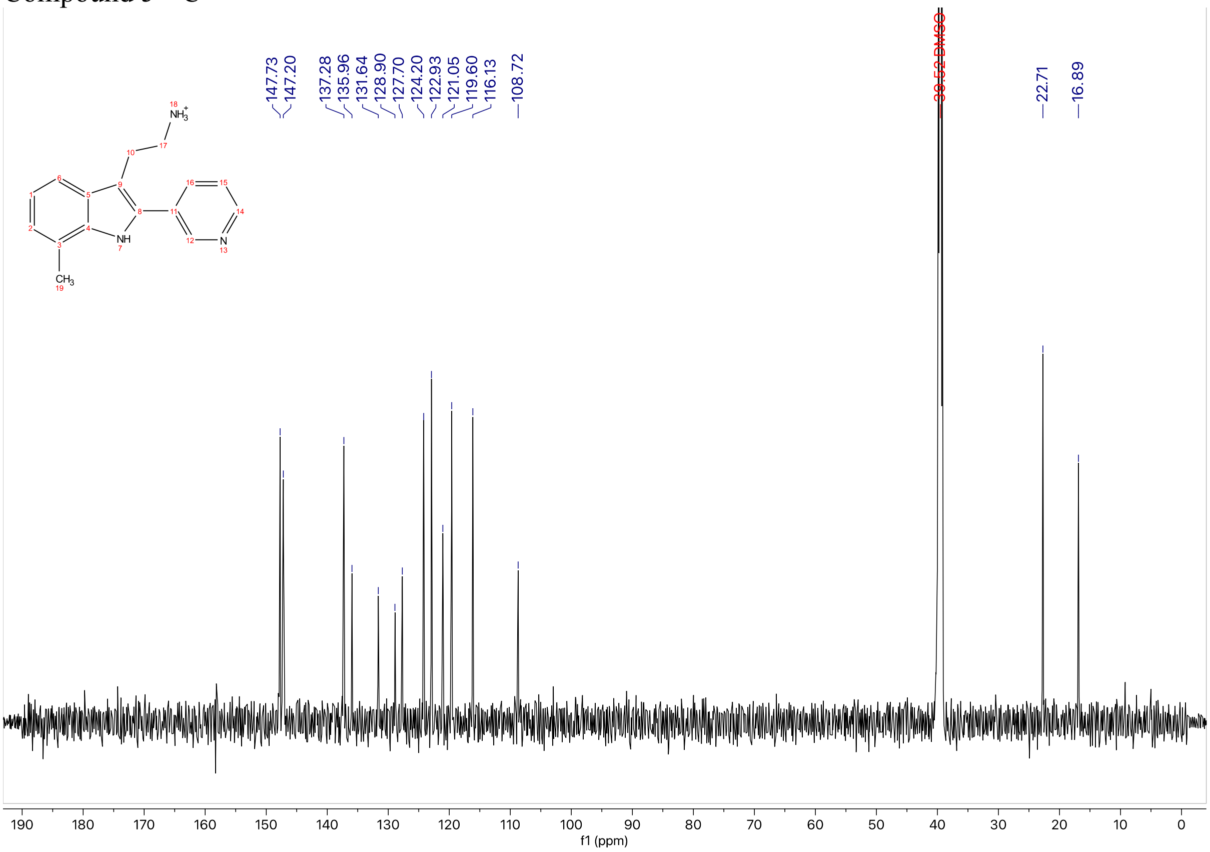

Compound 6 ^{1}H

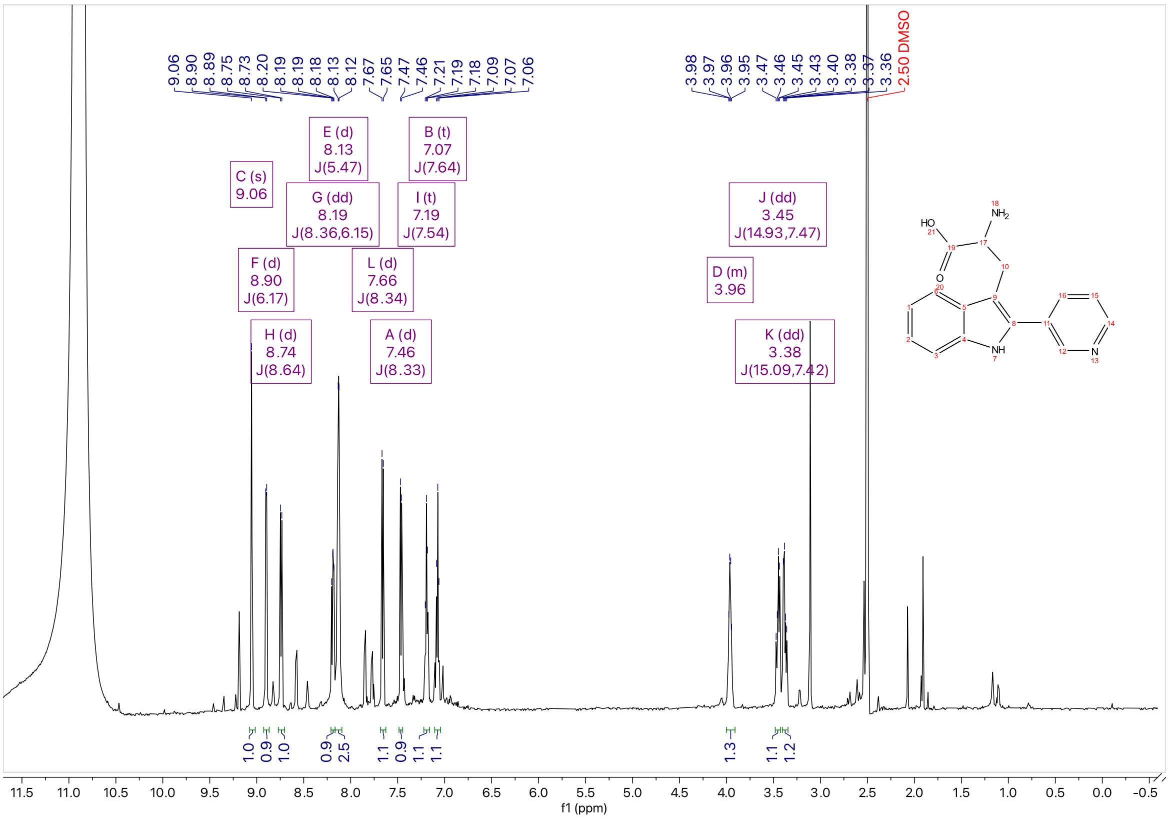

Compound 6 ^{13}C

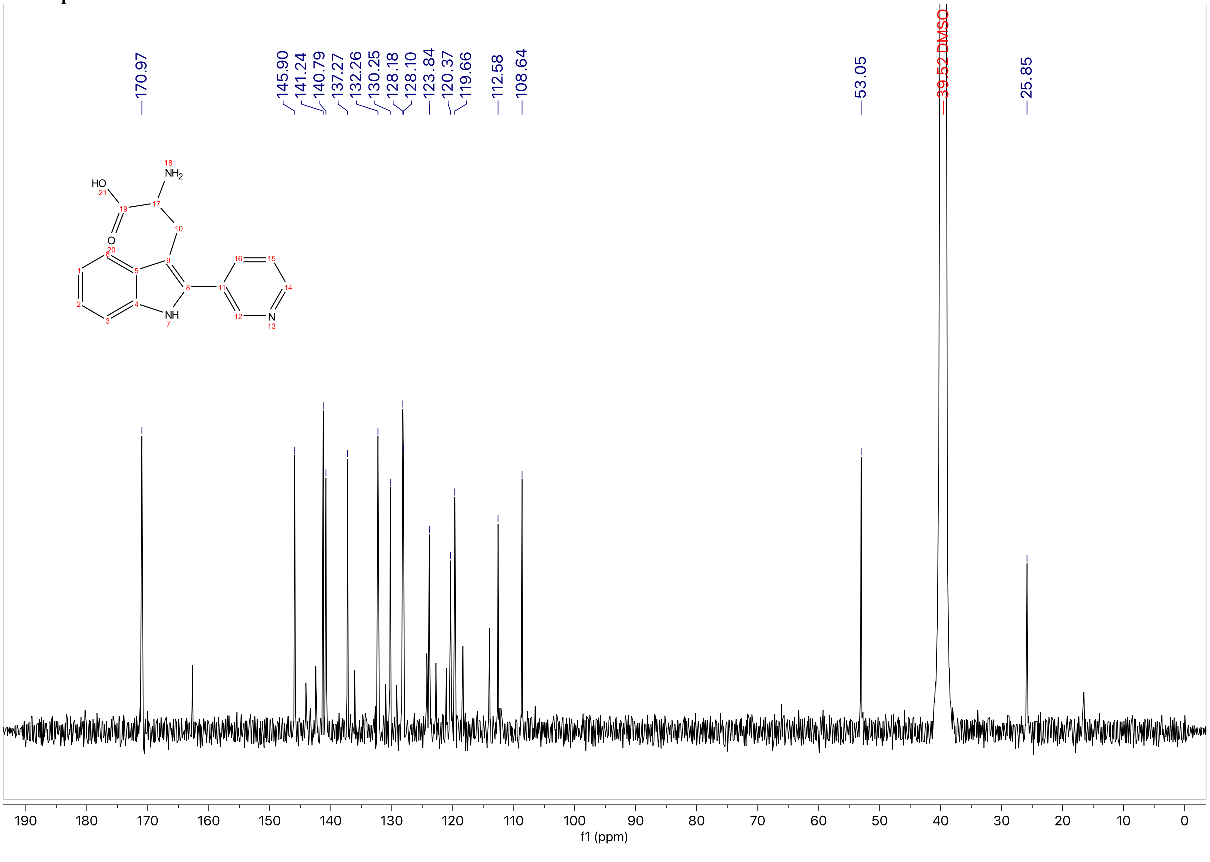

Compound 7 ^{1}H

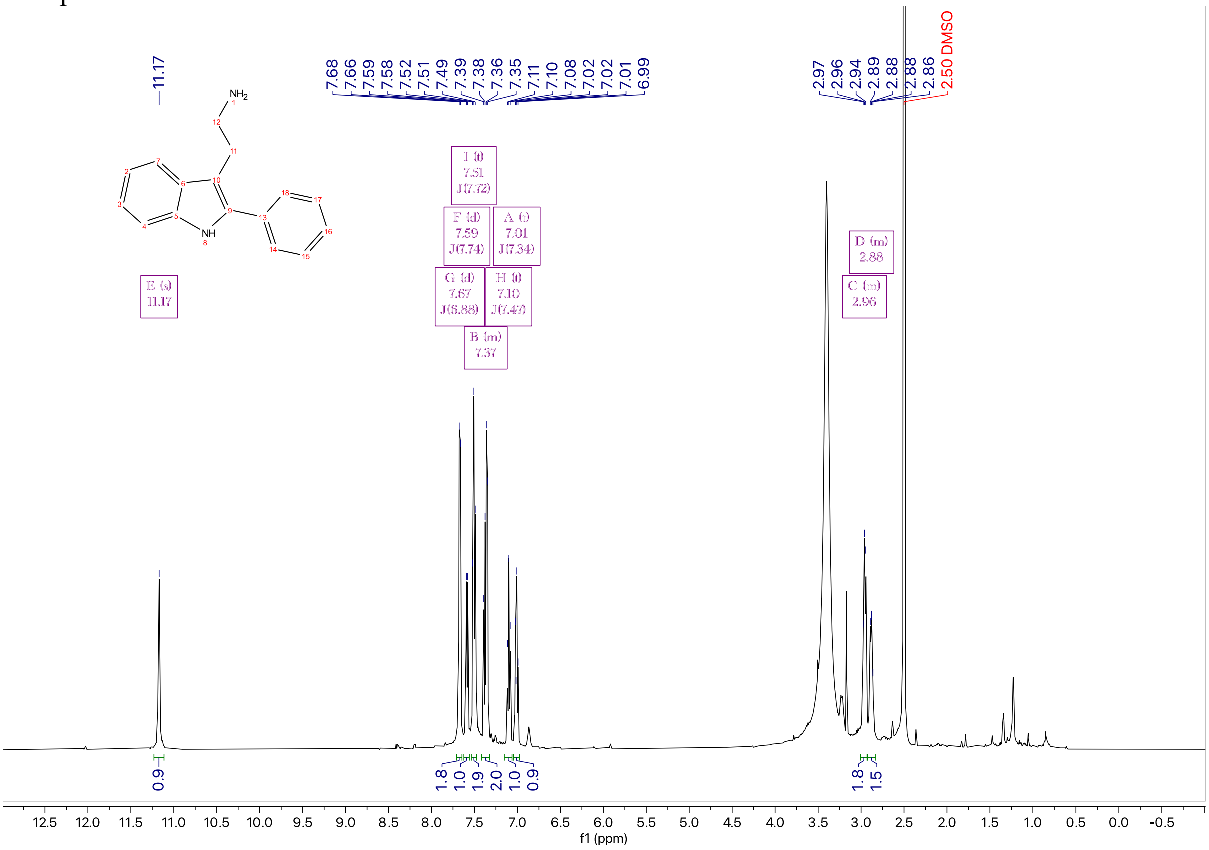

Compound 7 ^{13}C

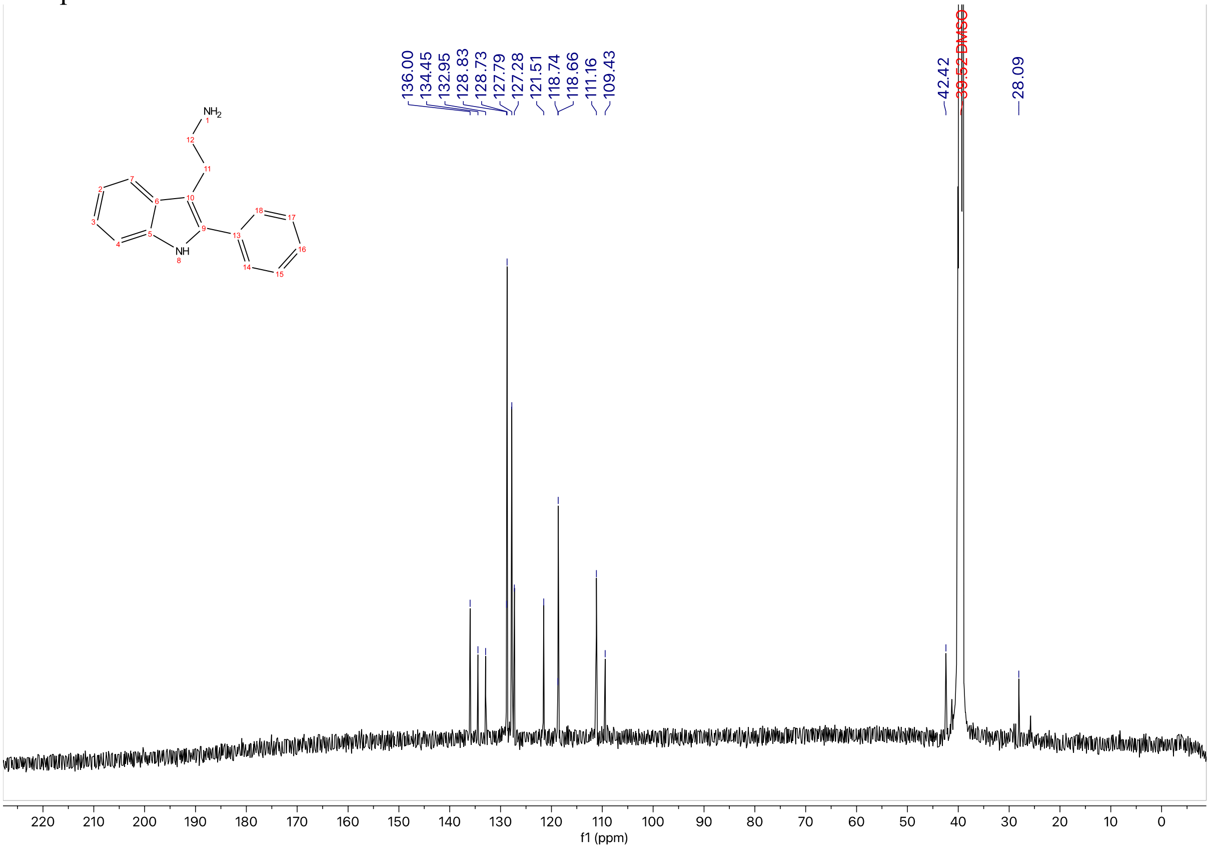

Compound 8 ^{1}H

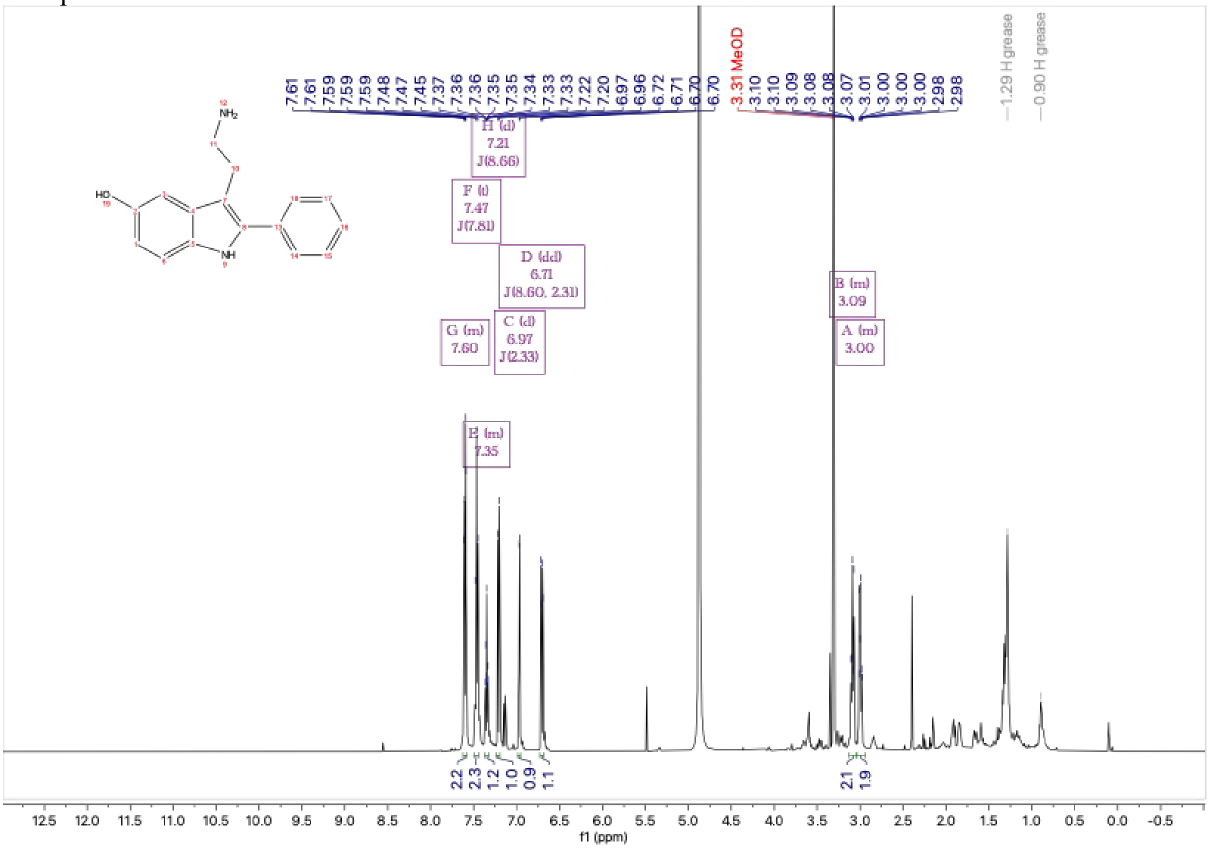

Compound 8 ^{13}C

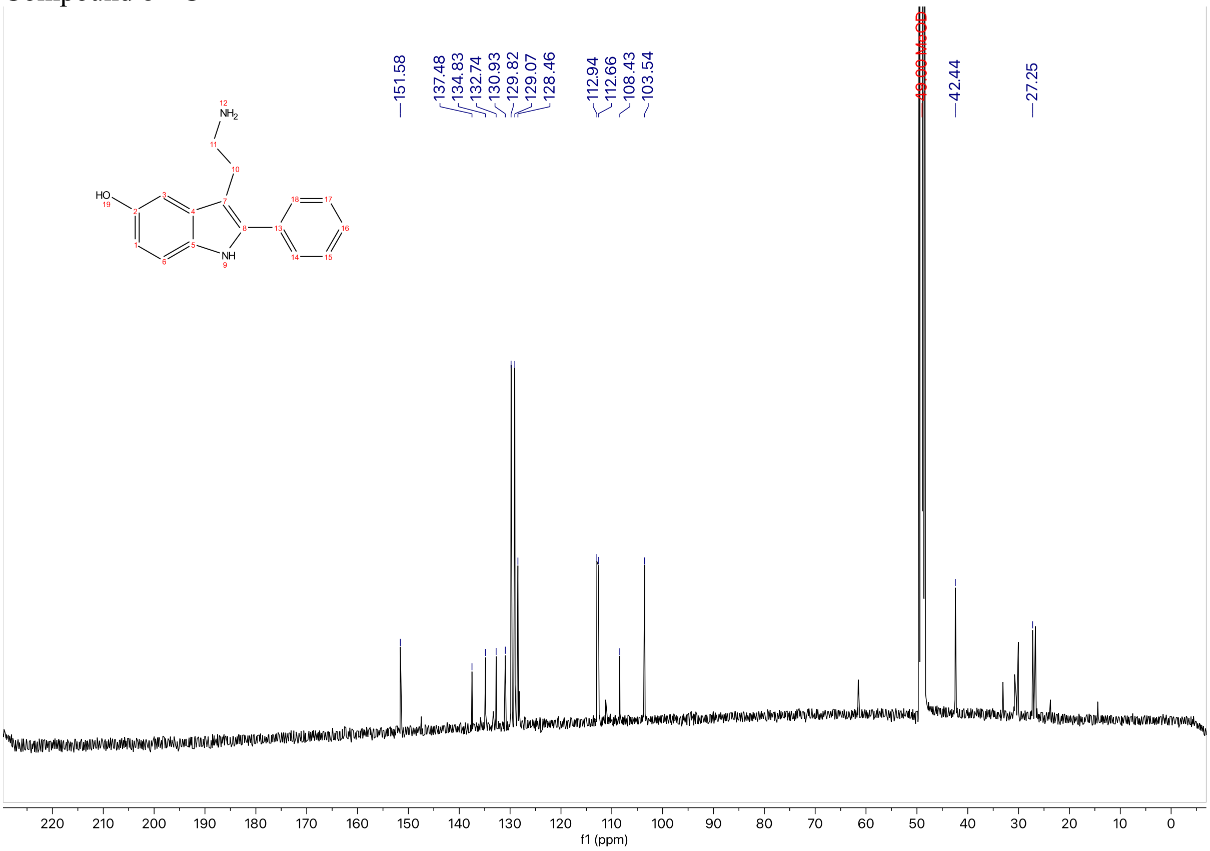

Compound 9 ^{1}H

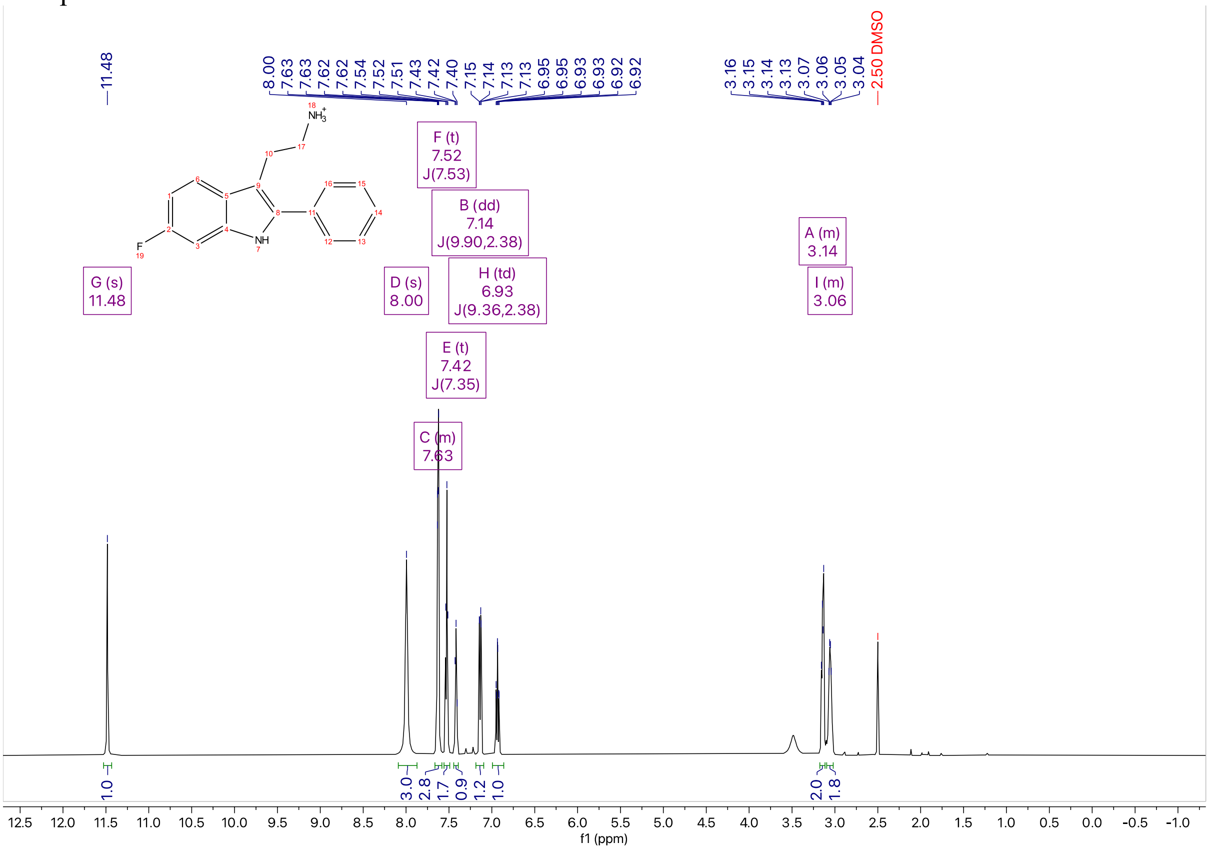

Compound 9 ^{13}C

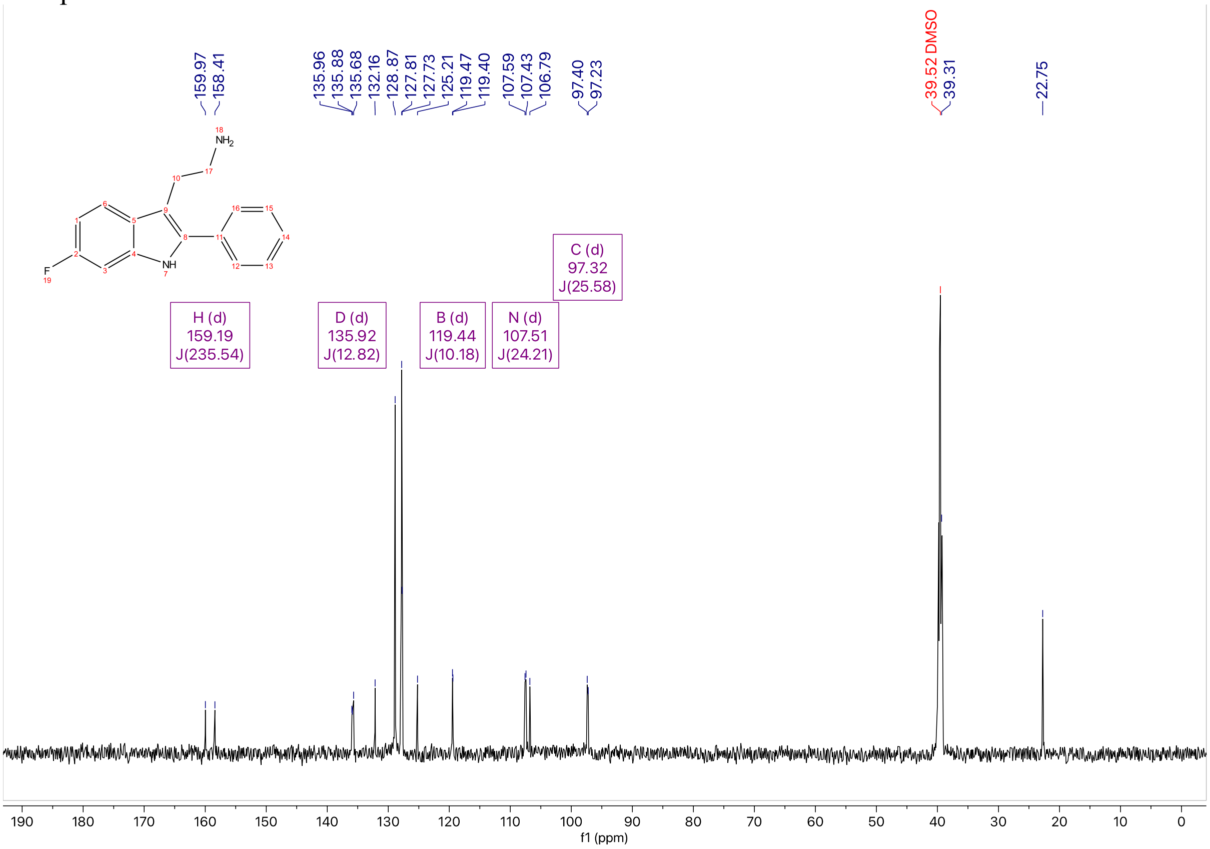

Compound 10 ^{1}H

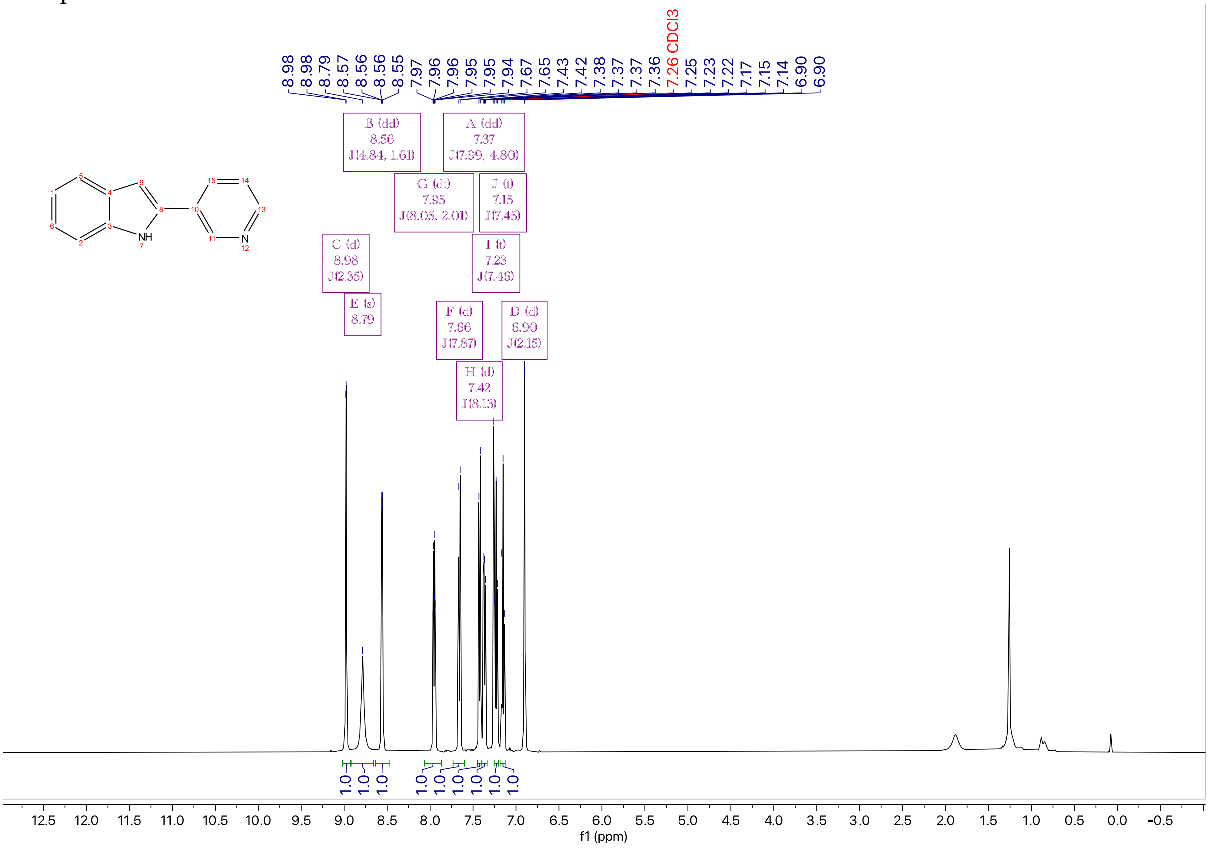

Compound 10 ^{13}C

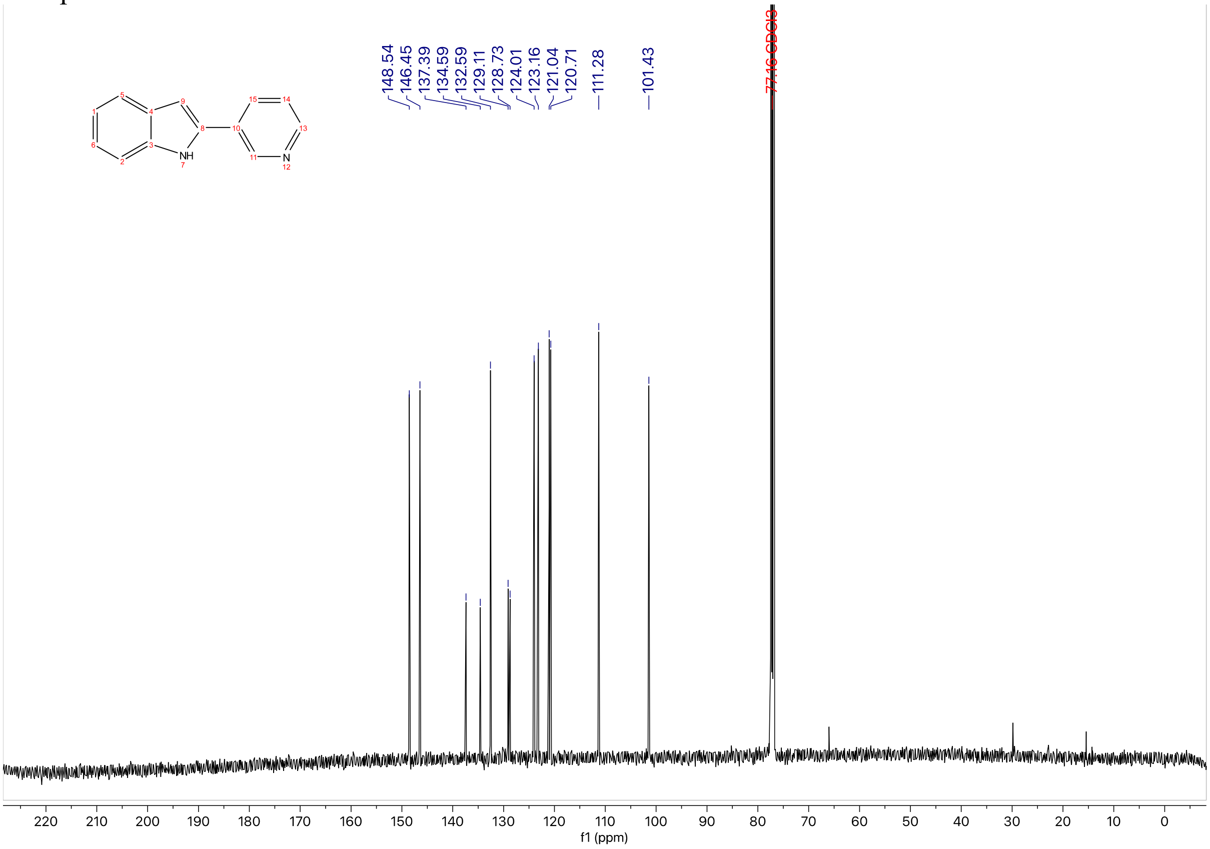

Compound 11 ^{1}H

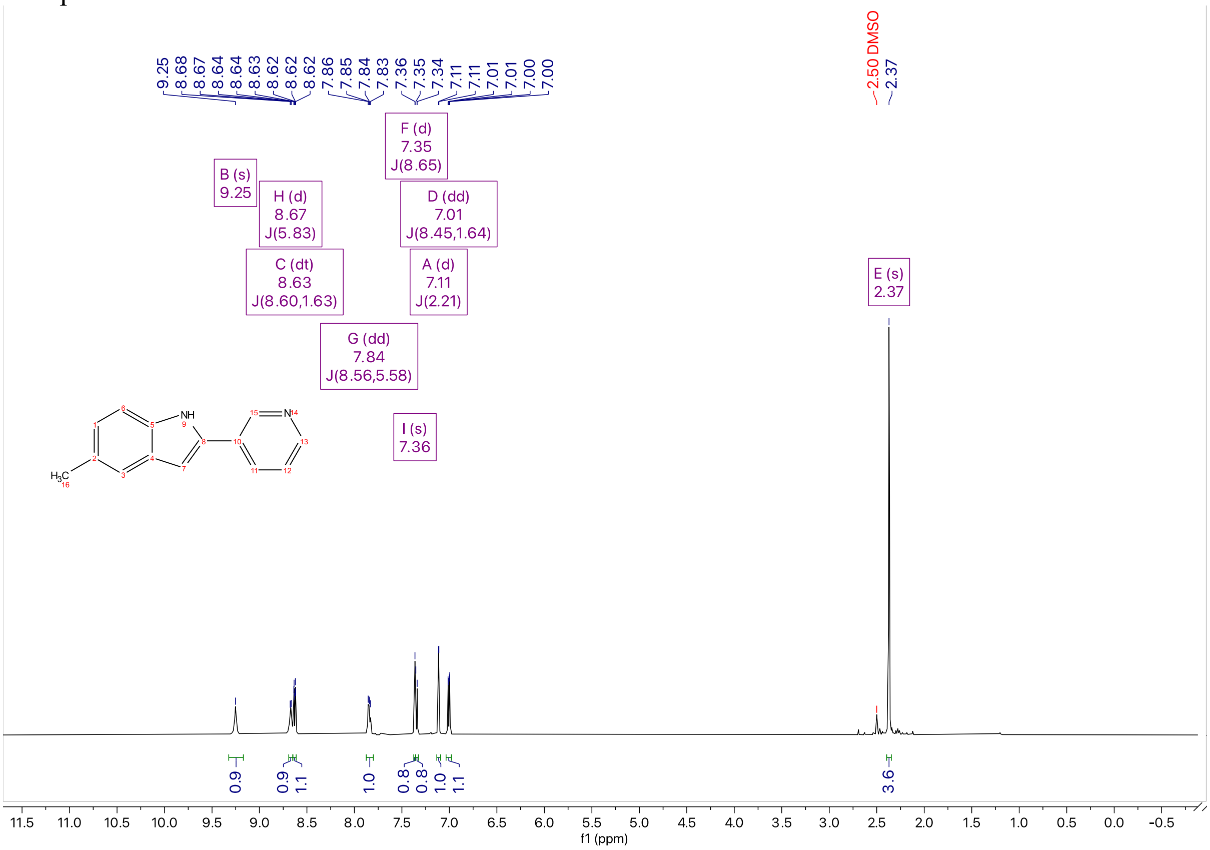

Compound 11 ^{13}C

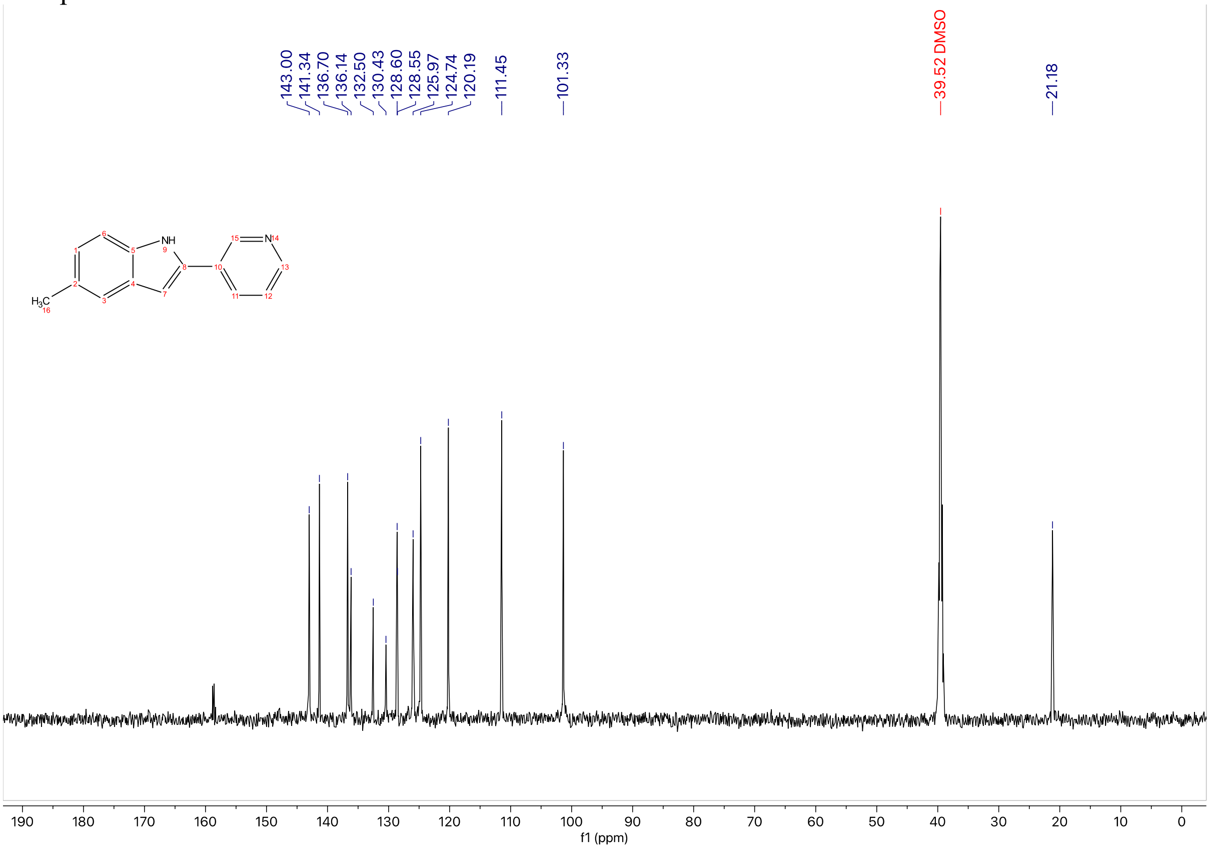

Compound 12 ^{1}H

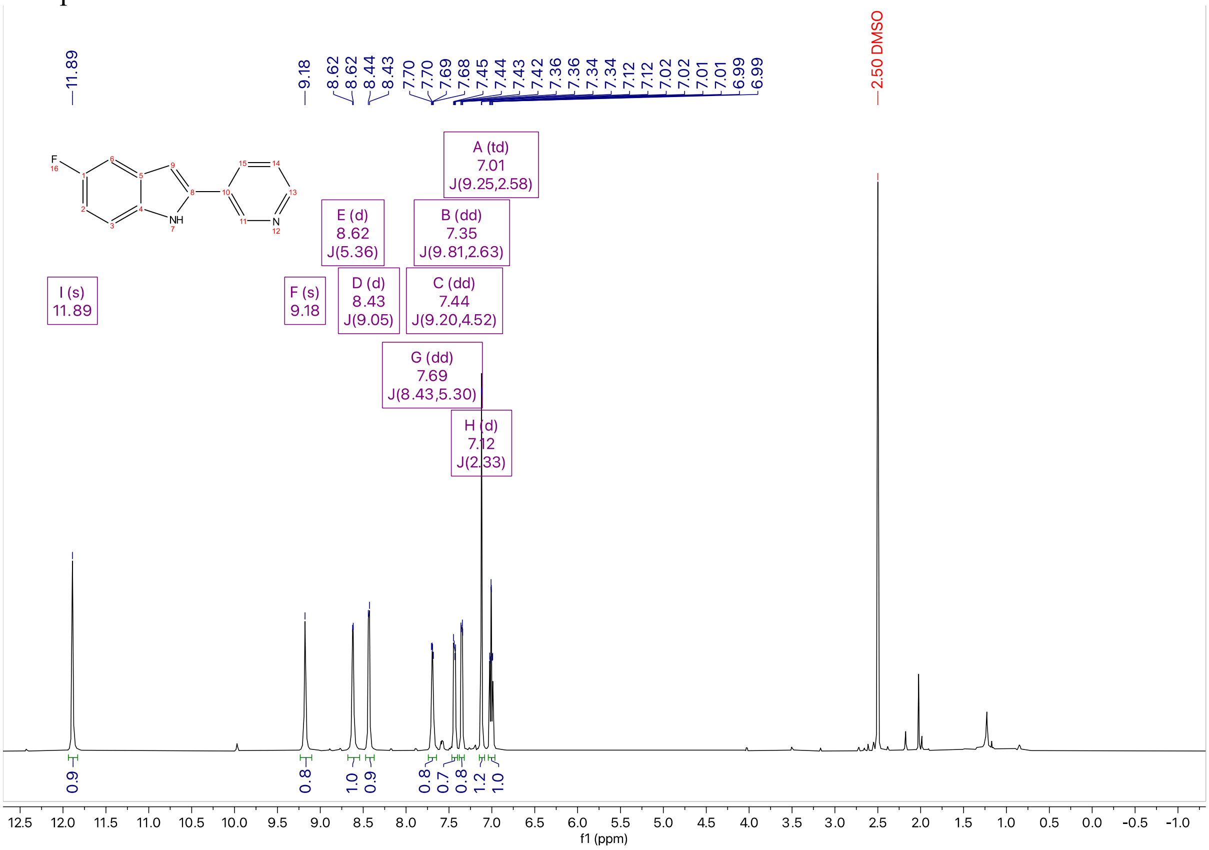

Compound 12 ^{13}C

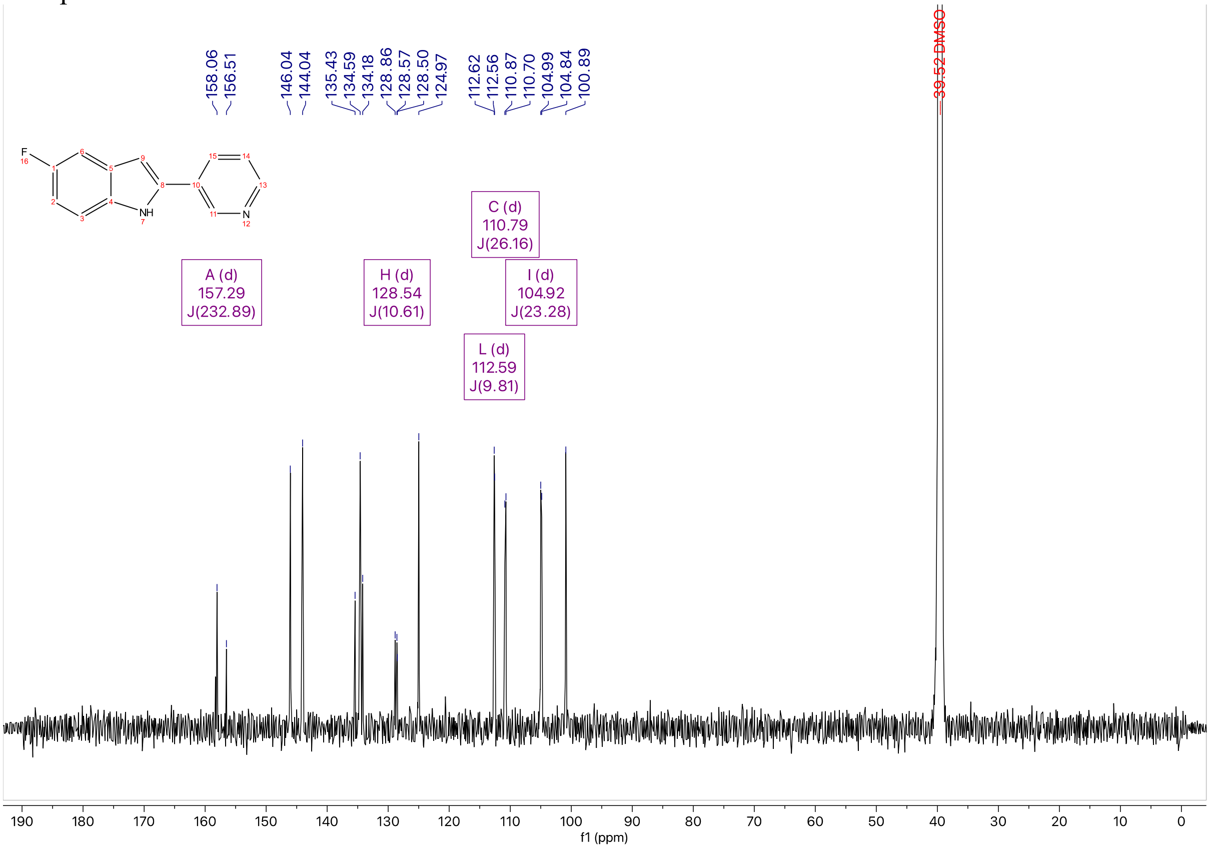

Compound 13 ^{1}H

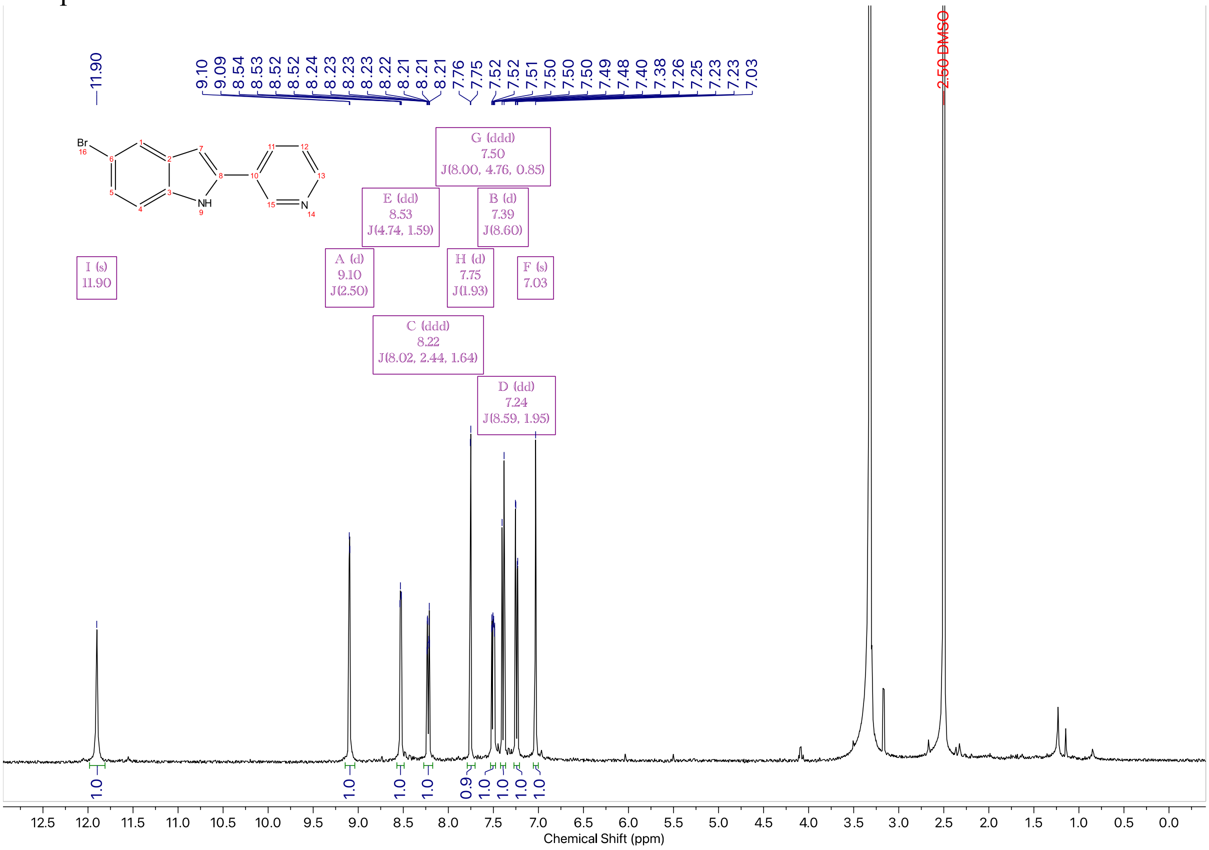

Compound 13 ^{13}C

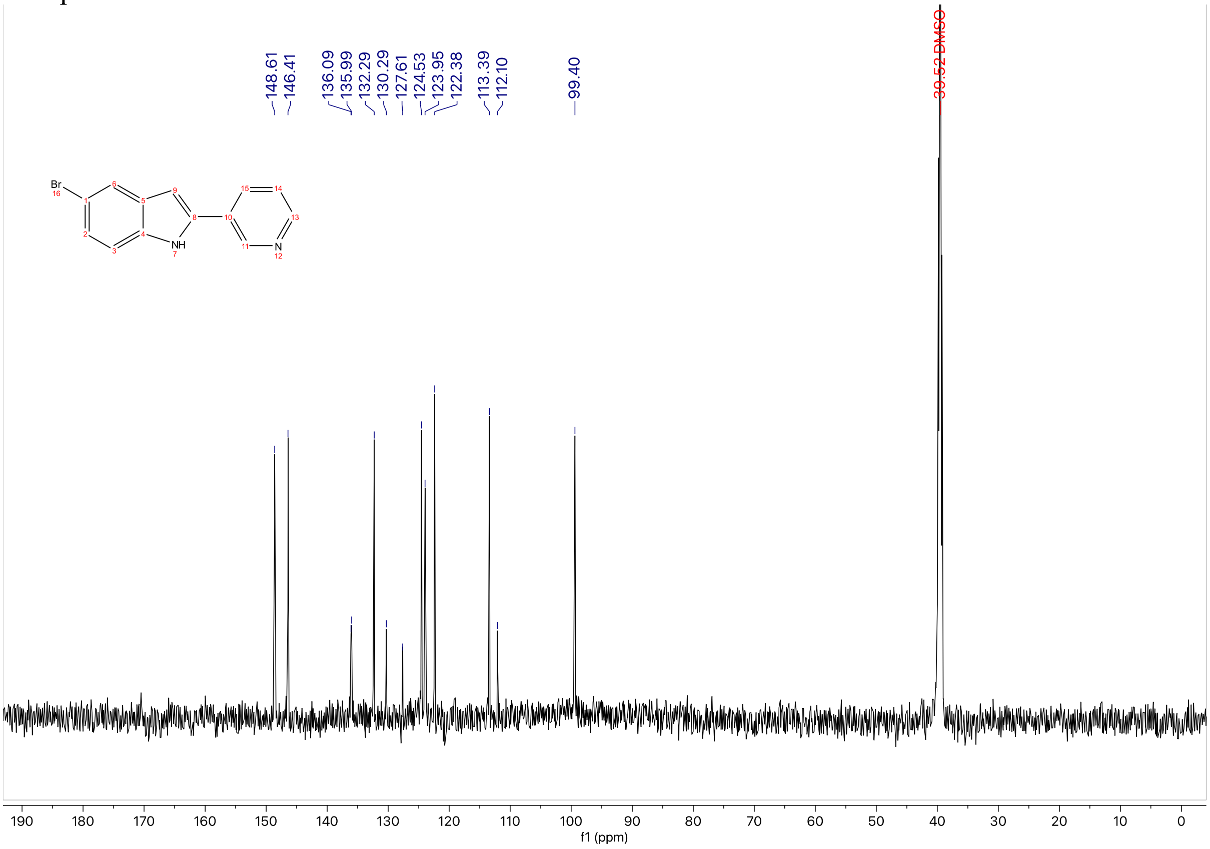

Compound 14 ^{1}H

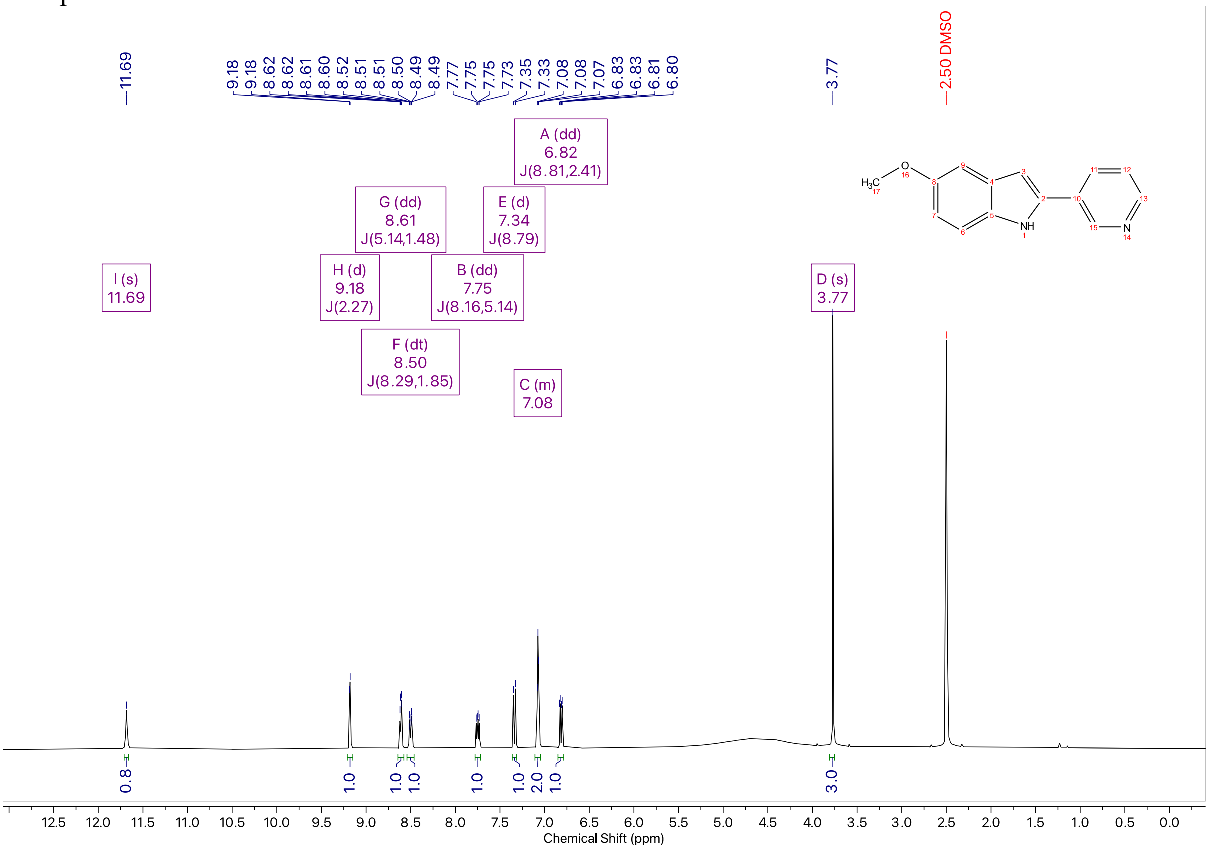

Compound 14 ^{13}C

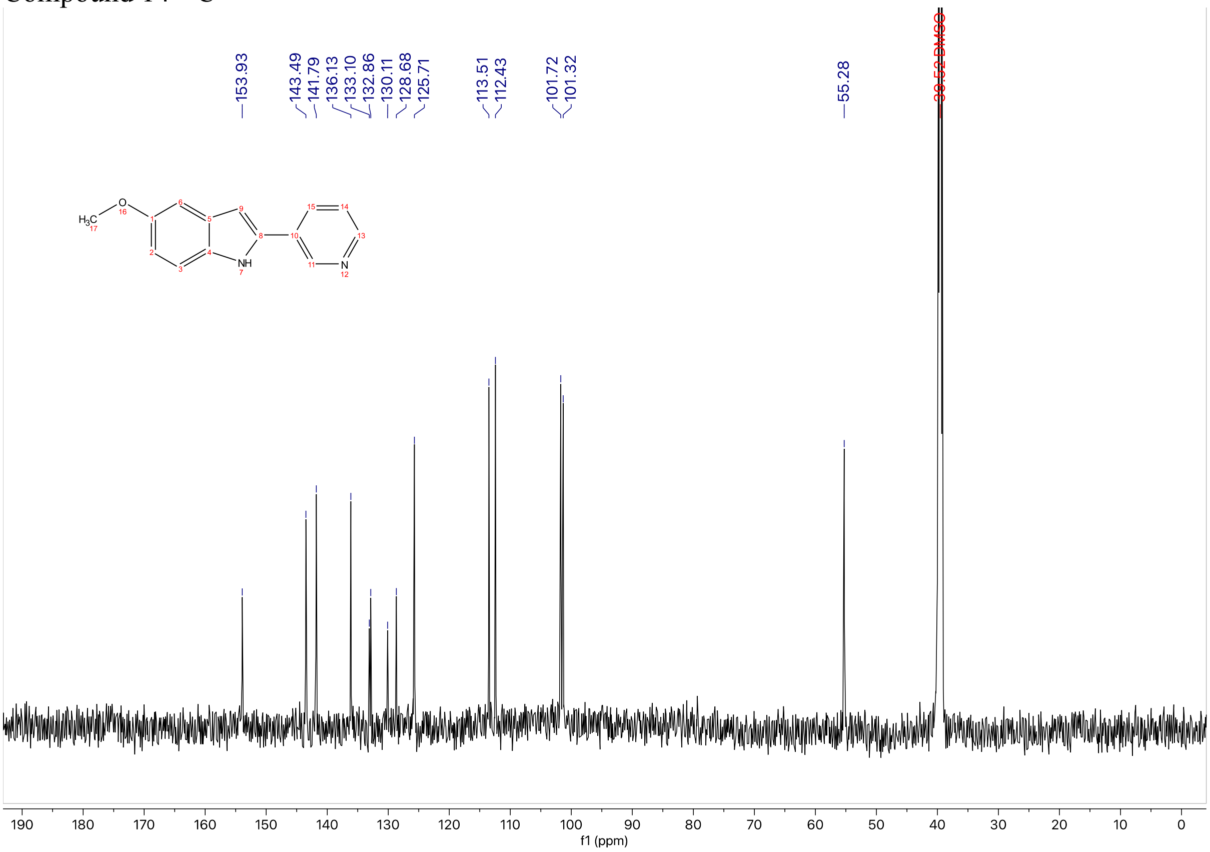

Compound 15 ^{1}H

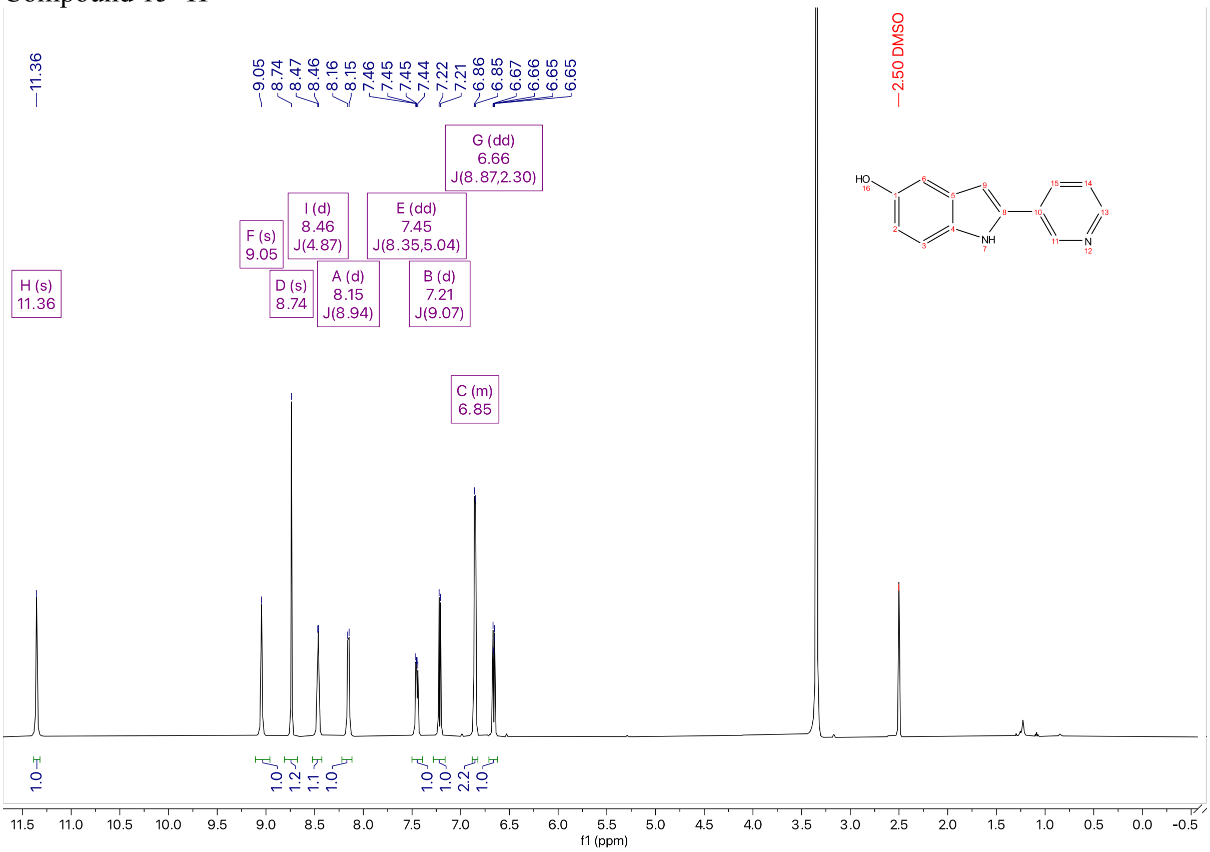

Compound 15 ^{13}C

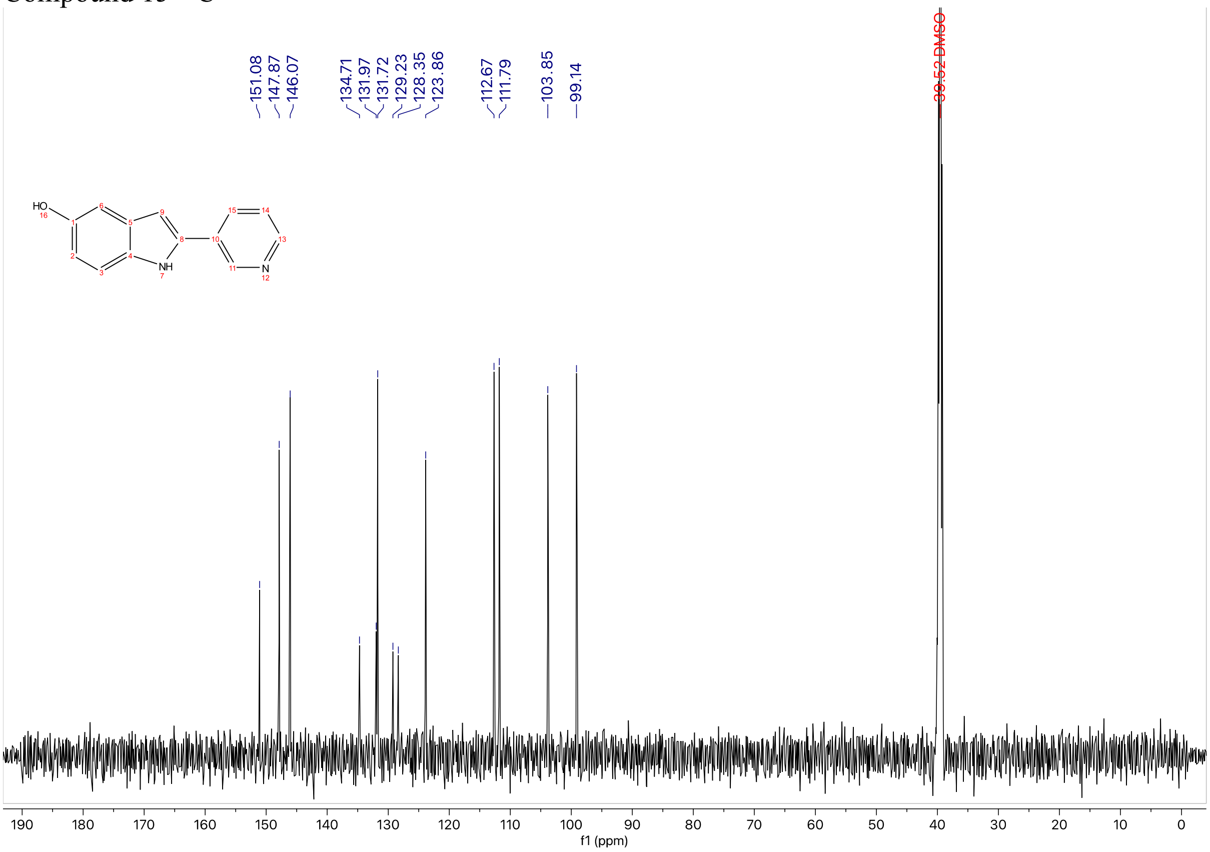

Compound 16 ^{1}H

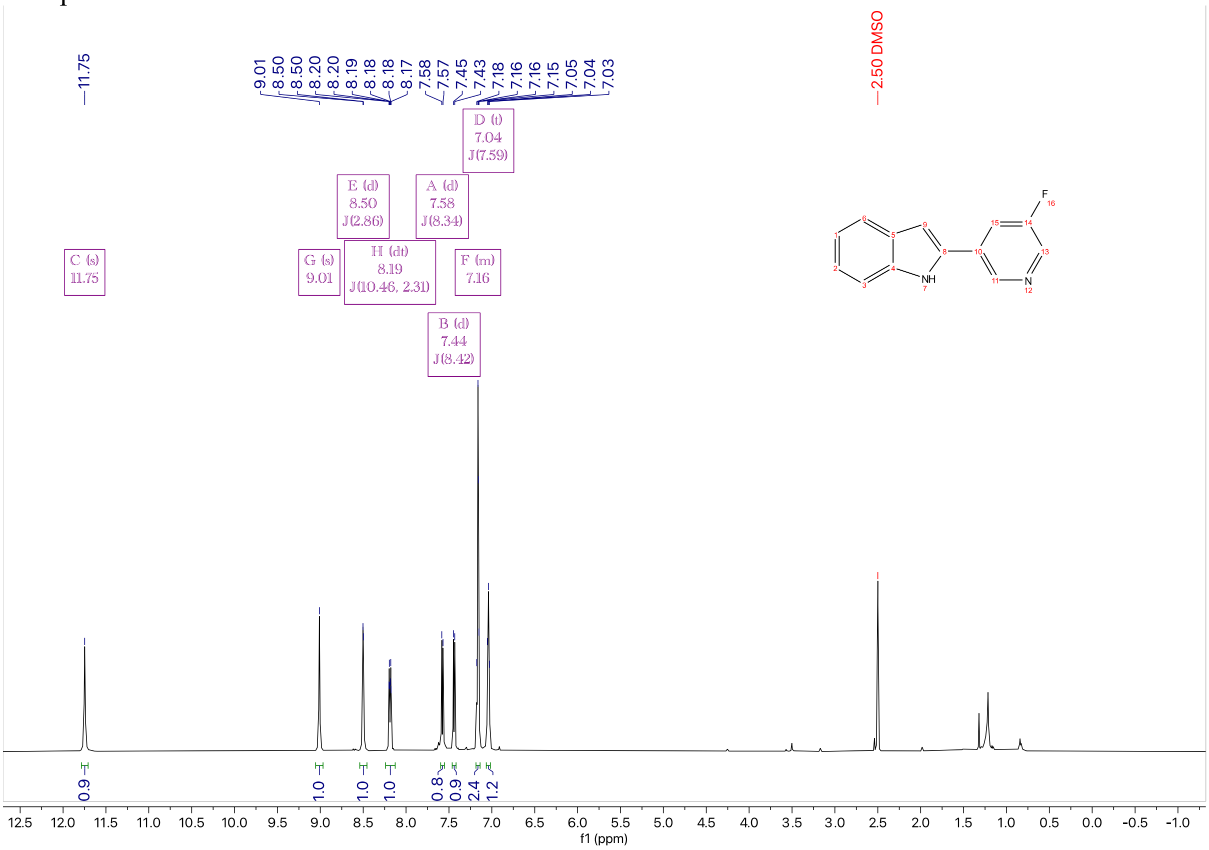

Compound 16 ^{13}C

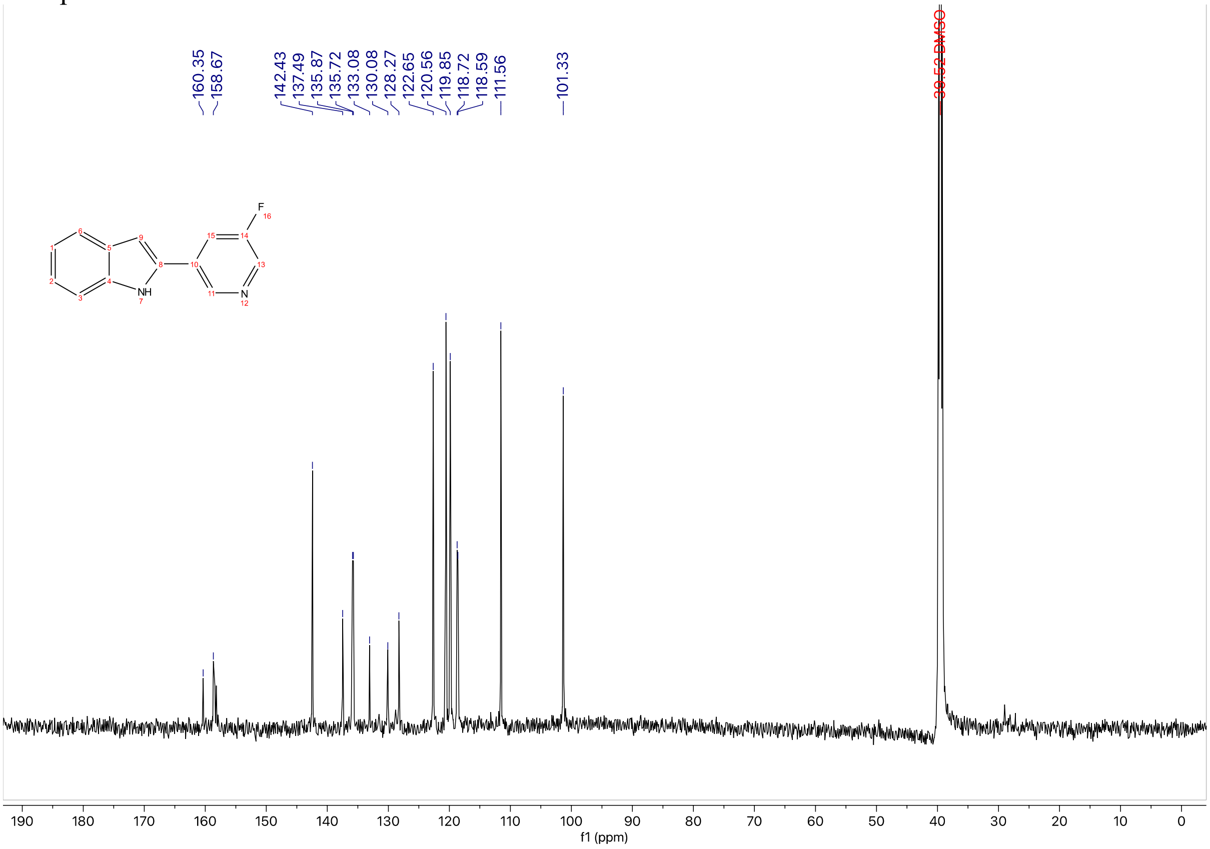

Compound 17 ^{1}H

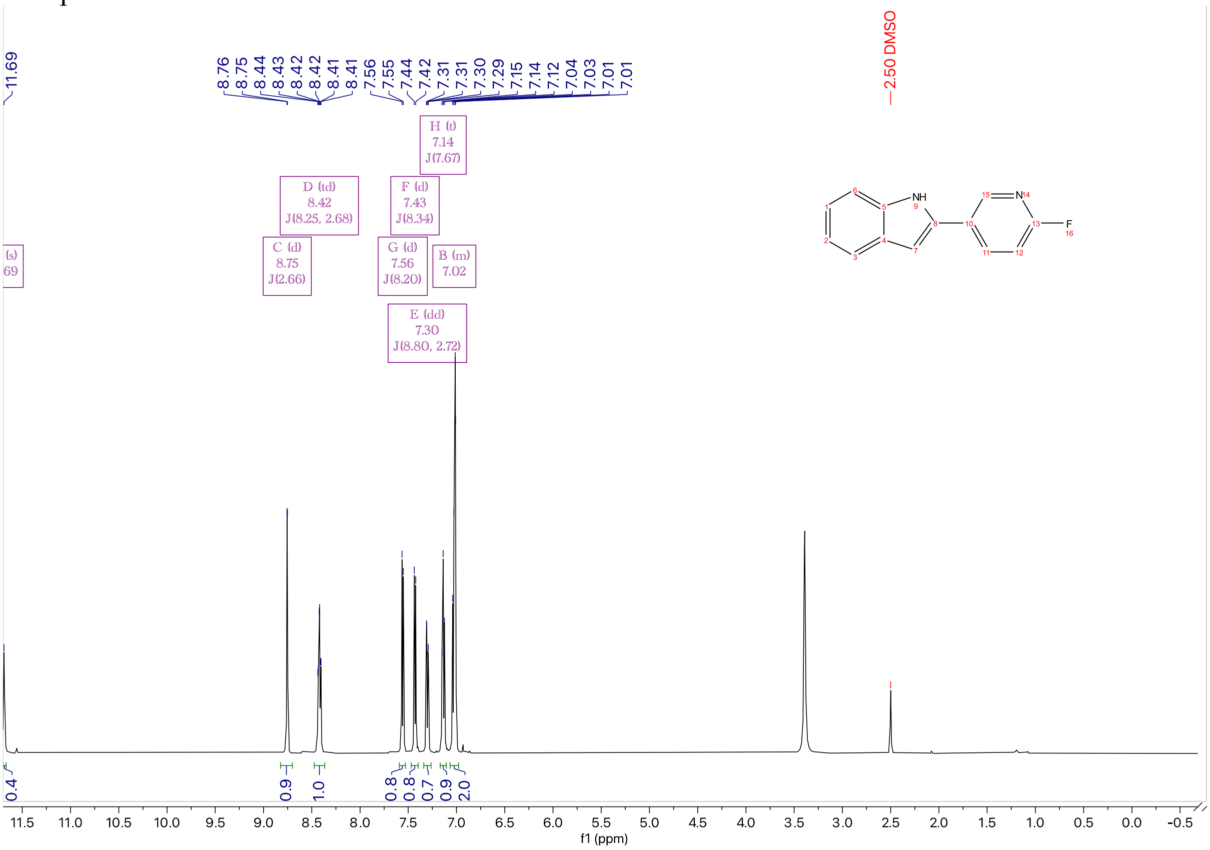

Compound 17 ^{13}C

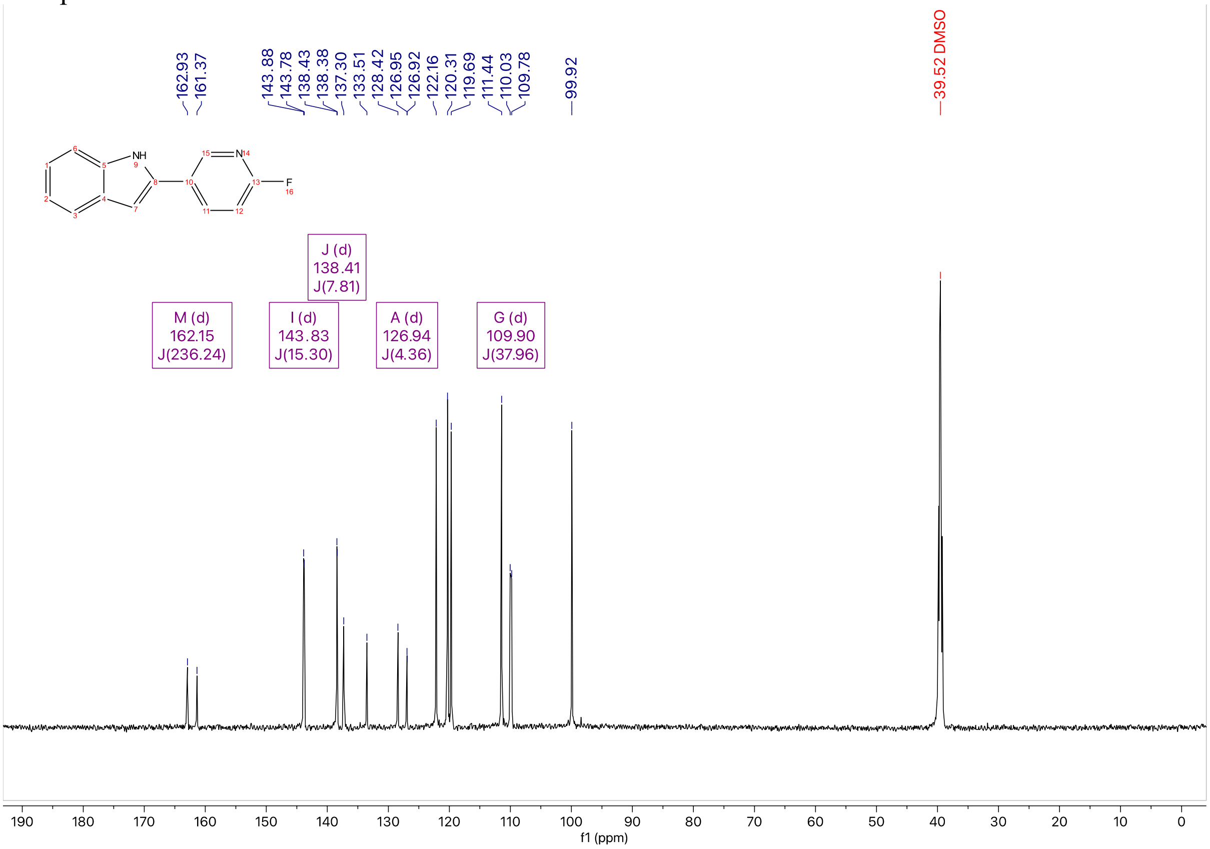

Compound 18 ^{1}H

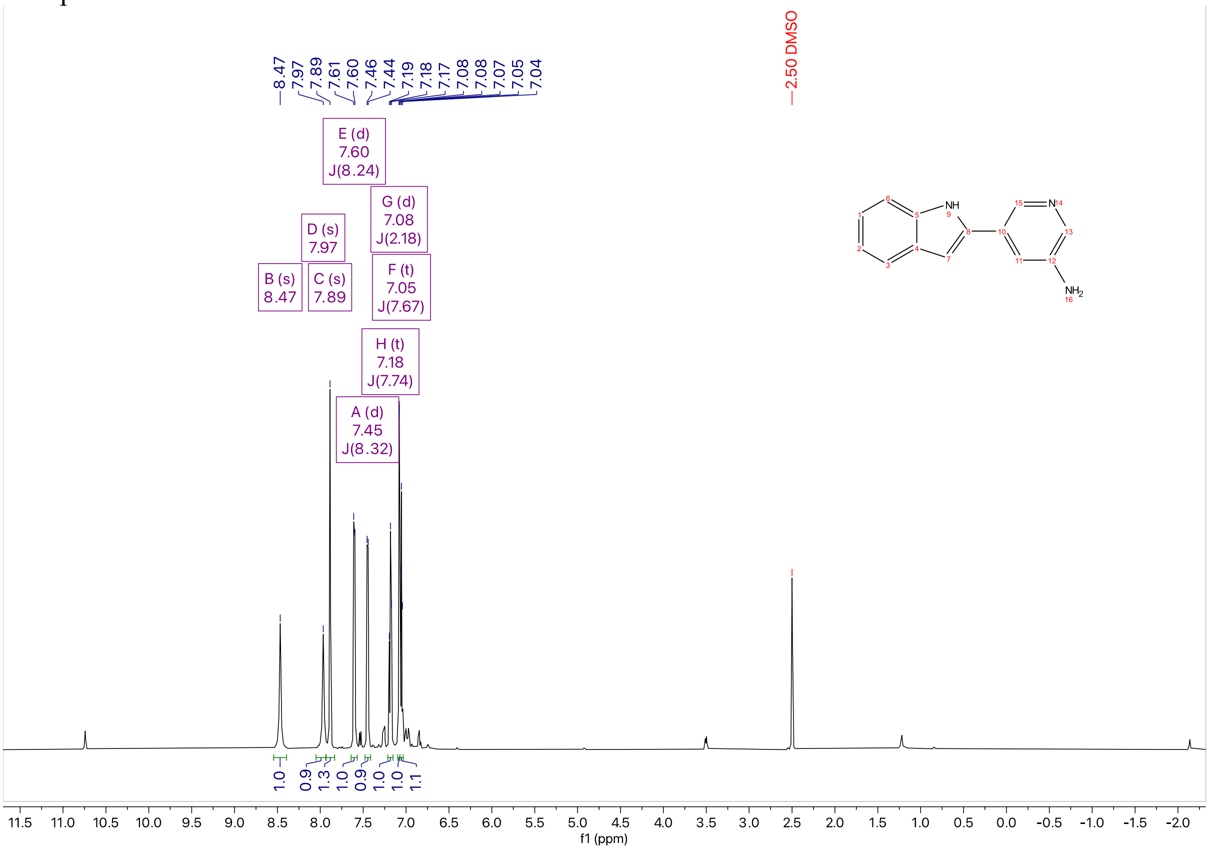

Compound 18 ^{13}C

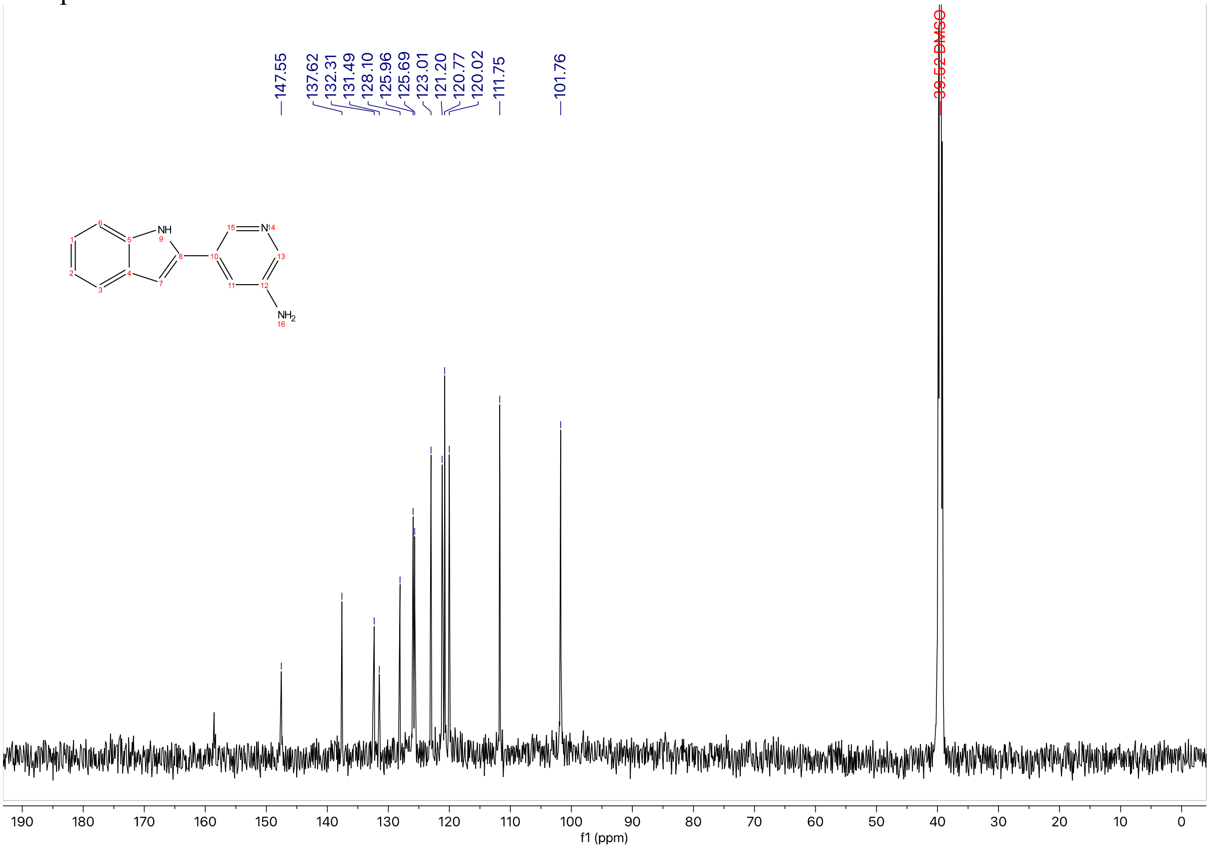

Compound 19 ^{1}H

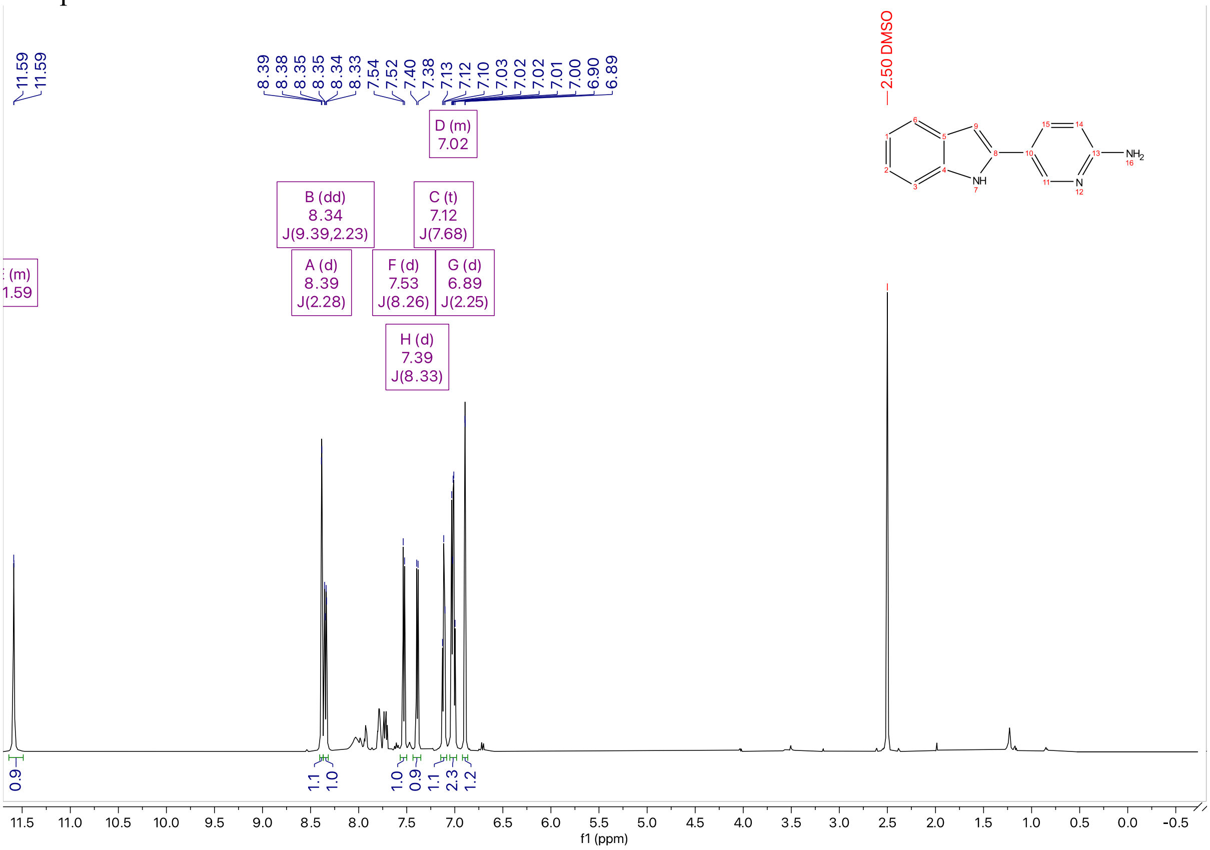

Compound 19 ^{13}C

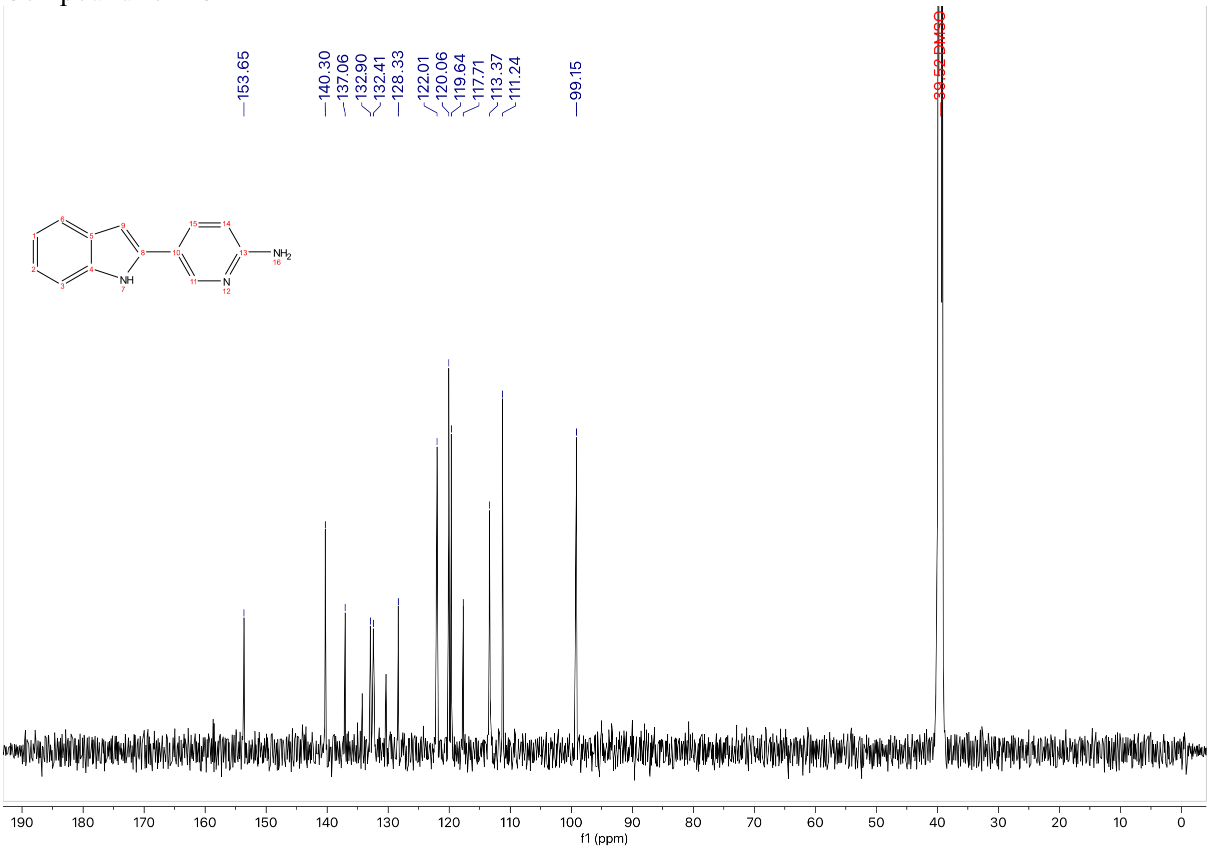

Compound 20 ^{1}H

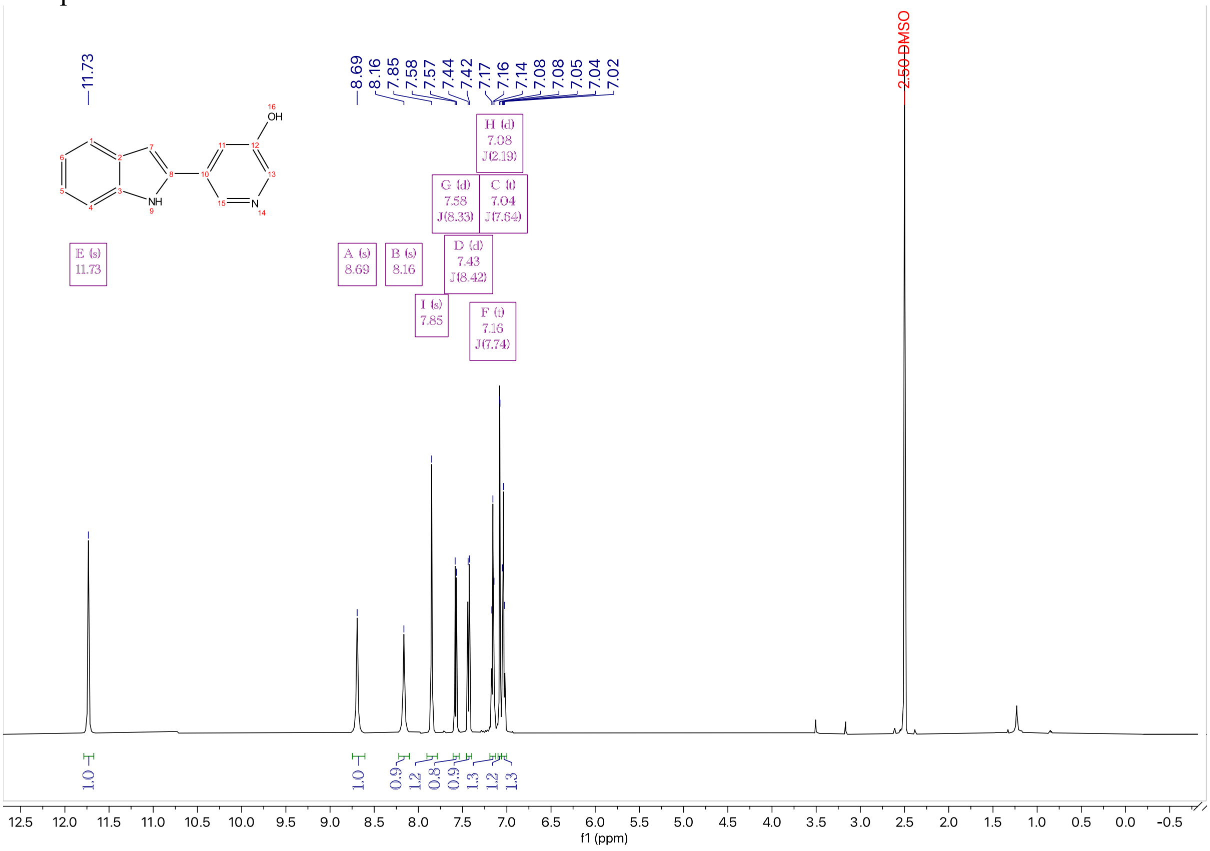

Compound 20 ^{13}C

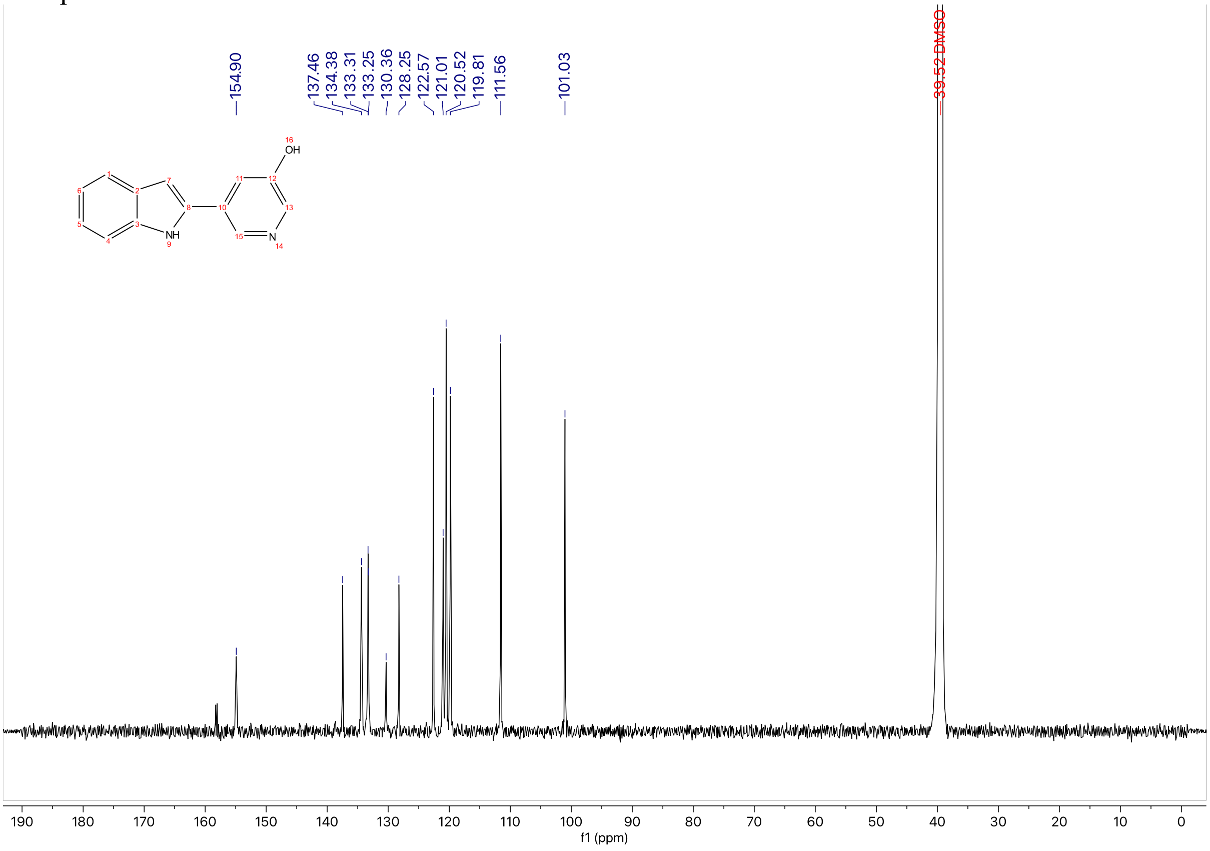

Compound 21 ^{1}H

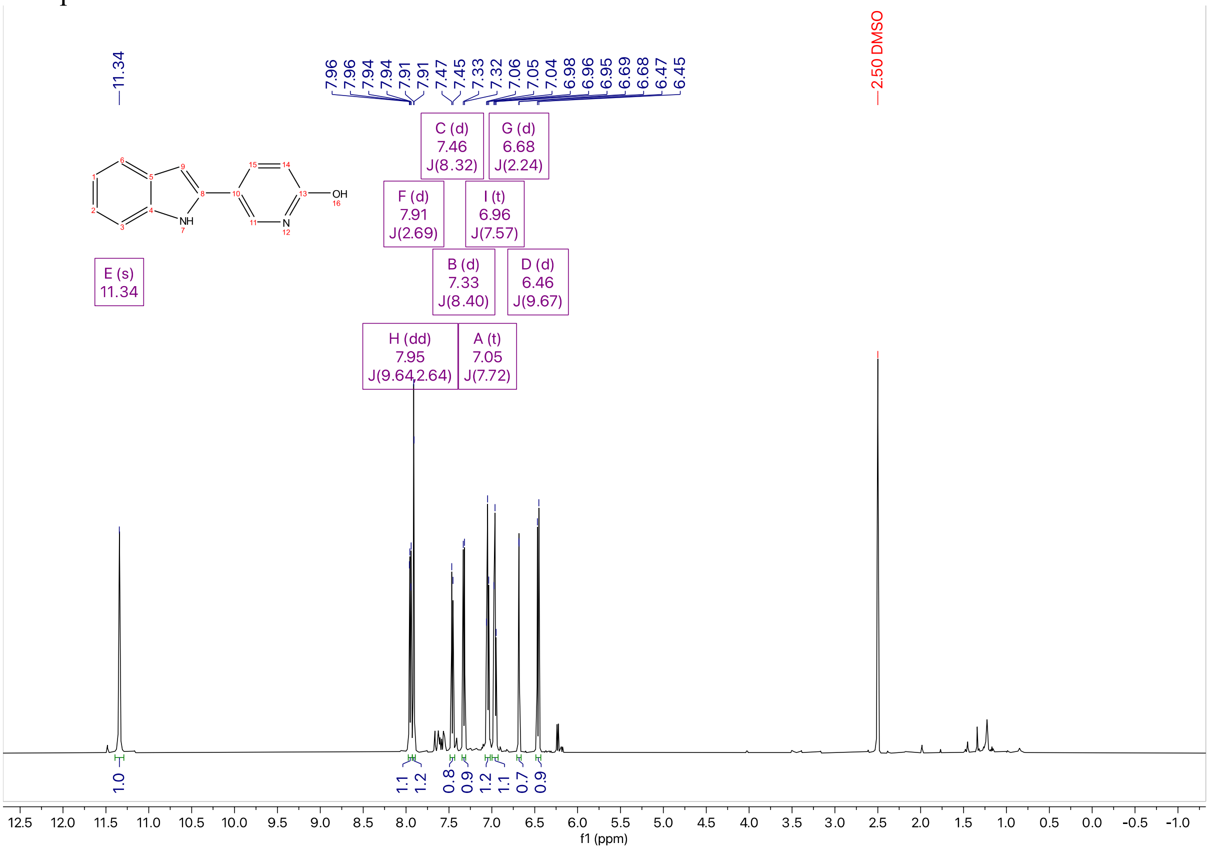

Compound 21 ^{13}C

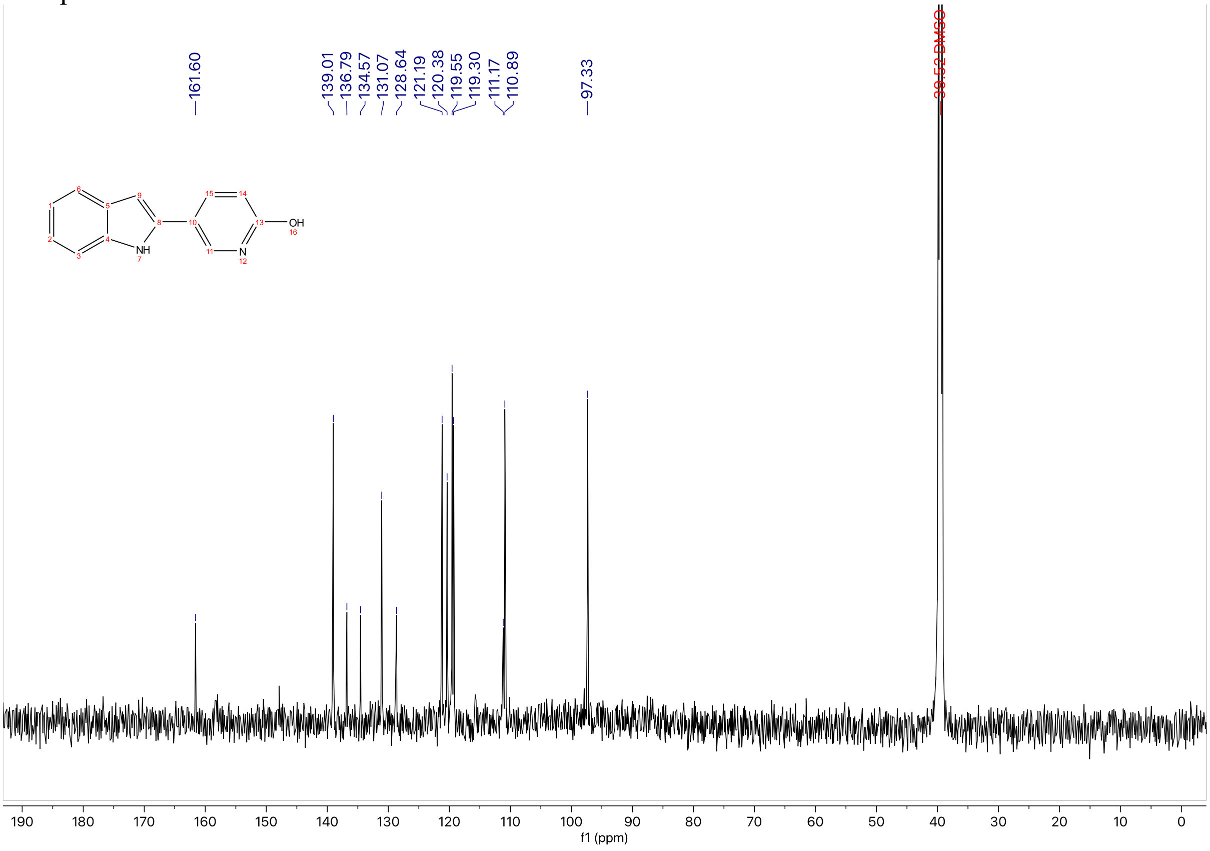

Compound 22 ^{1}H

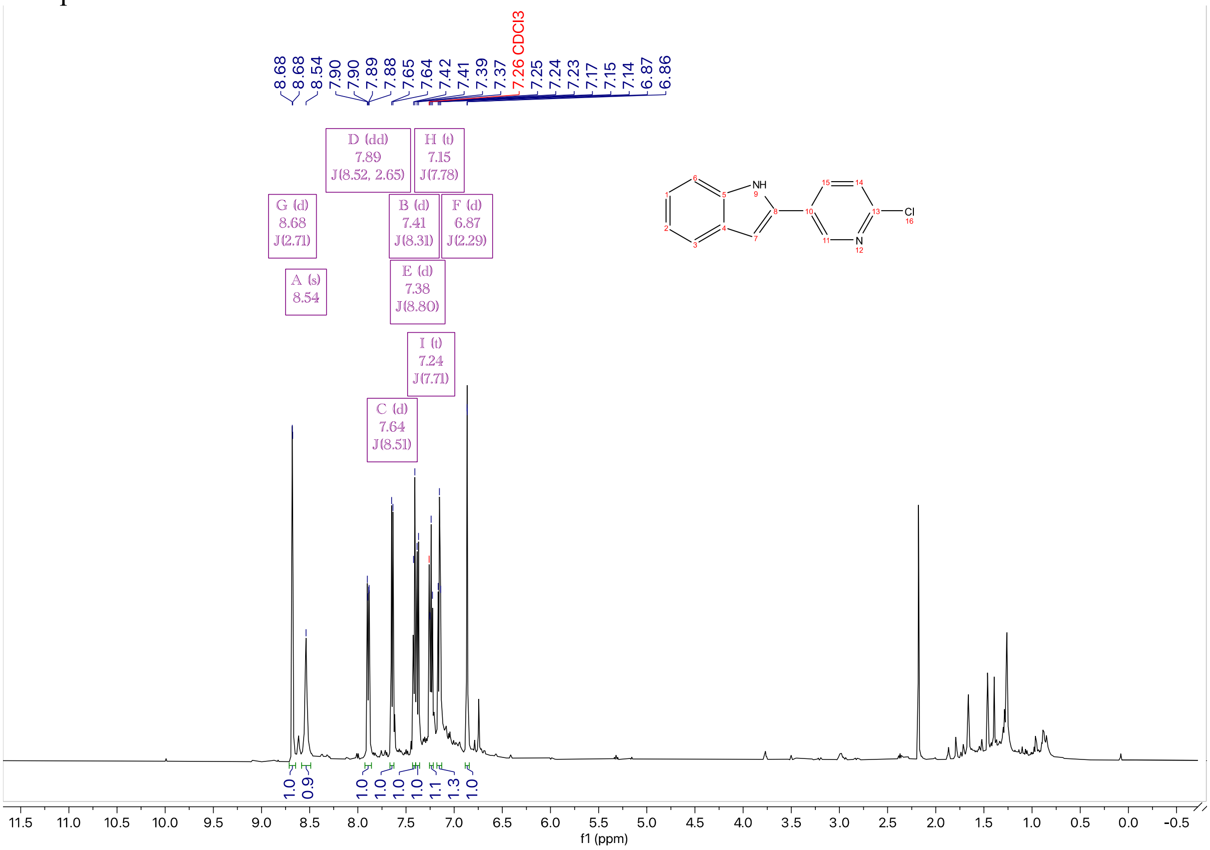

Compound 22 ^{13}C

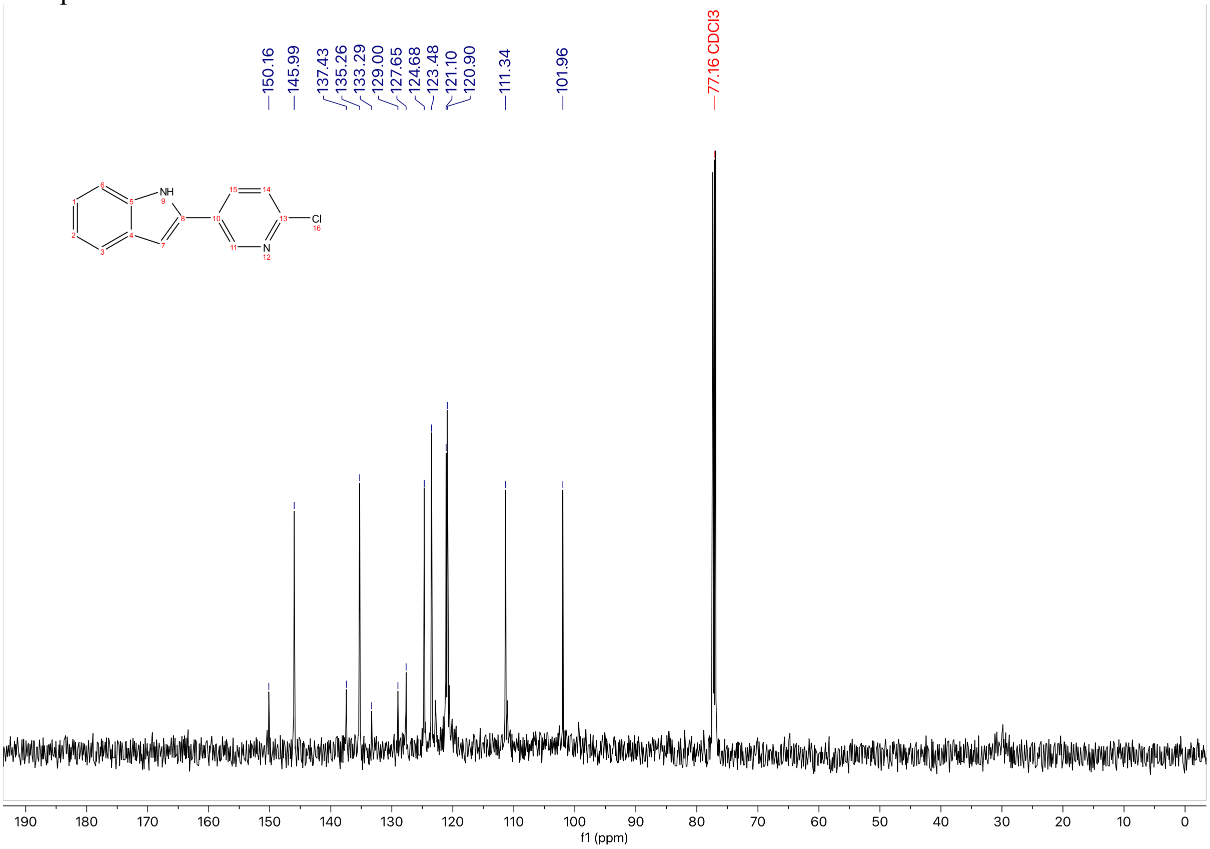

Compound 23 ^1H

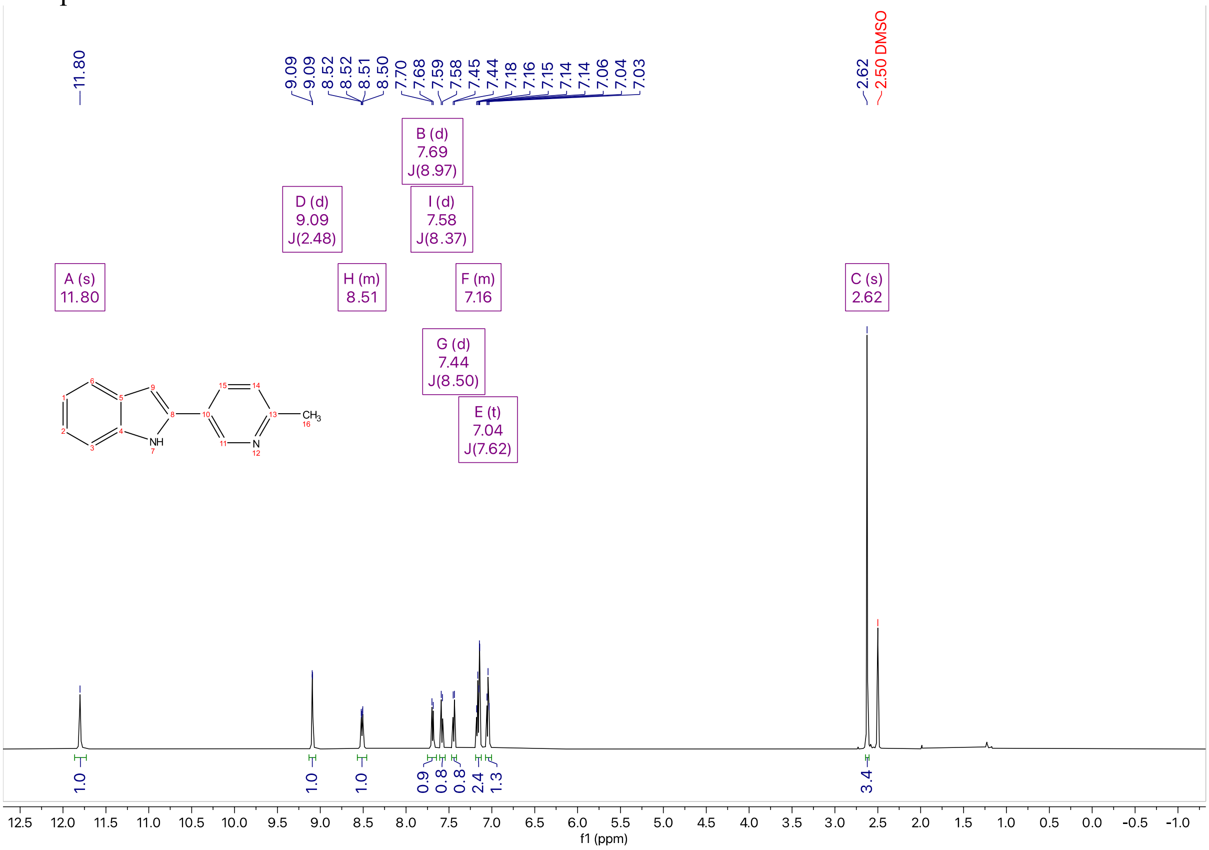

Compound 23 ^{13}C

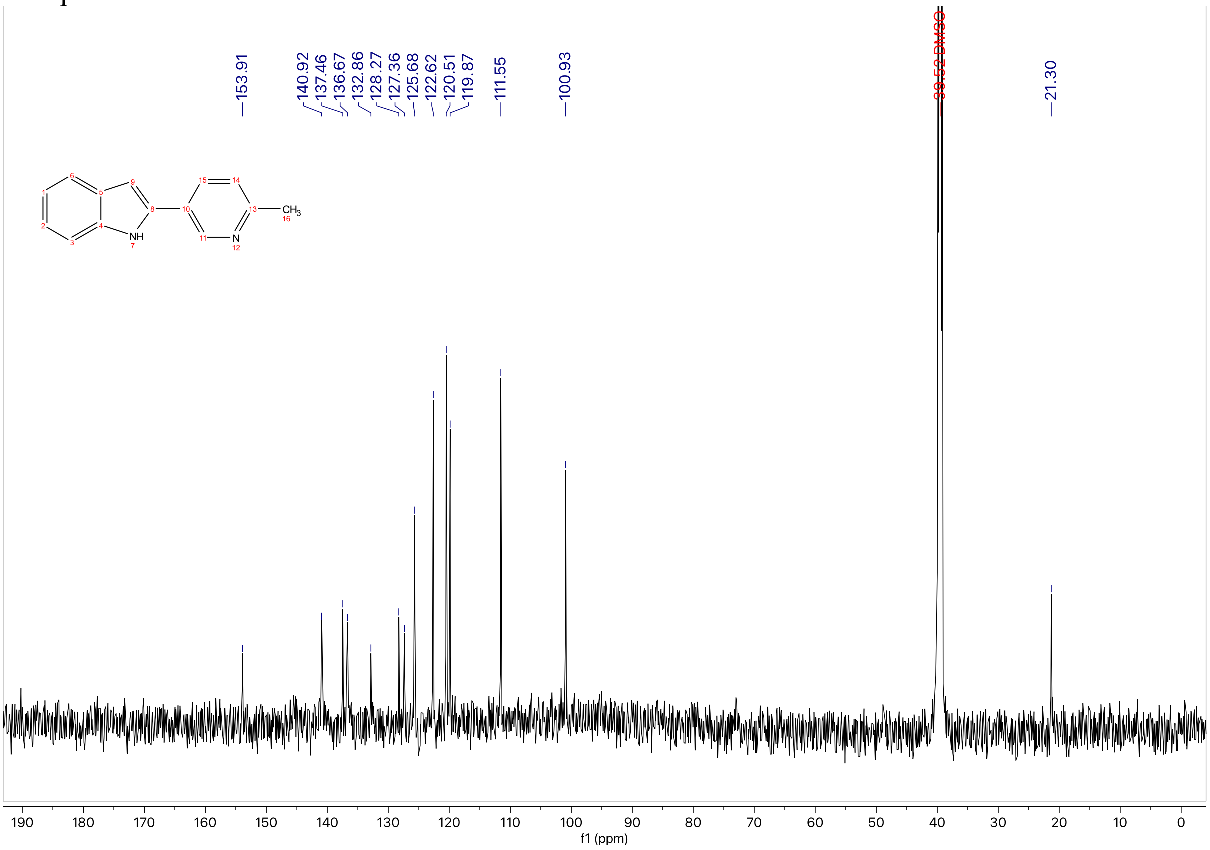

Compound 24 ^{1}H

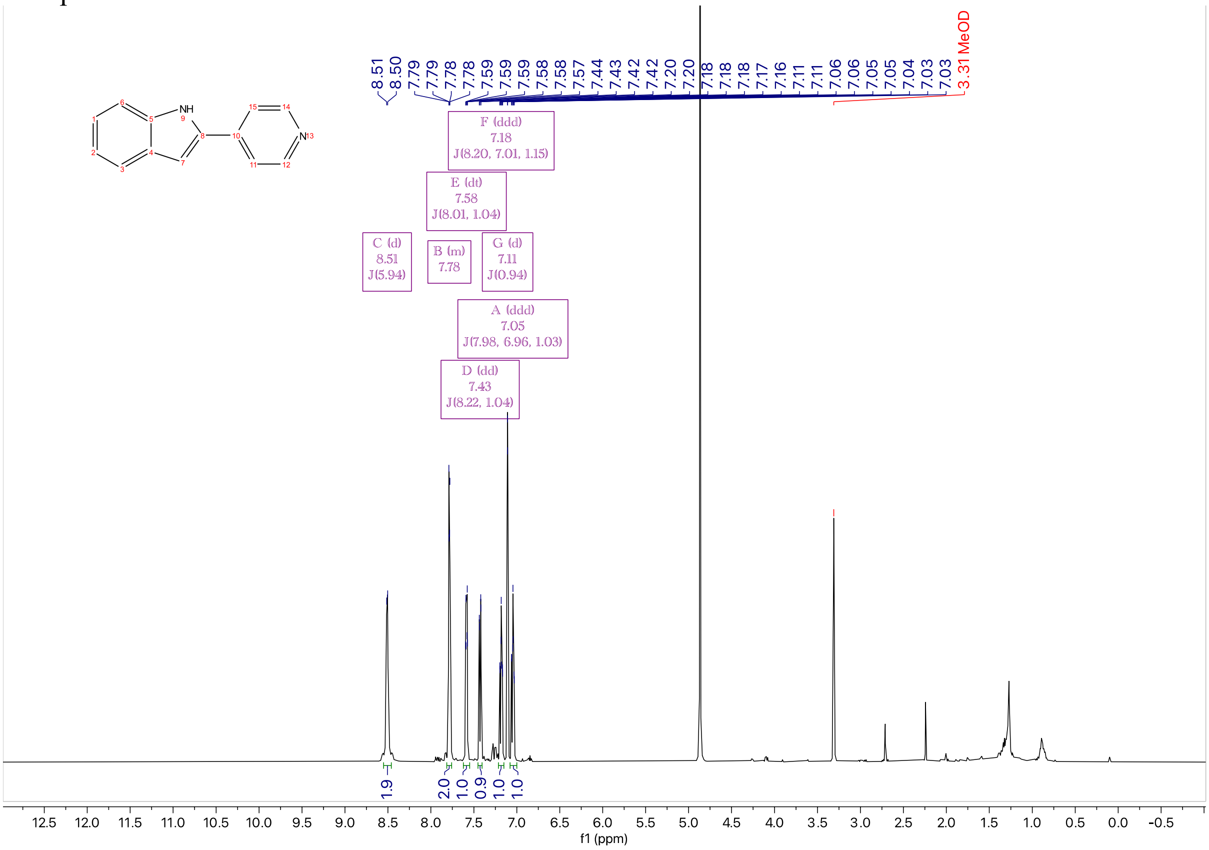

Compound 24 ^{13}C

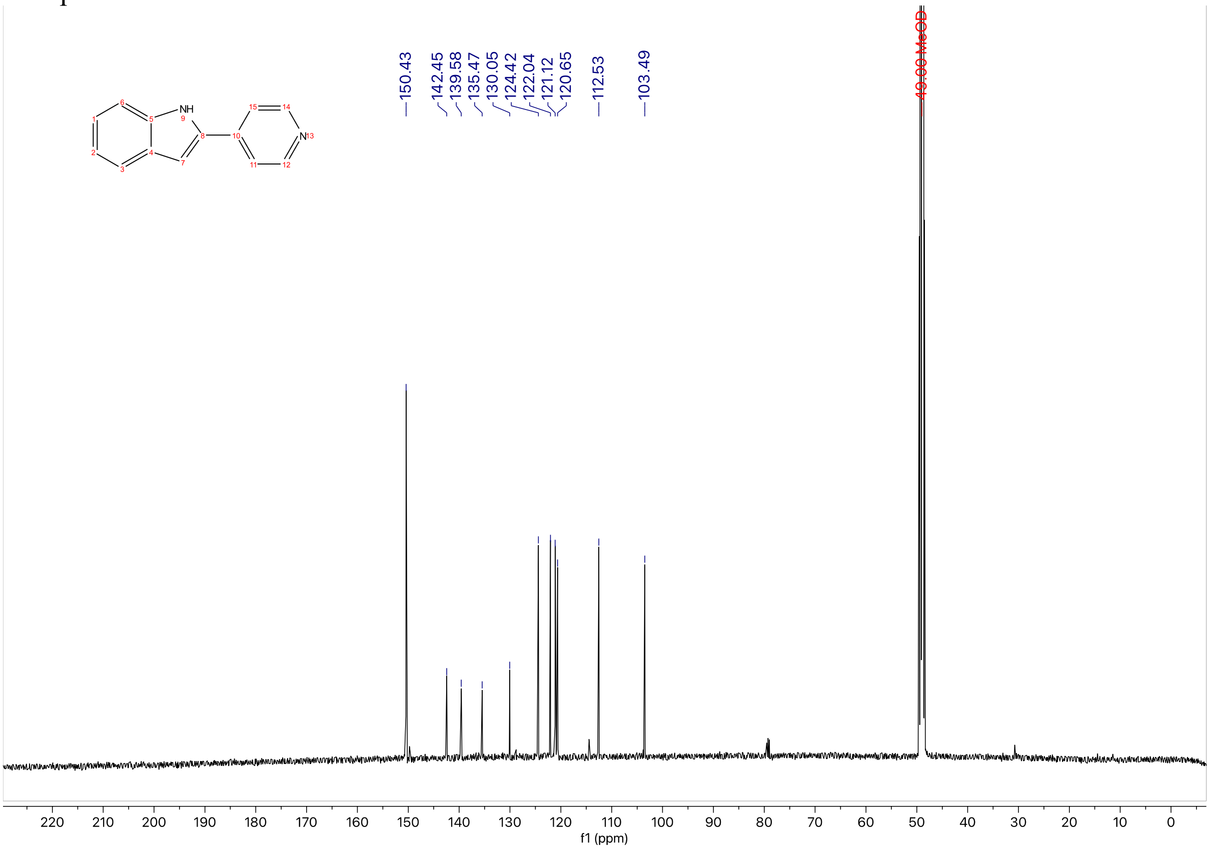

Compound 25 ^{1}H

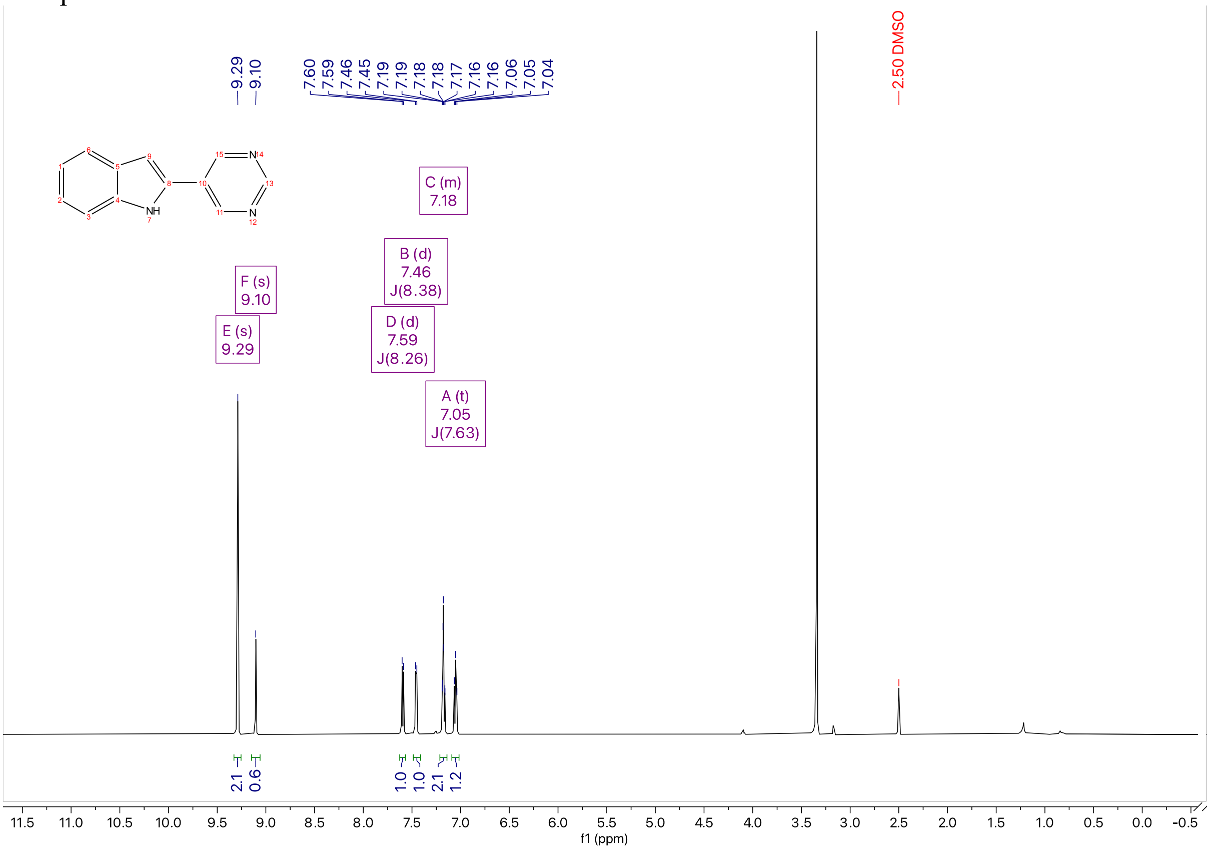

Compound 25 ^{13}C

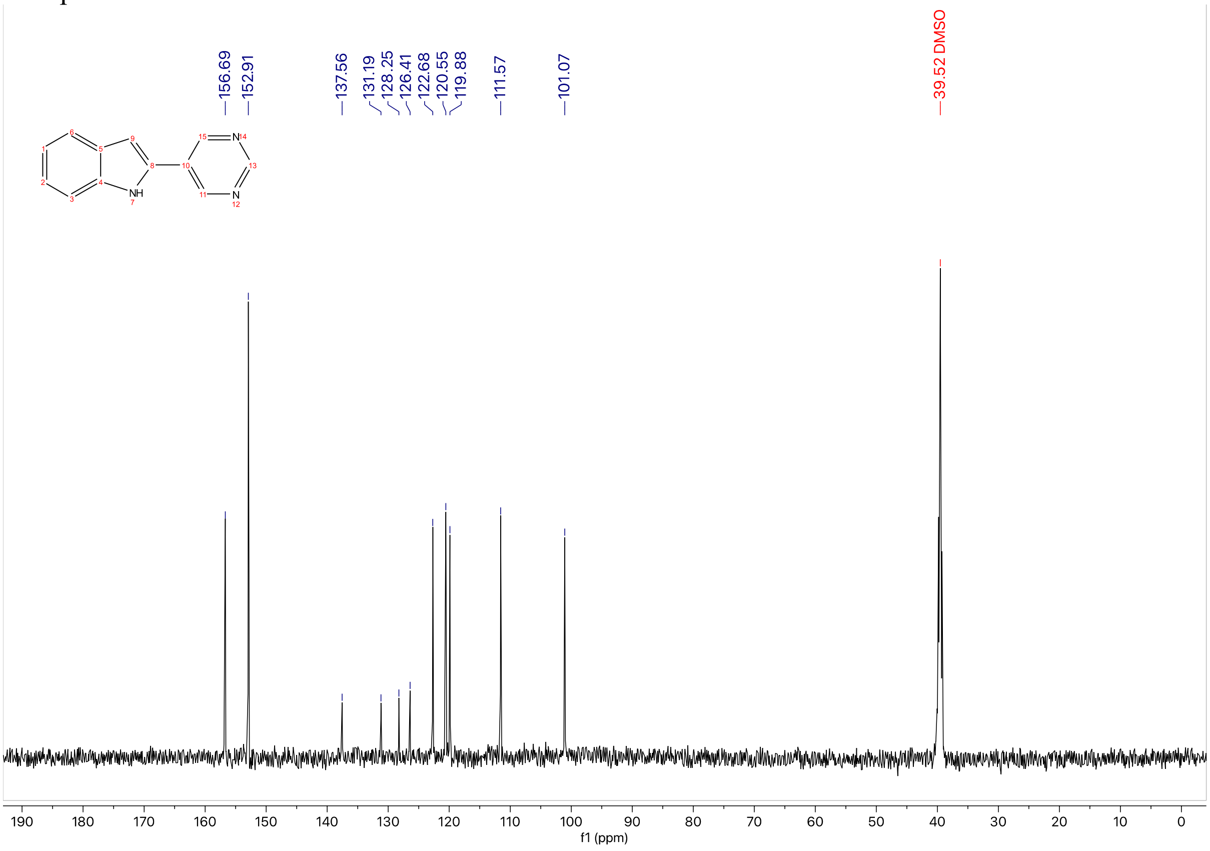

Compound 26 ^{1}H

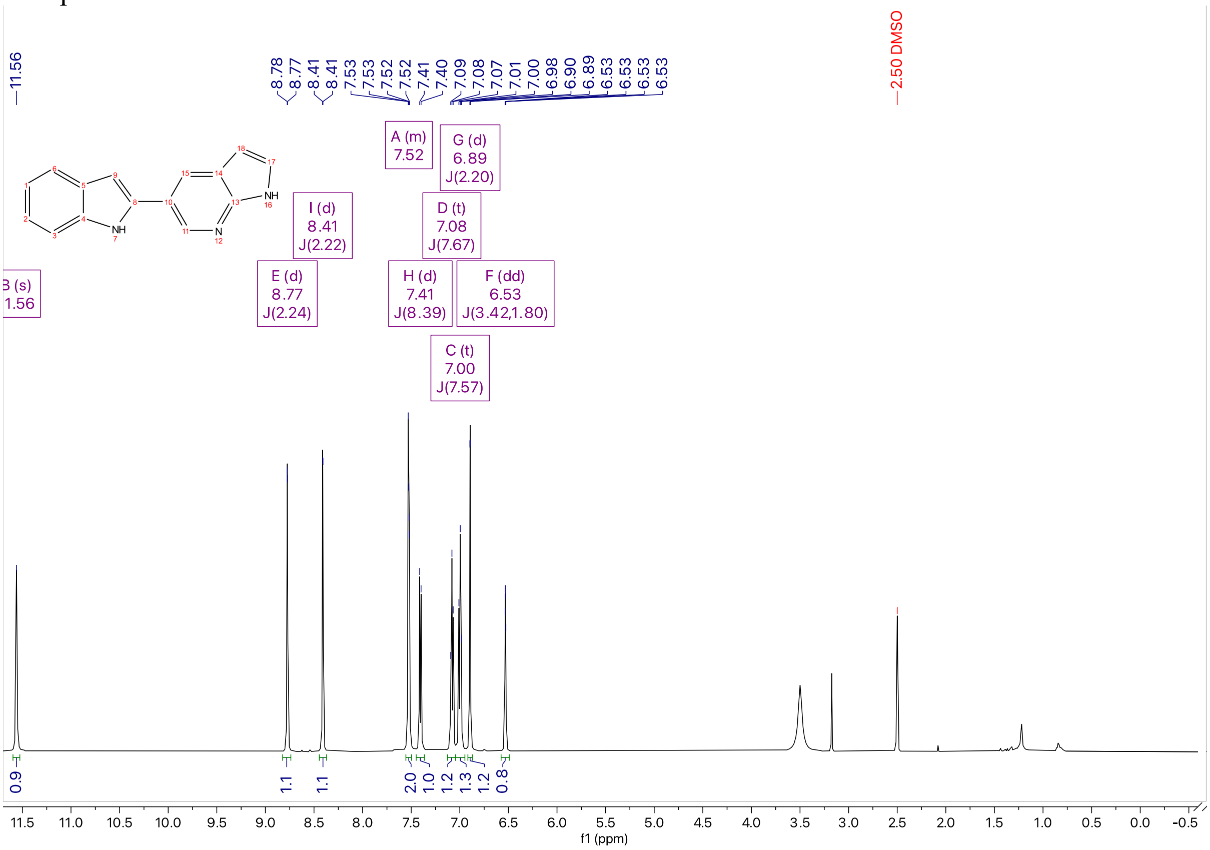

Compound 26 ^{13}C

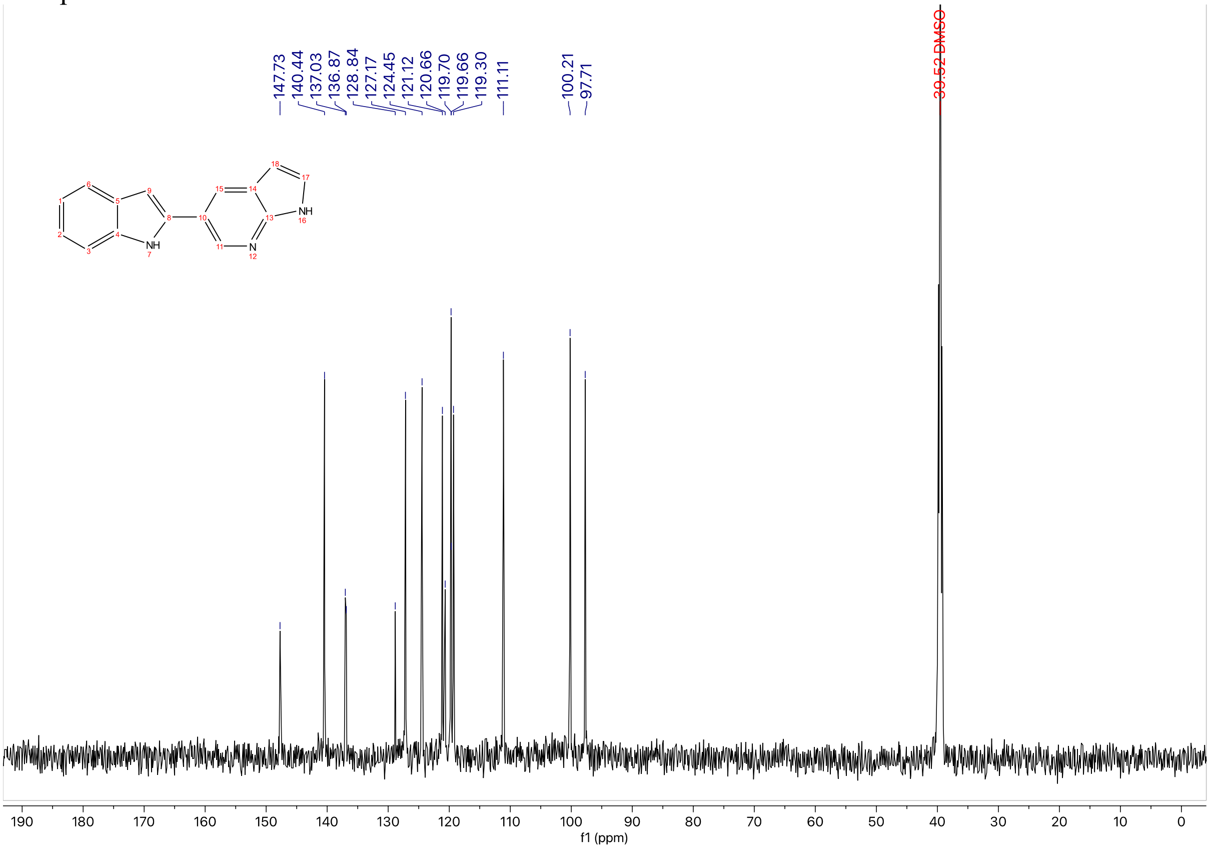

Compound 27 ^{1}H

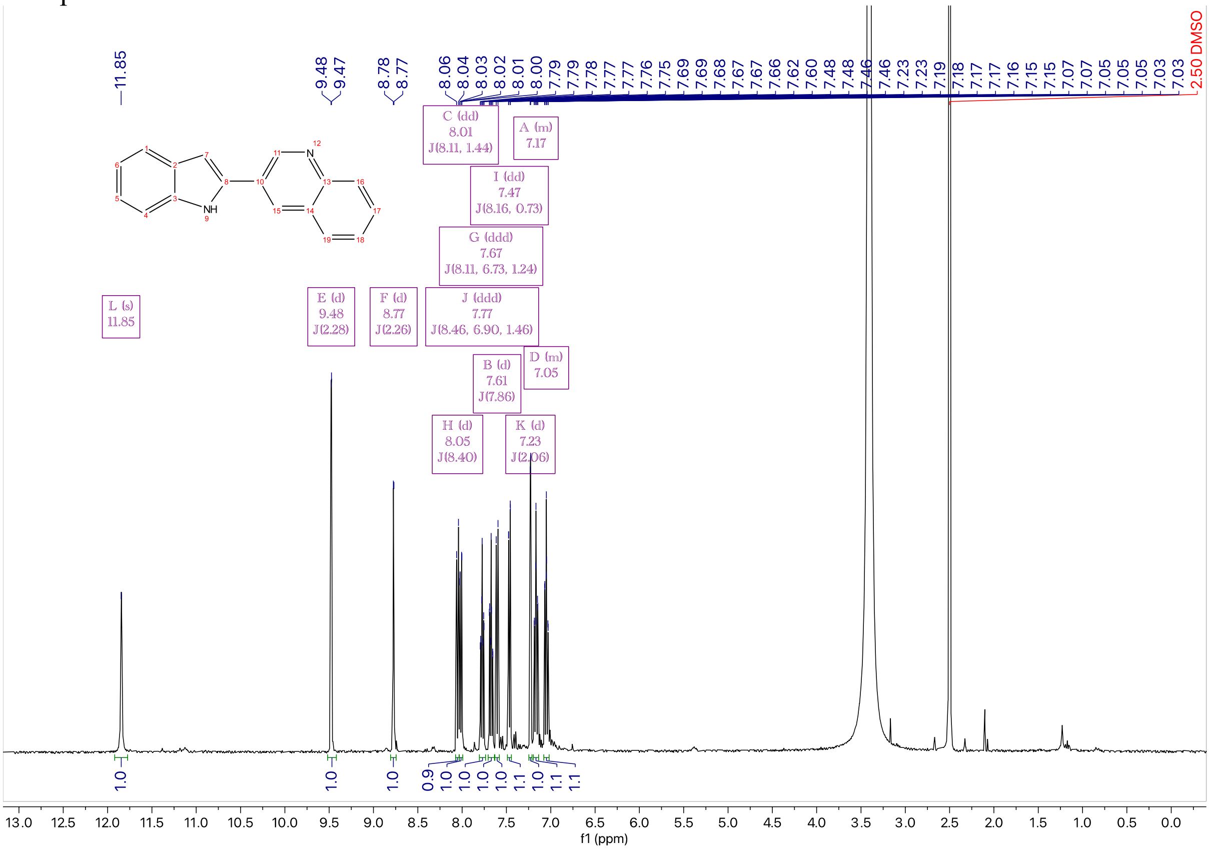

Compound 27 ^{13}C

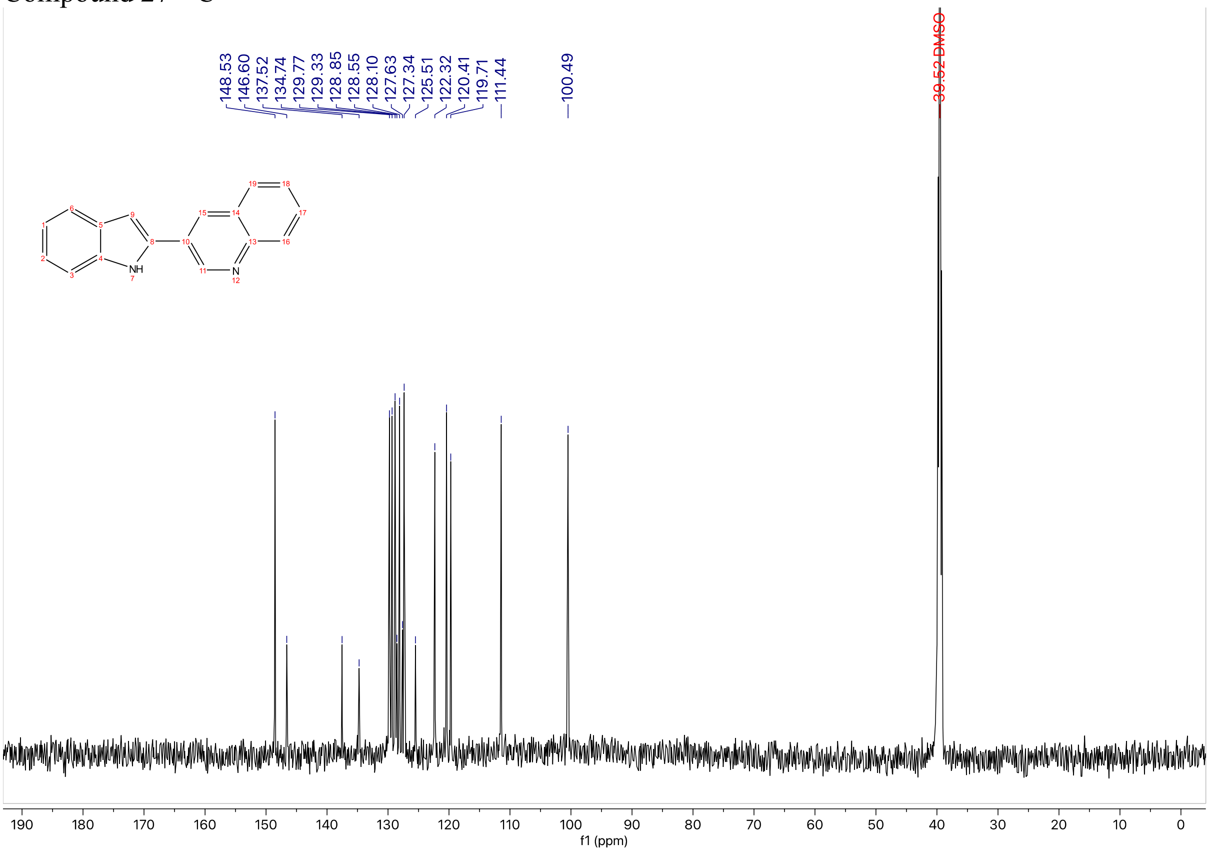

Compound 28 ^{1}H

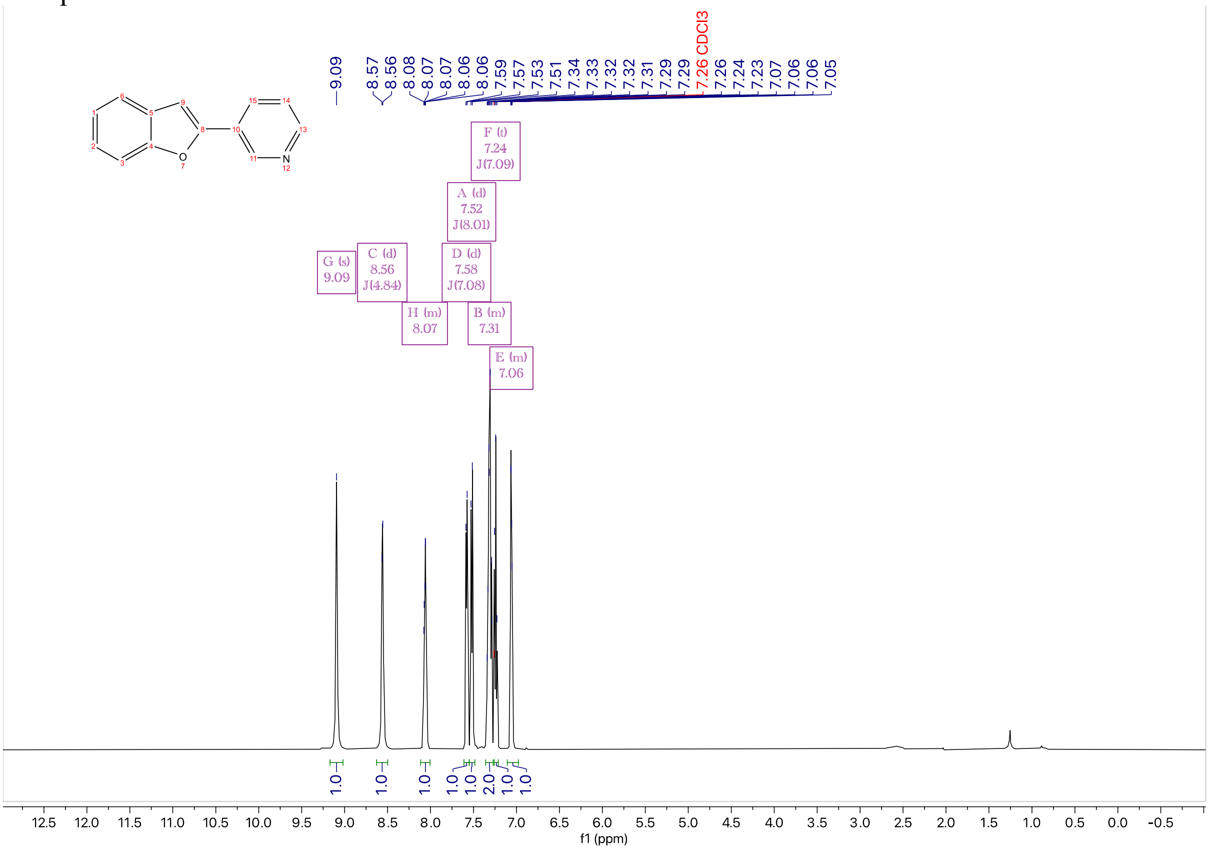

Compound 28 ^{13}C

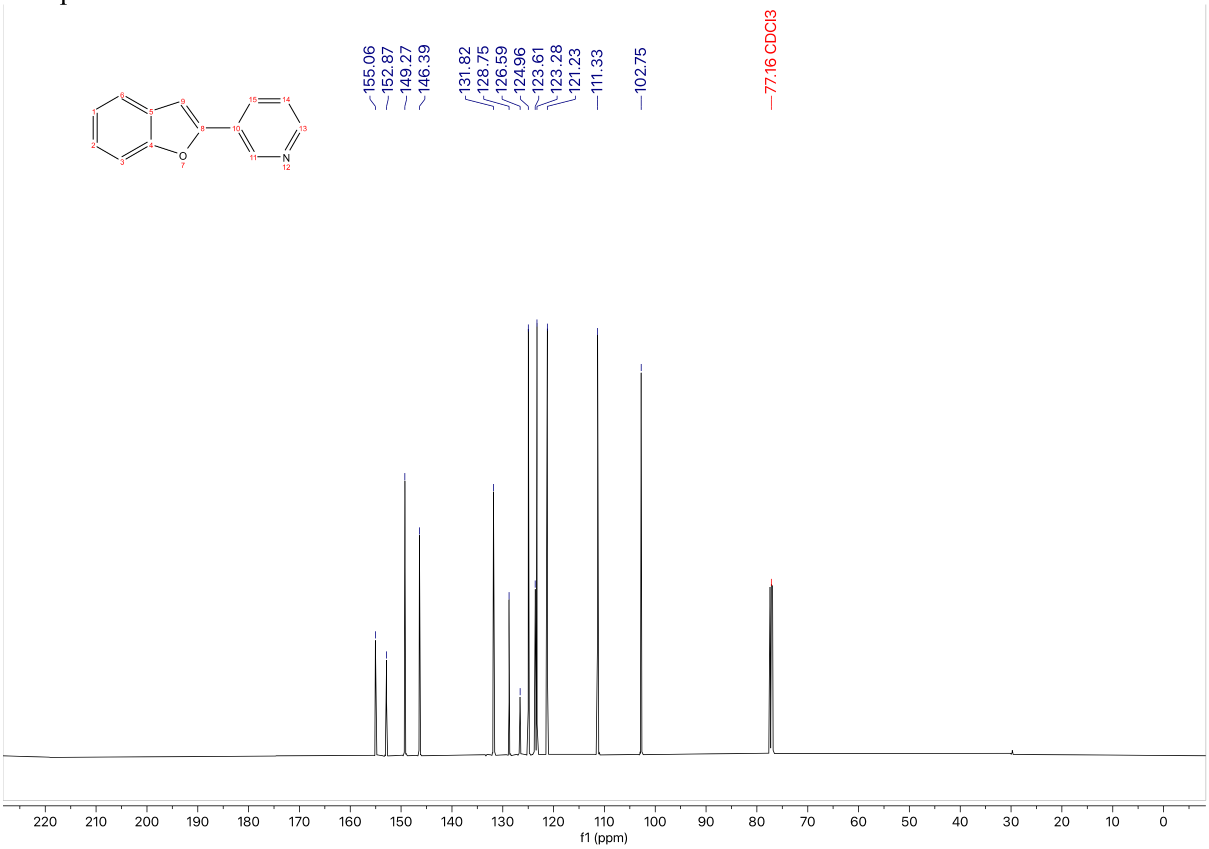

Compound 30 ^{1}H

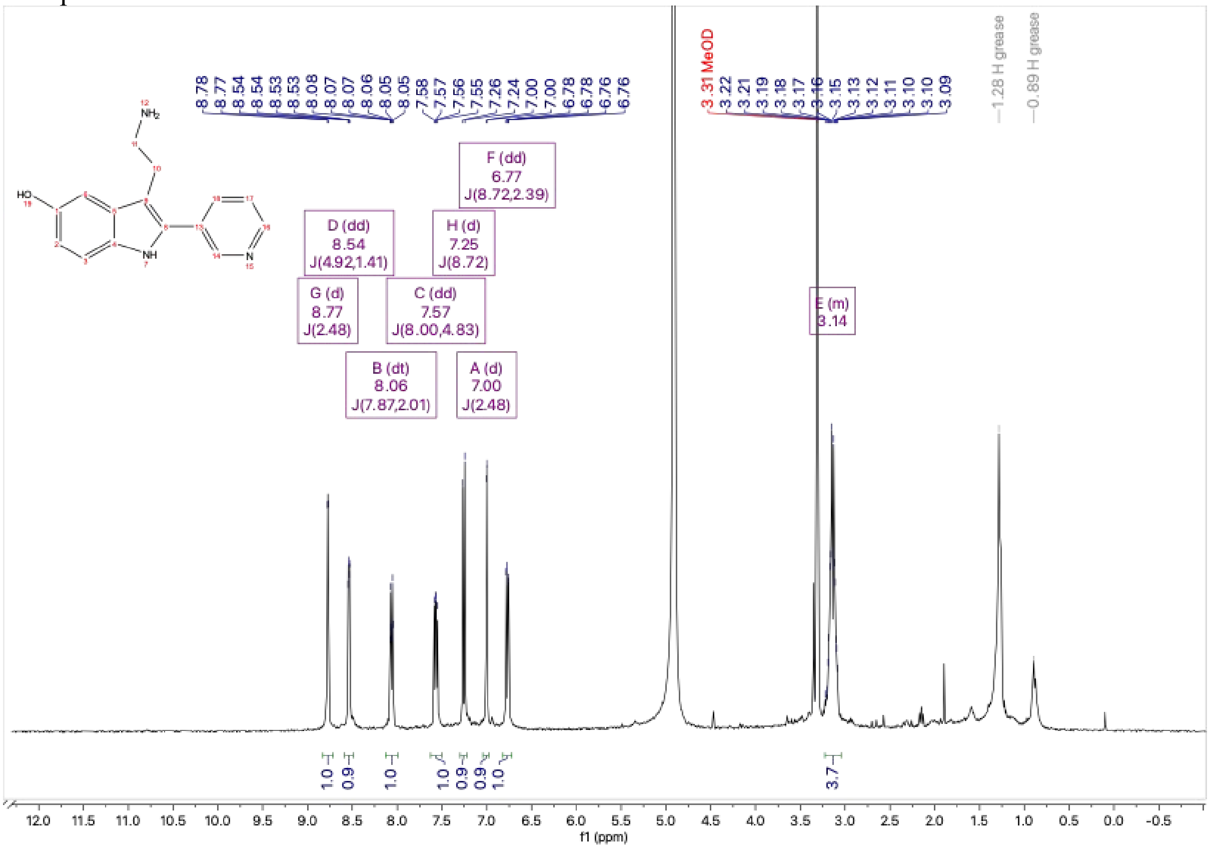

Compound 30 ^{13}C

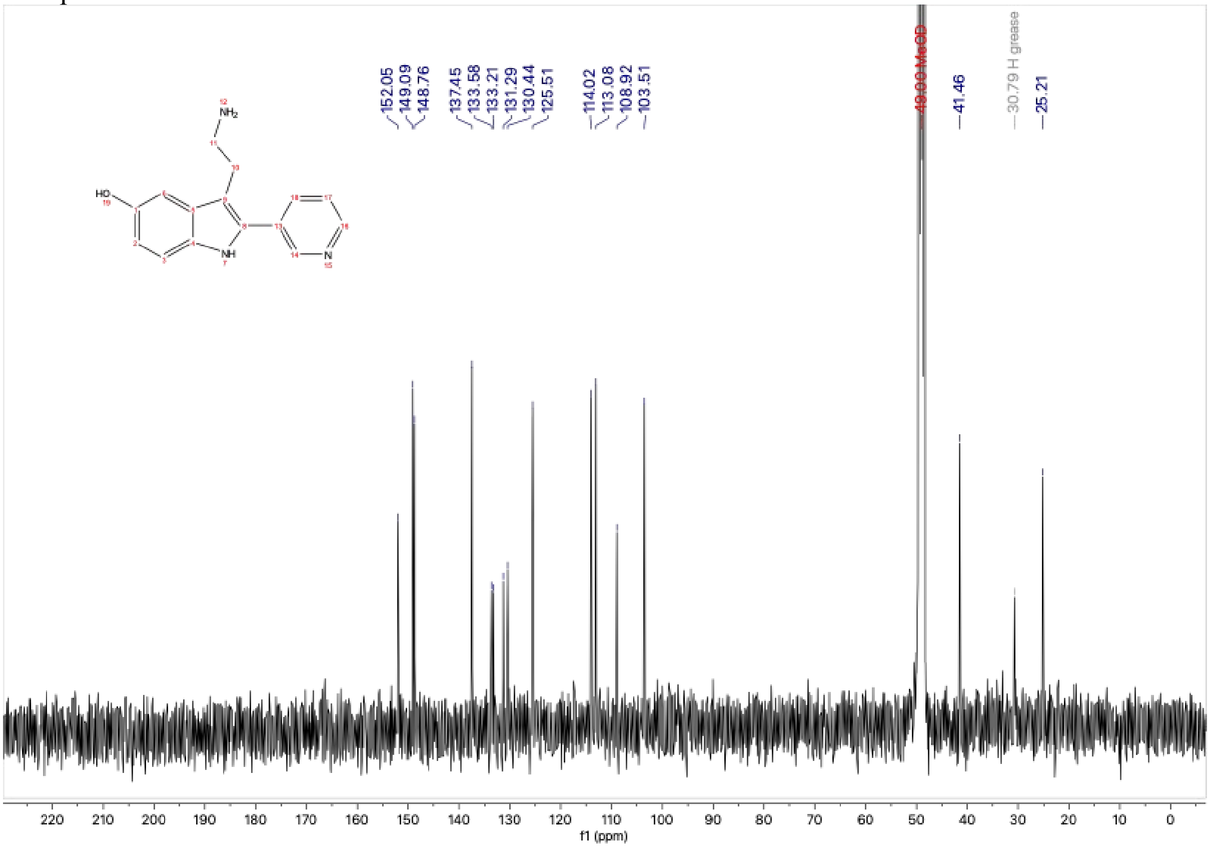

Compound 31 ^{1}H

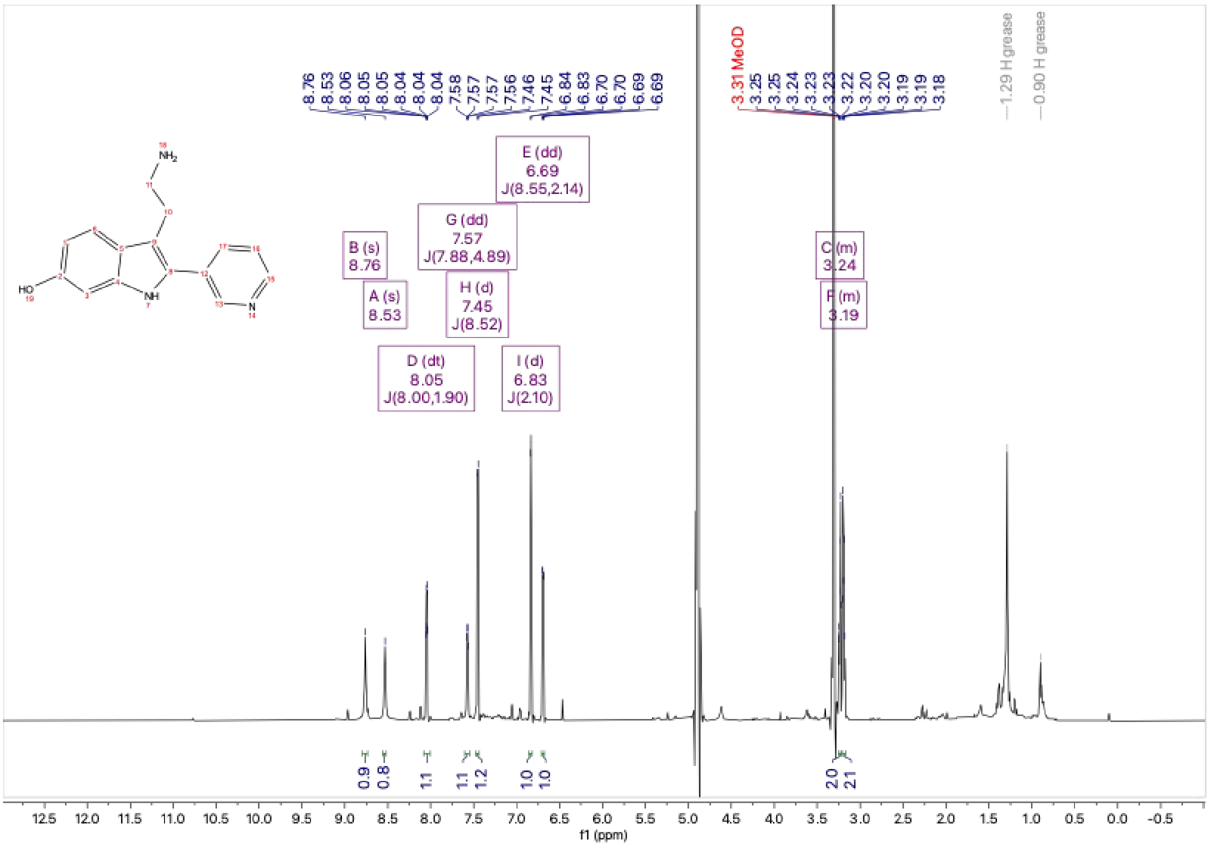

Compound 31 ^{13}C

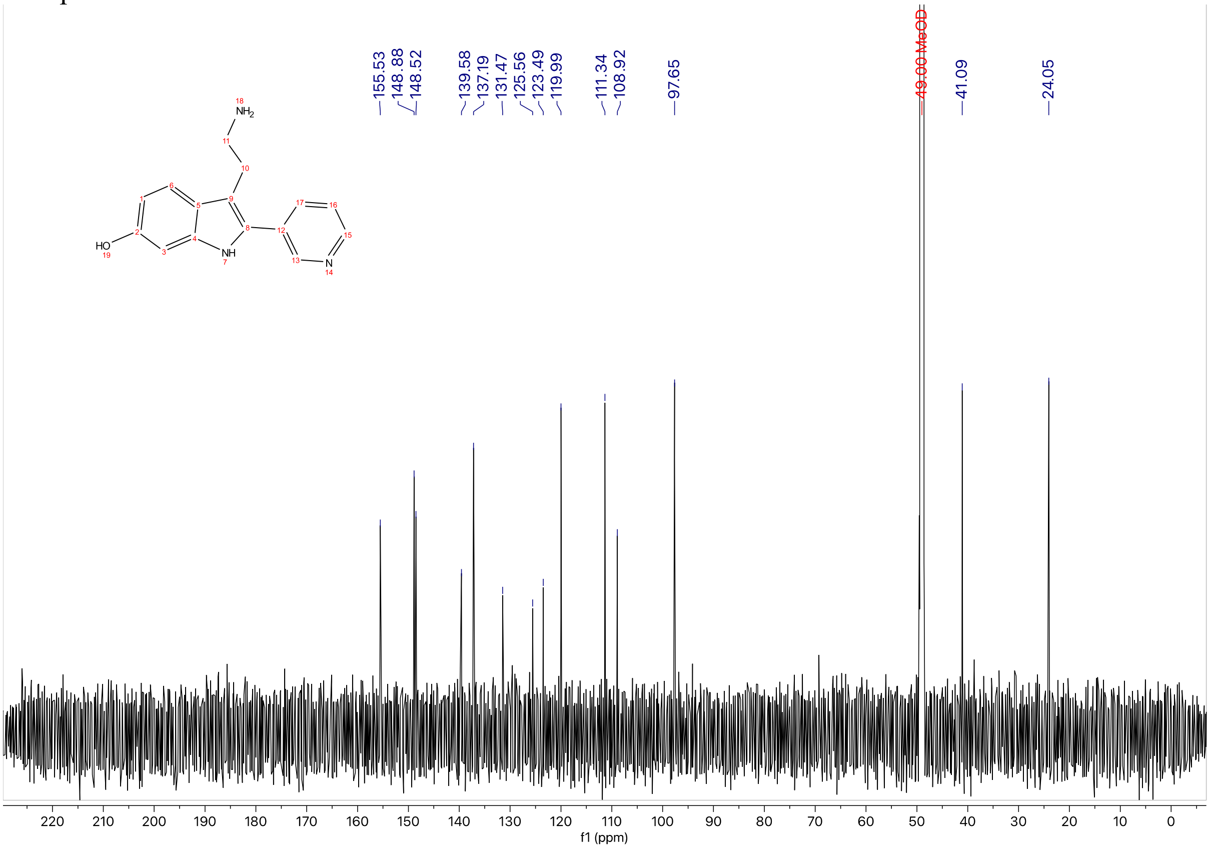

Compound 32 ^{1}H

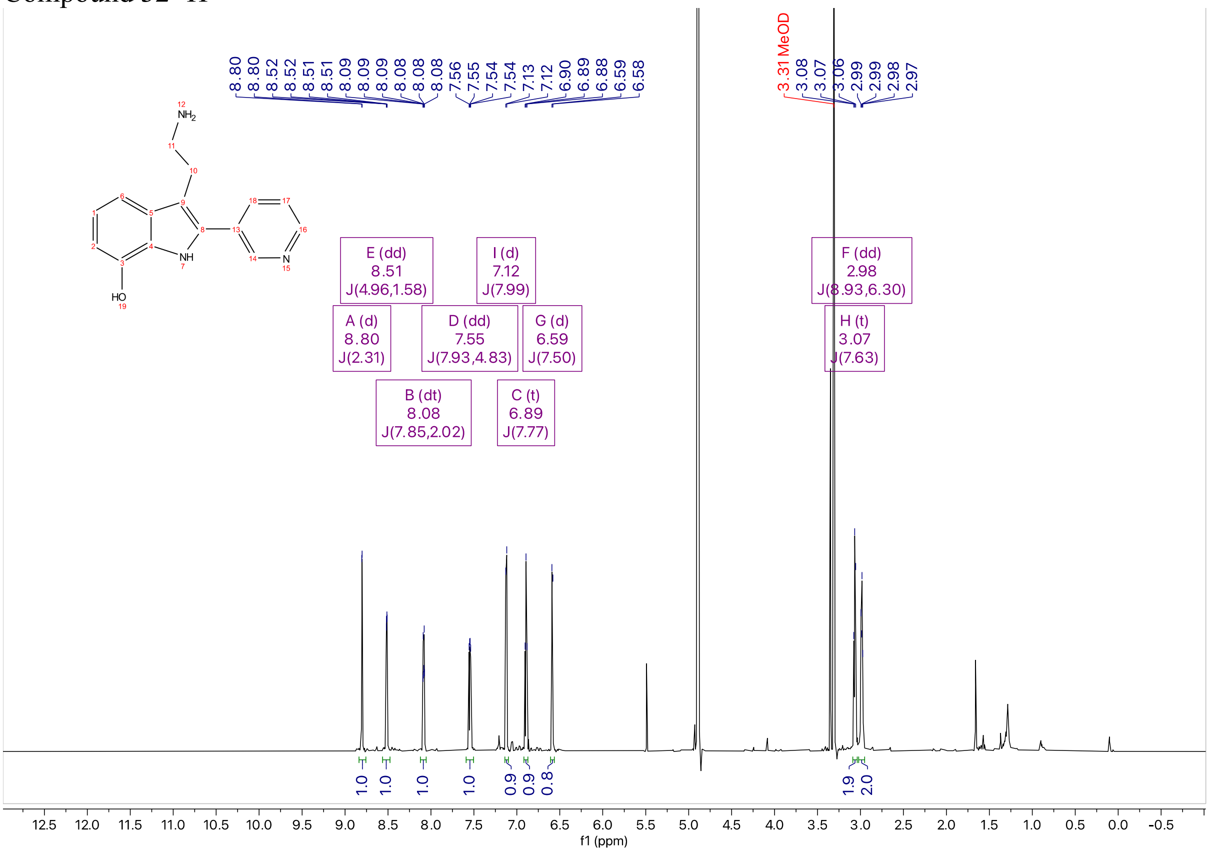

Compound 32 ^{13}C

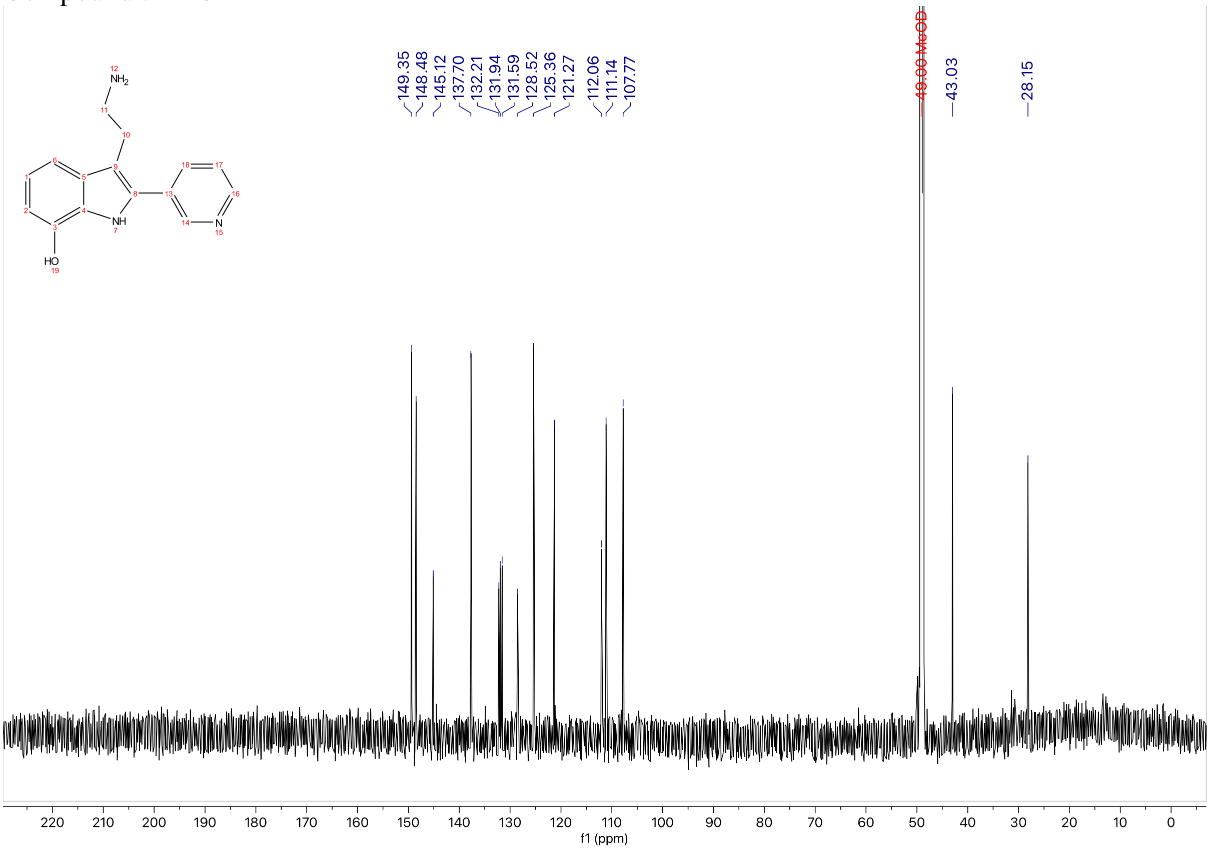

Compound 33 ^{1}H

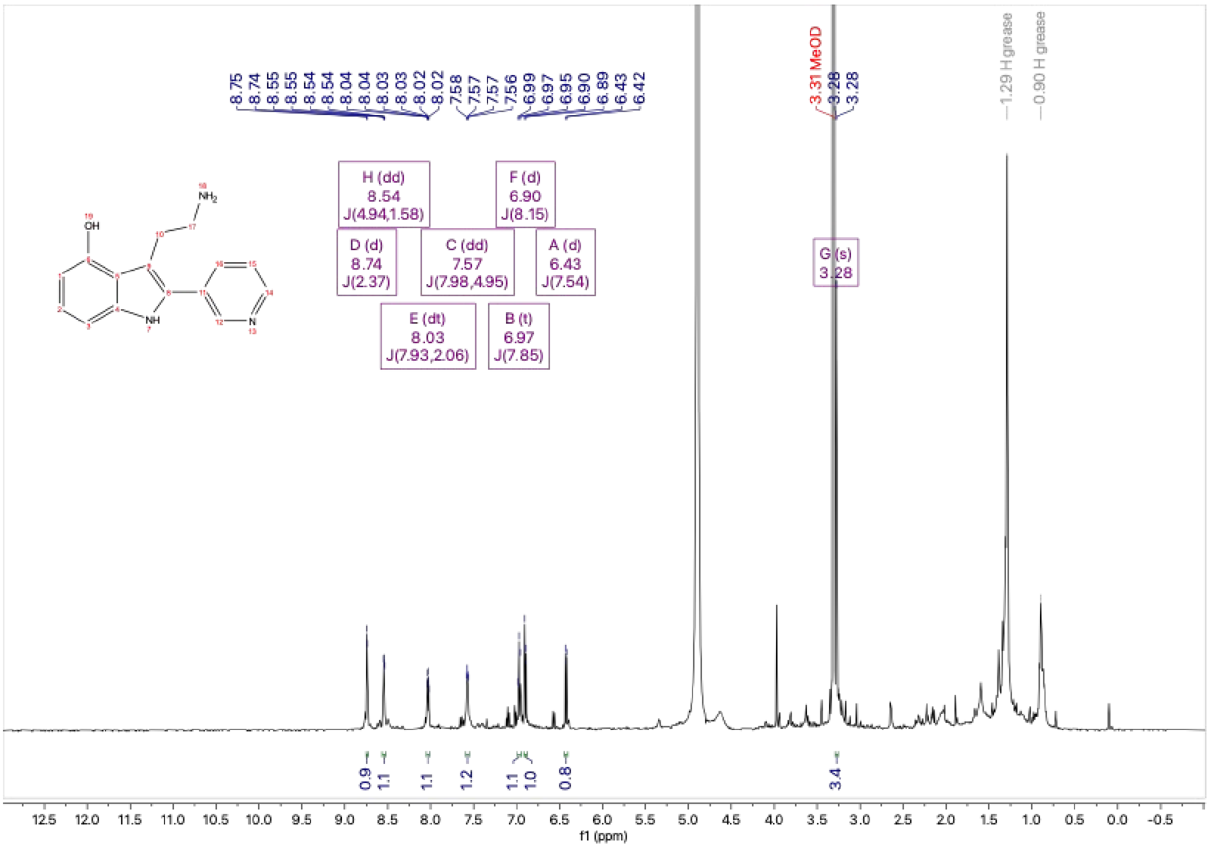

Compound 33 ^{13}C

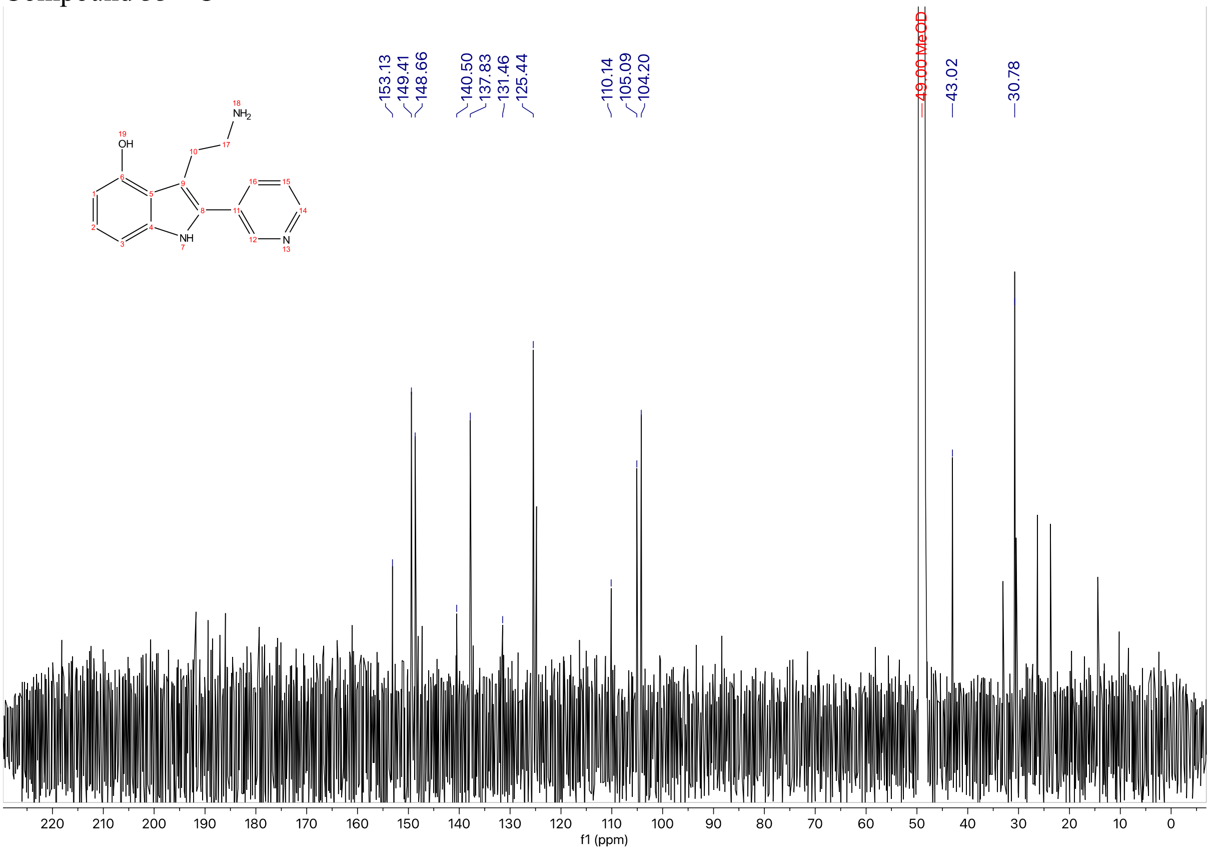

Compound 34 ^{1}H

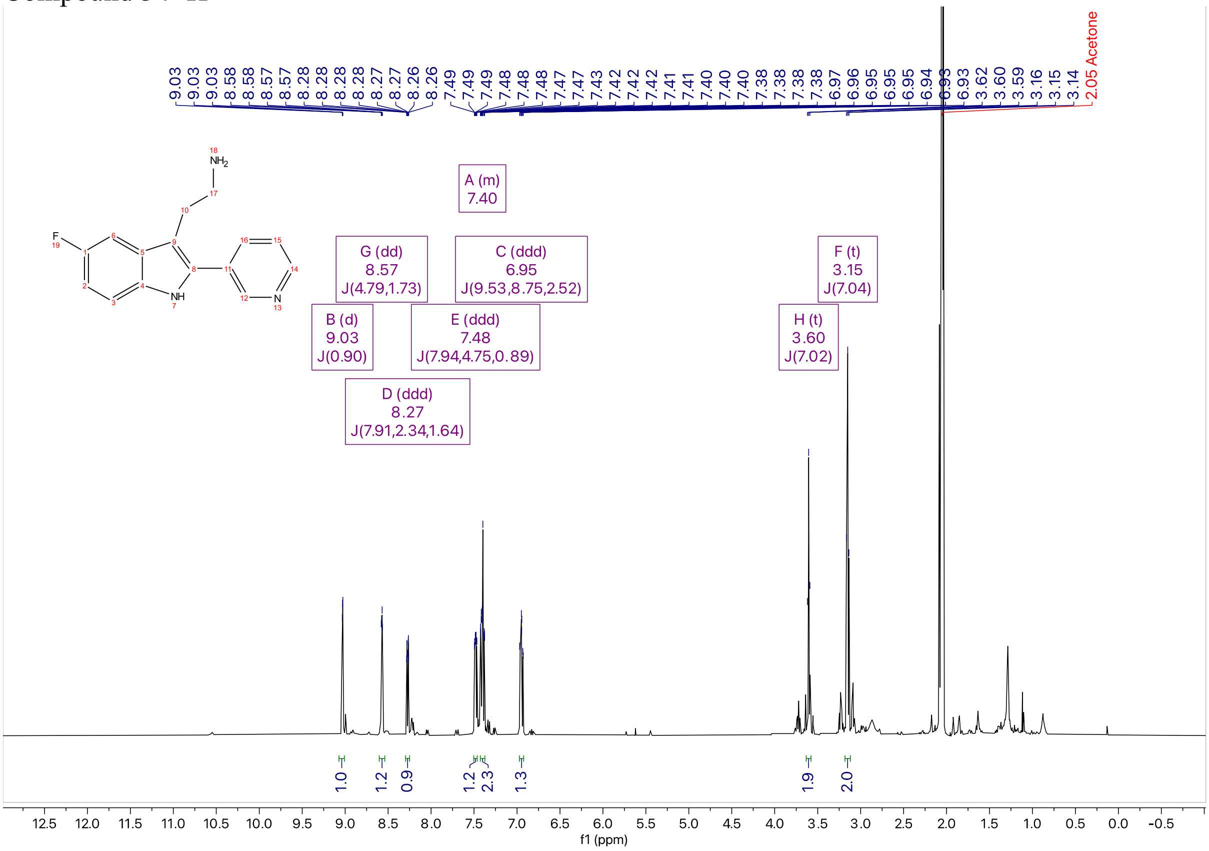

Compound 34 ^{13}C

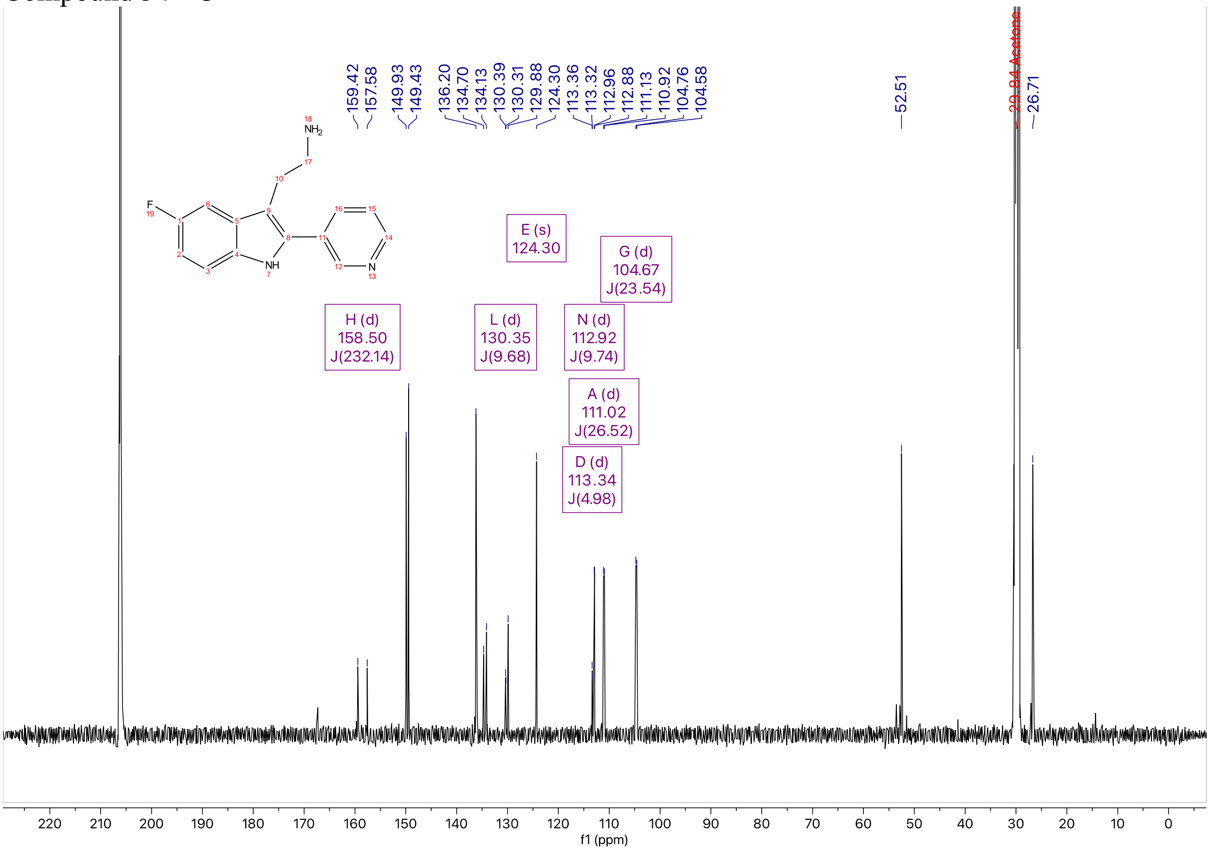

Compound 34 ^{19}F

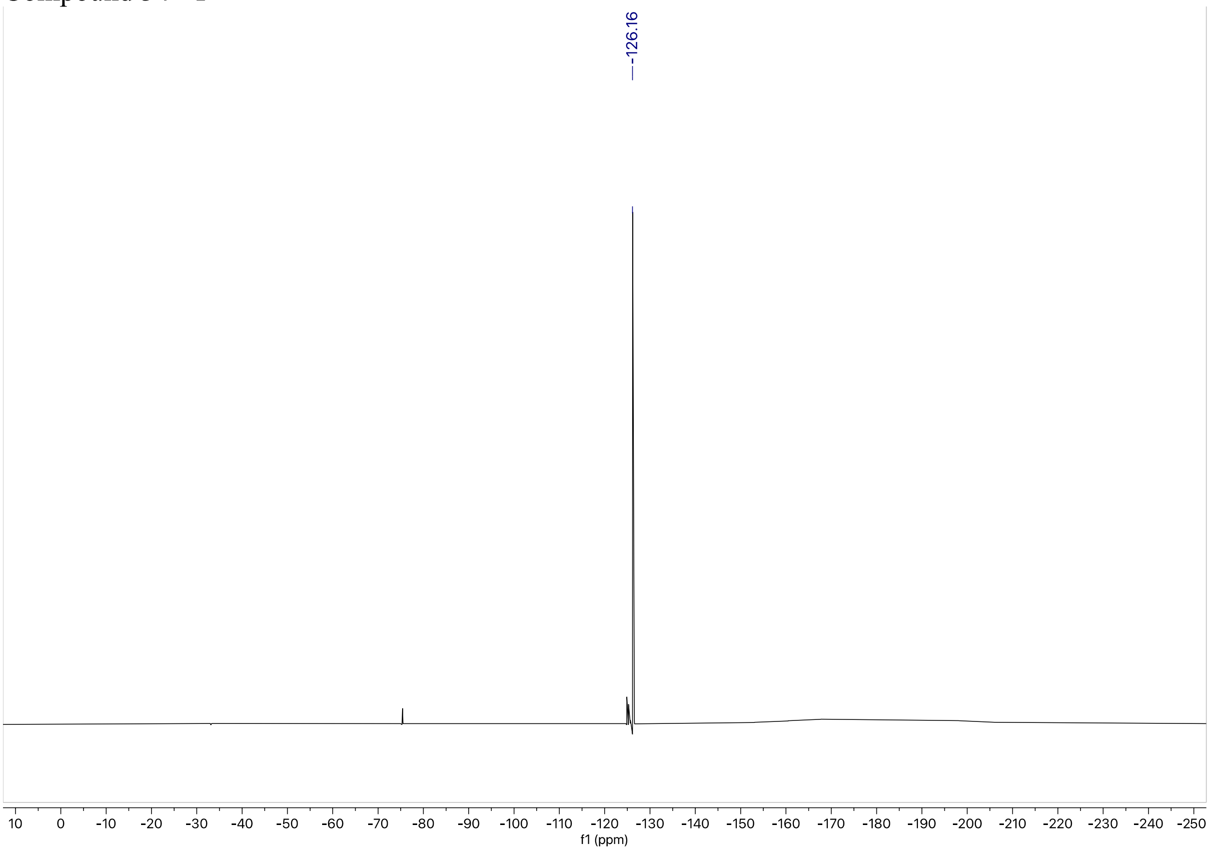

Compound 35 ^{1}H

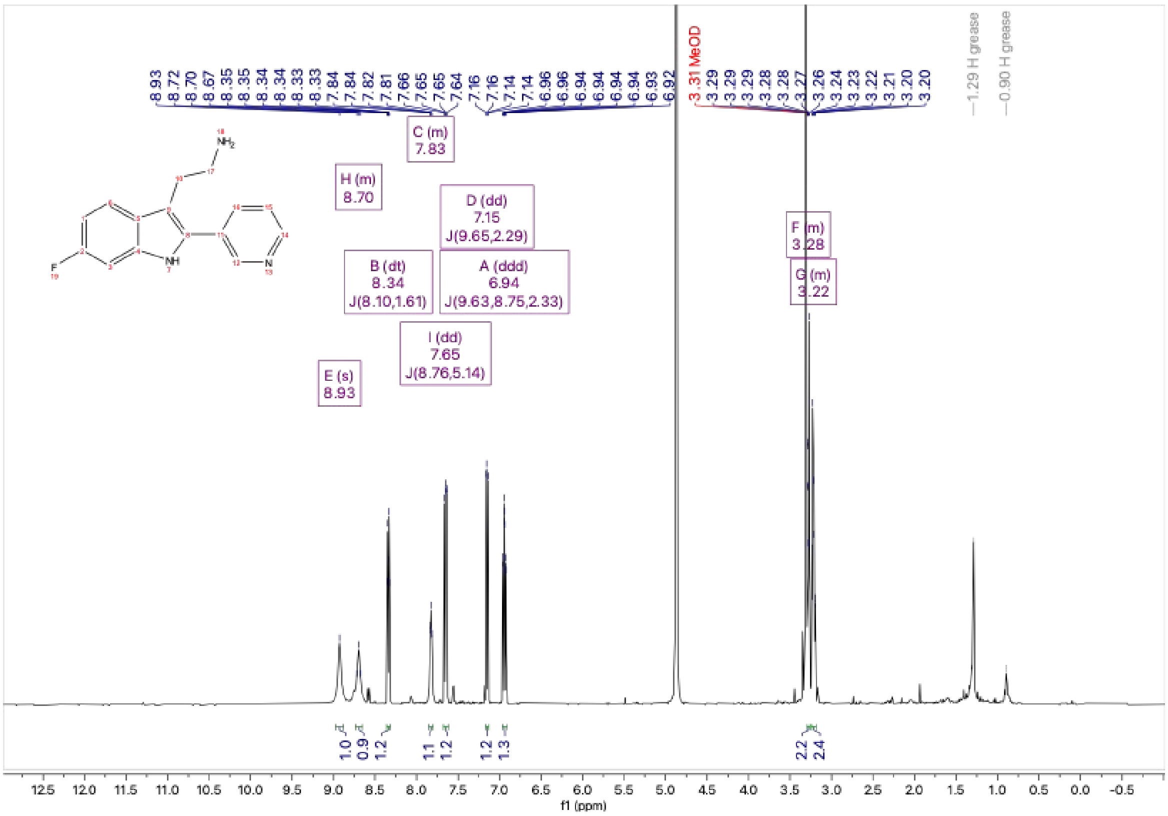

Compound 35 ^{13}C

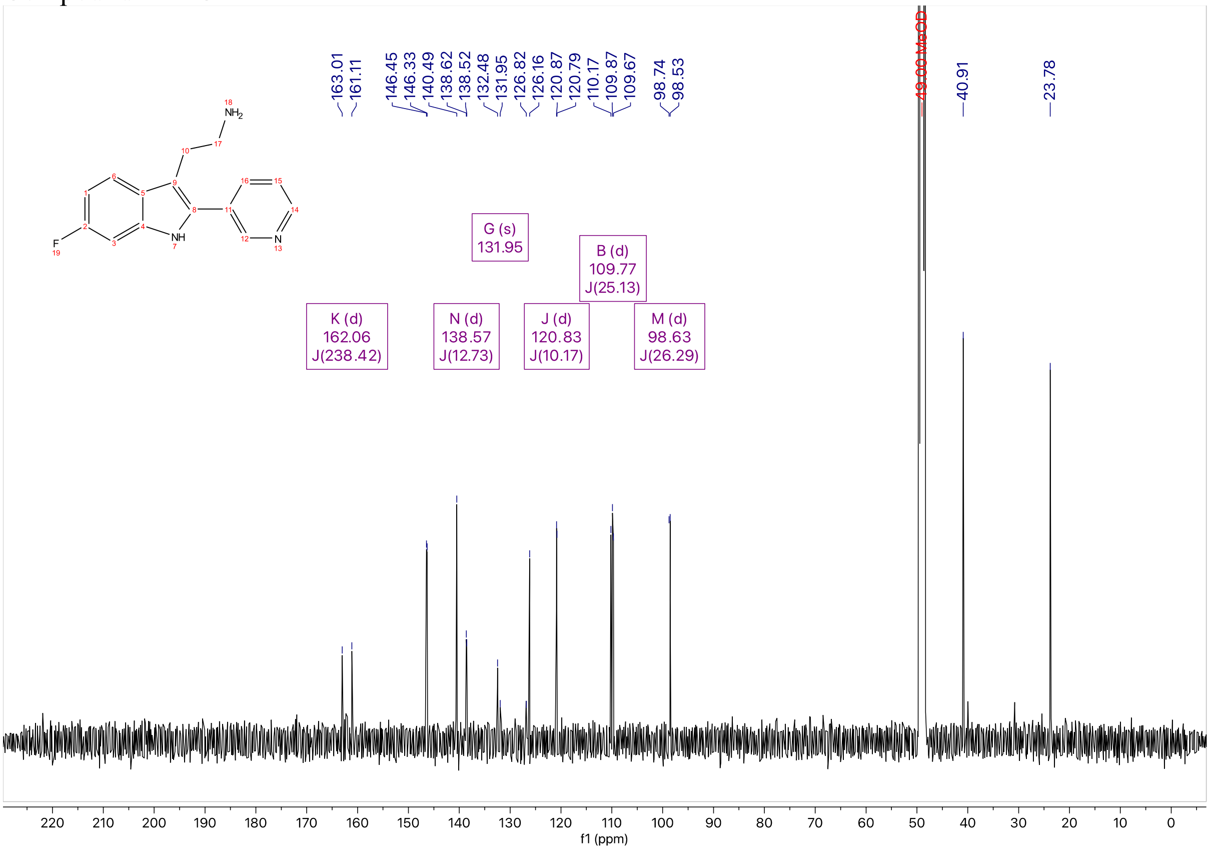

Compound 35 ^{19}F

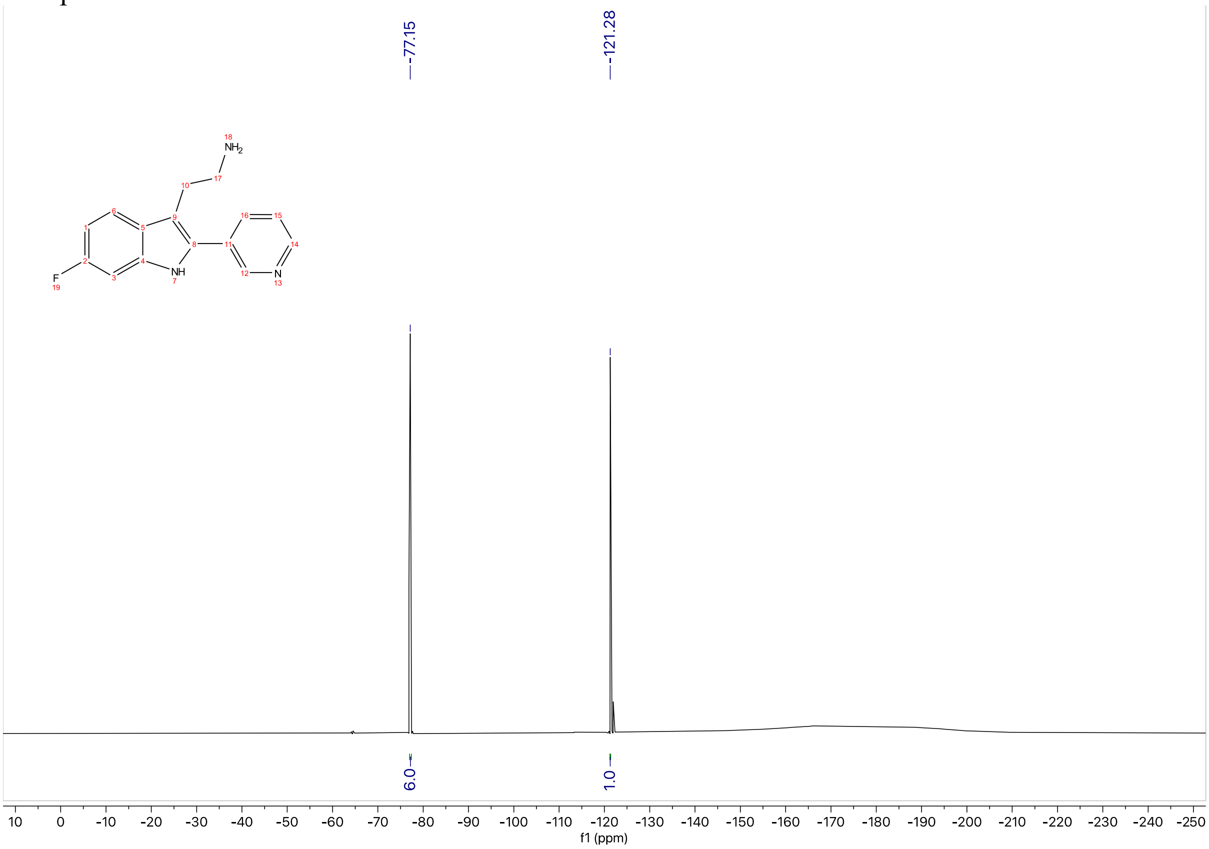

Compound 36 ^{1}H

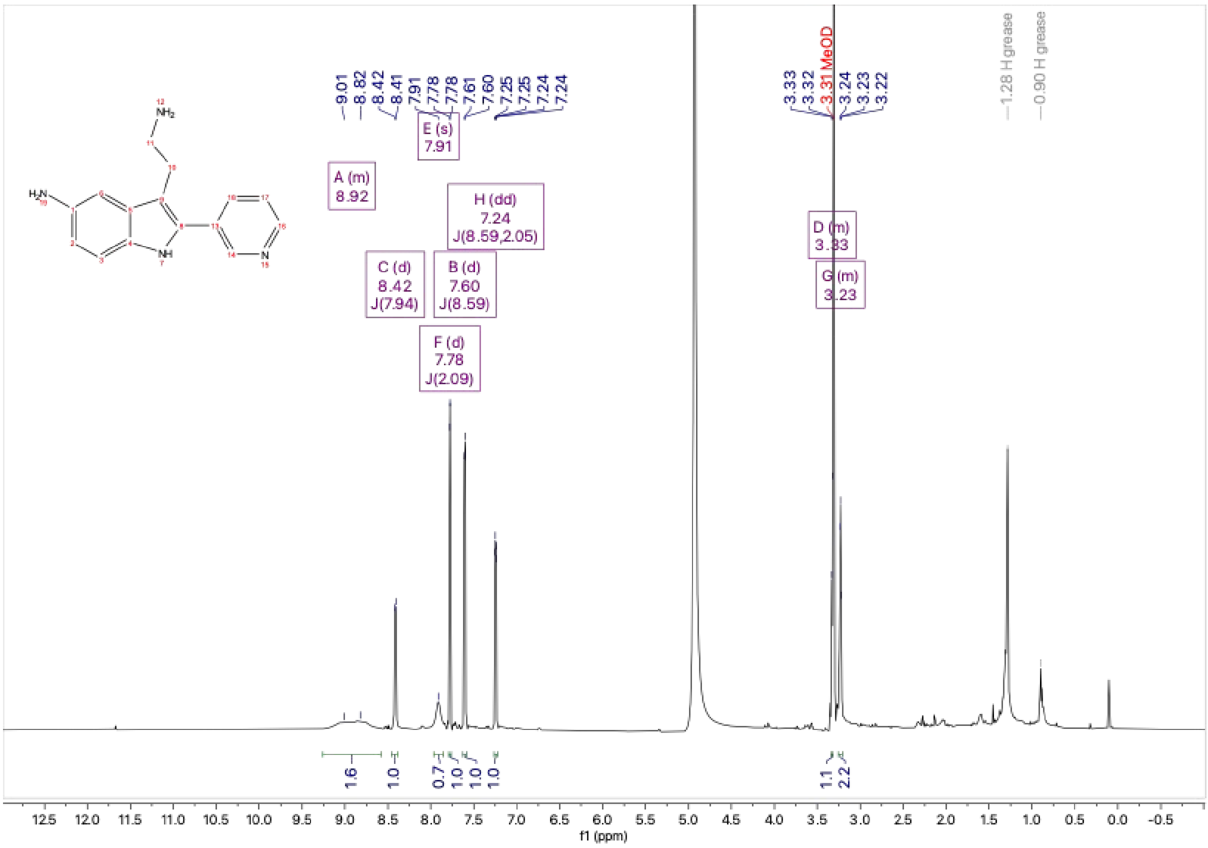

Compound 36 ^{13}C

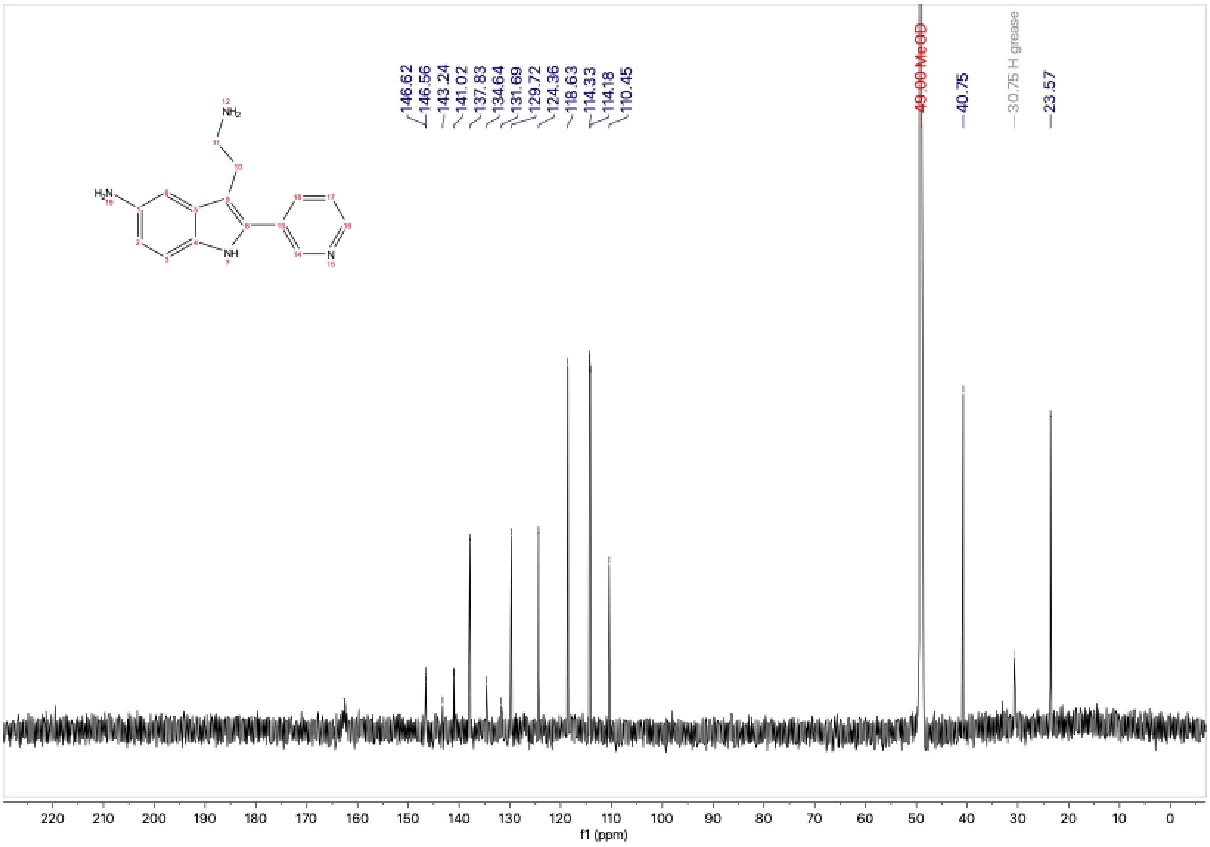

Compound 37 ^{1}H

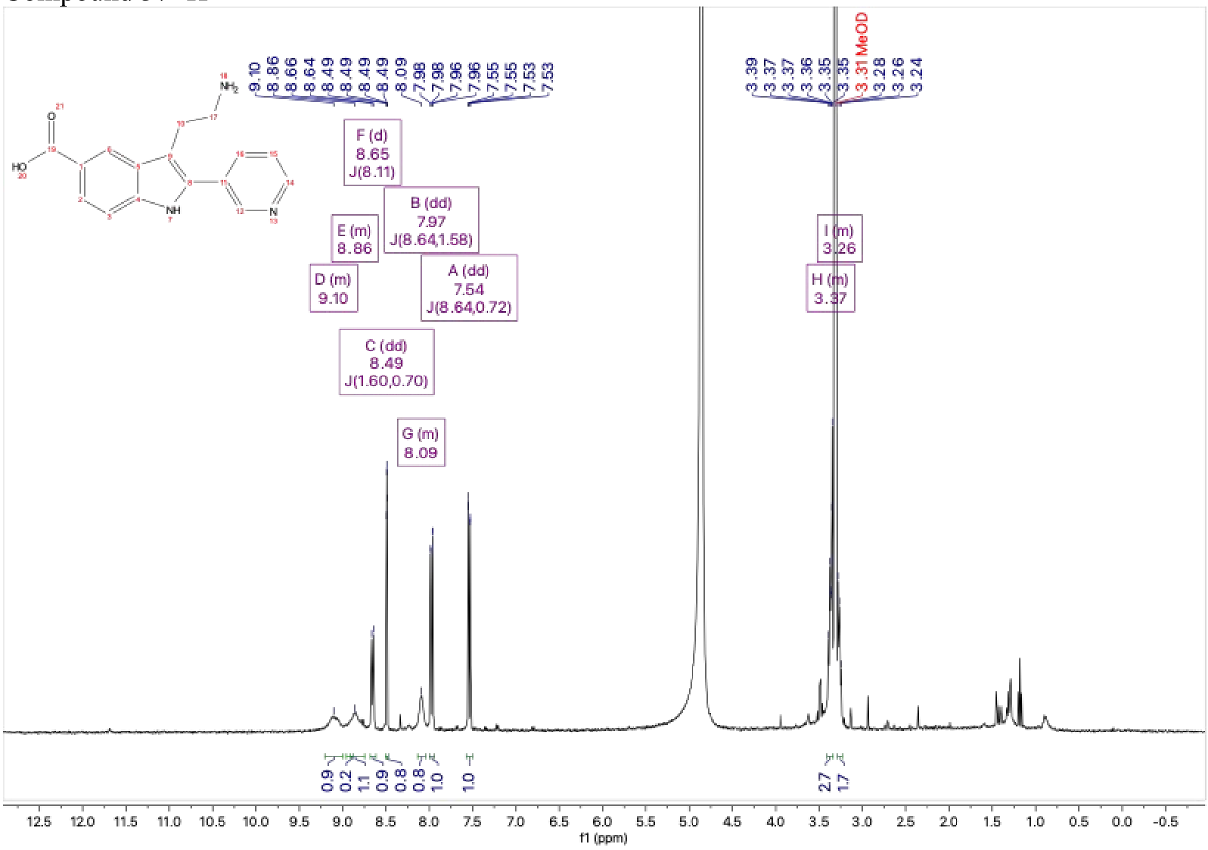

Compound 37 ^{13}C

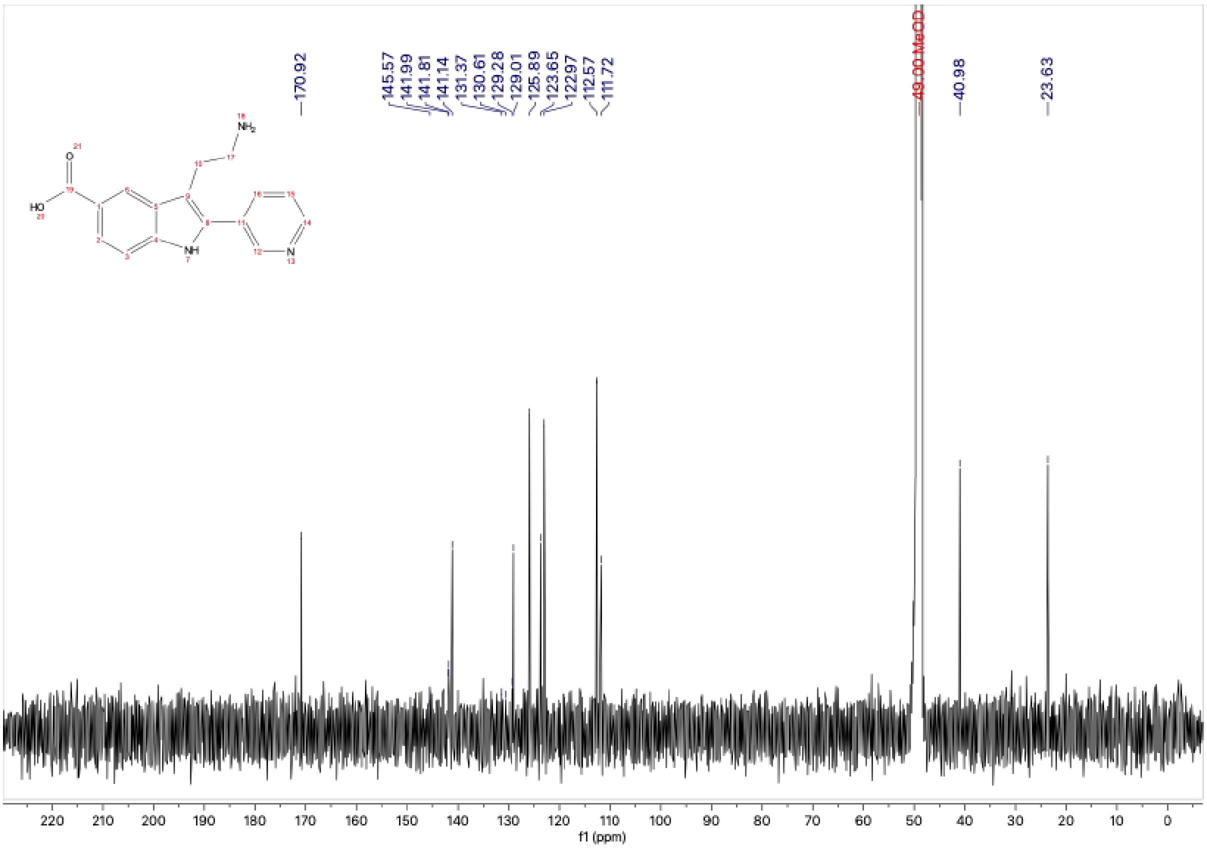

Compound 38 ^{1}H

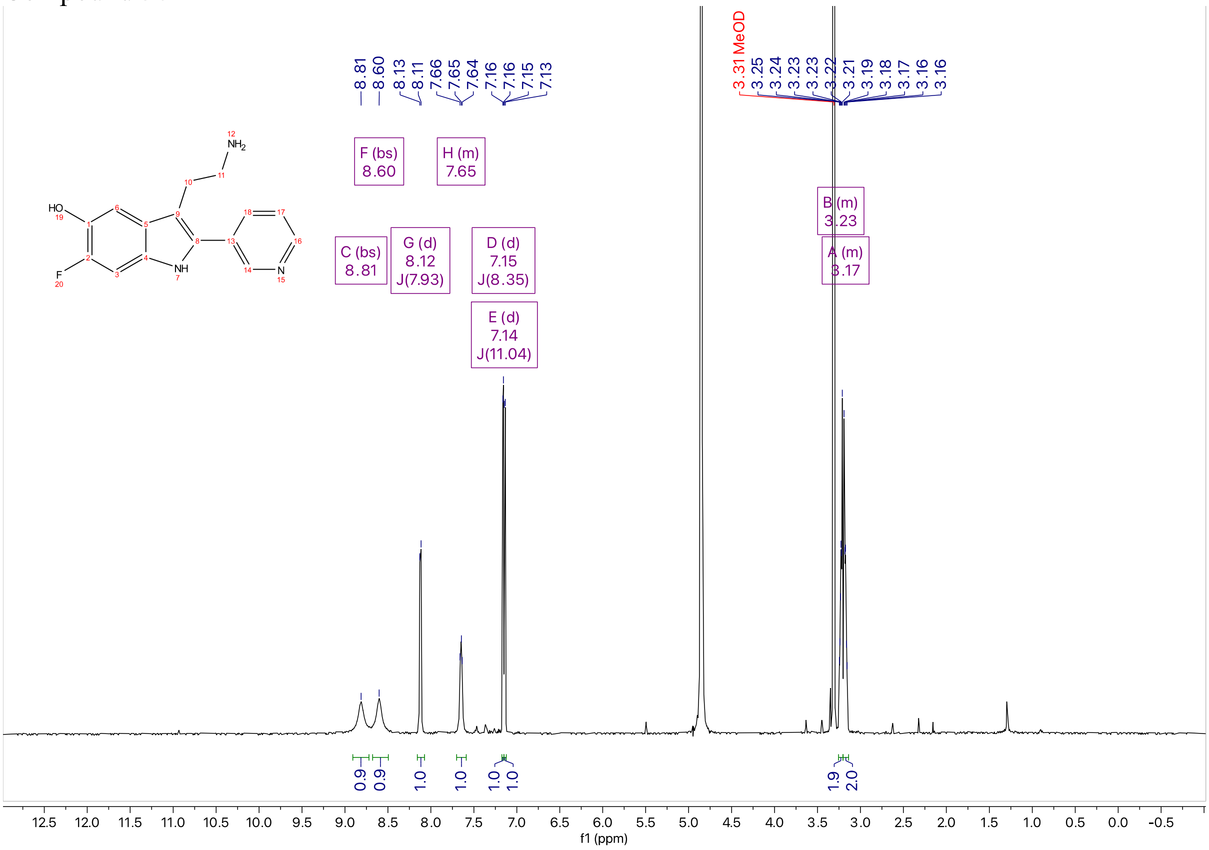

Compound 38 ^{13}C

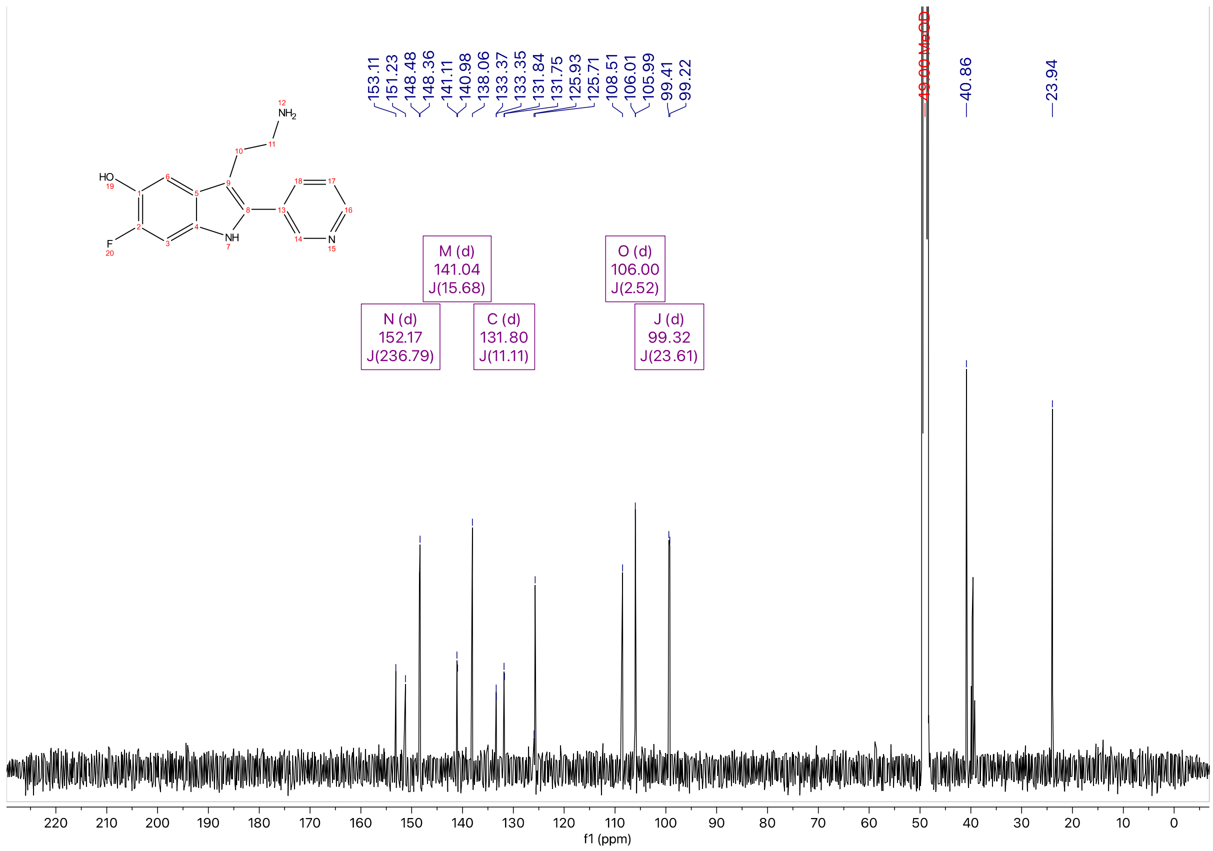

Compound 38 ^{19}F

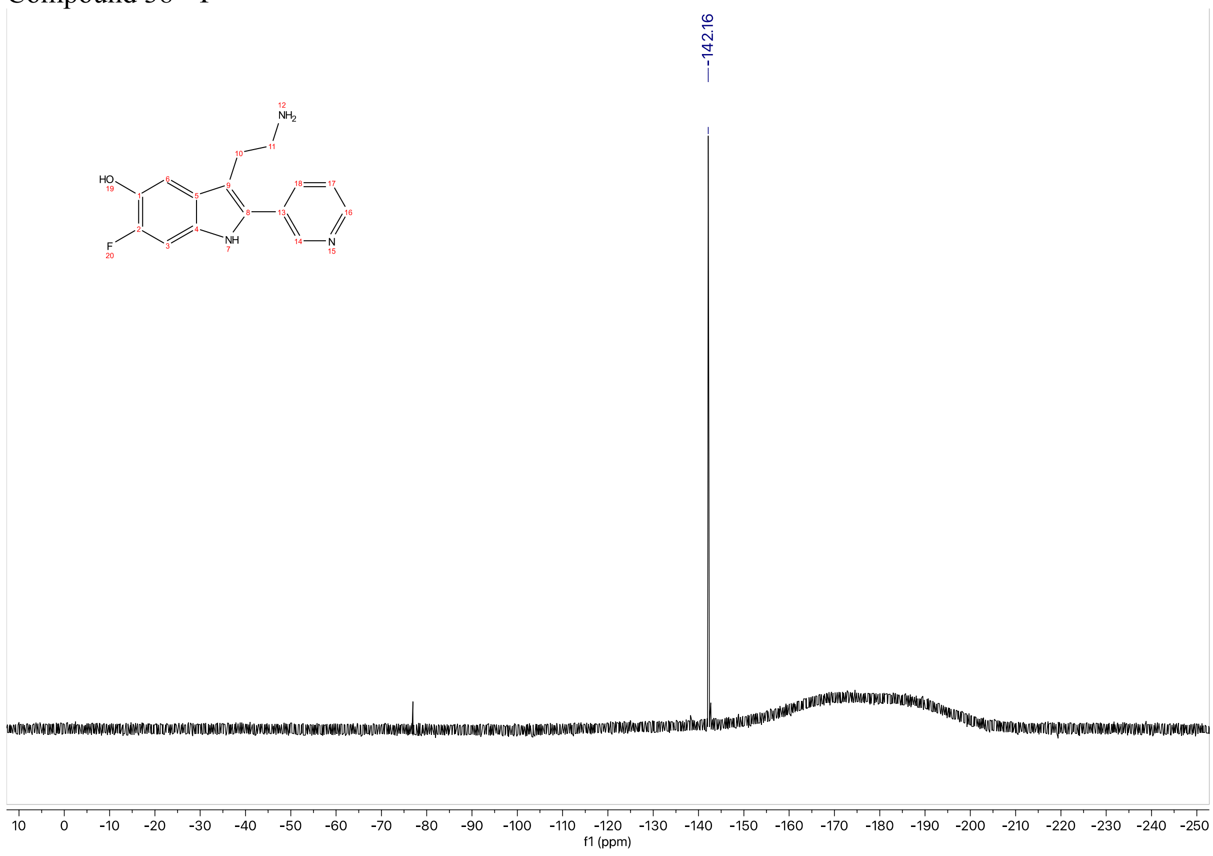

Compound 39 [1]H

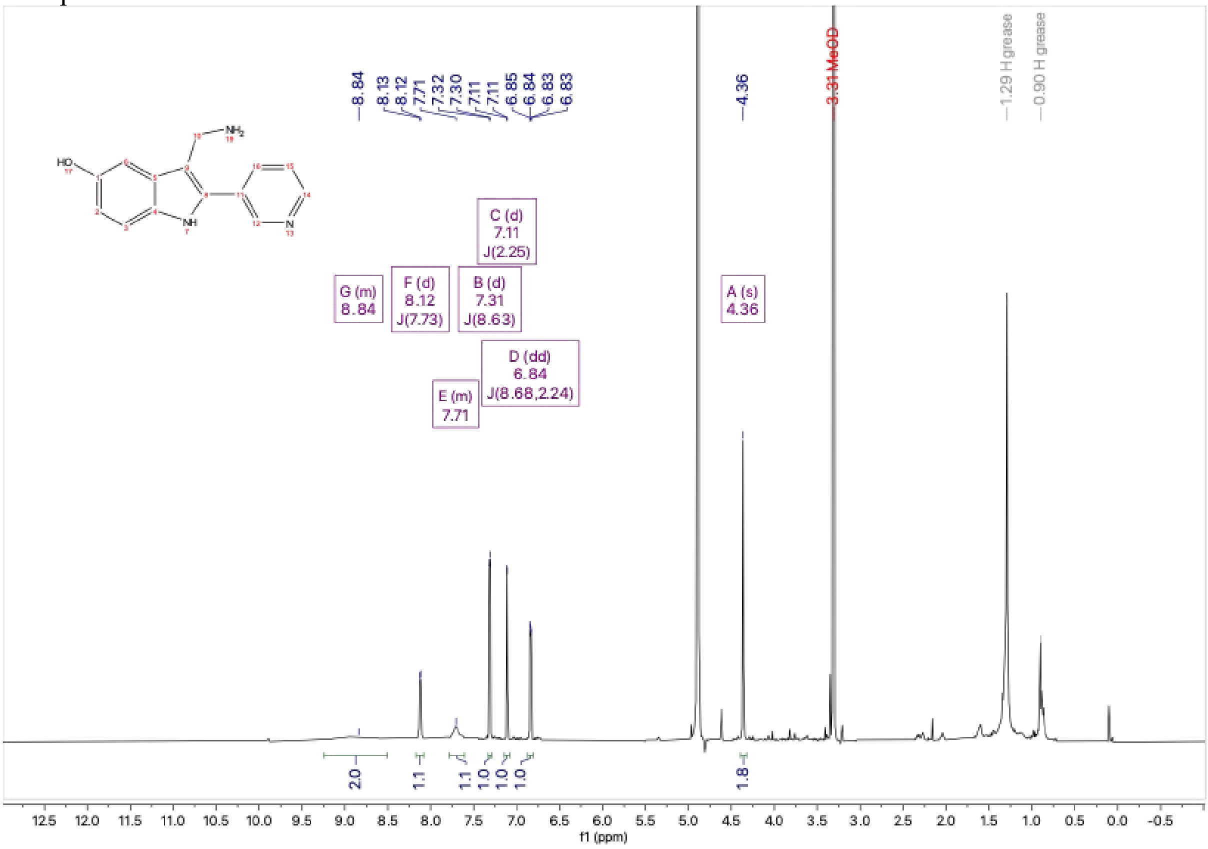

Compound 39 [13]C

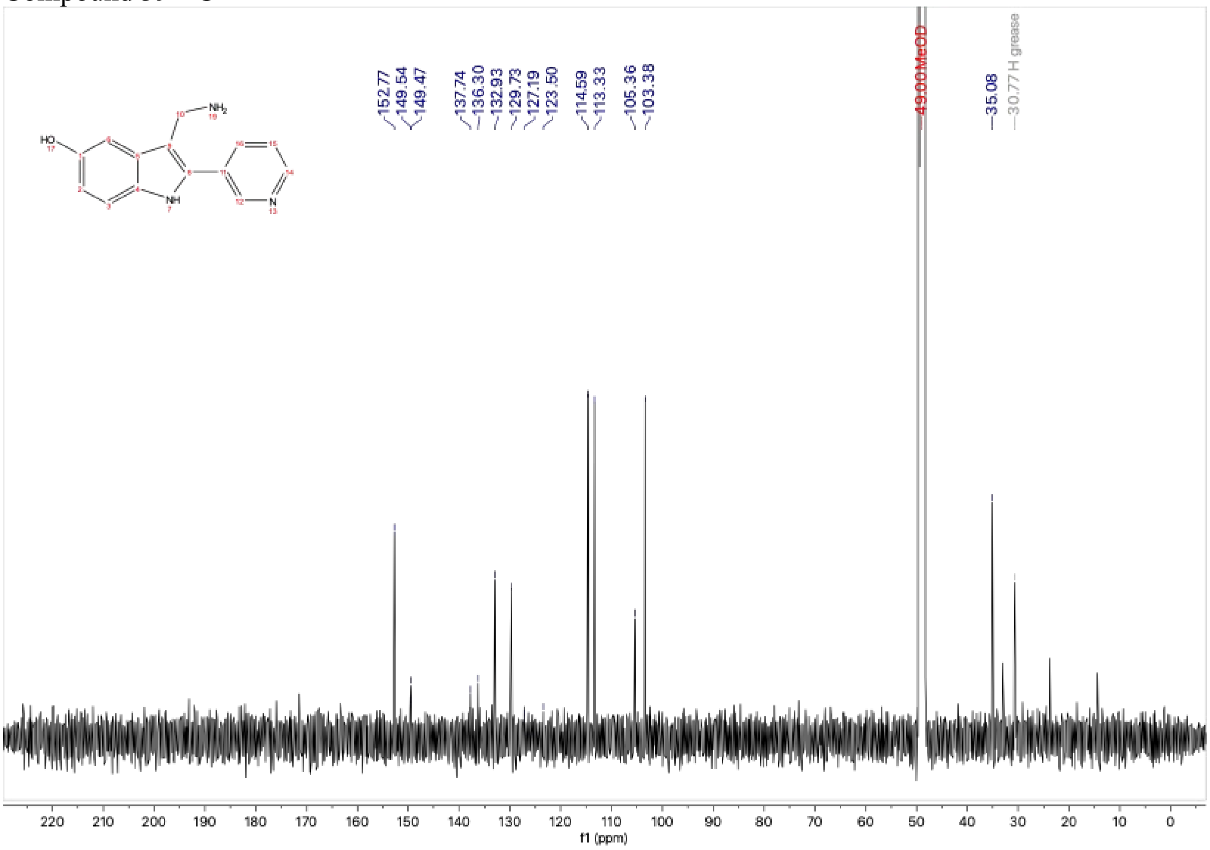

Compound 40 [1]H

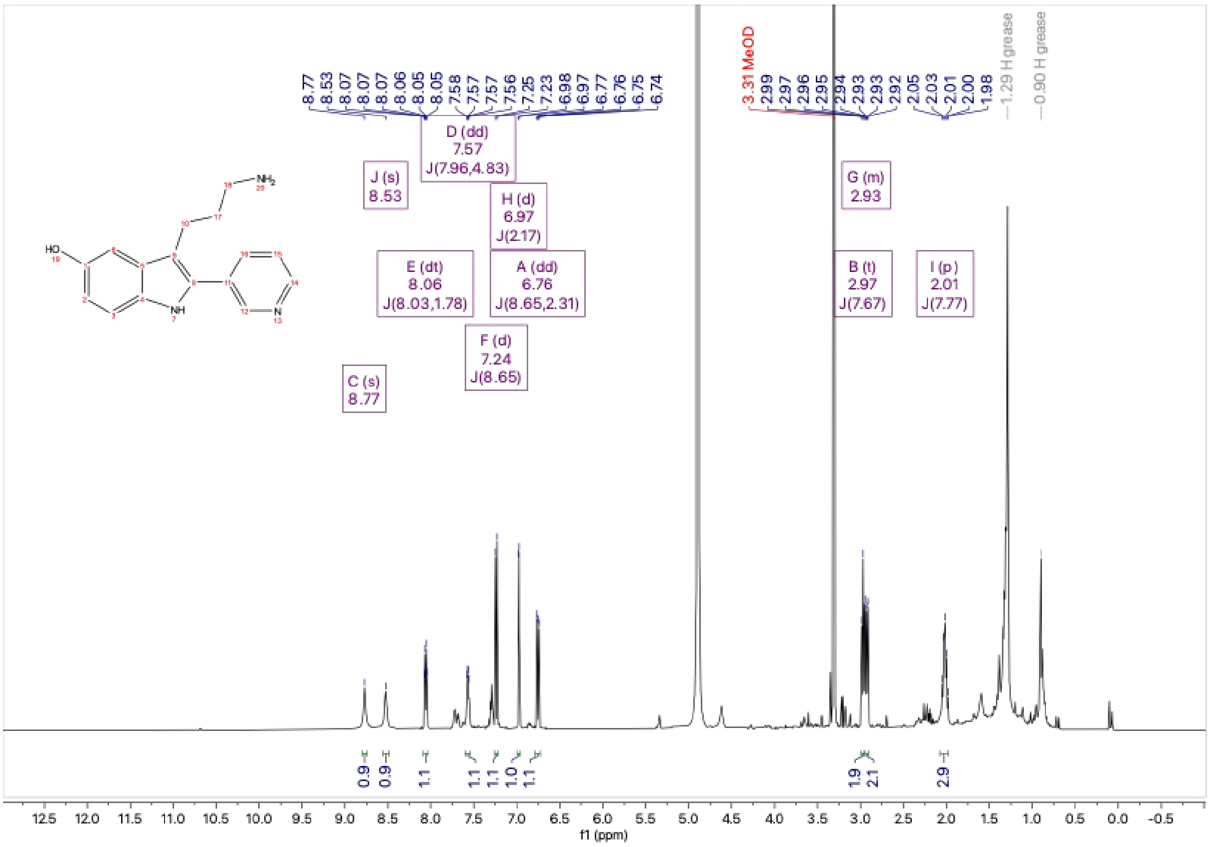

Compound 40 ^{13}C

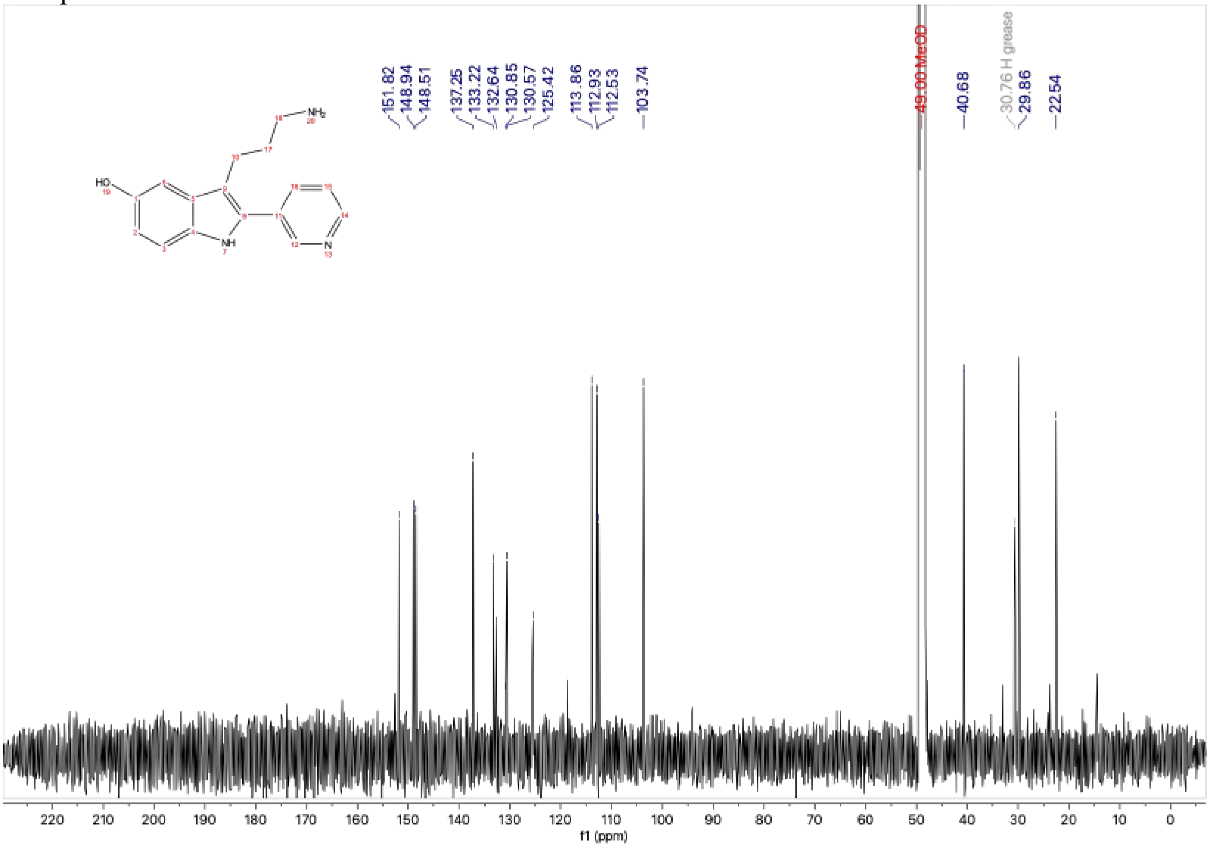

Compound 41 ^{1}H

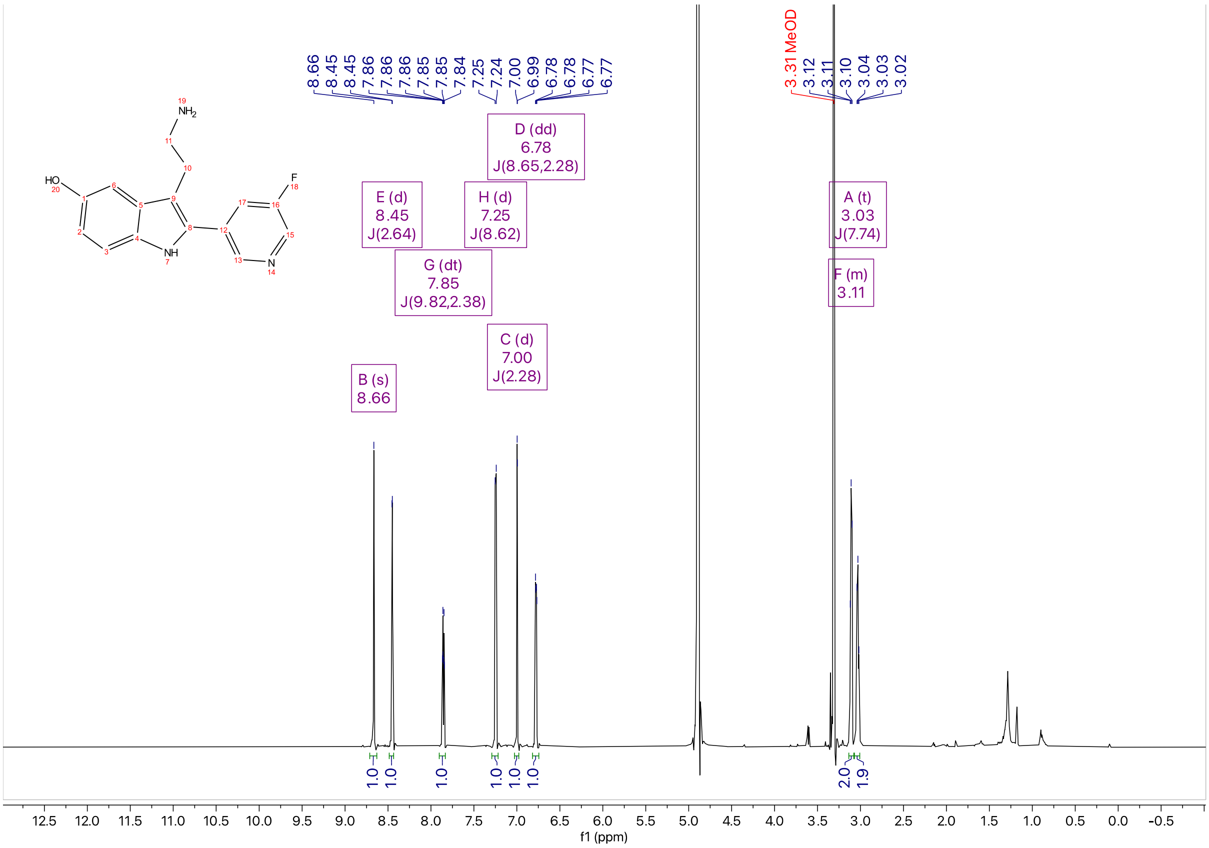

Compound 41 ^{13}C

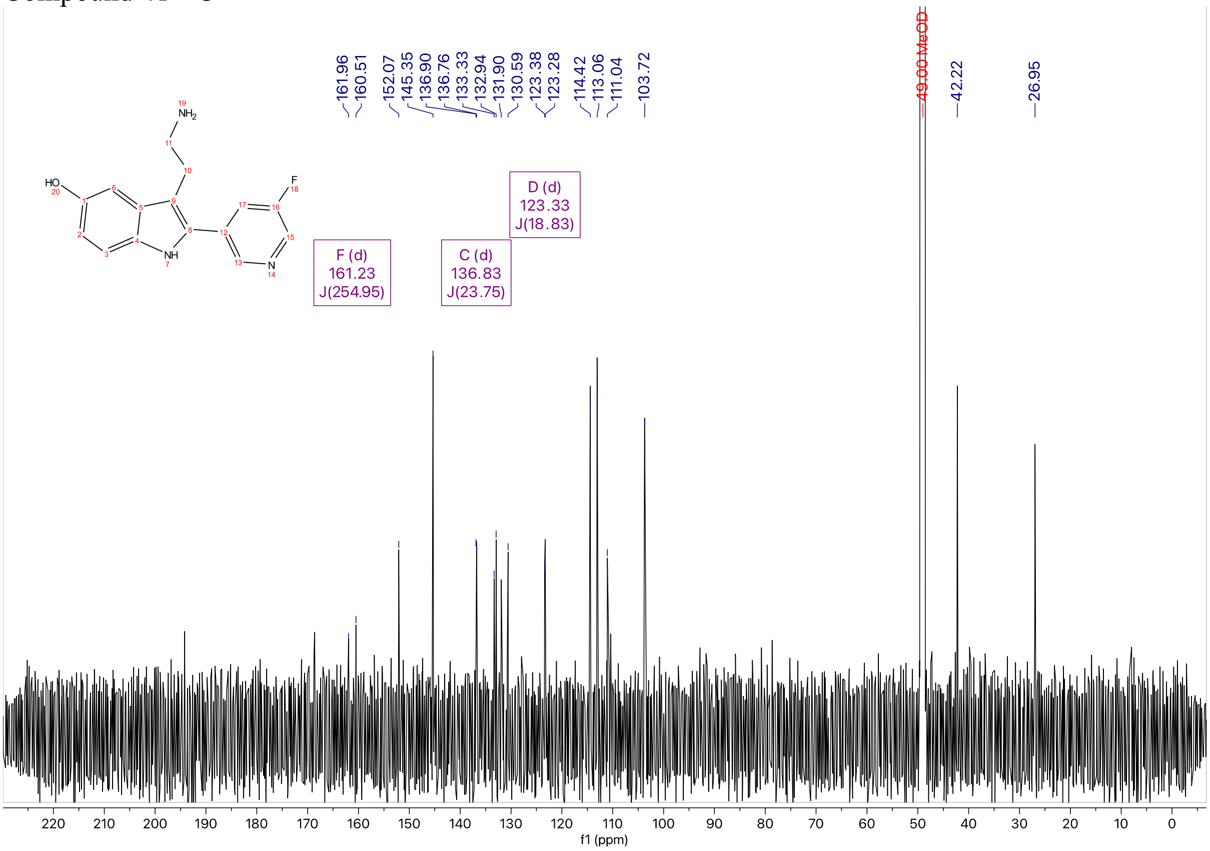

Compound 41 ^{19}F

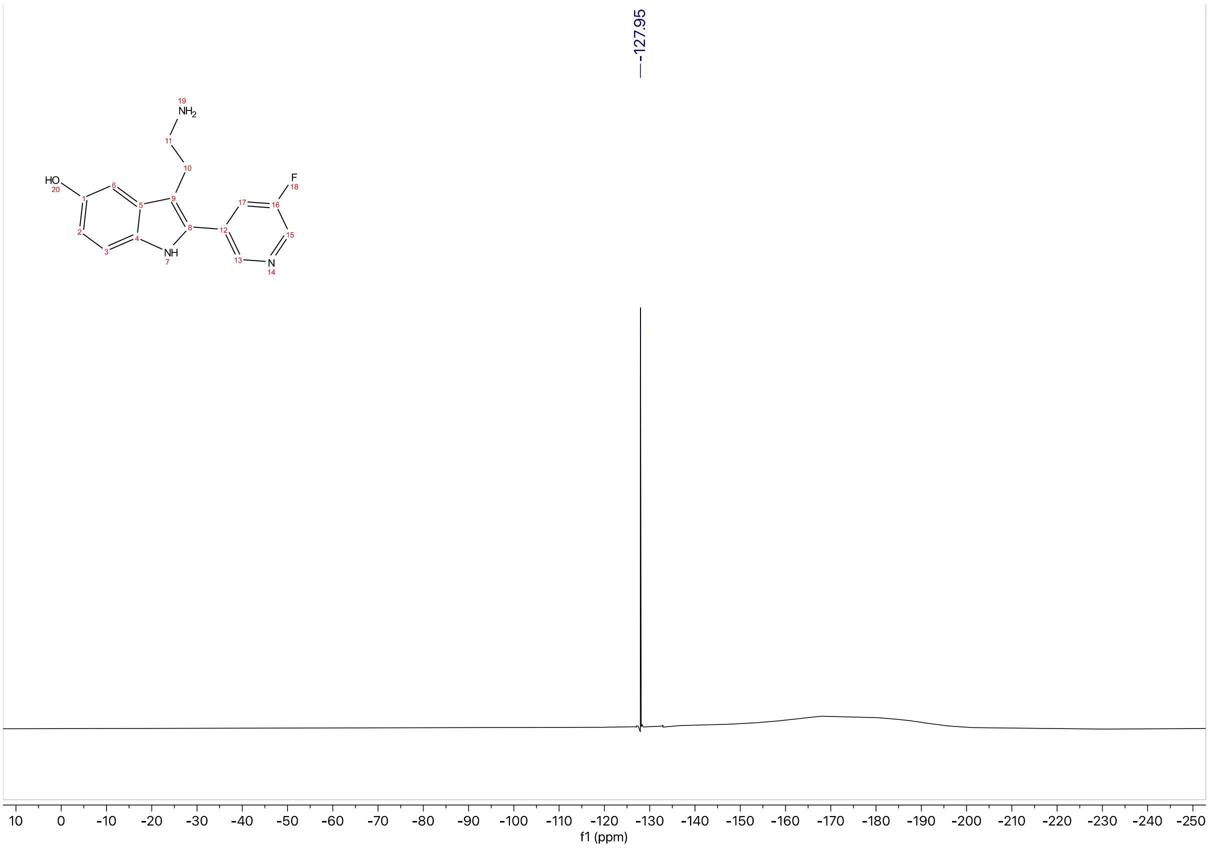

Compound 42 ^{1}H

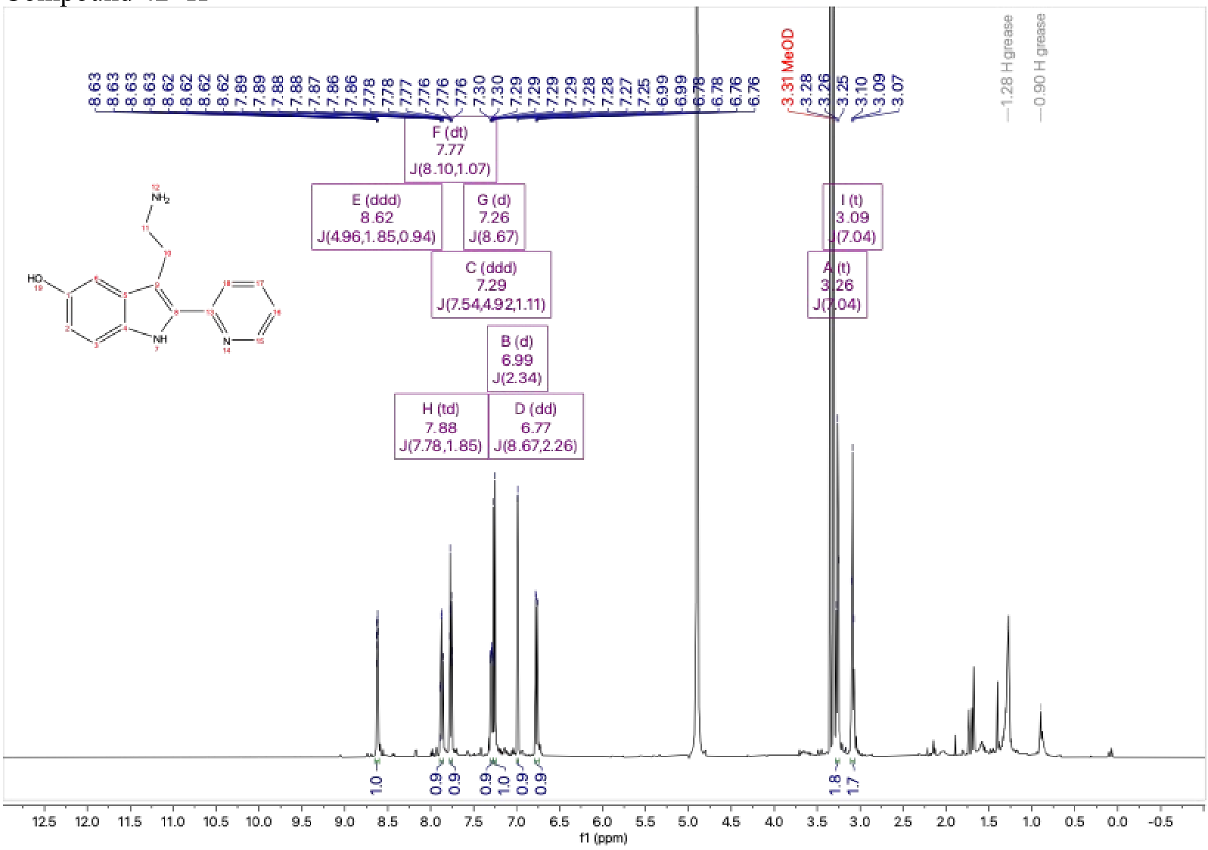

Compound 42 ^{13}C

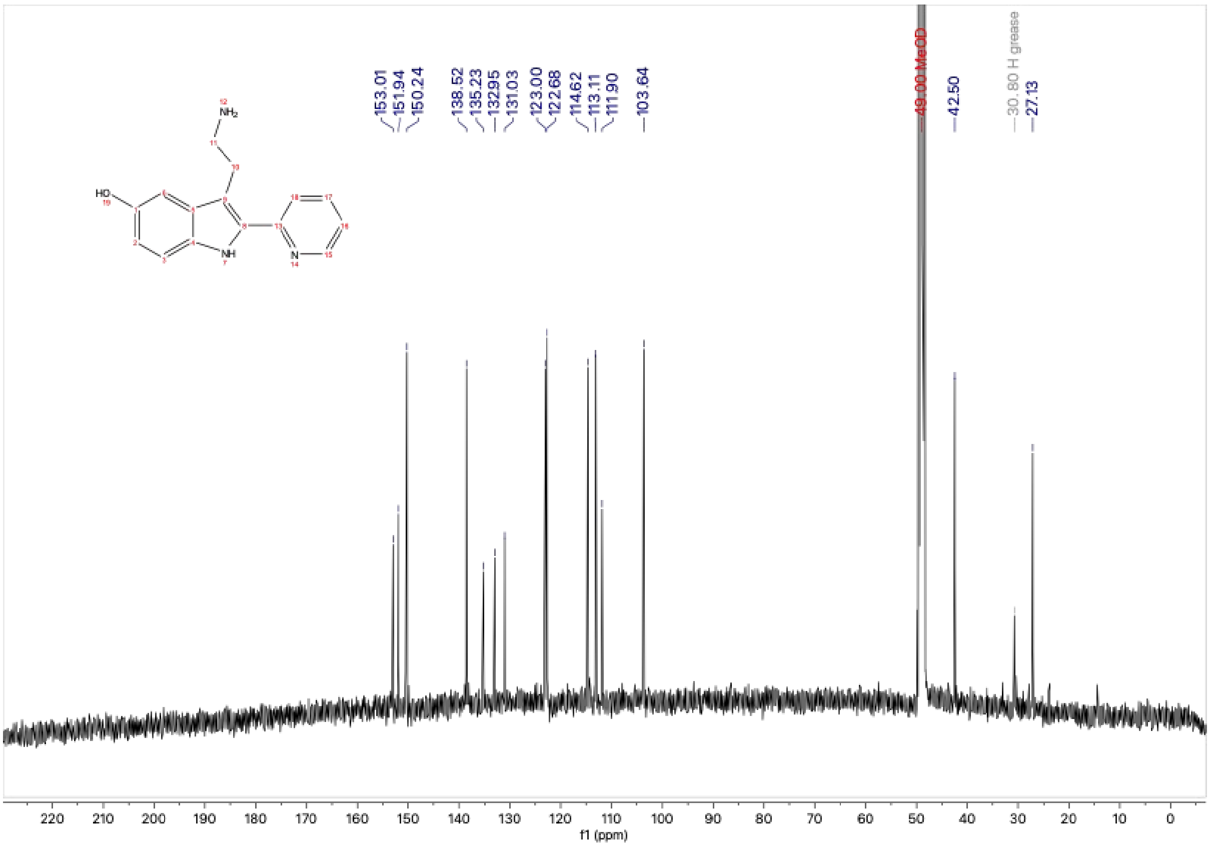

Compound 43 ^{1}H

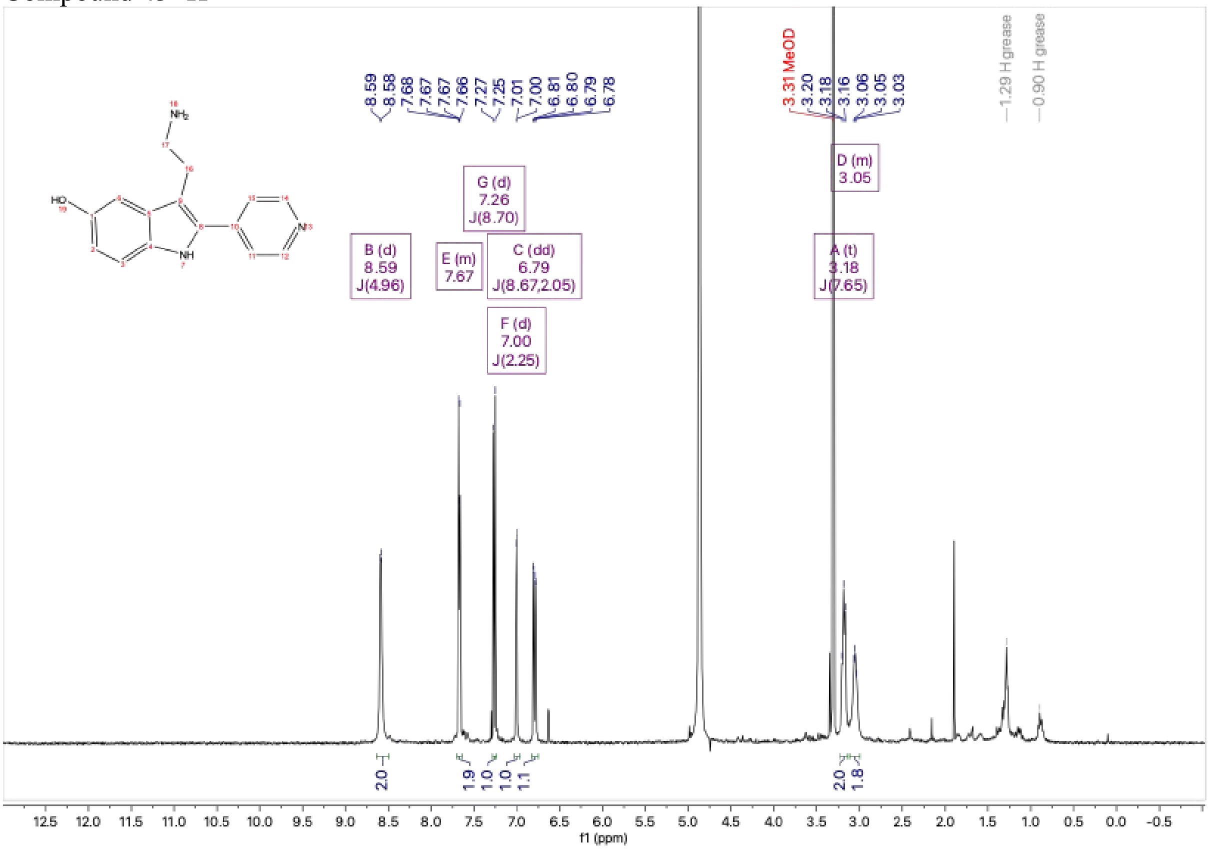

Compound 43 ^{13}C

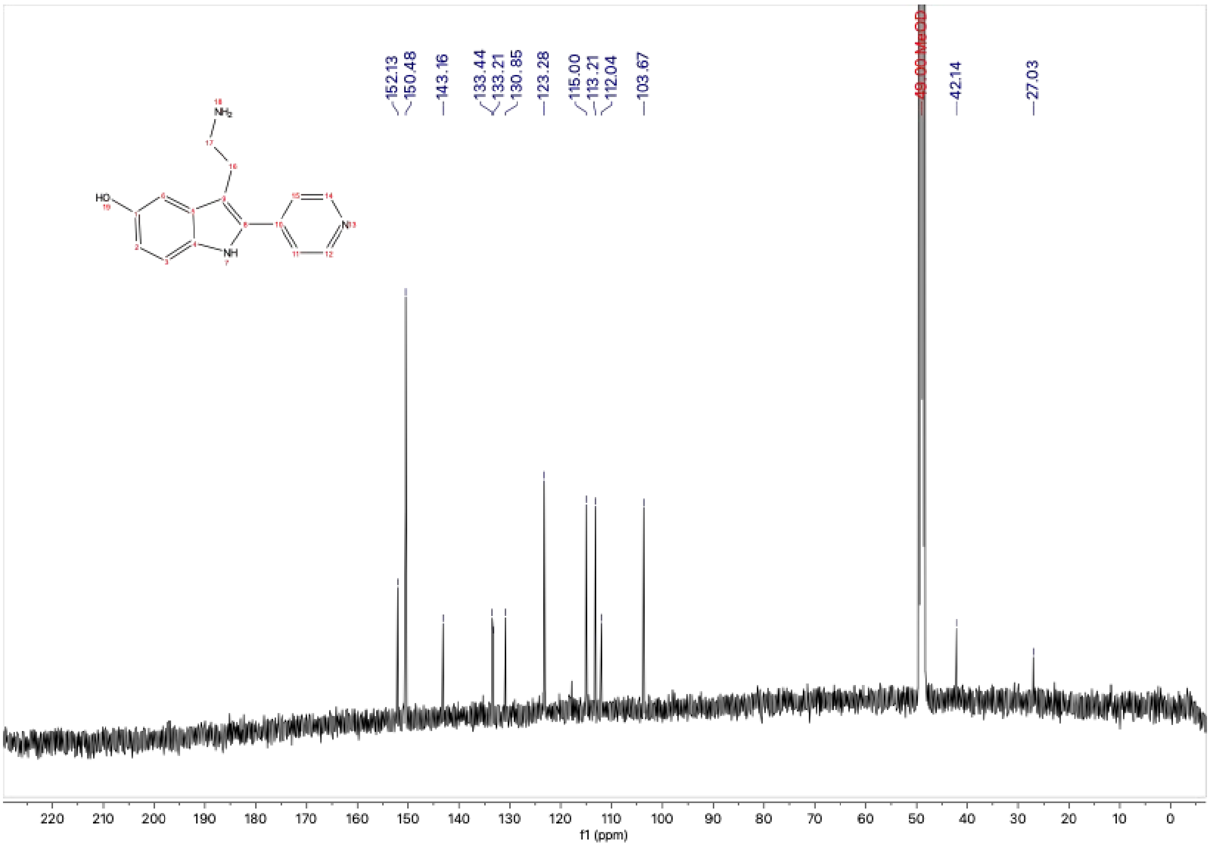

Compound 44 ^{1}H

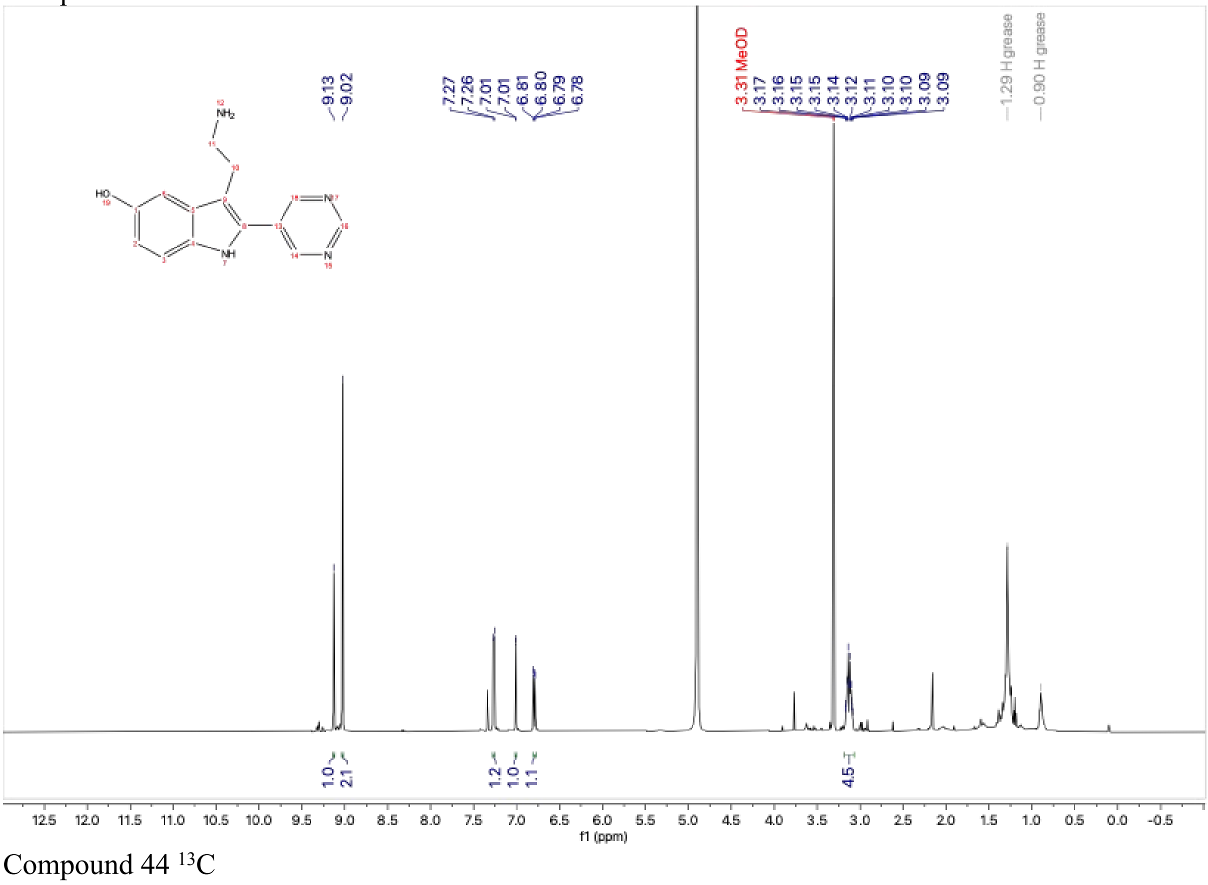

Compound 44 ^{13}C

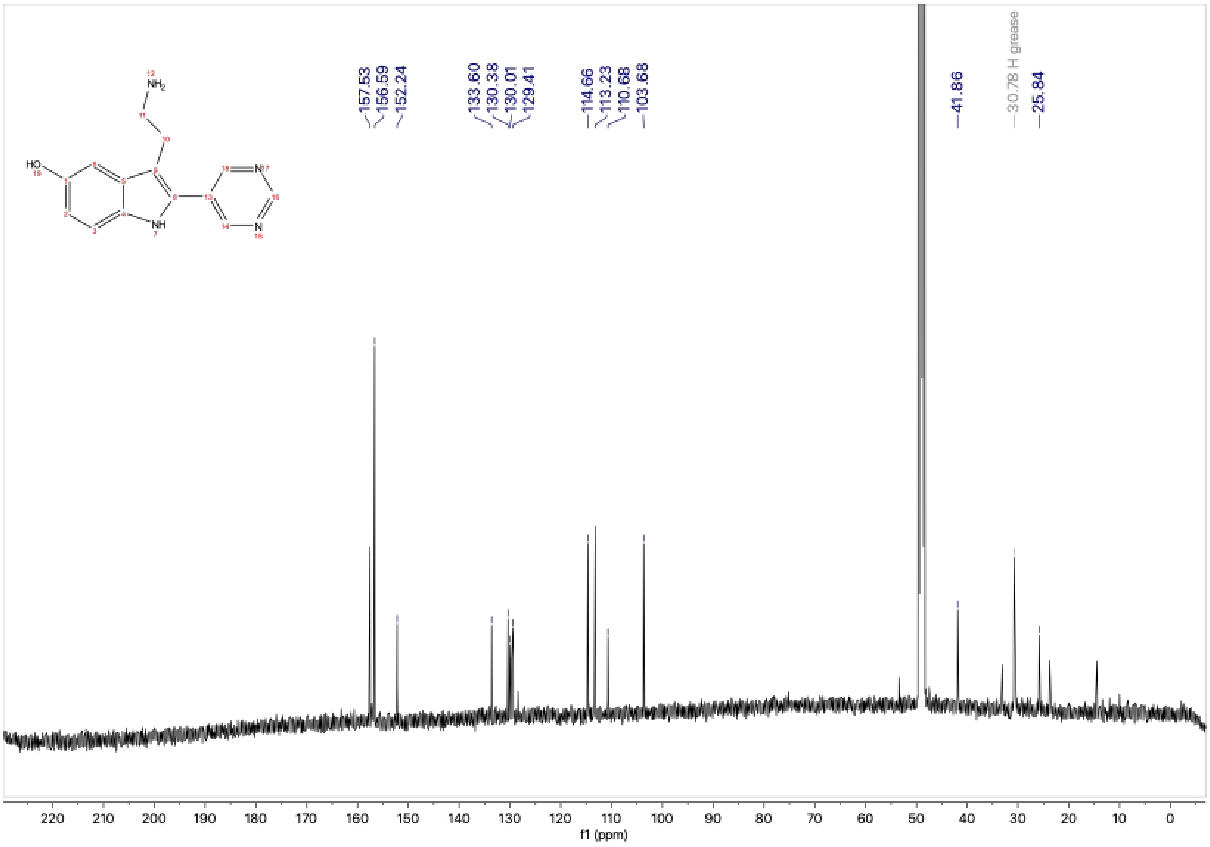

Compound 45 ^{1}H

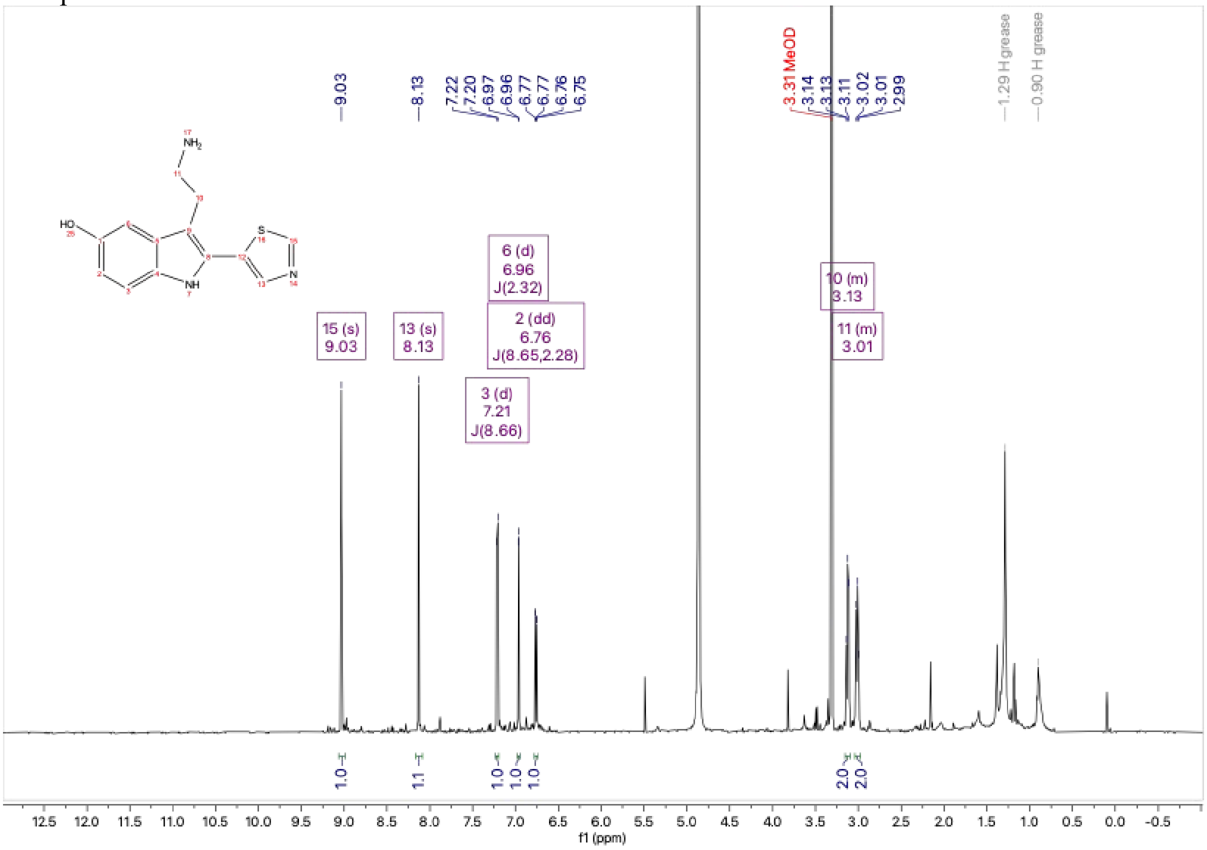

Compound 45 ^{13}C

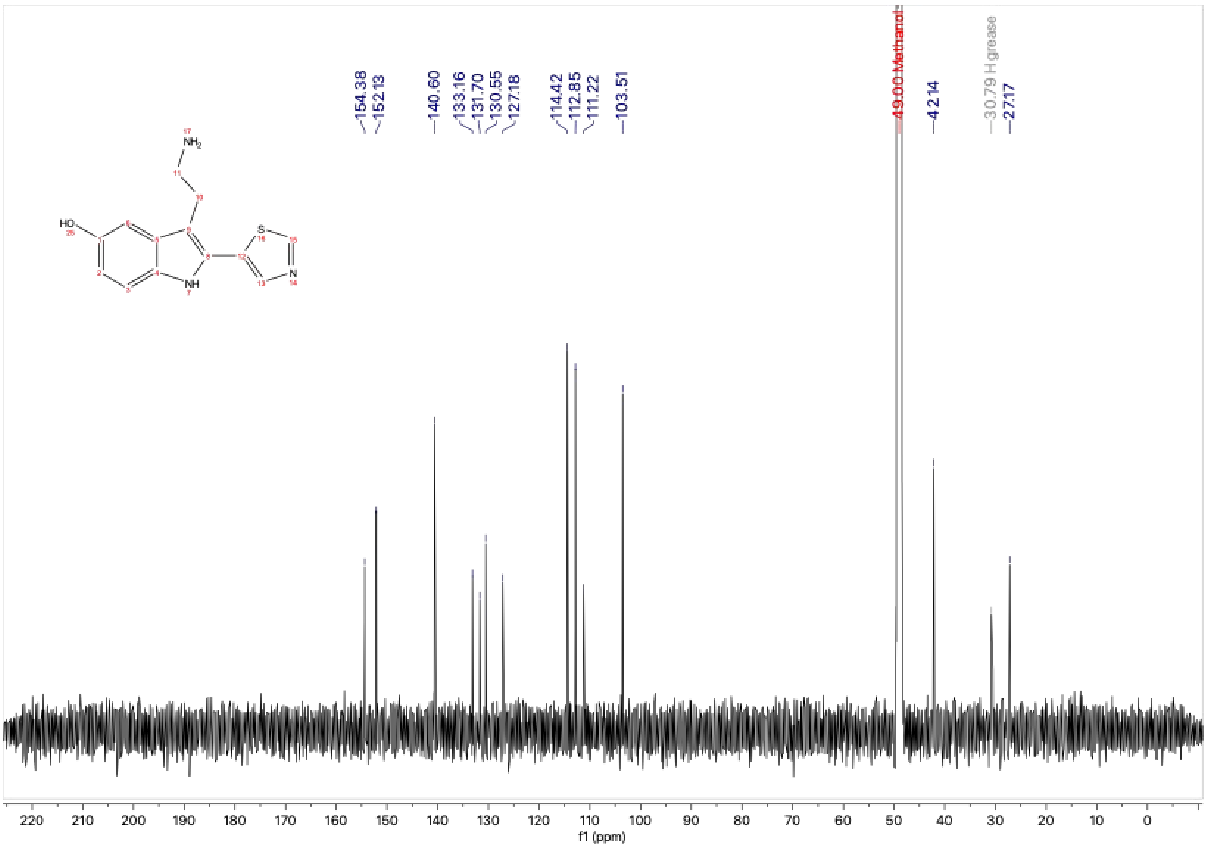

Compound 46 ^{1}H

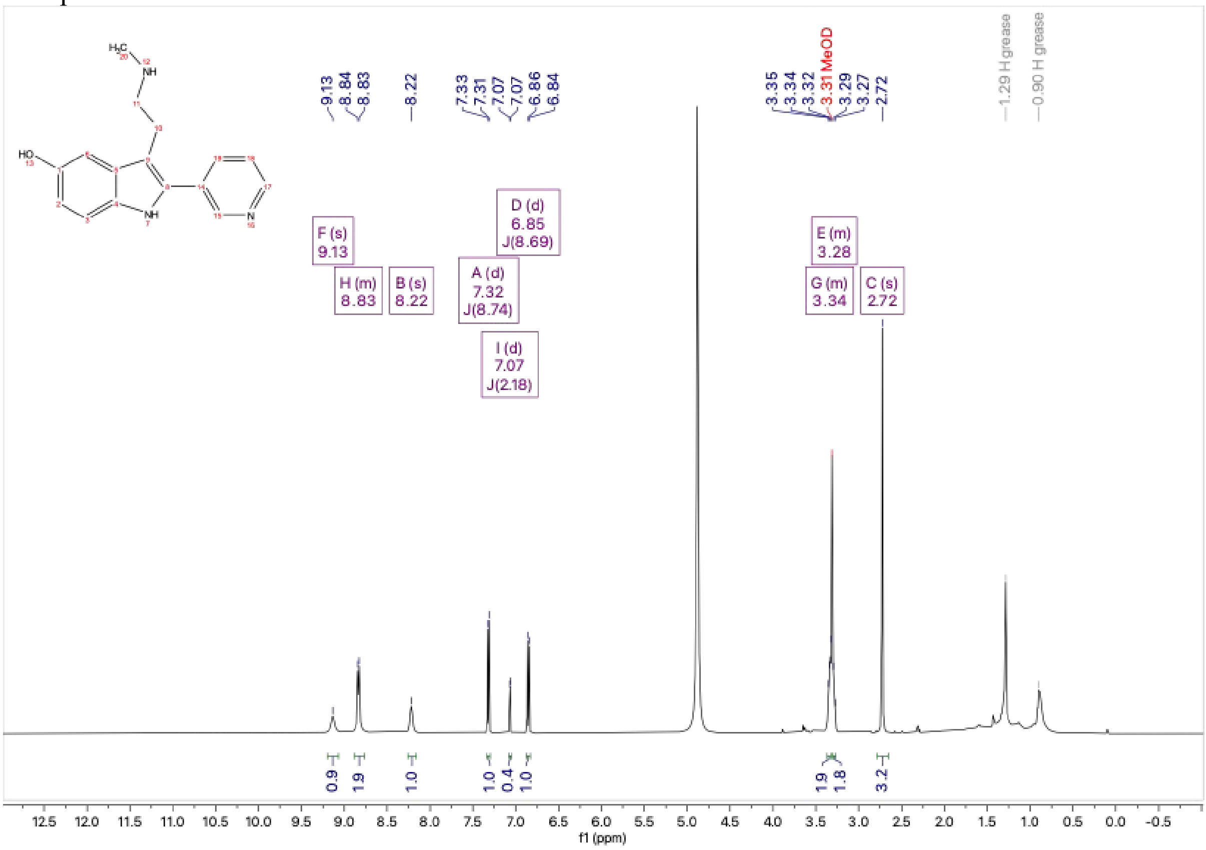

Compound 46 ^{13}C

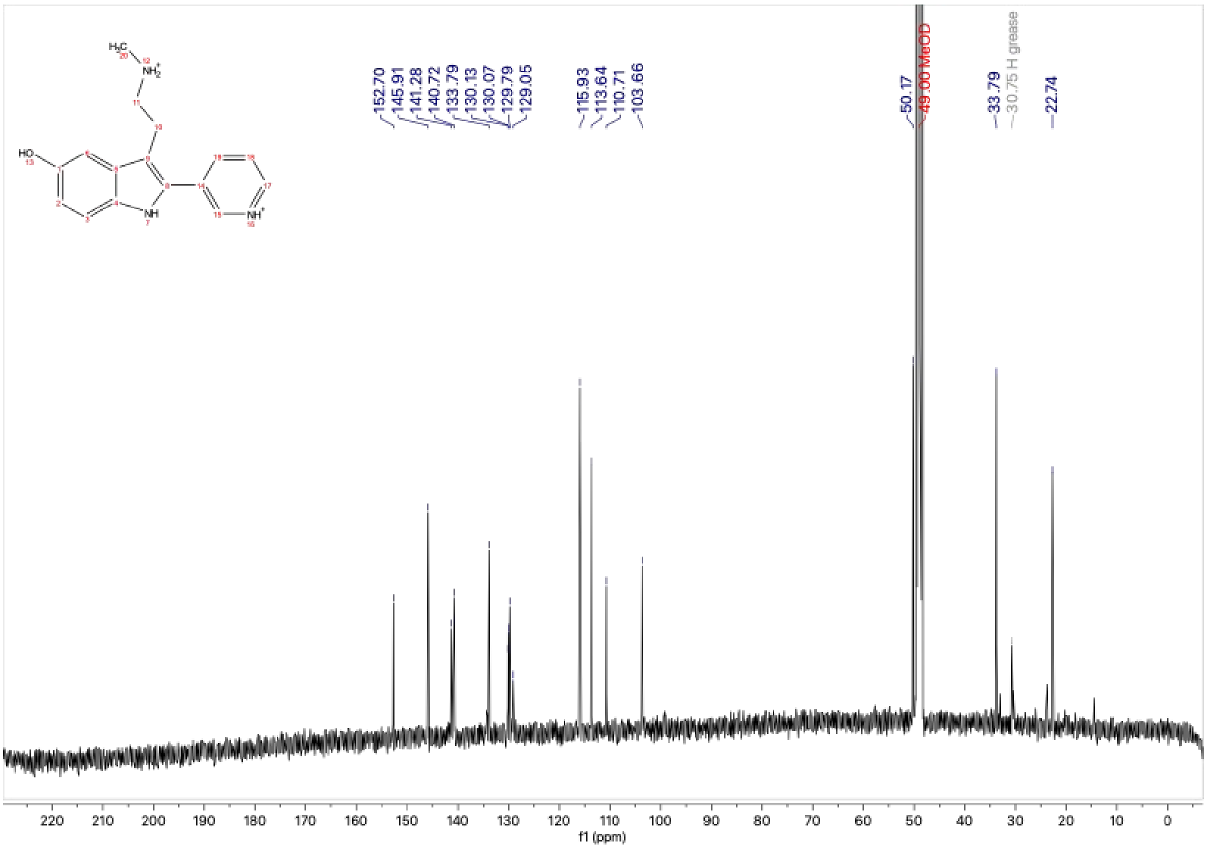

Compound 47 ^{1}H

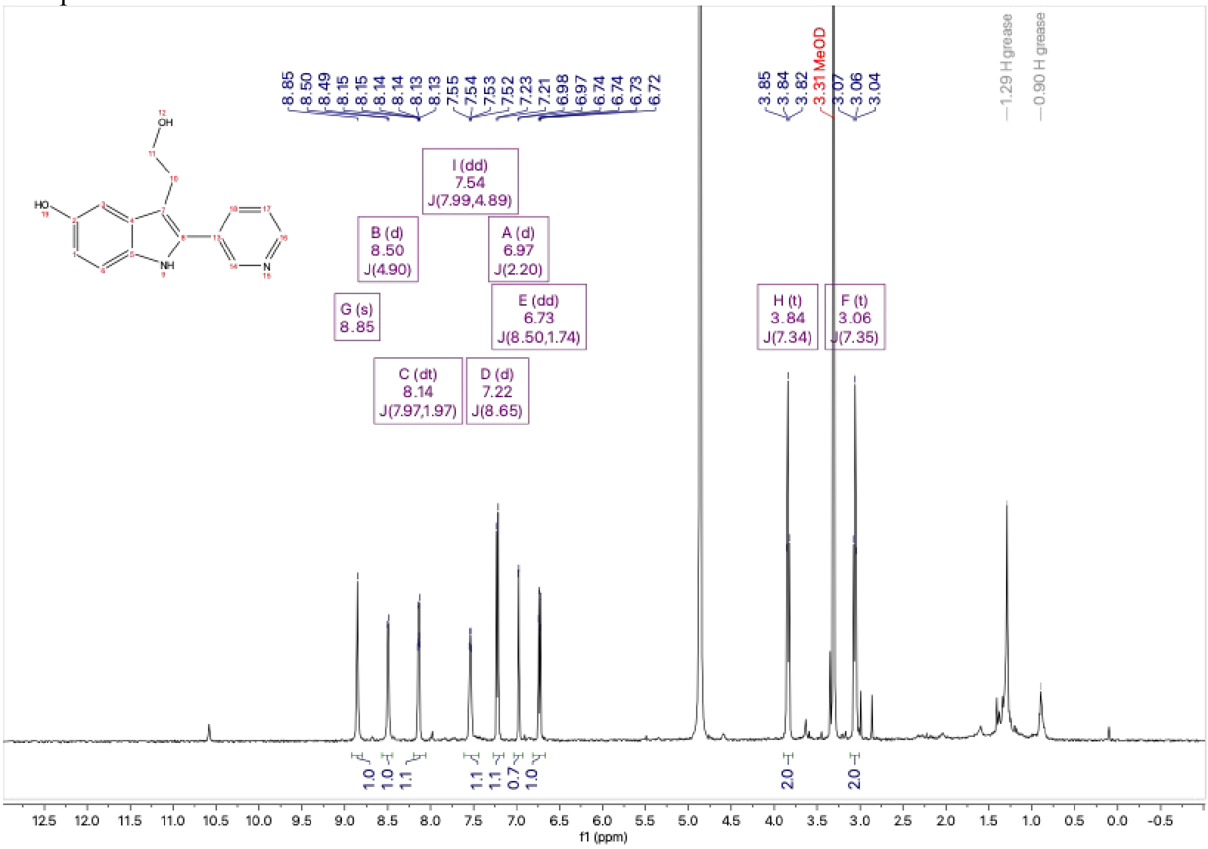

Compound 47 ^{13}C

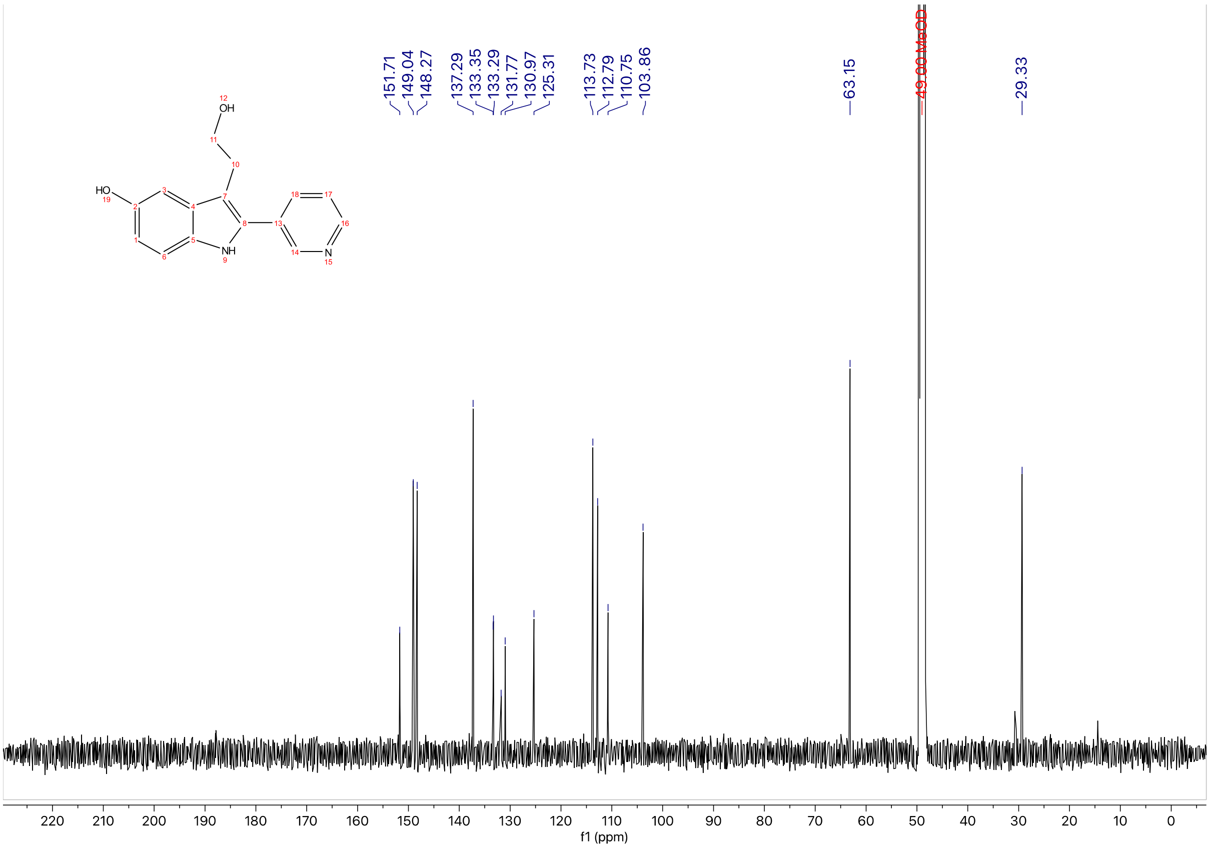

Compound 48 ^{1}H

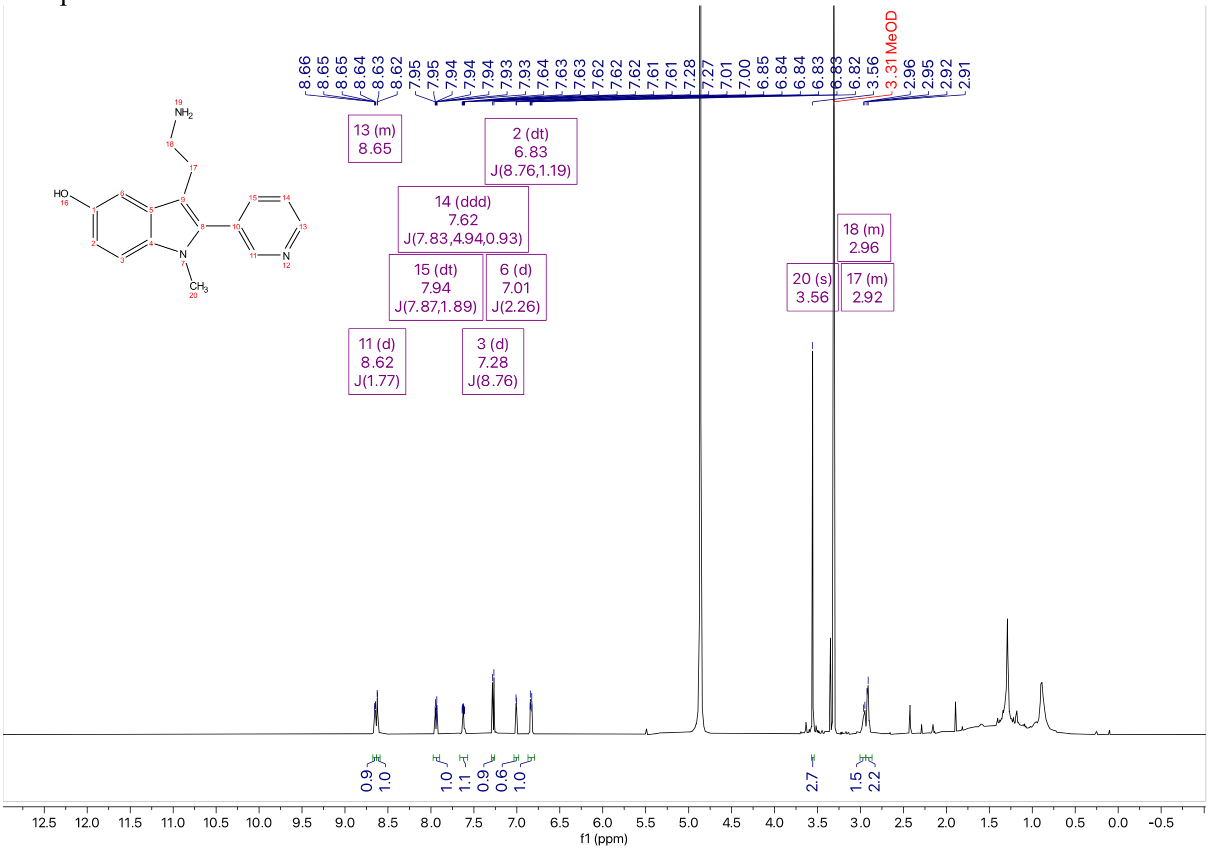

Compound 48 ^{13}C

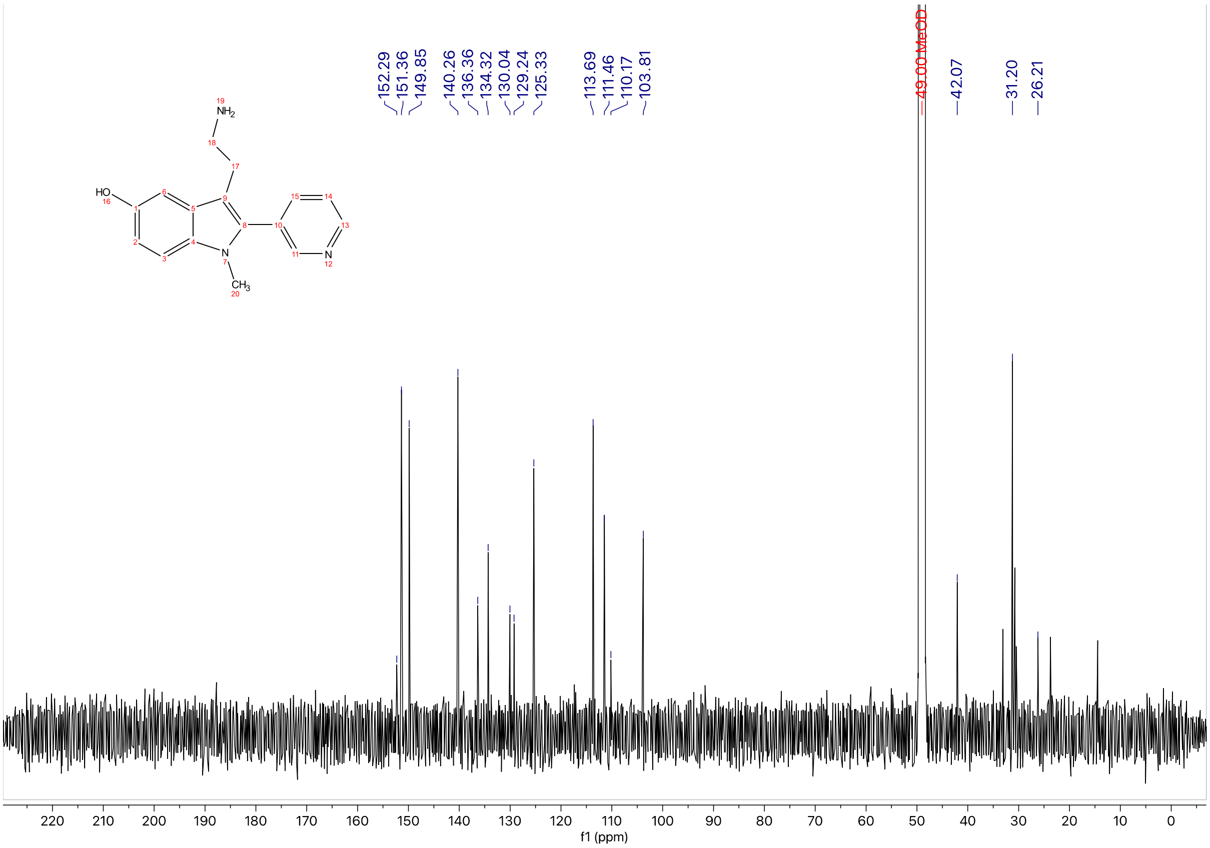

Compound 49 ^{1}H

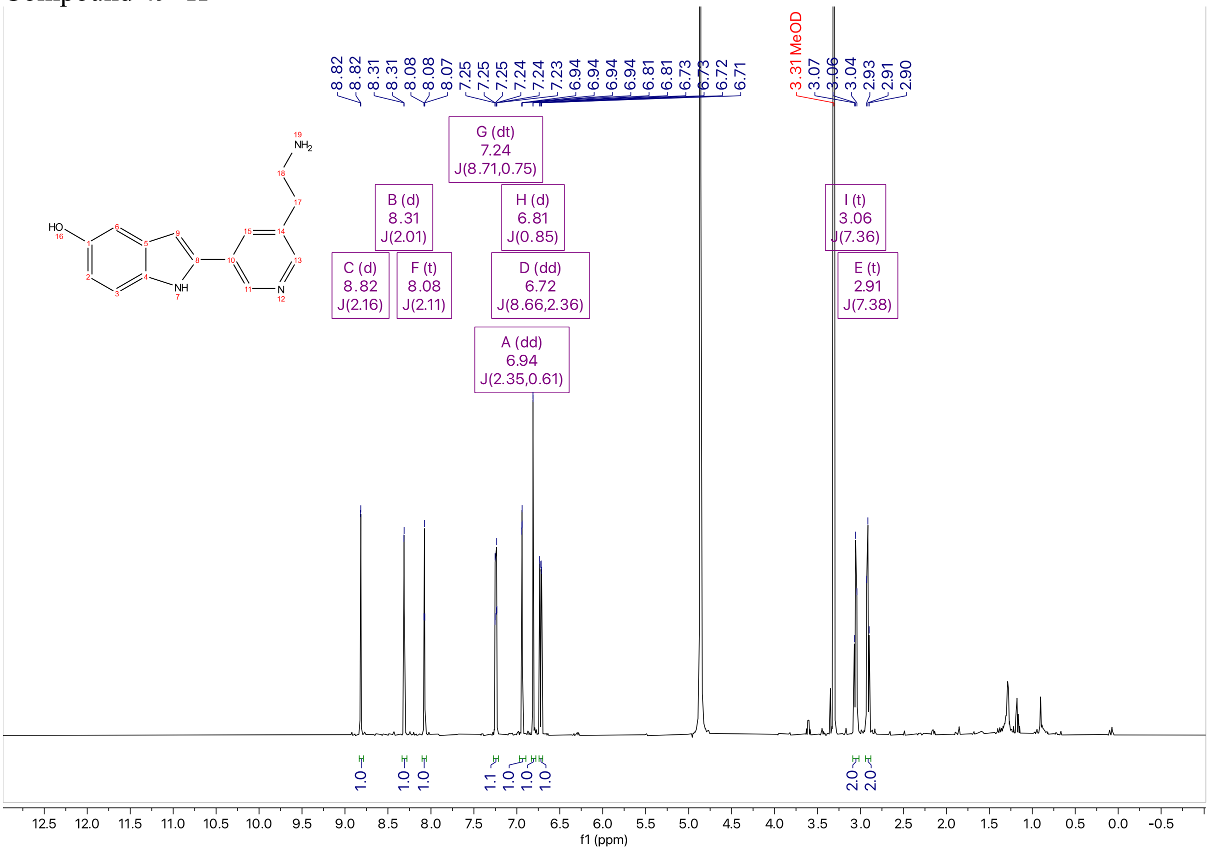

Compound 49 ^{13}C

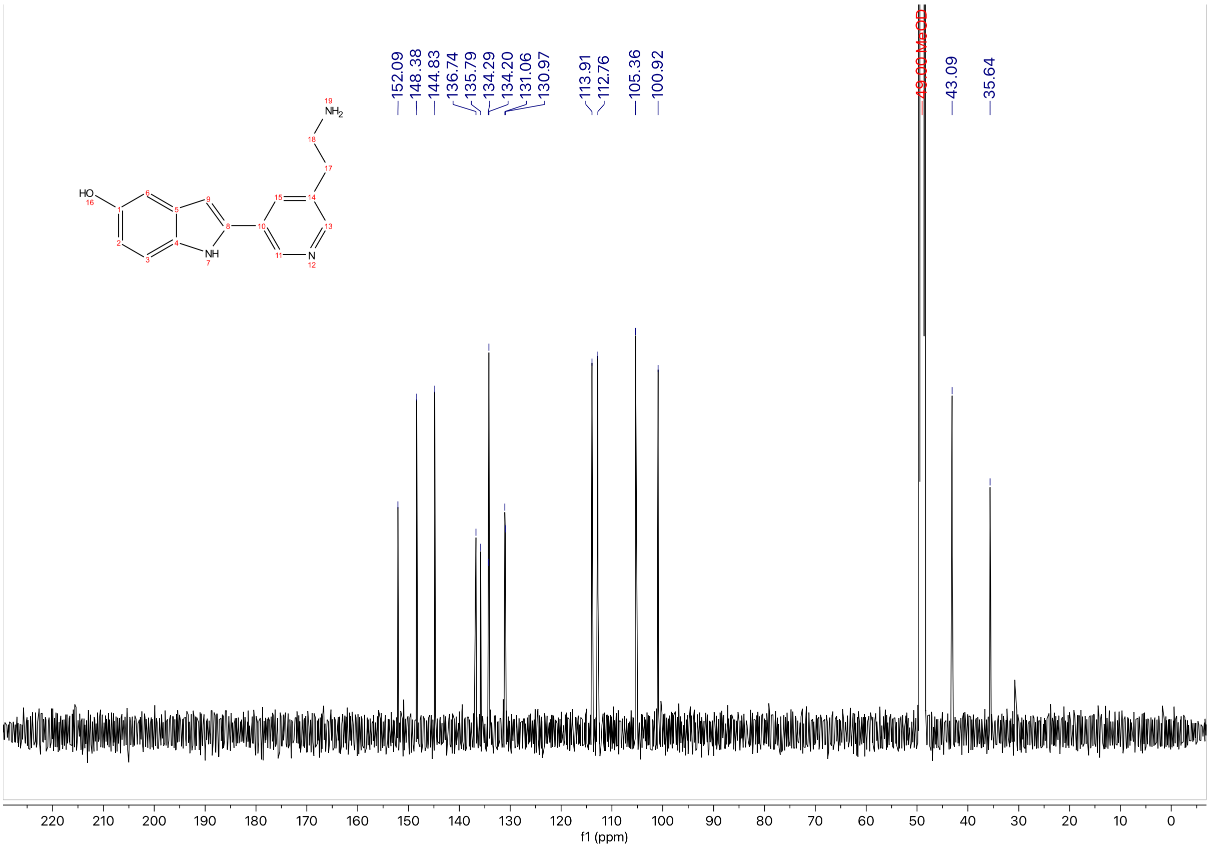

Compound 50 ^{1}H

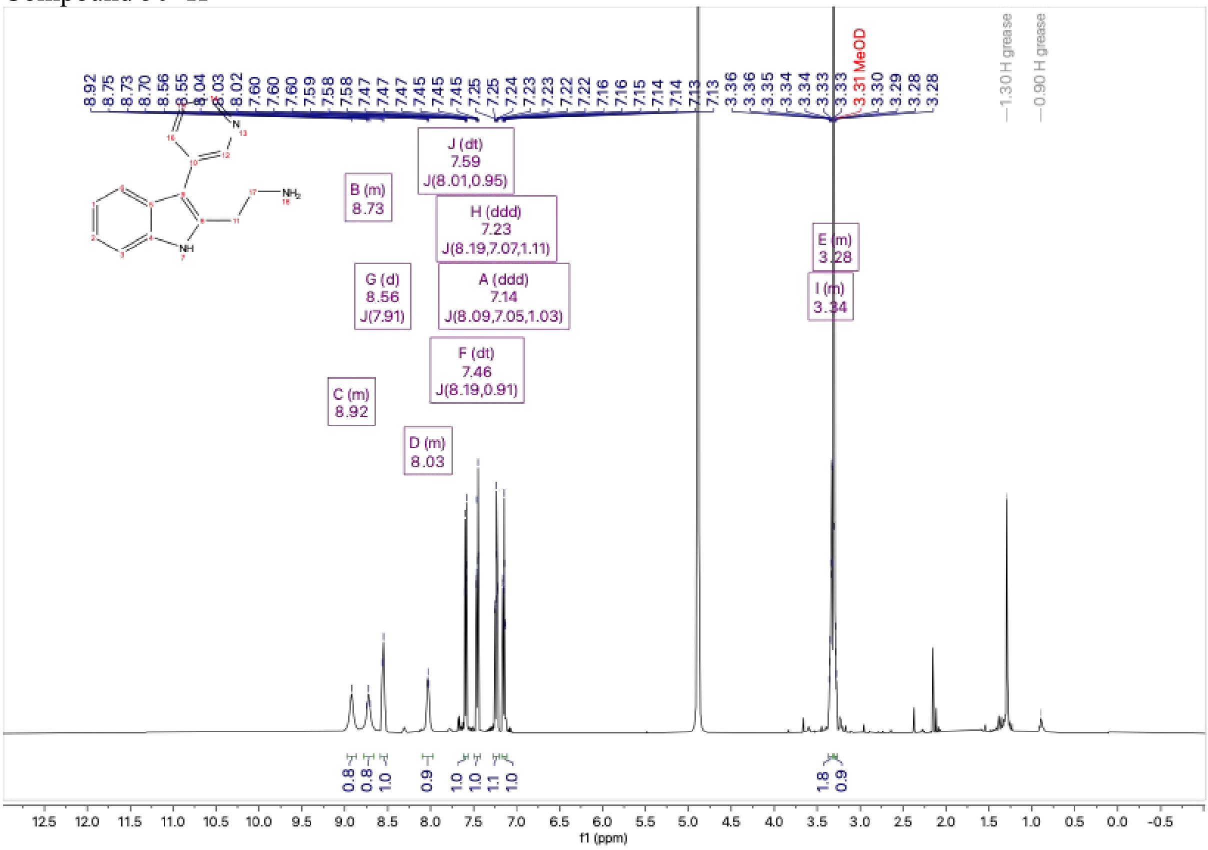

Compound 50 ^{13}C

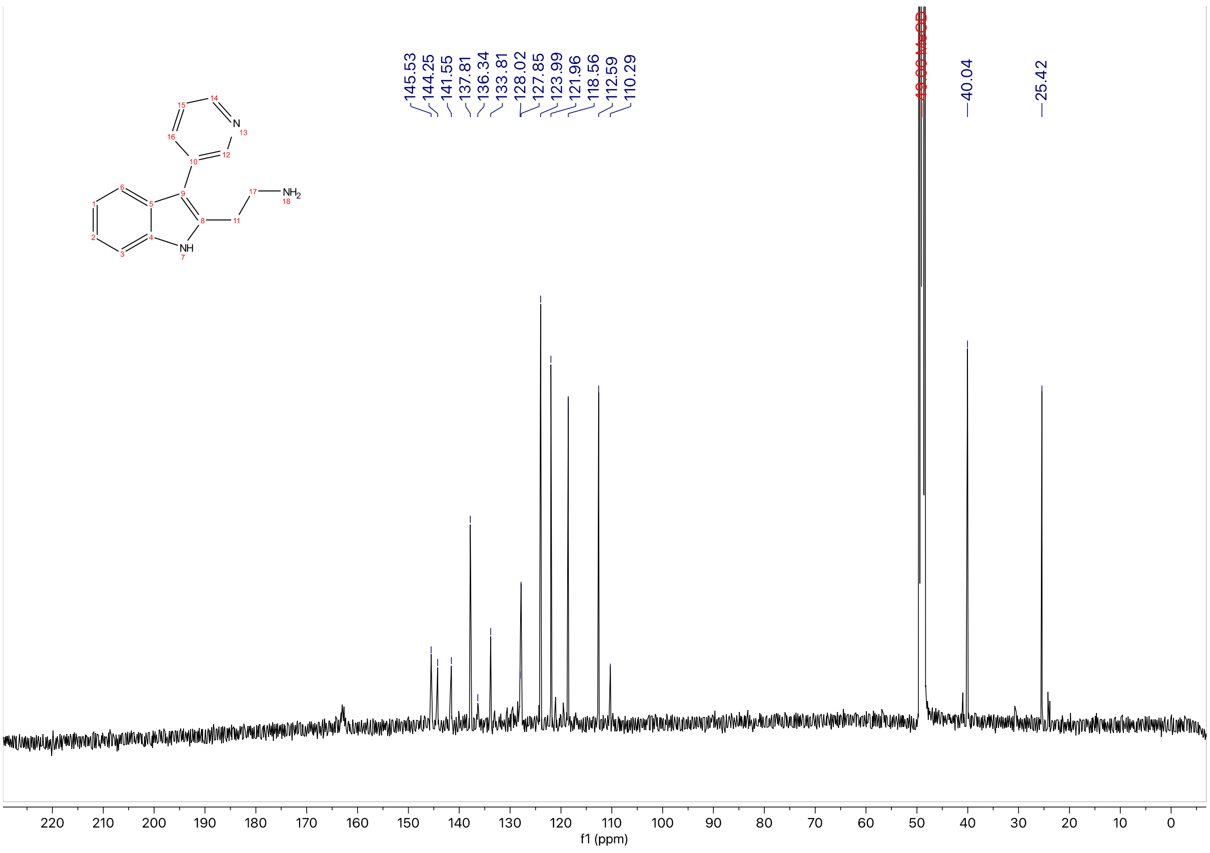

Compound 51 ^{1}H

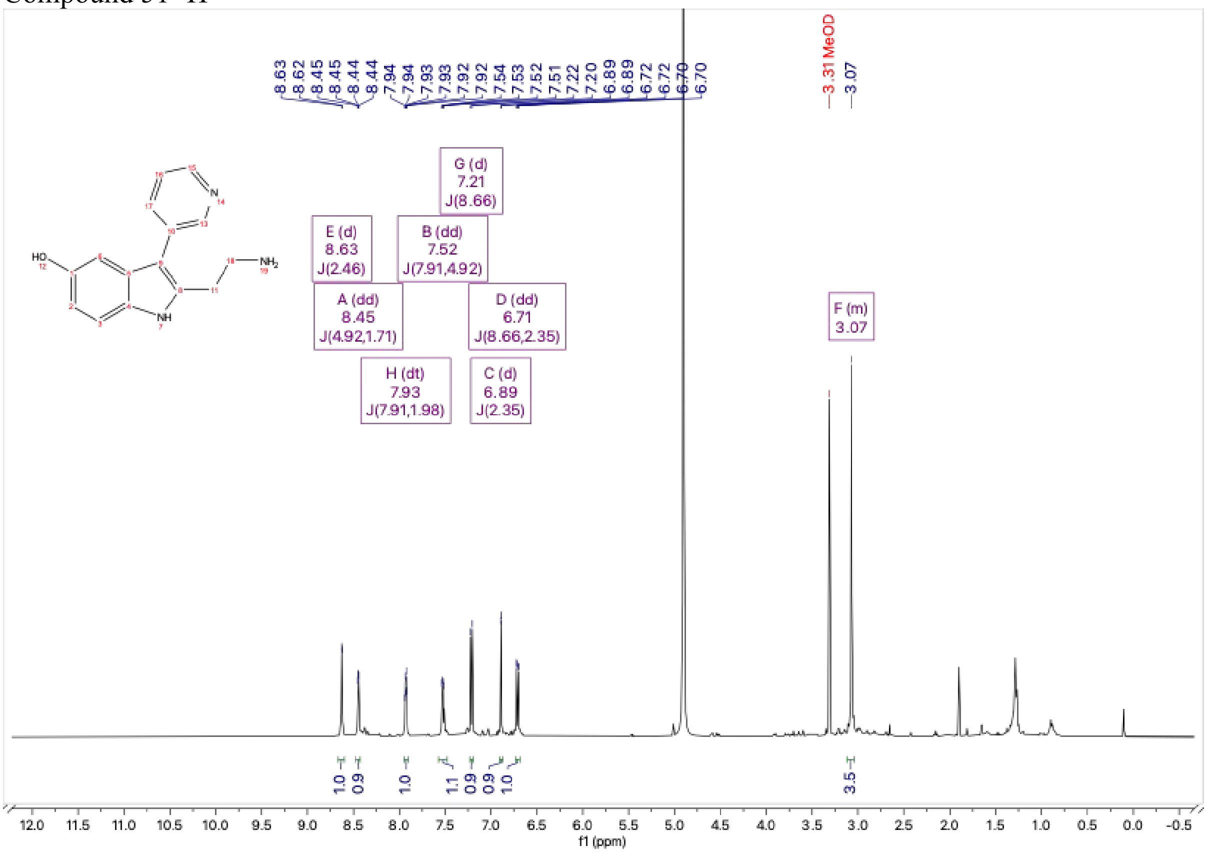

Compound 51 ^{13}C

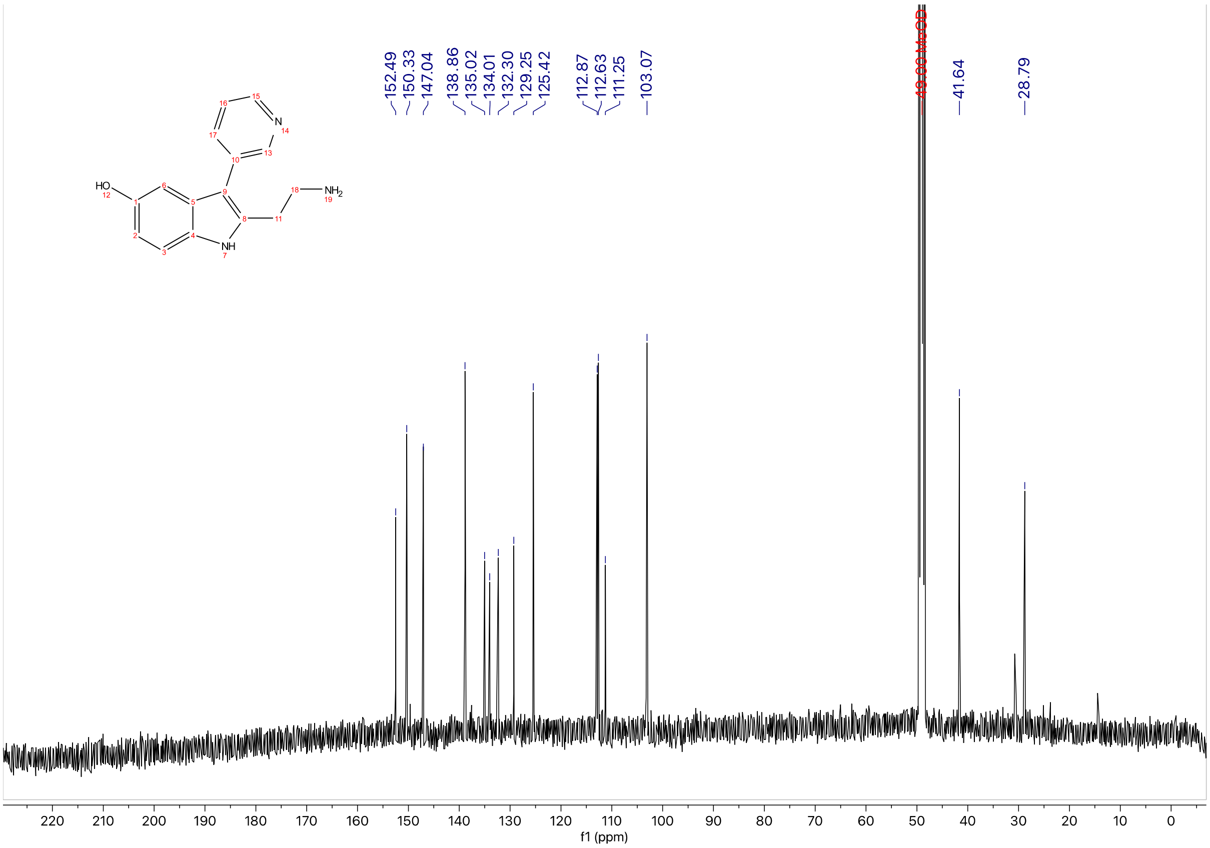

Compound 52 ^{1}H

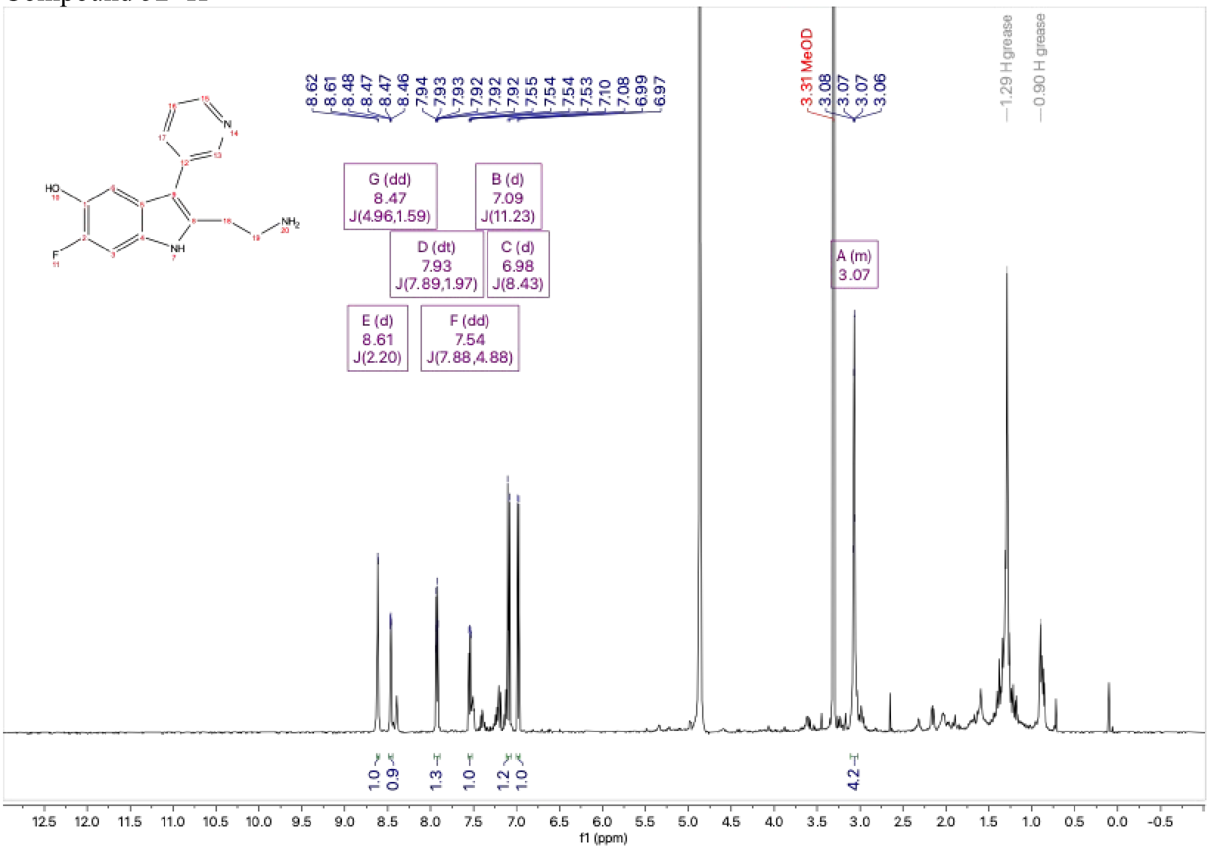

Compound 52 ^{13}C

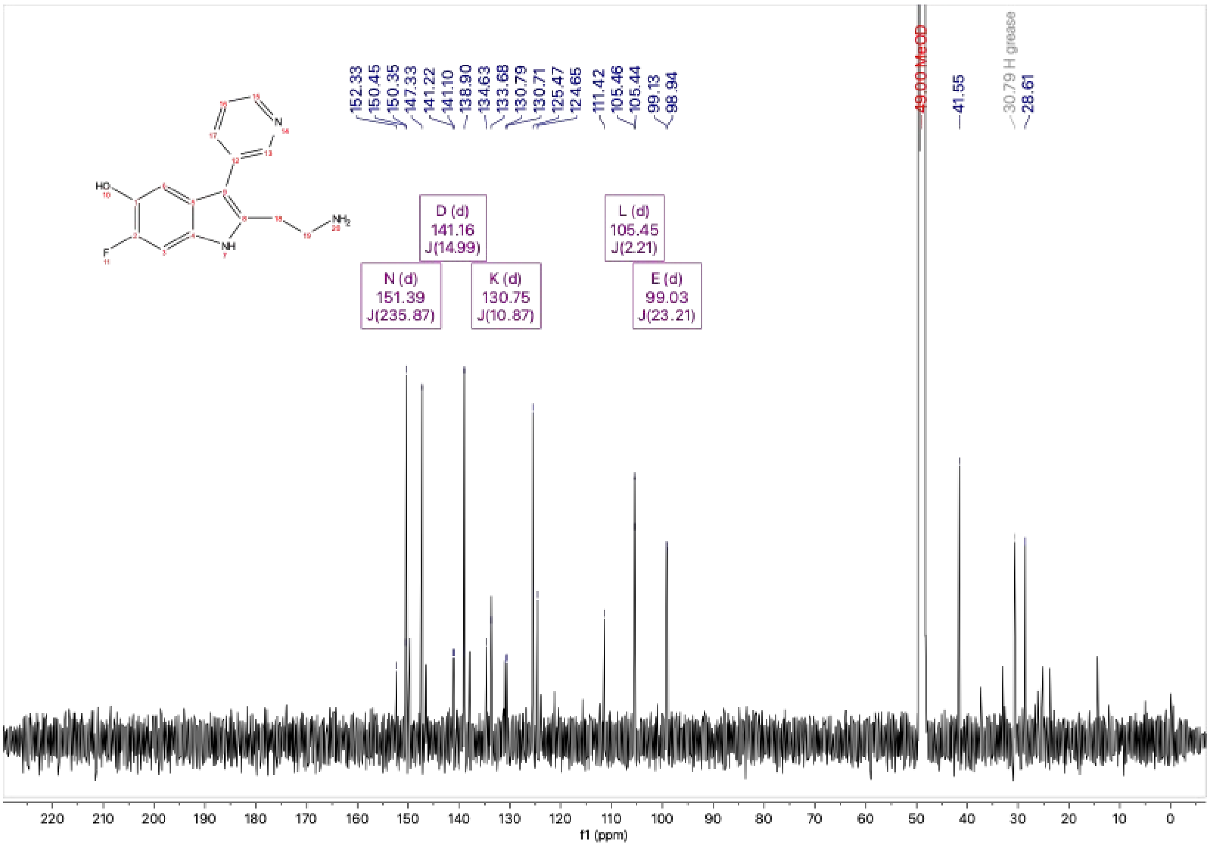

Compound 52 ^{19}F

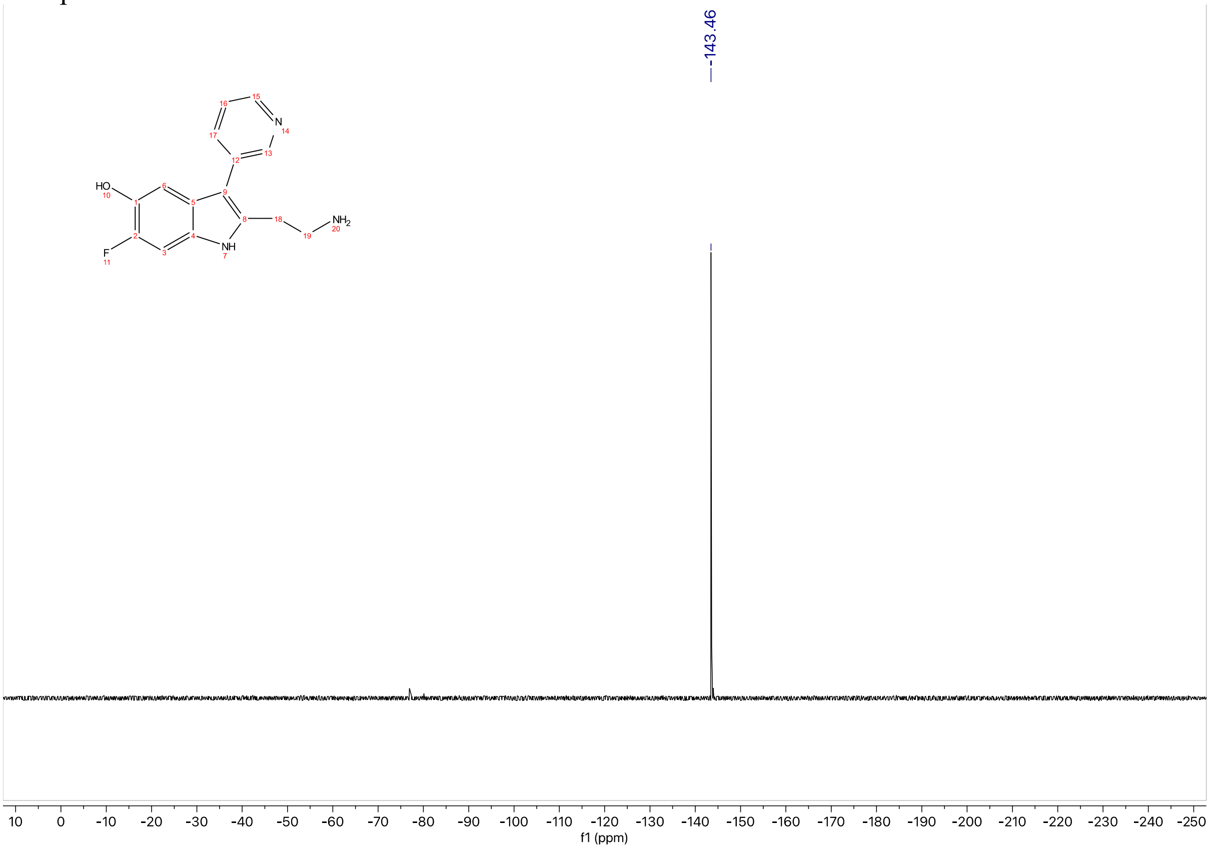

Compound 53 ^{1}H

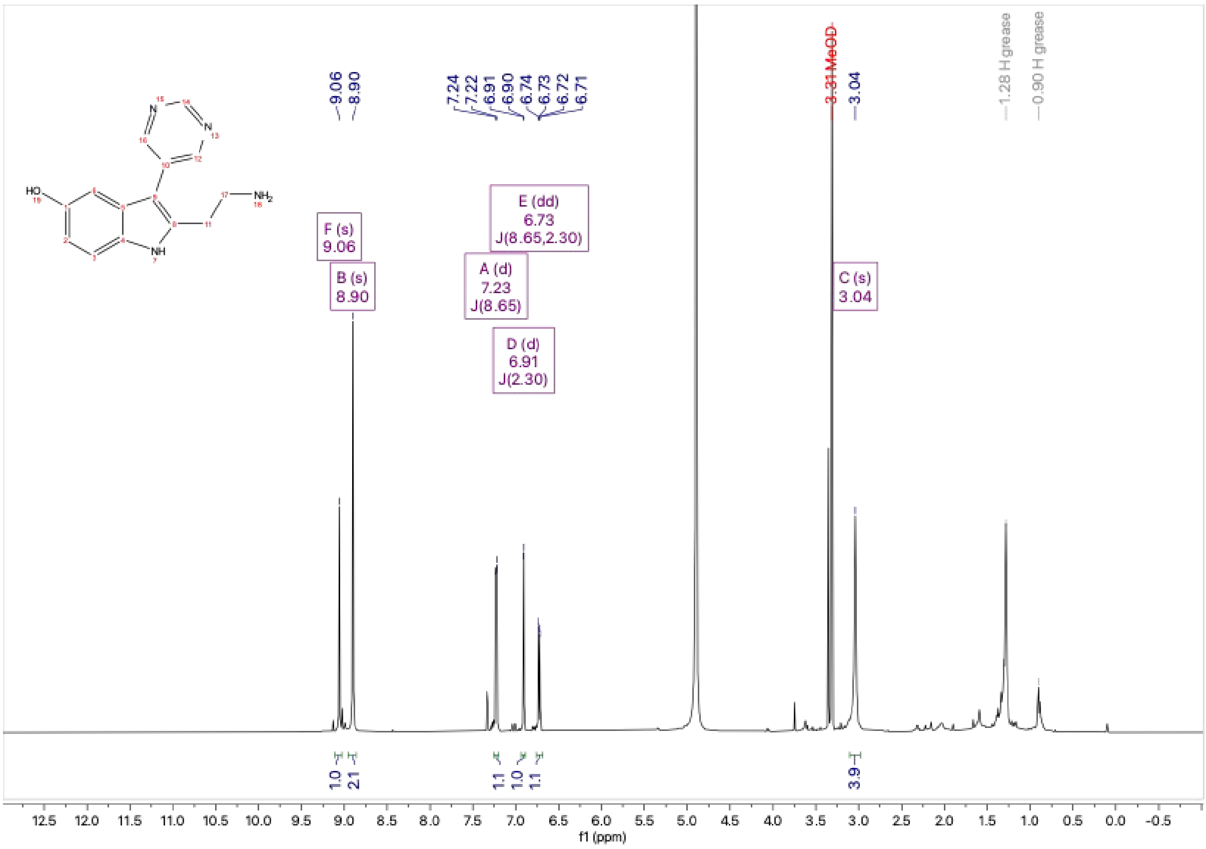

Compound 53 ^{13}C

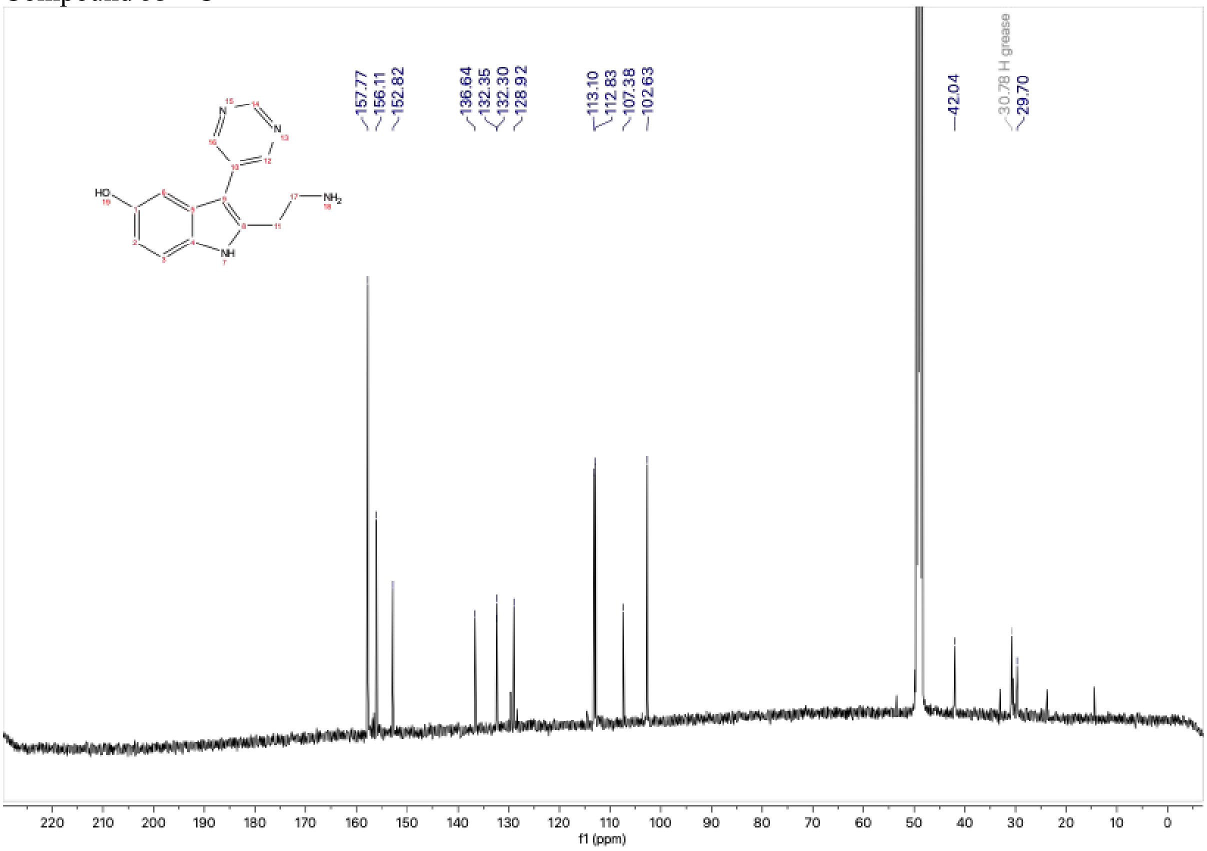

Compound 29a ^{1}H

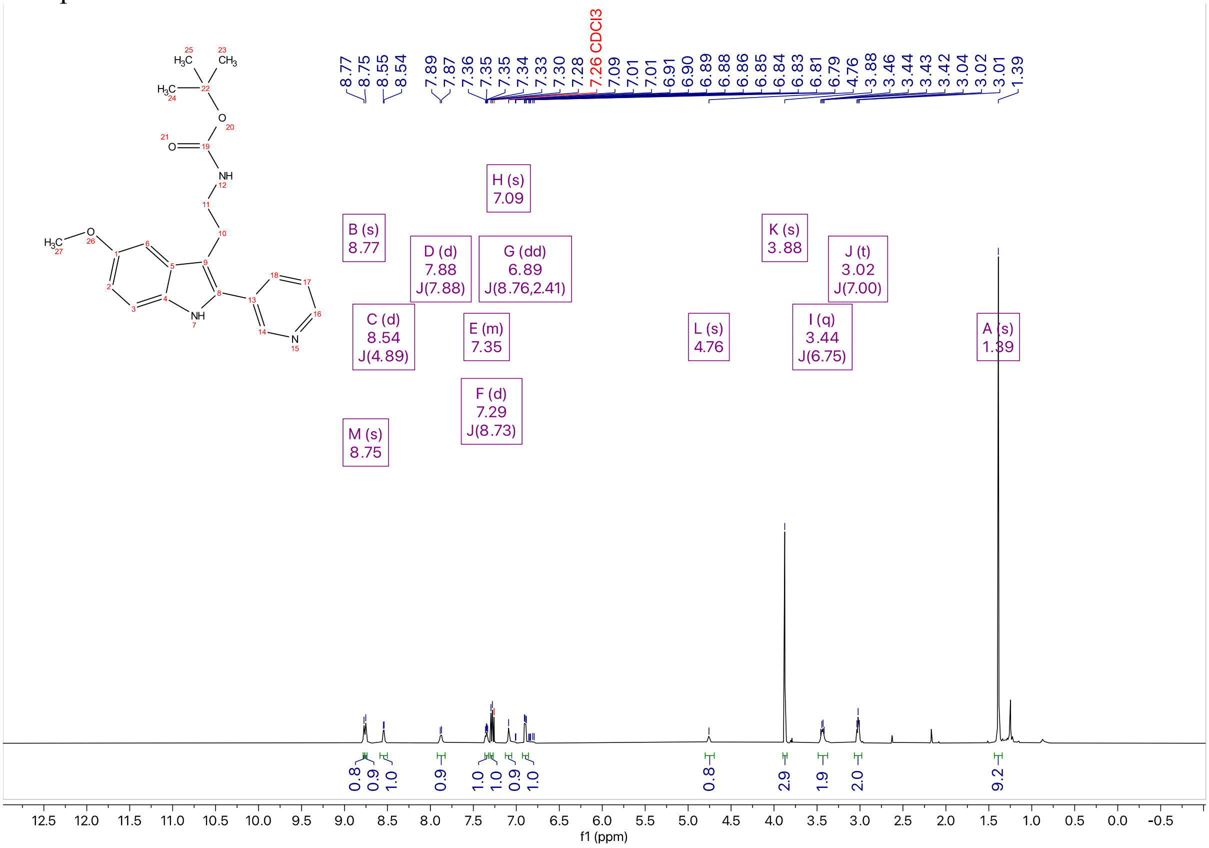

Compound 29a ^{13}C

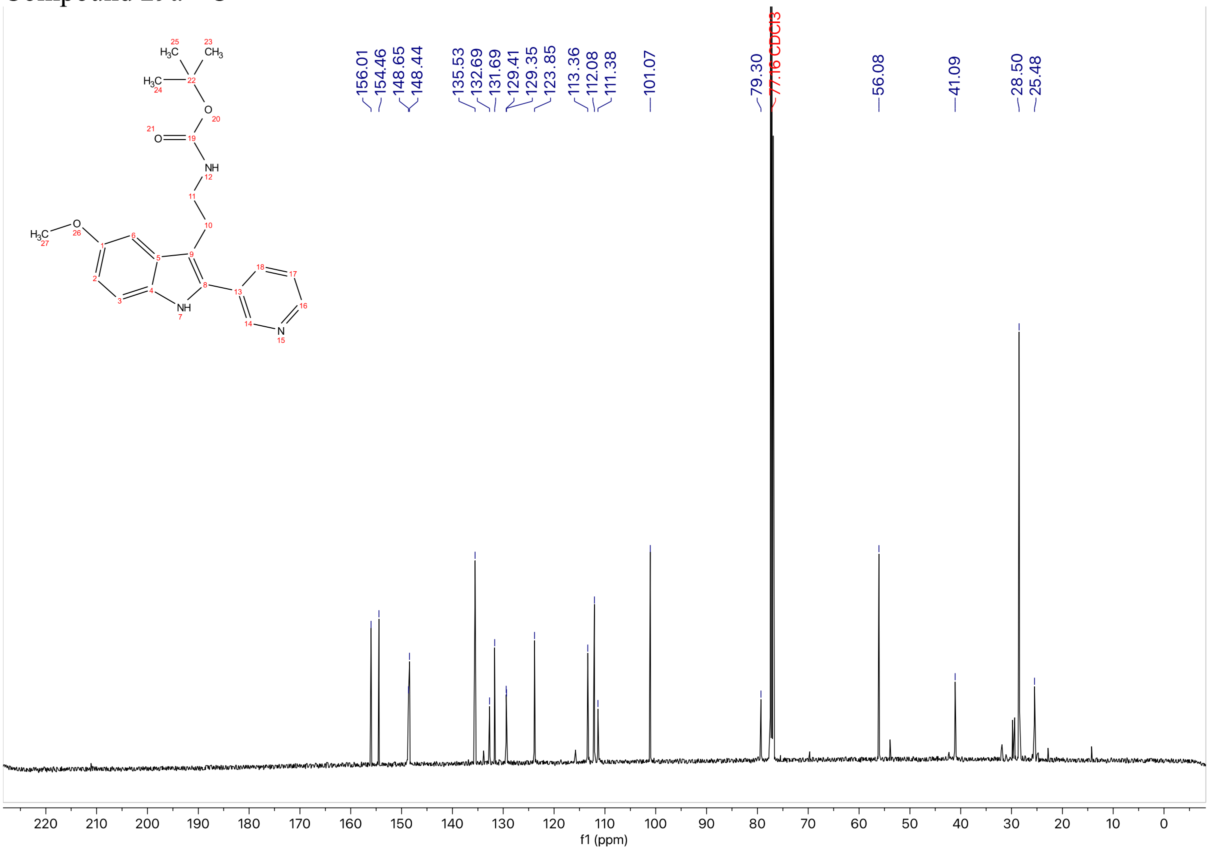

Compound 29b ¹H

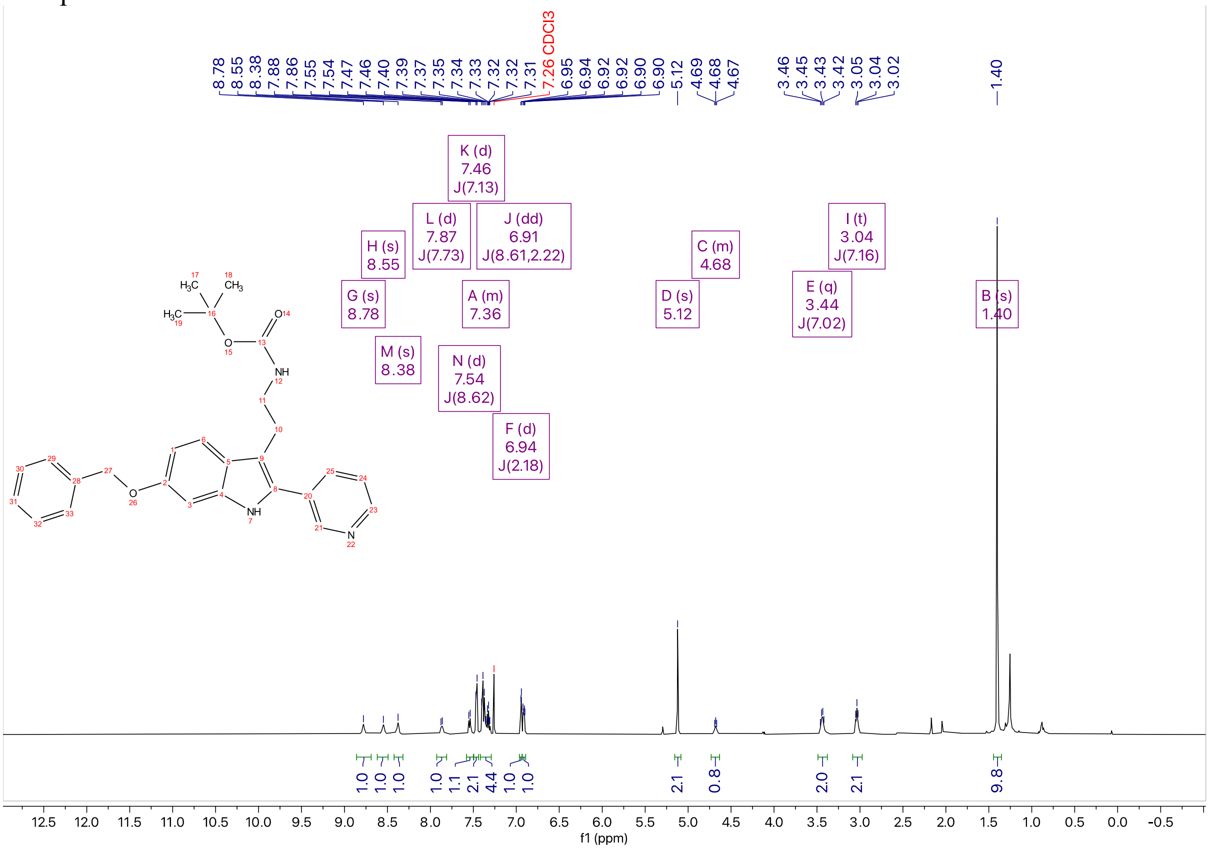

Compound 29b ¹³C

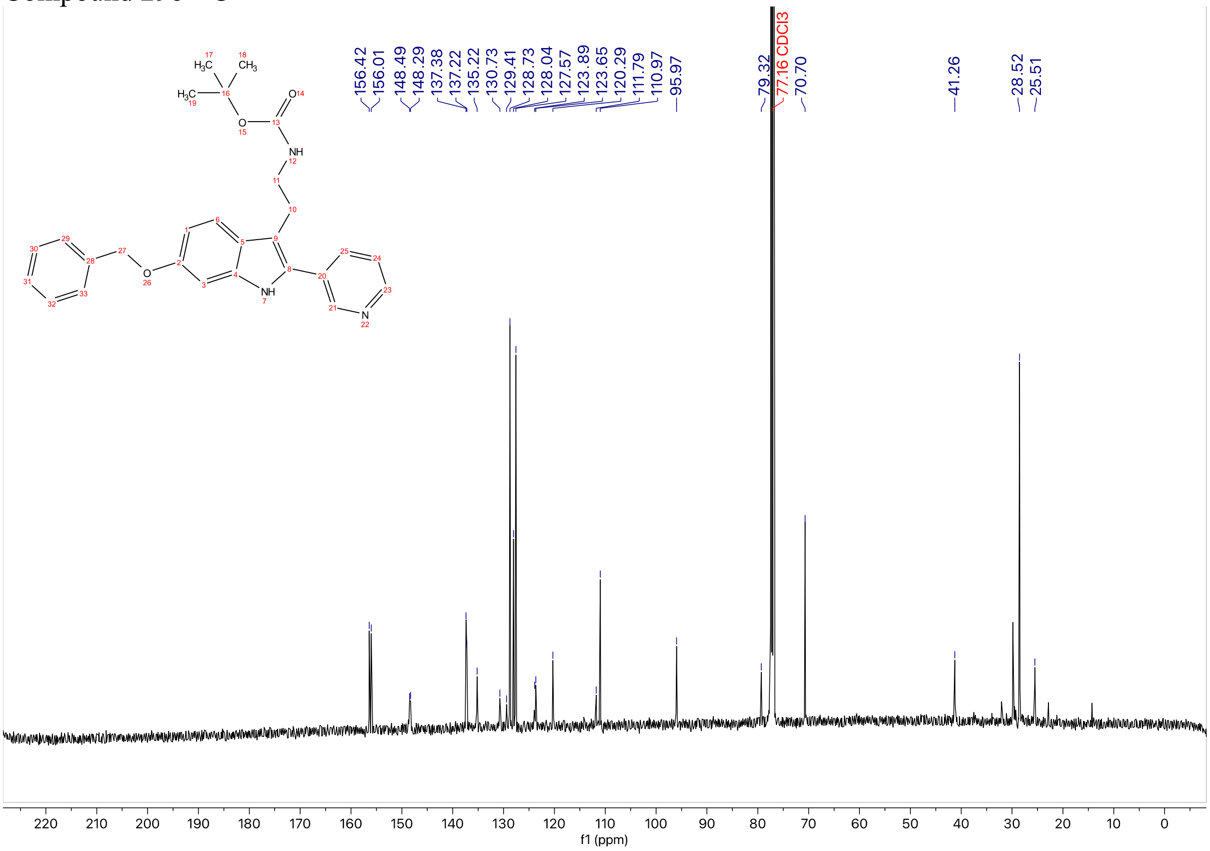

Compound 29c ¹H

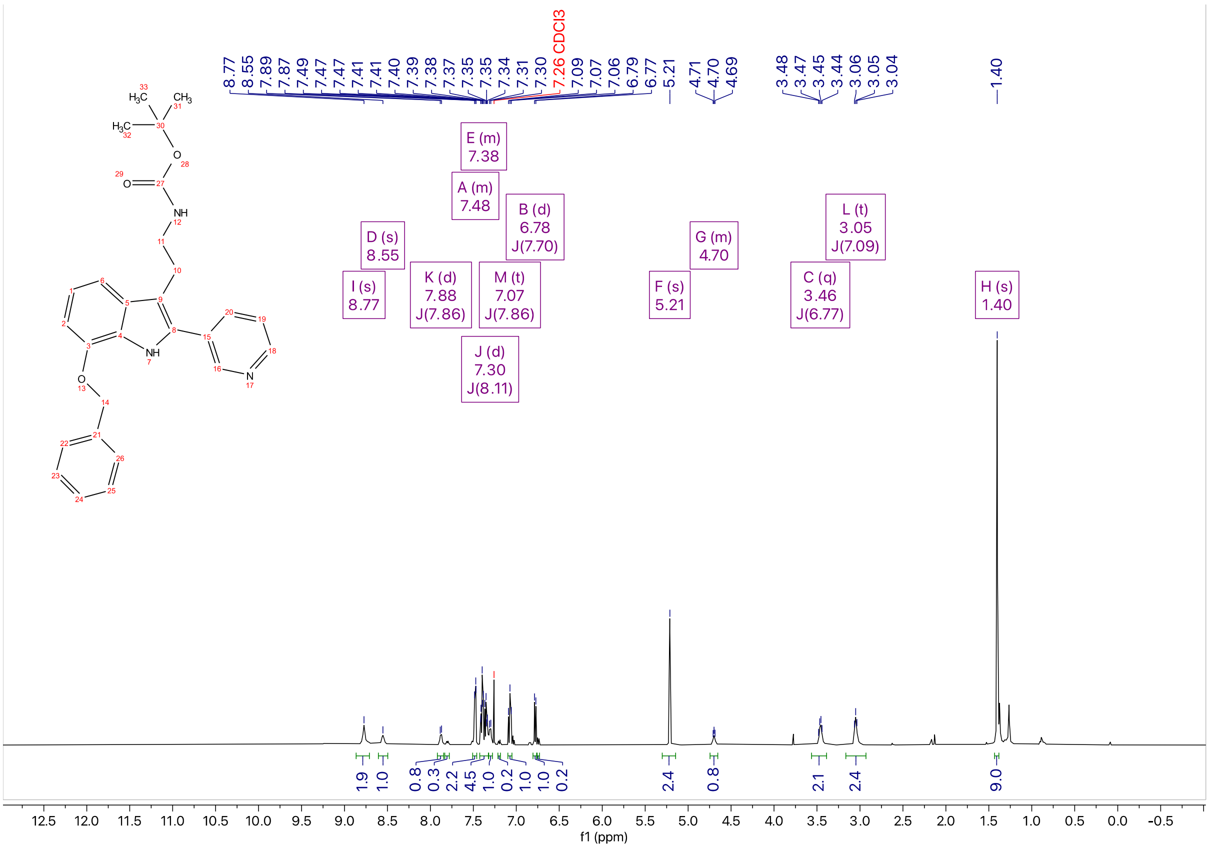

Compound 29c ^{13}C

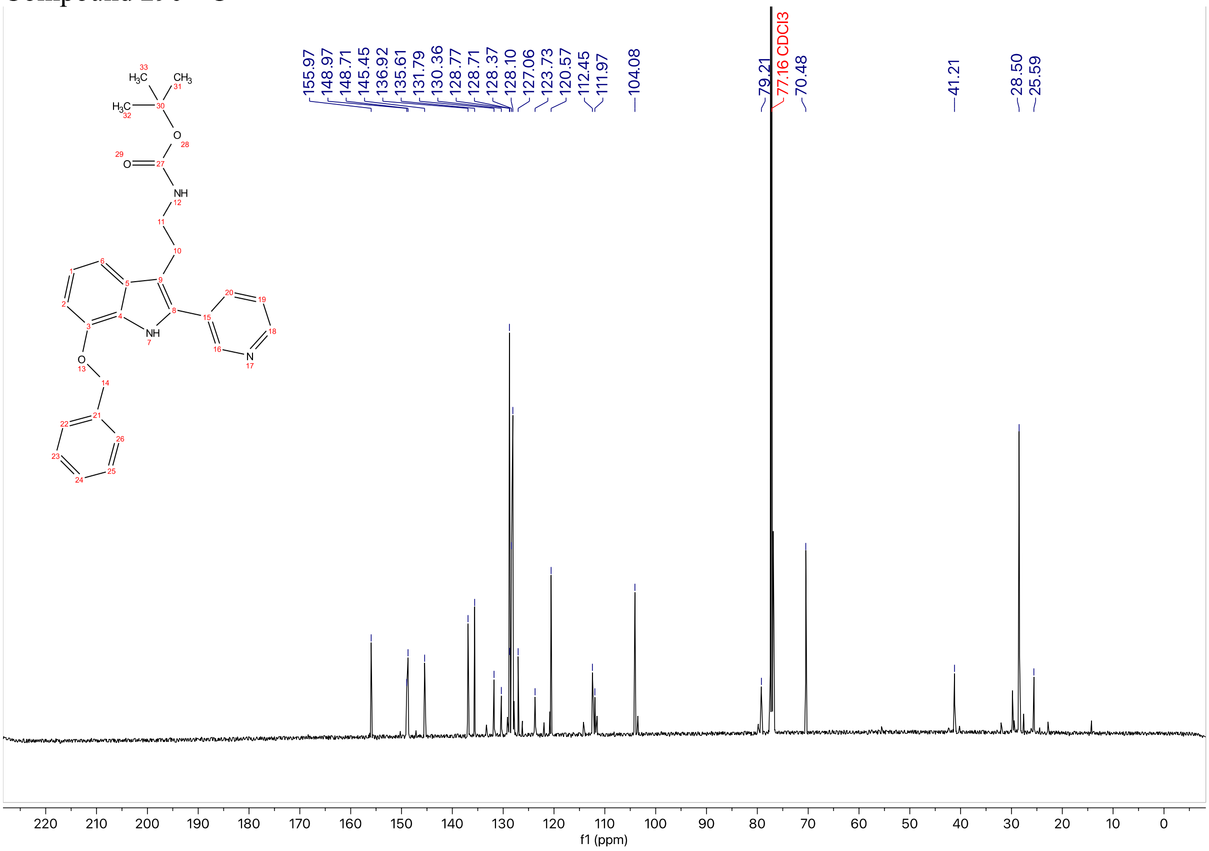

Compound 29d ^{1}H

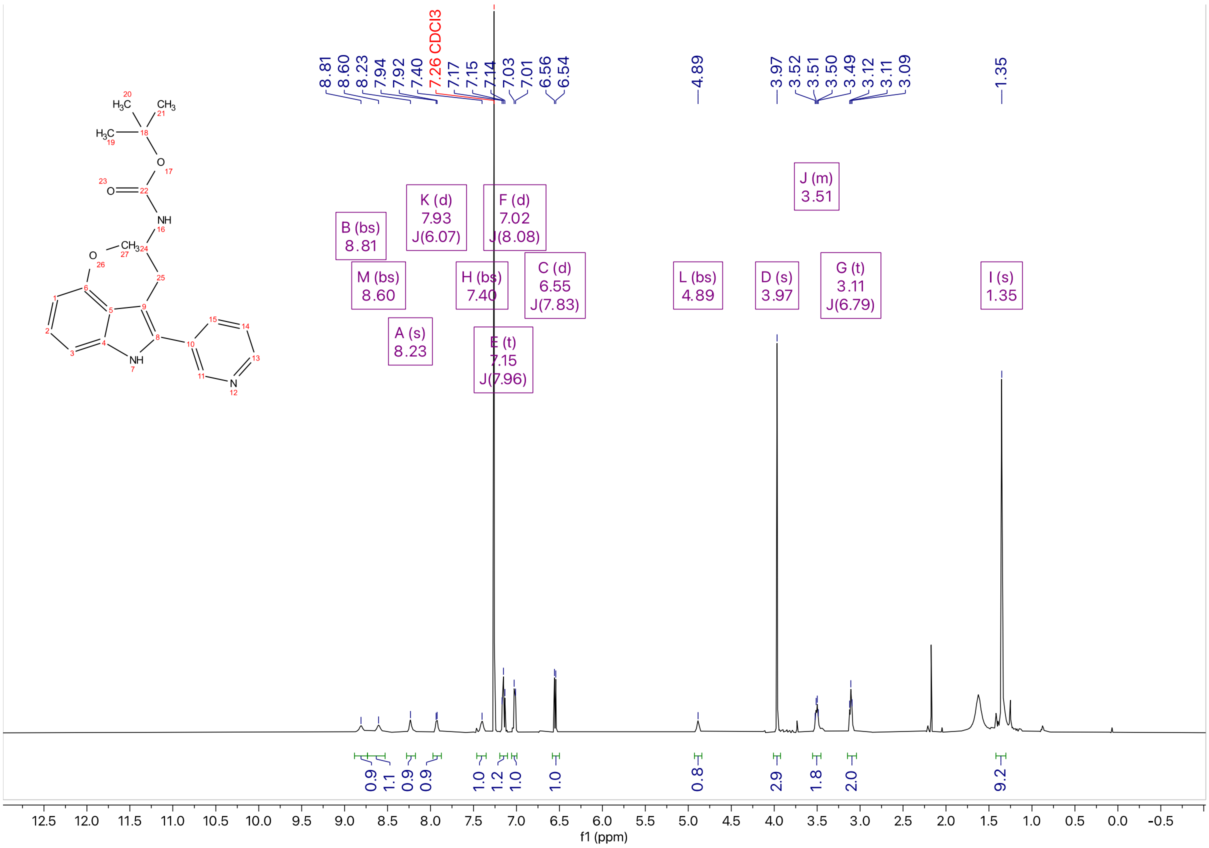

Compound 29d ^{13}C

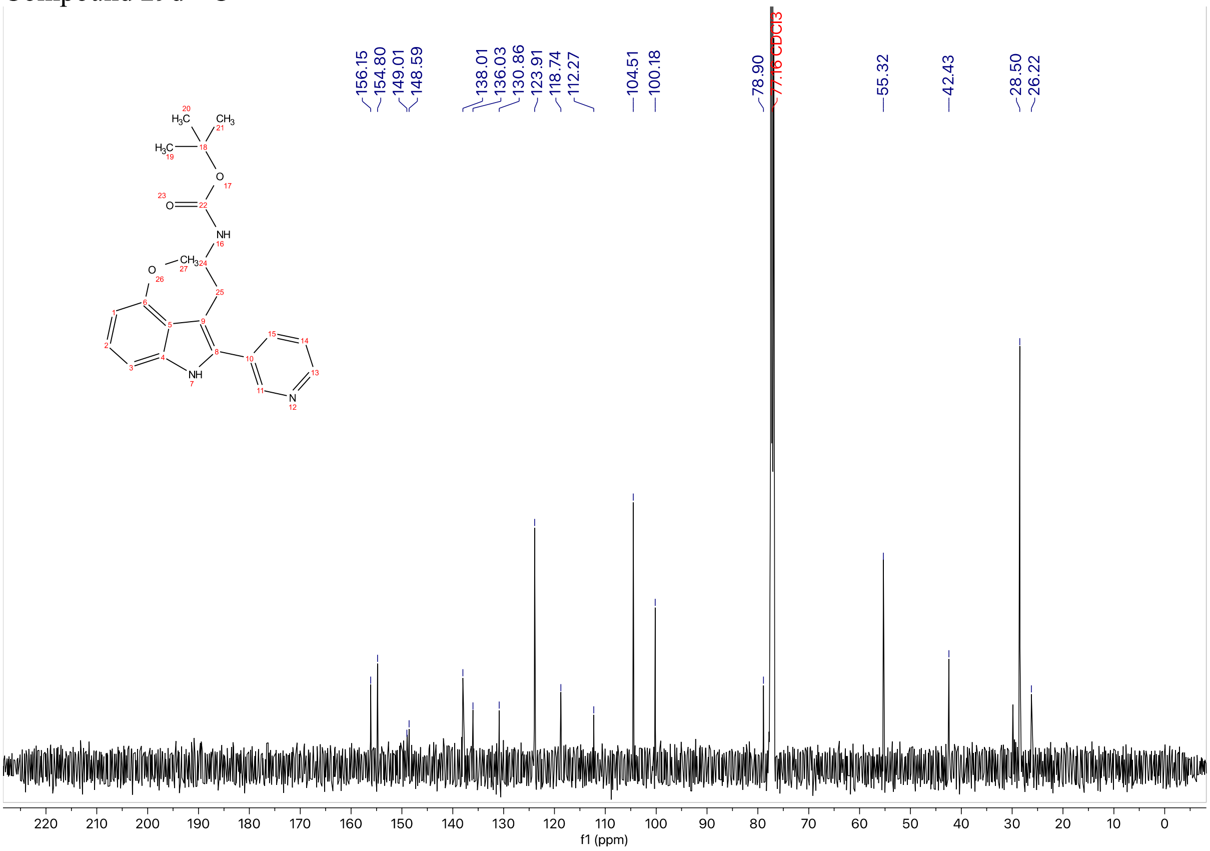

Compound 29e ^{1}H

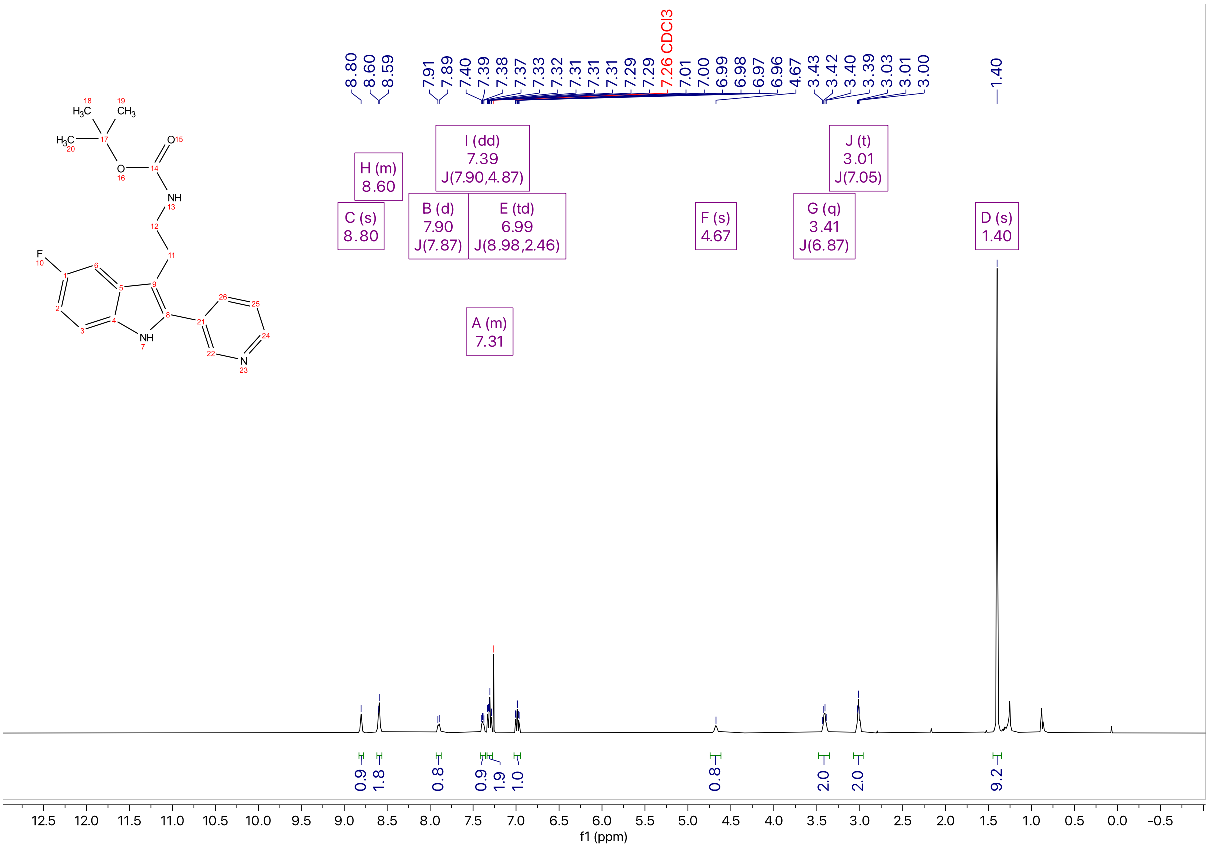

Compound 29e ^{13}C

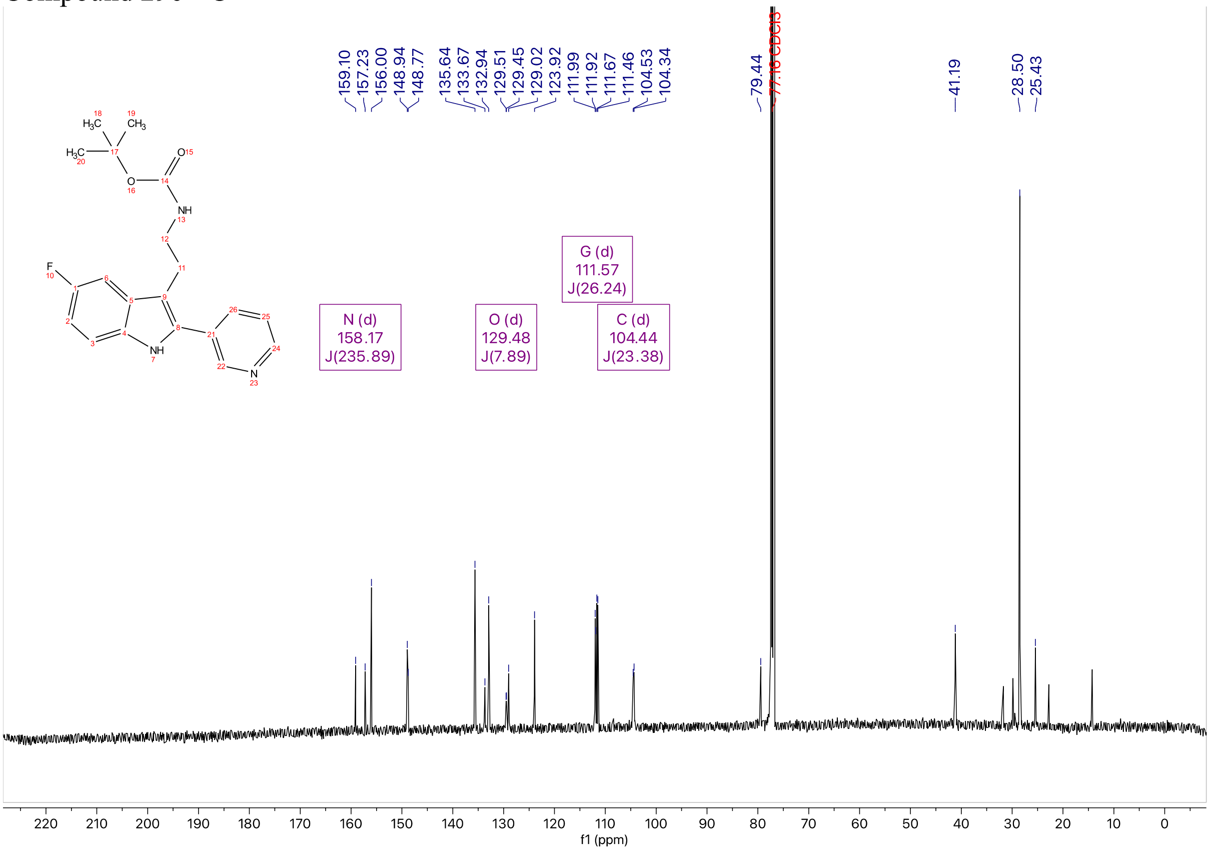

Compound 29e ^{19}F

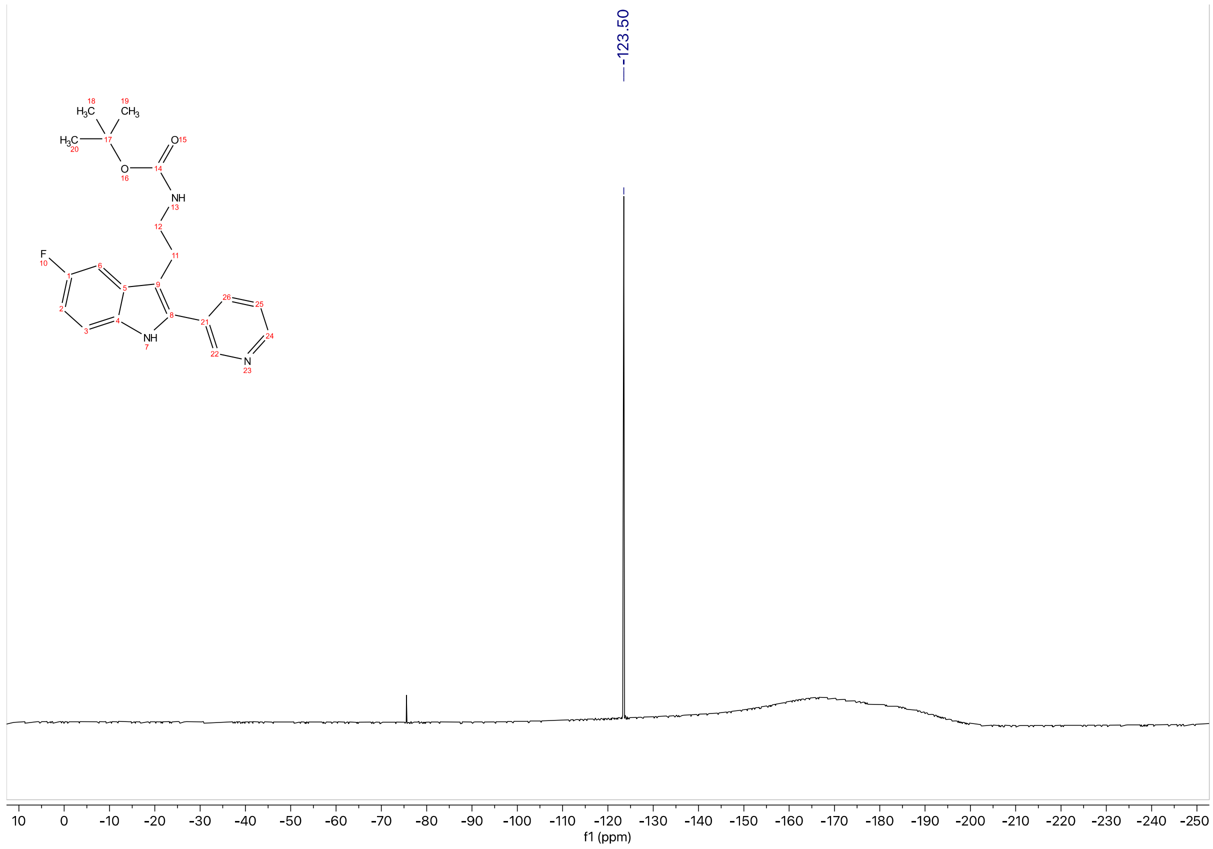

Compound 29f ^{1}H

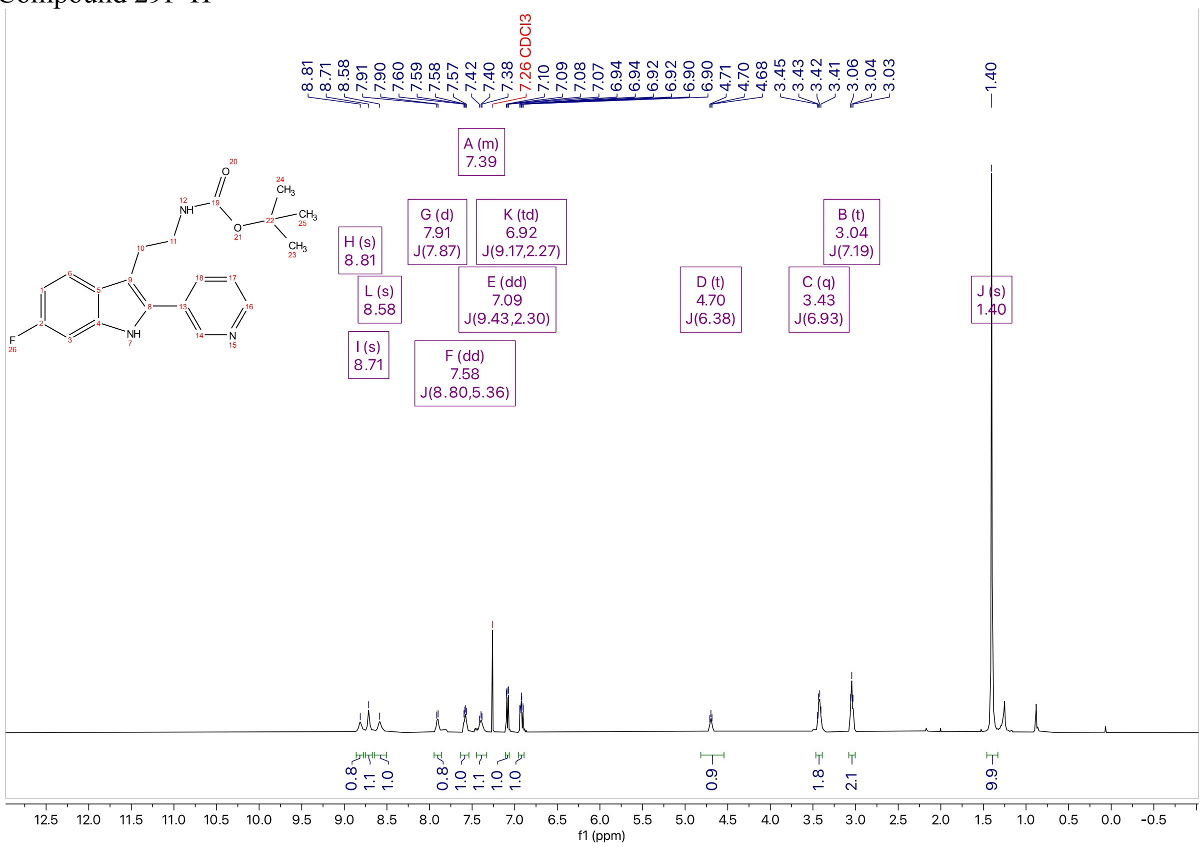

Compound 29f ^{13}C

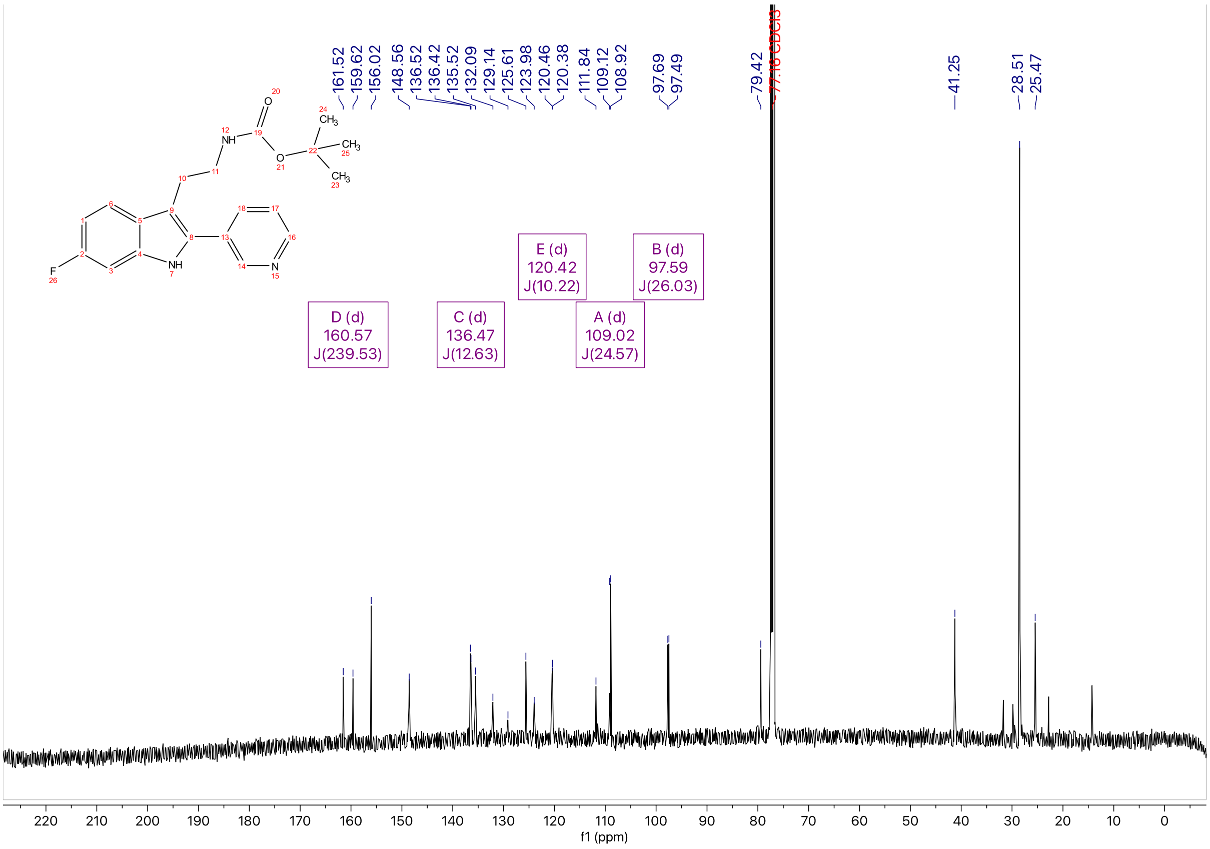

Compound 29f ^{19}F

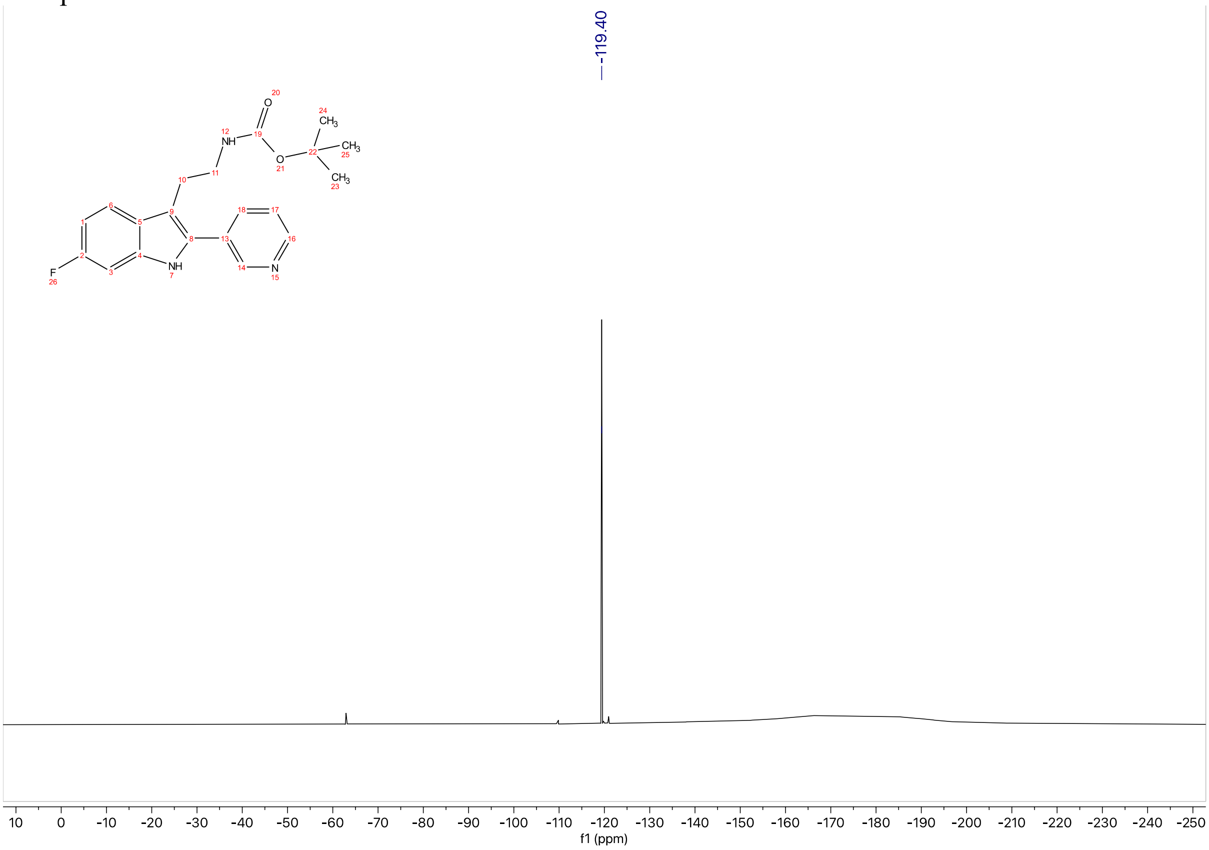

Compound 29g ^{1}H

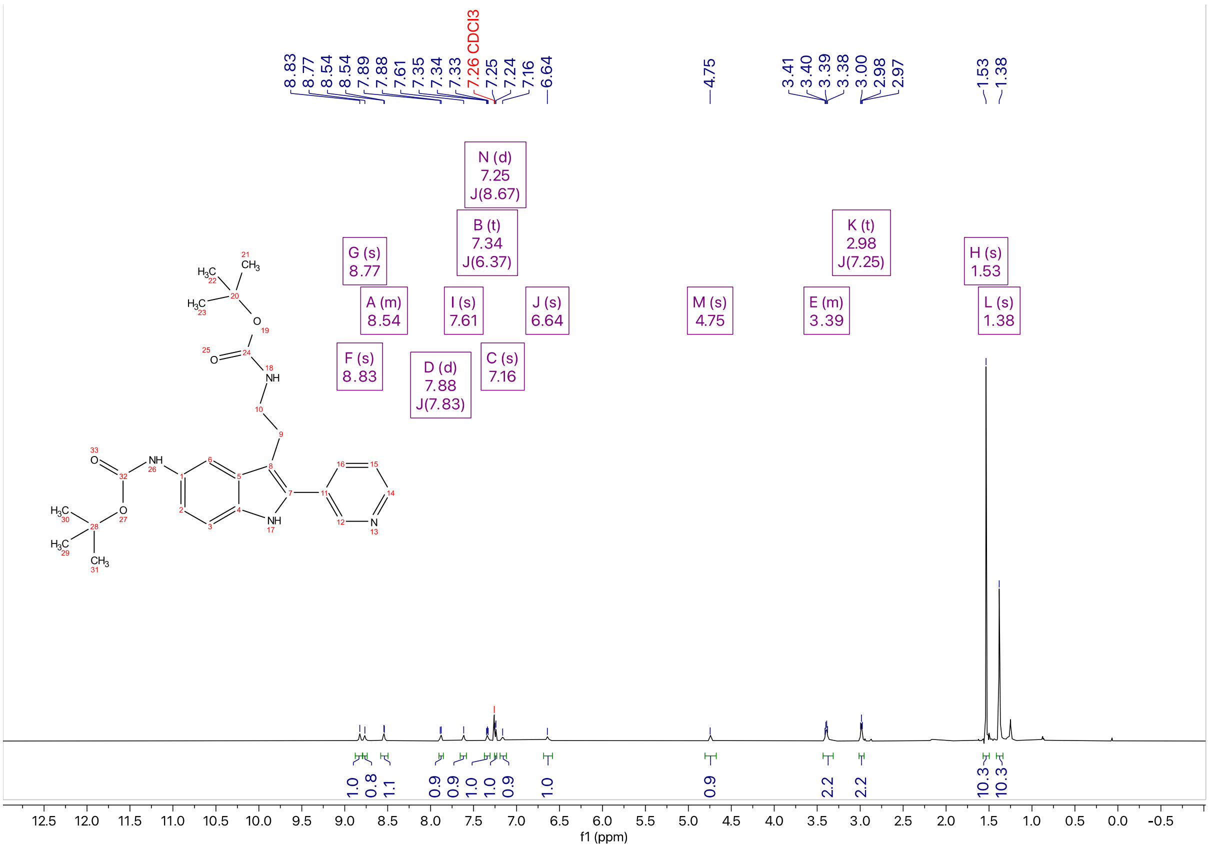

Compound 29g ^{13}C

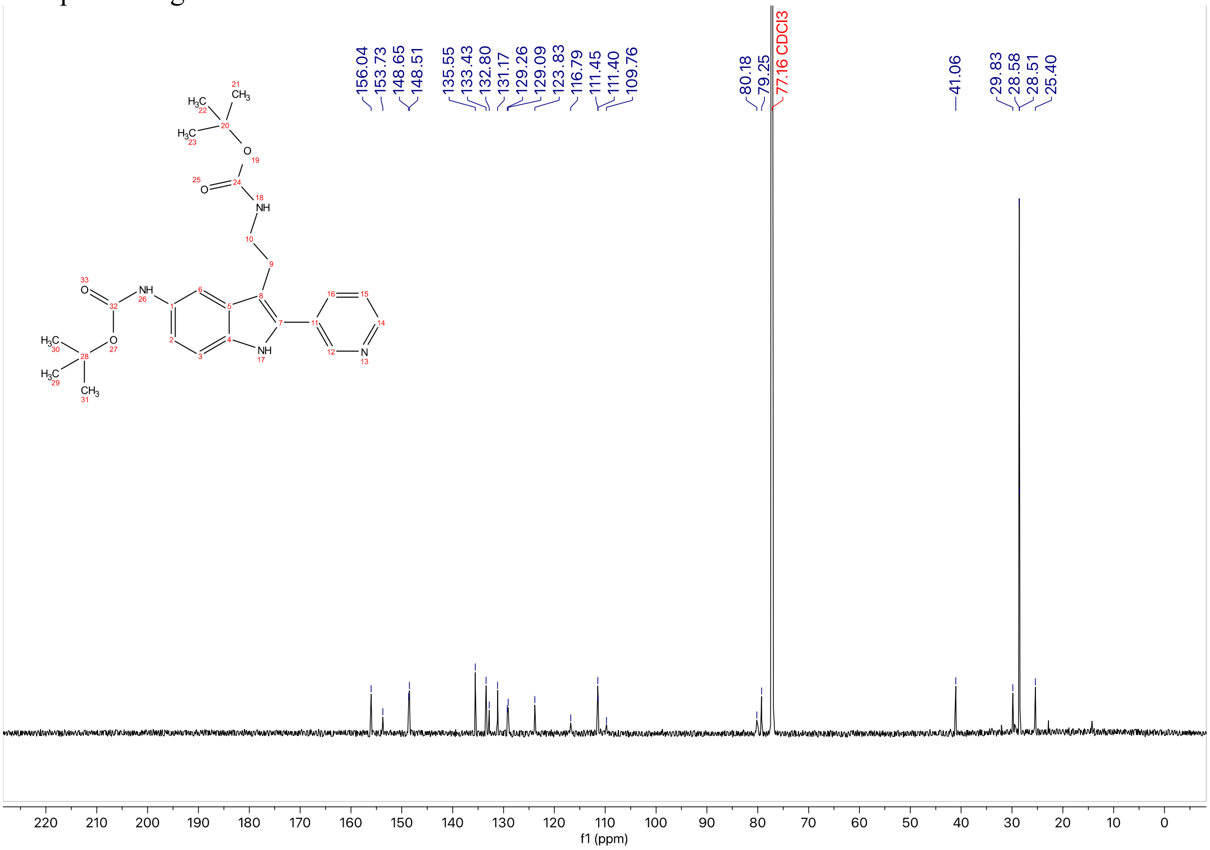

Compound 29h ^{1}H

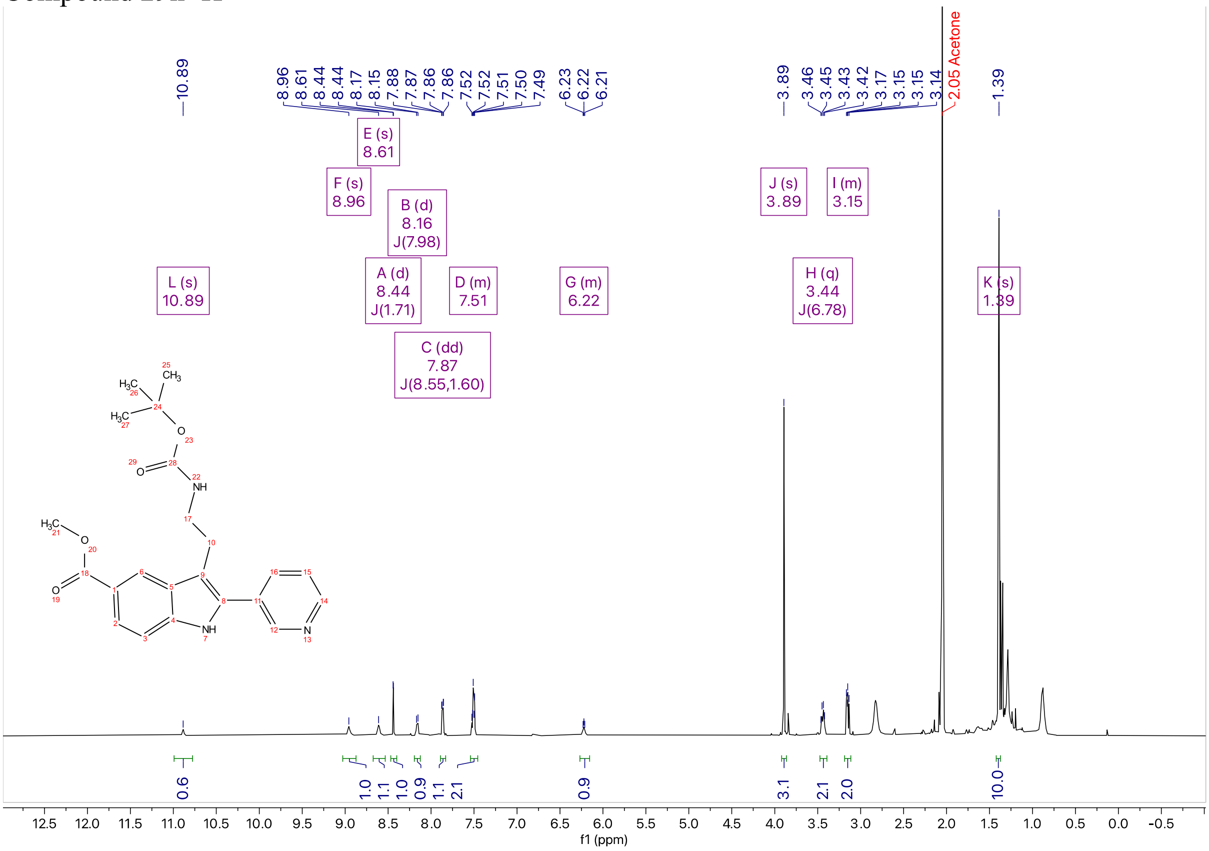

Compound 29h ^{13}C

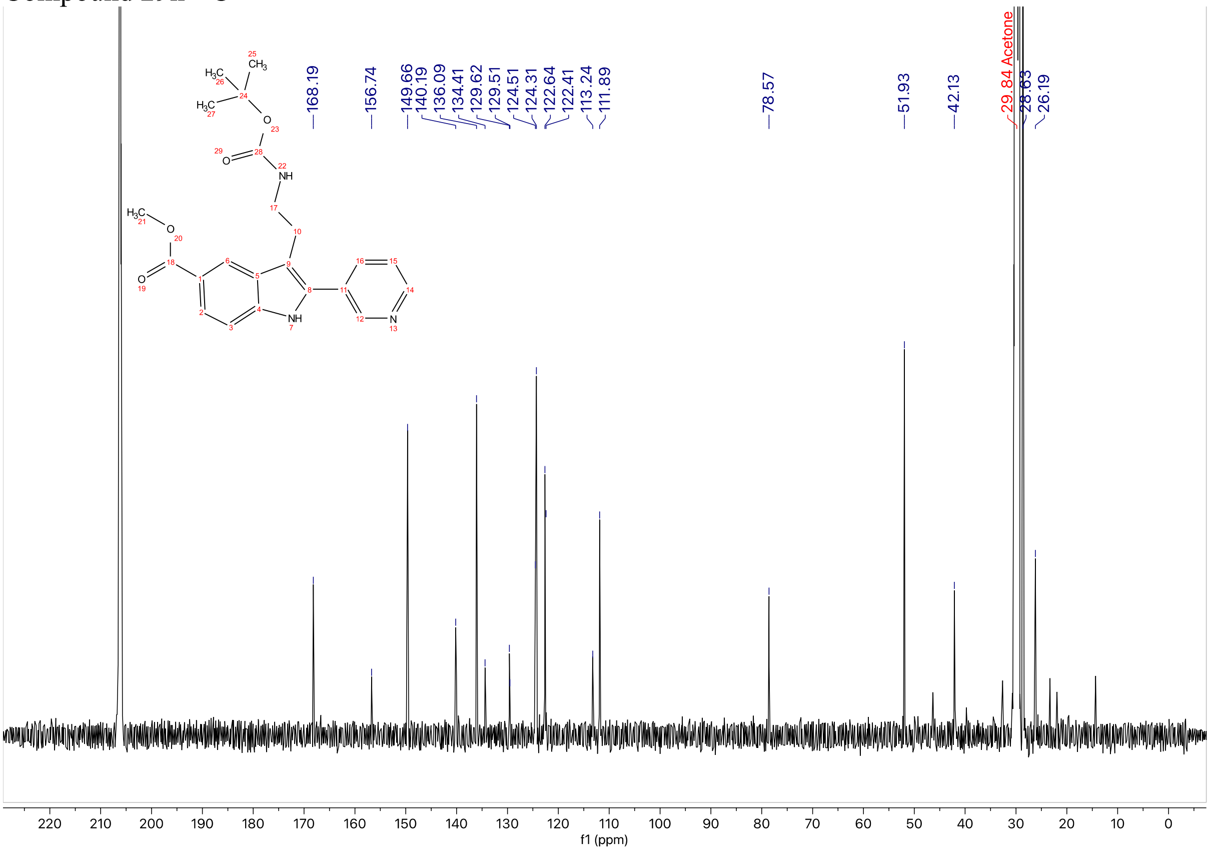

Compound 29i ^{1}H

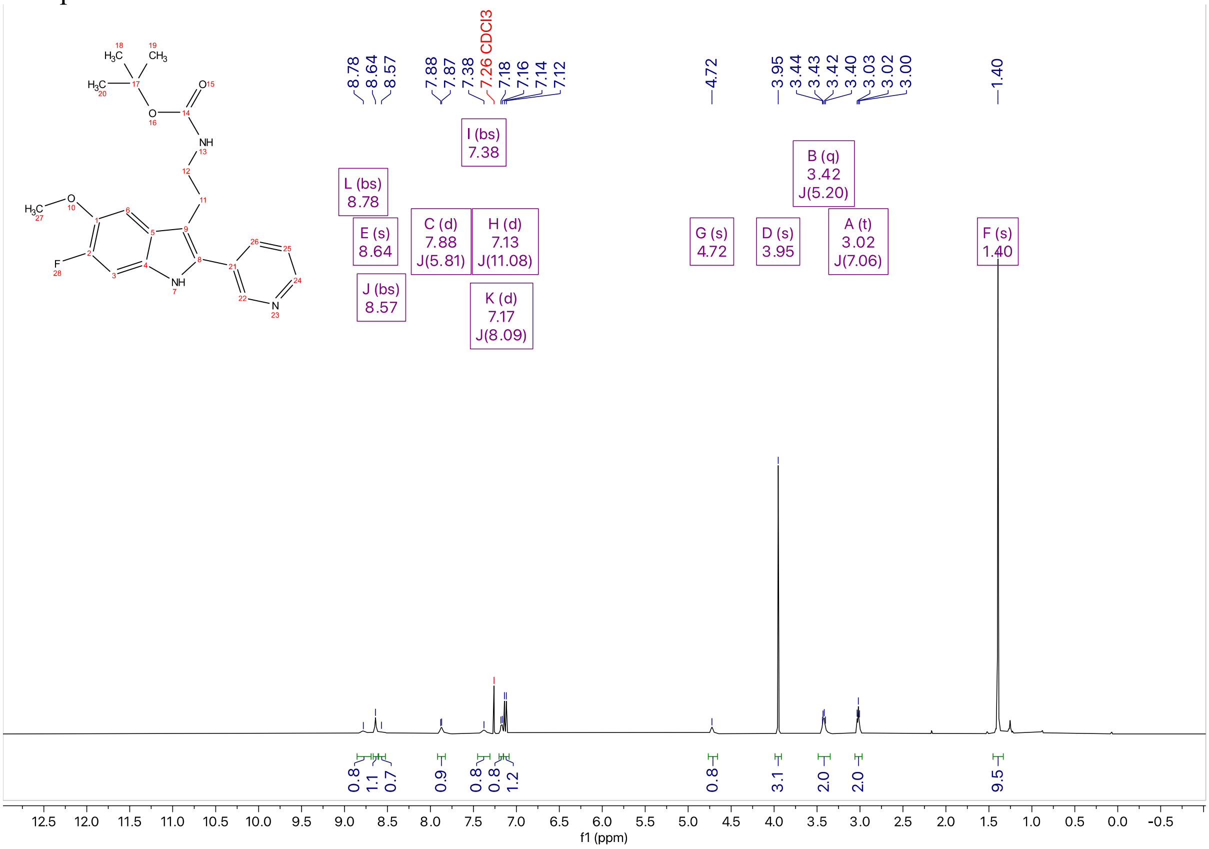

Compound 29i ^{13}C

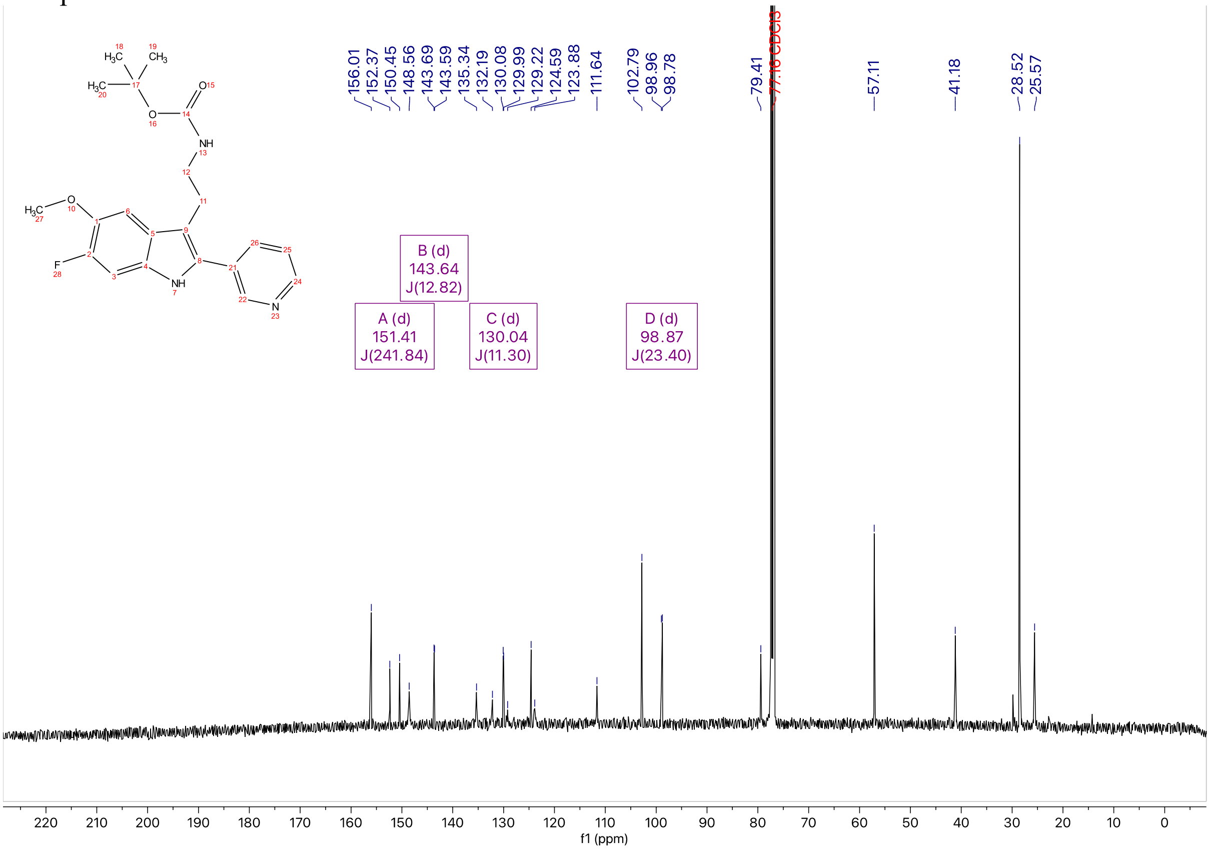

Compound 29i ^{19}F

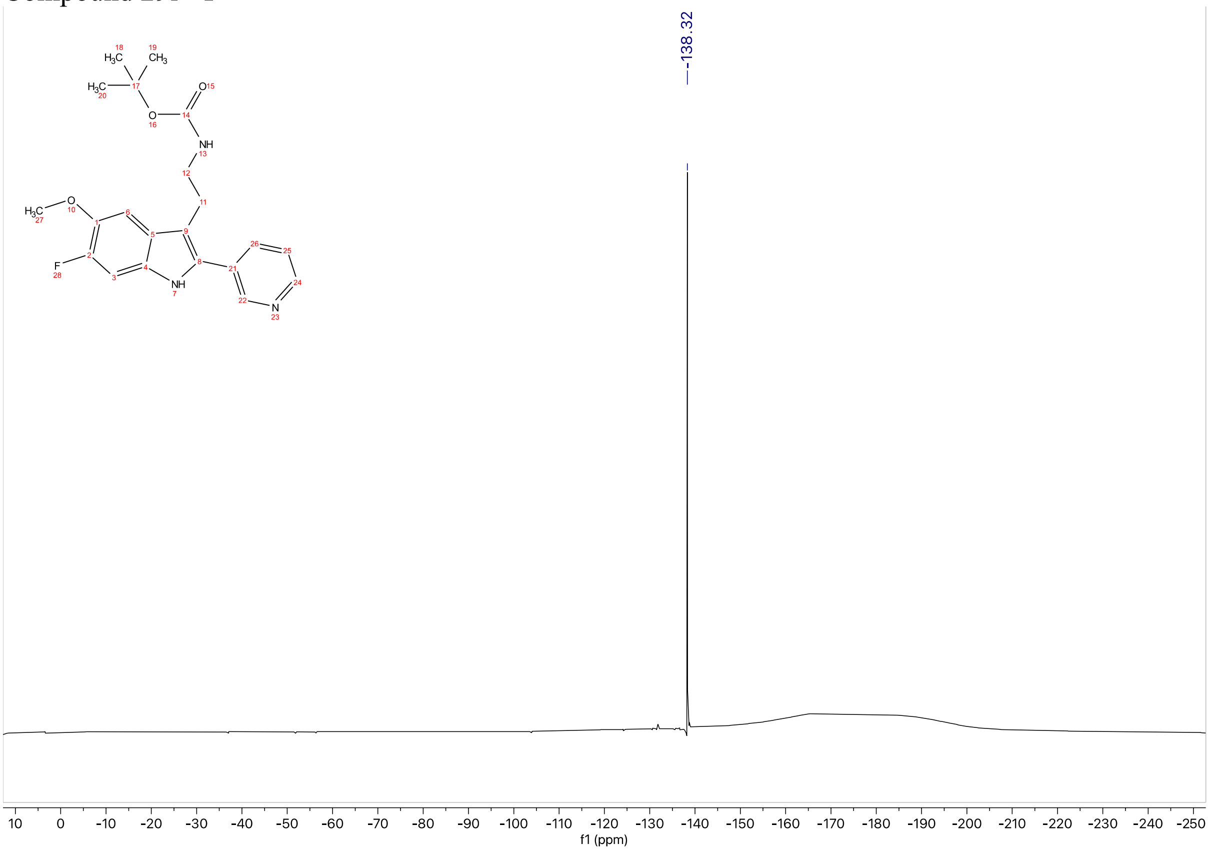

Compound 29j ^{1}H

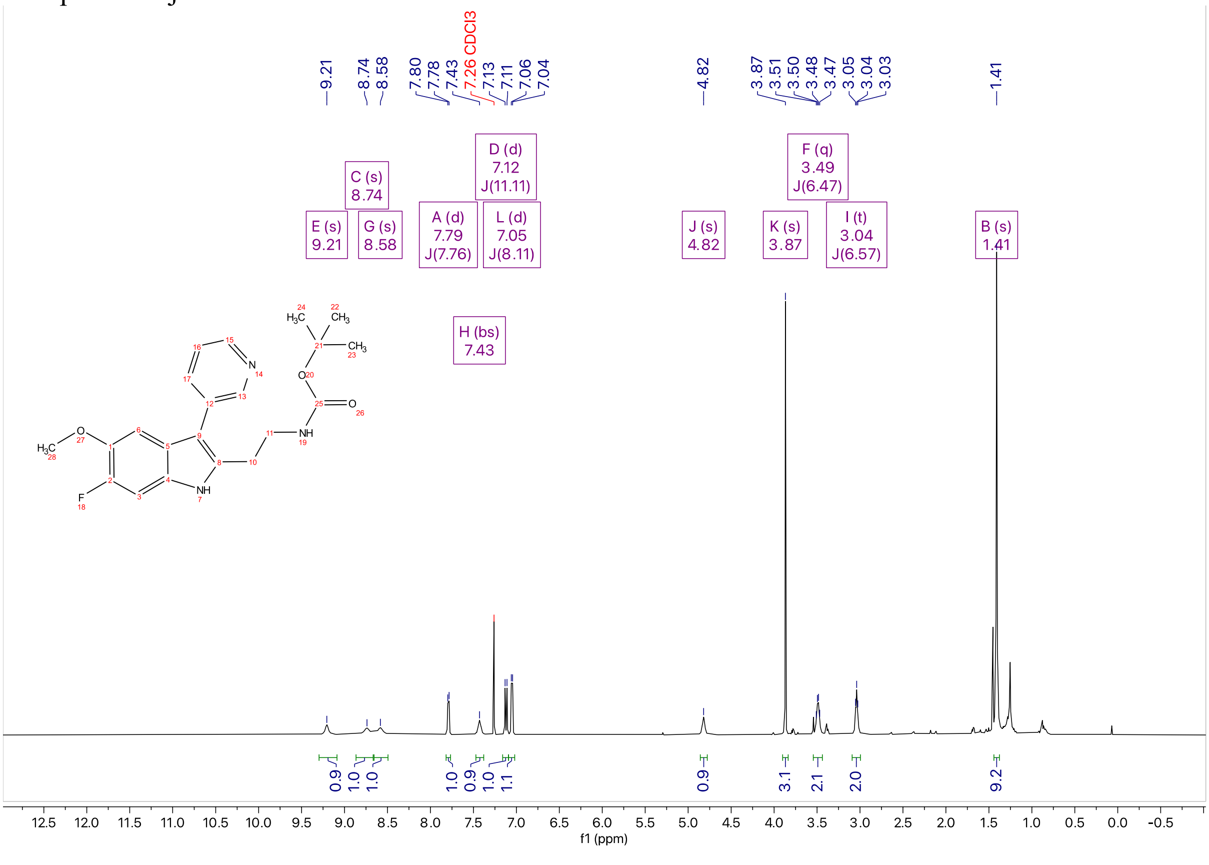

Compound 29j ^{13}C

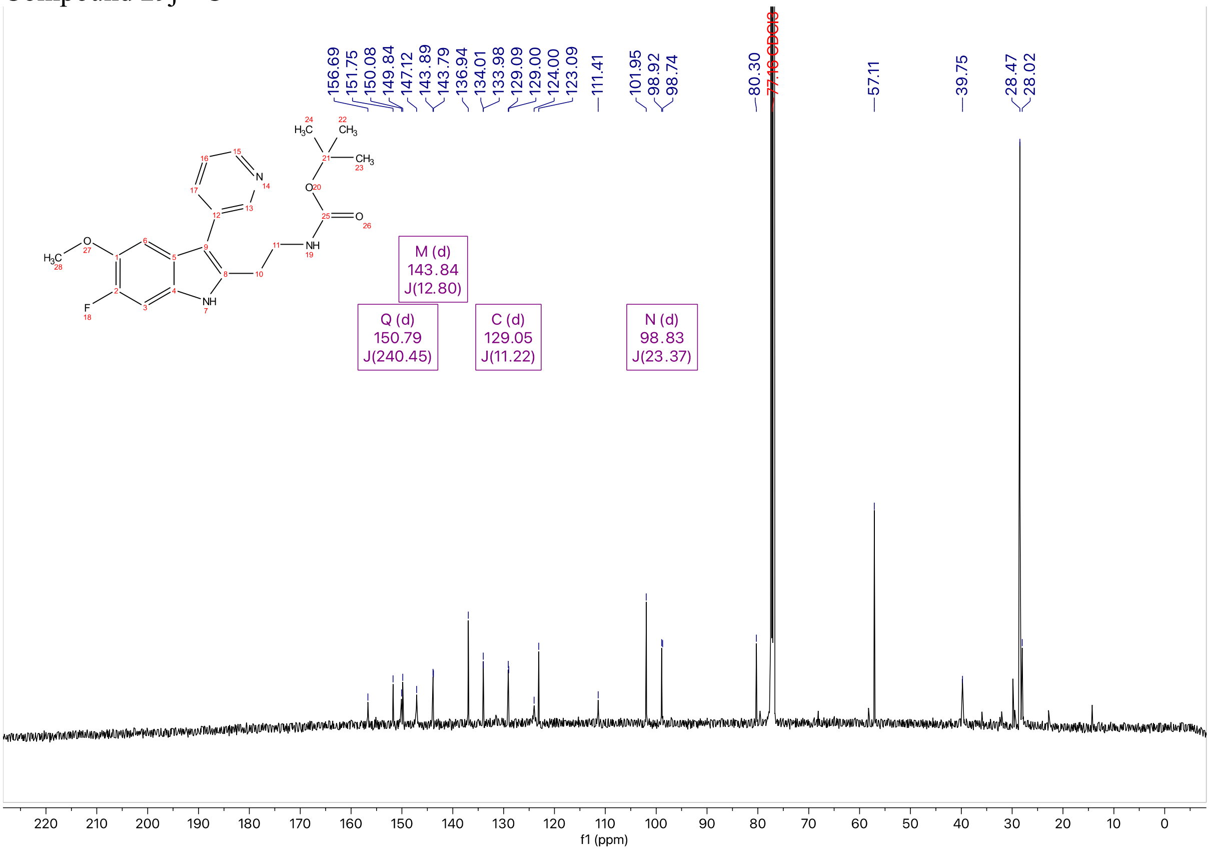

Compound 29j ^{19}F

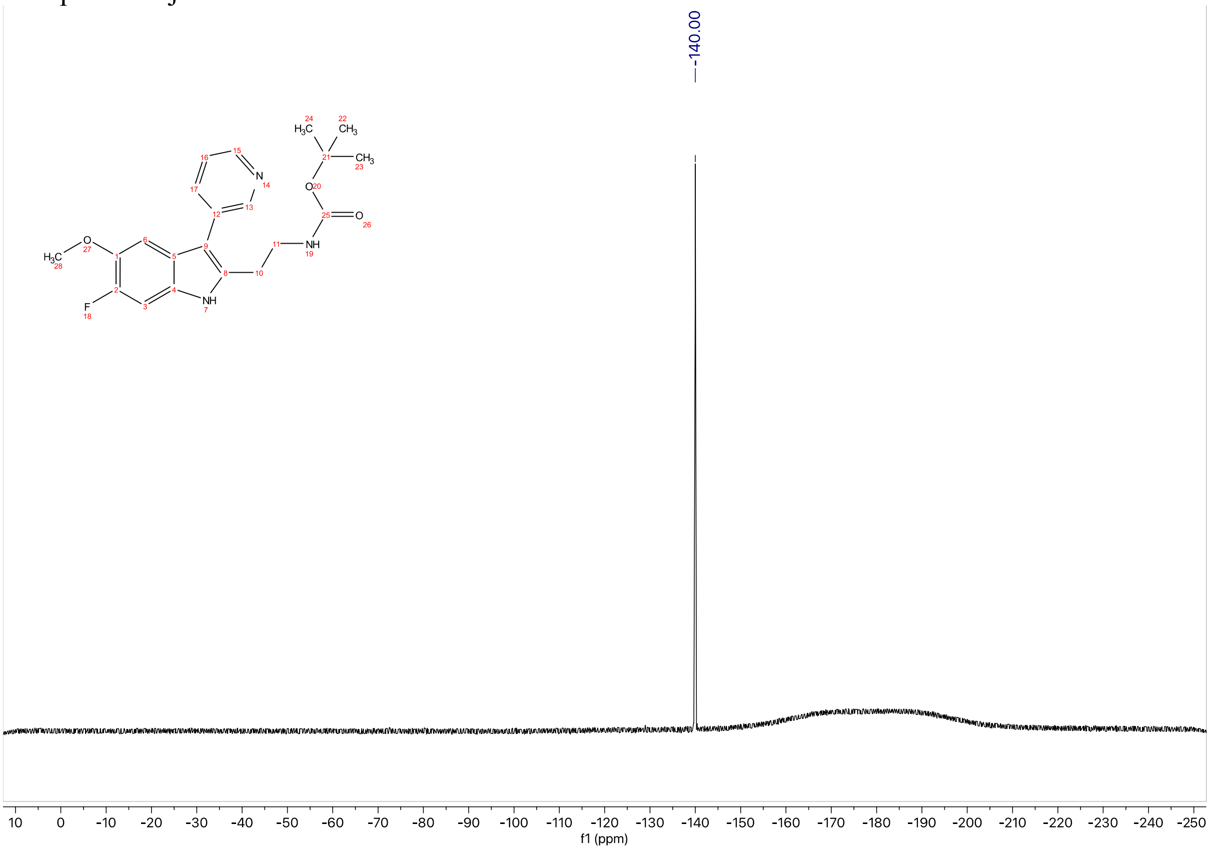

Compound 29k ^{1}H

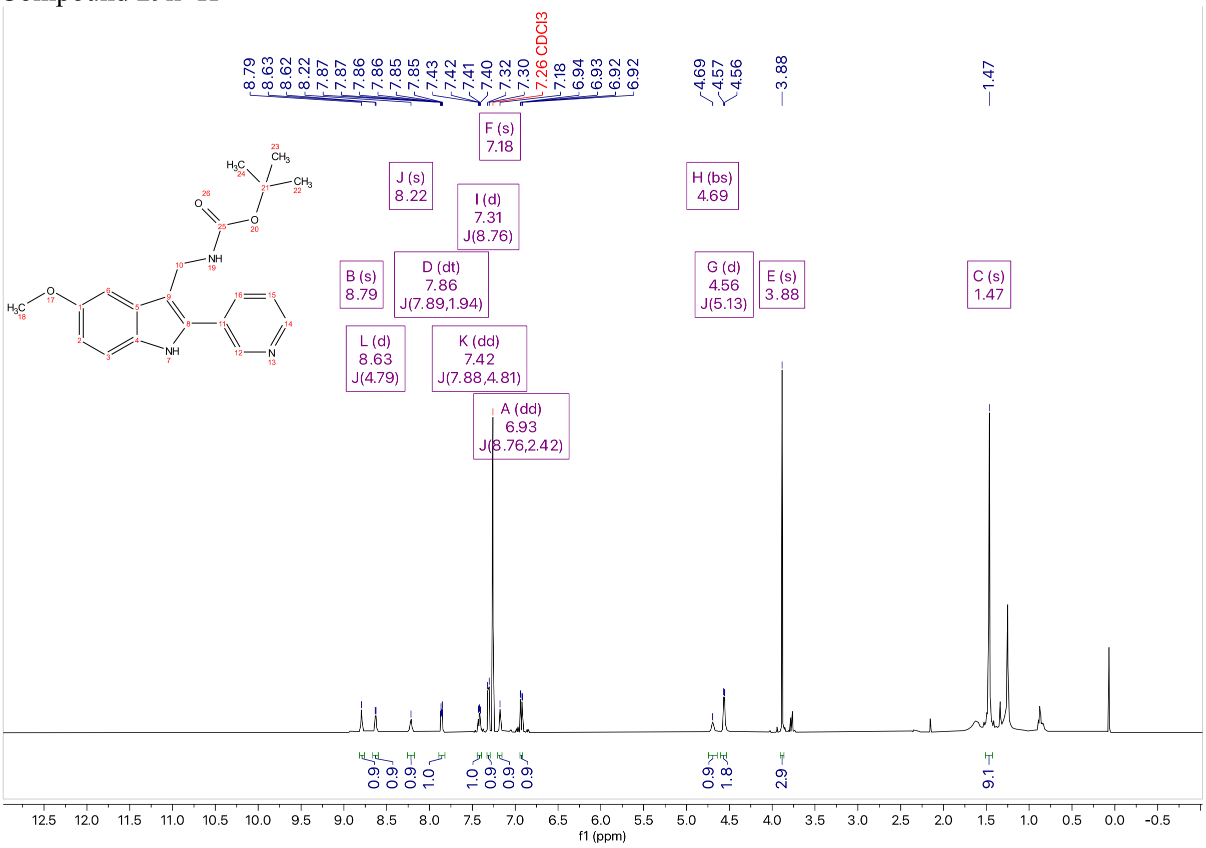

Compound 29k^{13}C

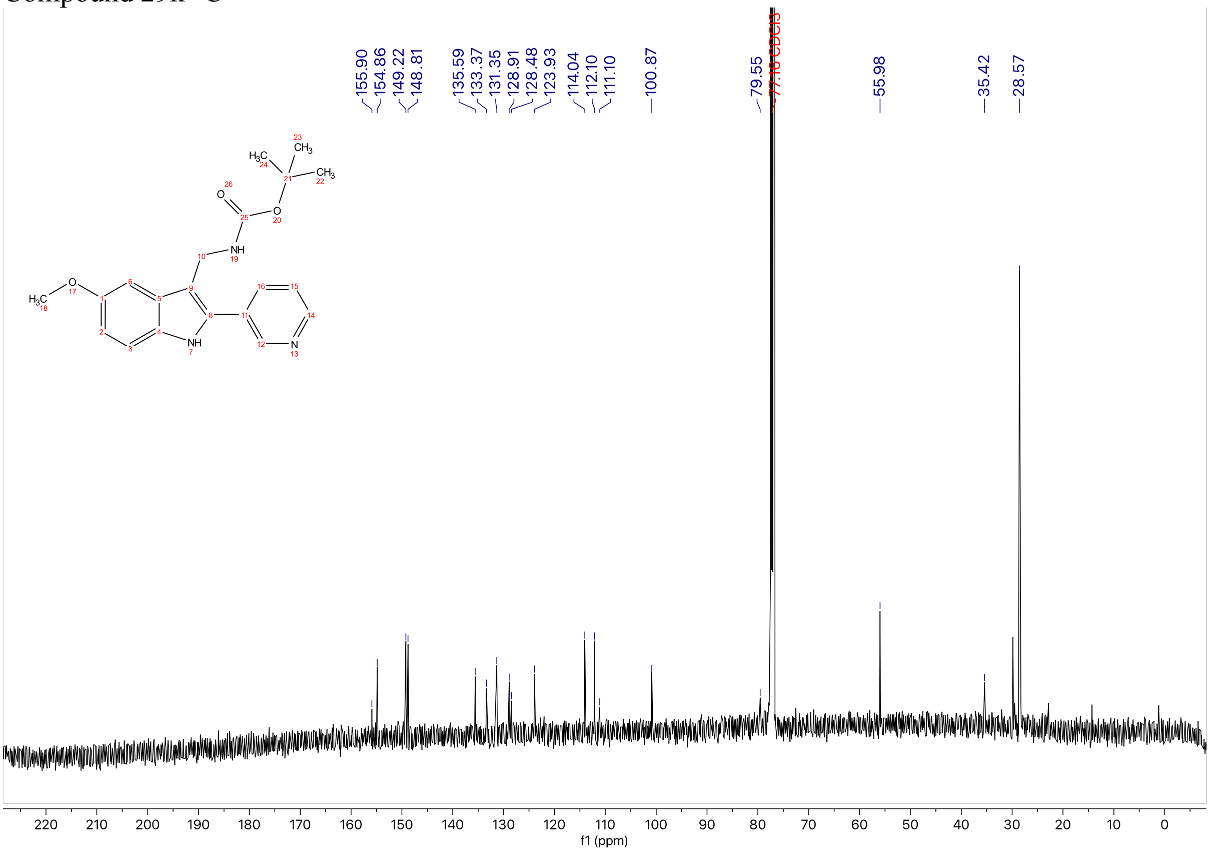

Compound 29l [1]H

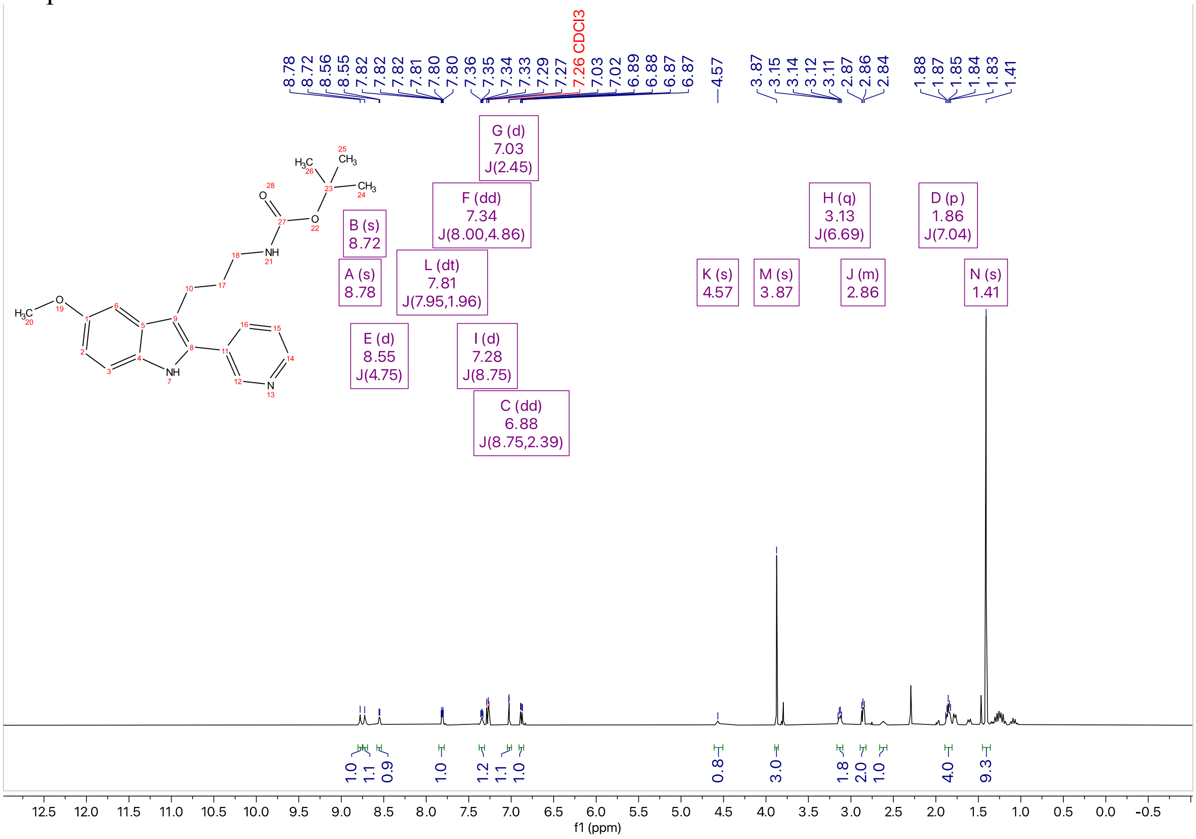

Compound 29l [13]C

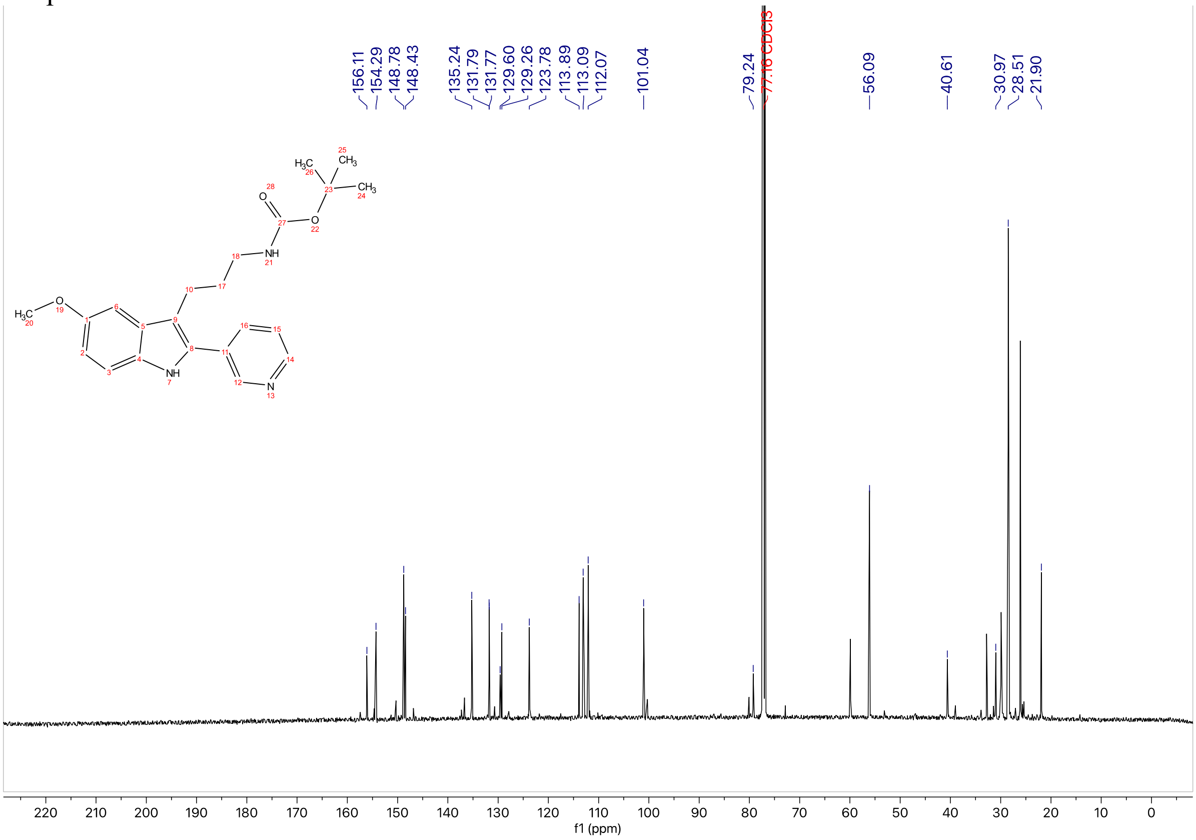

Compound 29m ^{1}H

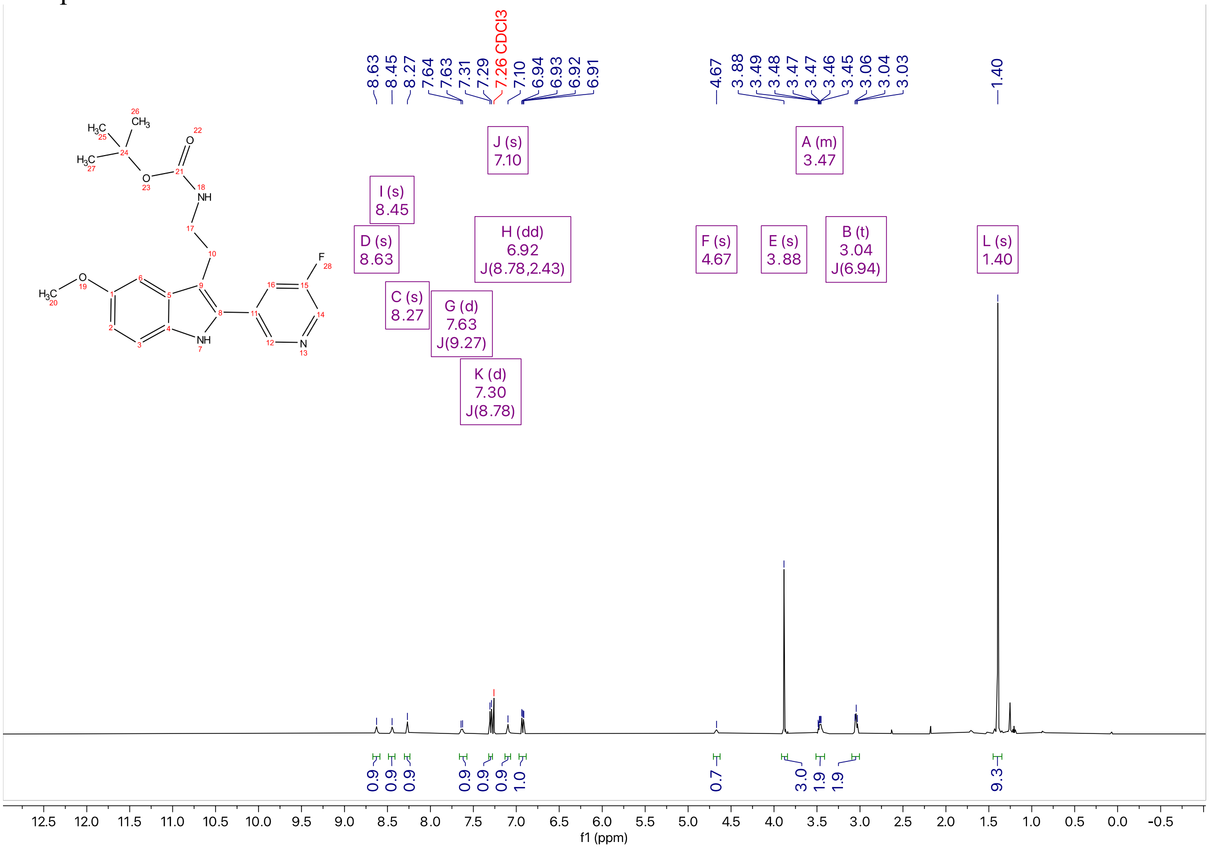

Compound 29m ^{13}C

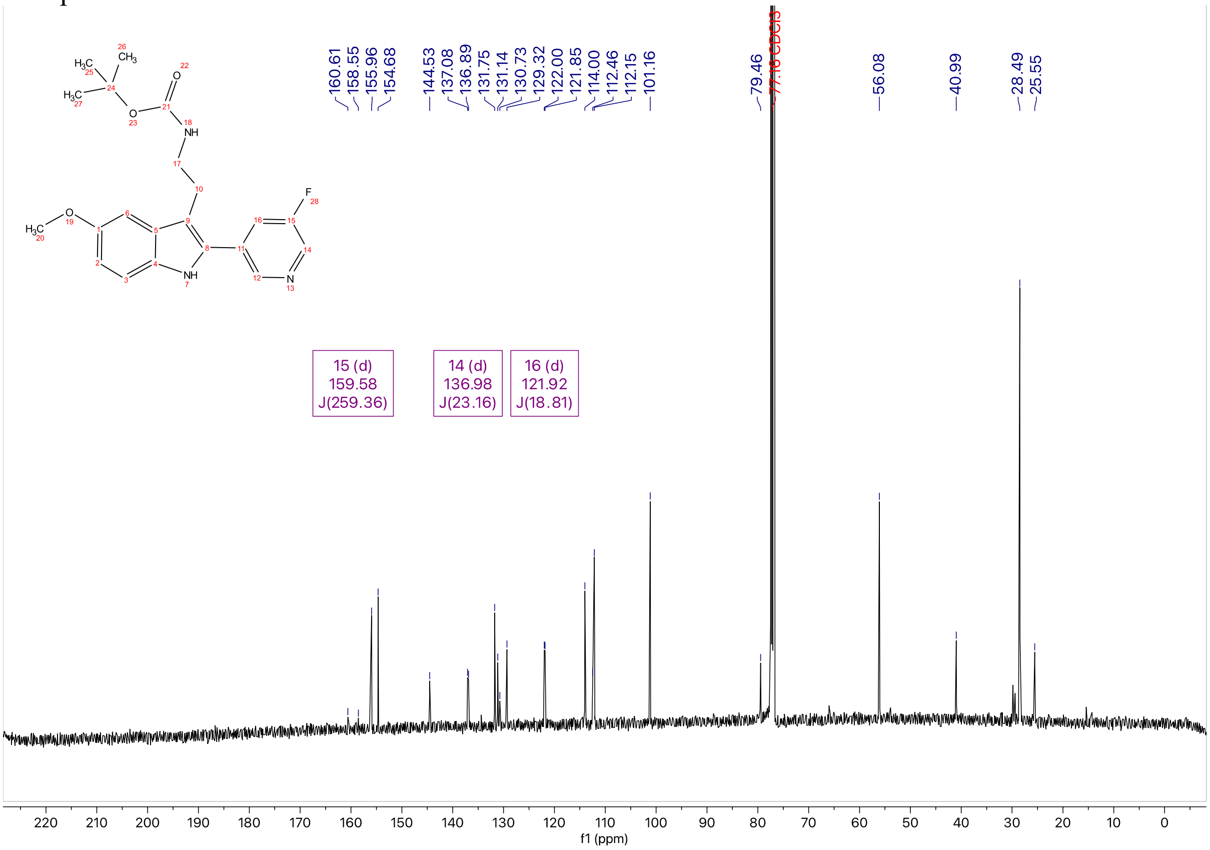

Compound 29m ^{19}F

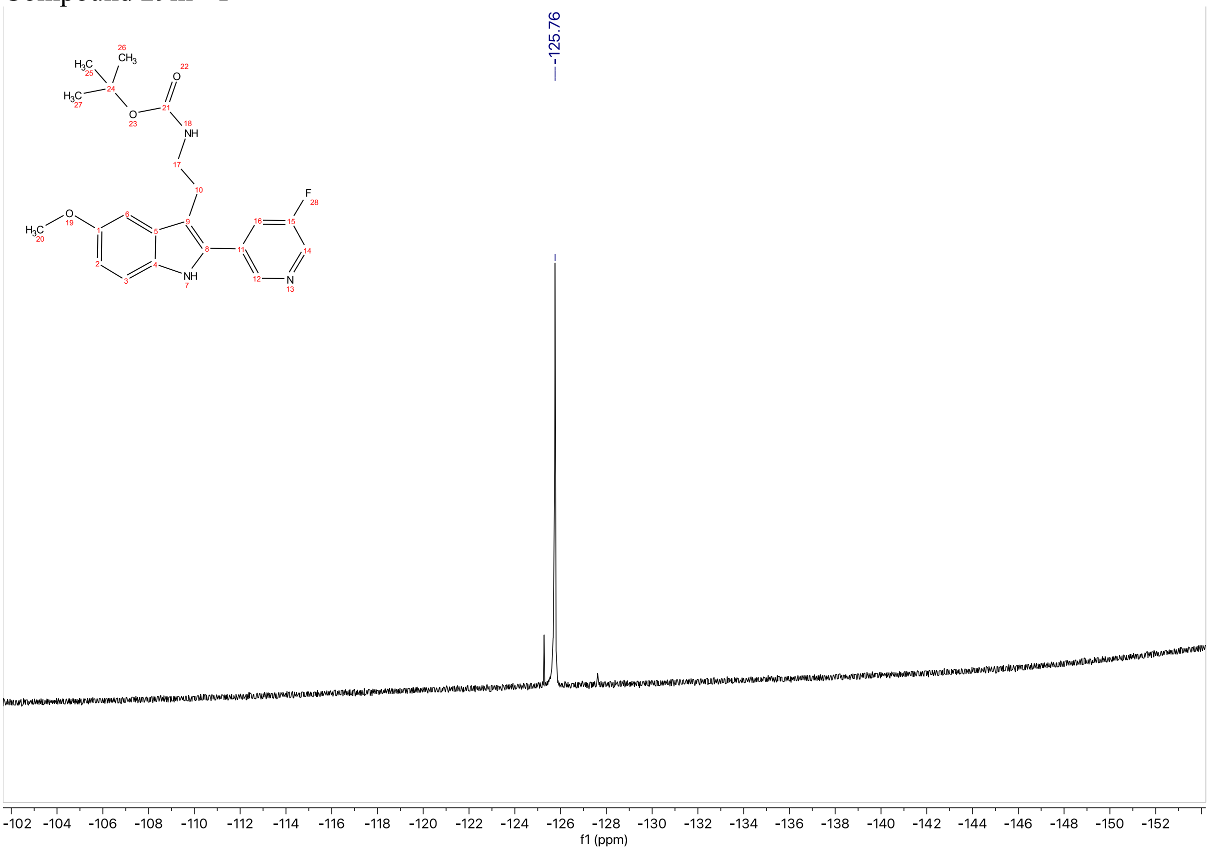

Compound 29n ^{1}H

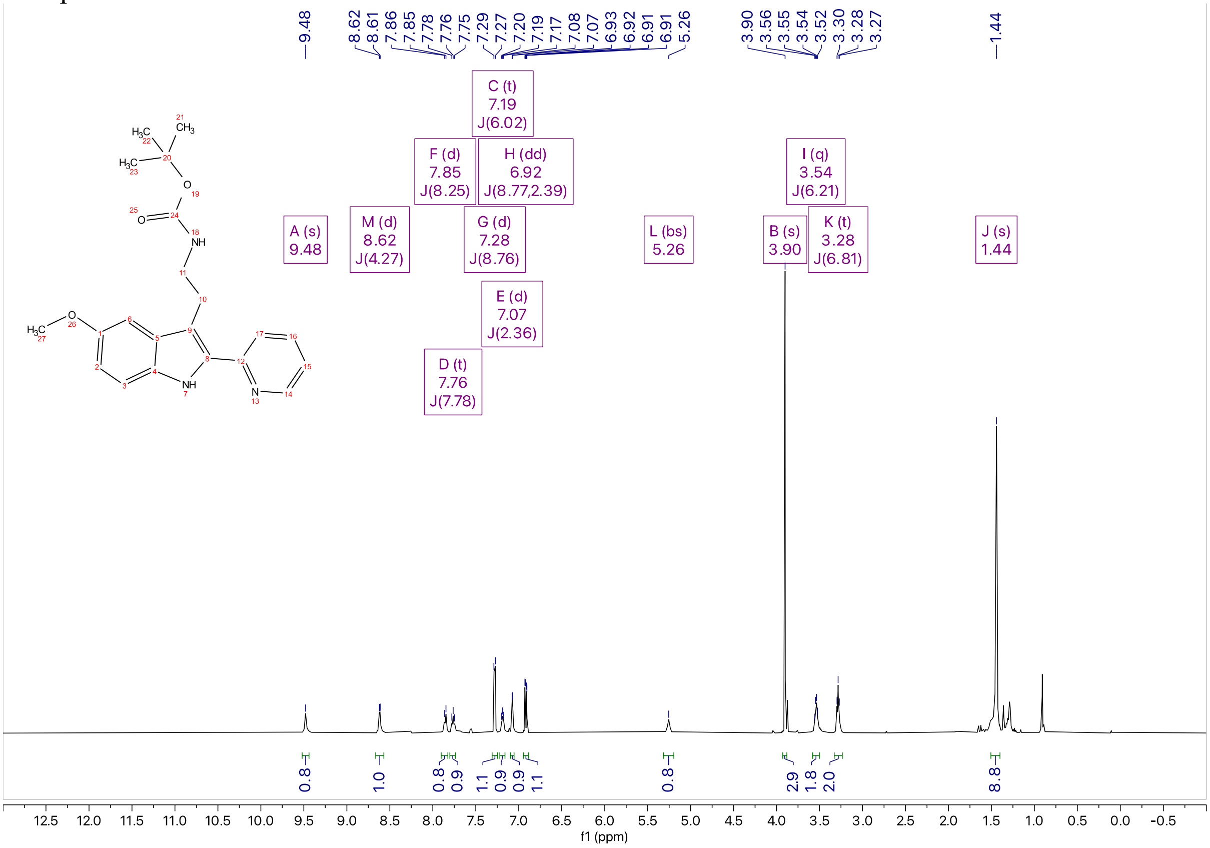

Compound 29n ^{13}C

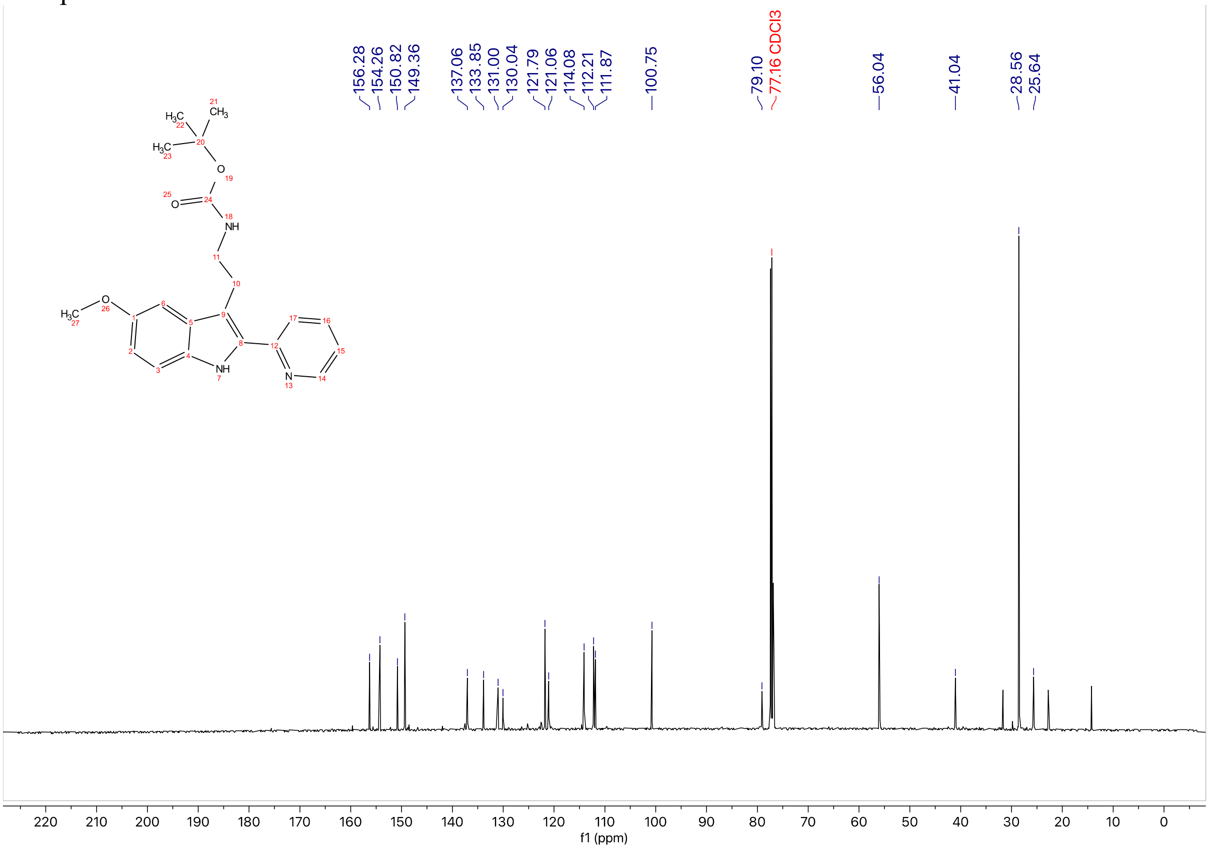

Compound 29o ^{1}H

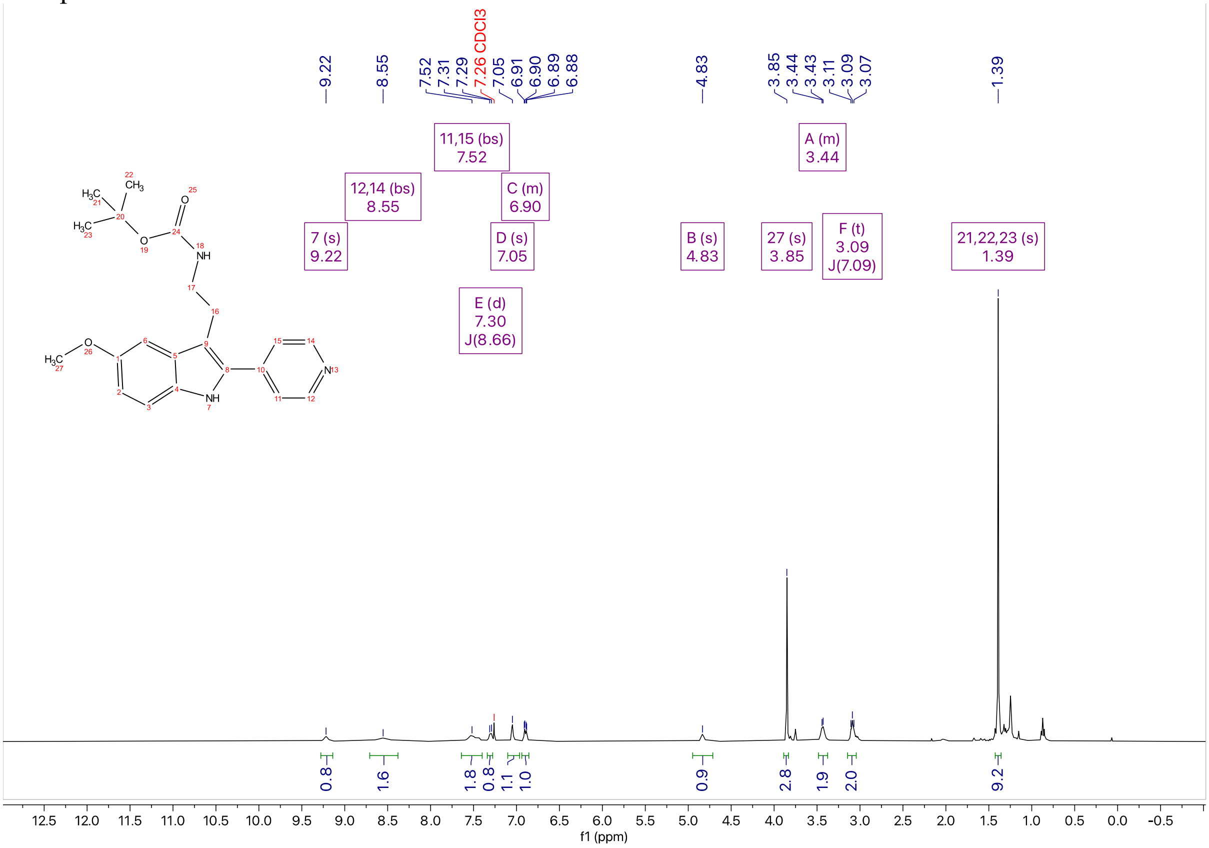

Compound 29o ^{13}C

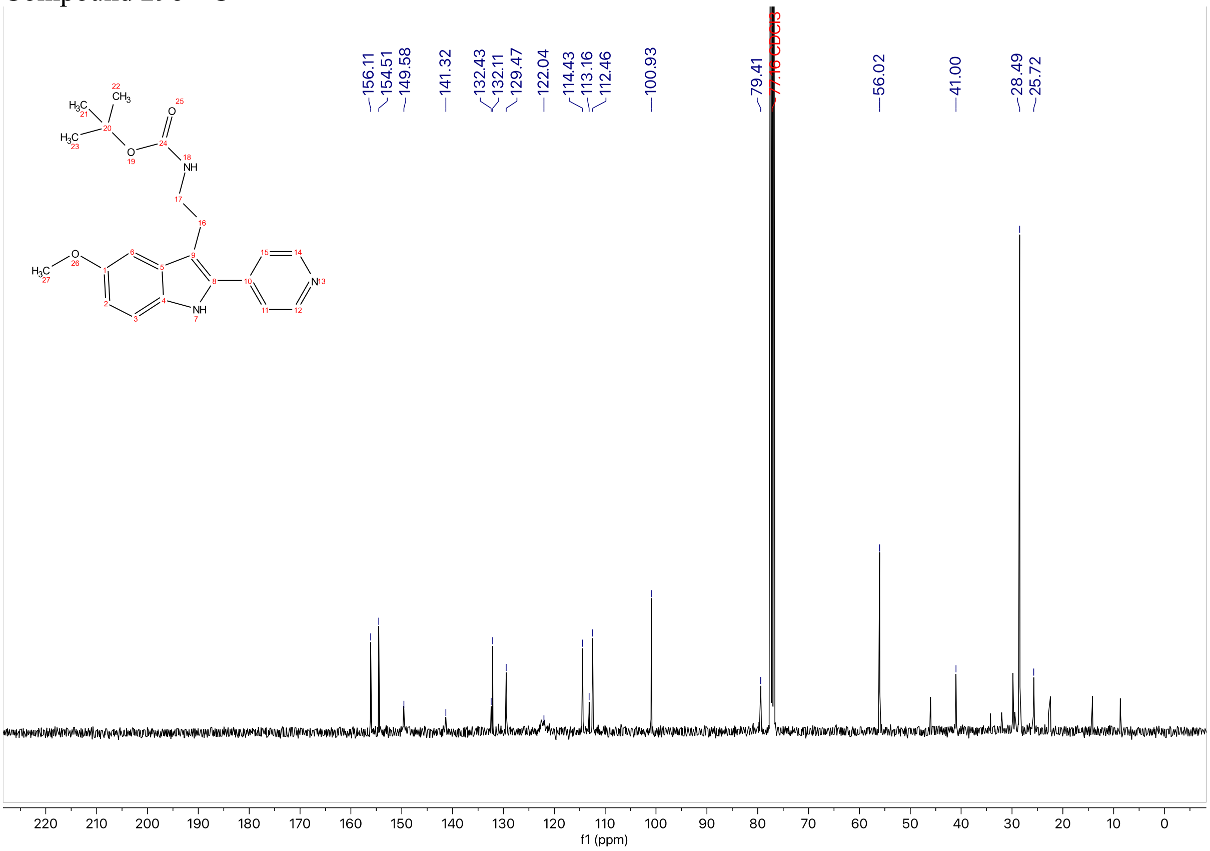

Compound 29p ^{1}H

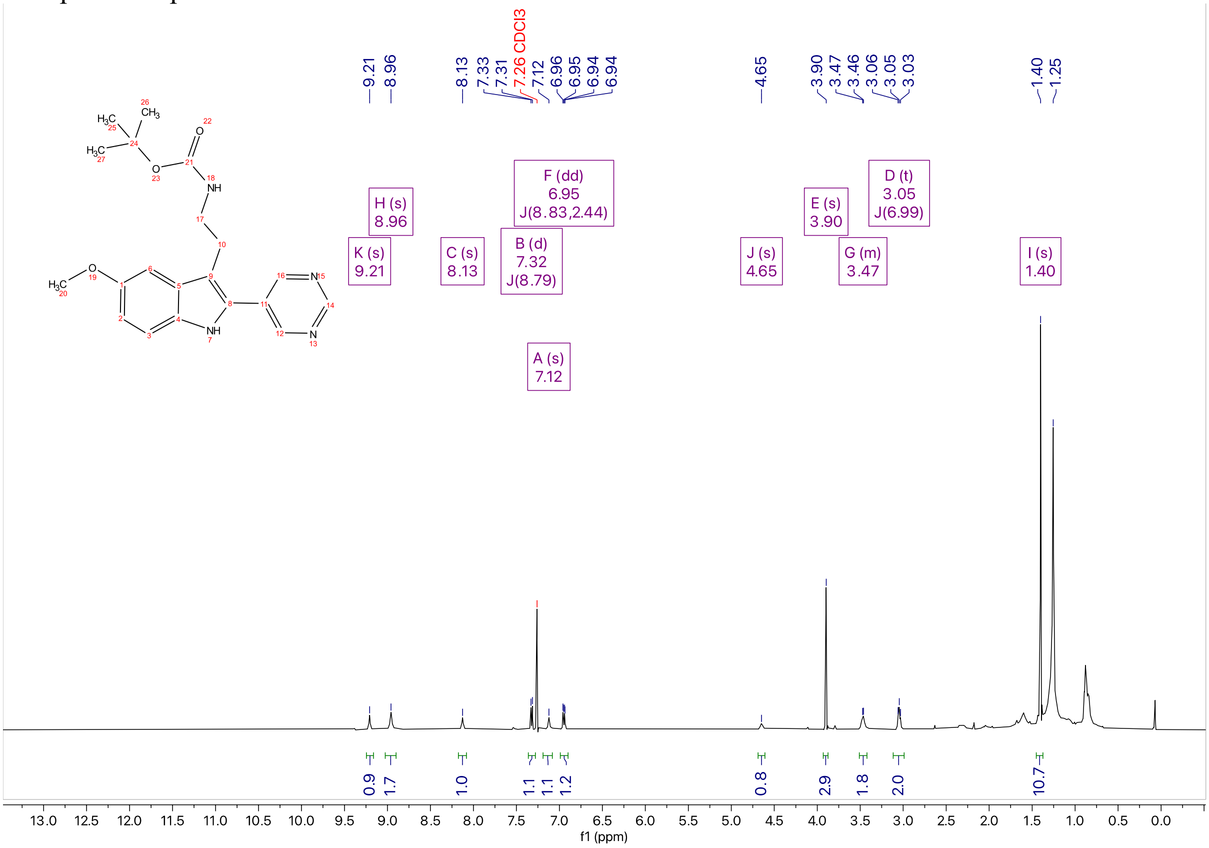

Compound 29p ^{13}C

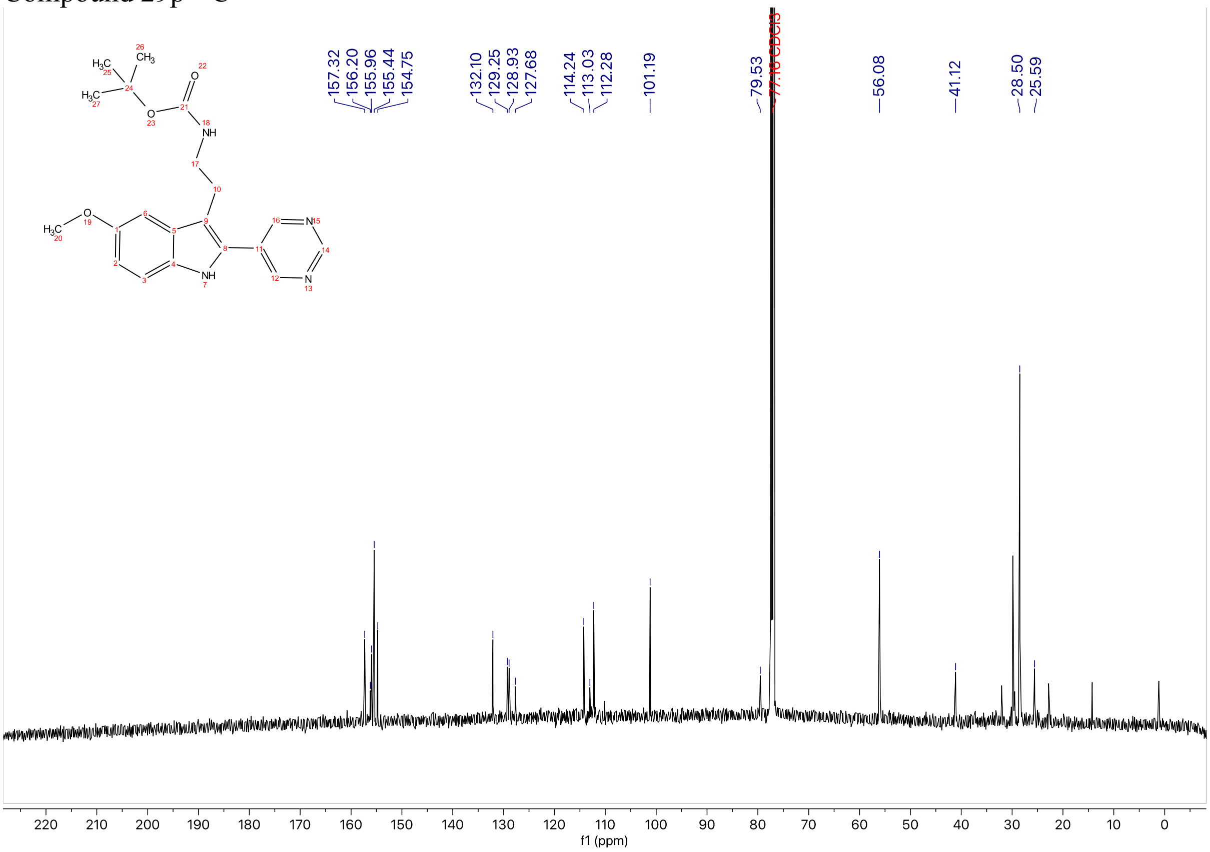

Compound 29q ^{1}H

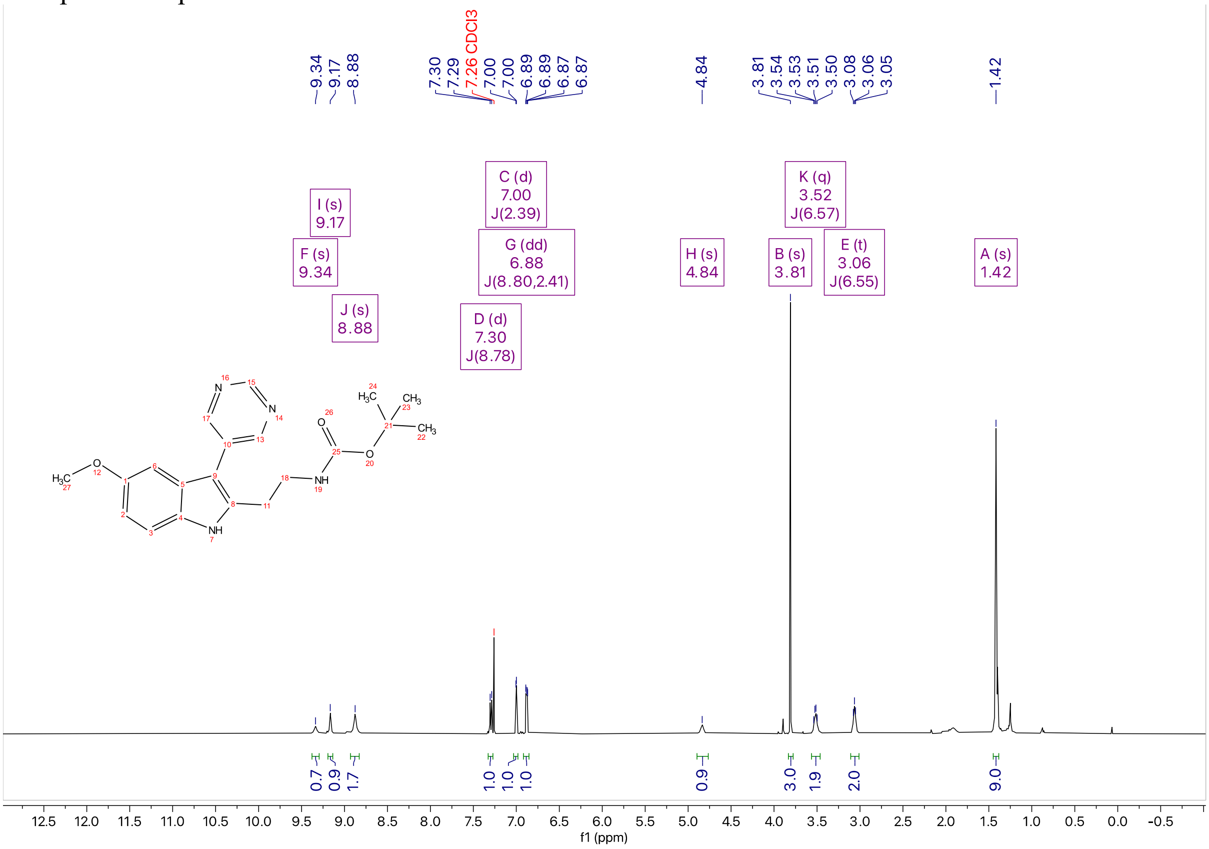

Compound 29q ^{13}C

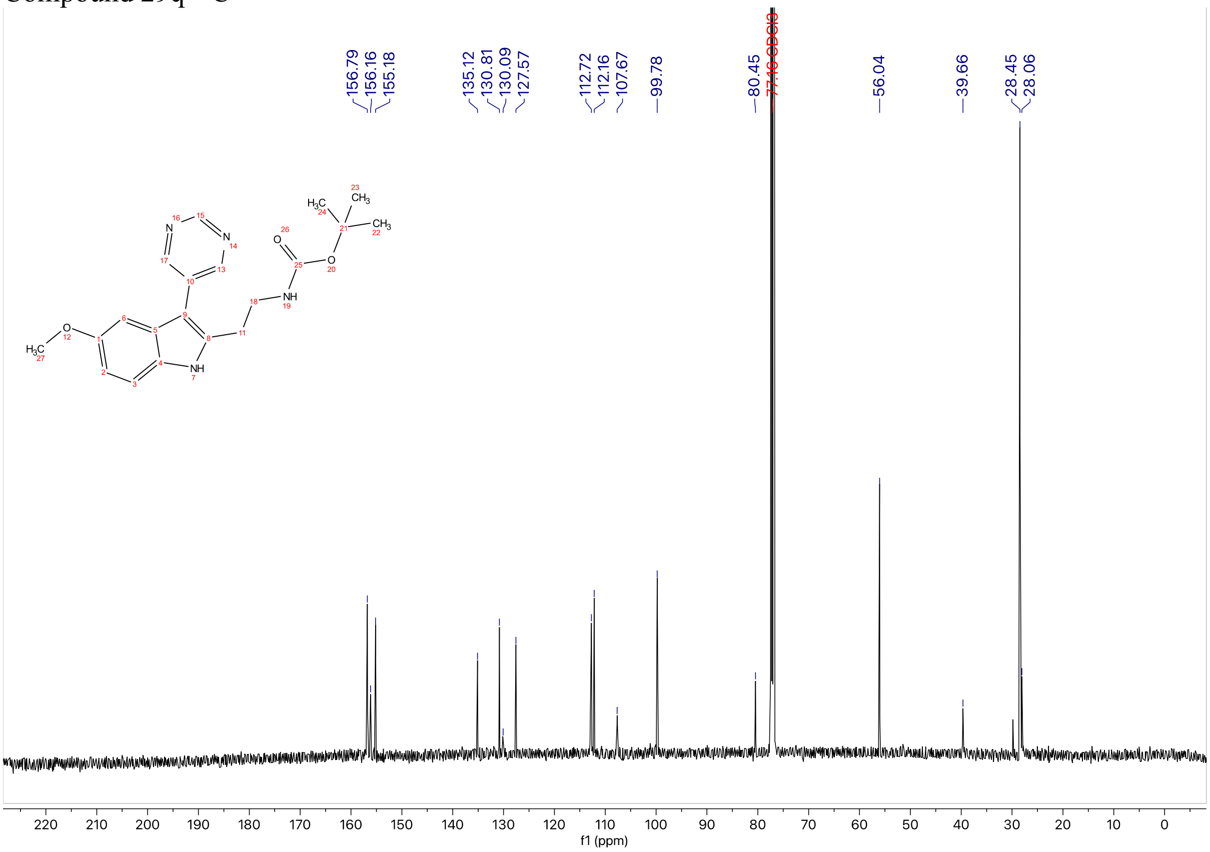

Compound 29r ^{1}H

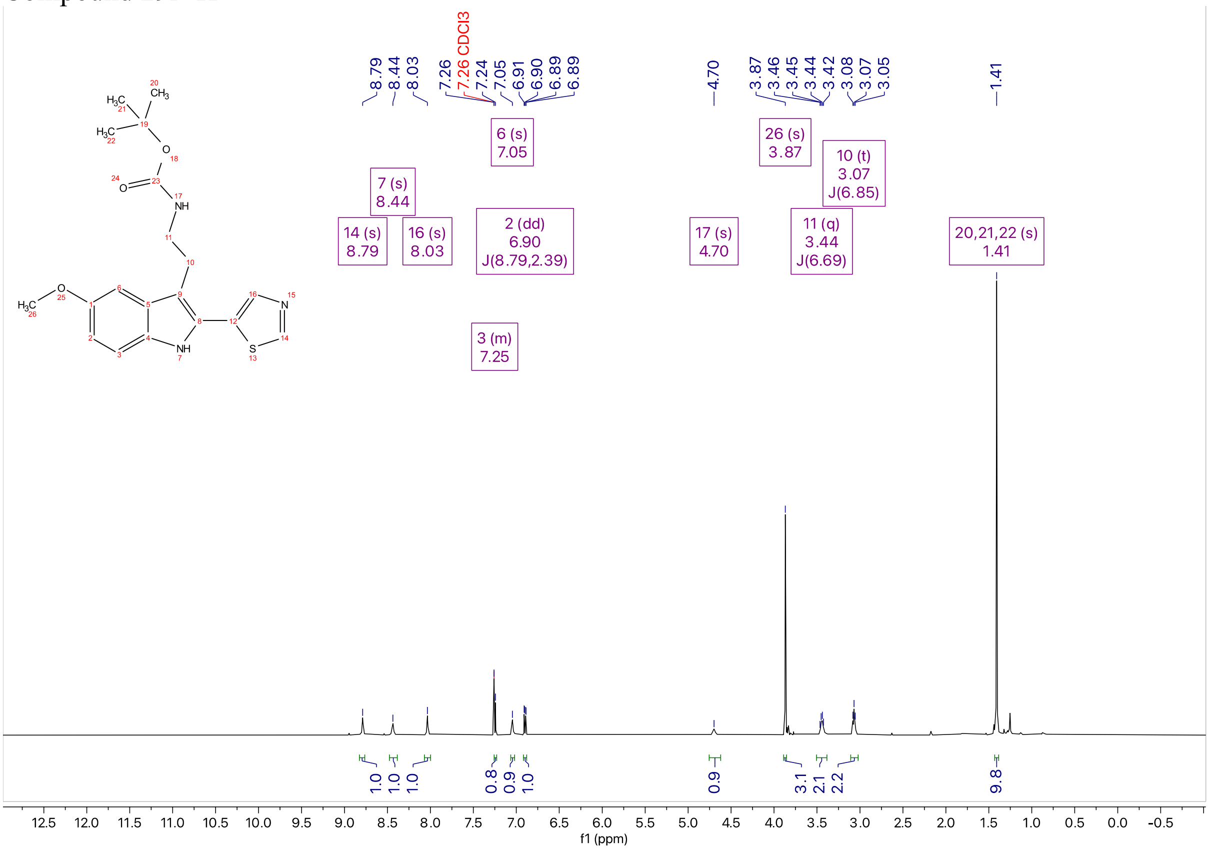

Compound 29r ^{13}C

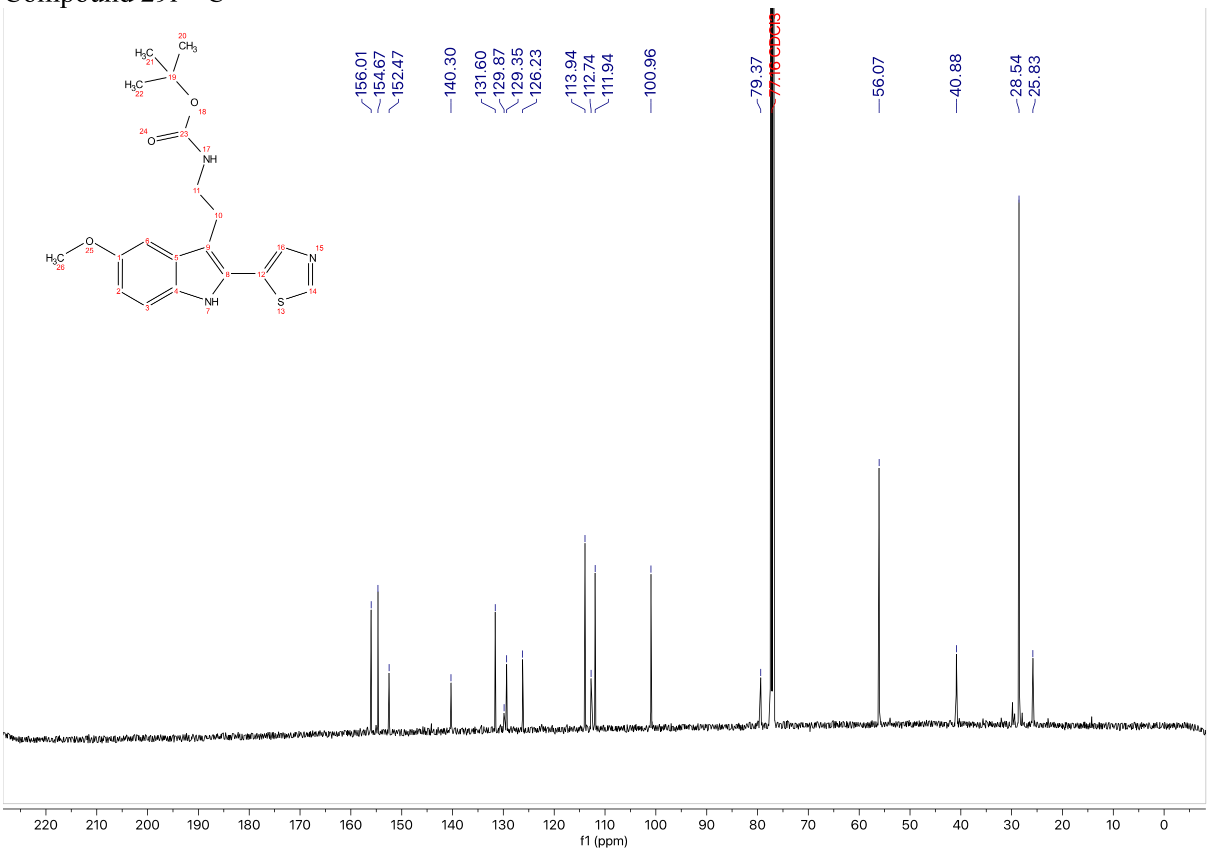

Compound 29s ^{1}H

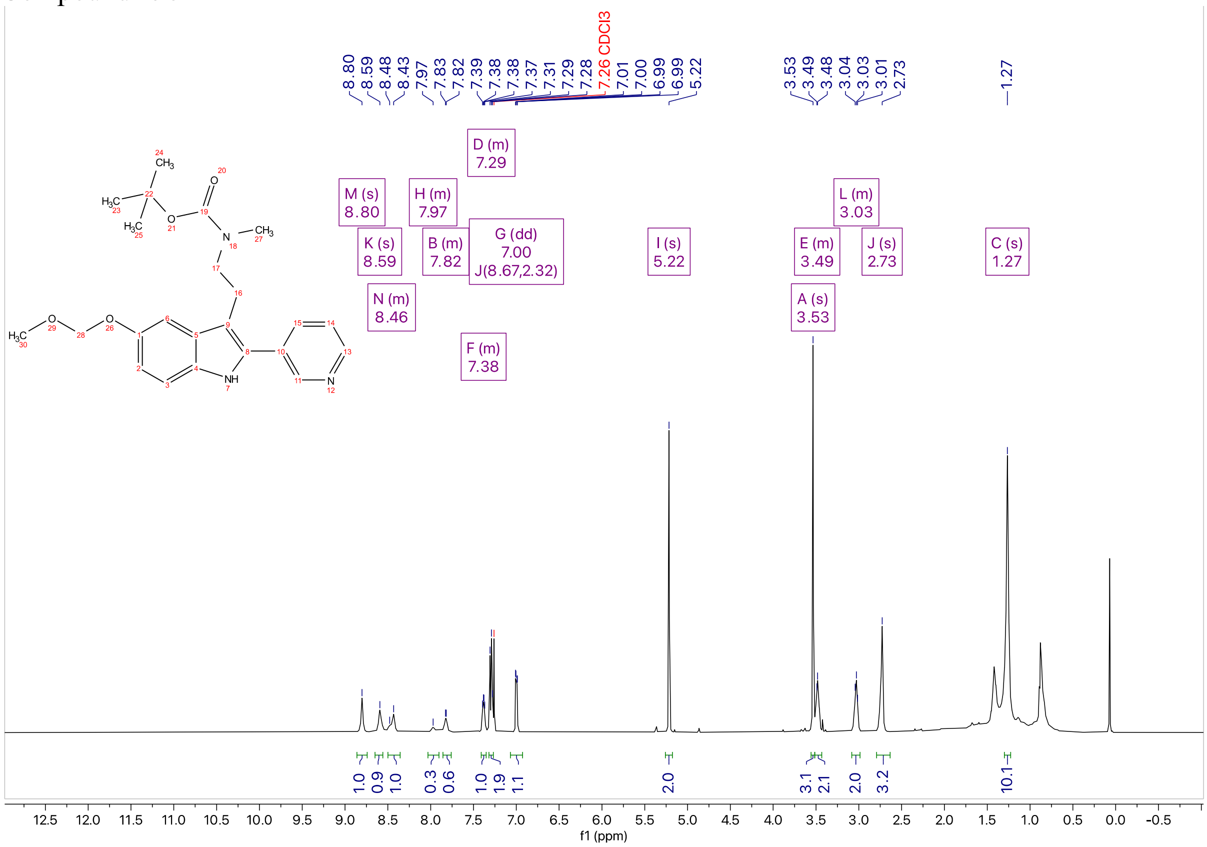

Compound 29s ^{13}C

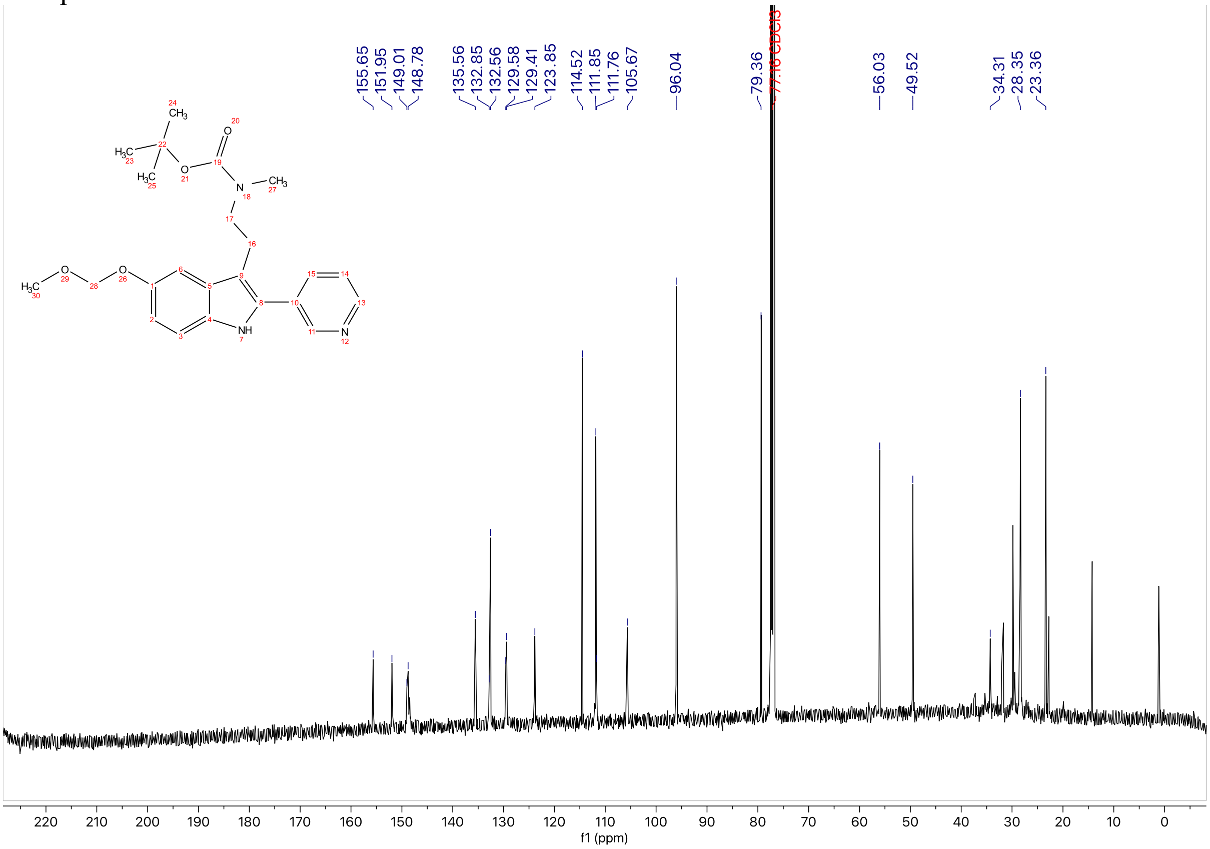

Compound 29t ^{1}H

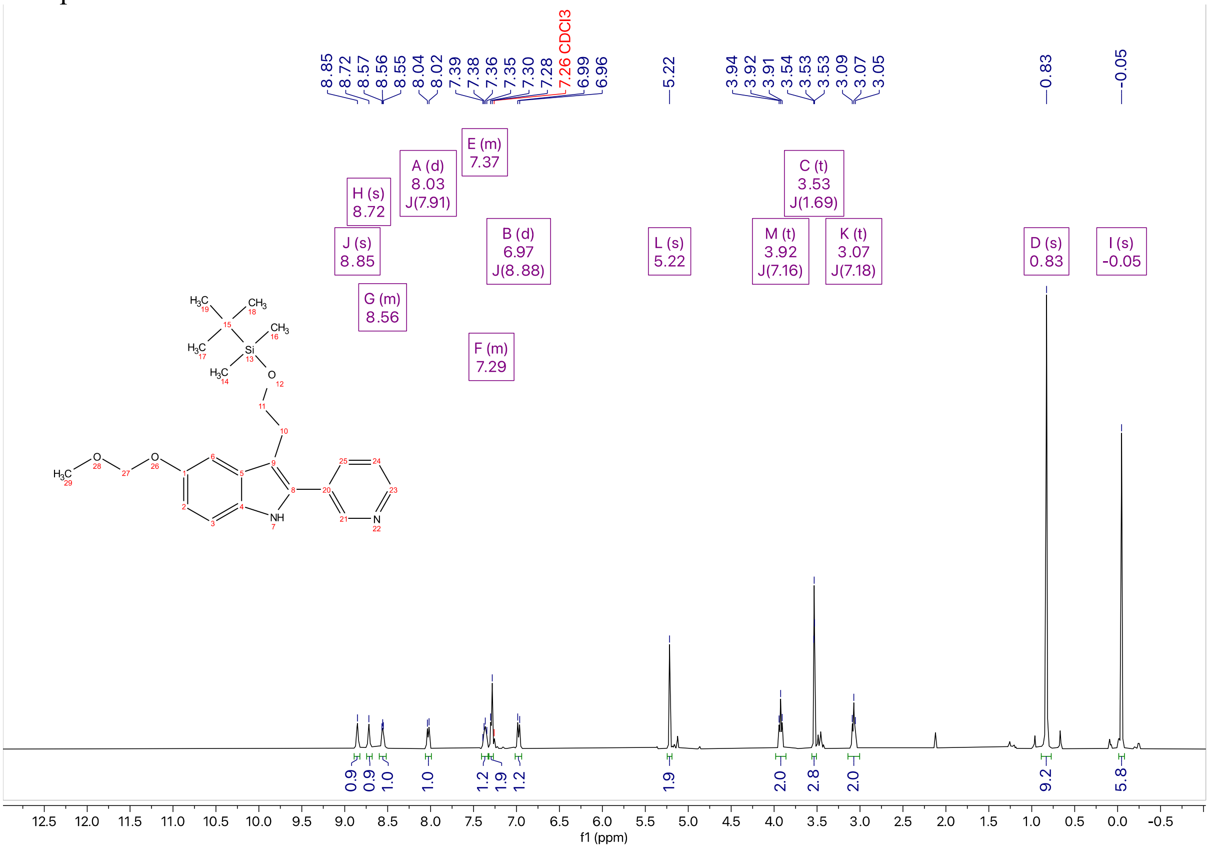

Compound 29t ^{13}C

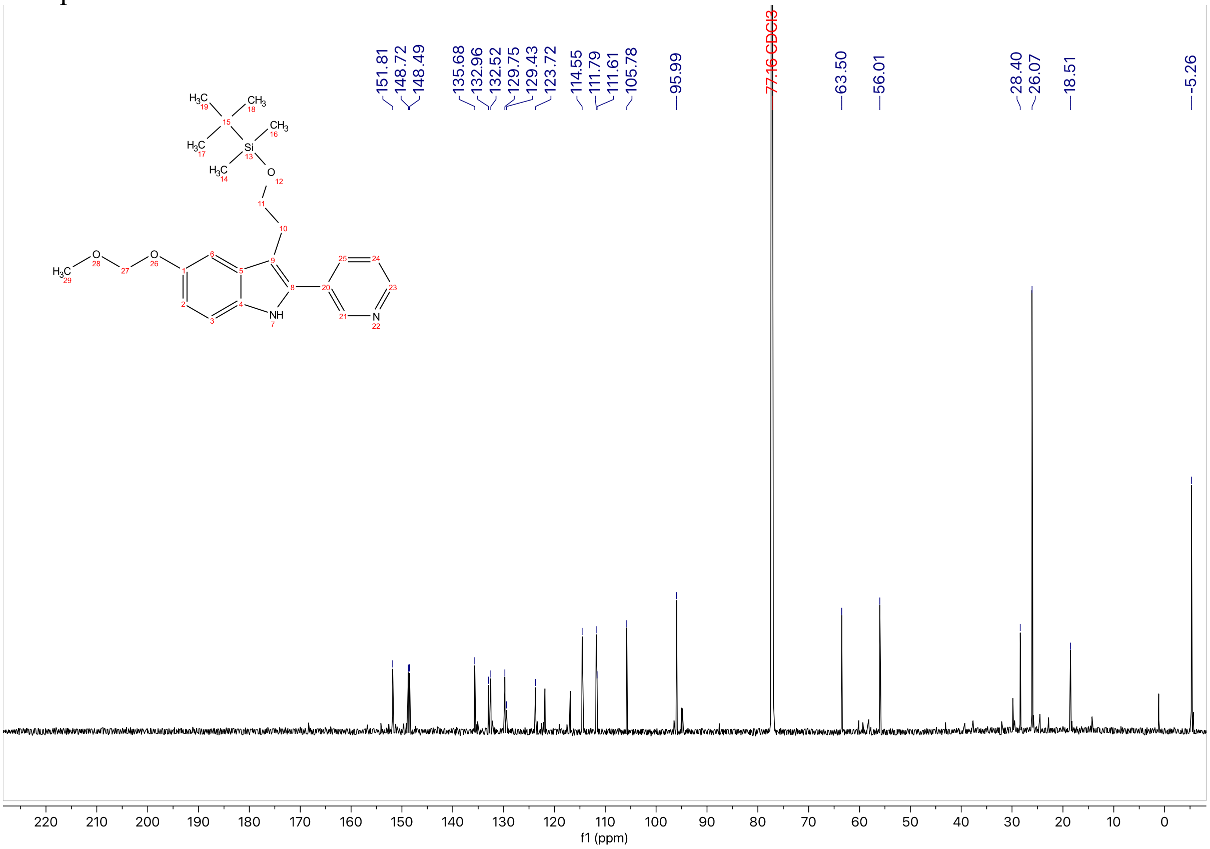

Compound 29u ^{1}H

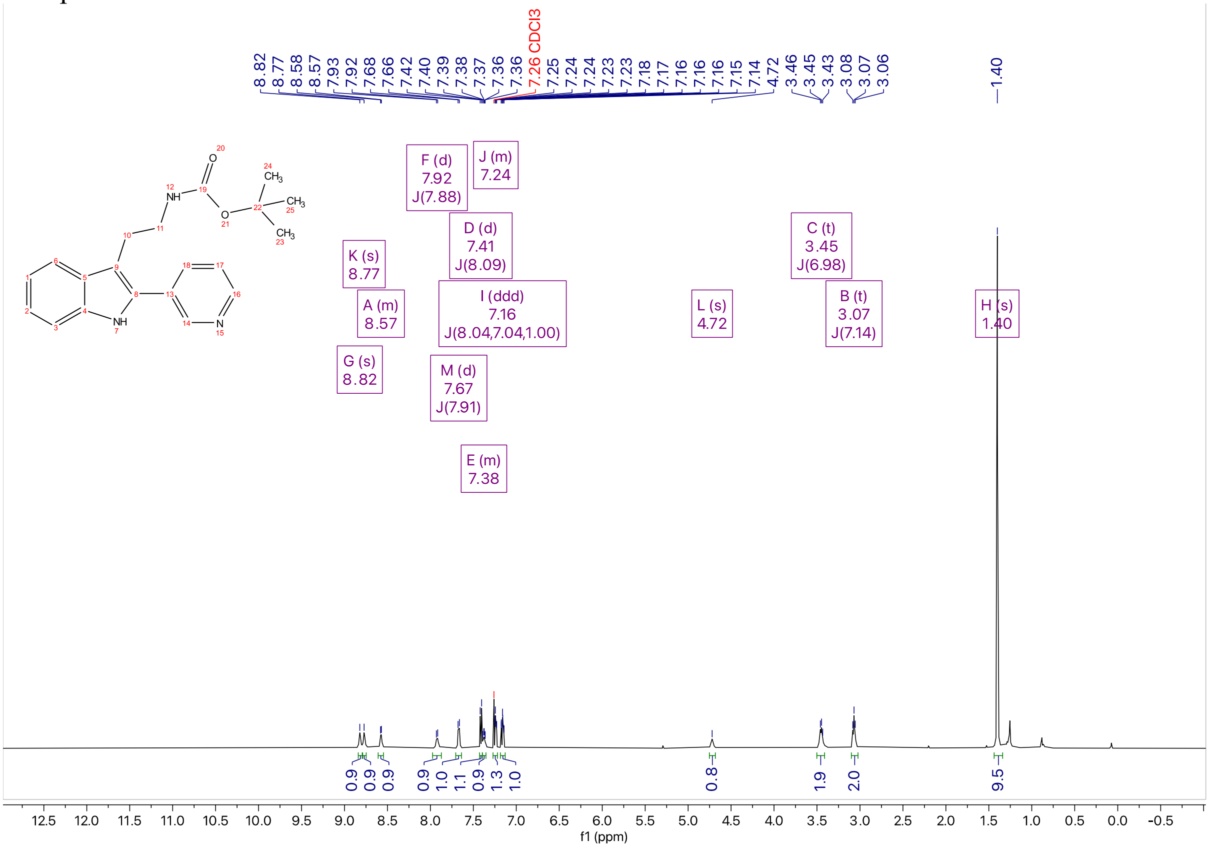

Compound 29u ^{13}C

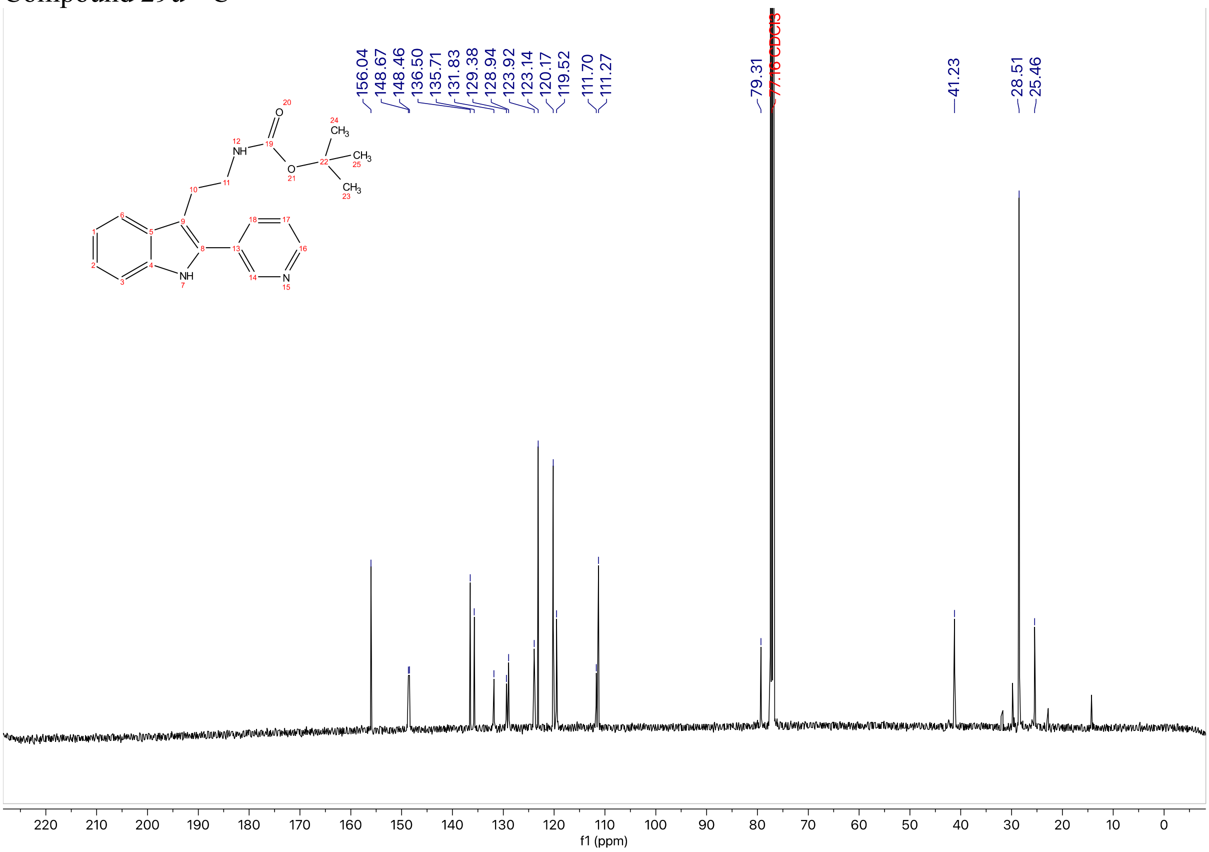

Compound 29v ^{1}H

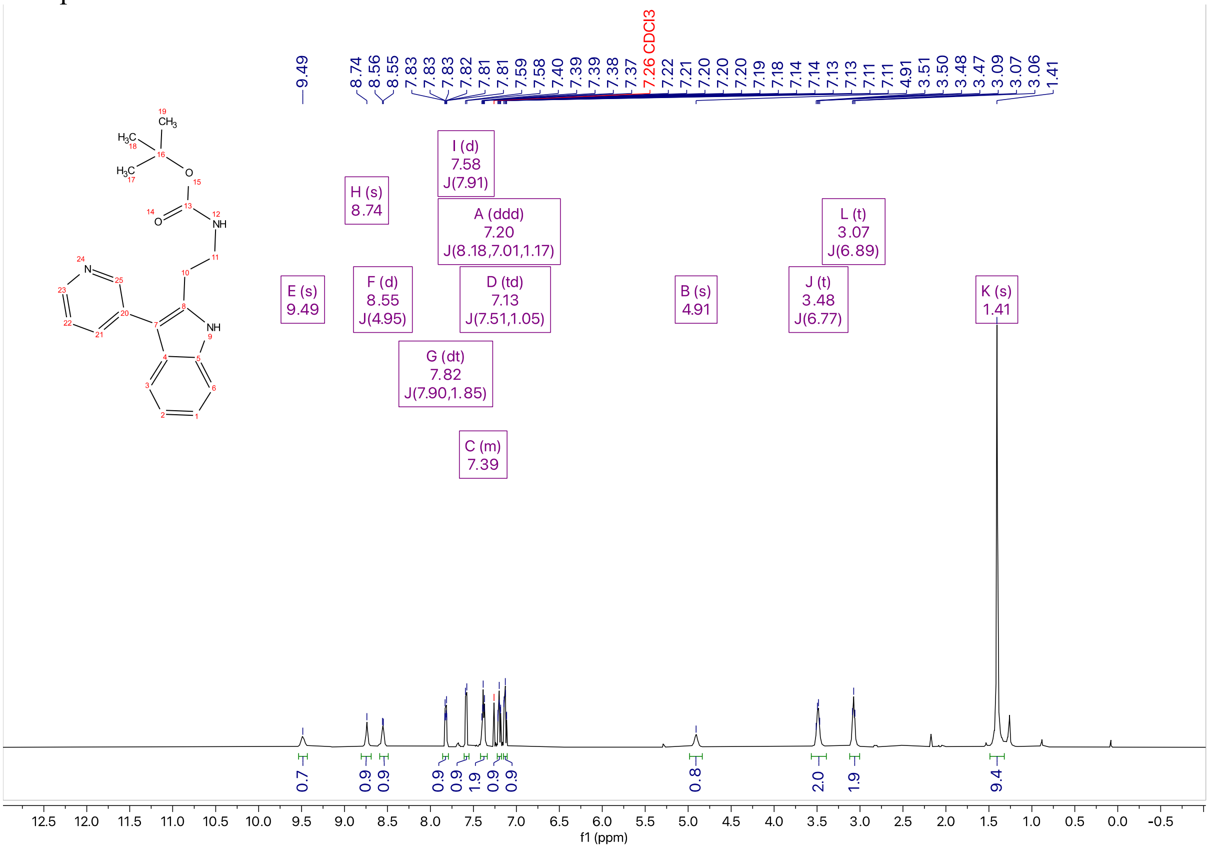

Compound 29v ^{13}C

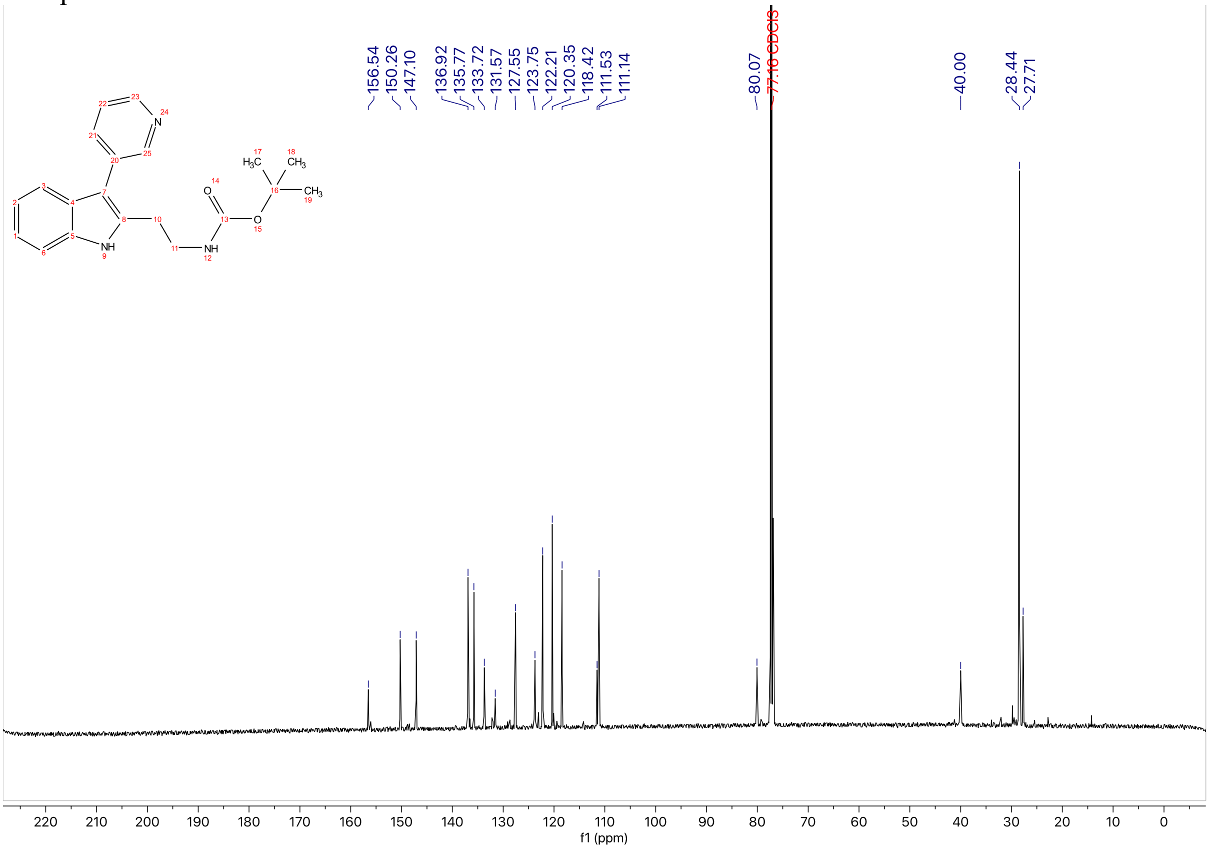

Compound S3 ^{1}H

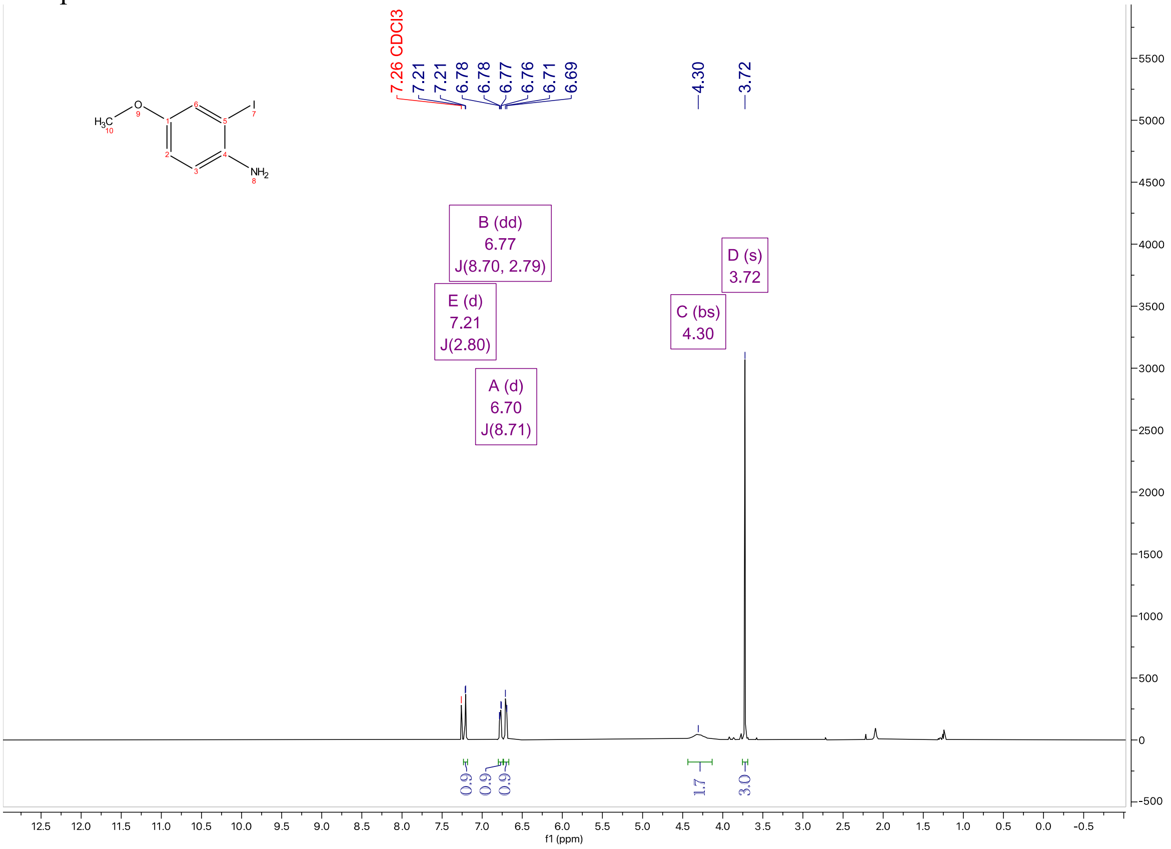

Compound S3 ^{13}C

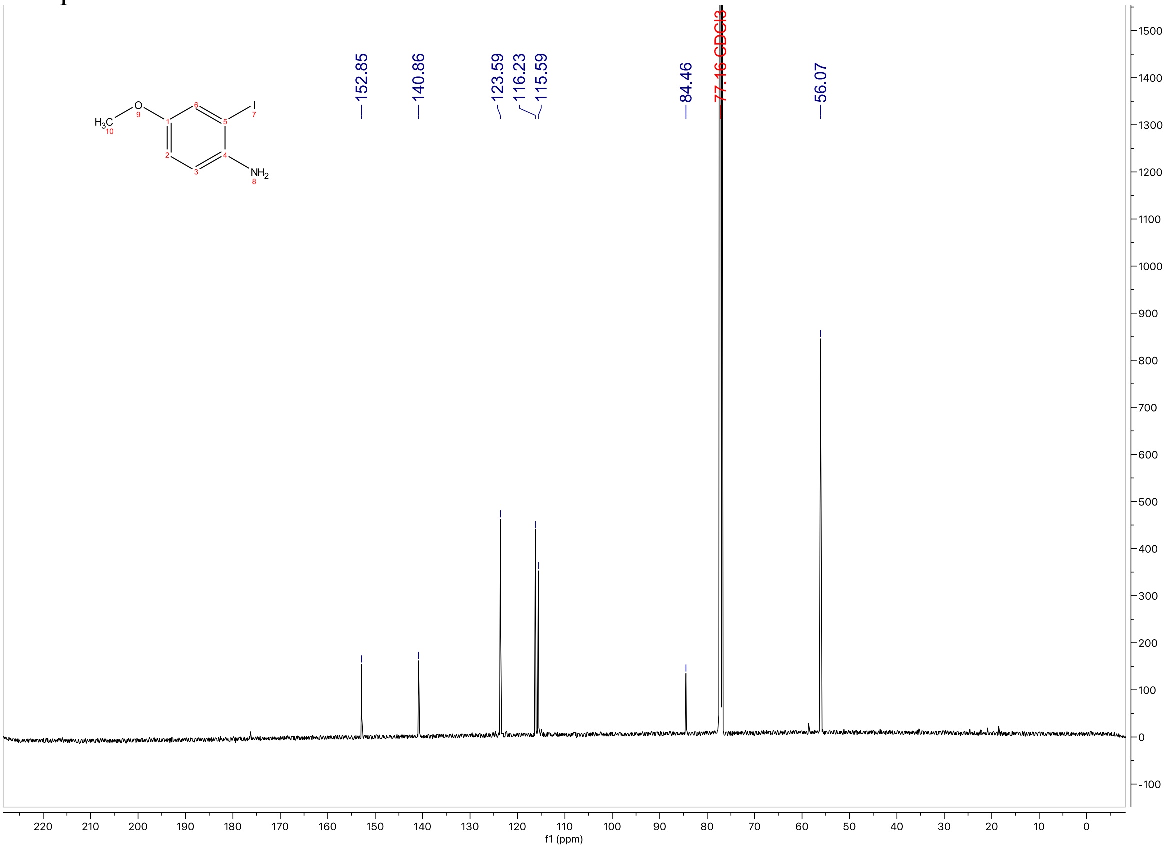

Compound S1a ^{1}H

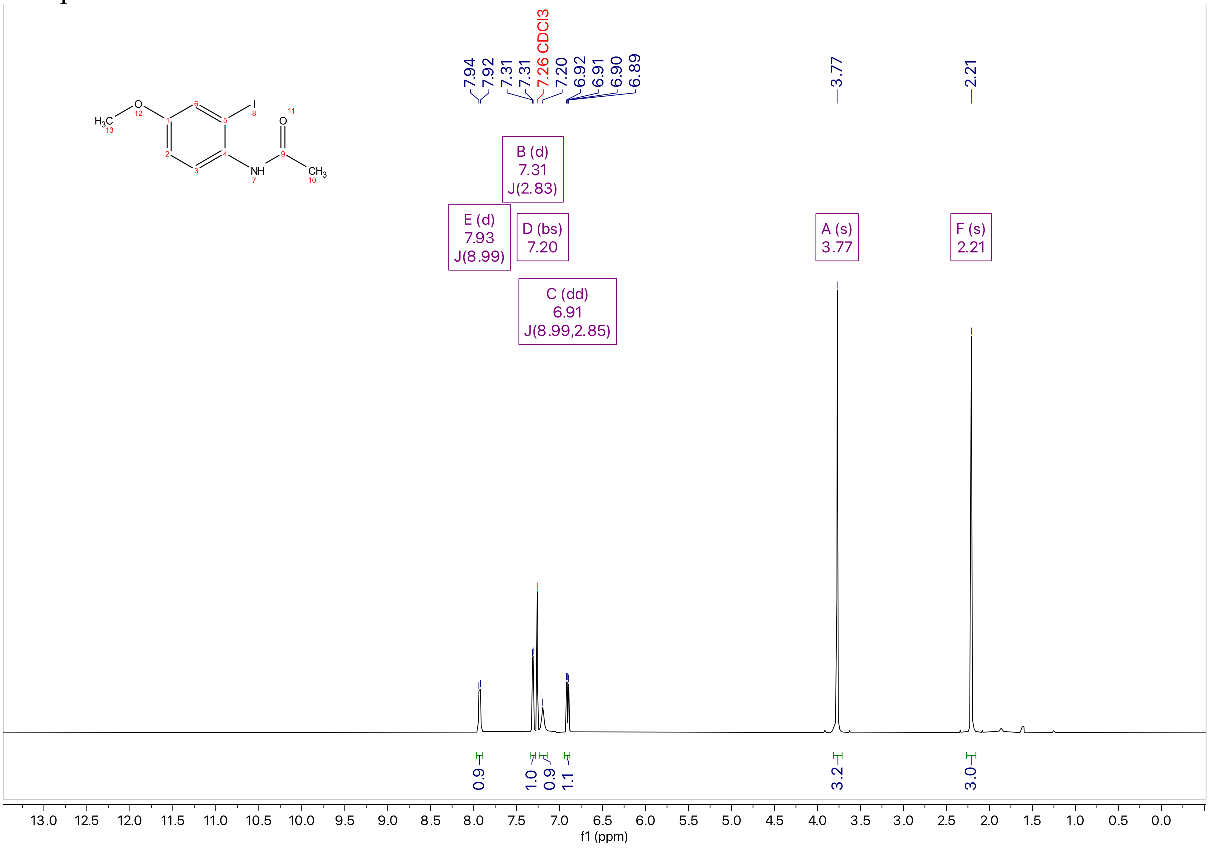

Compound S1a ^{13}C

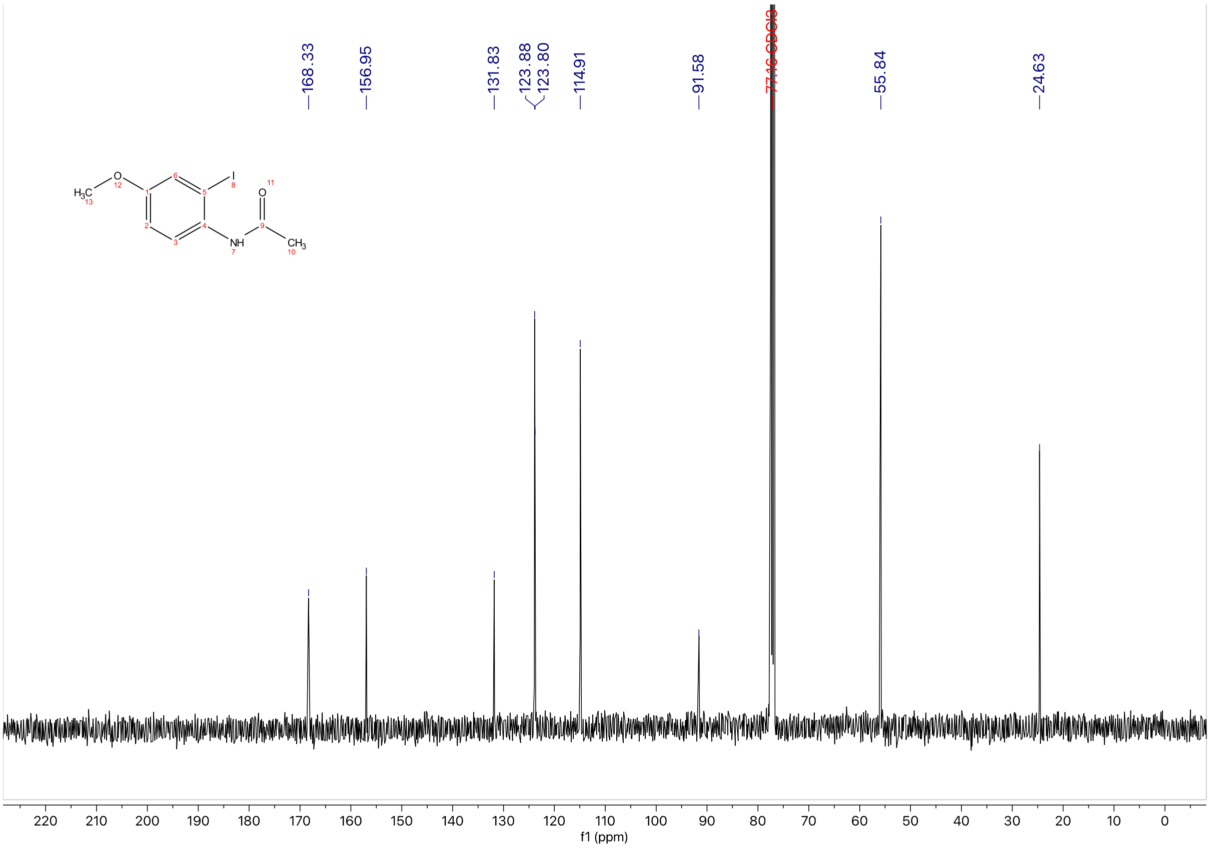

Compound S2a ^{1}H

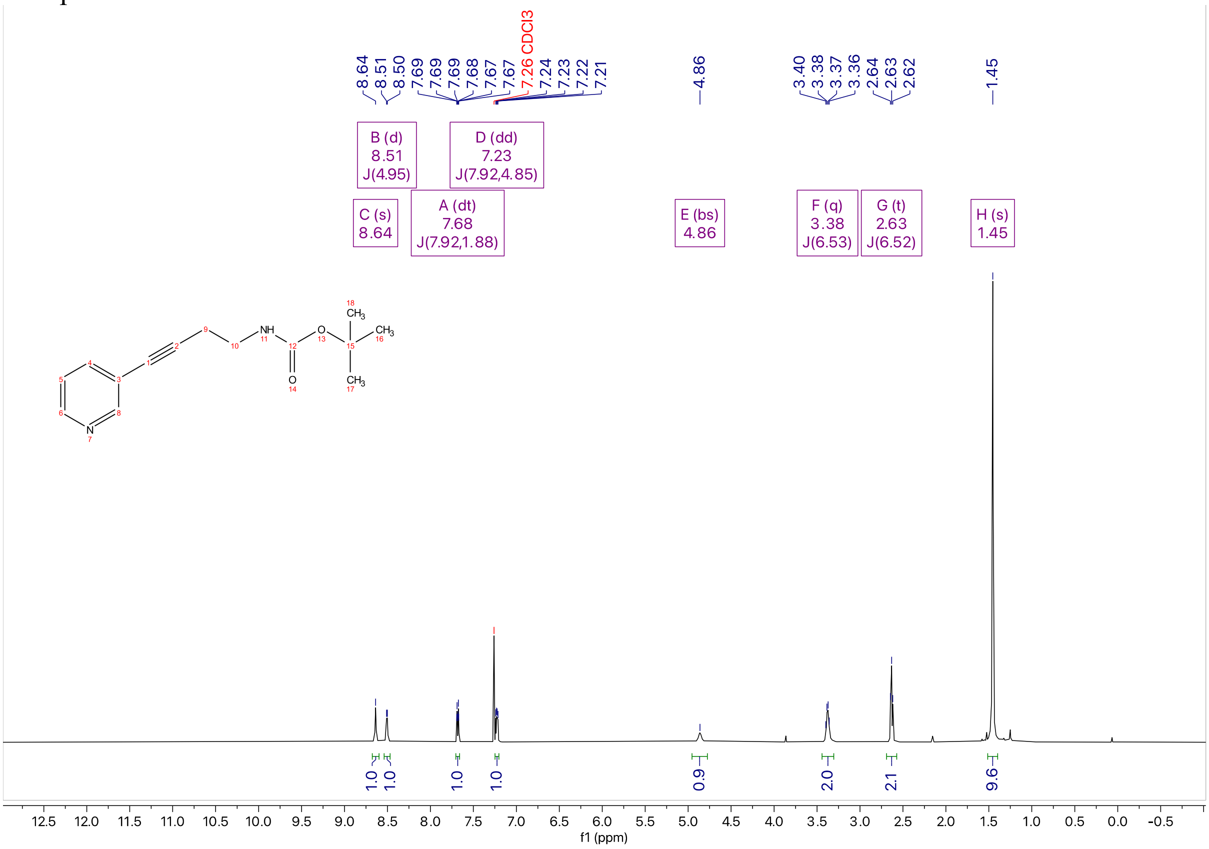

Compound S2a ^{13}C

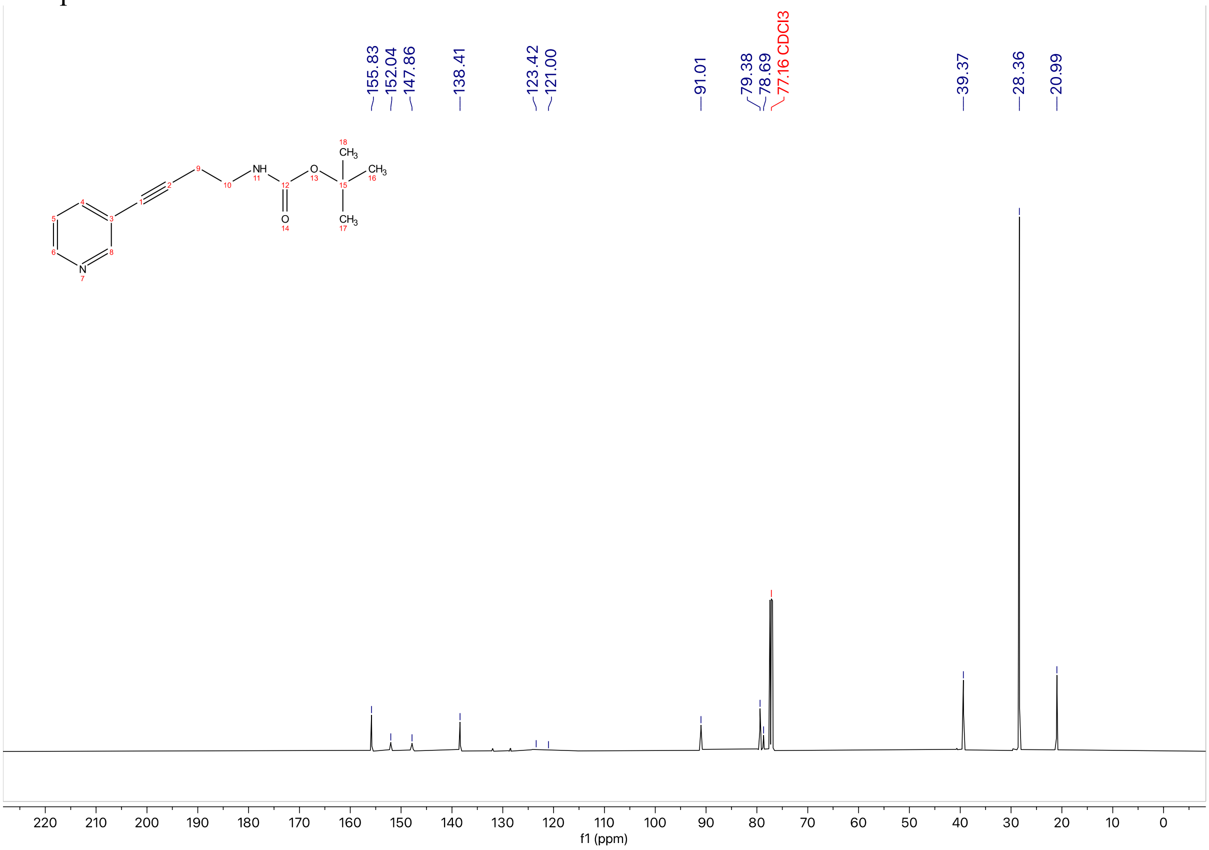

Compound S4 ^{1}H

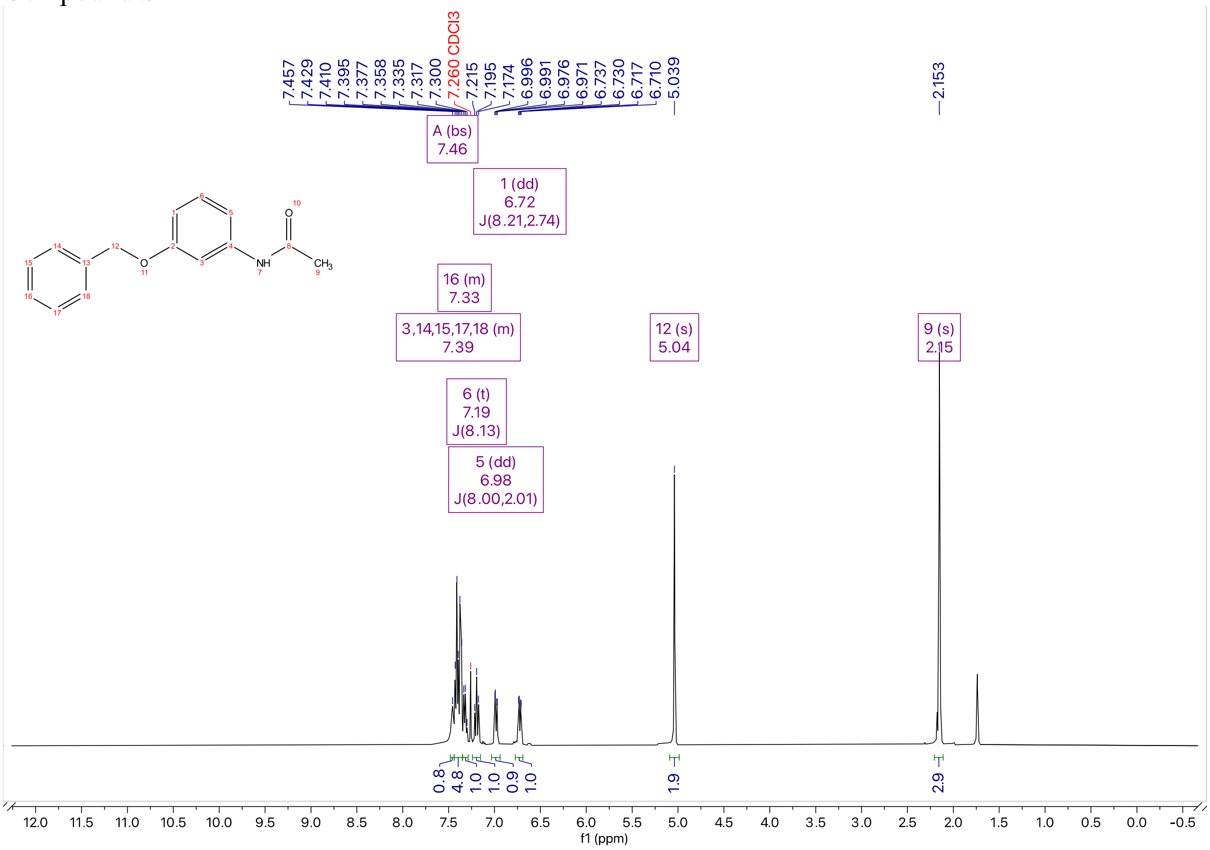

Compound S4 ^{13}C

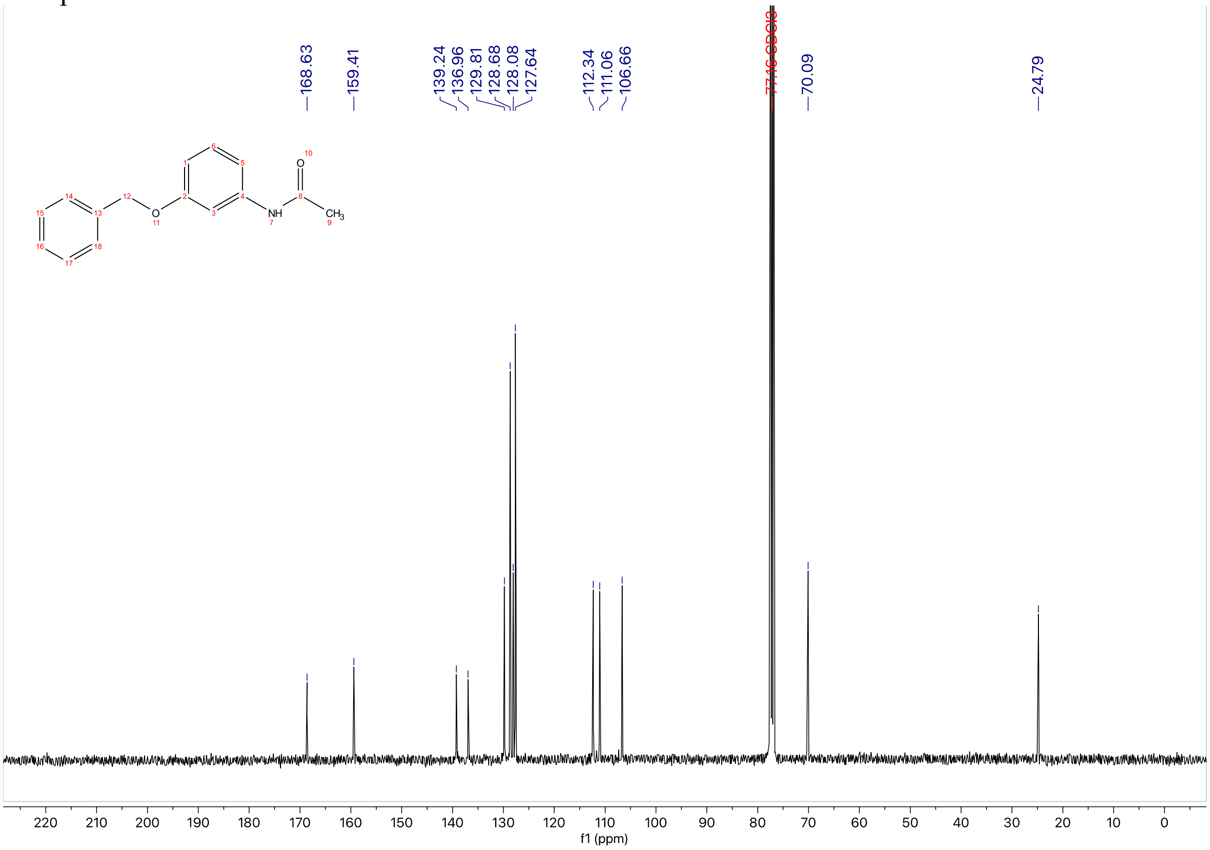

Compound S1b ^{1}H

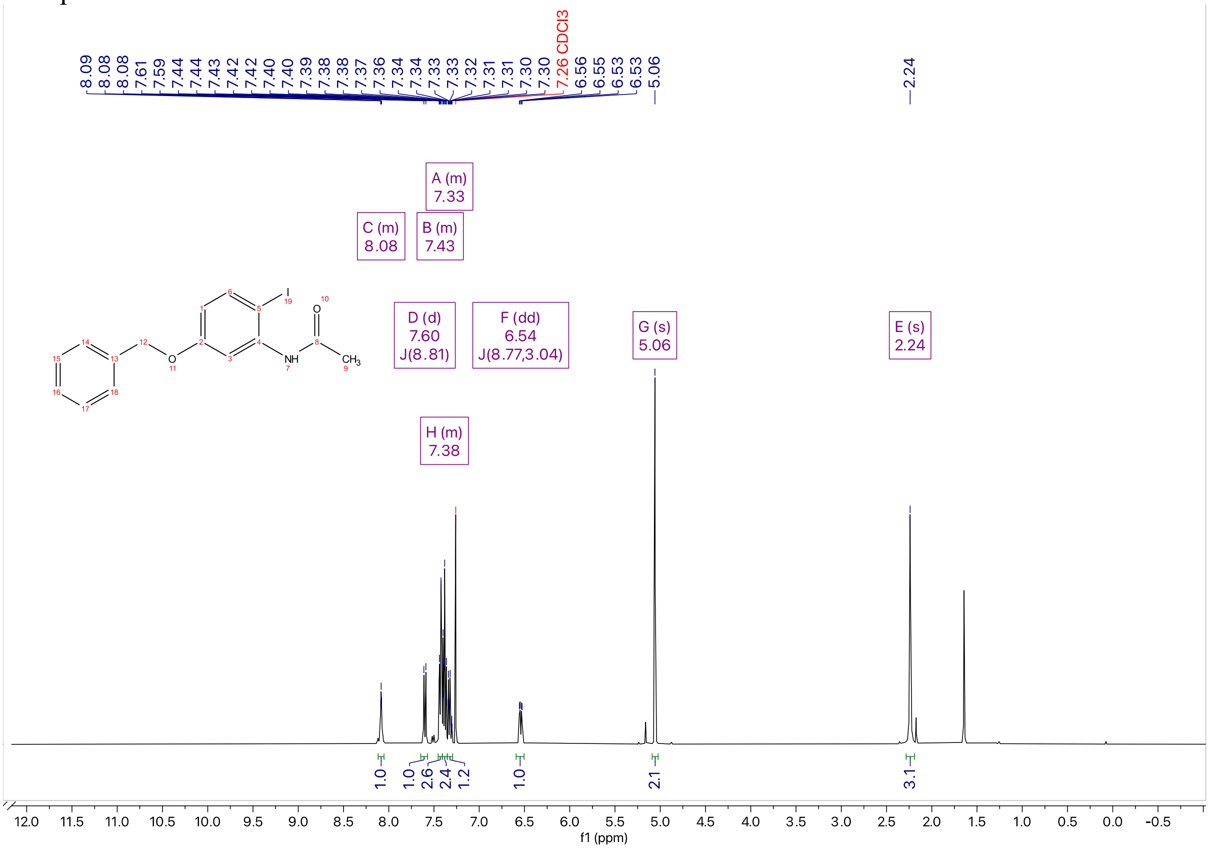

Compound S1b ^{13}C

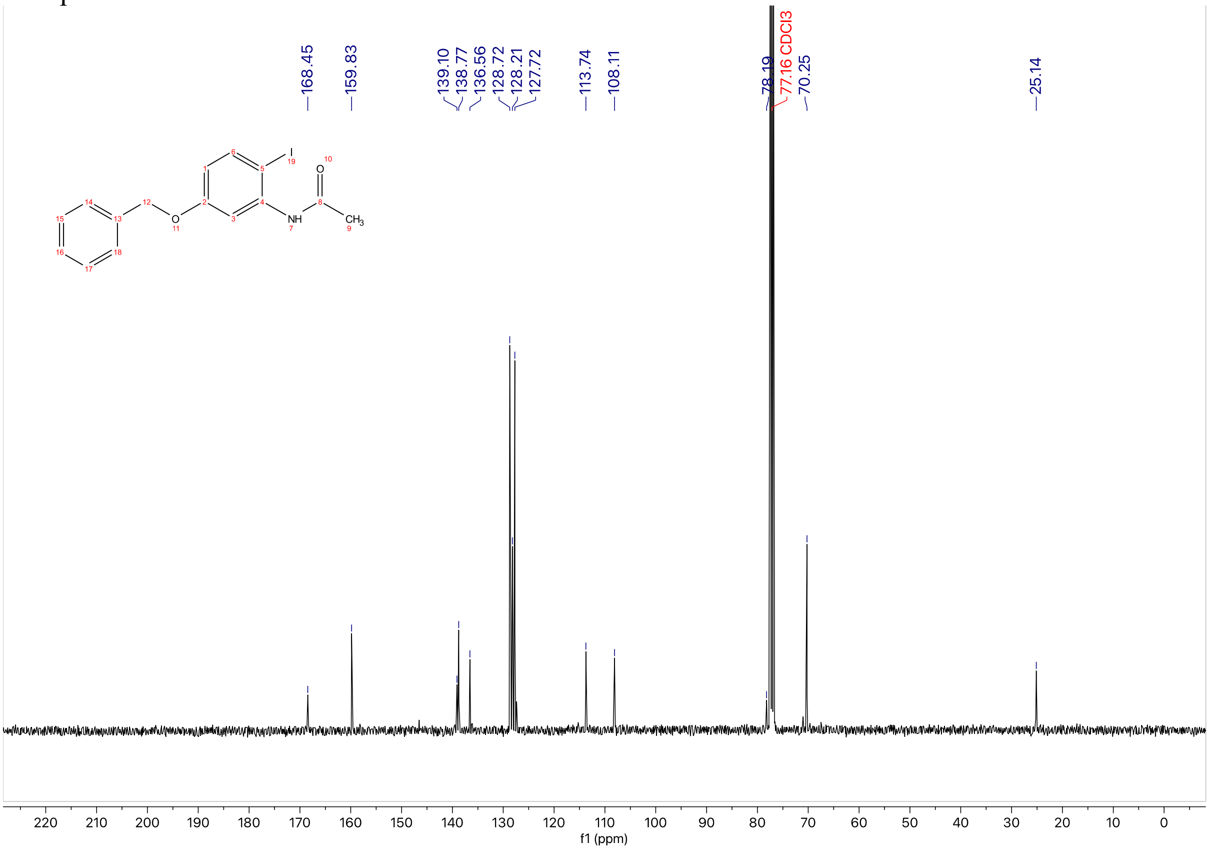

Compound S5 ^{1}H

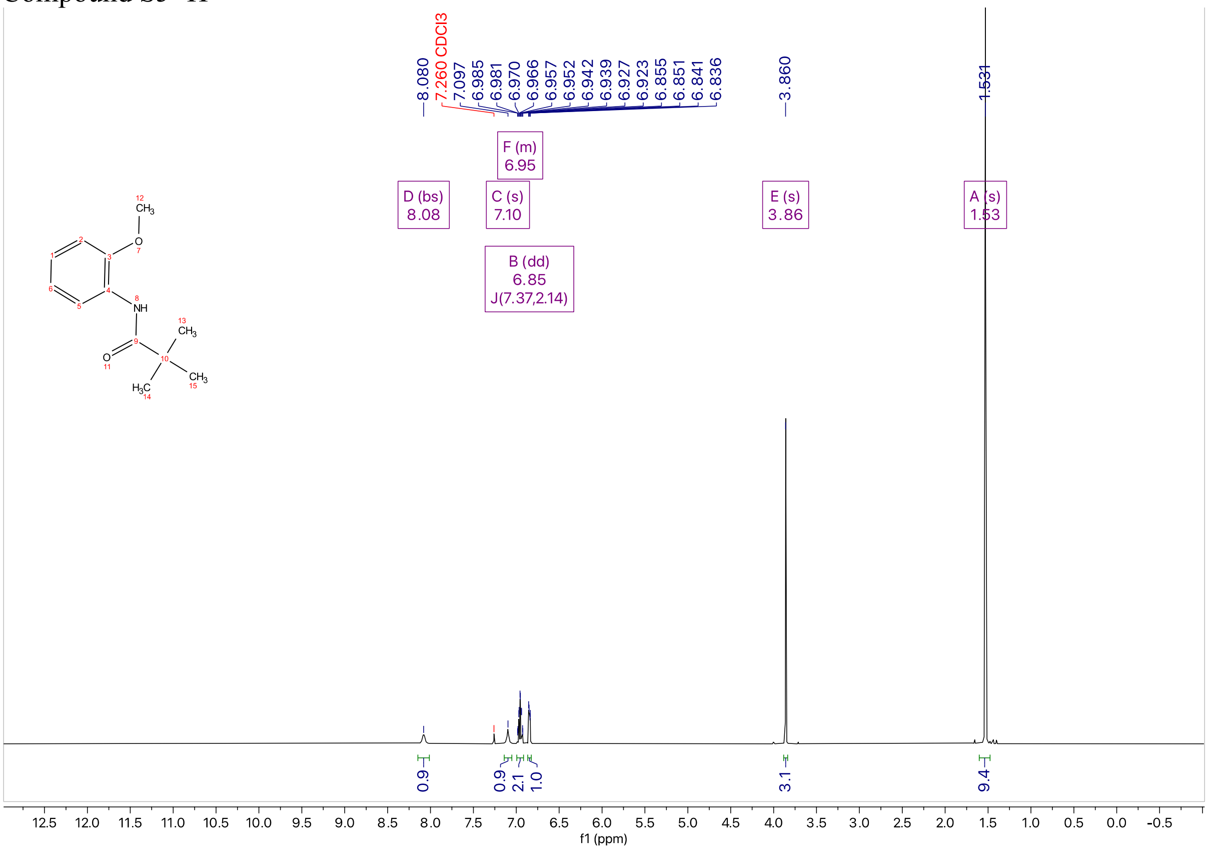

Compound S5 ^{13}C

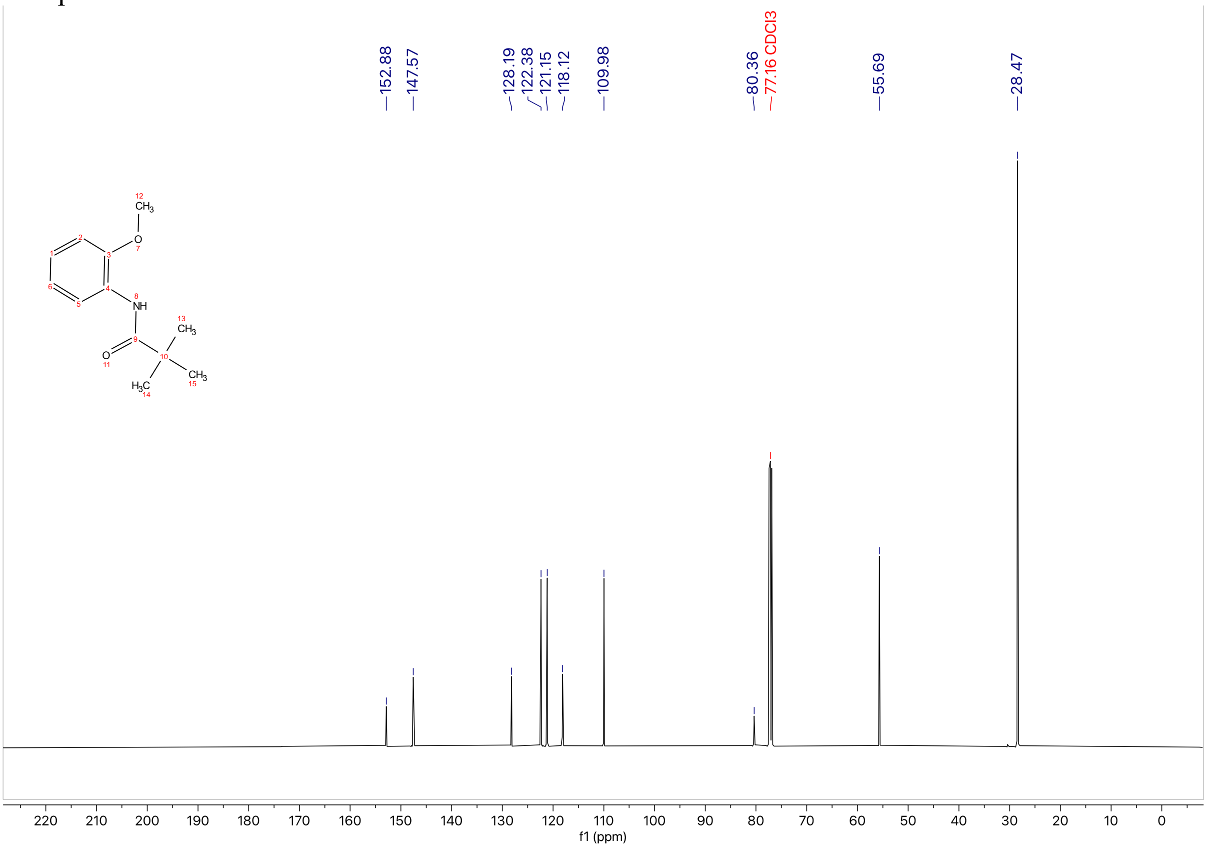

Compound S1c ^{1}H

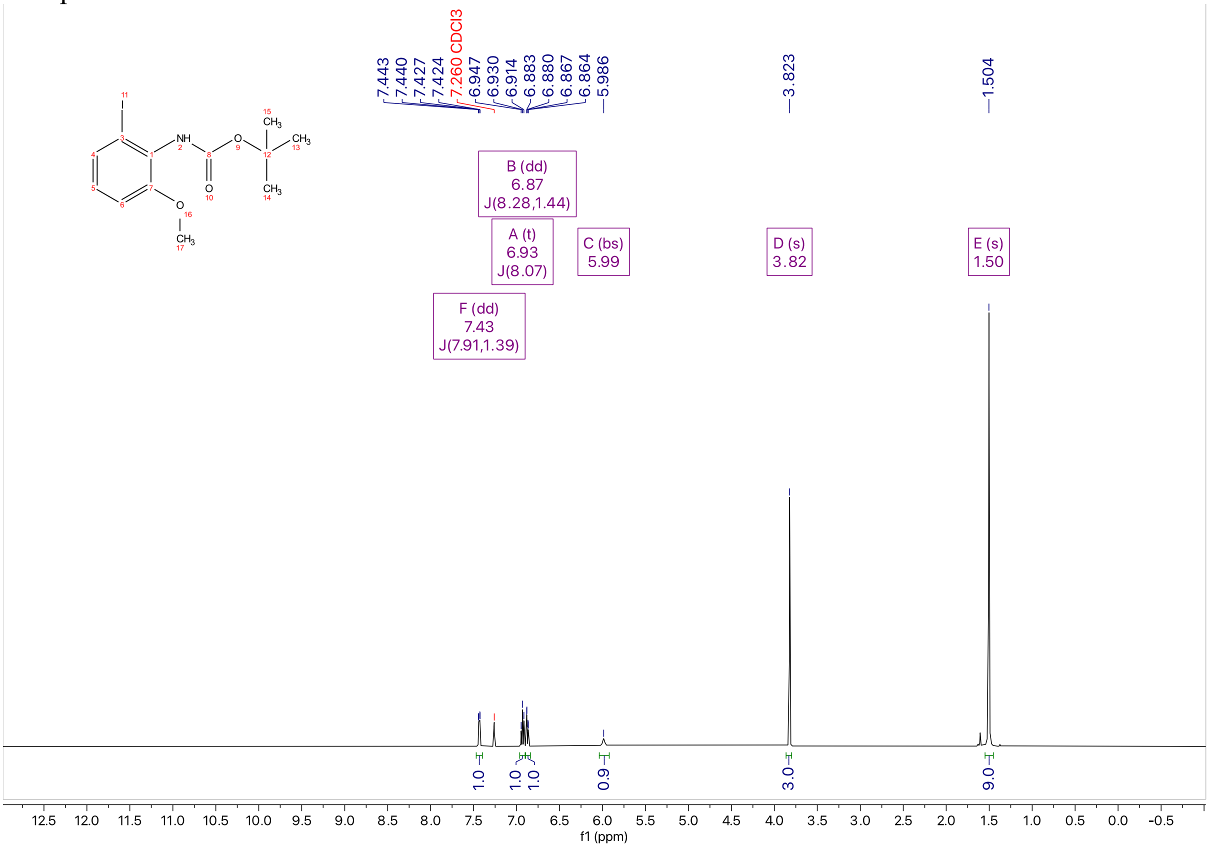

Compound S1c ^{13}C

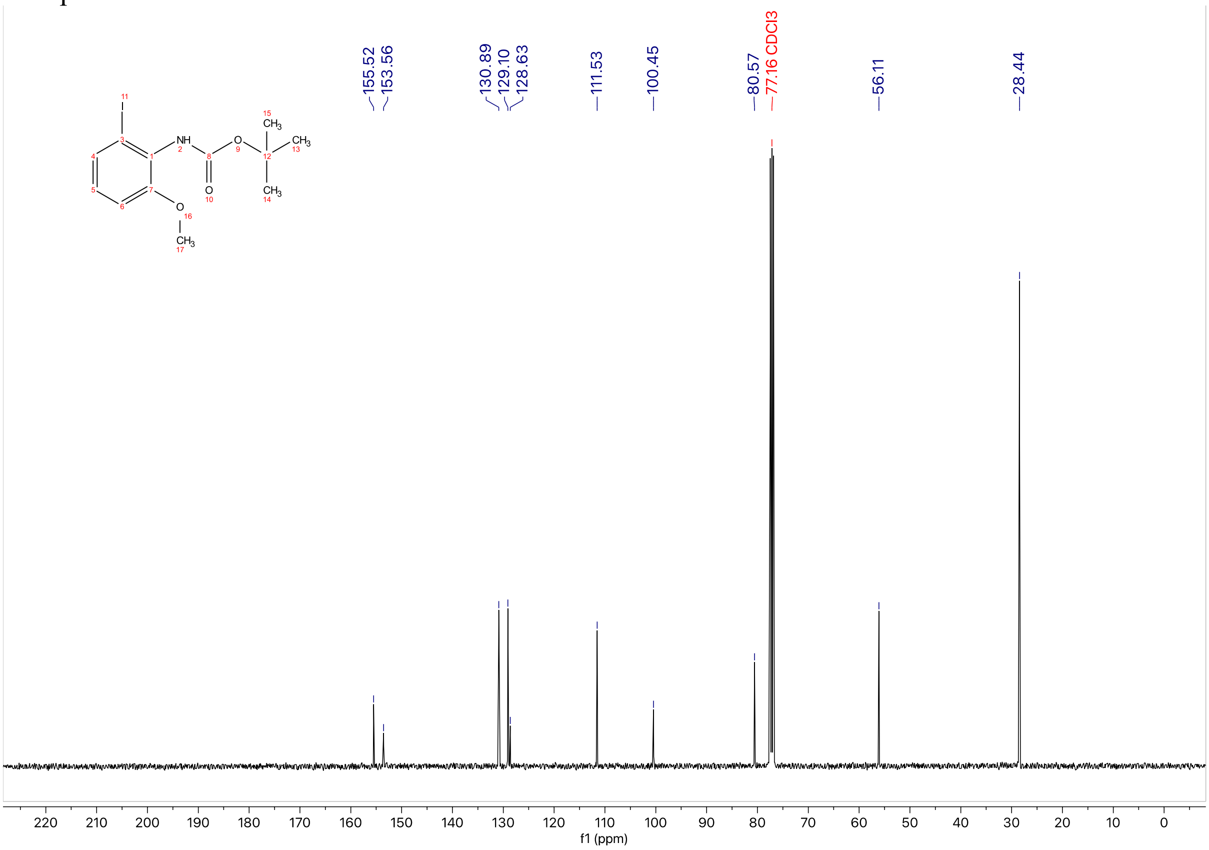

Compound S6 ^{1}H

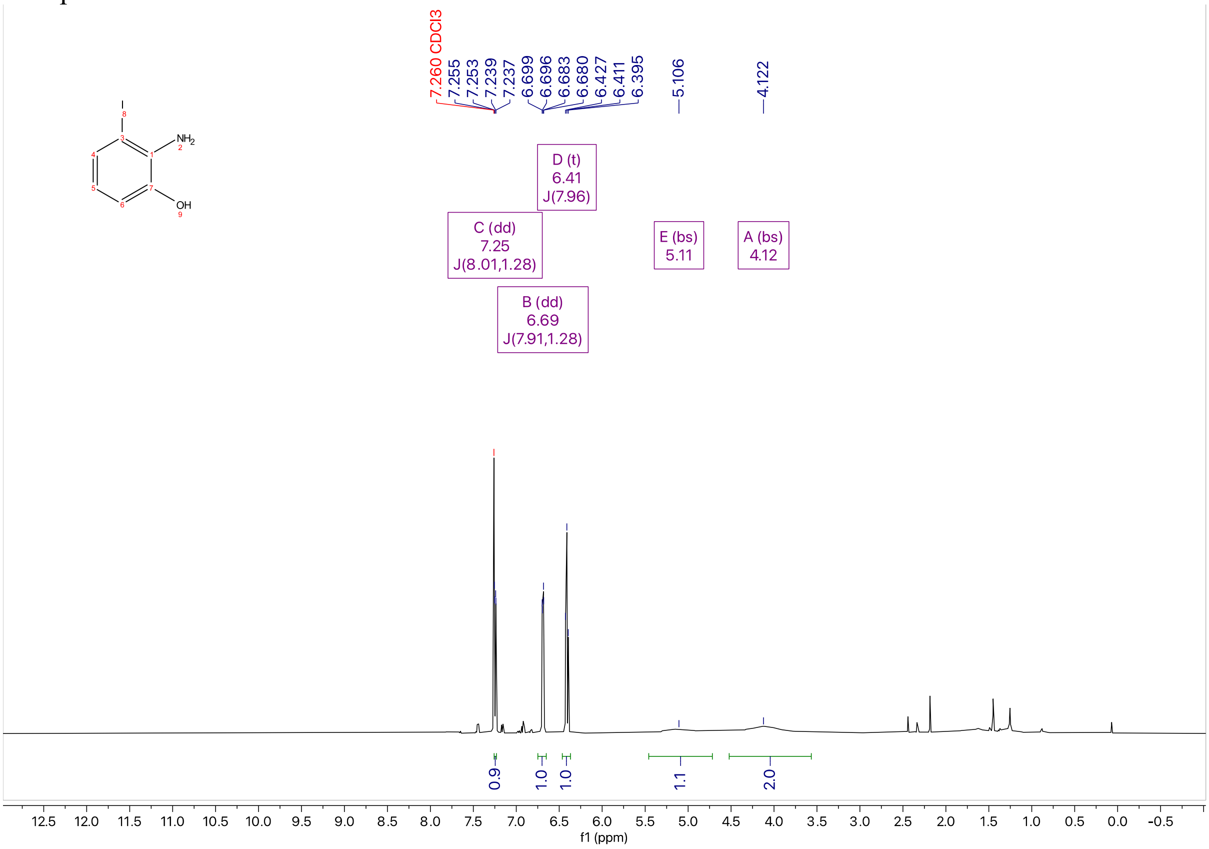

Compound S6 ^{13}C

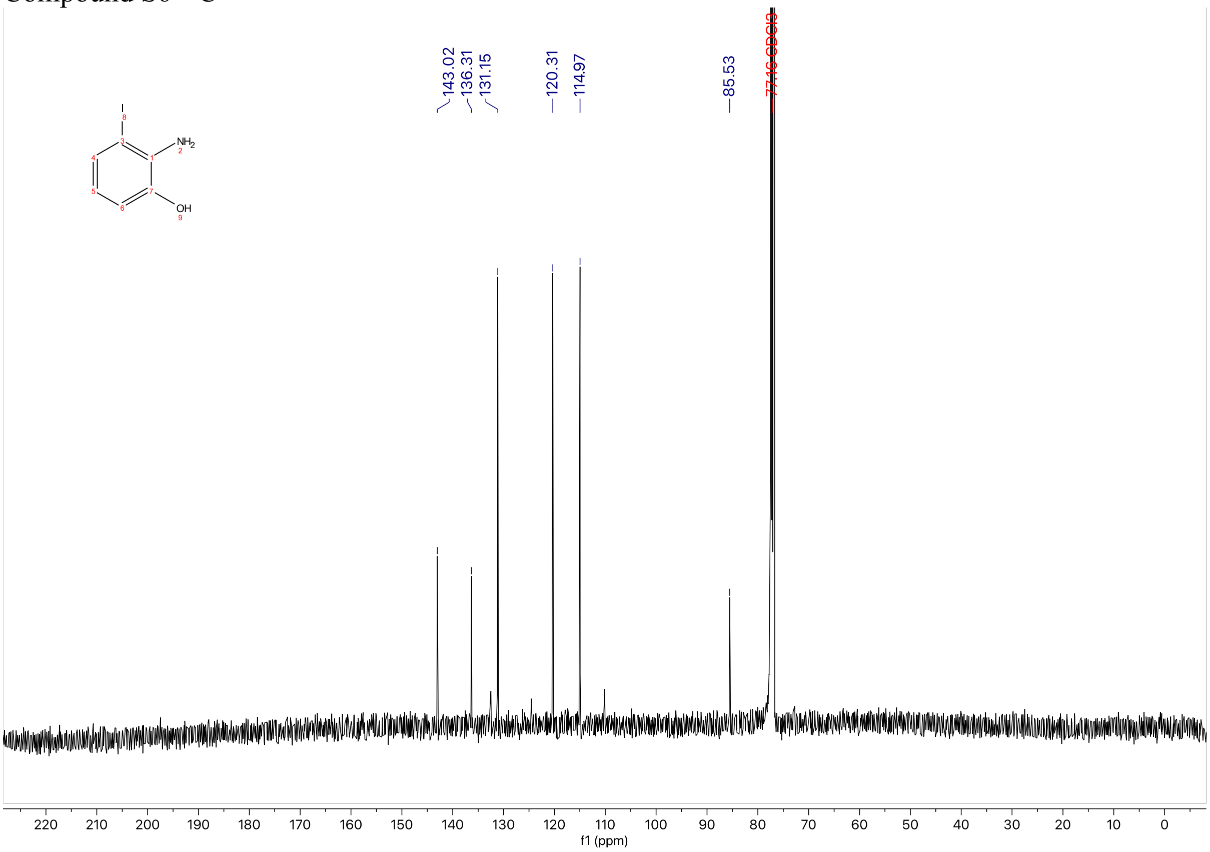

Compound S7 ^{1}H

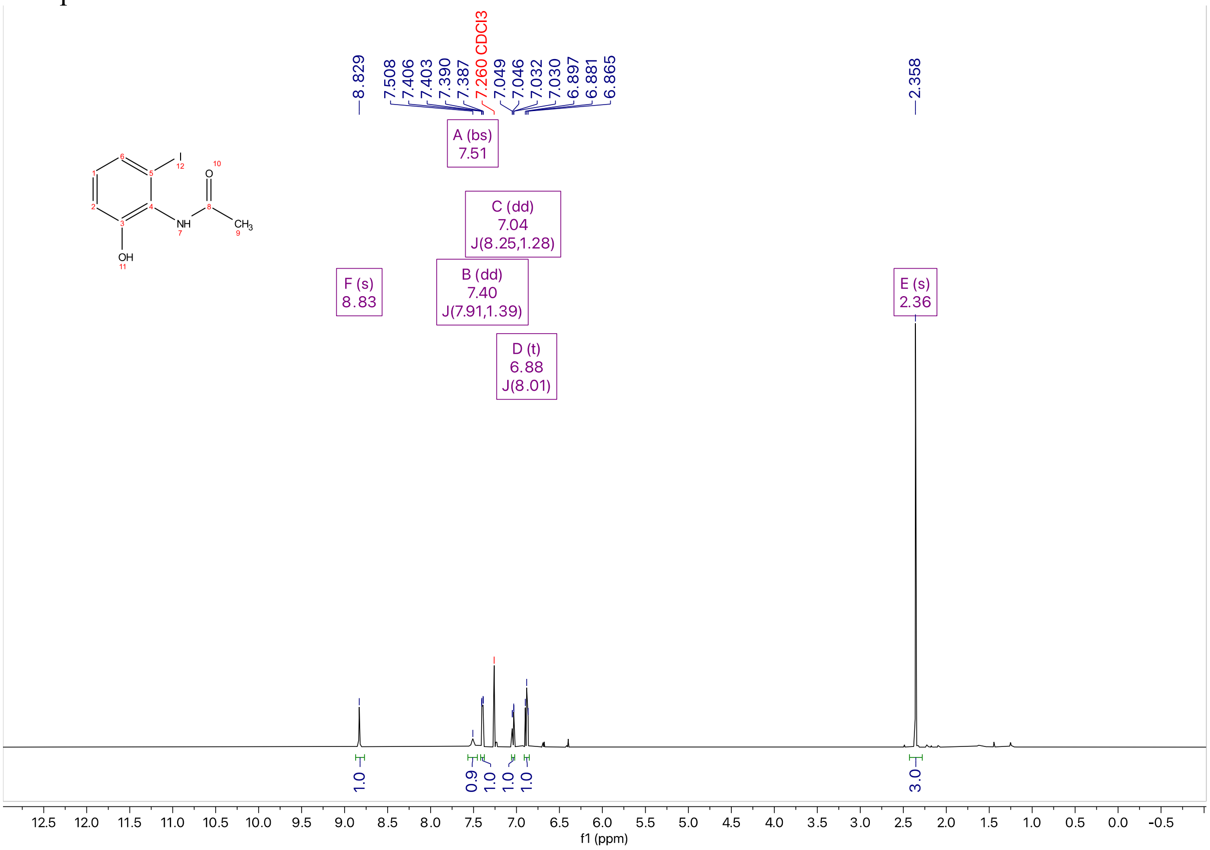

Compound S7 ^{13}C

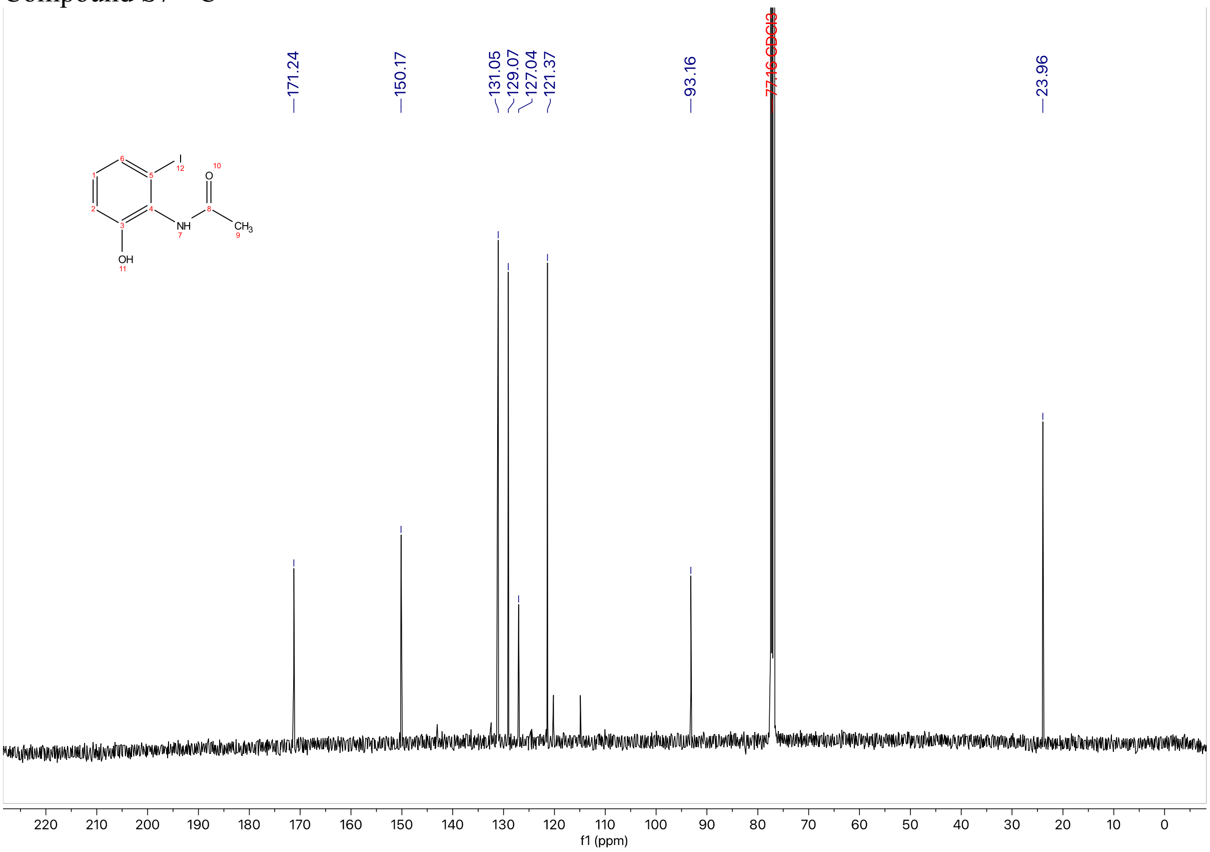

Compound S1d ^{1}H

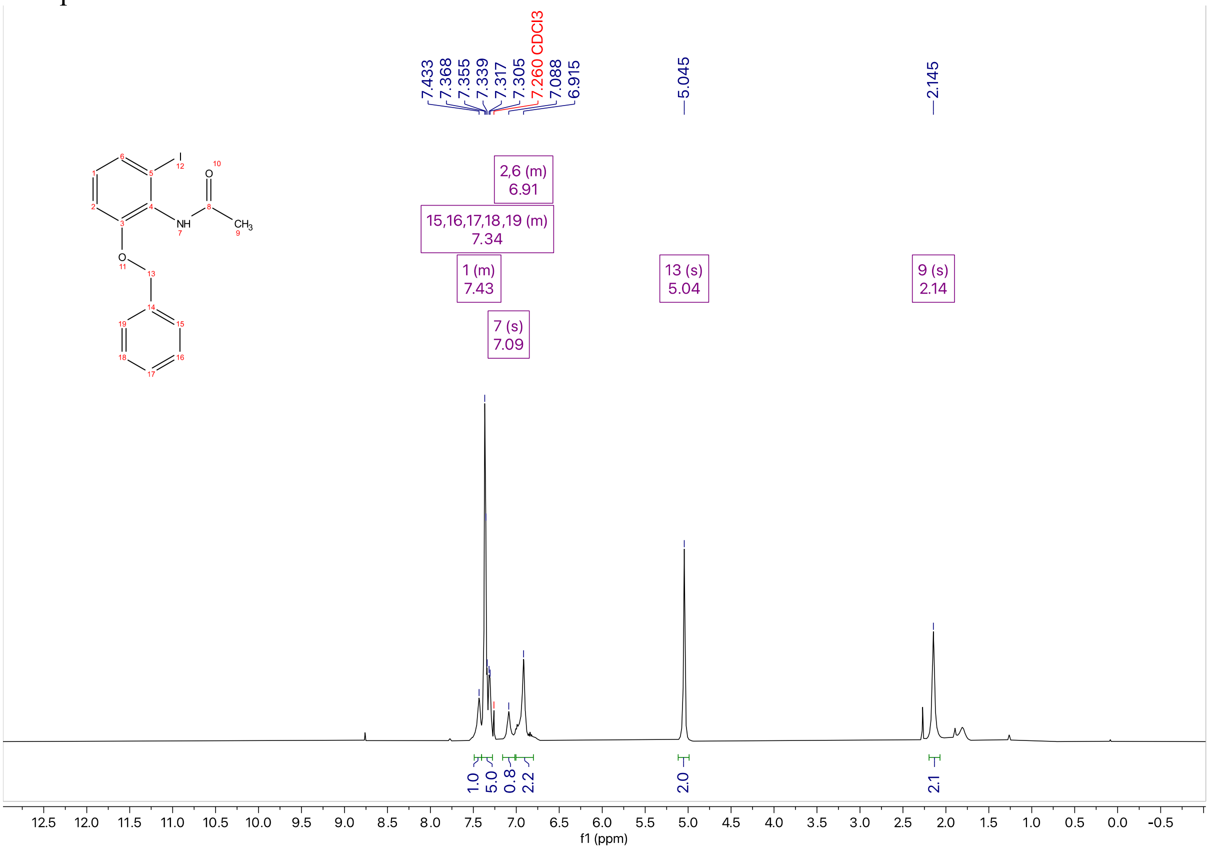

Compound S1d ^{13}C

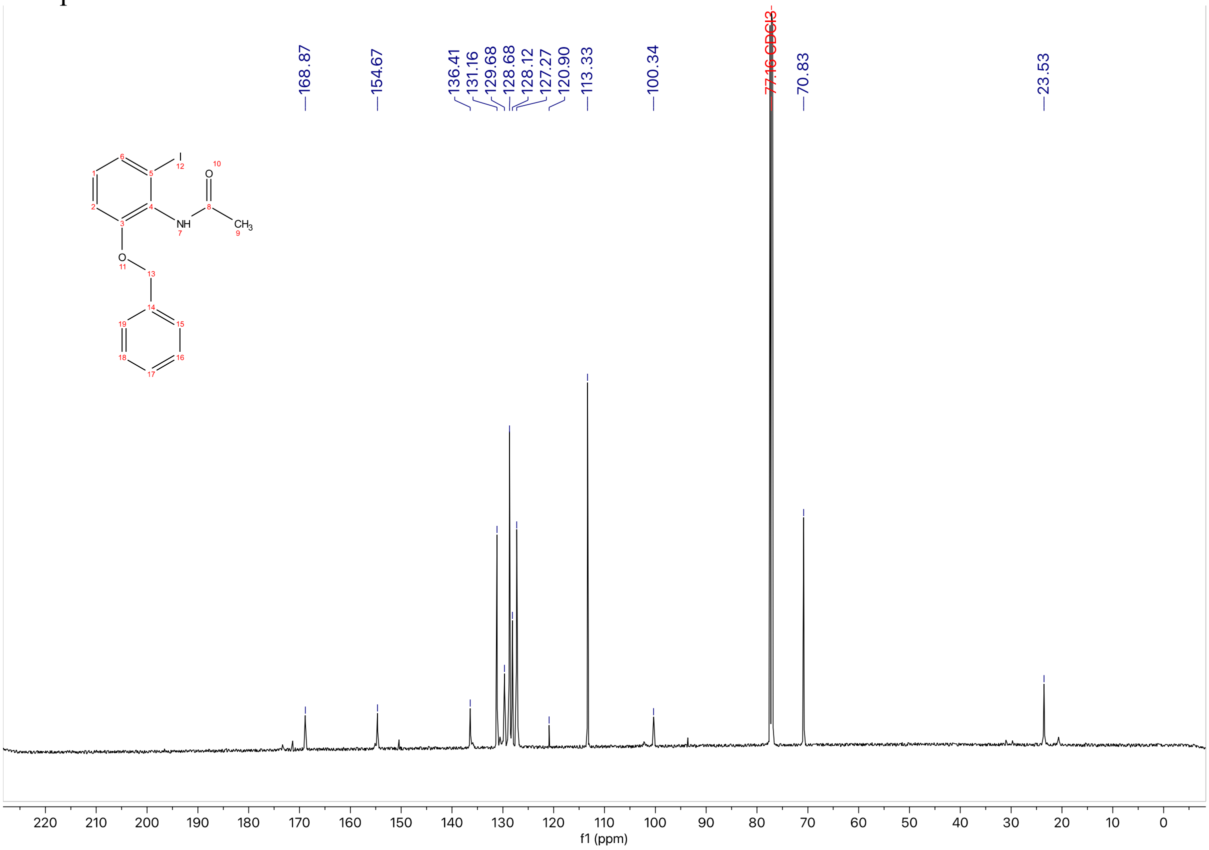

Compound S8 ^{1}H

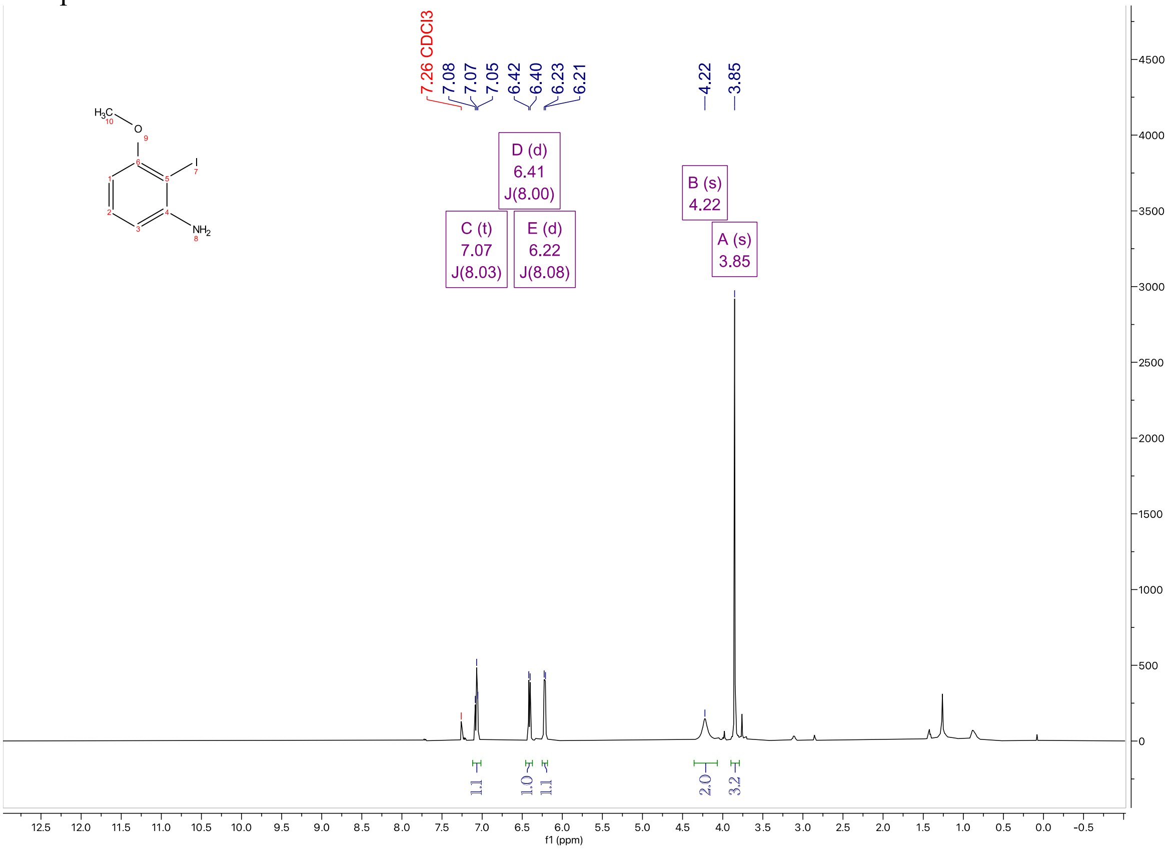

Compound S8 ^{13}C

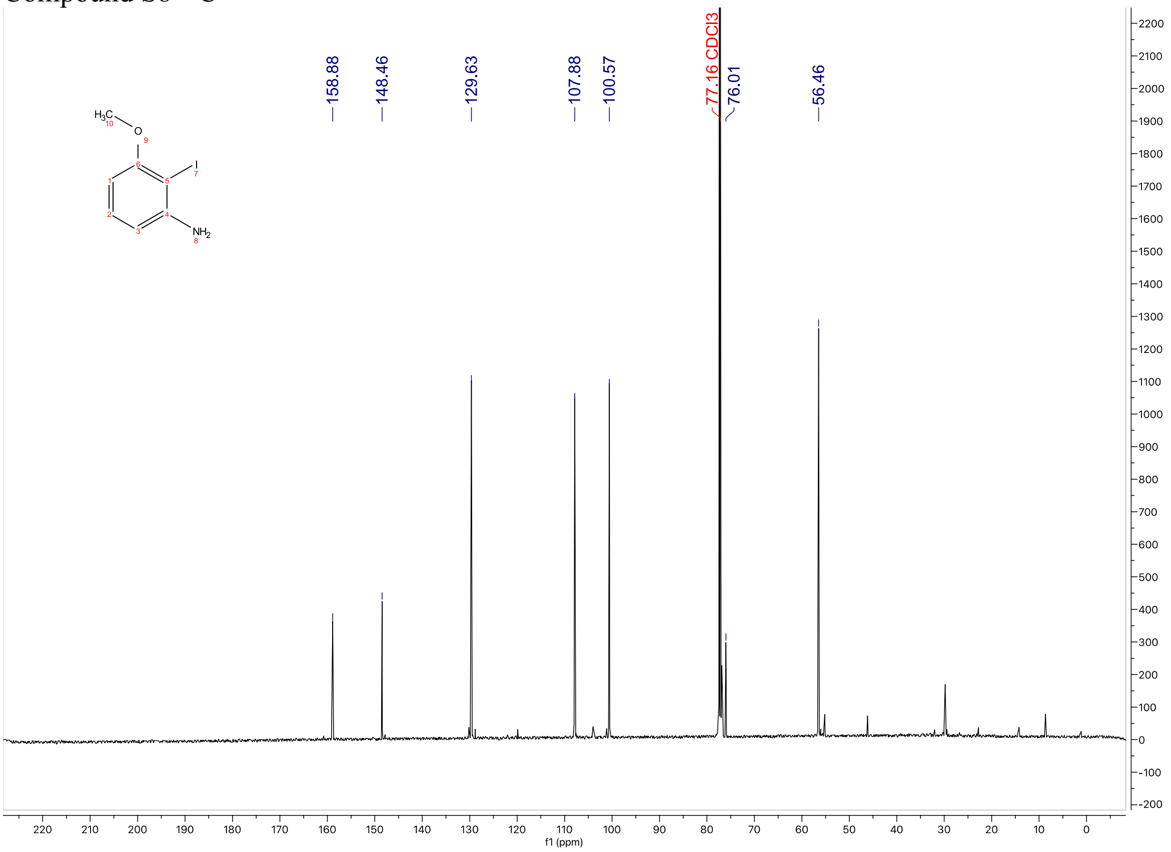

Compound S1e ^{1}H

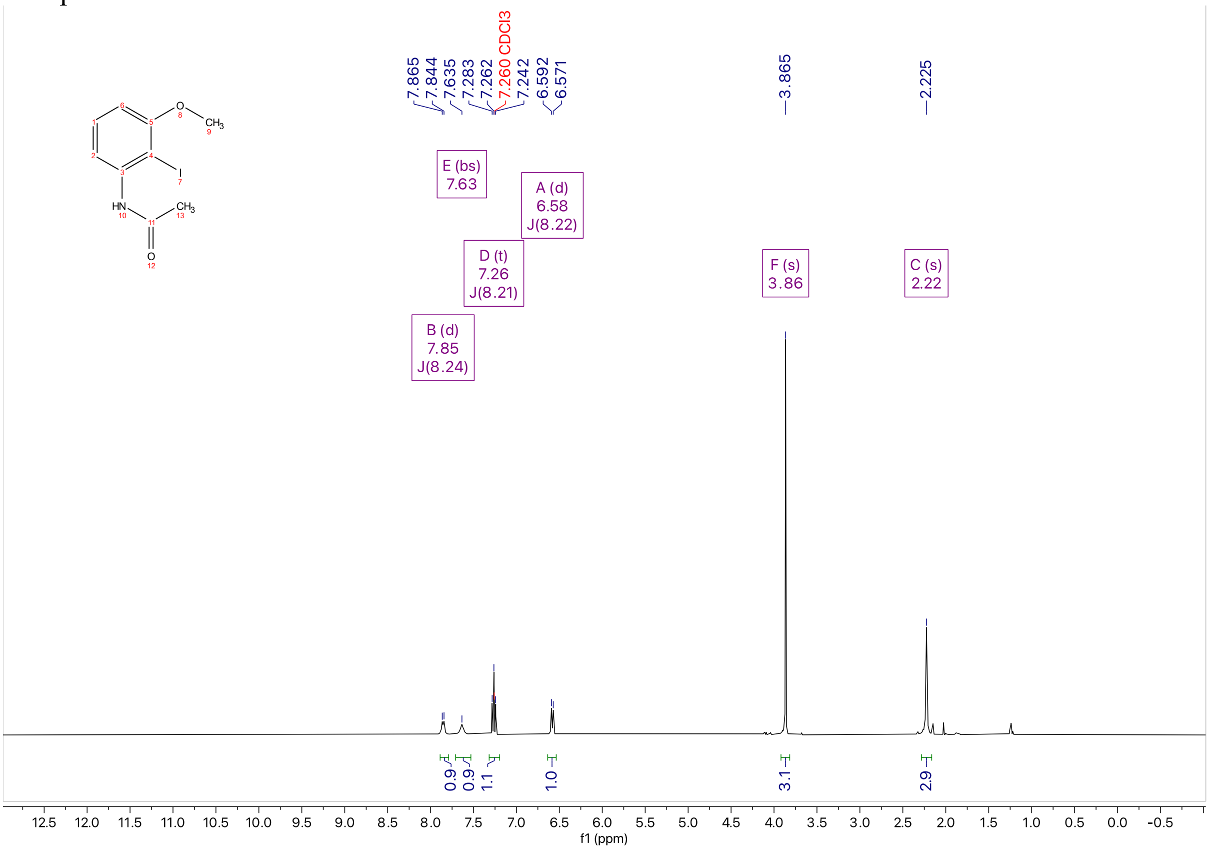

Compound S1e ^{13}C DEPTQ

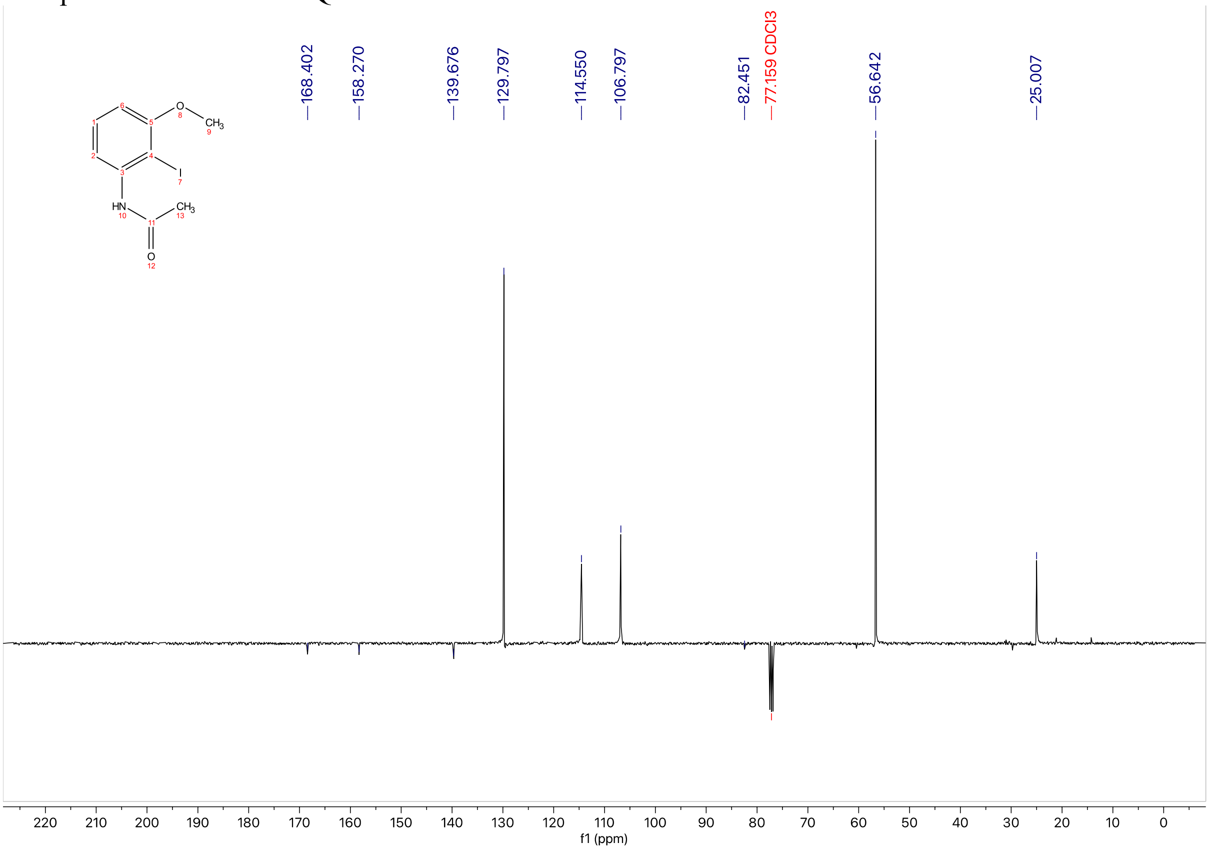

Compound S1f ^{1}H

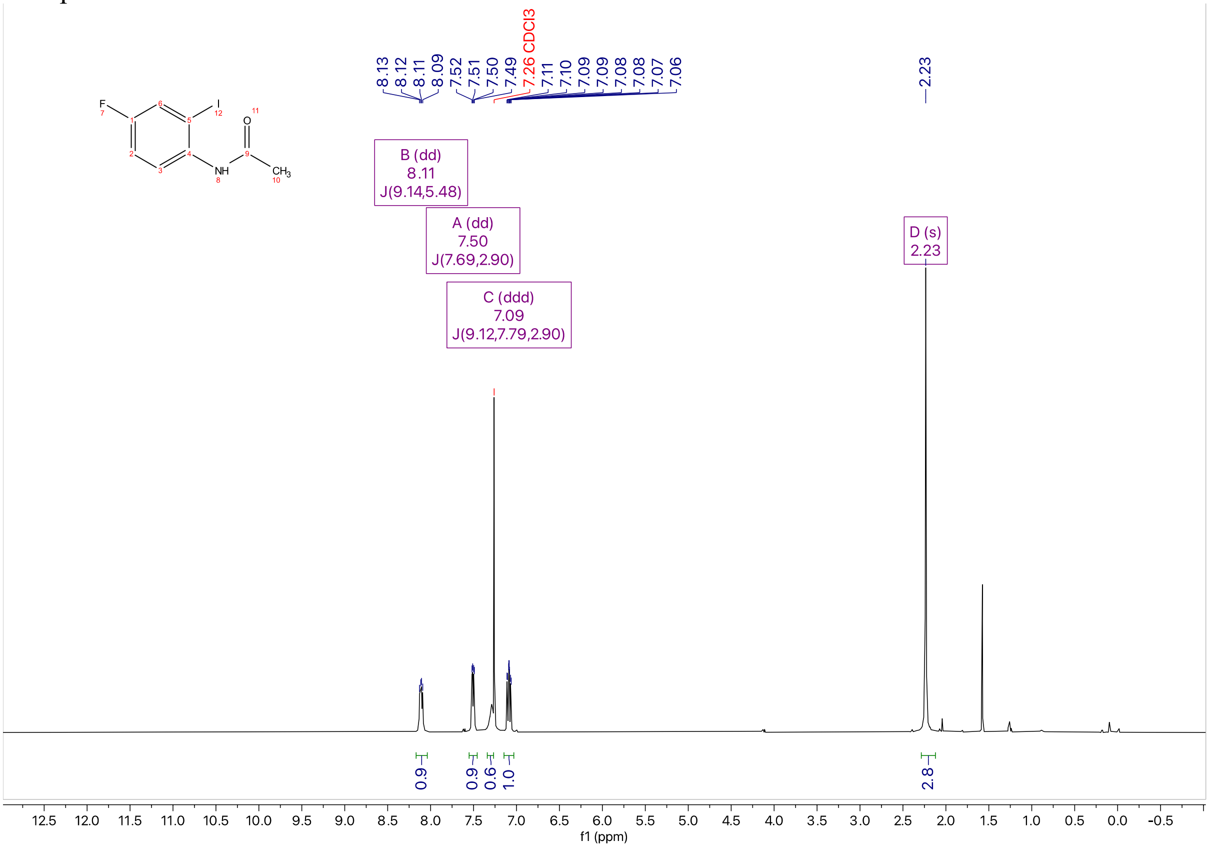

Compound S1f ^{13}C

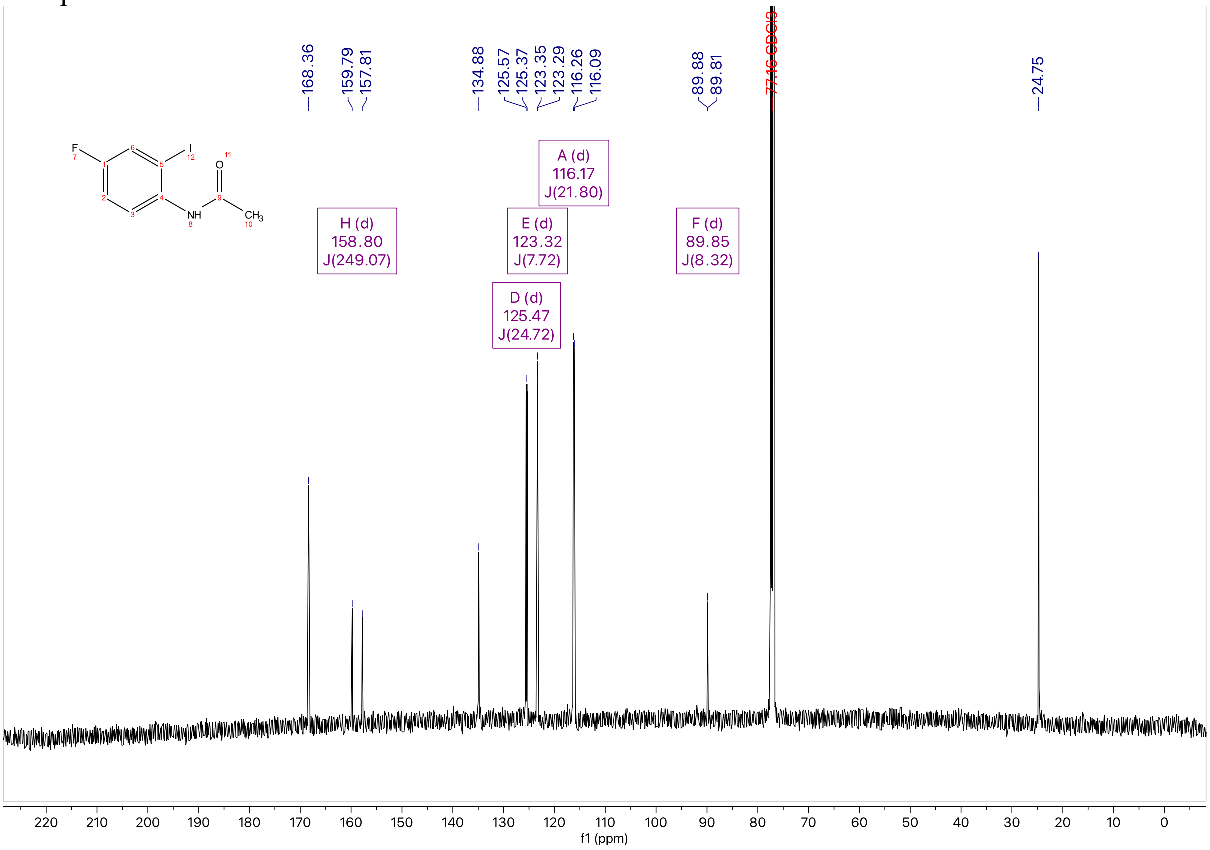

Compound S1f ^{19}F

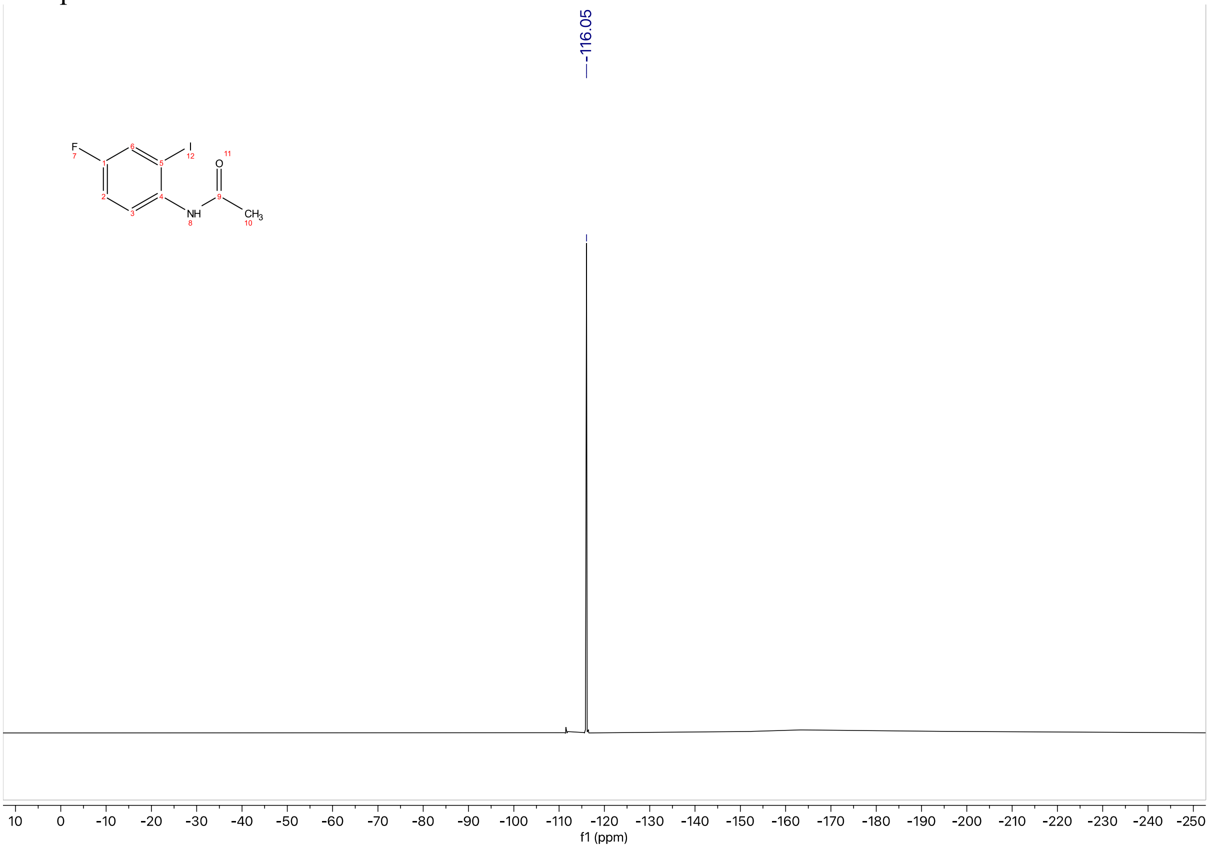

Compound S1g ^{1}H

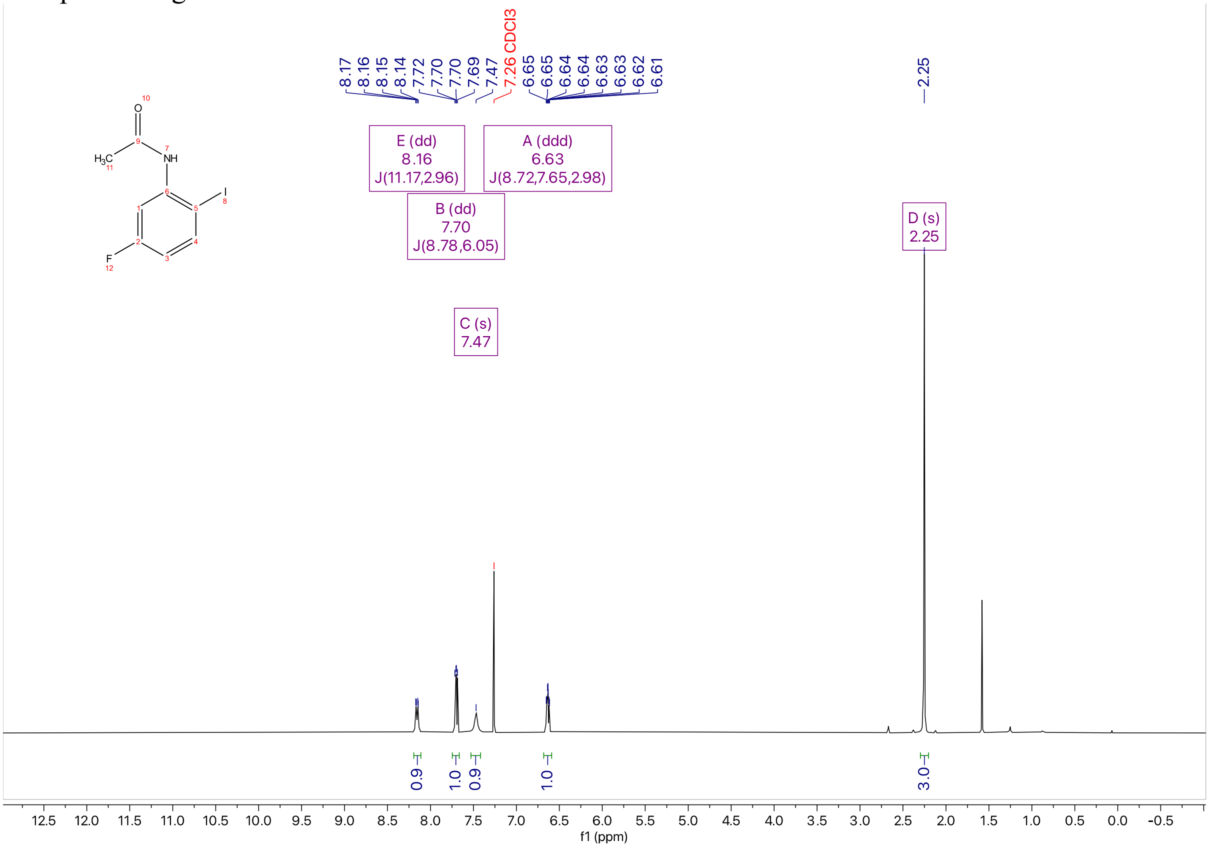

Compound S1g ^{13}C

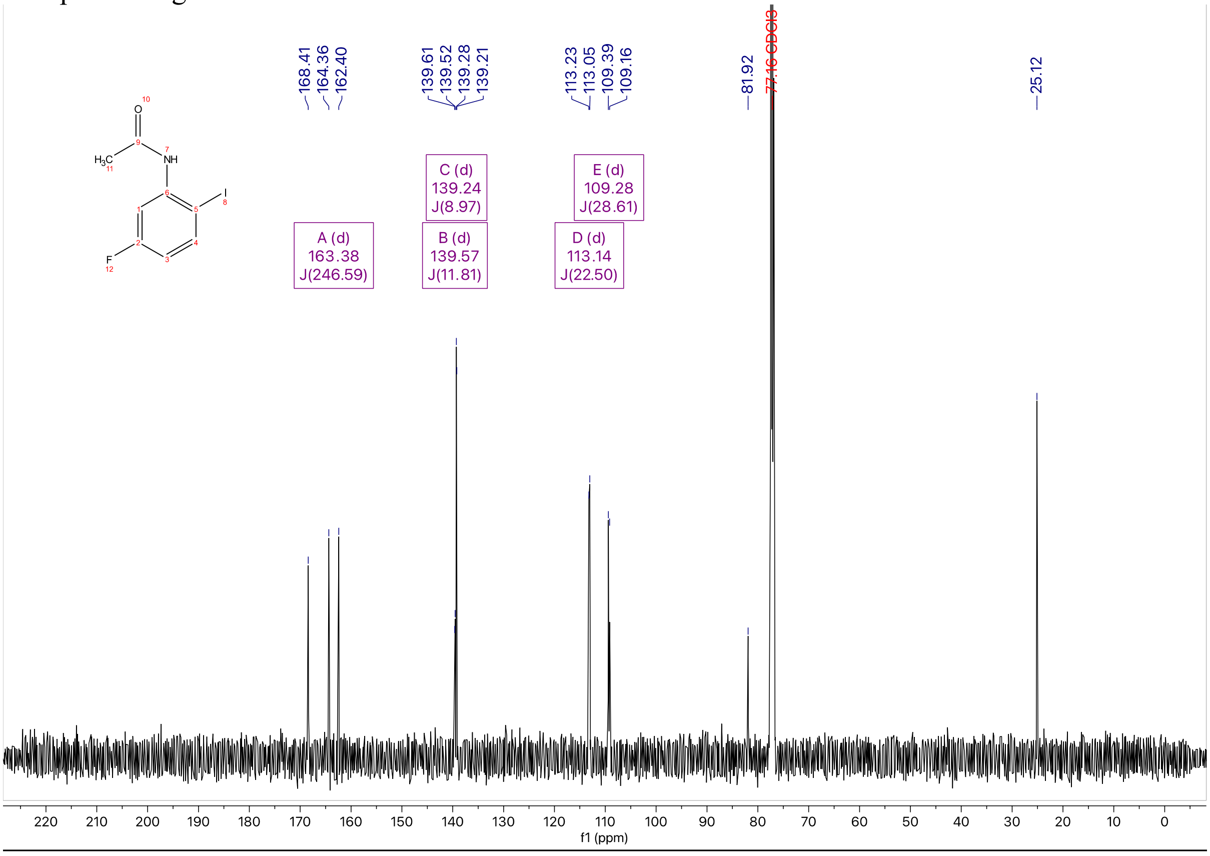

Compound S1g ^{19}F

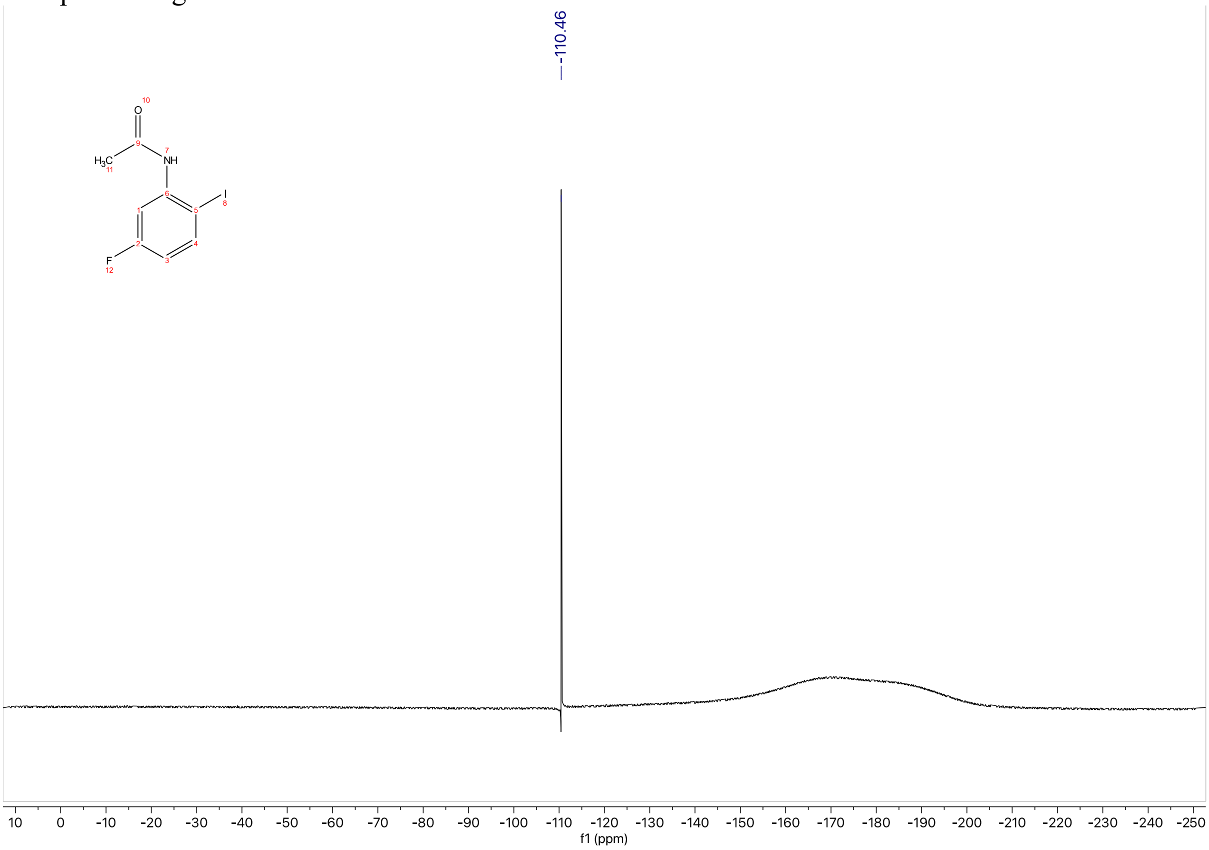

Compound S9 ^{1}H

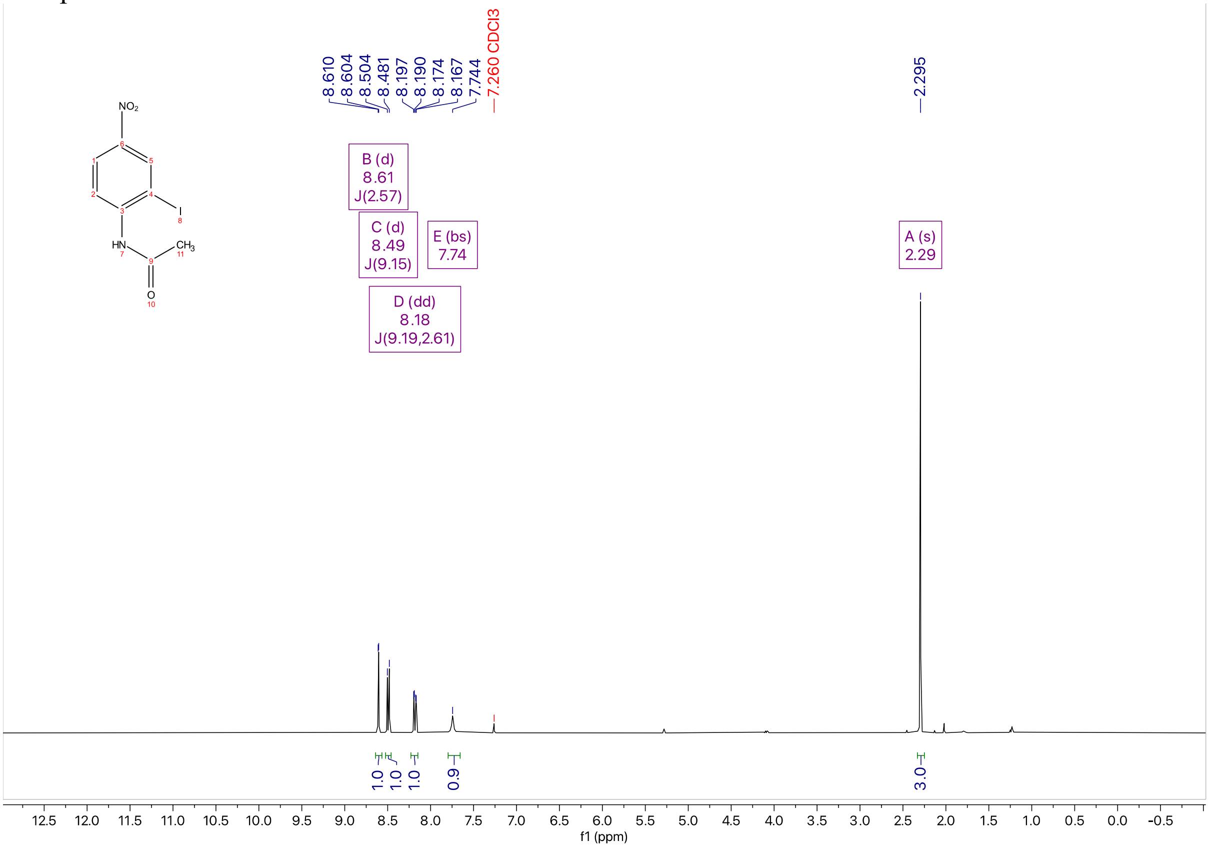

Compound S9 ^{13}C

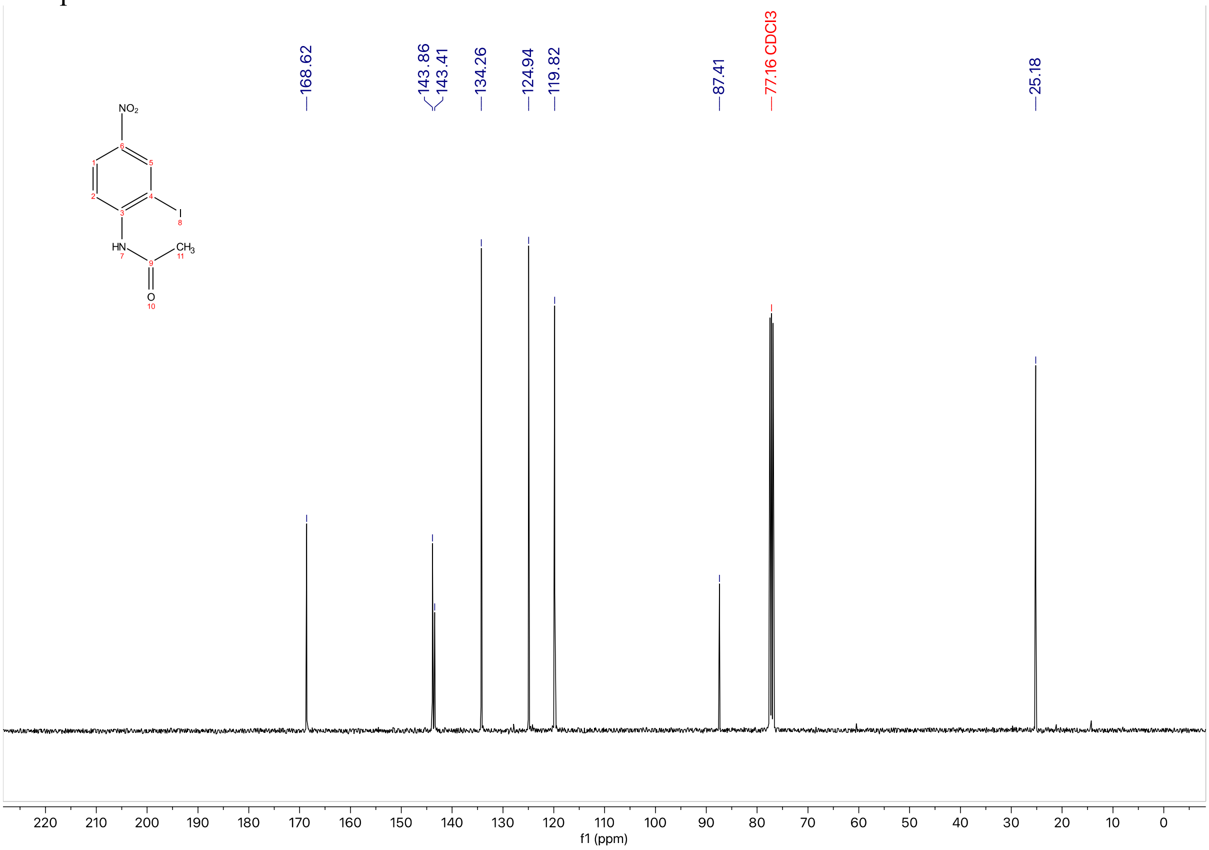

Compound S10 ^{1}H

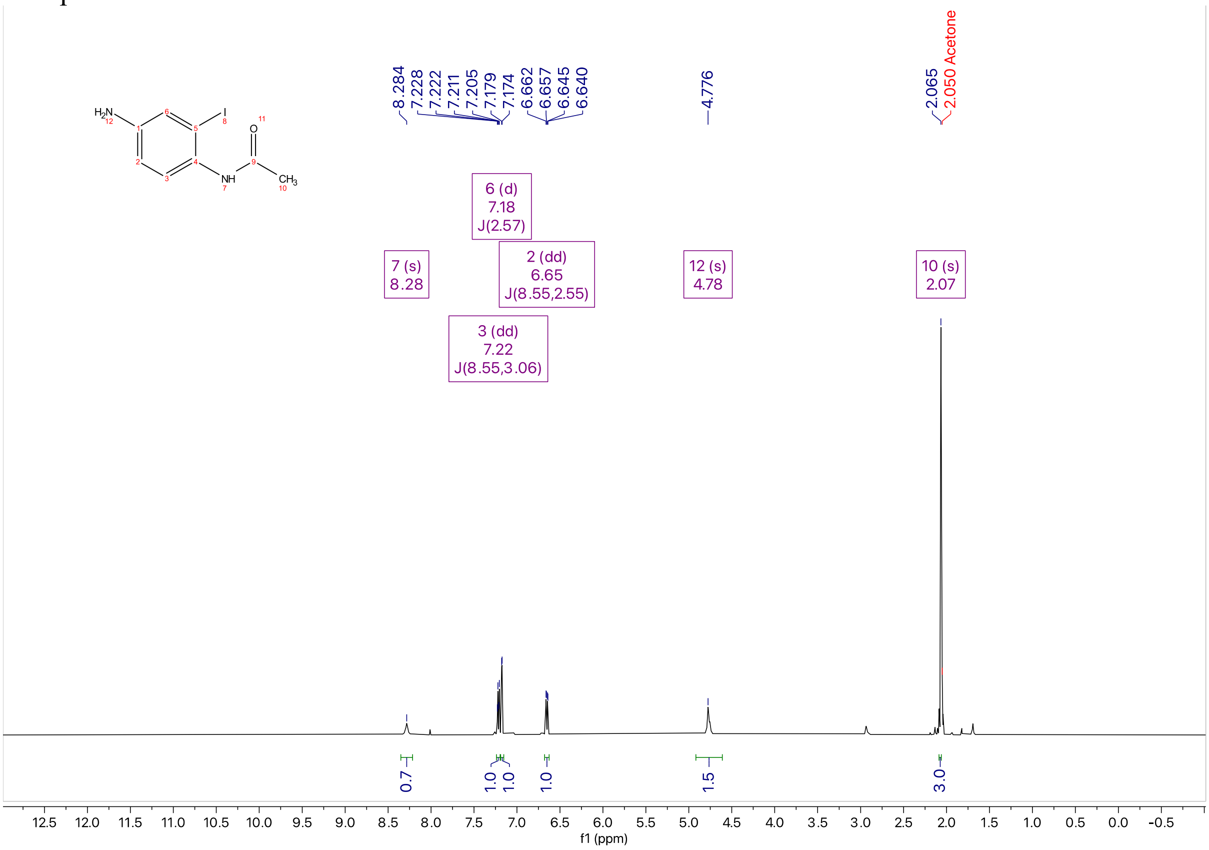

Compound S10 ^{13}C

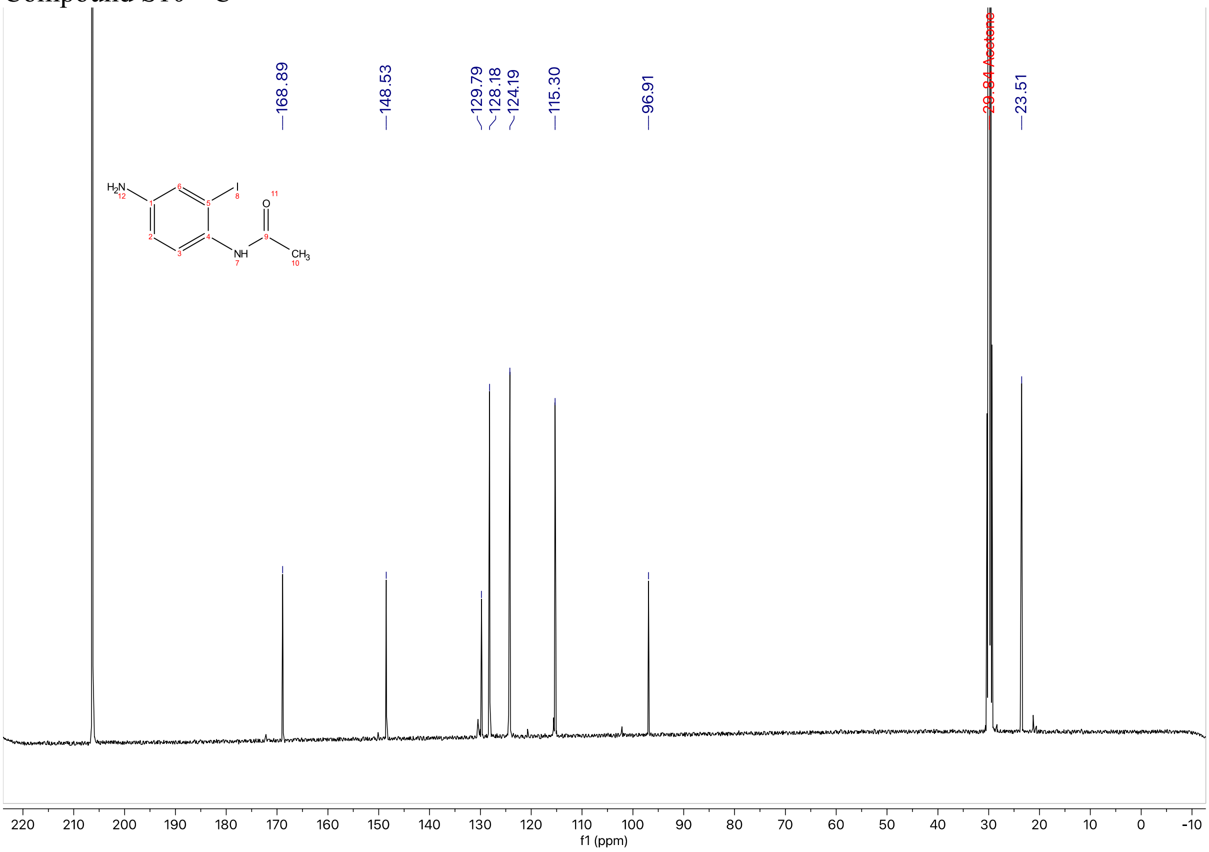

Compound S1h ^{1}H

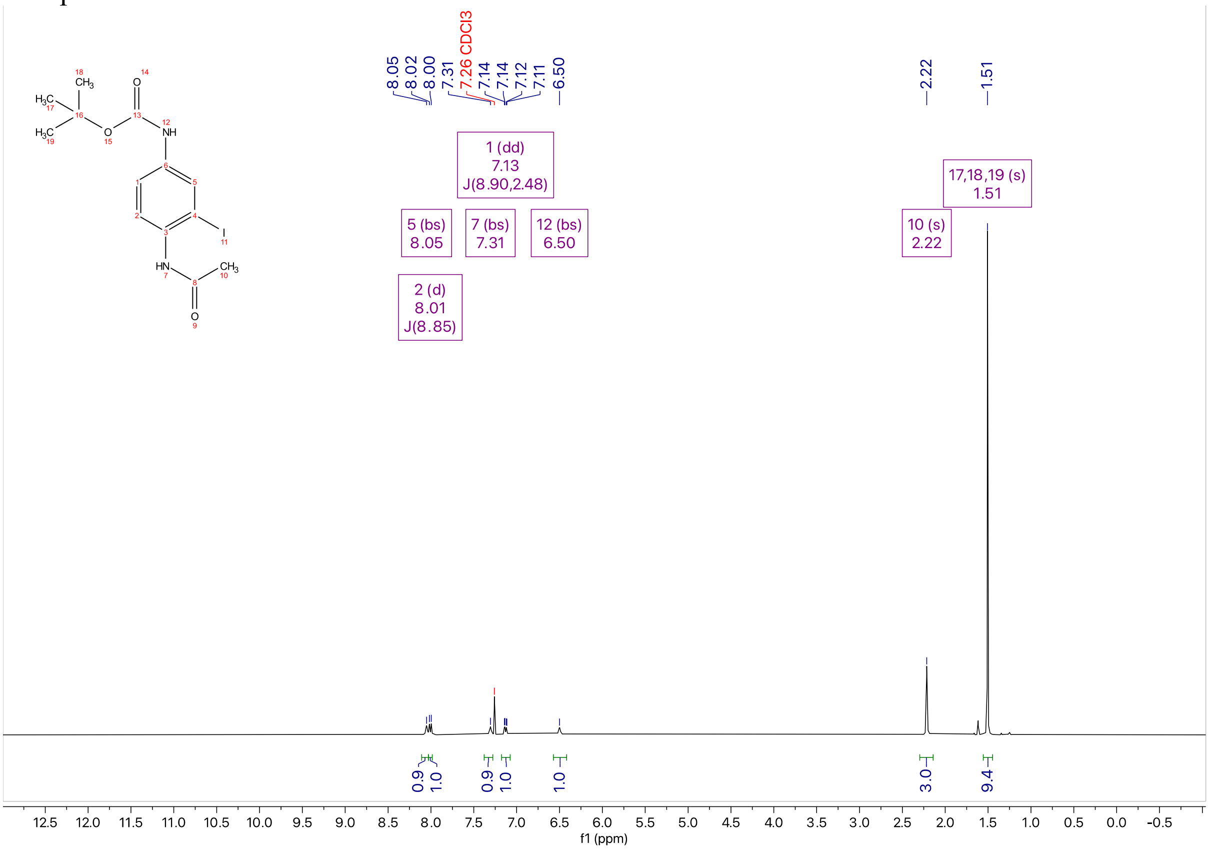

Compound S1h ^{13}C

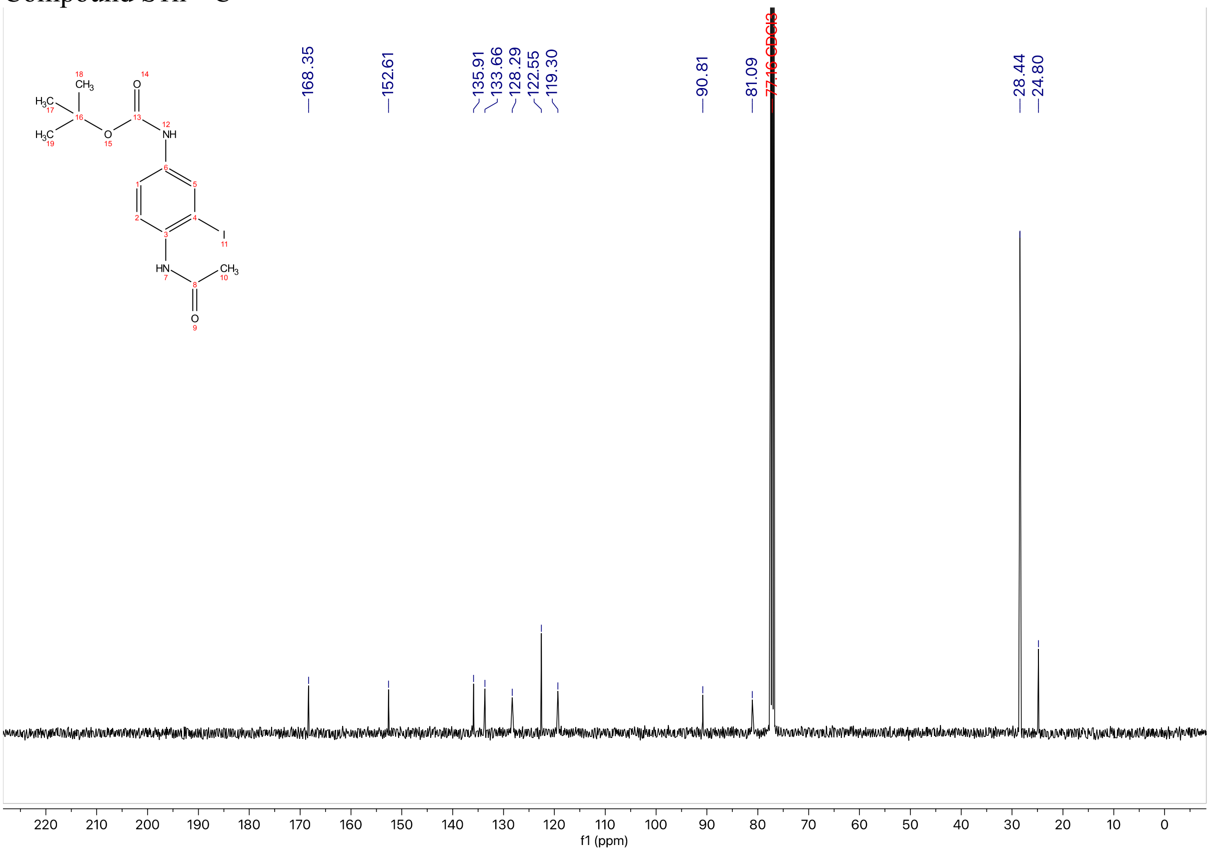

Compound S1i ^{1}H

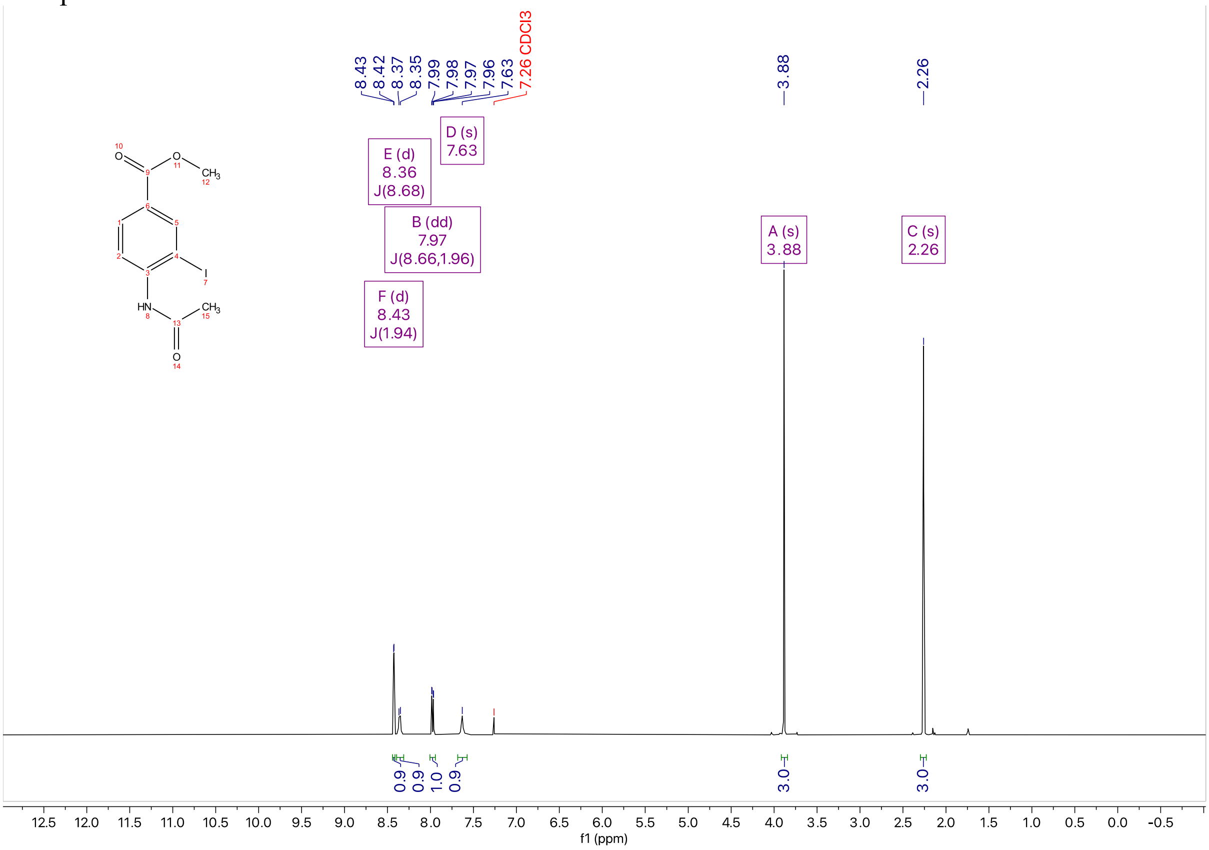

Compound S1i ^{13}C

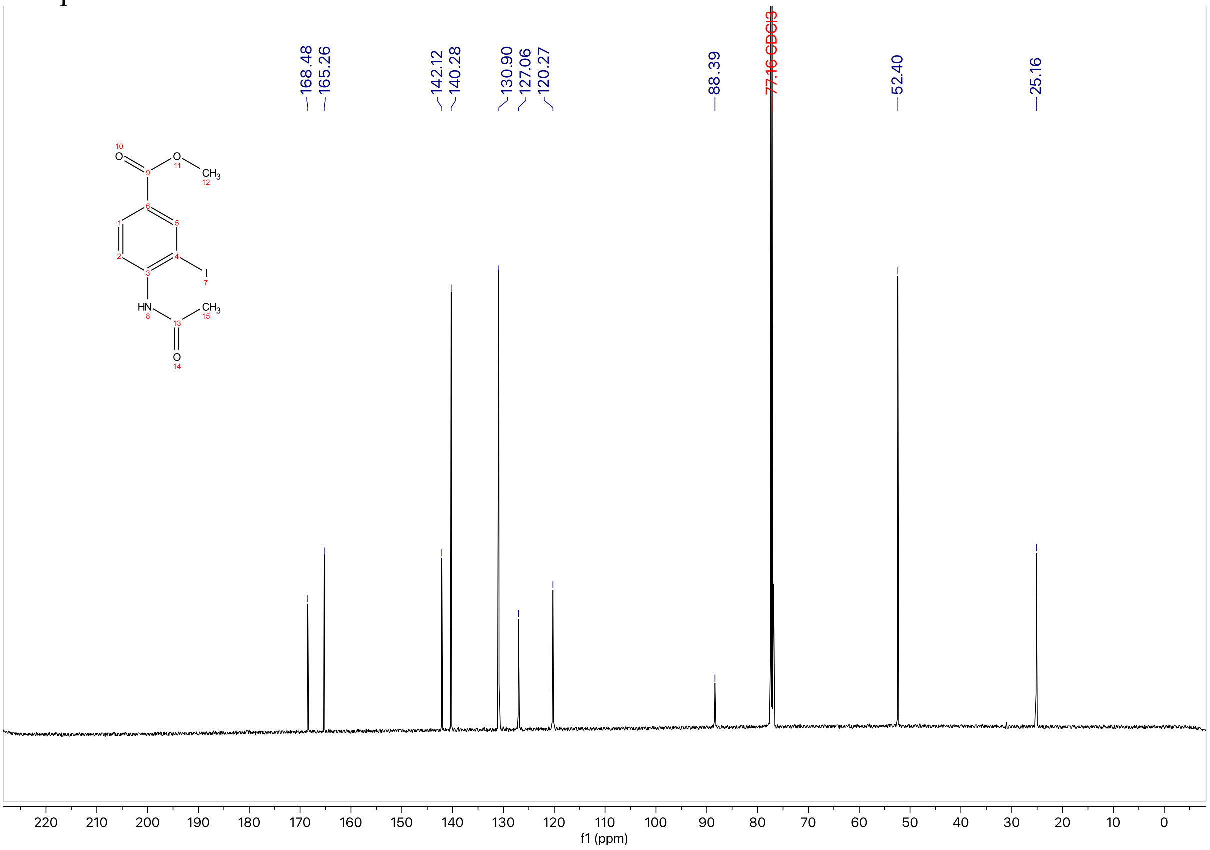

Compound S11 ^{1}H

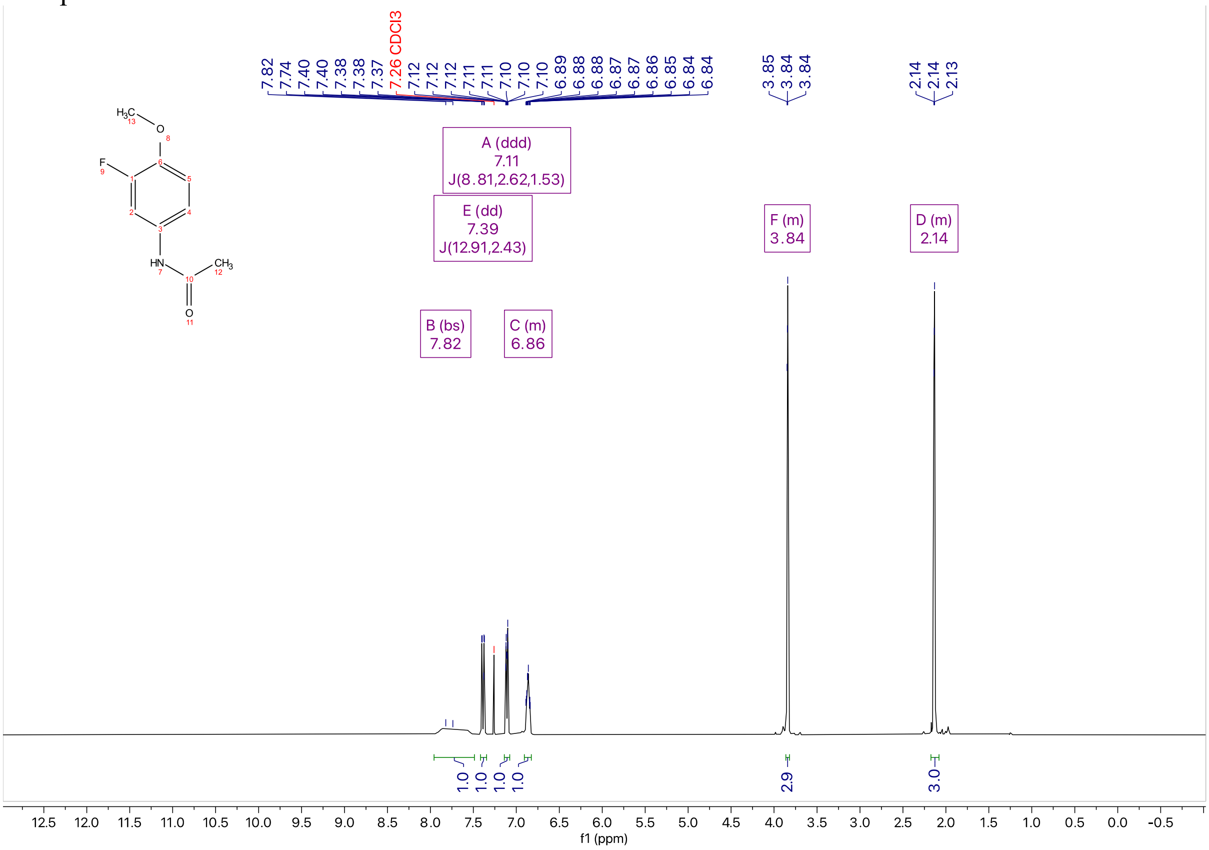

Compound S11 ^{13}C

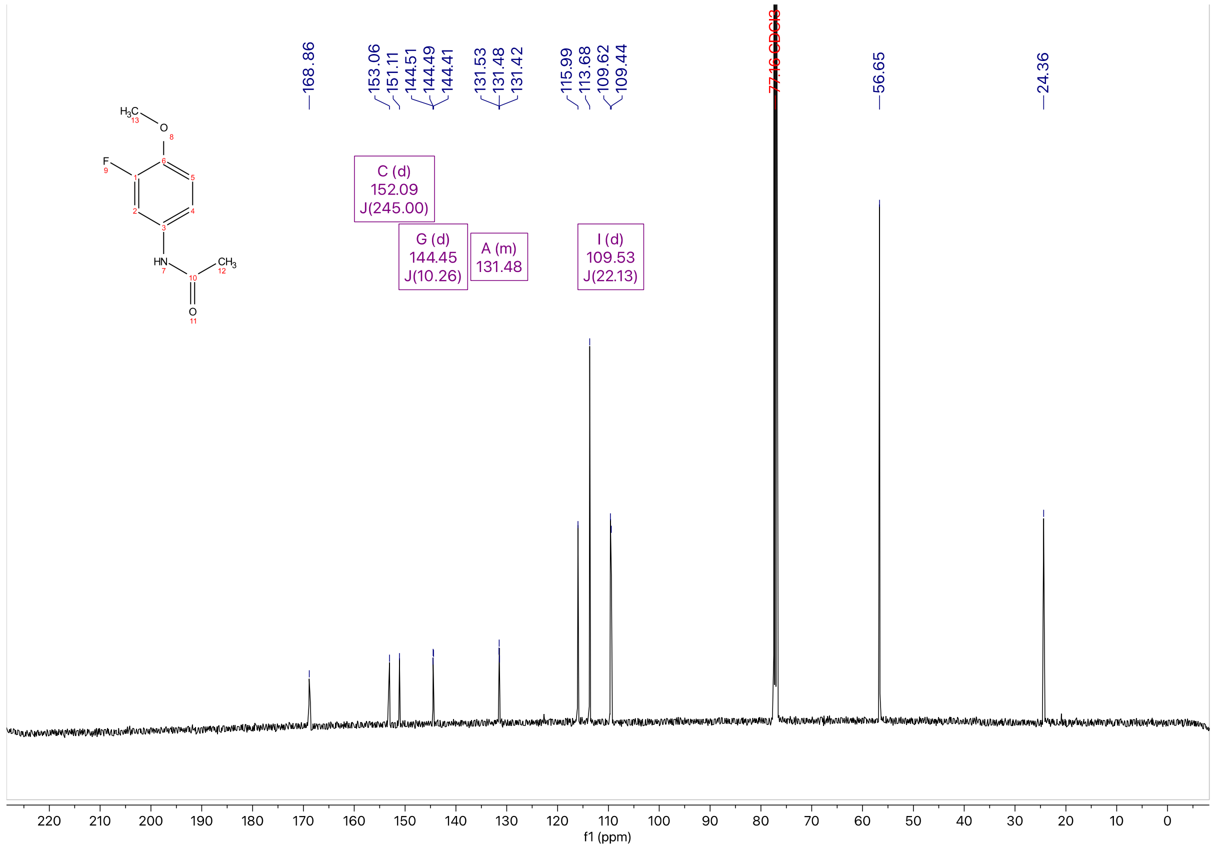

Compound S11 ^{19}F

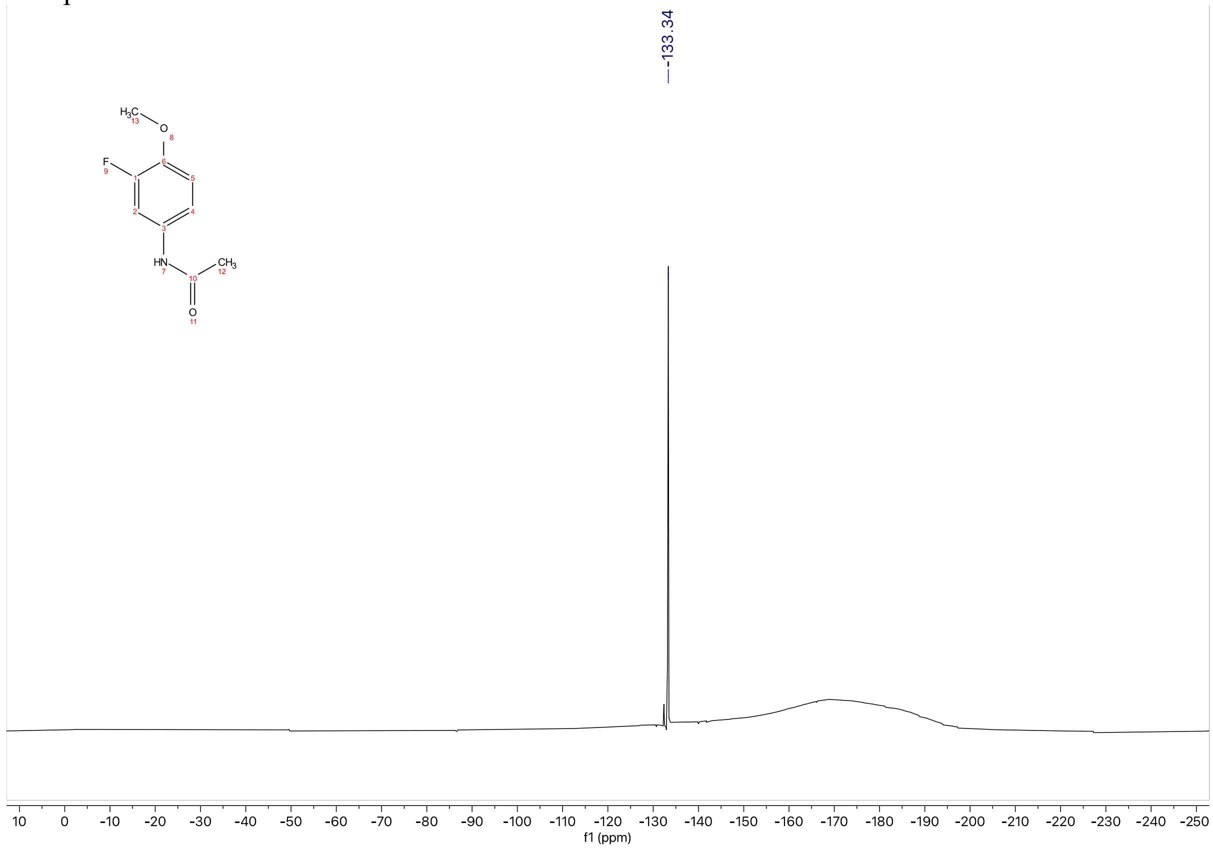

Compound S1j ^{1}H

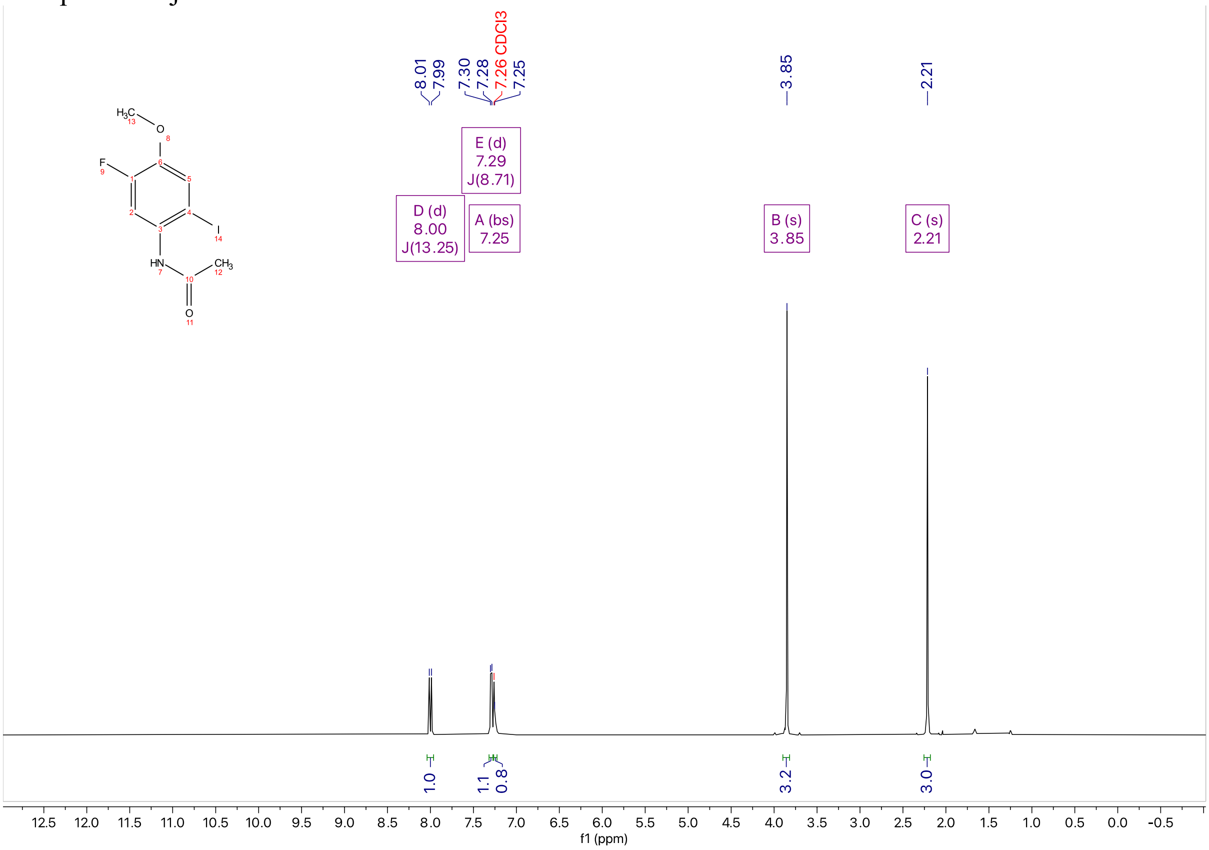

Compound S1j ^{13}C

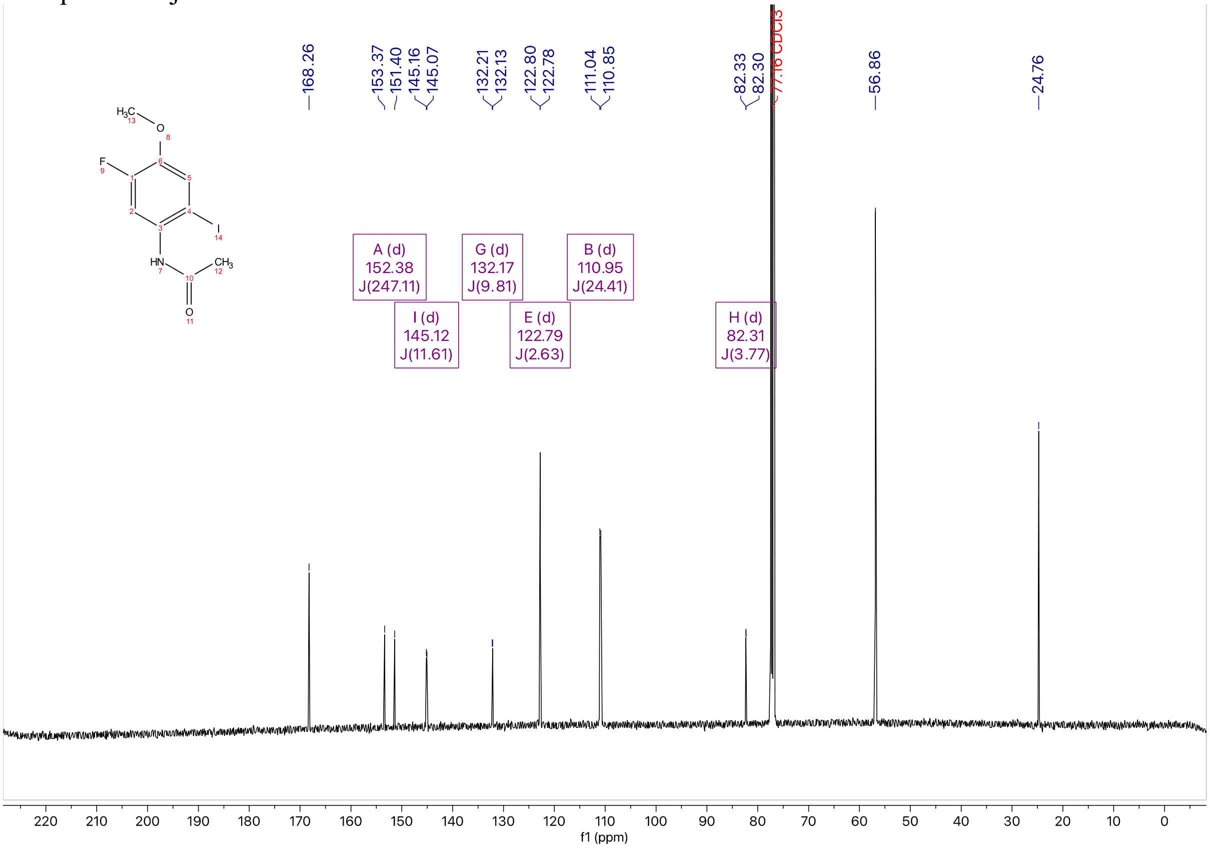

Compound S1j ^{19}F

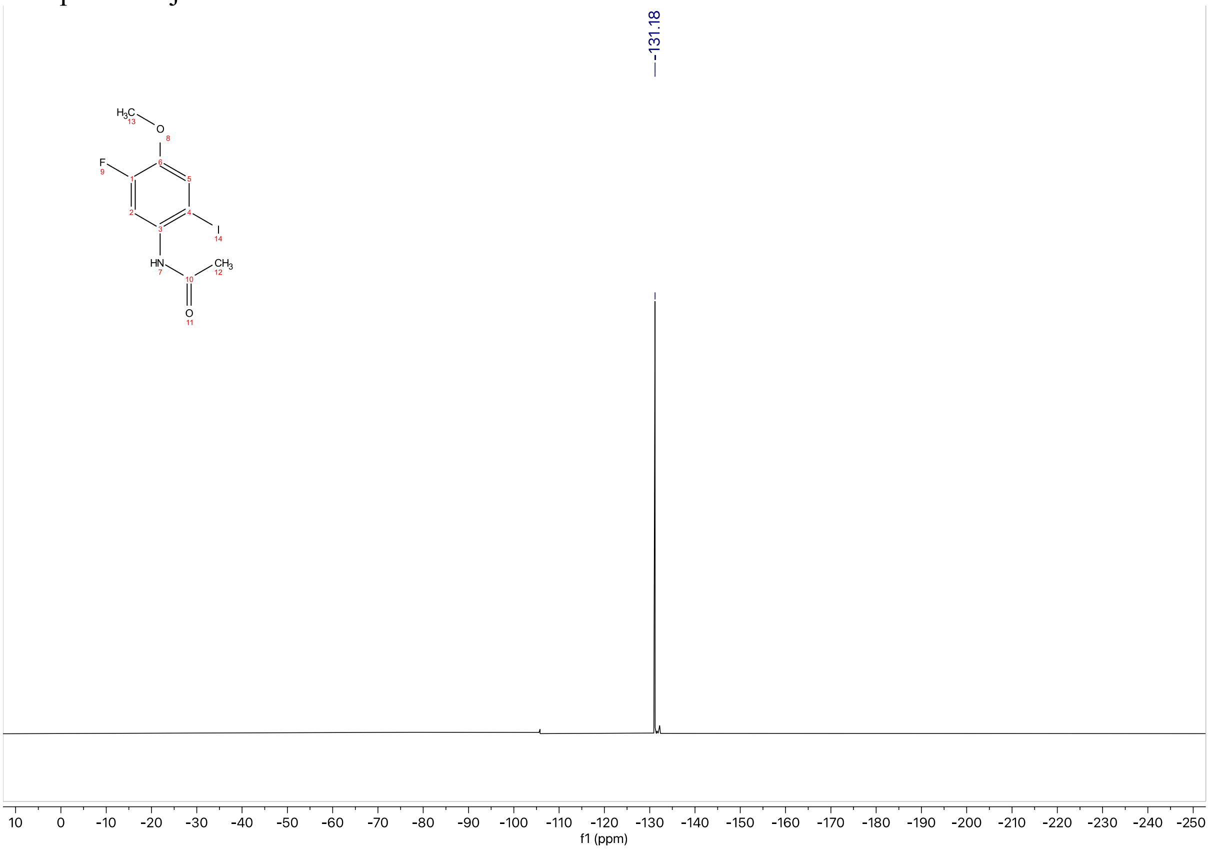

Hydroxylamine crude 1H

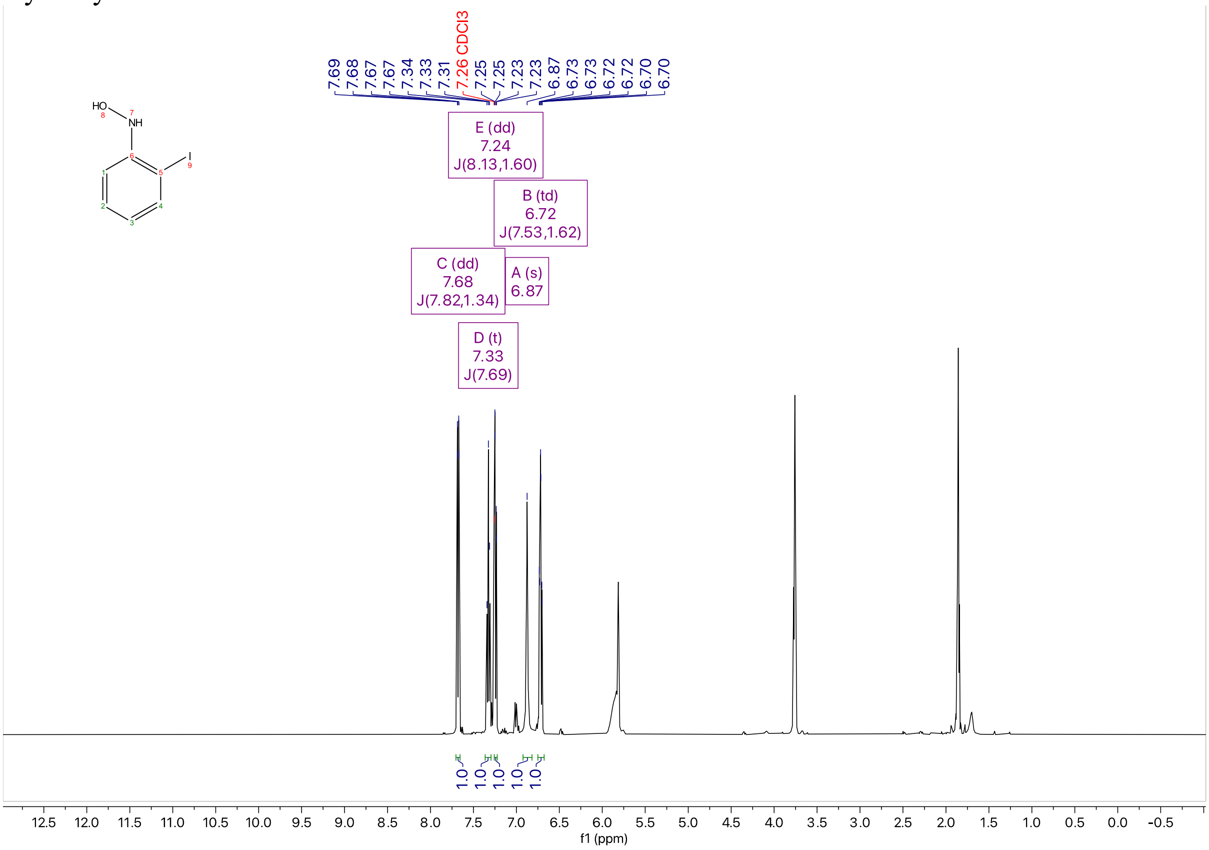

Compound S12 ^{1}H

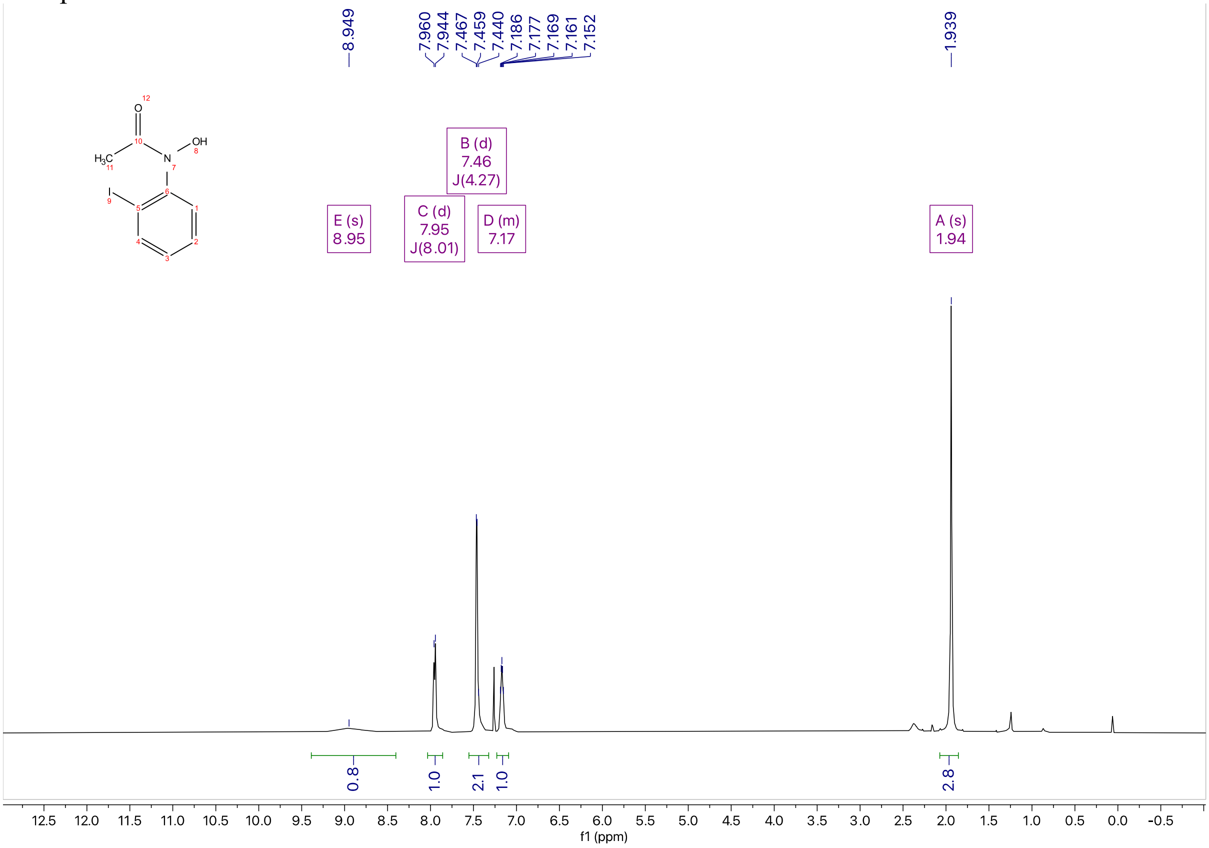

Compound S12 ^{13}C

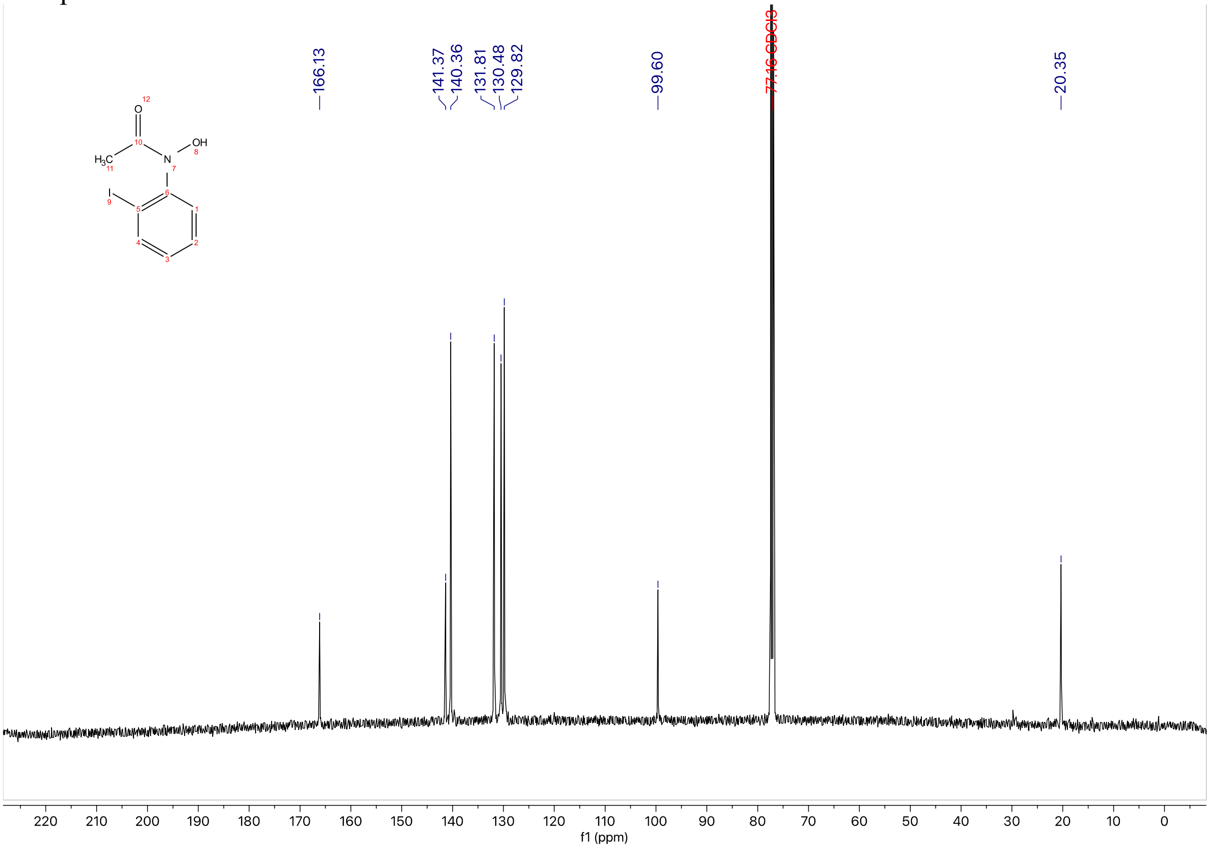

Compound 13 ^{1}H

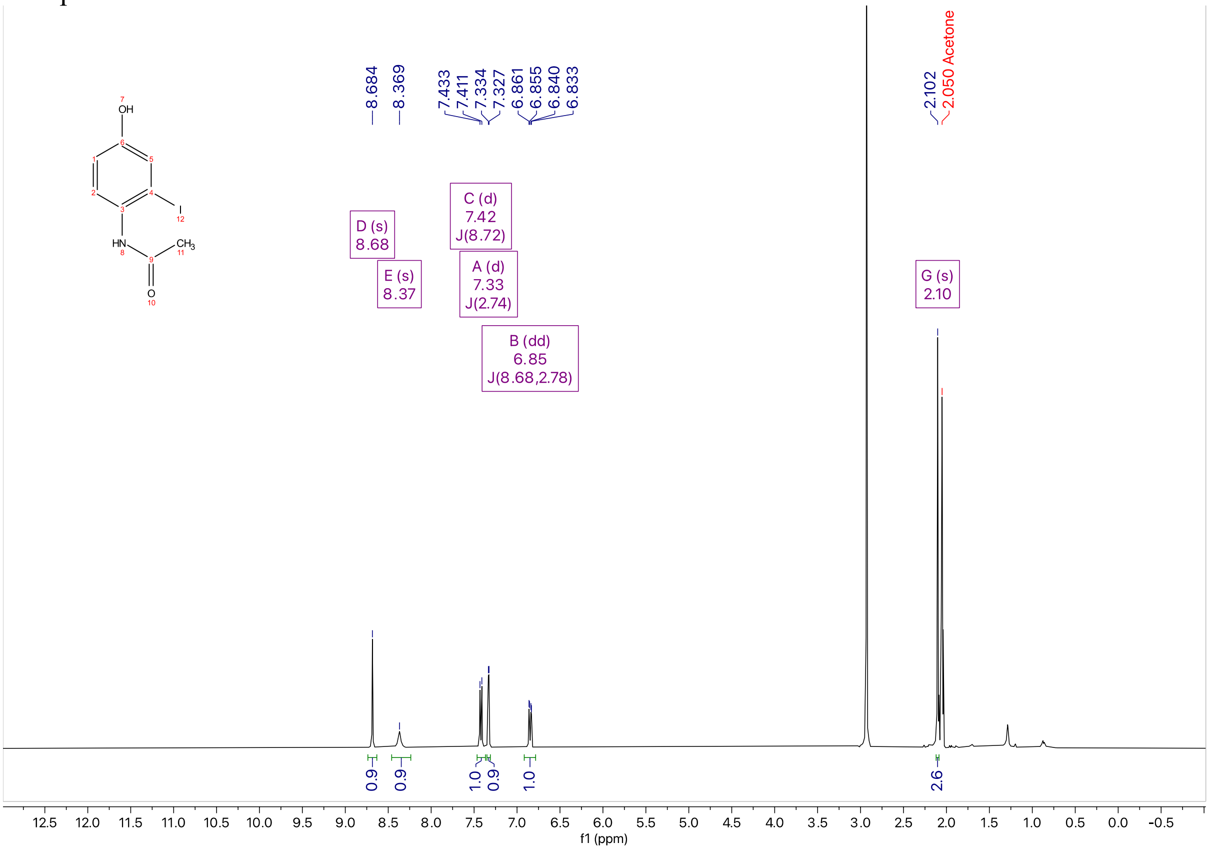

Compound S13 ^{13}C

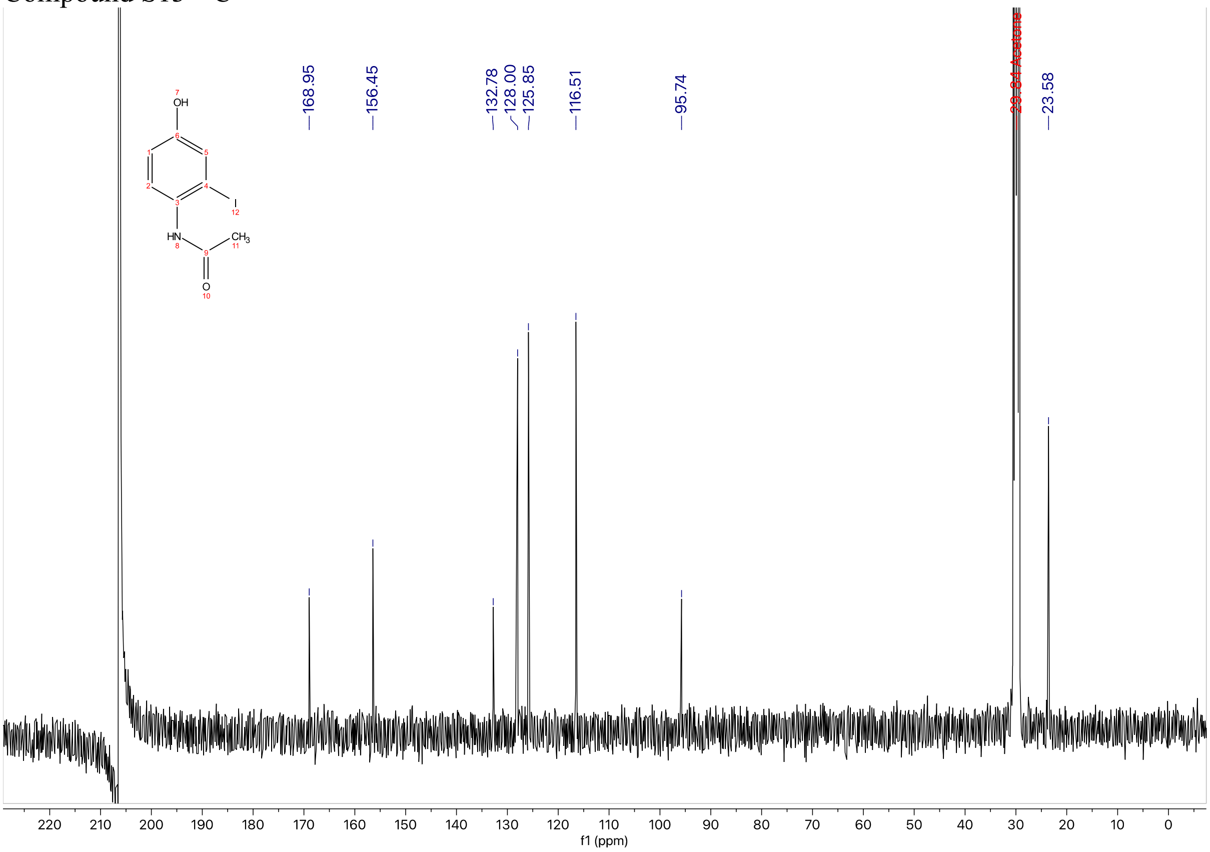

Compound S1k ^{1}H

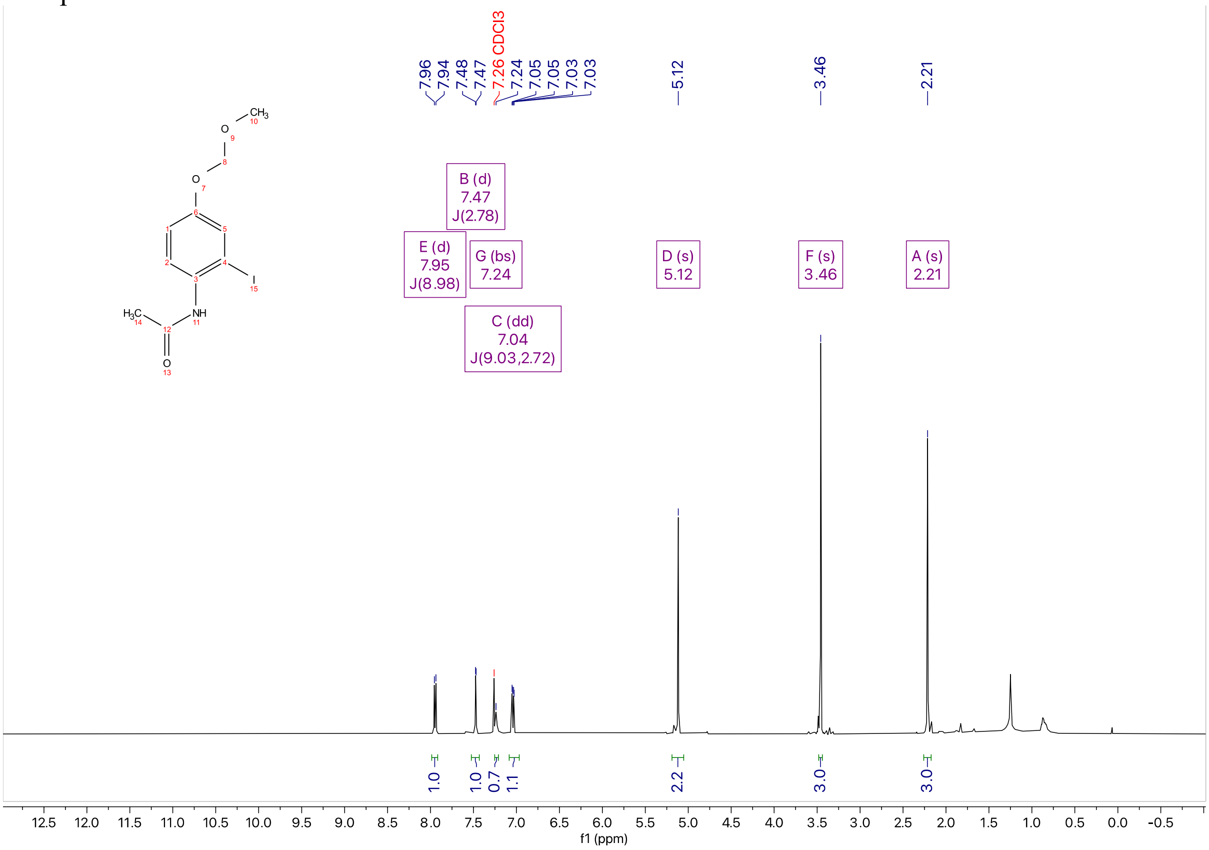

Compound S1k ^{13}C

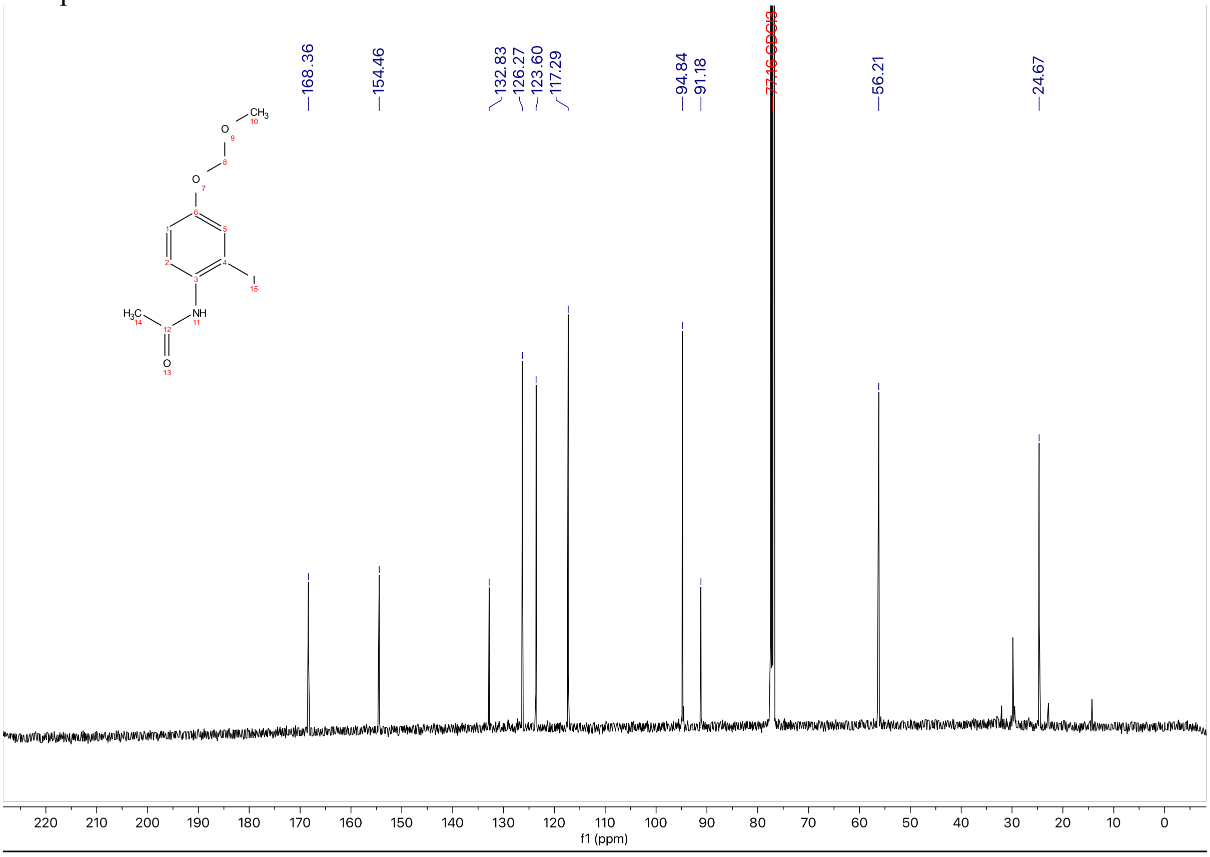

Compound S14 ^{1}H

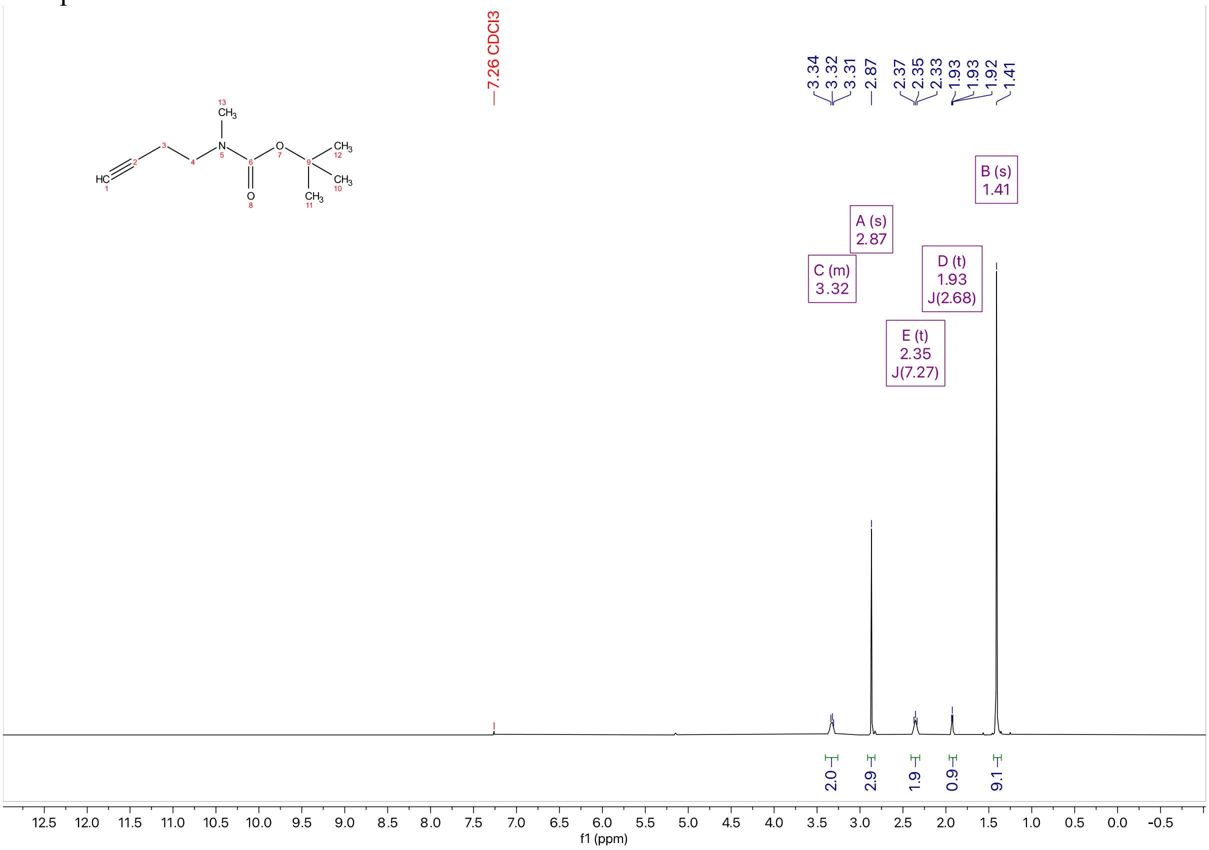

Compound S14 ^{13}C

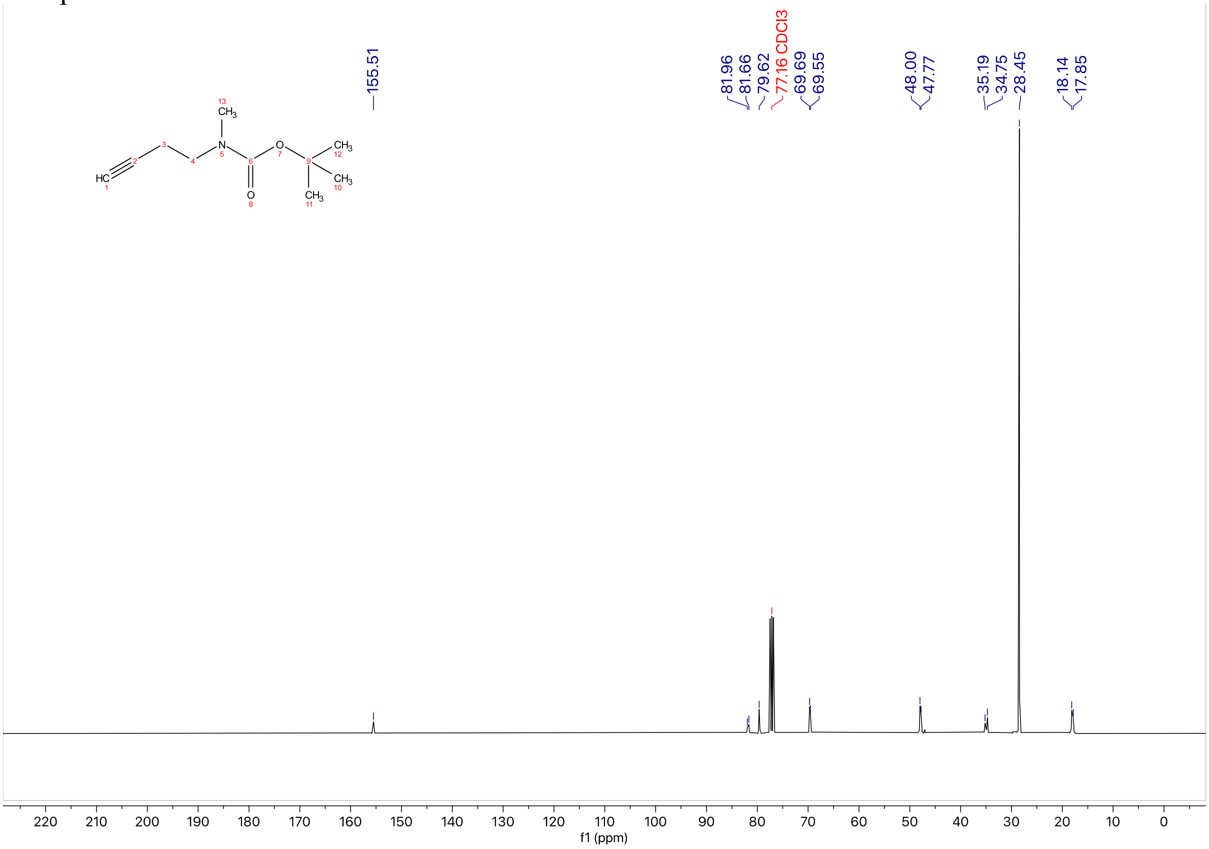

Compound S2b ^{1}H

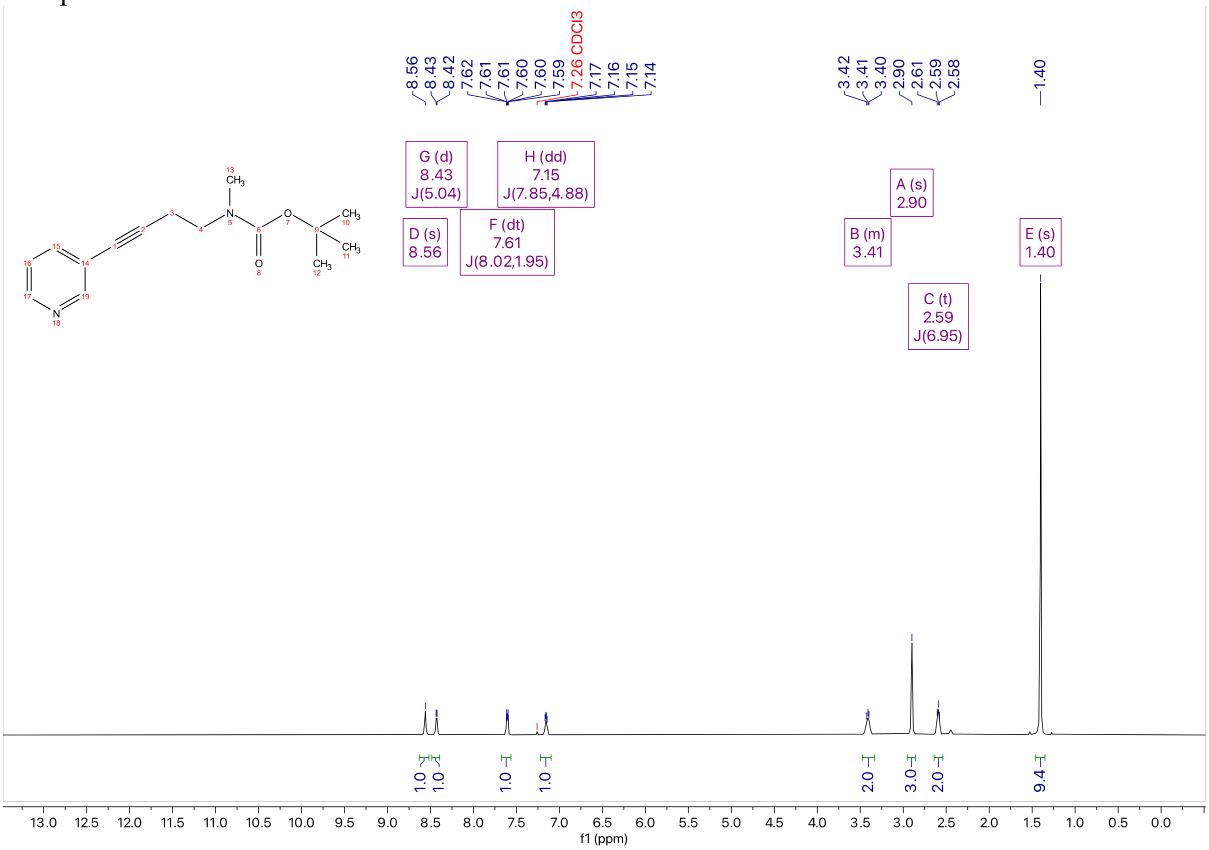

Compound S2b ^{13}C

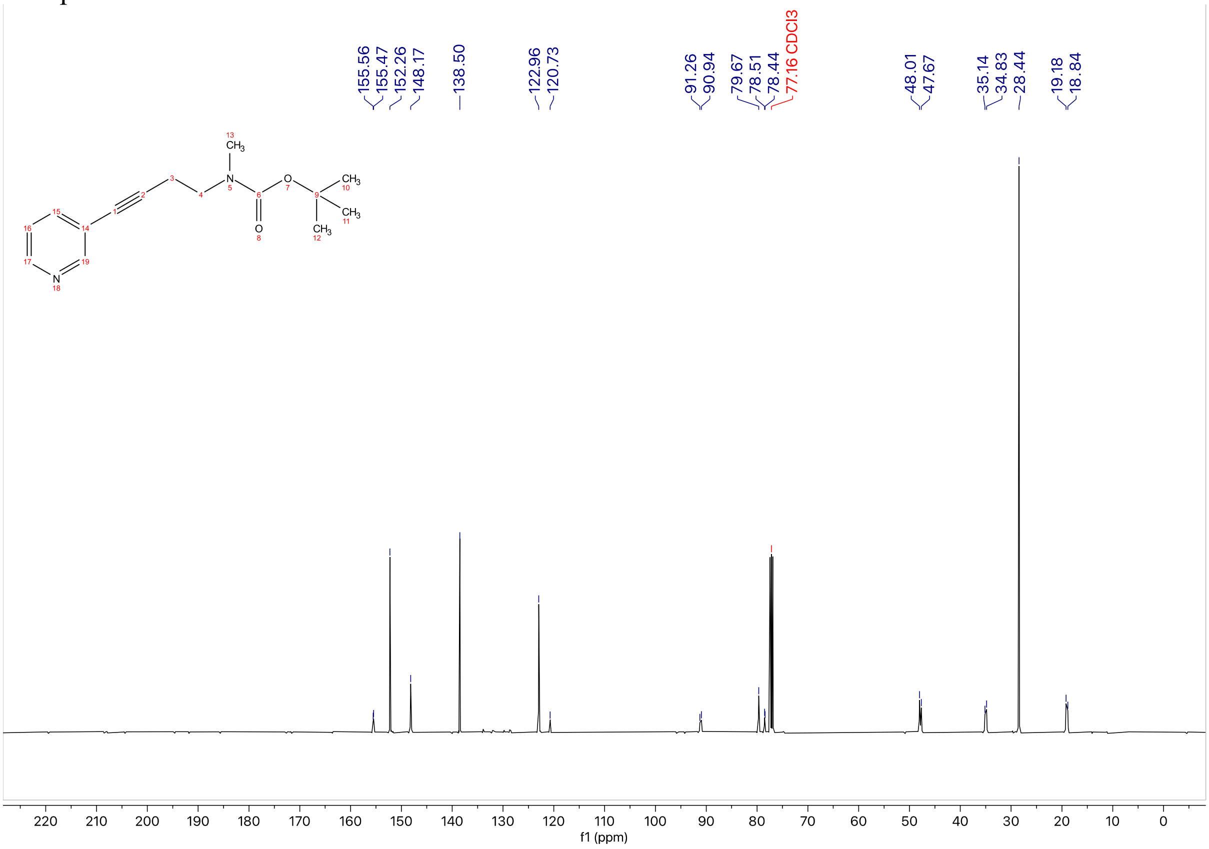

Compound S2c ^{1}H

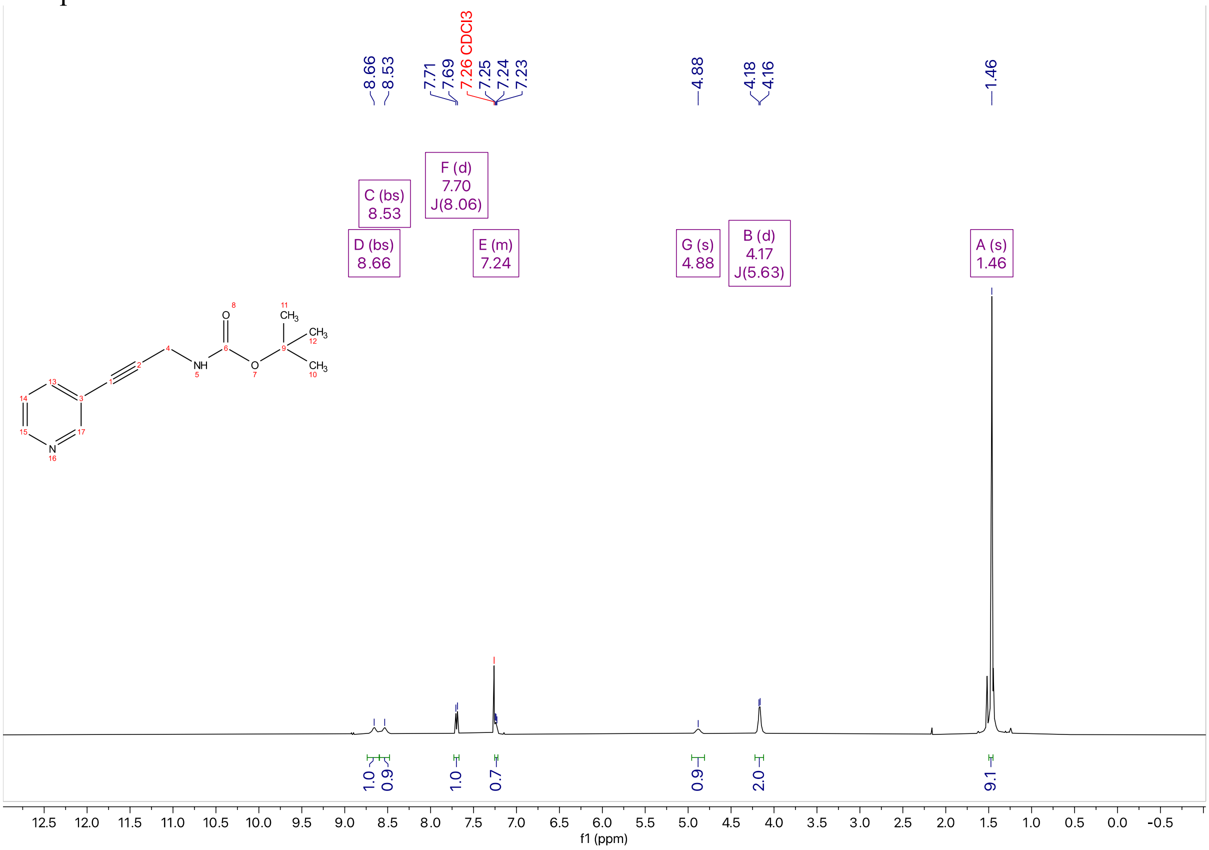

Compound S2c ^{13}C

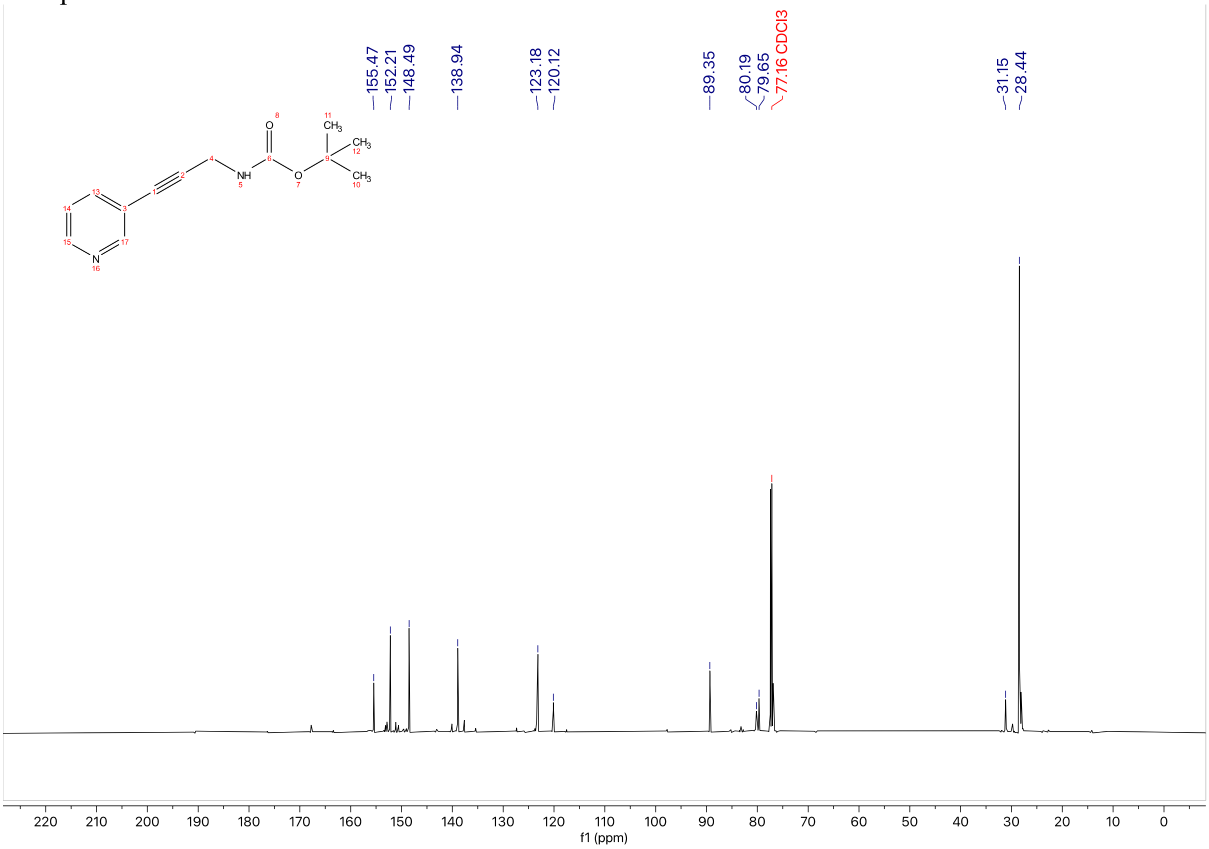

Compound S15 ^{1}H

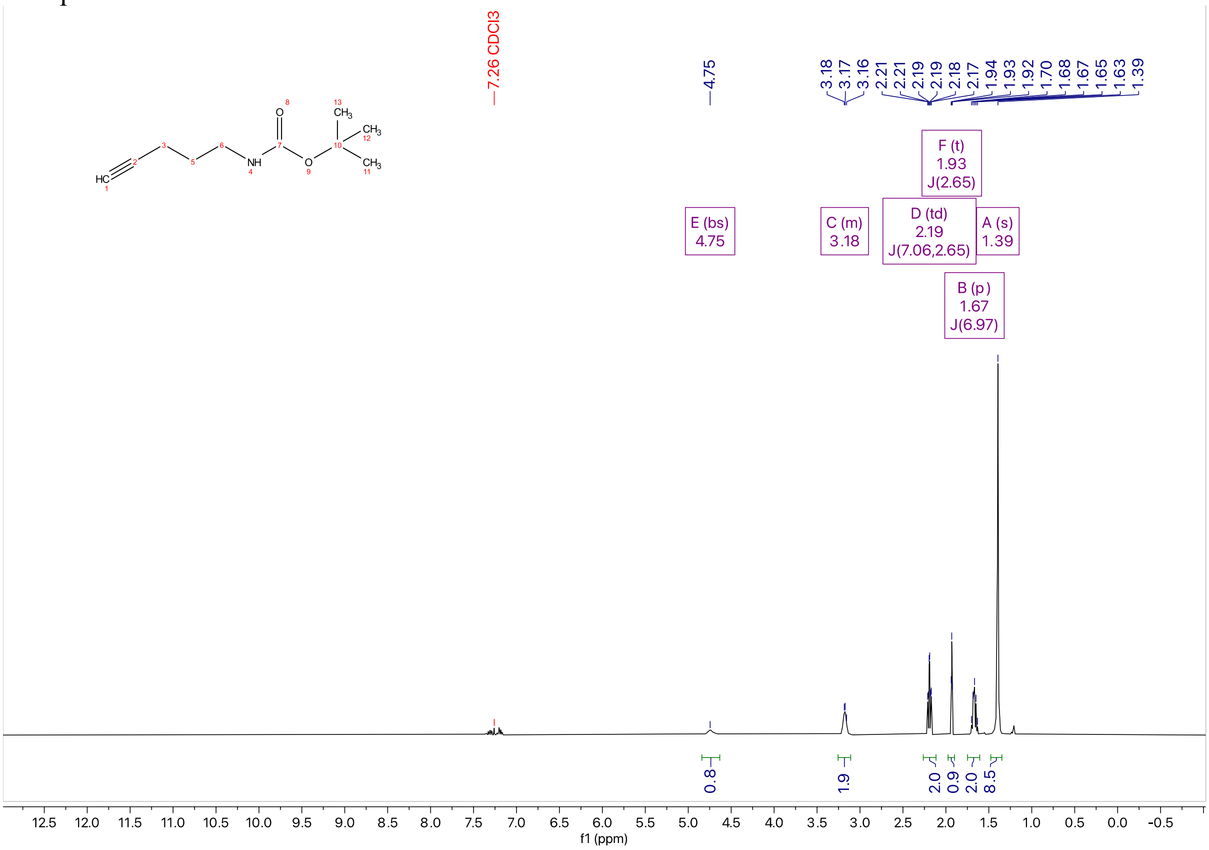

Compound S15 ^{13}C

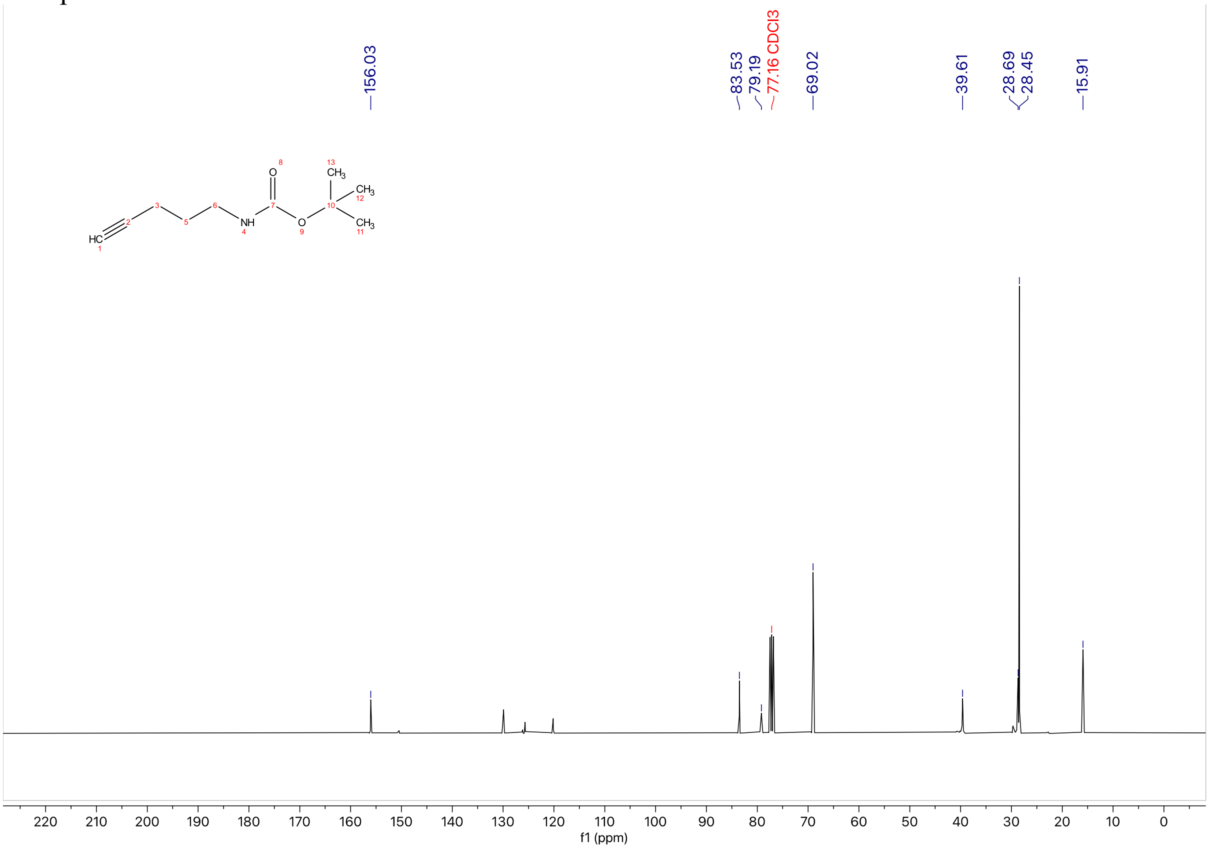

Compound S2d ^{1}H

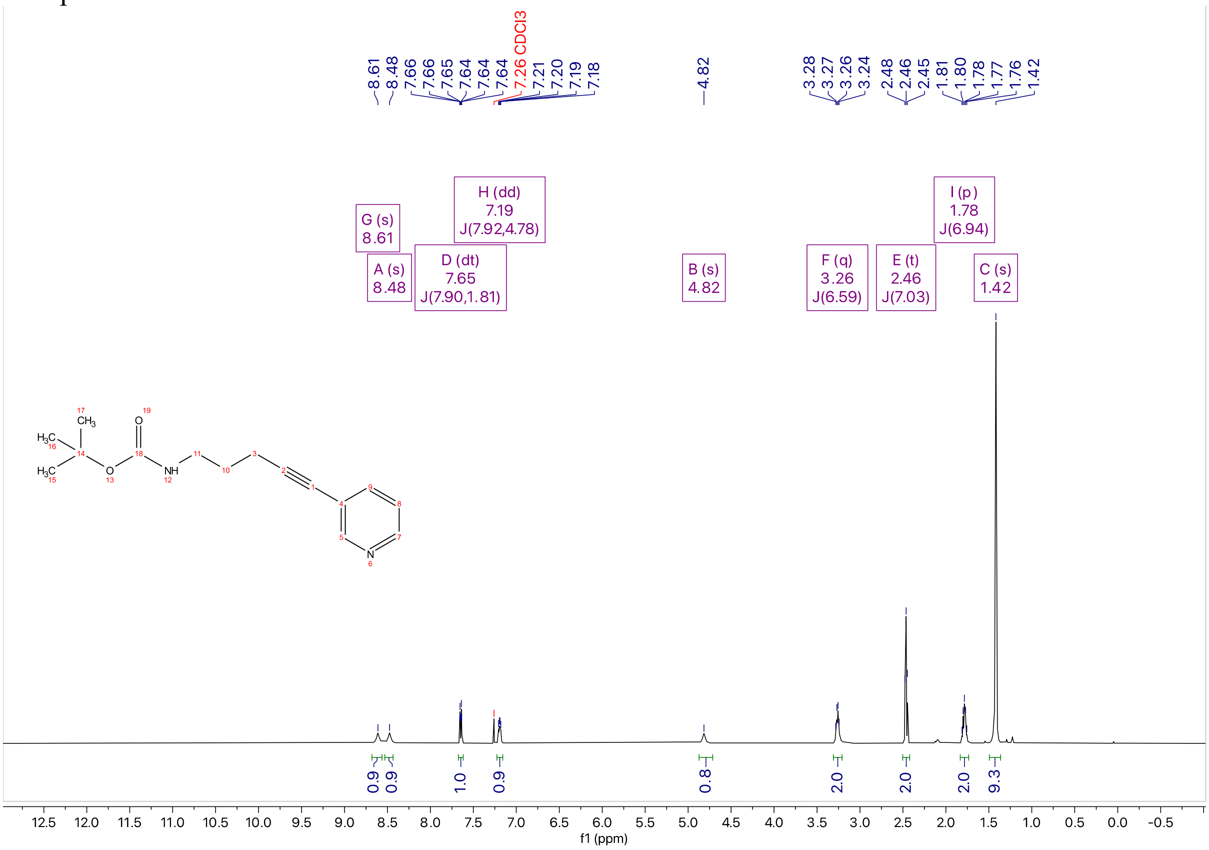

Compound S2d ^{13}C

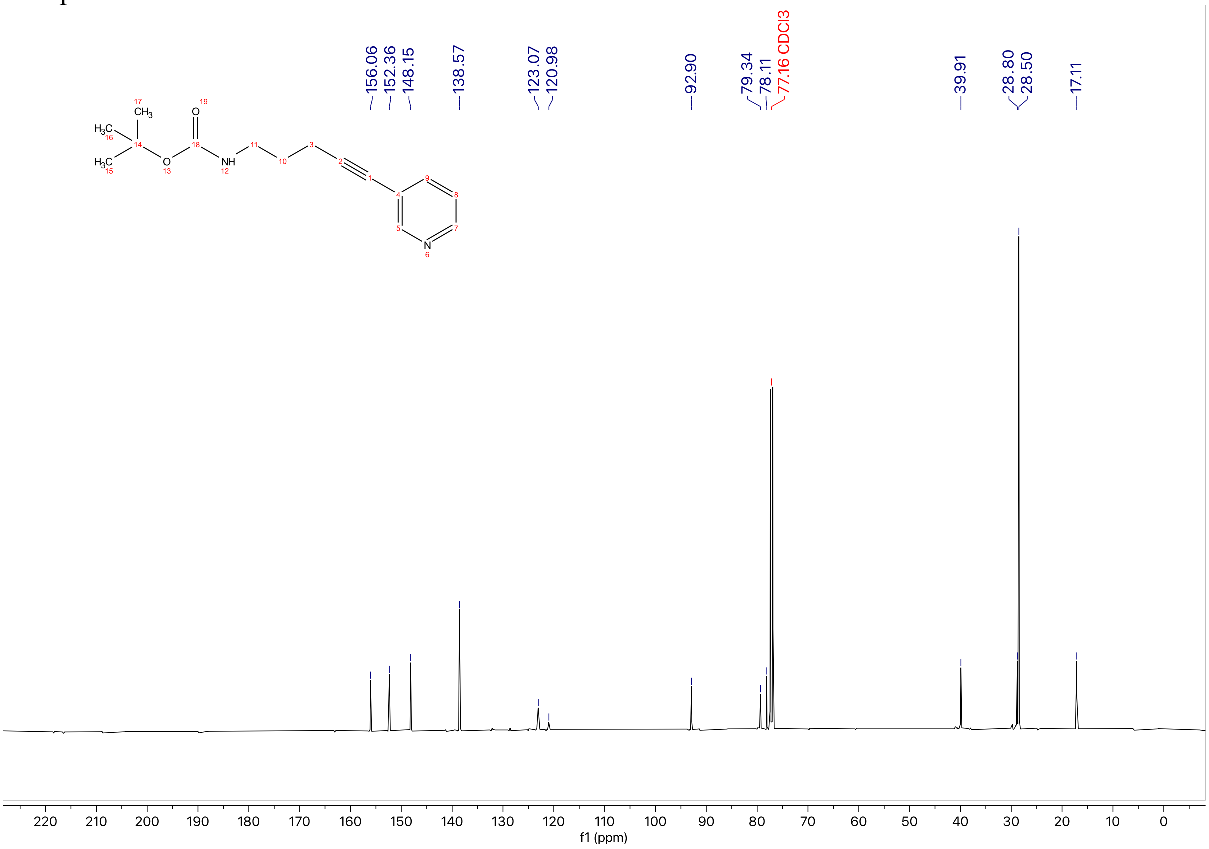

Compound S2e ^{1}H

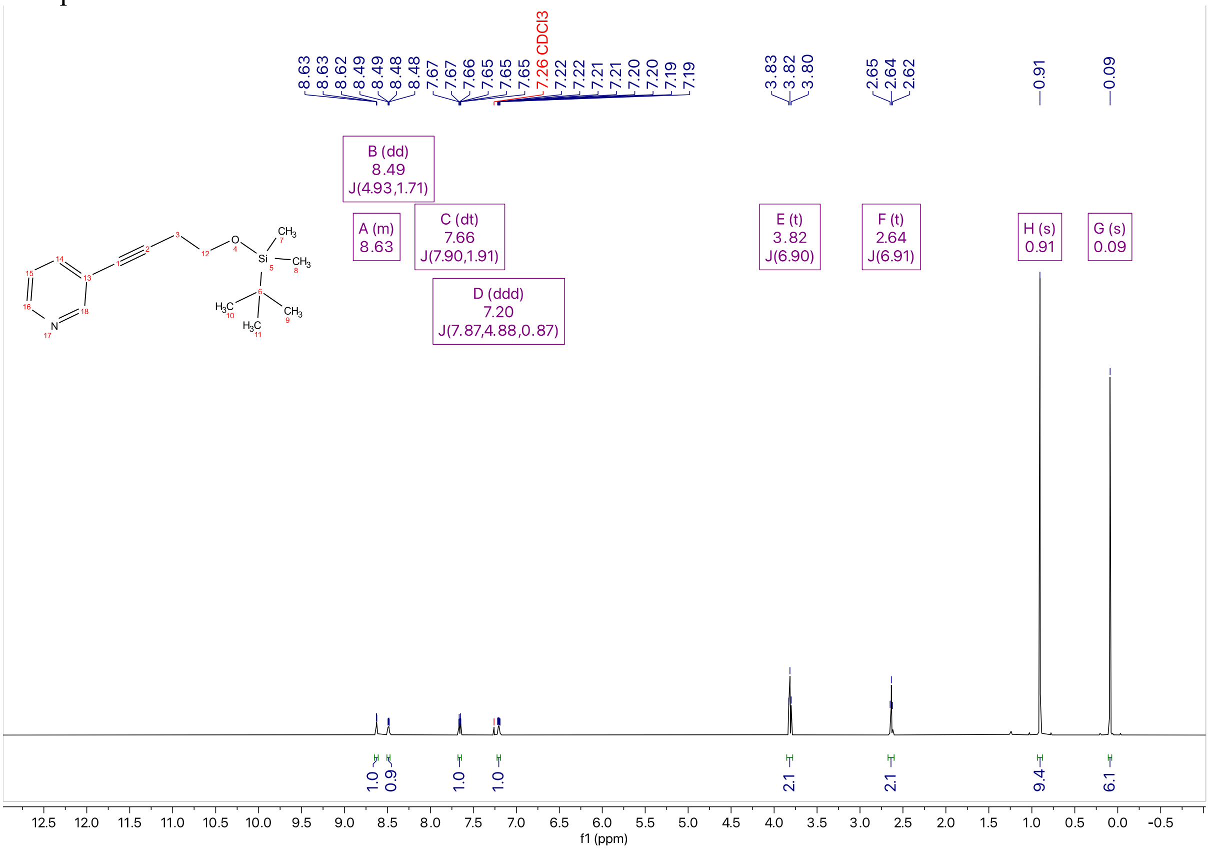

Compound S2e ^{13}C

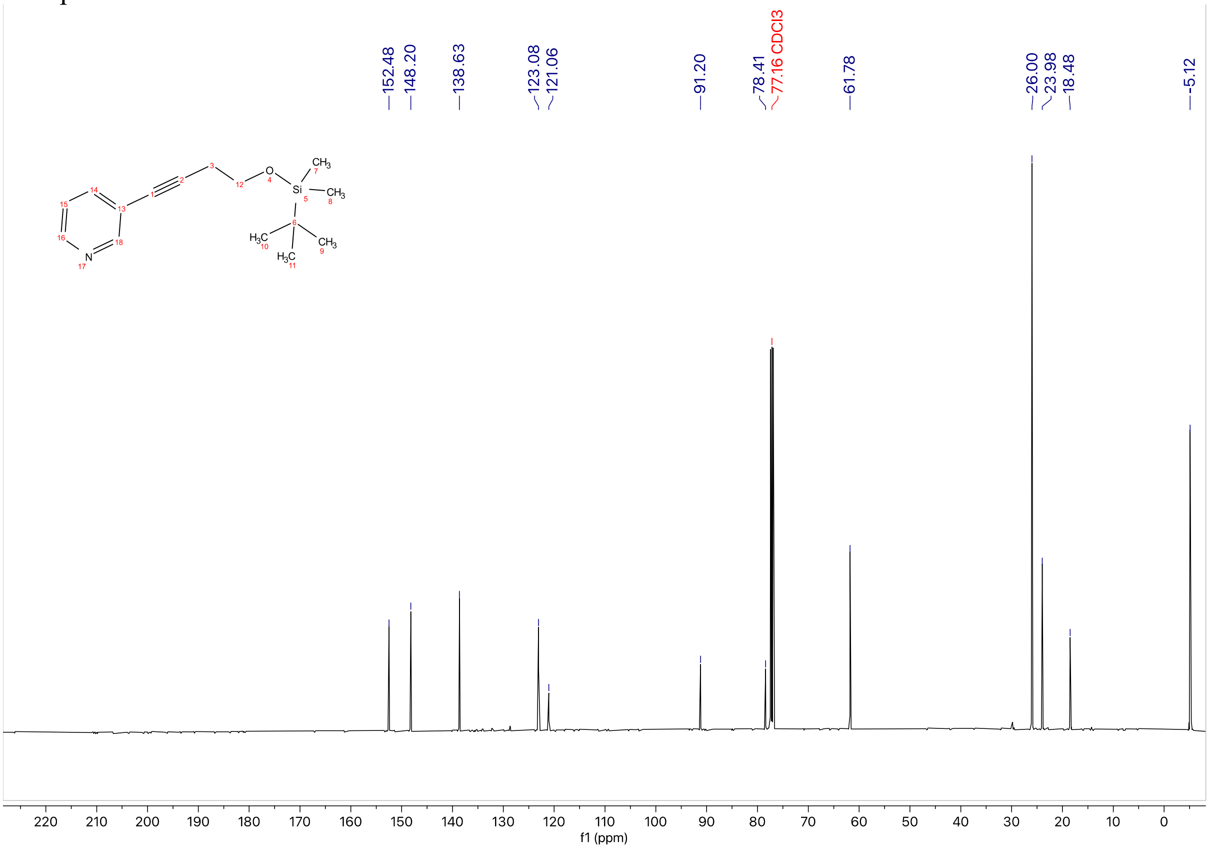

Compound S2f ^{1}H

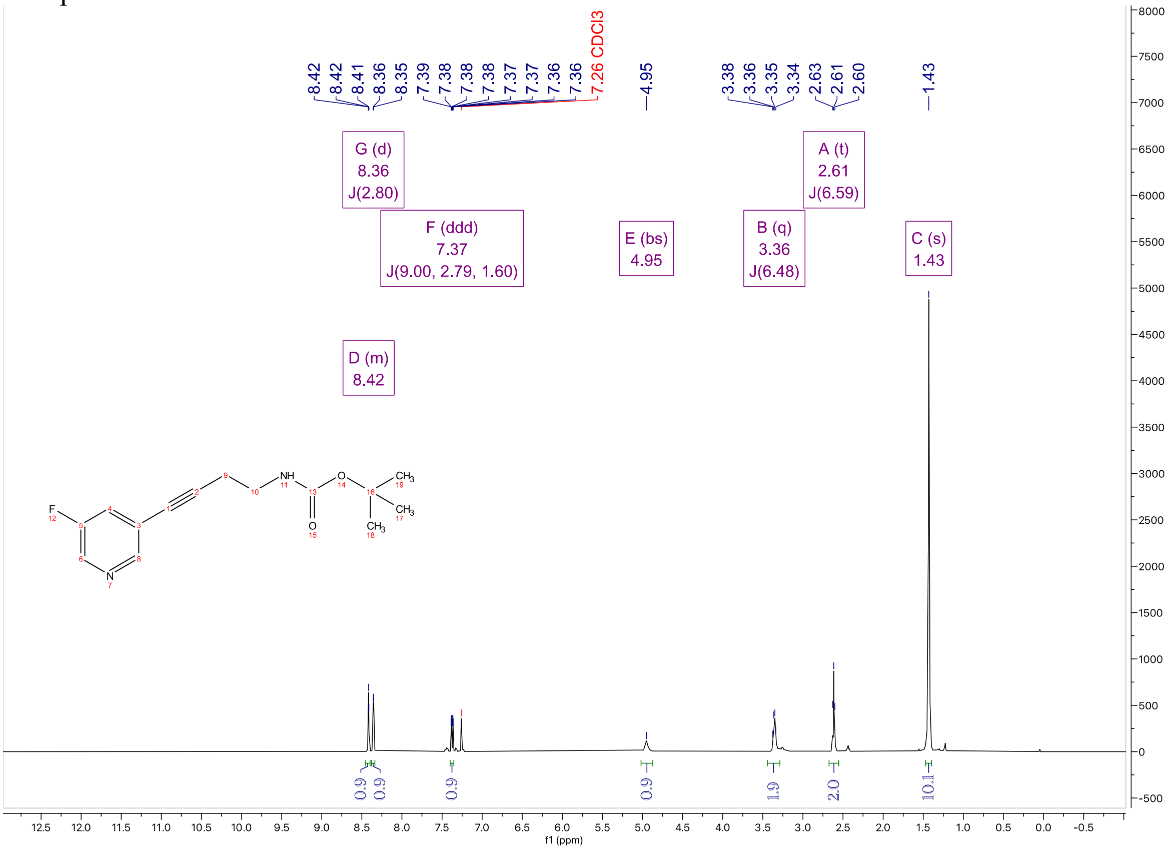

Compound S2f ^{13}C

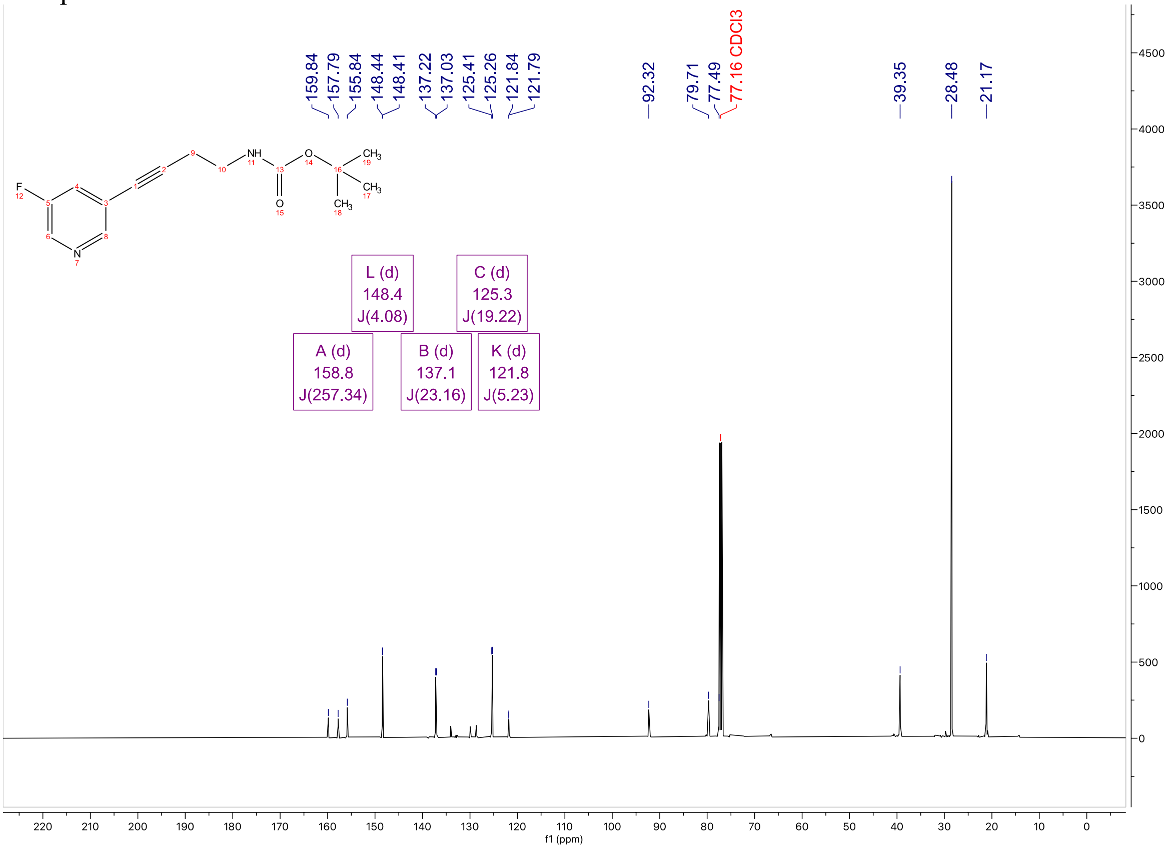

Compound S2f ^{19}F

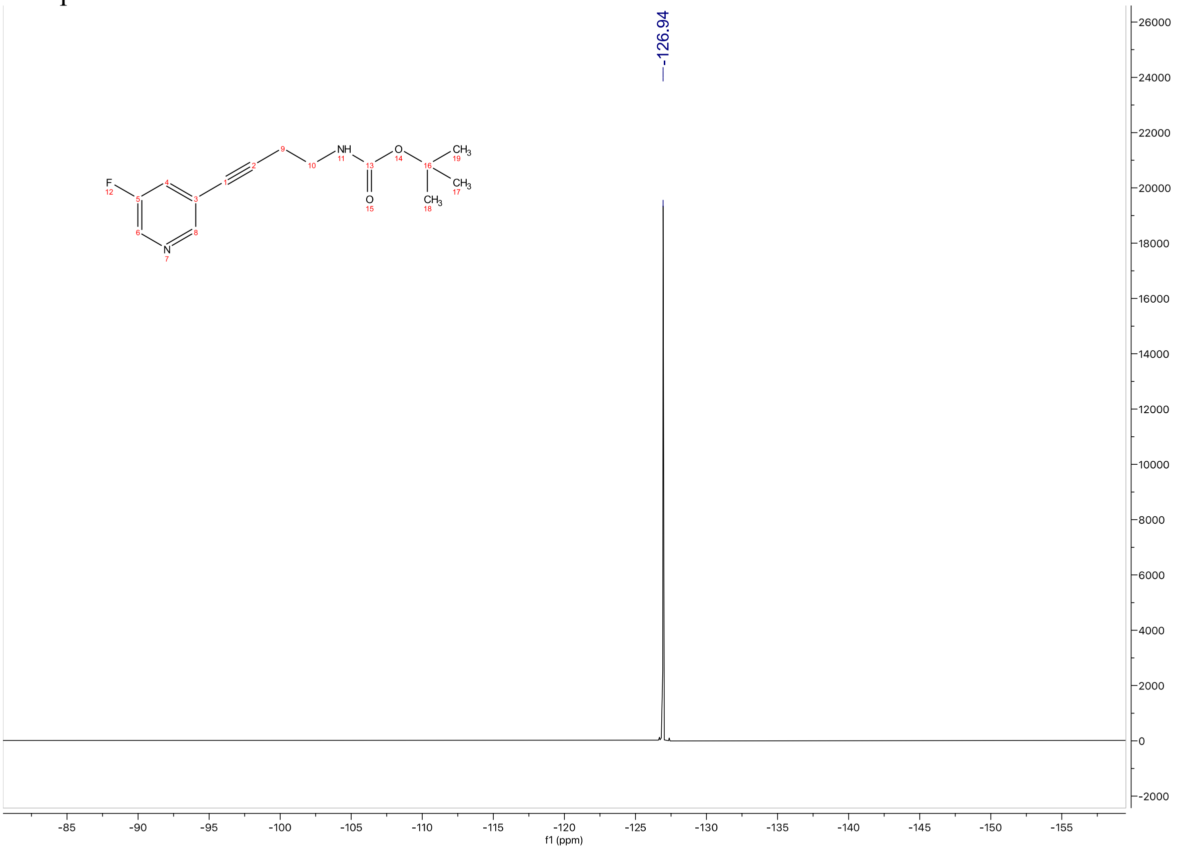

Compound S2g ^{1}H

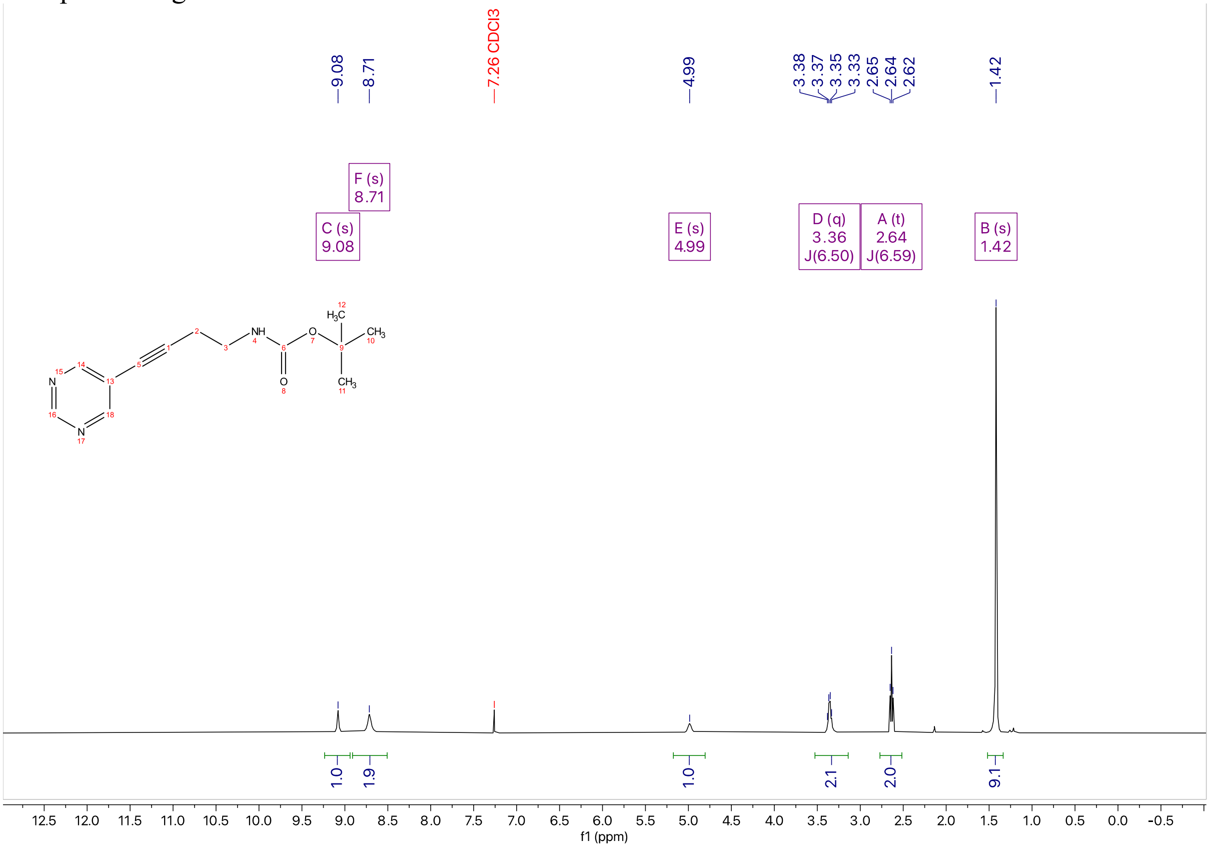

Compound S2g ^{13}C

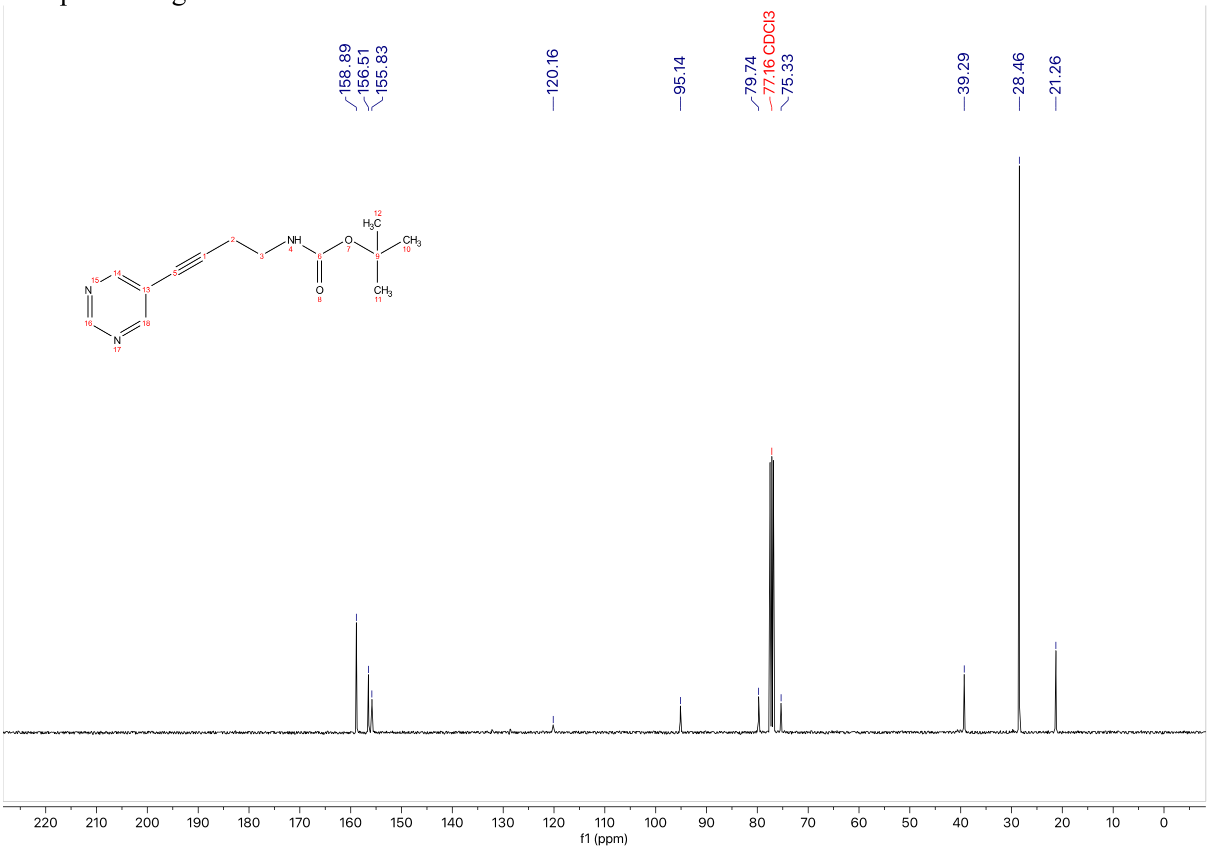

Compound S2h ^{1}H

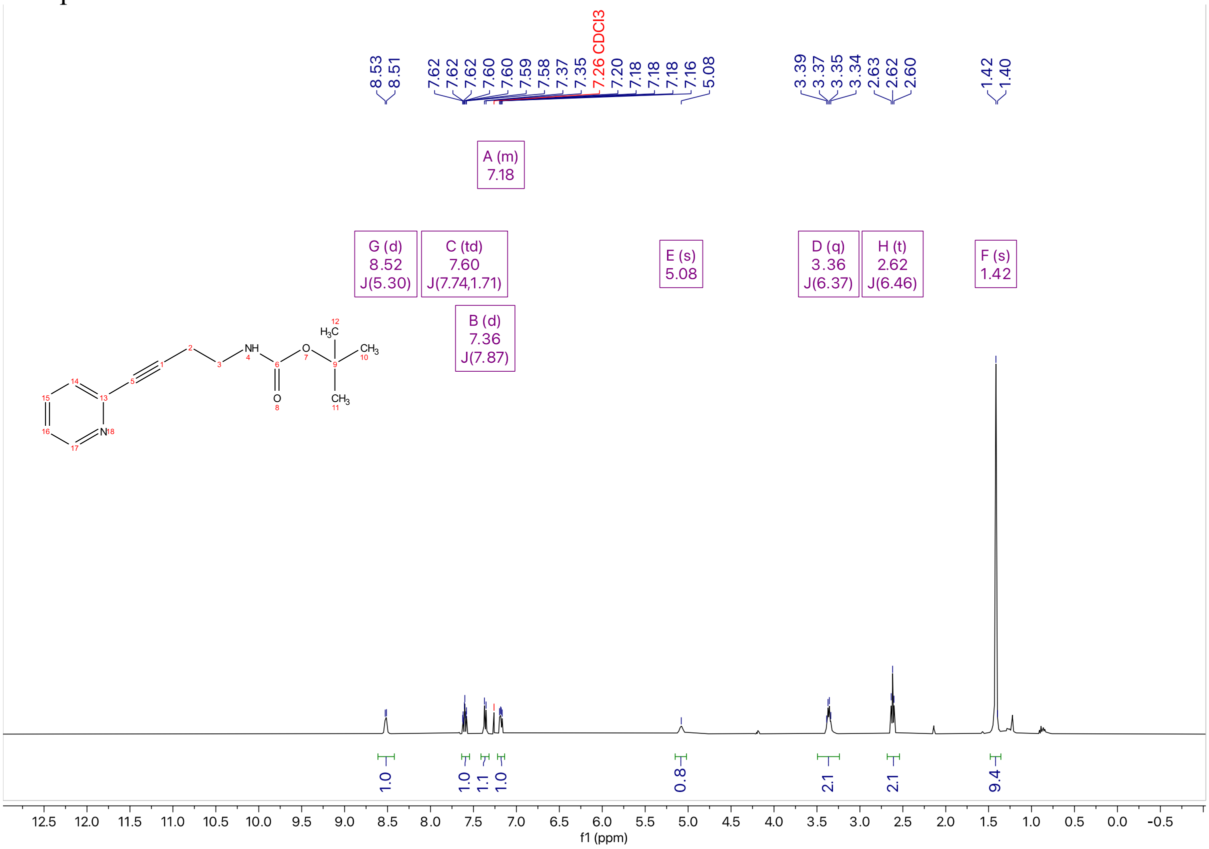

Compound S2h ^{13}C

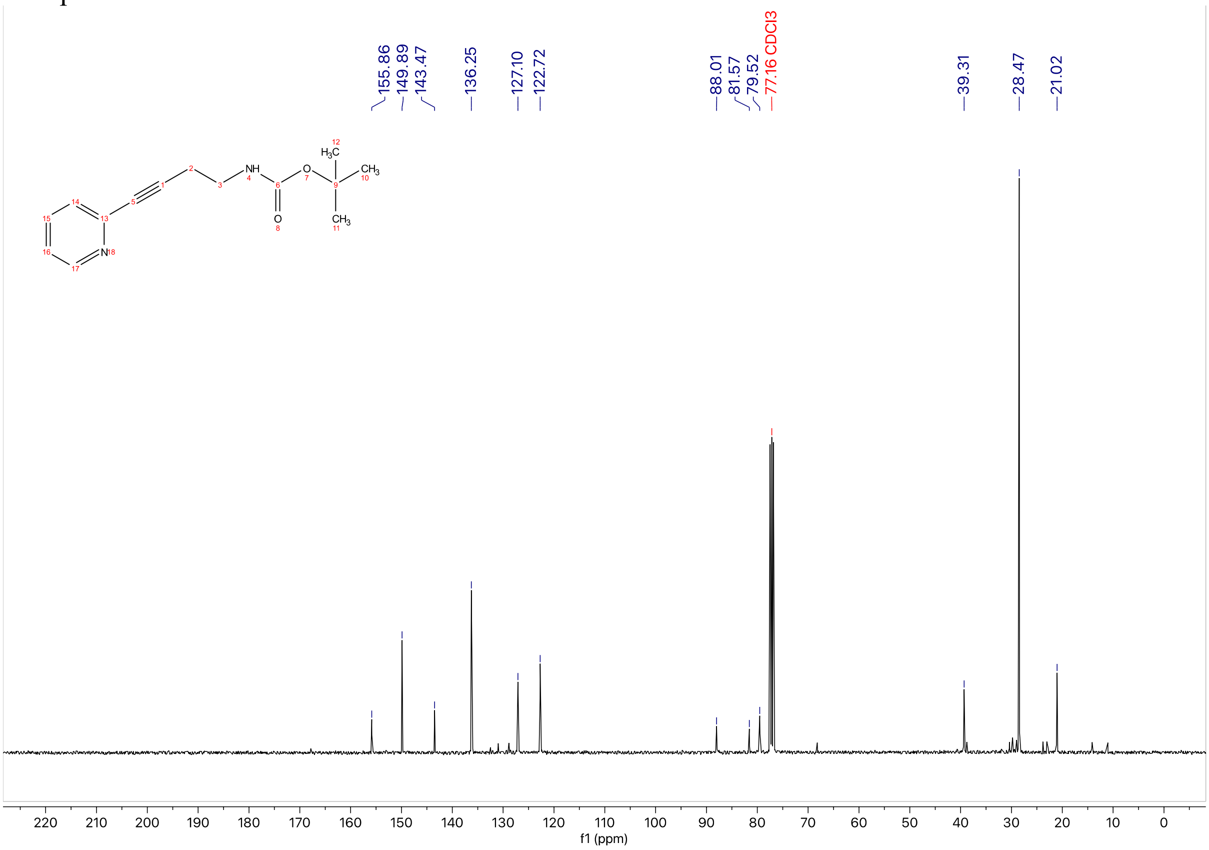

Compound S2i ^{1}H

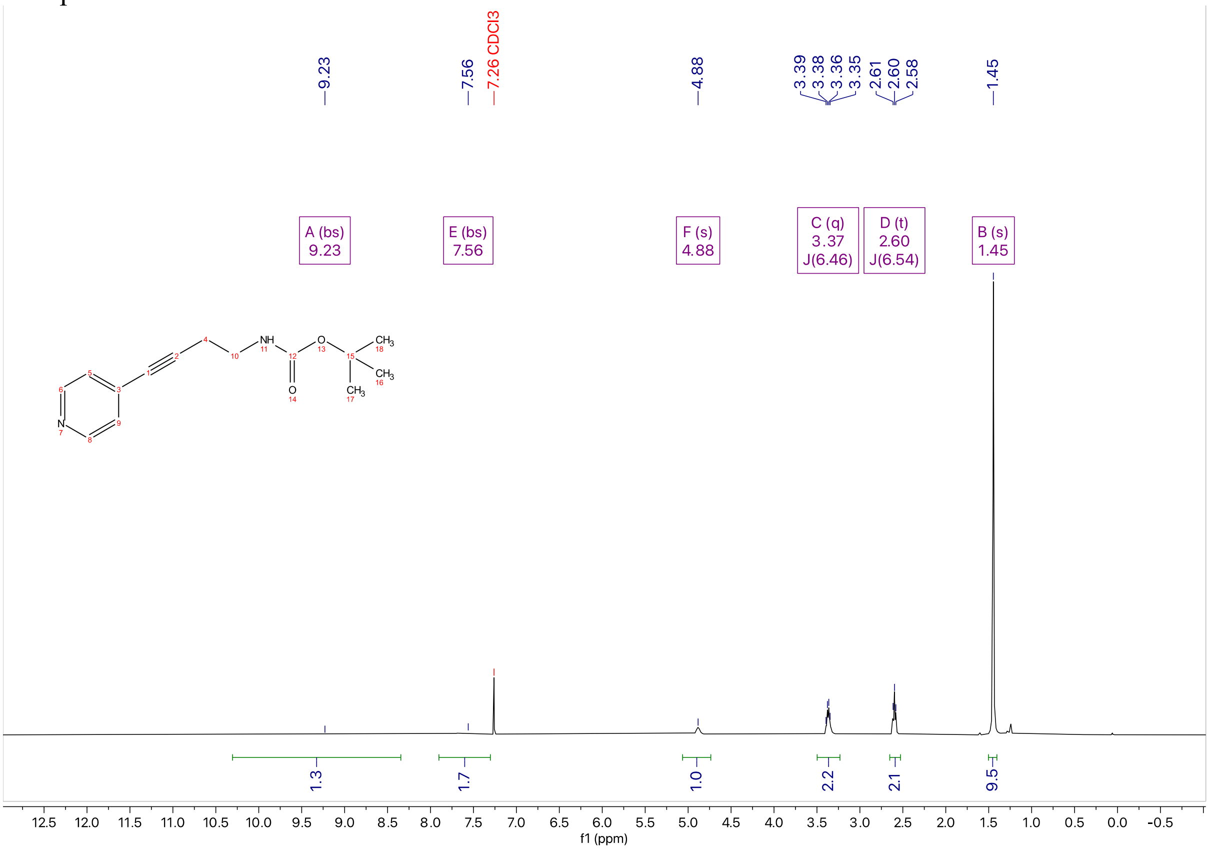

Compound S2i ^{13}C

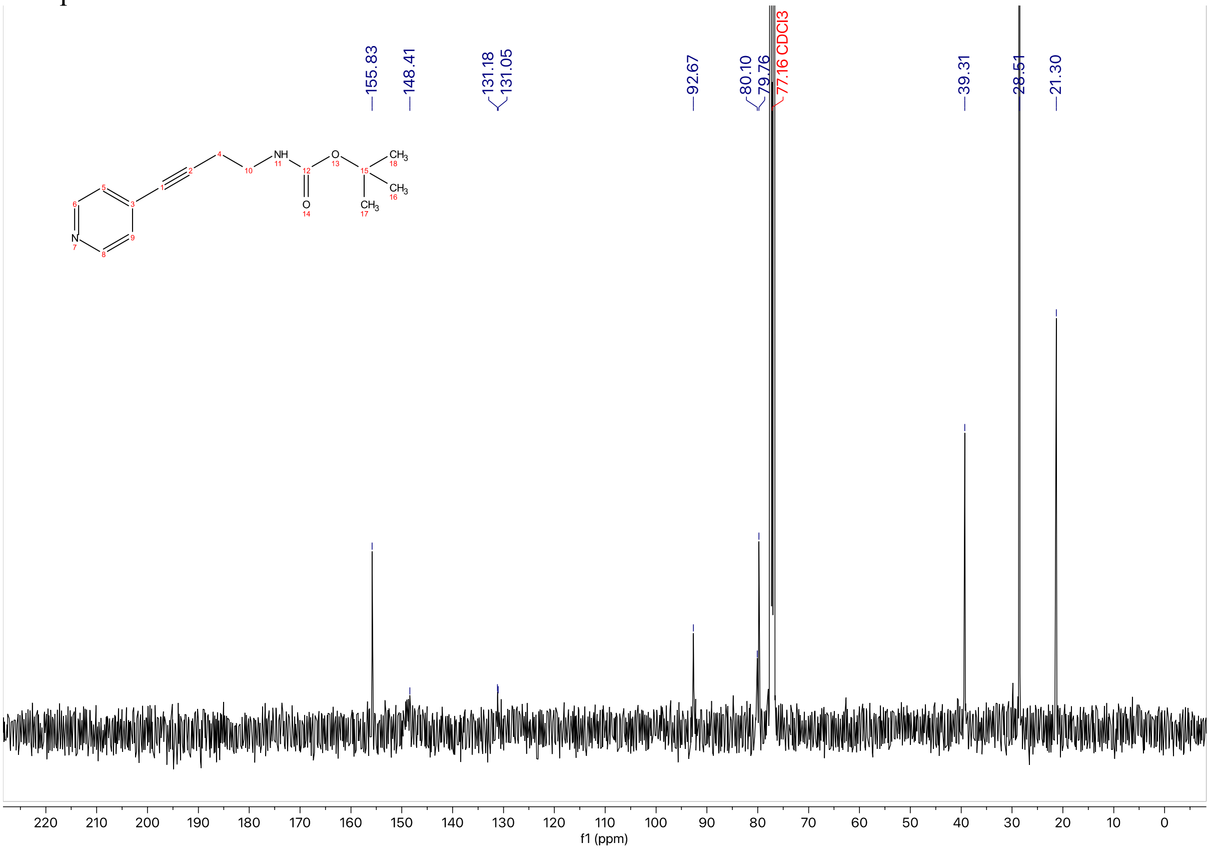

Compound S2j ^{1}H

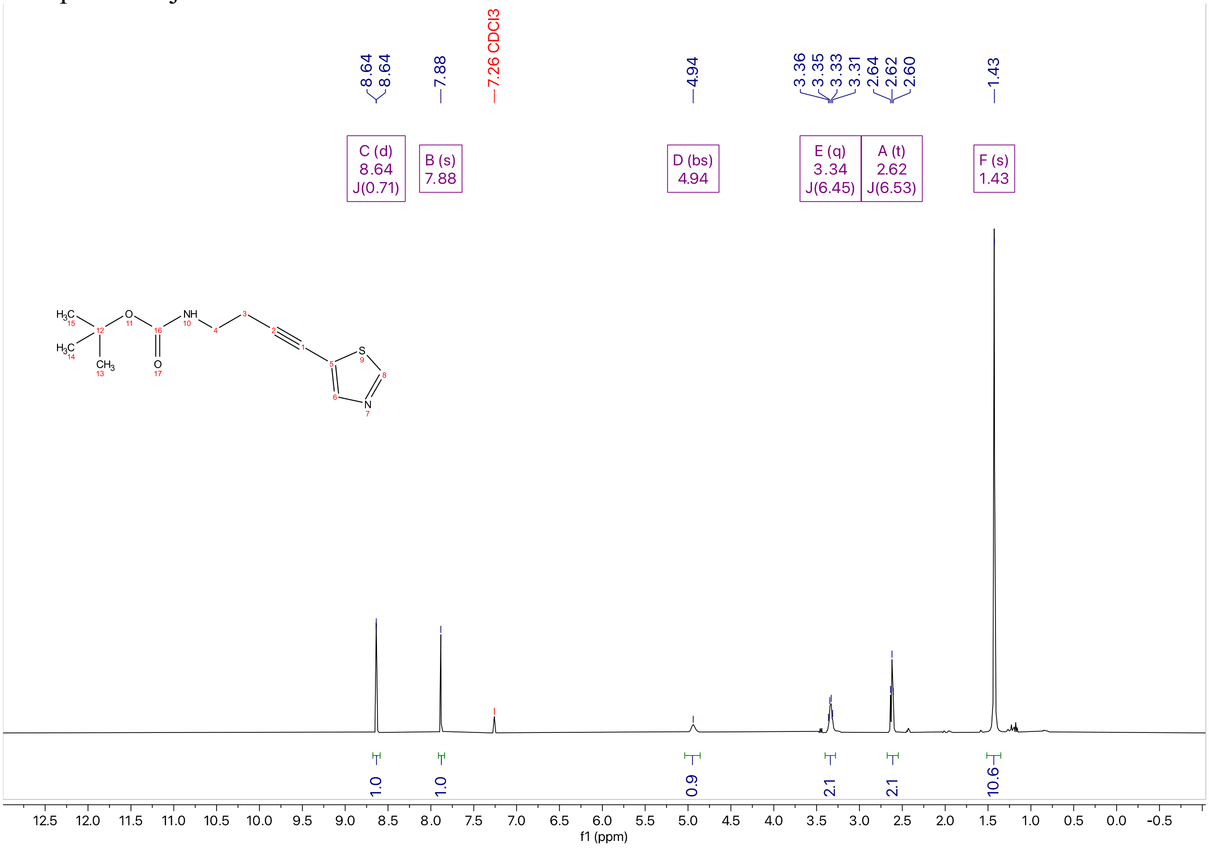

Compound S2j ^{13}C

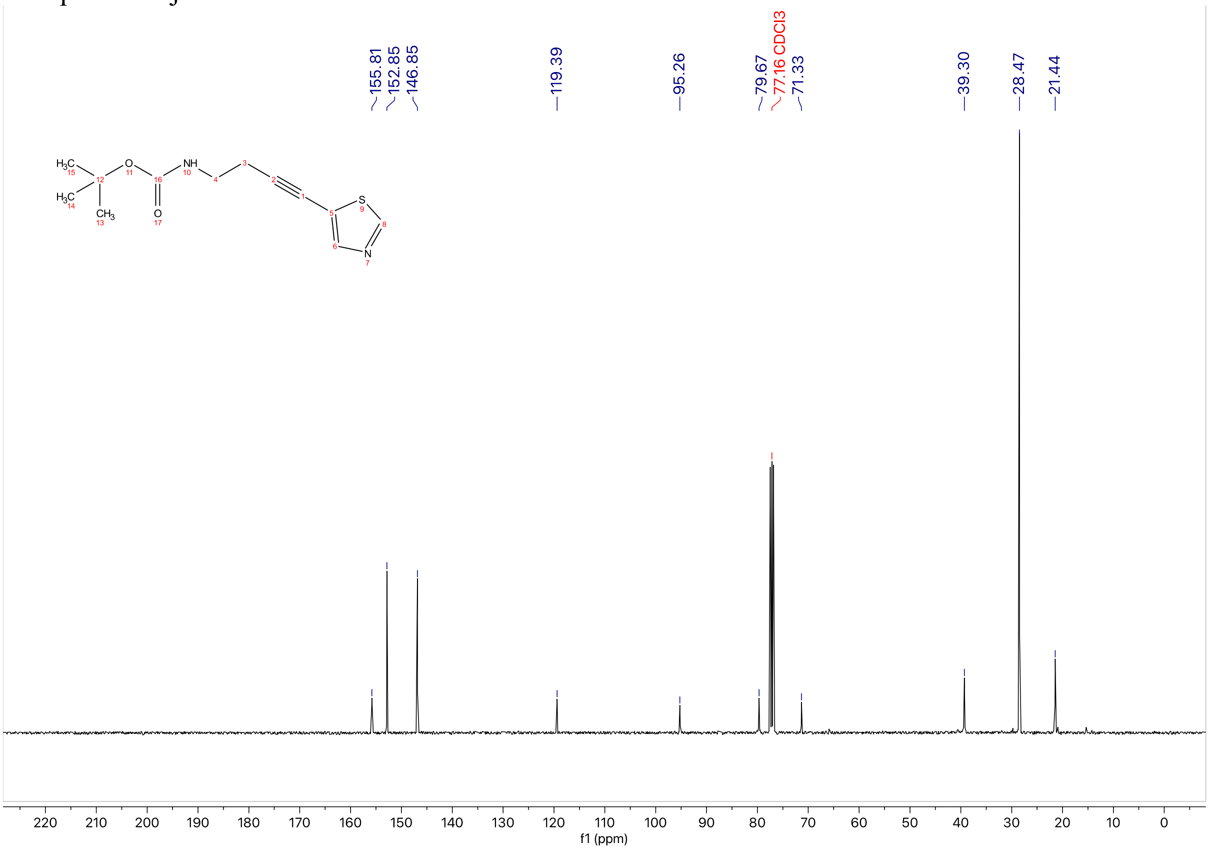

Compound S16 ^{1}H

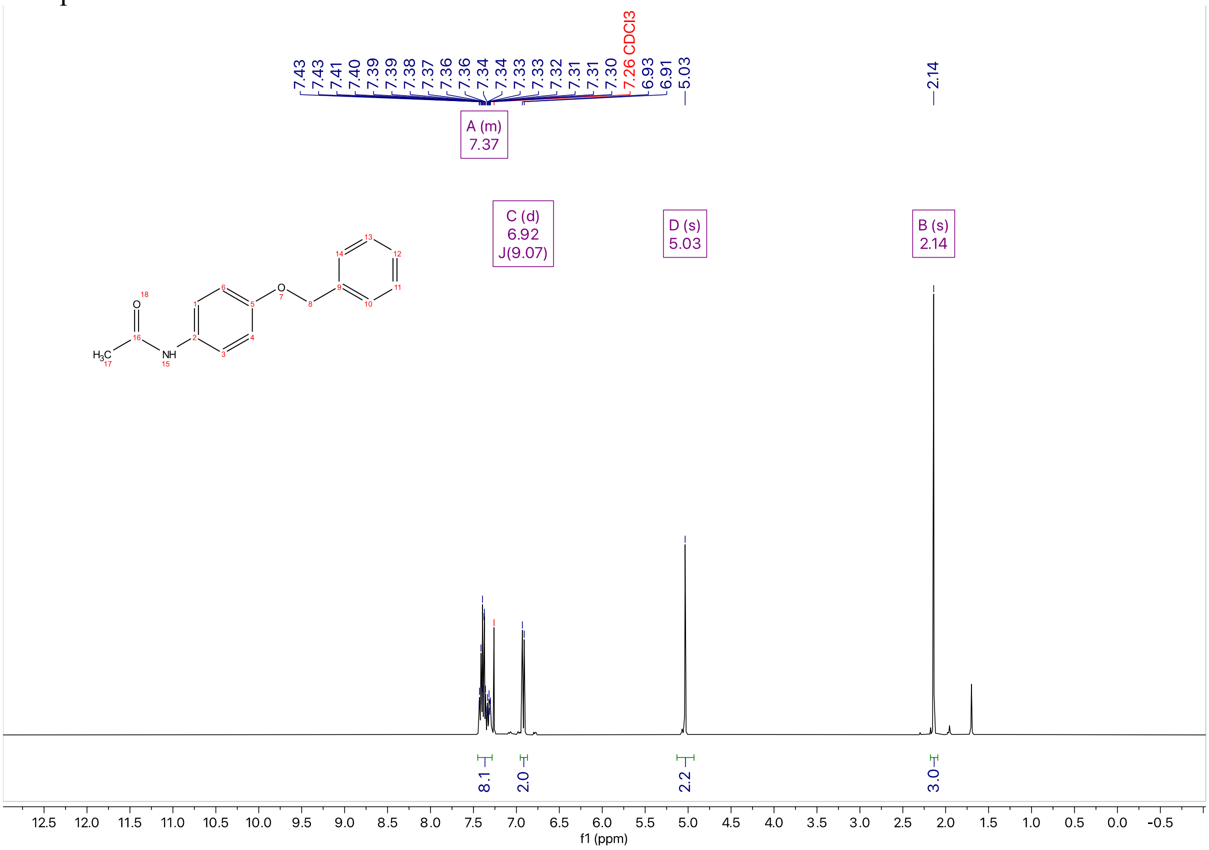

Compound S16 ^{13}C

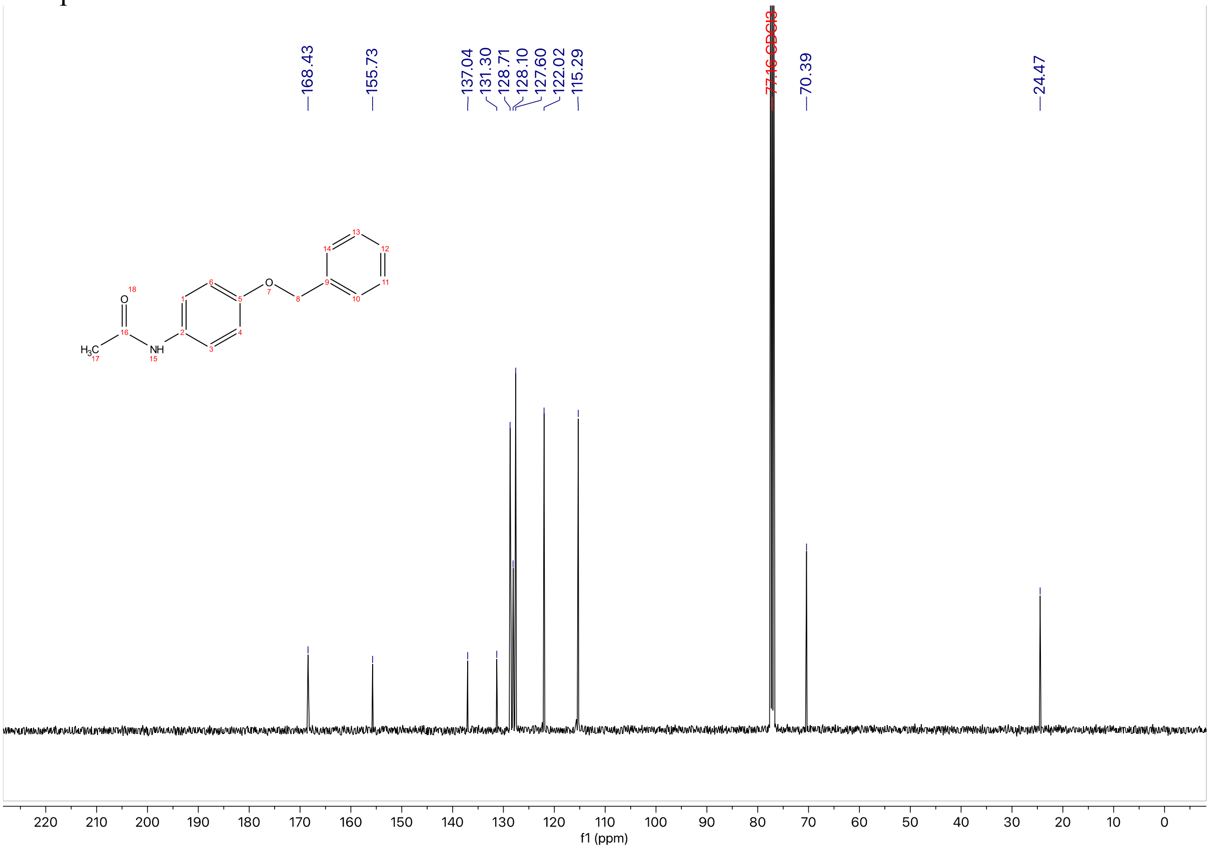

Compound S11 ^{1}H

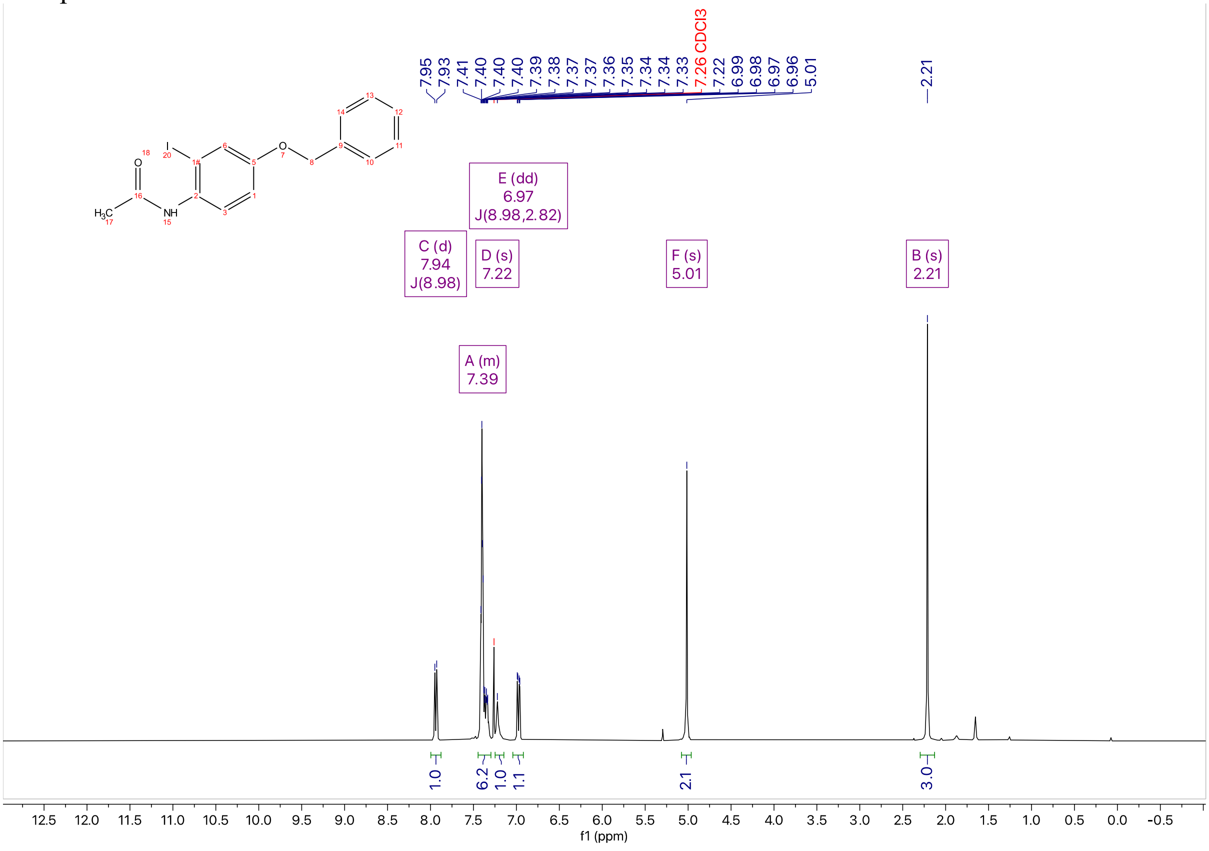

Compound S11 ^{13}C

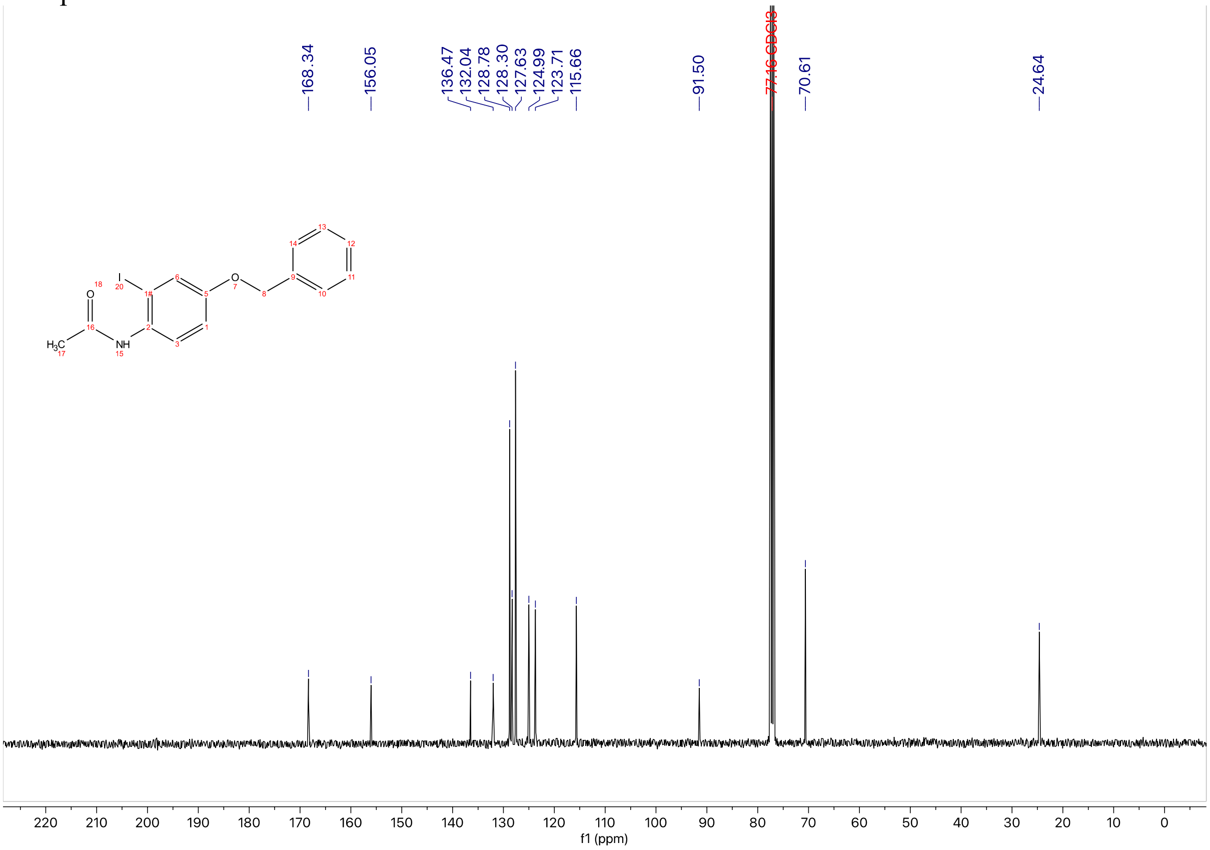

Compound S17 ^{1}H

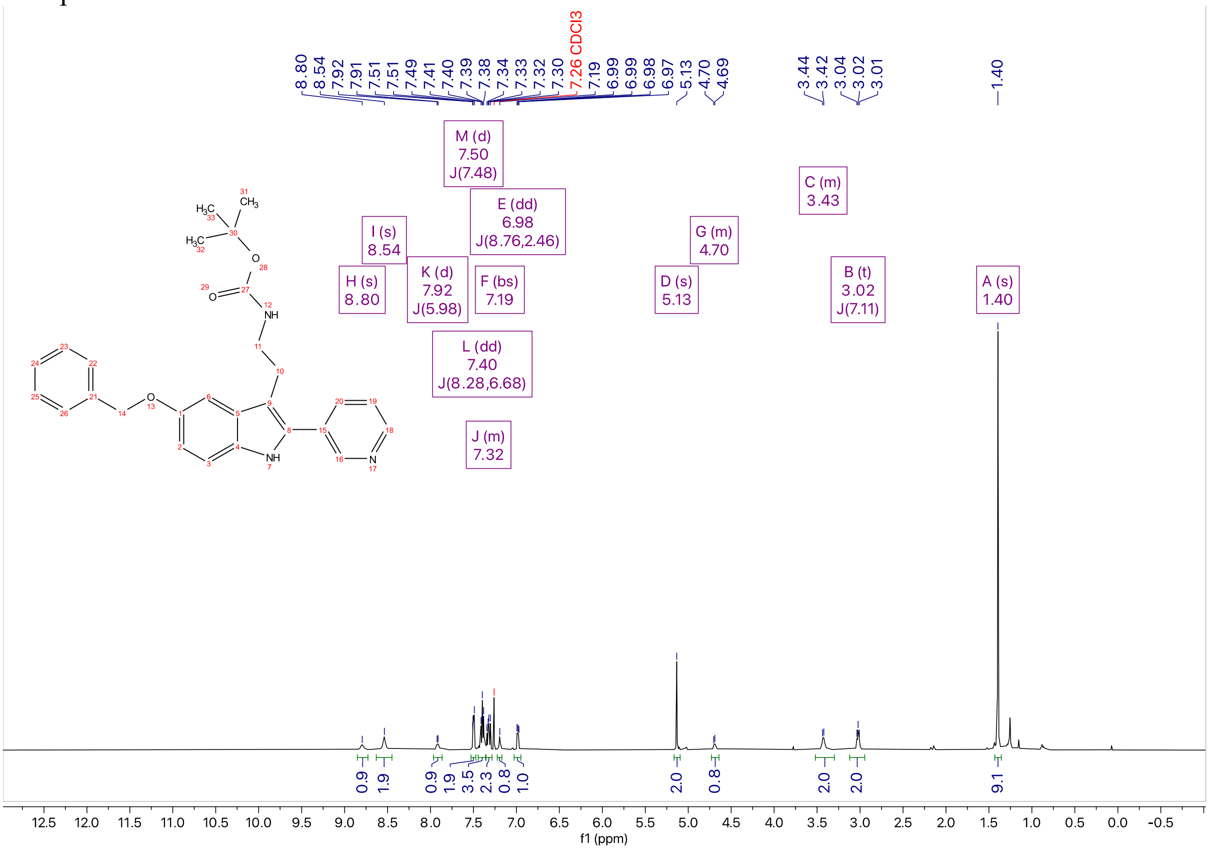

Compound S17 ^{13}C

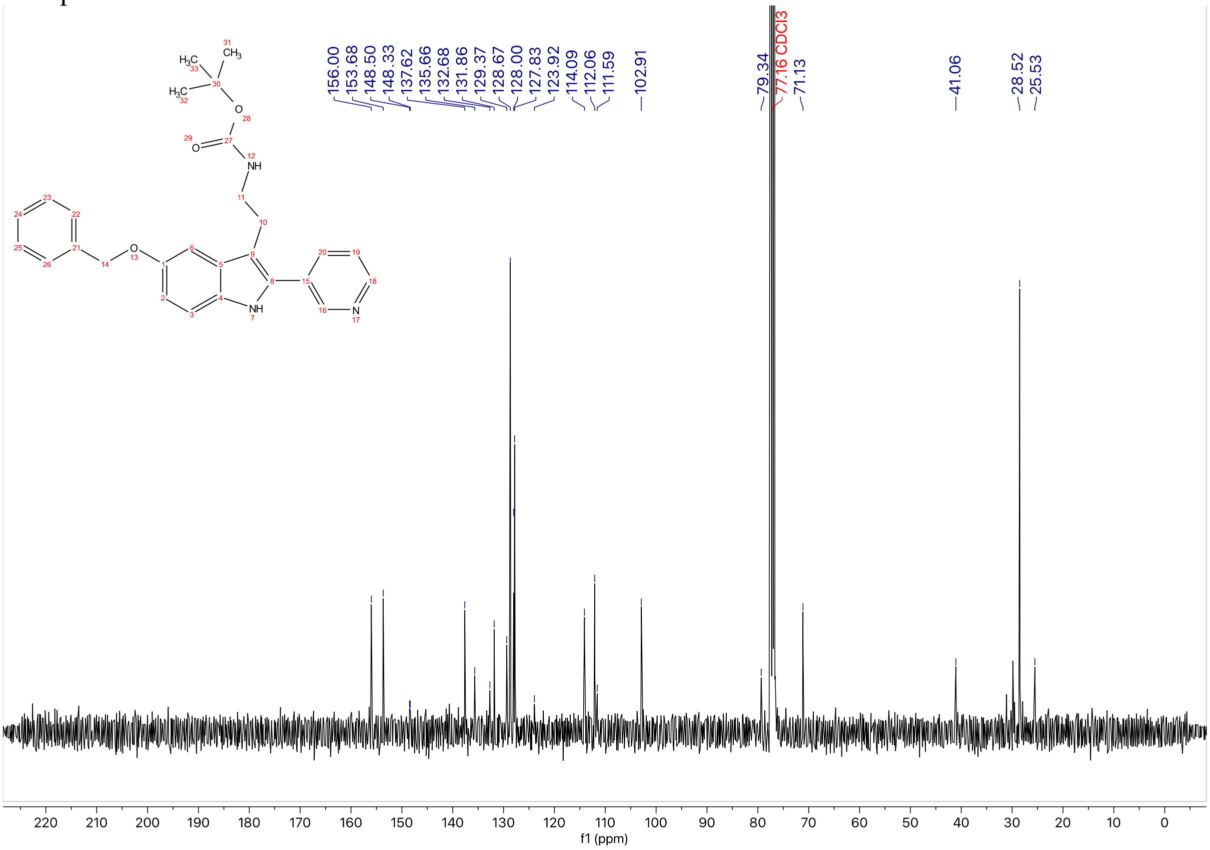

Compound S18 ^{1}H

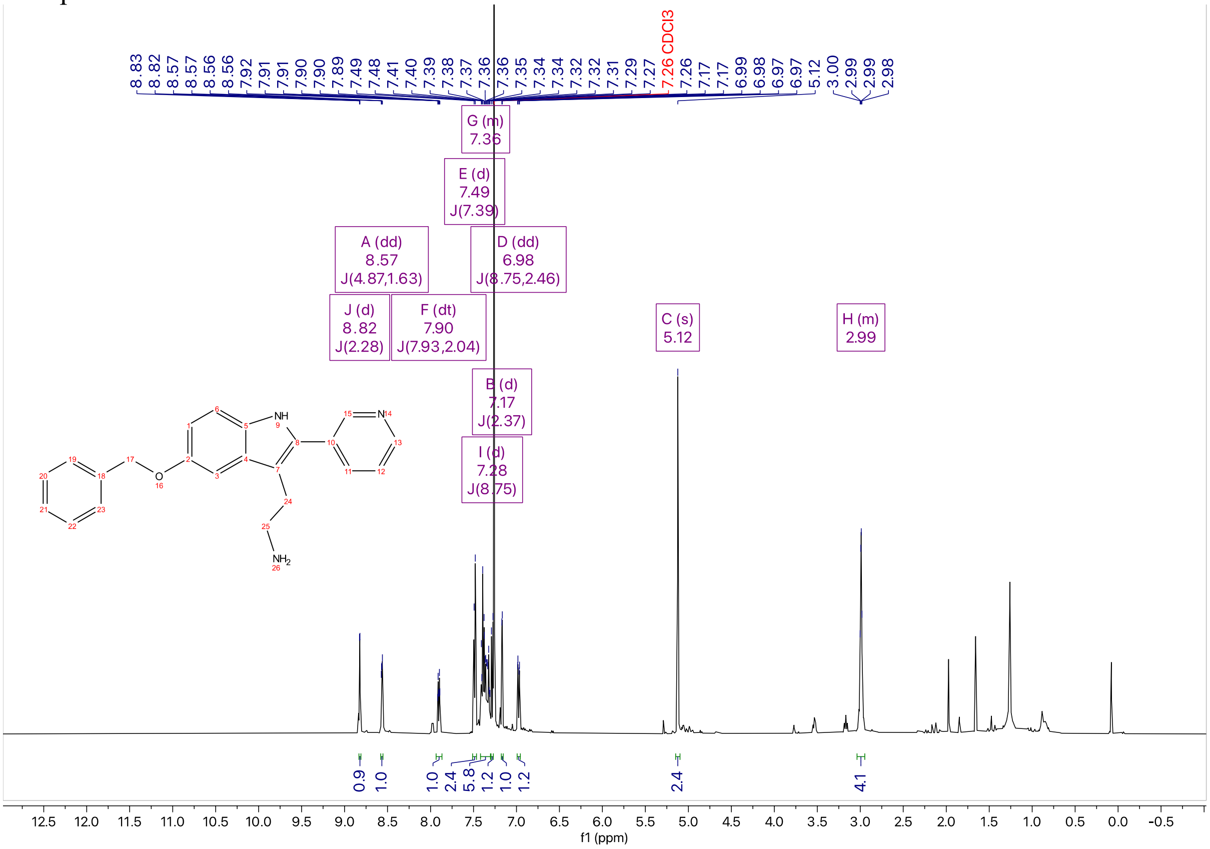

Compound S18 ^{13}C

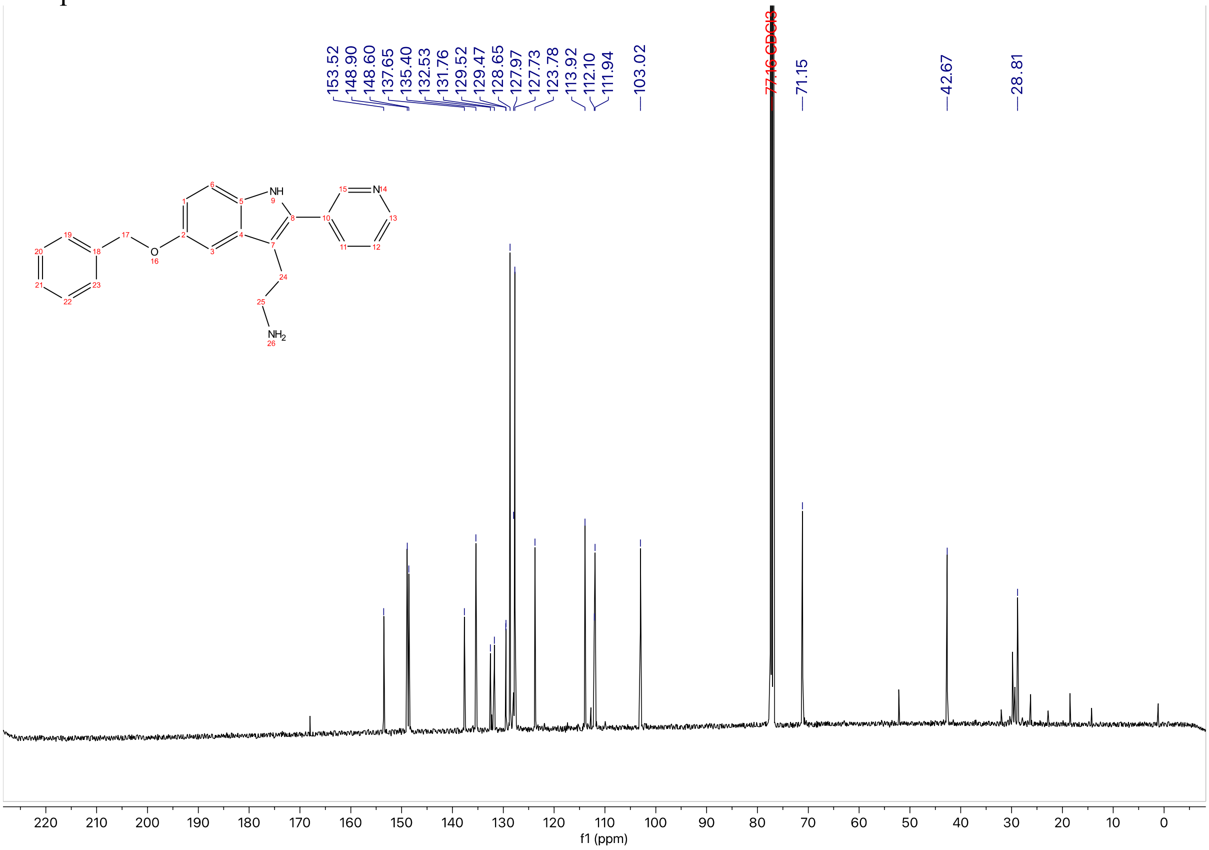

Compound S19 ^{1}H

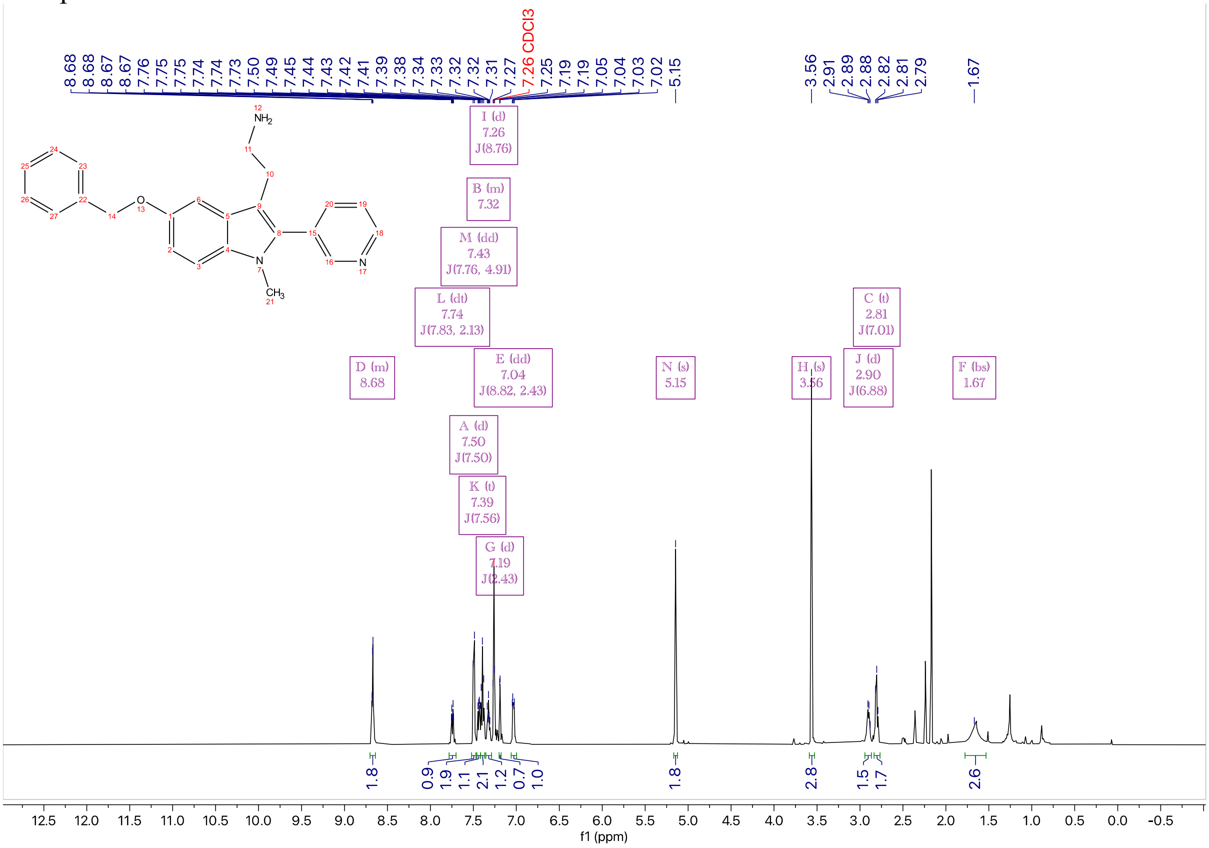

Compound S19 ^{13}C

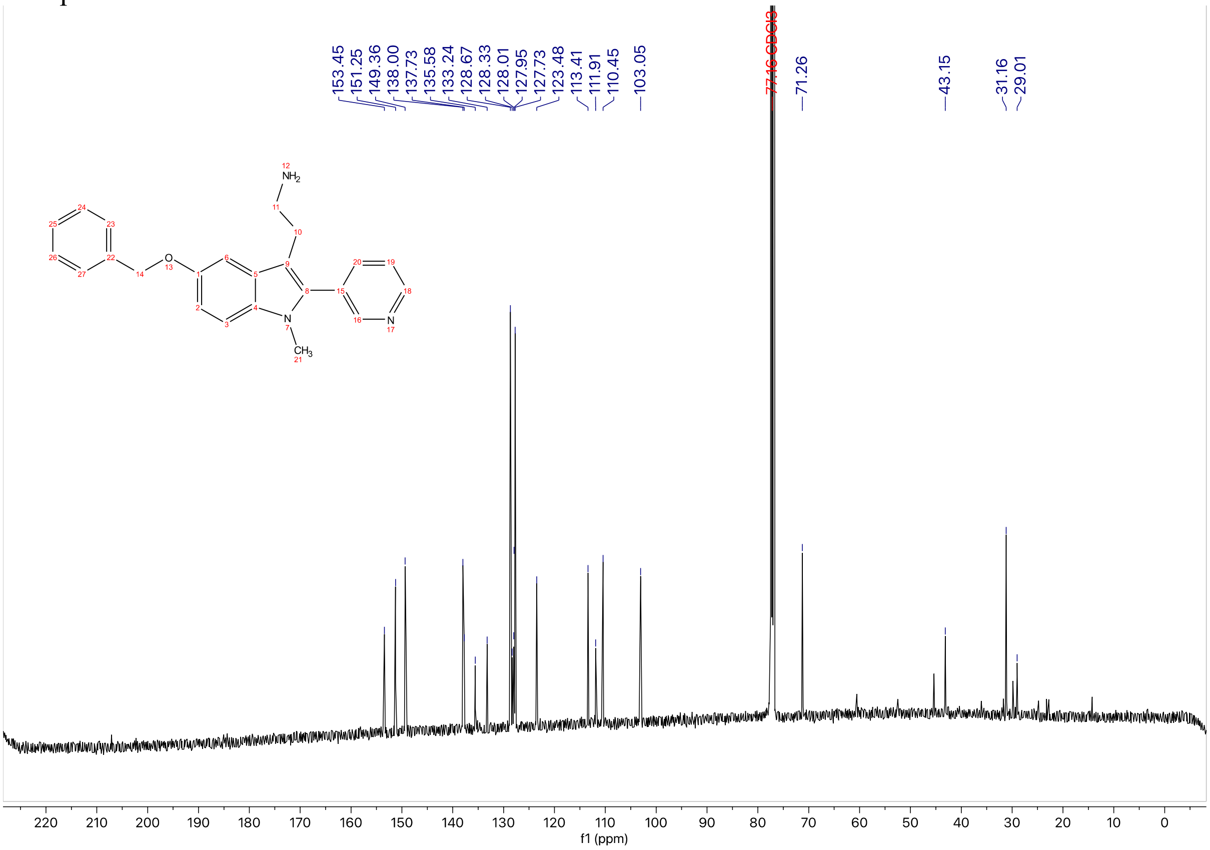

Compound S20 ^{1}H

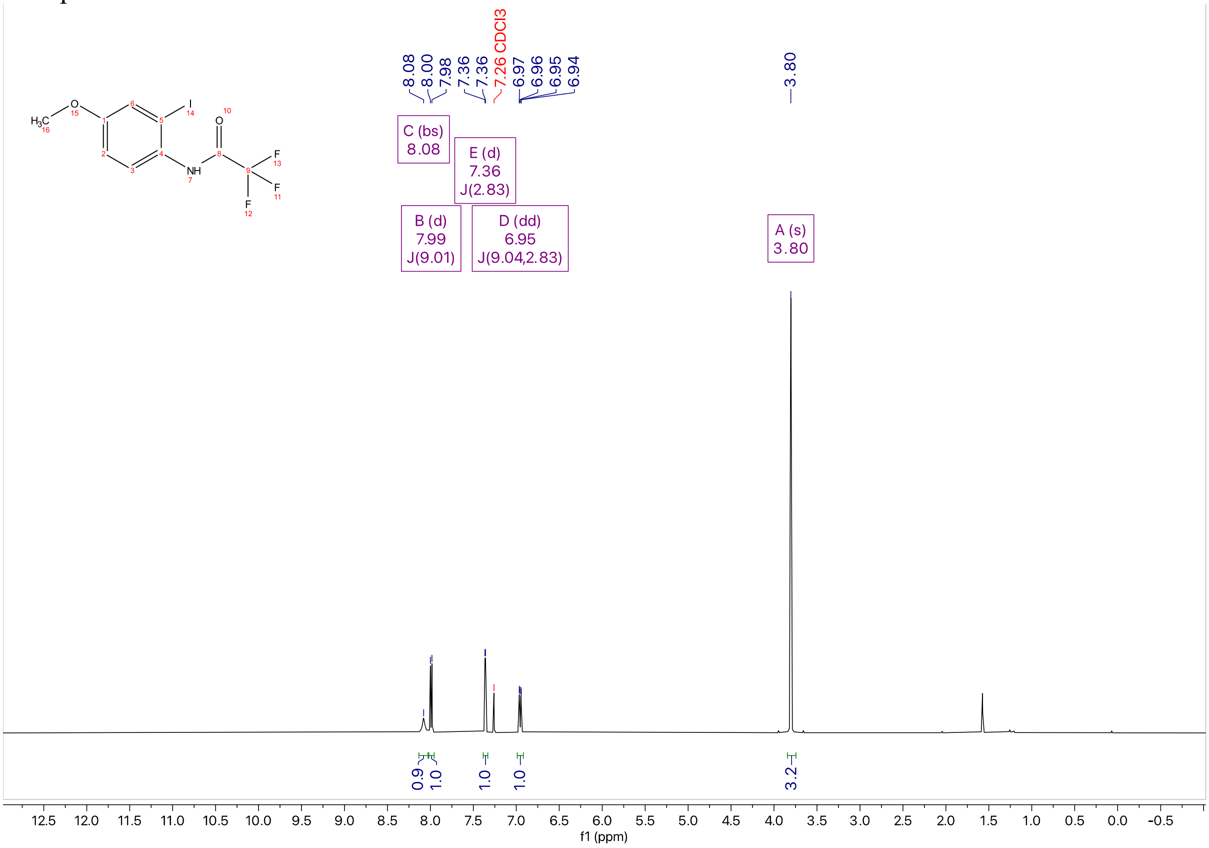

Compound S20 ^{13}C

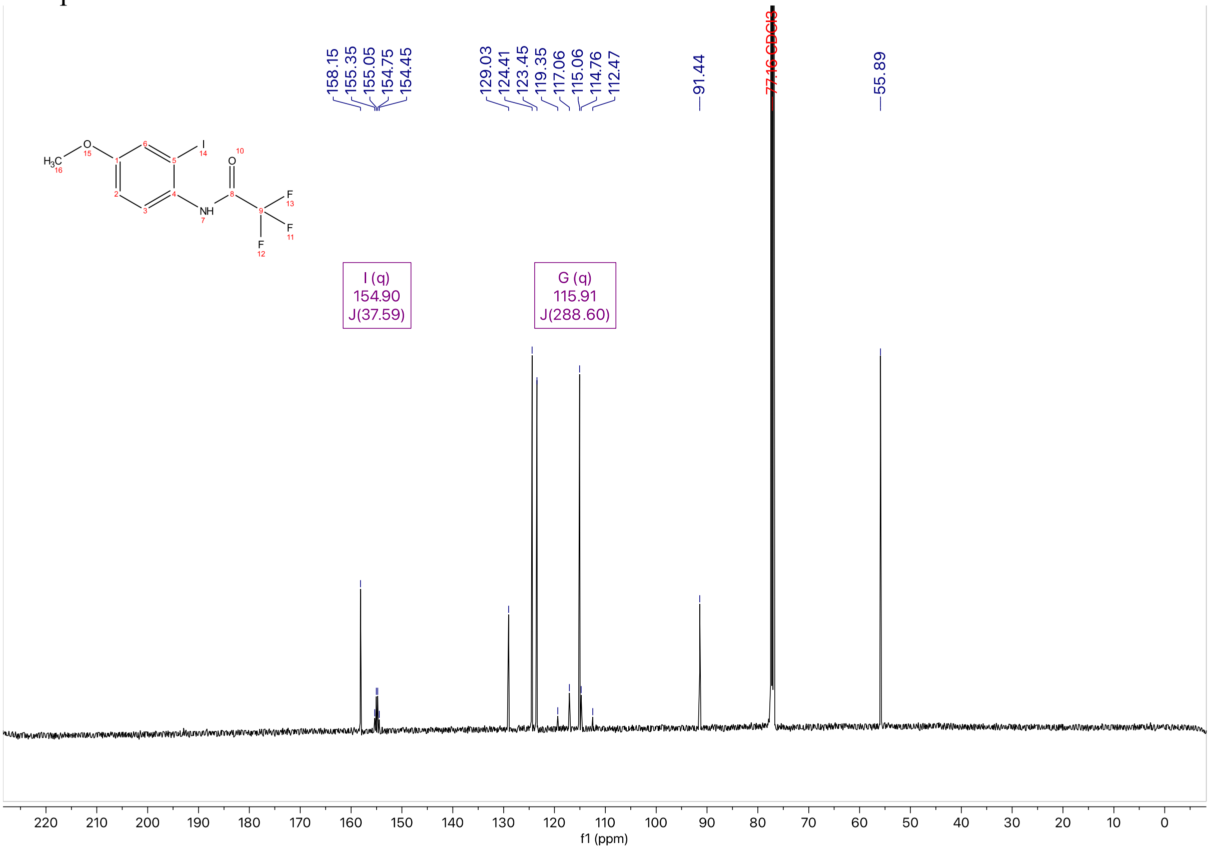

Compound S20 ^{19}F

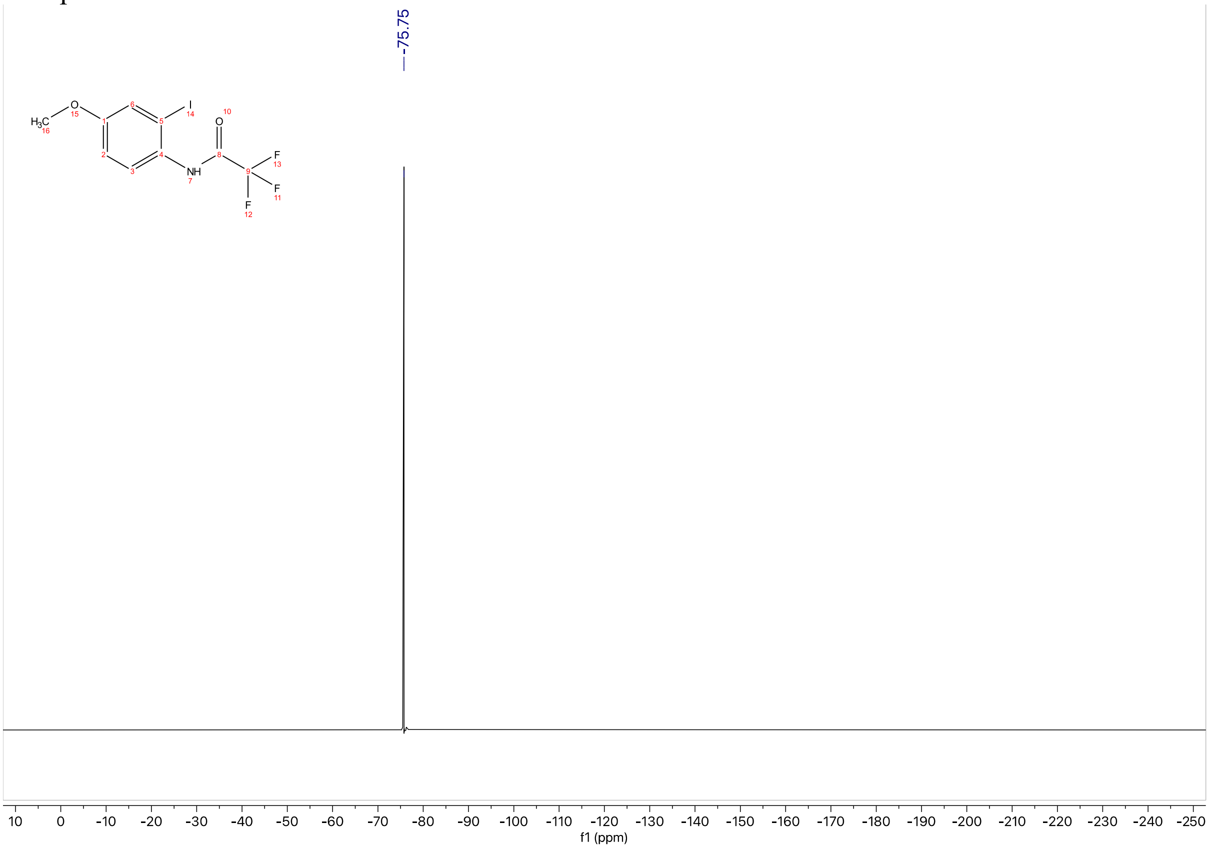

Compound S21 ^{1}H

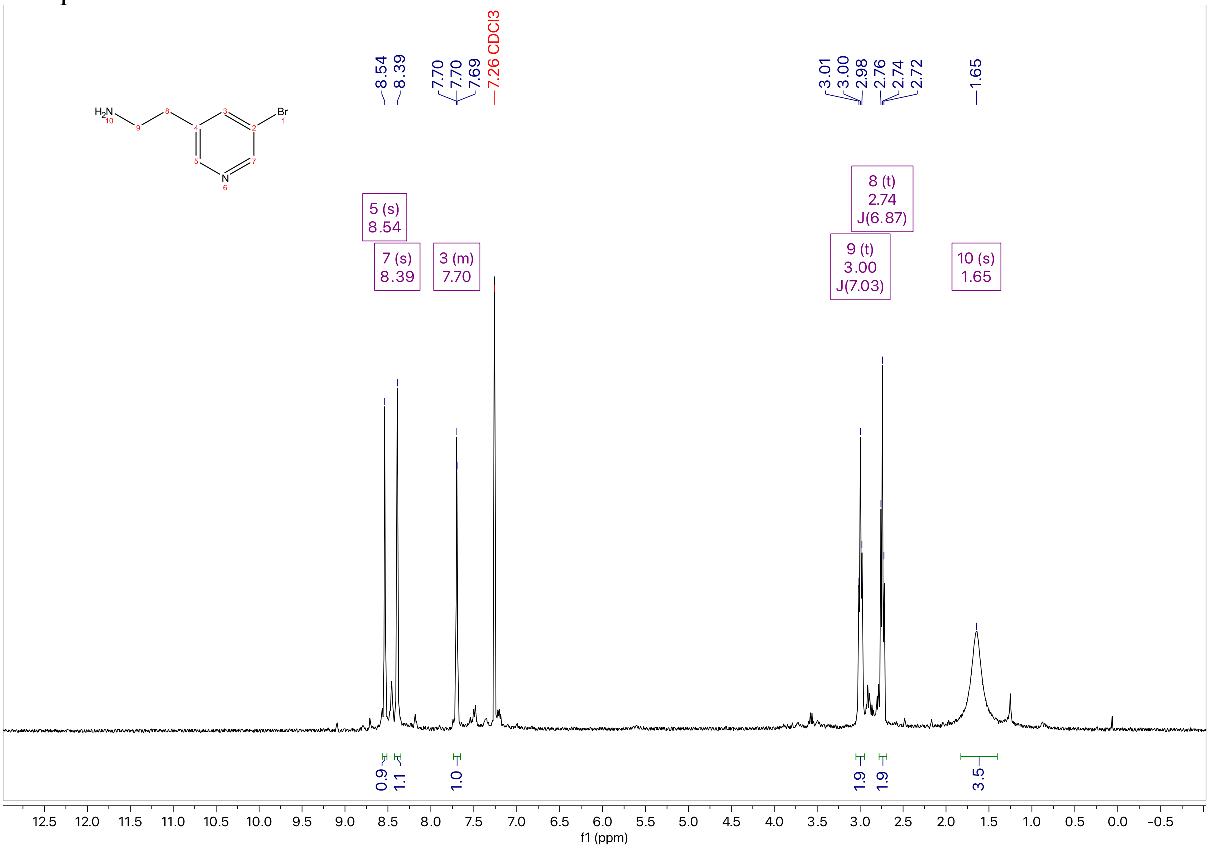

Compound S21 ^{13}C

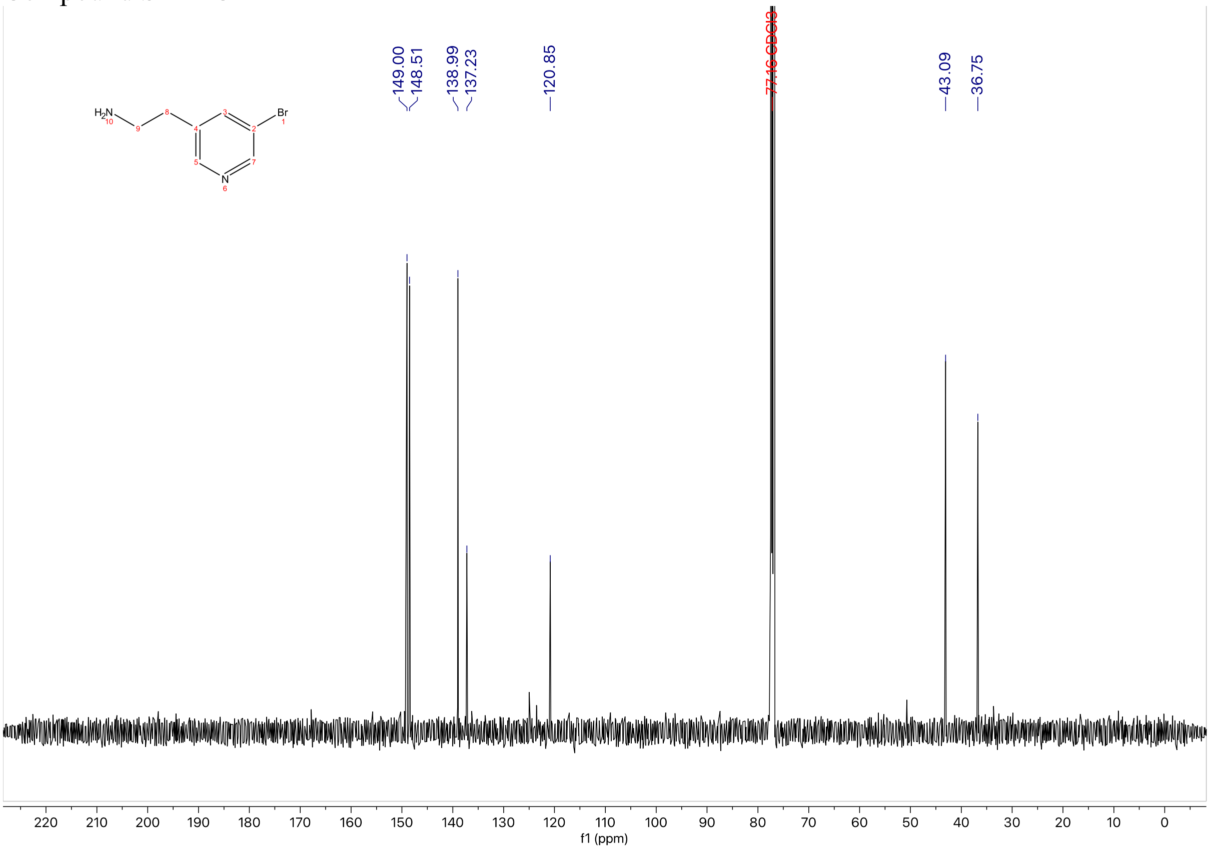

Compound S22 ^{1}H

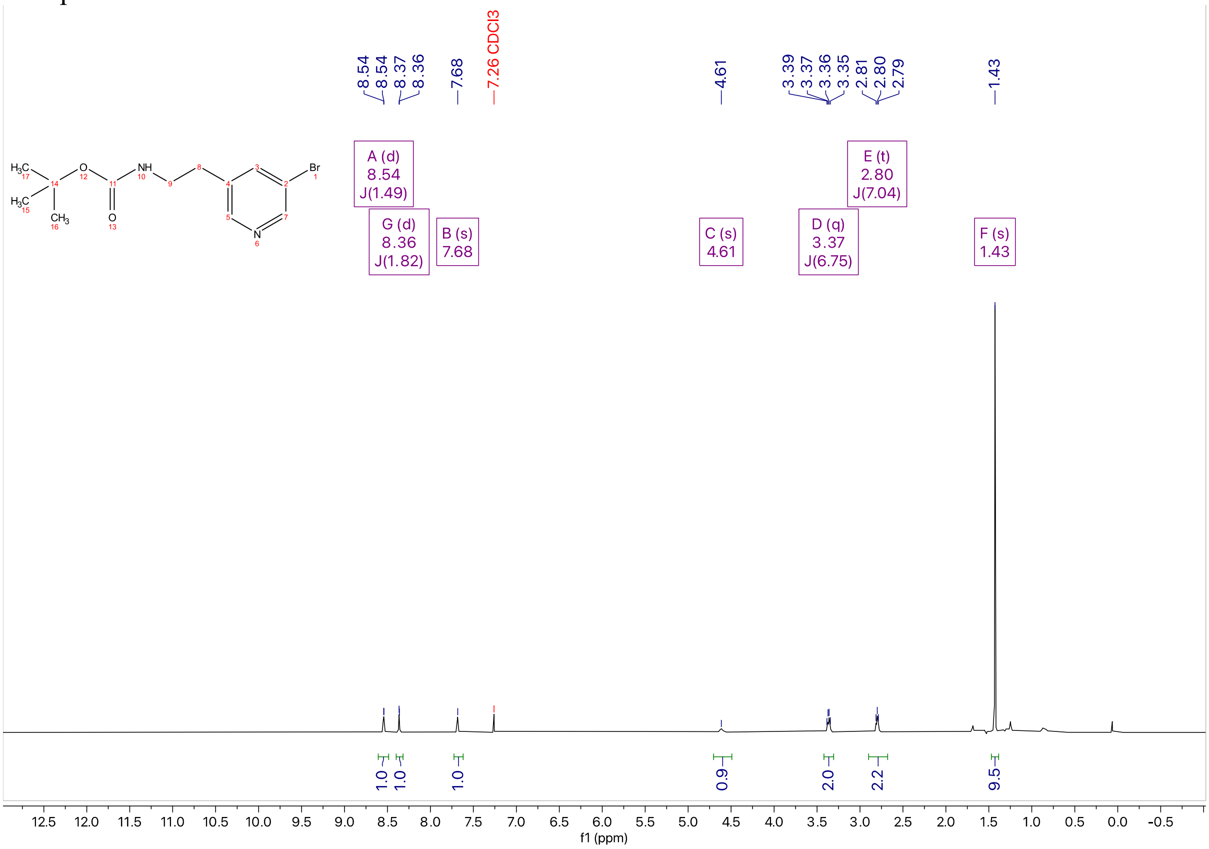

Compound S22 ^{13}C

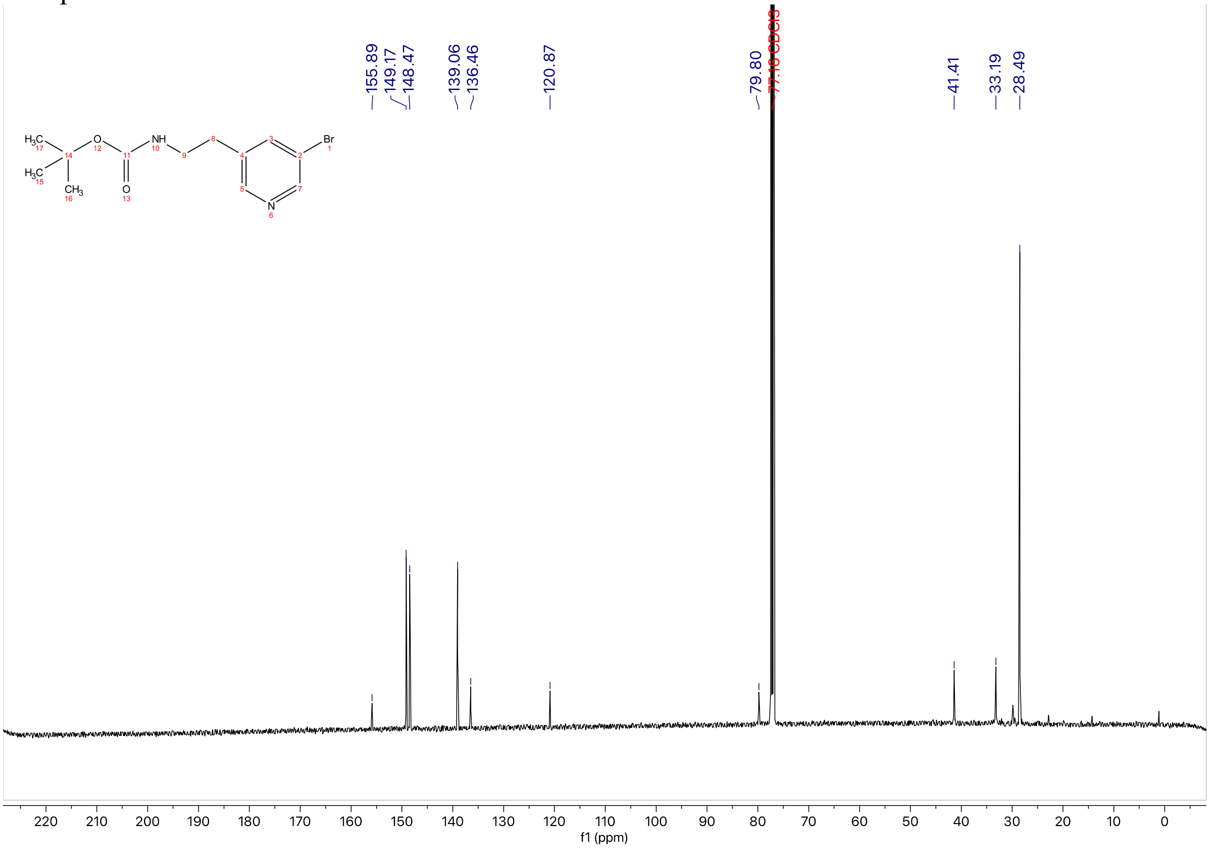

Compound S23 ^{1}H

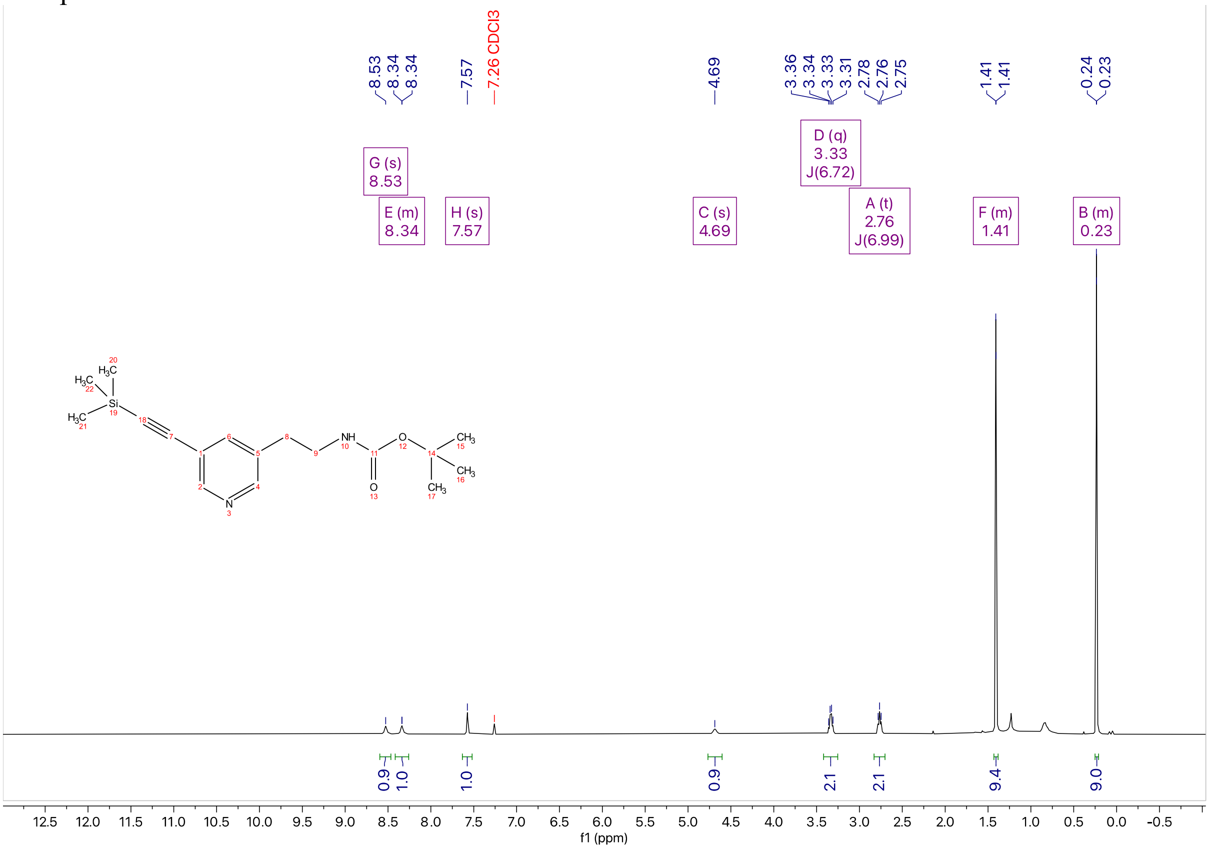

Compound S23 ^{13}C

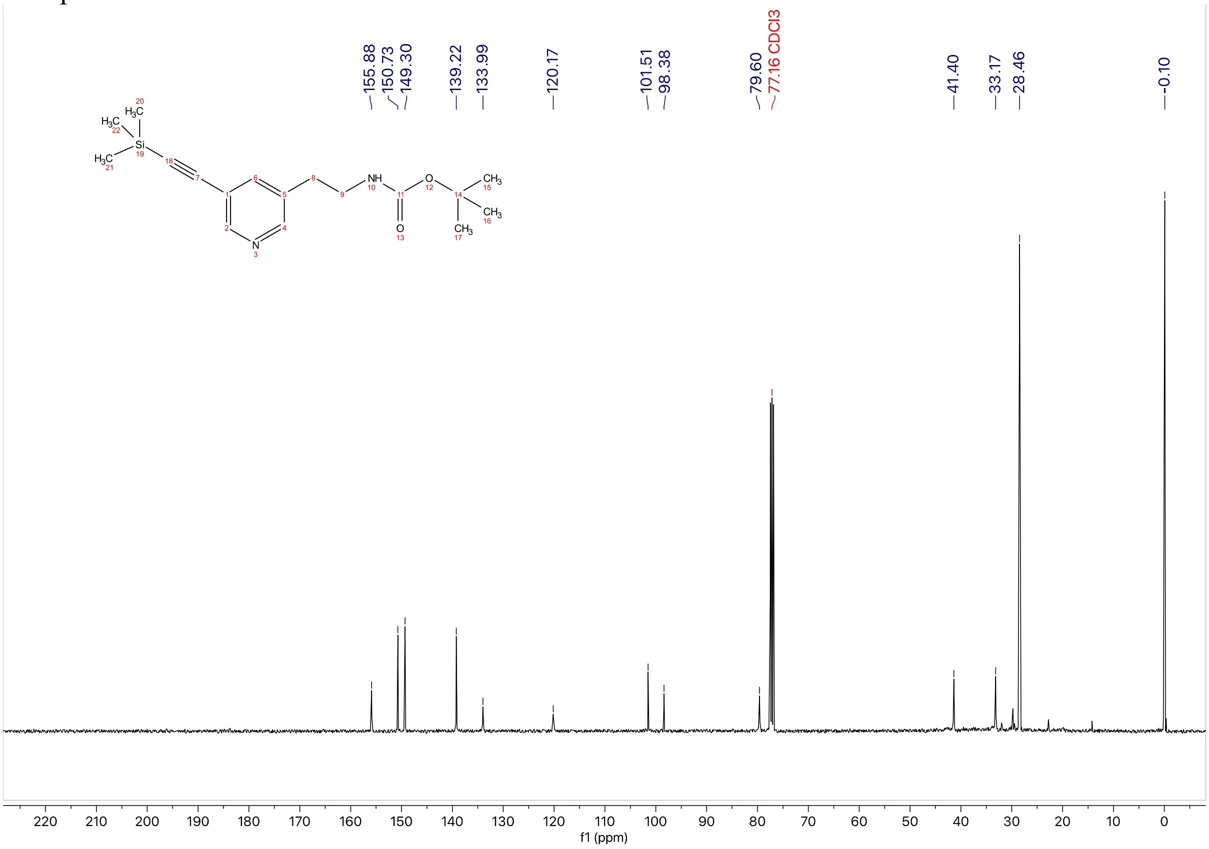

Compound S24 ^{1}H

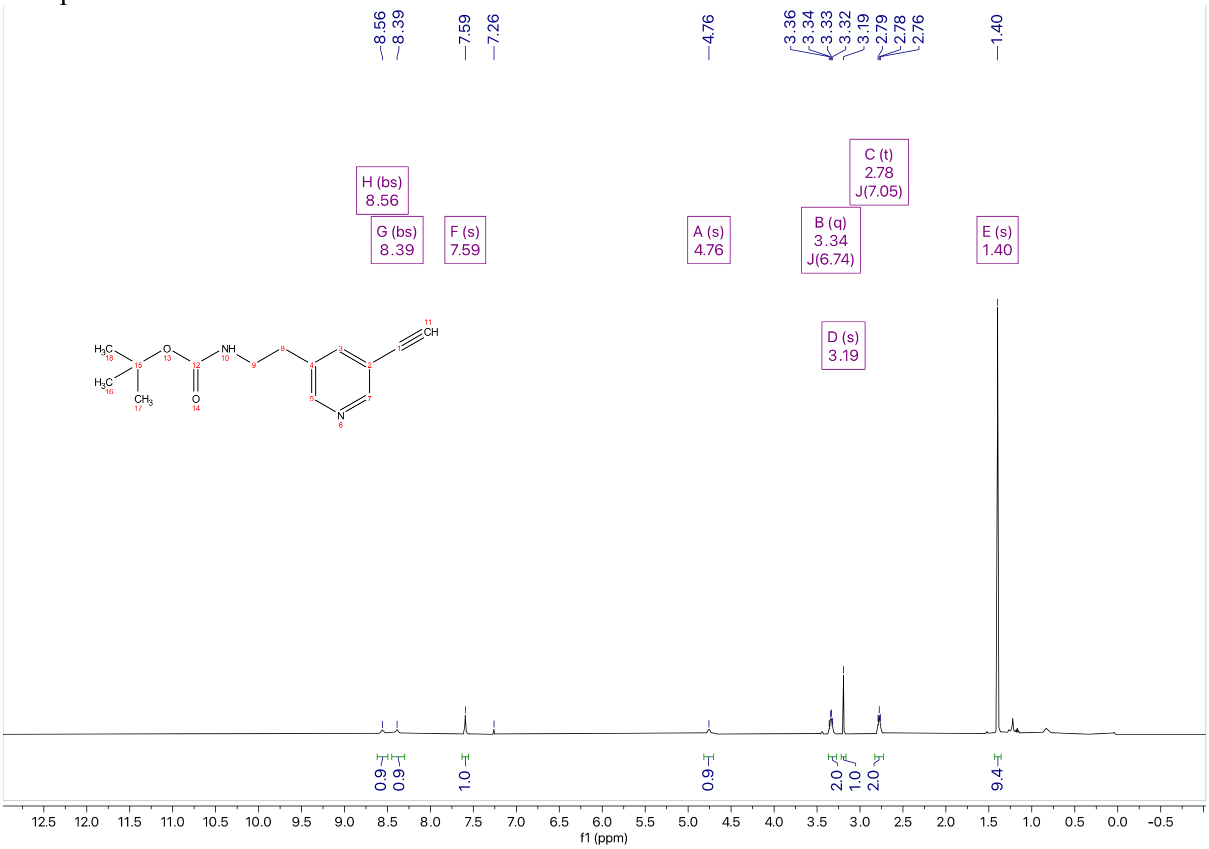

Compound S24 ^{13}C

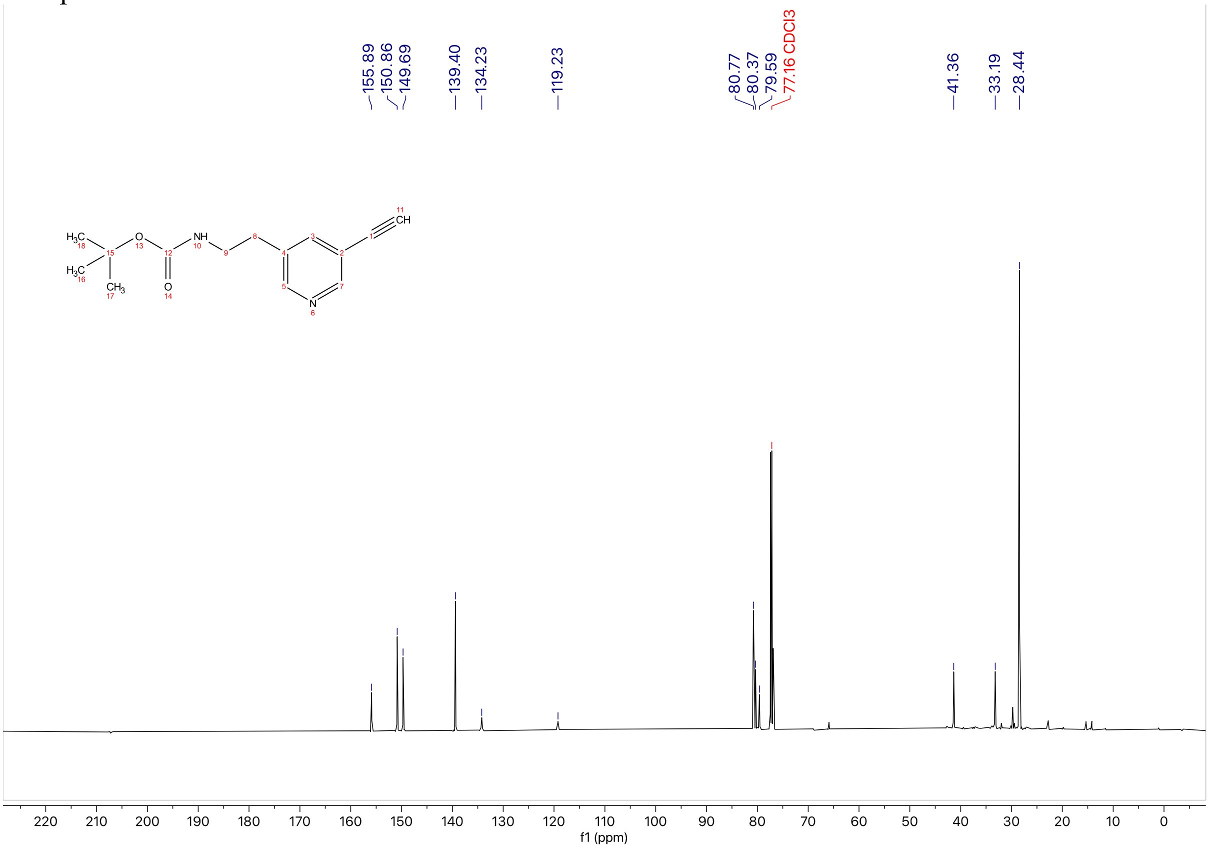

Compound S25 ^{1}H

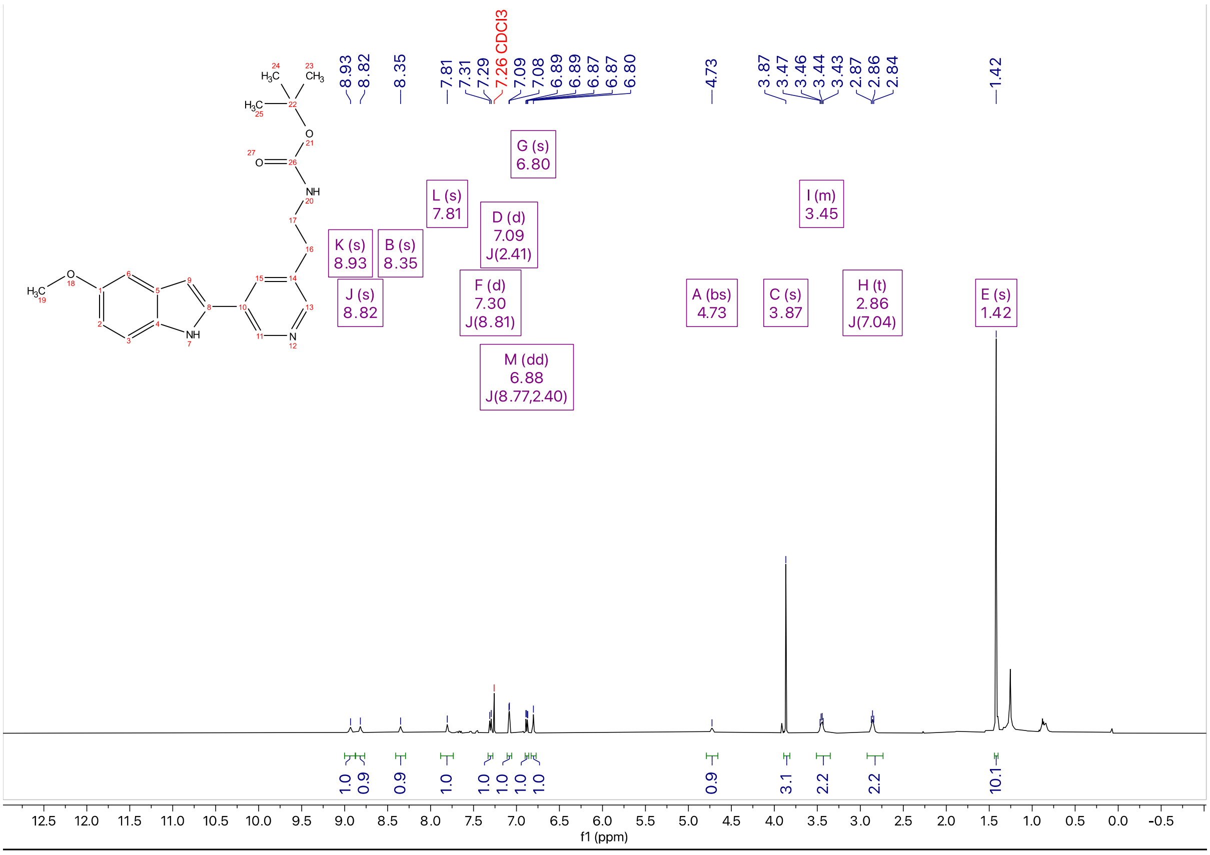

Compound S25 ^{13}C

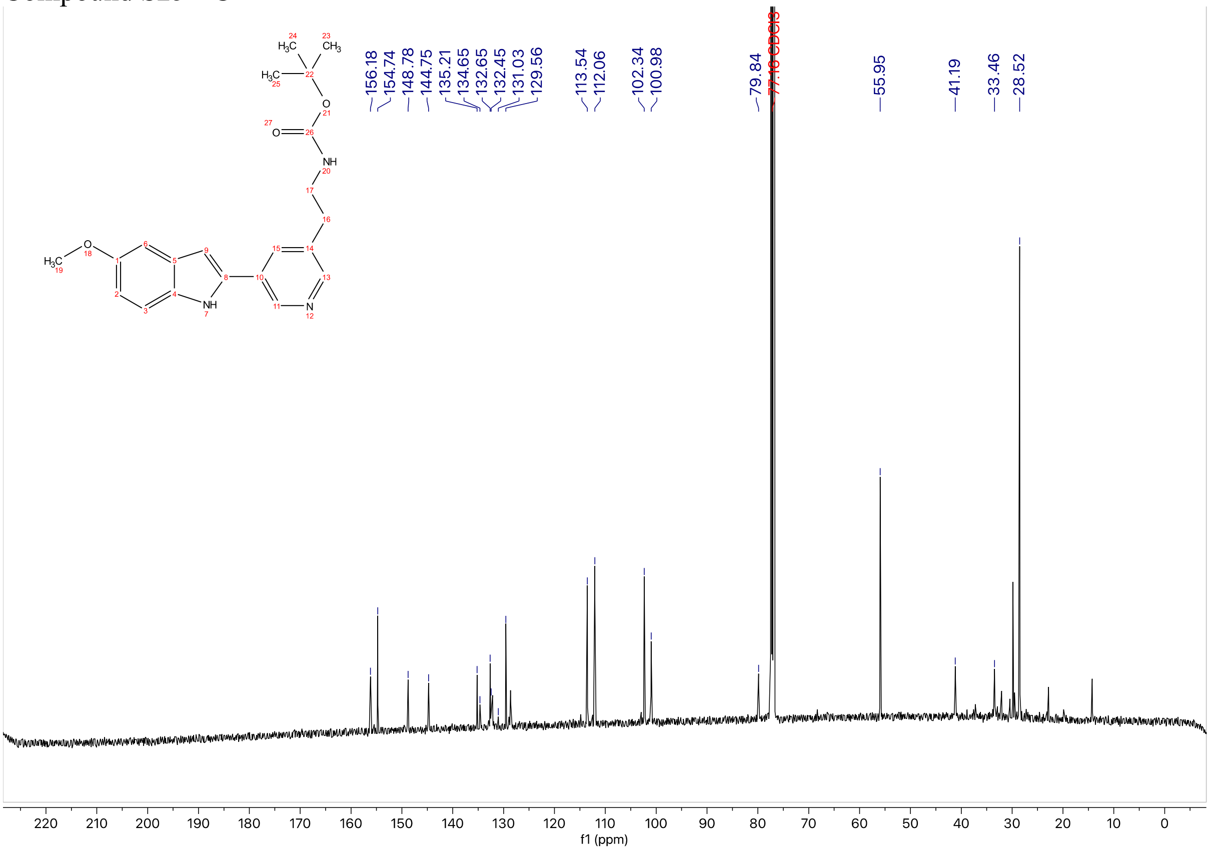

Compound S26 ^{1}H

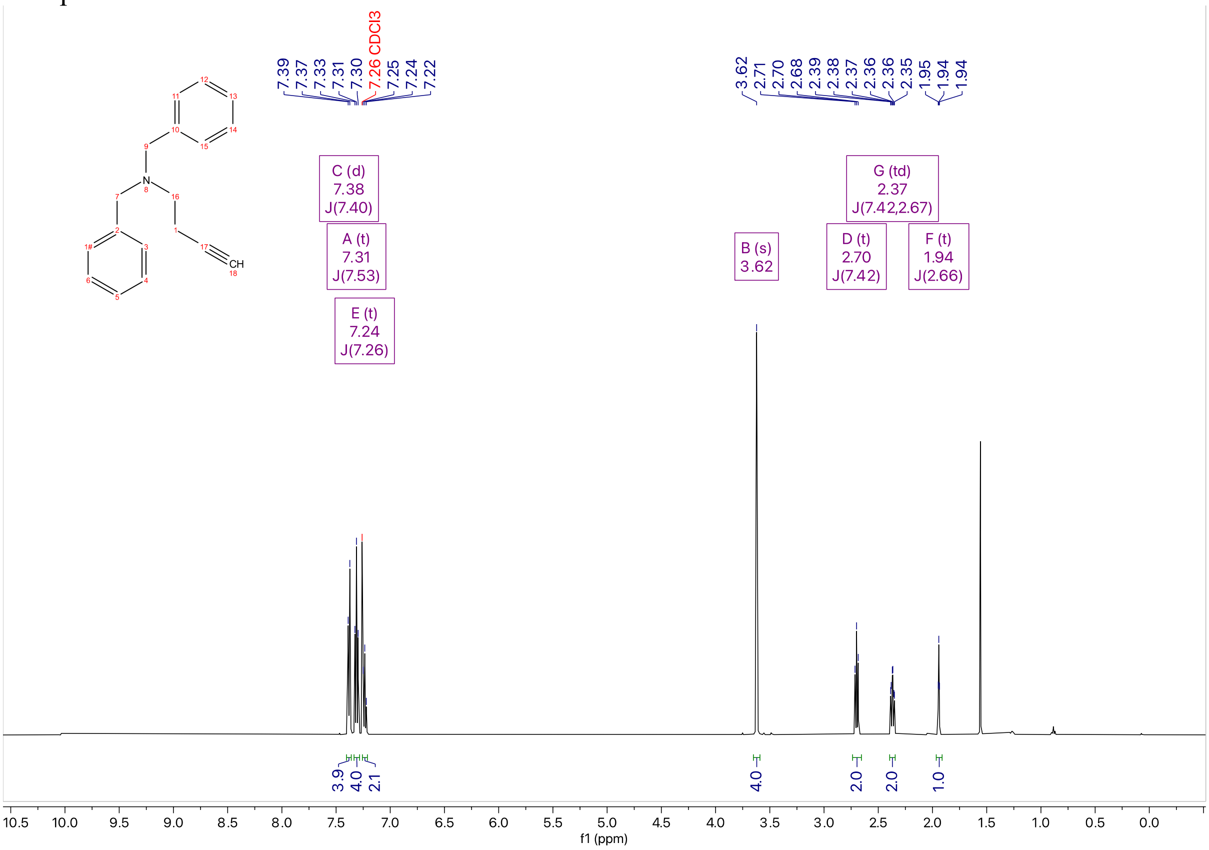

Compound S26 ^{13}C

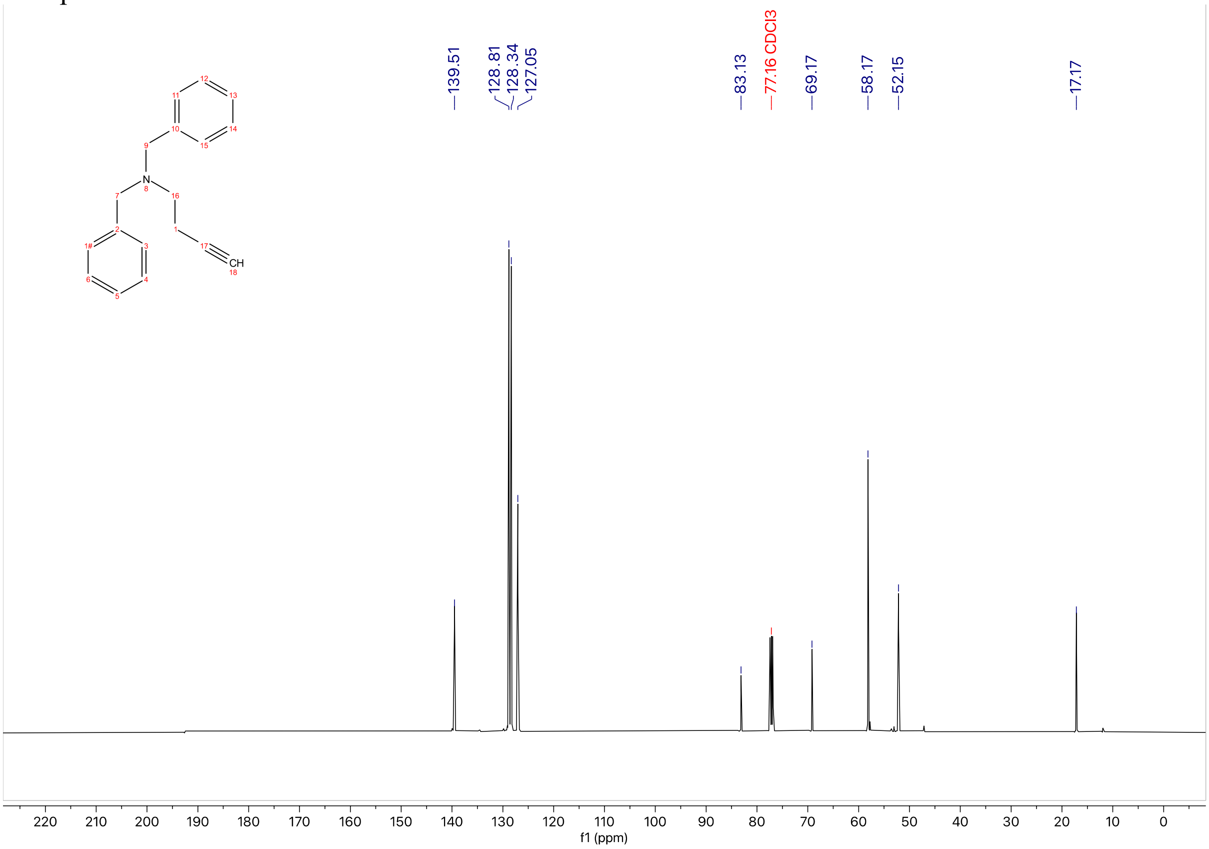

Compound S27 ^{1}H

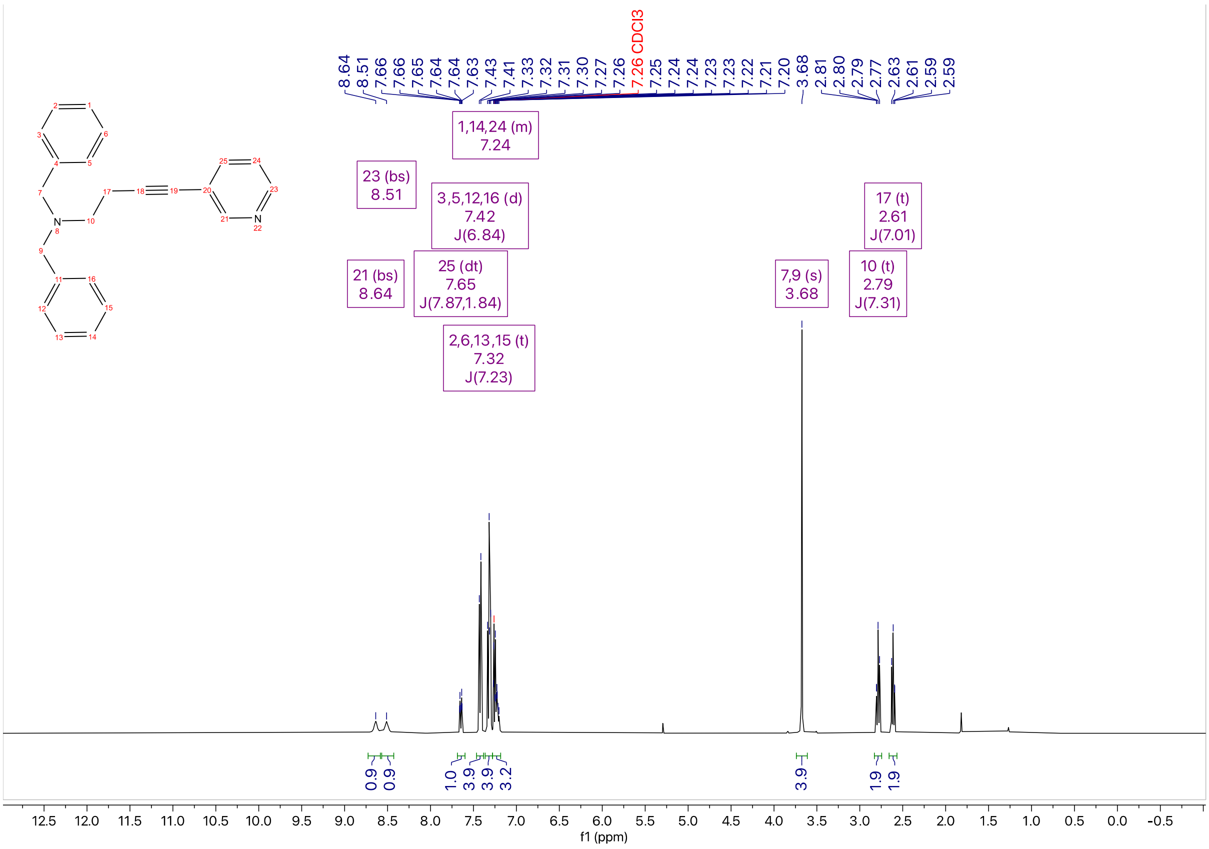

Compound S27 ^{13}C

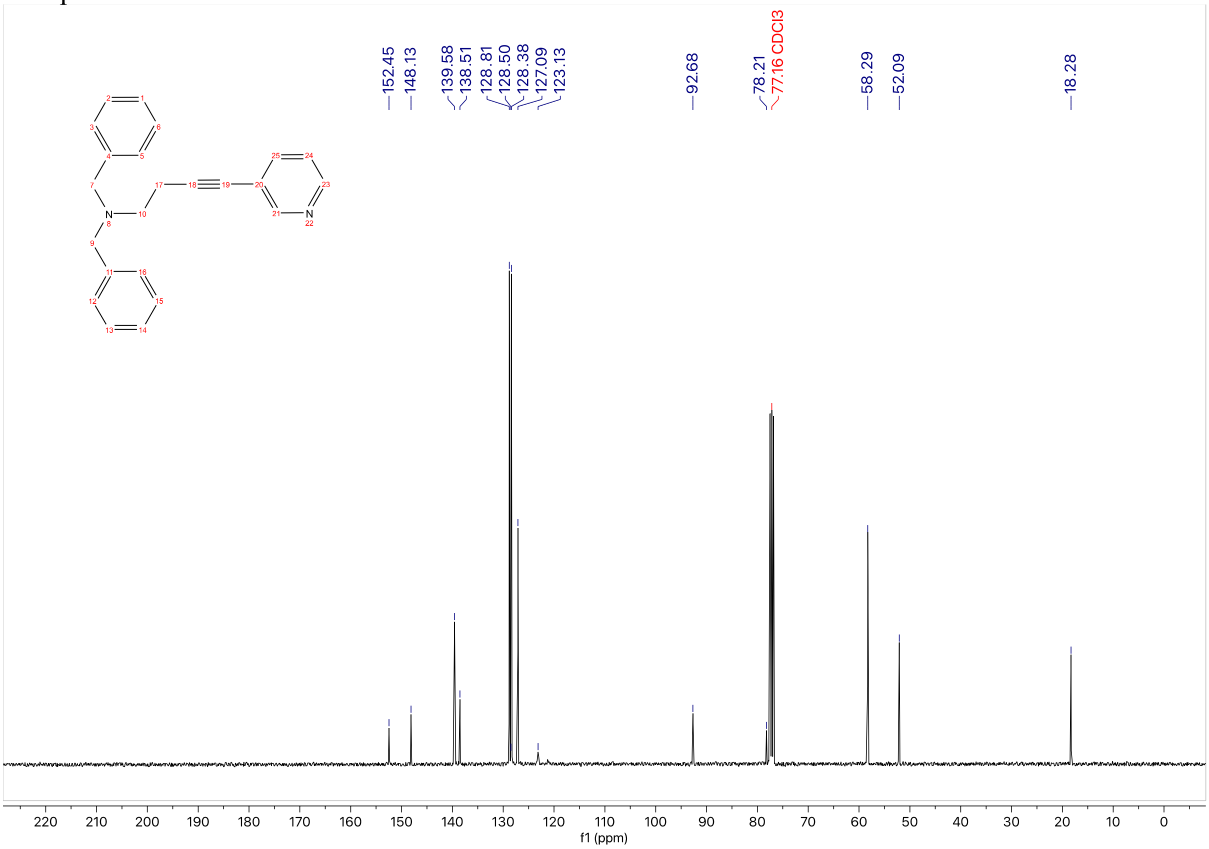

Compound S28 ^{1}H

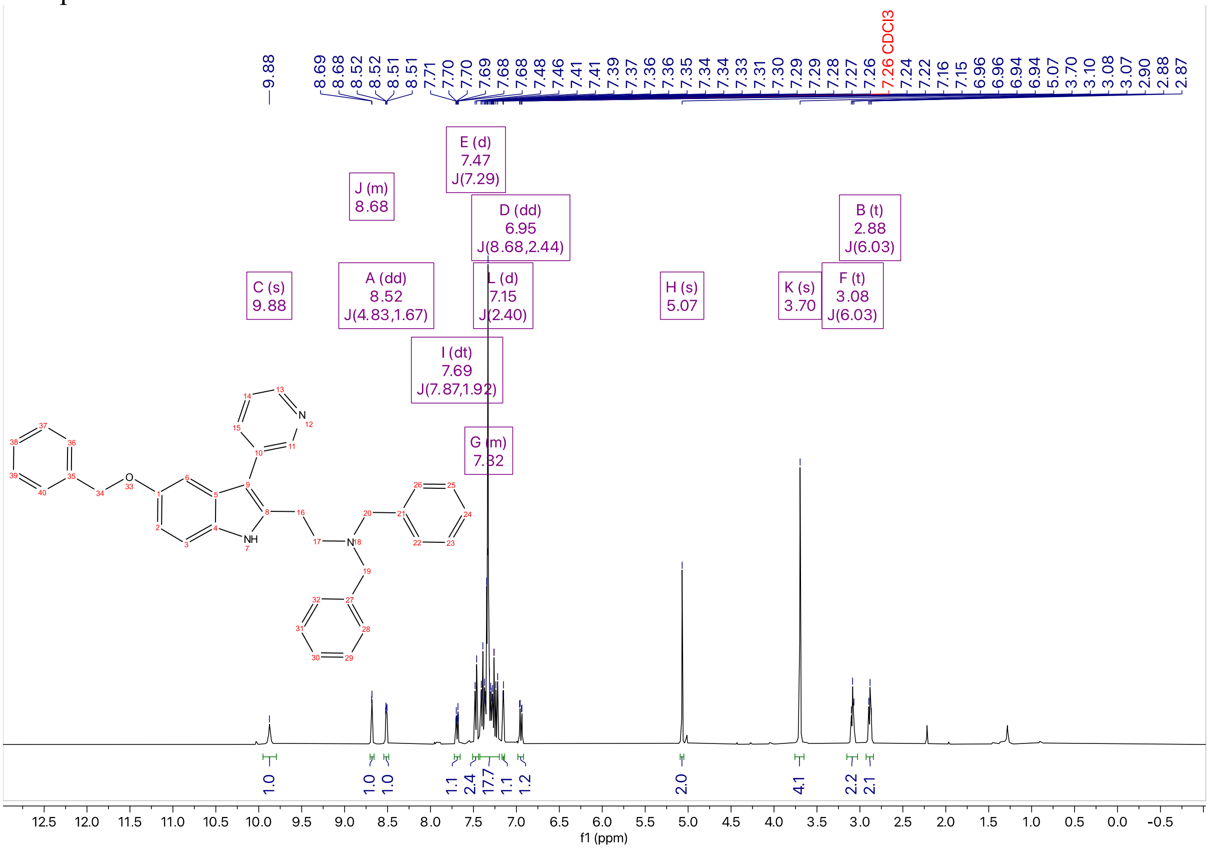

Compound S28 ^{13}C

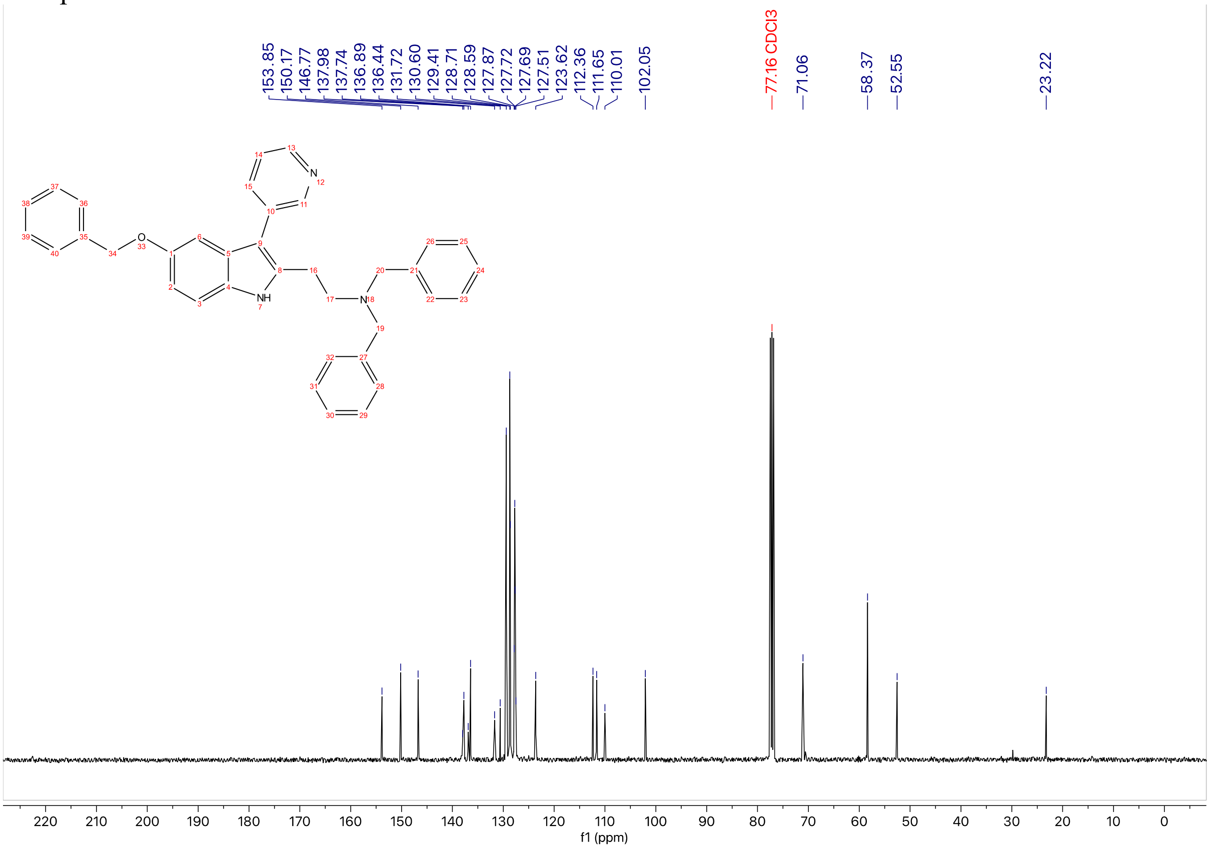

Supplement: Supplementary file 1 [file jm5c01454_si_001.pdf]
