# Supplementary material for: Giving an Enzyme Scissors: Serotonin Derivatives as Potent Organocatalytic Switches for DNA Repair Enzyme OGG1
Source: J Med Chem. 2025 Oct 15;68(21):22455–83. doi: 10.1021/acs.jmedchem.5c01454 (PMC12621200; doi:10.1021/acs.jmedchem.5c01454)
Supplement: Supplementary file 3 [file jm5c01454_si_003.pdf]

List of parameters

Lys249.prepc

0 0 2

This is a remark line
molecule.res
MOL XYZ 0
CHANGE OMIT DU BEG
0.0000
1 DUMM DU M 999.000 999.0
-999.0 .00000
2 DUMM DU M 999.000 -999.0
999.0 .00000
3 DUMM DU M -999.000 999.0
999.0 .00000
4 N1 n7 M -0.312000 -2.394000
-0.607000 -0.695713
5 H1 hn E 0.327000 -2.700000
0.102000 0.365186
6 C7 c3 3 -1.173000 -3.483000
-1.025000 0.026810
7 H11 h1 E -0.556000 -4.302000
-1.377000 0.038065
8 H12 h1 E -1.833000 -3.867000
-0.248000 0.038065
9 H13 h1 E -1.790000 -3.156000
-1.855000 0.038065
10 C1 c3 M -0.981000 -1.176000
-0.165000 0.316551
11 H2 h1 E -1.690000 -0.907000
-0.940000 0.079130
12 C3 c B 0.126000 -0.120000 -
0.099000 0.461224
13 O1 o E 0.729000 0.081000
0.923000 -0.569519
14 N3 ns B 0.421000 0.554000
-1.233000 -0.559357
15 H14 hn E 1.211000 1.152000
-1.137000 0.368396
16 C8 c3 3 -0.057000 0.275000
-2.571000 -0.003925
17 H15 h1 E 0.536000 0.855000
-3.265000 0.055765
18 H16 h1 E 0.048000 -0.772000
-2.822000 0.055765
19 H17 h1 E -1.093000 0.569000
-2.699000 0.055765
20 C2 c3 M -1.725000 -1.281000
1.171000 -0.193148
21 H3 hc E -2.405000 -2.127000
1.119000 0.061649
22 H4 hc E -1.005000 -1.492000
1.953000 0.061649
23 C4 c3 M -2.523000 -0.026000
1.531000 -0.082352
24 H5 hc E -1.848000 0.821000
1.607000 0.001243
25 H6 hc E -3.225000 0.201000
0.729000 0.001243
26 C5 c3 M -3.294000 -0.180000
2.844000 0.002811
27 H7 hc E -3.993000 -1.008000
2.775000 0.019027
28 H8 hc E -2.602000 -0.422000
3.647000 0.019027
29 C6 c3 M -4.071000 1.081000
3.224000 0.508926
30 H9 h1 E -3.386000 1.918000
3.320000 -0.047444
31 H10 h1 E -4.760000 1.328000
2.422000 -0.047444
32 N2 n2 M -4.874000 0.833000
4.402000 -0.788587
33 C9 c2 M -4.685000 1.413000
5.494000 0.489963
34 H18 h4 E -5.358000 1.133000
6.295000 0.003173
35 C10 c3 M -3.670000 2.455000
5.901000 -0.418044
36 H19 hc E -3.201000 2.150000
6.831000 0.112679
37 H20 hc E -2.894000 2.629000
5.170000 0.112679
38 H21 hc E -4.176000 3.398000
6.096000 0.112679

LOOP

IMPROPER
C1 N3 C3 O1
C3 C8 N3 H14
C10 H18 C9 N2

DONE
STOP

Lys249.frcmod

remark goes here
MASS
n7 14.010 0.530
hn 1.008 0.161
c3 12.010 0.878
h1 1.008 0.135
h1 1.008 0.135
h1 1.008 0.135
c3 12.010 0.878
h1 1.008 0.135
c 12.010 0.616
o 16.000 0.434
ns 14.010 0.530
hn 1.008 0.161
c3 12.010 0.878
h1 1.008 0.135
h1 1.008 0.135
h1 1.008 0.135
c3 12.010 0.878
hc 1.008 0.135
hc 1.008 0.135
c3 12.010 0.878
hc 1.008 0.135
hc 1.008 0.135
c3 12.010 0.878
hc 1.008 0.135
hc 1.008 0.135
c3 12.010 0.878
h1 1.008 0.135
h1 1.008 0.135
n2 14.010 0.530
c2 12.010 0.360
h4 1.008 0.135
c3 12.010 0.878
hc 1.008 0.135
hc 1.008 0.135
hc 1.008 0.135

BOND
n7-hn 511.28 1.019 same as hn-n3
n7-c3 261.19 1.465 same as c3-n3
n7-c3 261.19 1.465
c3-h1 375.92 1.097
c3-h1 375.92 1.097
c3-h1 375.92 1.097
c3-h1 375.92 1.097
c3-c 243.22 1.524
c3-c3 232.52 1.538
c -o 652.57 1.218
c -ns 356.21 1.379 same as c -n
ns-hn 527.31 1.013 same as hn-n
ns-c3 263.77 1.462 same as c3-n
c3-h1 375.92 1.097
c3-h1 375.92 1.097
c3-h1 375.92 1.097
c3-hc 375.92 1.097
c3-hc 375.92 1.097
c3-c3 232.52 1.538
c3-hc 375.92 1.097
c3-hc 375.92 1.097
c3-c3 232.52 1.538
c3-hc 375.92 1.097
c3-hc 375.92 1.097
c3-c3 232.52 1.538
c3-h1 375.92 1.097
c3-h1 375.92 1.097
c3-n2 259.91 1.466
n2-c2 518.67 1.282
c2-h4 394.23 1.087
c2-c3 255.56 1.510
c3-hc 375.92 1.097
c3-hc 375.92 1.097
c3-hc 375.92 1.097

ANGLE
n7-c3-h1 61.163 109.880 same as h1-c3-n3
n7-c3-h1 61.163 109.880
n7-c3-h1 61.163 109.880
n7-c3-h1 61.163 109.880
n7-c3-h1 61.163 109.880
n7-c3-h1 61.163 109.880
n7-c3-h1 61.163 109.880
n7-c3-c 83.673 111.140 same as c -c3-n3
n7-c3-c3 83.305 111.040 same as c3-c3-n3

hn-n7-c3 47.782 109.290 same as c3-n3-hn
hn-n7-c3 47.782 109.290
hn-n7-c3 47.782 109.290
c3-n7-hn 47.782 109.290
c3-n7-hn 47.782 109.290
c3-n7-c3 65.697 112.350 same as c3-n3-c3
h1-c3-n7 61.163 109.880
h1-c3-n7 61.163 109.880
h1-c3-h1 38.802 108.460
h1-c3-h1 38.802 108.460
h1-c3-h1 38.802 108.460
h1-c3-h1 38.802 108.460
h1-c3-n7 61.163 109.880
h1-c3-n7 61.163 109.880
h1-c3-h1 38.802 108.460
h1-c3-h1 38.802 108.460
h1-c3-h1 38.802 108.460
h1-c3-h1 38.802 108.460
h1-c3-n7 61.163 109.880
h1-c3-n7 61.163 109.880
h1-c3-h1 38.802 108.460
h1-c3-h1 38.802 108.460
h1-c3-h1 38.802 108.460
h1-c3-h1 38.802 108.460
c3-n7-hn 47.782 109.290
c3-n7-hn 47.782 109.290
c3-n7-c3 65.697 112.350
c3-n7-c3 65.697 112.350
c3-c -o 84.552 123.200
c3-c -o 84.552 123.200
c3-c -ns 84.266 115.180 same as c3-c -n
c3-c3-hc 46.816 109.800
c3-c3-hc 46.816 109.800
c3-c3-hc 46.816 109.800
c3-c3-hc 46.816 109.800
c3-c3-c3 64.888 111.510
c3-c3-c3 64.888 111.510
h1-c3-n7 61.163 109.880
h1-c3-n7 61.163 109.880
h1-c3-c 47.531 108.220
h1-c3-c 47.531 108.220
h1-c3-c3 46.868 109.560
h1-c3-c3 46.868 109.560
c -c3-n7 83.673 111.140
c -c3-n7 83.673 111.140
c -c3-h1 47.531 108.220
c -c3-h1 47.531 108.220
c -c3-c3 65.307 111.040
c -c3-c3 65.307 111.040
c -ns-hn 48.691 117.550 same as c -n -hn
c -ns-c3 65.252 120.690 same as c -n -c3
o -c -c3 84.552 123.200
o -c -c3 84.552 123.200
o -c -ns 113.811 123.050 same as n -c -o
ns-c -c3 84.266 115.180
ns-c -c3 84.266 115.180
ns-c -o 113.811 123.050
ns-c -o 113.811 123.050

ns-c3-h1 61.544 108.880 same as h1-c3-n
ns-c3-h1 61.544 108.880
ns-c3-h1 61.544 108.880
ns-c3-h1 61.544 108.880
ns-c3-h1 61.544 108.880
hn-ns-c 48.691 117.550
hn-ns-c 48.691 117.550
hn-ns-c3 46.147 117.680 same as c3-n -hn
c3-ns-c 65.252 120.690
c3-ns-c 65.252 120.690
c3-ns-hn 46.147 117.680
c3-ns-hn 46.147 117.680
h1-c3-ns 61.544 108.880
h1-c3-ns 61.544 108.880
h1-c3-h1 38.802 108.460
h1-c3-h1 38.802 108.460
h1-c3-h1 38.802 108.460
h1-c3-h1 38.802 108.460
h1-c3-ns 61.544 108.880
h1-c3-ns 61.544 108.880
h1-c3-h1 38.802 108.460
h1-c3-h1 38.802 108.460
h1-c3-h1 38.802 108.460
h1-c3-h1 38.802 108.460
h1-c3-ns 61.544 108.880
h1-c3-ns 61.544 108.880
h1-c3-h1 38.802 108.460
h1-c3-h1 38.802 108.460
h1-c3-h1 38.802 108.460
h1-c3-h1 38.802 108.460
c3-c3-n7 83.305 111.040
c3-c3-n7 83.305 111.040
c3-c3-h1 46.868 109.560
c3-c3-h1 46.868 109.560
c3-c3-c 65.307 111.040
c3-c3-c 65.307 111.040
c3-c3-hc 46.816 109.800
c3-c3-hc 46.816 109.800
c3-c3-hc 46.816 109.800
c3-c3-hc 46.816 109.800
c3-c3-c3 64.888 111.510
c3-c3-c3 64.888 111.510
hc-c3-c3 46.816 109.800
hc-c3-c3 46.816 109.800
hc-c3-hc 38.960 107.580
hc-c3-hc 38.960 107.580
hc-c3-c3 46.816 109.800
hc-c3-c3 46.816 109.800
hc-c3-c3 46.816 109.800
hc-c3-c3 46.816 109.800
hc-c3-hc 38.960 107.580
hc-c3-hc 38.960 107.580
hc-c3-c3 46.816 109.800
hc-c3-c3 46.816 109.800
c3-c3-c3 64.888 111.510
c3-c3-c3 64.888 111.510
c3-c3-hc 46.816 109.800
c3-c3-hc 46.816 109.800
c3-c3-hc 46.816 109.800
c3-c3-hc 46.816 109.800
c3-c3-hc 46.816 109.800

c3-c3-hc 46.816 109.800
c3-c3-hc 46.816 109.800
c3-c3-hc 46.816 109.800
c3-c3-c3 64.888 111.510
c3-c3-c3 64.888 111.510
hc-c3-c3 46.816 109.800
hc-c3-c3 46.816 109.800
hc-c3-hc 38.960 107.580
hc-c3-hc 38.960 107.580
hc-c3-c3 46.816 109.800
hc-c3-c3 46.816 109.800
hc-c3-c3 46.816 109.800
hc-c3-c3 46.816 109.800
hc-c3-hc 38.960 107.580
hc-c3-hc 38.960 107.580
hc-c3-c3 46.816 109.800
hc-c3-c3 46.816 109.800
c3-c3-c3 64.888 111.510
c3-c3-c3 64.888 111.510
c3-c3-hc 46.816 109.800
c3-c3-hc 46.816 109.800
c3-c3-hc 46.816 109.800
c3-c3-hc 46.816 109.800
c3-c3-h1 46.868 109.560
c3-c3-h1 46.868 109.560
c3-c3-h1 46.868 109.560
c3-c3-h1 46.868 109.560
c3-c3-n2 84.123 108.800
c3-c3-n2 84.123 108.800
hc-c3-c3 46.816 109.800
hc-c3-c3 46.816 109.800
hc-c3-hc 38.960 107.580
hc-c3-hc 38.960 107.580
hc-c3-c3 46.816 109.800
hc-c3-c3 46.816 109.800
hc-c3-c3 46.816 109.800
hc-c3-c3 46.816 109.800
hc-c3-hc 38.960 107.580
hc-c3-hc 38.960 107.580
hc-c3-c3 46.816 109.800
hc-c3-c3 46.816 109.800
c3-c3-c3 64.888 111.510
c3-c3-c3 64.888 111.510
c3-c3-hc 46.816 109.800
c3-c3-hc 46.816 109.800
c3-c3-hc 46.816 109.800
c3-c3-hc 46.816 109.800
c3-n2-c2 68.517 115.300
c3-n2-c2 68.517 115.300
h1-c3-c3 46.868 109.560
h1-c3-c3 46.868 109.560
h1-c3-h1 38.802 108.460
h1-c3-h1 38.802 108.460
h1-c3-n2 61.133 109.810
h1-c3-n2 61.133 109.810
h1-c3-c3 46.868 109.560
h1-c3-c3 46.868 109.560
h1-c3-h1 38.802 108.460
h1-c3-h1 38.802 108.460
h1-c3-n2 61.133 109.810
h1-c3-n2 61.133 109.810
n2-c3-c3 84.123 108.800
n2-c3-c3 84.123 108.800

n2-c3-h1 61.133 109.810
n2-c3-h1 61.133 109.810
n2-c3-h1 61.133 109.810
n2-c3-h1 61.133 109.810
n2-c2-h4 64.810 120.990
n2-c2-h4 64.810 120.990
n2-c2-c3 83.960 123.430
n2-c2-c3 83.960 123.430
c2-n2-c3 68.517 115.300
c2-n2-c3 68.517 115.300
c2-c3-hc 47.475 110.360
c2-c3-hc 47.475 110.360
c2-c3-hc 47.475 110.360
c2-c3-hc 47.475 110.360
c2-c3-hc 47.475 110.360
c2-c3-hc 47.475 110.360
h4-c2-n2 64.810 120.990
h4-c2-n2 64.810 120.990
h4-c2-c3 45.760 119.020
h4-c2-c3 45.760 119.020
c3-c2-n2 83.960 123.430
c3-c2-n2 83.960 123.430
c3-c2-h4 45.760 119.020
c3-c2-h4 45.760 119.020
hc-c3-c2 47.475 110.360
hc-c3-c2 47.475 110.360
hc-c3-hc 38.960 107.580
hc-c3-hc 38.960 107.580
hc-c3-hc 38.960 107.580
hc-c3-hc 38.960 107.580
hc-c3-c2 47.475 110.360
hc-c3-c2 47.475 110.360
hc-c3-hc 38.960 107.580
hc-c3-hc 38.960 107.580
hc-c3-hc 38.960 107.580
hc-c3-hc 38.960 107.580
hc-c3-c2 47.475 110.360
hc-c3-c2 47.475 110.360
hc-c3-hc 38.960 107.580
hc-c3-hc 38.960 107.580
hc-c3-hc 38.960 107.580
hc-c3-hc 38.960 107.580

DIHE
n7-c3-c -o 1 0.000 180.000 2.000
n7-c3-c -o 1 0.000 180.000 2.000
n7-c3-c -ns 1 0.000 180.000 2.000
n7-c3-c -ns 1 0.000 180.000 2.000
n7-c3-c3-hc 1 0.156 0.000 3.000
n7-c3-c3-hc 1 0.156 0.000 3.000
n7-c3-c3-hc 1 0.156 0.000 3.000
n7-c3-c3-hc 1 0.156 0.000 3.000
n7-c3-c3-c3 1 0.156 0.000 3.000
n7-c3-c3-c3 1 0.156 0.000 3.000
hn-n7-c3-h1 1 0.300 0.000 3.000 same as X -c3-n3-X
hn-n7-c3-h1 1 0.300 0.000 3.000
hn-n7-c3-h1 1 0.300 0.000 3.000
hn-n7-c3-h1 1 0.300 0.000 3.000
hn-n7-c3-h1 1 0.300 0.000 3.000
hn-n7-c3-h1 1 0.300 0.000 3.000
hn-n7-c3-h1 1 0.300 0.000 3.000
hn-n7-c3-c 1 0.300 0.000 3.000 same as X -c3-n3-X
hn-n7-c3-c3 1 0.217 0.000 3.000 same as hn-n3-c3-c3
c3-n7-c3-h1 1 0.225 0.000 3.000 same as h1-c3-n3-c3
c3-n7-c3-c 1 0.300 0.000 3.000 same as X -c3-n3-X
c3-n7-c3-c3 1 0.020 180.000 -3.000
c3-n7-c3-c3 1 0.050 0.000 2.000
c3-n7-c3-c3 1 0.020 180.000 -3.000
c3-n7-c3-c3 1 0.050 0.000 2.000
h1-c3-n7-hn 1 0.300 0.000 3.000
h1-c3-n7-hn 1 0.300 0.000 3.000
h1-c3-n7-c3 1 0.225 0.000 3.000
h1-c3-n7-c3 1 0.225 0.000 3.000
h1-c3-n7-hn 1 0.300 0.000 3.000
h1-c3-n7-hn 1 0.300 0.000 3.000
h1-c3-n7-c3 1 0.225 0.000 3.000
h1-c3-n7-c3 1 0.225 0.000 3.000
h1-c3-n7-hn 1 0.300 0.000 3.000
h1-c3-n7-hn 1 0.300 0.000 3.000
h1-c3-n7-c3 1 0.225 0.000 3.000
h1-c3-n7-c3 1 0.225 0.000 3.000
c3-n7-c3-h1 1 0.225 0.000 3.000
c3-n7-c3-h1 1 0.225 0.000 3.000
c3-n7-c3-h1 1 0.225 0.000 3.000
c3-n7-c3-h1 1 0.225 0.000 3.000
c3-n7-c3-h1 1 0.225 0.000 3.000
c3-n7-c3-h1 1 0.225 0.000 3.000
c3-c -ns-hn 1 2.500 180.000 2.000 same as X -c -n -X
c3-c -ns-c3 1 0.260 180.000 -2.000 same as c3-n -c -c3
c3-c -ns-c3 1 0.500 0.000 1.000 same as c3-n -c -c3
c3-c3-c3-hc 1 0.080 0.000 3.000
c3-c3-c3-hc 1 0.080 0.000 3.000
c3-c3-c3-hc 1 0.080 0.000 3.000
c3-c3-c3-hc 1 0.080 0.000 3.000
c3-c3-c3-c3 1 0.130 0.000 -3.000
c3-c3-c3-c3 1 0.290 180.000 -2.000
c3-c3-c3-c3 1 0.110 0.000 1.000
c3-c3-c3-c3 1 0.130 0.000 -3.000
c3-c3-c3-c3 1 0.290 180.000 -2.000
c3-c3-c3-c3 1 0.110 0.000 1.000
h1-c3-n7-hn 1 0.300 0.000 3.000
h1-c3-n7-hn 1 0.300 0.000 3.000
h1-c3-n7-c3 1 0.225 0.000 3.000
h1-c3-n7-c3 1 0.225 0.000 3.000
h1-c3-c -o 1 0.800 0.000 -1.000
h1-c3-c -o 1 0.080 180.000 3.000
h1-c3-c -o 1 0.800 0.000 -1.000
h1-c3-c -o 1 0.080 180.000 3.000
h1-c3-c -ns 1 0.000 180.000 2.000
h1-c3-c -ns 1 0.000 180.000 2.000
h1-c3-c3-hc 1 0.156 0.000 3.000
h1-c3-c3-hc 1 0.156 0.000 3.000
h1-c3-c3-hc 1 0.156 0.000 3.000
h1-c3-c3-hc 1 0.156 0.000 3.000
h1-c3-c3-c3 1 0.156 0.000 3.000
h1-c3-c3-c3 1 0.156 0.000 3.000

c -c3-n7-hn 1 0.300 0.000
3.000
c -c3-n7-hn 1 0.300 0.000
3.000
c -c3-n7-c3 1 0.300 0.000
3.000
c -c3-n7-c3 1 0.300 0.000
3.000
c -c3-c3-hc 1 0.156 0.000
3.000
c -c3-c3-hc 1 0.156 0.000
3.000
c -c3-c3-hc 1 0.156 0.000
3.000
c -c3-c3-hc 1 0.156 0.000
3.000
c -c3-c3-c3 1 0.100 0.000 3.000
c -c3-c3-c3 1 0.100 0.000 3.000
c -ns-c3-h1 1 0.000 180.000
2.000 same as h1-c3-n -c
c -ns-c3-h1 1 0.000 180.000
2.000
c -ns-c3-h1 1 0.000 180.000
2.000
c -ns-c3-h1 1 0.000 180.000
2.000
c -ns-c3-h1 1 0.000 180.000
2.000
o -c -c3-n7 1 0.000 180.000
2.000
o -c -c3-n7 1 0.000 180.000
2.000
o -c -c3-h1 1 0.800 0.000 -1.000
o -c -c3-h1 1 0.080 180.000
3.000
o -c -c3-h1 1 0.800 0.000 -1.000
o -c -c3-h1 1 0.080 180.000
3.000
o -c -c3-c3 1 0.270 180.000
2.000
o -c -c3-c3 1 0.270 180.000
2.000
o -c -ns-hn 1 2.500 180.000 -
2.000 same as hn-n -c -o
o -c -ns-hn 1 2.000 0.000 1.000
same as hn-n -c -o
o -c -ns-c3 1 2.500 180.000
2.000 same as X -c -n -X
ns-c -c3-n7 1 0.000 180.000
2.000
ns-c -c3-n7 1 0.000 180.000
2.000
ns-c -c3-h1 1 0.000 180.000
2.000
ns-c -c3-h1 1 0.000 180.000
2.000
ns-c -c3-c3 1 0.000 180.000
2.000
ns-c -c3-c3 1 0.000 180.000
2.000
hn-ns-c -c3 1 2.500 180.000
2.000

hn-ns-c -c3 1 2.500 180.000
2.000
hn-ns-c -o 1 2.500 180.000 -
2.000
hn-ns-c -o 1 2.000 0.000 1.000
hn-ns-c -o 1 2.500 180.000 -
2.000
hn-ns-c -o 1 2.000 0.000 1.000
hn-ns-c3-h1 1 0.000 0.000
2.000 same as X -c3-n -X
hn-ns-c3-h1 1 0.000 0.000
2.000
hn-ns-c3-h1 1 0.000 0.000
2.000
hn-ns-c3-h1 1 0.000 0.000
2.000
hn-ns-c3-h1 1 0.000 0.000
2.000
c3-ns-c -c3 1 0.260 180.000 -
2.000
c3-ns-c -c3 1 0.500 0.000 1.000
c3-ns-c -c3 1 0.260 180.000 -
2.000
c3-ns-c -c3 1 0.500 0.000 1.000
c3-ns-c -o 1 2.500 180.000
2.000
c3-ns-c -o 1 2.500 180.000
2.000
h1-c3-ns-c 1 0.000 180.000
2.000
h1-c3-ns-c 1 0.000 180.000
2.000
h1-c3-ns-hn 1 0.000 0.000
2.000
h1-c3-ns-hn 1 0.000 0.000
2.000
h1-c3-ns-c 1 0.000 180.000
2.000
h1-c3-ns-c 1 0.000 180.000
2.000
h1-c3-ns-hn 1 0.000 0.000
2.000
h1-c3-ns-hn 1 0.000 0.000
2.000
h1-c3-ns-c 1 0.000 180.000
2.000
h1-c3-ns-c 1 0.000 180.000
2.000
h1-c3-ns-hn 1 0.000 0.000
2.000
h1-c3-ns-hn 1 0.000 0.000
2.000
c3-c3-n7-hn 1 0.217 0.000
3.000
c3-c3-n7-hn 1 0.217 0.000
3.000
c3-c3-n7-c3 1 0.020 180.000 -
3.000
c3-c3-n7-c3 1 0.050 0.000
2.000
c3-c3-n7-c3 1 0.020 180.000 -
3.000

c3-c3-n7-c3 1 0.050 0.000
2.000
c3-c3-c -o 1 0.270 180.000
2.000
c3-c3-c -o 1 0.270 180.000
2.000
c3-c3-c -ns 1 0.000 180.000
2.000
c3-c3-c -ns 1 0.000 180.000
2.000
c3-c3-c3-hc 1 0.080 0.000
3.000
c3-c3-c3-hc 1 0.080 0.000
3.000
c3-c3-c3-hc 1 0.080 0.000
3.000
c3-c3-c3-hc 1 0.080 0.000
3.000
c3-c3-c3-c3 1 0.130 0.000 -
3.000
c3-c3-c3-c3 1 0.290 180.000 -
2.000
c3-c3-c3-c3 1 0.110 0.000
1.000
c3-c3-c3-c3 1 0.130 0.000 -
3.000
c3-c3-c3-c3 1 0.290 180.000 -
2.000
c3-c3-c3-c3 1 0.110 0.000
1.000
hc-c3-c3-n7 1 0.156 0.000
3.000
hc-c3-c3-n7 1 0.156 0.000
3.000
hc-c3-c3-h1 1 0.156 0.000
3.000
hc-c3-c3-h1 1 0.156 0.000
3.000
hc-c3-c3-c 1 0.156 0.000 3.000
hc-c3-c3-c 1 0.156 0.000 3.000
hc-c3-c3-hc 1 0.120 0.000
3.000
hc-c3-c3-hc 1 0.120 0.000
3.000
hc-c3-c3-hc 1 0.120 0.000
3.000
hc-c3-c3-hc 1 0.120 0.000
3.000
hc-c3-c3-c3 1 0.080 0.000
3.000
hc-c3-c3-c3 1 0.080 0.000
3.000
hc-c3-c3-n7 1 0.156 0.000
3.000
hc-c3-c3-n7 1 0.156 0.000
3.000
hc-c3-c3-h1 1 0.156 0.000
3.000
hc-c3-c3-h1 1 0.156 0.000
3.000
hc-c3-c3-c 1 0.156 0.000 3.000
hc-c3-c3-c 1 0.156 0.000 3.000

hc-c3-c3-hc 1 0.120 0.000
3.000
hc-c3-c3-hc 1 0.120 0.000
3.000
hc-c3-c3-hc 1 0.120 0.000
3.000
hc-c3-c3-hc 1 0.120 0.000
3.000
hc-c3-c3-c3 1 0.080 0.000
3.000
hc-c3-c3-c3 1 0.080 0.000
3.000
c3-c3-c3-n7 1 0.156 0.000
3.000
c3-c3-c3-n7 1 0.156 0.000
3.000
c3-c3-c3-h1 1 0.156 0.000
3.000
c3-c3-c3-h1 1 0.156 0.000
3.000
c3-c3-c3-c 1 0.100 0.000 3.000
c3-c3-c3-c 1 0.100 0.000 3.000
c3-c3-c3-h1 1 0.156 0.000
3.000
c3-c3-c3-h1 1 0.156 0.000
3.000
c3-c3-c3-h1 1 0.156 0.000
3.000
c3-c3-c3-h1 1 0.156 0.000
3.000
c3-c3-c3-n2 1 0.156 0.000
3.000
c3-c3-c3-n2 1 0.156 0.000
3.000
hc-c3-c3-c3 1 0.080 0.000
3.000
hc-c3-c3-c3 1 0.080 0.000
3.000
hc-c3-c3-hc 1 0.120 0.000
3.000
hc-c3-c3-hc 1 0.120 0.000
3.000
hc-c3-c3-hc 1 0.120 0.000
3.000
hc-c3-c3-hc 1 0.120 0.000
3.000
hc-c3-c3-hc 1 0.120 0.000
3.000
hc-c3-c3-hc 1 0.120 0.000
3.000
hc-c3-c3-hc 1 0.120 0.000
3.000
hc-c3-c3-hc 1 0.120 0.000
3.000
hc-c3-c3-c3 1 0.080 0.000
3.000
hc-c3-c3-c3 1 0.080 0.000
3.000
hc-c3-c3-c3 1 0.080 0.000
3.000
hc-c3-c3-c3 1 0.080 0.000
3.000

hc-c3-c3-hc 1 0.120 0.000
3.000
hc-c3-c3-hc 1 0.120 0.000
3.000
hc-c3-c3-hc 1 0.120 0.000
3.000
hc-c3-c3-hc 1 0.120 0.000
3.000
hc-c3-c3-hc 1 0.120 0.000
3.000
hc-c3-c3-hc 1 0.120 0.000
3.000
hc-c3-c3-hc 1 0.120 0.000
3.000
hc-c3-c3-hc 1 0.120 0.000
3.000
hc-c3-c3-c3 1 0.080 0.000
3.000
hc-c3-c3-c3 1 0.080 0.000
3.000
c3-c3-c3-c3 1 0.130 0.000 -
3.000
c3-c3-c3-c3 1 0.290 180.000 -
2.000
c3-c3-c3-c3 1 0.110 0.000
1.000
c3-c3-c3-c3 1 0.130 0.000 -
3.000
c3-c3-c3-c3 1 0.290 180.000 -
2.000
c3-c3-c3-c3 1 0.110 0.000
1.000
c3-c3-c3-hc 1 0.080 0.000
3.000
c3-c3-c3-hc 1 0.080 0.000
3.000
c3-c3-c3-hc 1 0.080 0.000
3.000
c3-c3-c3-hc 1 0.080 0.000
3.000
c3-c3-n2-c2 1 0.000 0.000
3.000
c3-c3-n2-c2 1 0.000 0.000
3.000
hc-c3-c3-c3 1 0.080 0.000
3.000
hc-c3-c3-c3 1 0.080 0.000
3.000
hc-c3-c3-hc 1 0.120 0.000
3.000
hc-c3-c3-hc 1 0.120 0.000
3.000
hc-c3-c3-hc 1 0.120 0.000
3.000
hc-c3-c3-hc 1 0.120 0.000
3.000
hc-c3-c3-h1 1 0.156 0.000
3.000
hc-c3-c3-h1 1 0.156 0.000
3.000
hc-c3-c3-h1 1 0.156 0.000
3.000

hc-c3-c3-h1 1 0.156 0.000
3.000
hc-c3-c3-n2 1 0.156 0.000
3.000
hc-c3-c3-n2 1 0.156 0.000
3.000
hc-c3-c3-c3 1 0.080 0.000
3.000
hc-c3-c3-c3 1 0.080 0.000
3.000
hc-c3-c3-hc 1 0.120 0.000
3.000
hc-c3-c3-hc 1 0.120 0.000
3.000
hc-c3-c3-hc 1 0.120 0.000
3.000
hc-c3-c3-hc 1 0.120 0.000
3.000
hc-c3-c3-h1 1 0.156 0.000
3.000
hc-c3-c3-h1 1 0.156 0.000
3.000
hc-c3-c3-h1 1 0.156 0.000
3.000
hc-c3-c3-h1 1 0.156 0.000
3.000
hc-c3-c3-n2 1 0.156 0.000
3.000
hc-c3-c3-n2 1 0.156 0.000
3.000
c3-c3-c3-c3 1 0.130 0.000 -
3.000
c3-c3-c3-c3 1 0.290 180.000 -
2.000
c3-c3-c3-c3 1 0.110 0.000
1.000
c3-c3-c3-c3 1 0.130 0.000 -
3.000
c3-c3-c3-c3 1 0.290 180.000 -
2.000
c3-c3-c3-c3 1 0.110 0.000
1.000
c3-c3-c3-hc 1 0.080 0.000
3.000
c3-c3-c3-hc 1 0.080 0.000
3.000
c3-c3-c3-hc 1 0.080 0.000
3.000
c3-c3-c3-hc 1 0.080 0.000
3.000
c3-n2-c2-h4 1 4.150 180.000
2.000
c3-n2-c2-h4 1 4.150 180.000
2.000
c3-n2-c2-c3 1 10.370 180.000
2.000
c3-n2-c2-c3 1 10.370 180.000
2.000
h1-c3-c3-c3 1 0.156 0.000
3.000
h1-c3-c3-c3 1 0.156 0.000
3.000

h1-c3-c3-hc 1 0.156 0.000
3.000
h1-c3-c3-hc 1 0.156 0.000
3.000
h1-c3-c3-hc 1 0.156 0.000
3.000
h1-c3-c3-hc 1 0.156 0.000
3.000
h1-c3-n2-c2 1 0.165 180.000
3.000
h1-c3-n2-c2 1 0.165 180.000
3.000
h1-c3-c3-c3 1 0.156 0.000
3.000
h1-c3-c3-c3 1 0.156 0.000
3.000
h1-c3-c3-hc 1 0.156 0.000
3.000
h1-c3-c3-hc 1 0.156 0.000
3.000
h1-c3-c3-hc 1 0.156 0.000
3.000
h1-c3-c3-hc 1 0.156 0.000
3.000
h1-c3-n2-c2 1 0.165 180.000
3.000
h1-c3-n2-c2 1 0.165 180.000
3.000
n2-c3-c3-c3 1 0.156 0.000
3.000
n2-c3-c3-c3 1 0.156 0.000
3.000
n2-c3-c3-hc 1 0.156 0.000
3.000
n2-c3-c3-hc 1 0.156 0.000
3.000
n2-c3-c3-hc 1 0.156 0.000
3.000
n2-c3-c3-hc 1 0.156 0.000
3.000
n2-c2-c3-hc 1 0.000 0.000
2.000
n2-c2-c3-hc 1 0.000 0.000
2.000
n2-c2-c3-hc 1 0.000 0.000
2.000
n2-c2-c3-hc 1 0.000 0.000
2.000
n2-c2-c3-hc 1 0.000 0.000
2.000
n2-c2-c3-hc 1 0.000 0.000
2.000
c2-n2-c3-c3 1 0.000 0.000
3.000
c2-n2-c3-c3 1 0.000 0.000
3.000
c2-n2-c3-h1 1 0.165 180.000
3.000
c2-n2-c3-h1 1 0.165 180.000
3.000
c2-n2-c3-h1 1 0.165 180.000
3.000
c2-n2-c3-h1 1 0.165 180.000
3.000
h4-c2-n2-c3 1 4.150 180.000
2.000
h4-c2-n2-c3 1 4.150 180.000
2.000
h4-c2-c3-hc 1 0.000 0.000
2.000
h4-c2-c3-hc 1 0.000 0.000
2.000
h4-c2-c3-hc 1 0.000 0.000
2.000
h4-c2-c3-hc 1 0.000 0.000
2.000
h4-c2-c3-hc 1 0.000 0.000
2.000
h4-c2-c3-hc 1 0.000 0.000
2.000
c3-c2-n2-c3 1 10.370 180.000
2.000
c3-c2-n2-c3 1 10.370 180.000
2.000
hc-c3-c2-n2 1 0.000 0.000
2.000
hc-c3-c2-n2 1 0.000 0.000
2.000
hc-c3-c2-h4 1 0.000 0.000
2.000
hc-c3-c2-h4 1 0.000 0.000
2.000
hc-c3-c2-n2 1 0.000 0.000
2.000
hc-c3-c2-n2 1 0.000 0.000
2.000
hc-c3-c2-h4 1 0.000 0.000
2.000
hc-c3-c2-h4 1 0.000 0.000
2.000
hc-c3-c2-n2 1 0.000 0.000
2.000
hc-c3-c2-n2 1 0.000 0.000
2.000
hc-c3-c2-h4 1 0.000 0.000
2.000
hc-c3-c2-h4 1 0.000 0.000
2.000

IMPROPER
c3-ns-c -o 10.5 180.0 2.0
General improper torsional angle (2 general atom types)
c -c3-ns-hn 1.1 180.0 2.0
Using default value
c3-h4-c2-n2 1.1 180.0 2.0
Using default value

NONBON
n7 1.9686 0.0522
hn 0.6210 0.0100
c3 1.9069 0.1078
h1 1.3593 0.0208
h1 1.3593 0.0208
h1 1.3593 0.0208
c3 1.9069 0.1078
h1 1.3593 0.0208
c 1.8606 0.0988
o 1.7107 0.1463
ns 1.8352 0.1174
hn 0.6210 0.0100
c3 1.9069 0.1078
h1 1.3593 0.0208
h1 1.3593 0.0208
h1 1.3593 0.0208
c3 1.9069 0.1078
hc 1.4593 0.0208
hc 1.4593 0.0208
c3 1.9069 0.1078
hc 1.4593 0.0208
hc 1.4593 0.0208
c3 1.9069 0.1078
hc 1.4593 0.0208
hc 1.4593 0.0208
c3 1.9069 0.1078
h1 1.3593 0.0208
h1 1.3593 0.0208
n2 1.8993 0.0941
c2 1.8606 0.0988
h4 1.4235 0.0161
c3 1.9069 0.1078
hc 1.4593 0.0208
hc 1.4593 0.0208
hc 1.4593 0.0208

30.prepc

0 0 2

This is a remark line
molecule.res
MOL XYZ 0
CHANGE OMIT DU BEG
0.0000
1 DUMM DU M 999.000 999.0
-999.0 .00000
2 DUMM DU M 999.000 -999.0
999.0 .00000
3 DUMM DU M -999.000 999.0
999.0 .00000
4 C7 cc M -0.197000 0.490000
0.295000 -0.102782
5 C1 ca S -1.528000 -0.068000
0.176000 -0.038632
6 C2 ca B -1.382000 -1.428000
-0.109000 0.299197
7 C3 ca B -2.480000 -2.262000
-0.263000 -0.276775
8 C4 ca B -3.735000 -1.709000
-0.131000 -0.339005
9 C5 ca B -3.896000 -0.344000
0.146000 0.404471
10 C6 ca S -2.807000 0.482000
0.298000 -0.348547
11 H3 ha E -2.963000 1.523000
0.514000 0.241365
12 O1 oh S -5.132000 0.208000
0.275000 -0.657136

13 H15 ho E -5.803000 -0.447000
0.151000 0.450536
14 H2 ha E -4.606000 -2.333000
-0.244000 0.172757
15 H1 ha E -2.366000 -3.309000
-0.481000 0.189560
16 N2 na S -0.034000 -1.694000
-0.201000 -0.555012
17 H14 hn E 0.365000 -2.601000
-0.127000 0.369793
18 C14 c3 3 0.118000 1.947000
0.502000 -0.137808
19 H8 hc E 1.151000 2.066000
0.814000 0.071300
20 H9 hc E -0.493000 2.352000
1.303000 0.071300
21 C15 c3 3 -0.111000 2.793000
-0.754000 0.465466
22 H10 h1 E 0.495000 2.391000
-1.567000 -0.042280
23 H11 h1 E -1.146000 2.709000
-1.064000 -0.042280
24 N1 n8 B 0.158000 4.194000
-0.468000 -1.090122
25 H12 hn E 1.138000 4.340000
-0.311000 0.385805
26 H13 hn E -0.098000 4.770000
-1.246000 0.385805
27 C8 cd M 0.664000 -0.539000
0.089000 0.068449
28 C9 ca M 2.142000 -0.578000
0.098000 -0.136602
29 C11 ca S 2.844000 -1.122000
-0.975000 0.403095
30 H5 h4 E 2.306000 -1.494000
-1.830000 0.042924
31 C10 ca M 2.892000 -0.108000
1.170000 0.138802
32 H4 ha E 2.399000 0.299000
2.034000 0.105103
33 C12 ca M 4.271000 -0.188000
1.116000 -0.507953
34 H6 ha E 4.880000 0.165000
1.927000 0.199121
35 C13 ca M 4.854000 -0.749000
-0.007000 0.424145
36 H7 h4 E 5.924000 -0.833000
-0.082000 0.051636
37 N3 nb M 4.159000 -1.210000
-1.031000 -0.665696

LOOP
C6 C1
C8 N2
N3 C11

IMPROPER
C14 C1 C7 C8
C2 C6 C1 C7
C1 C3 C2 N2
C2 C4 C3 H1
C3 C5 C4 H2
C4 C6 C5 O1
C1 C5 C6 H3
C2 C8 N2 H14
C9 C7 C8 N2
C11 C10 C9 C8
C9 H5 C11 N3
C9 C12 C10 H4
C10 C13 C12 H6
C12 H7 C13 N3

DONE
STOP

30.frcmod

remark goes here
MASS
cc 12.010 0.360
ca 12.010 0.360
ha 1.008 0.135
oh 16.000 0.465
ho 1.008 0.135
na 14.010 0.530
hn 1.008 0.161
c3 12.010 0.878
hc 1.008 0.135
h1 1.008 0.135
n8 14.010 0.530
cd 12.010 0.360
h4 1.008 0.135
nb 14.010 0.530

BOND
cc-ca 308.19 1.456
cc-c3 262.64 1.502
cc-cd 416.13 1.373
ca-ca 378.57 1.398
ca-na 349.52 1.384
ca-ha 395.72 1.086
ca-oh 365.55 1.364
oh-ho 563.51 0.973
na-hn 535.14 1.010
na-cd 354.49 1.380
c3-hc 375.92 1.097
c3-c3 232.52 1.538
c3-h1 375.92 1.097
c3-n8 261.19 1.465 same as c3-n3
n8-hn 511.28 1.019 same as hn-n3
cd-ca 308.19 1.456
ca-h4 390.15 1.089
ca-nb 414.24 1.339

ANGLE
cc-ca-ca 67.122 120.790
cc-c3-hc 47.671 110.490
cc-c3-c3 65.515 111.930
cc-cd-na 92.653 106.990
cc-cd-ca 69.802 113.510
ca-cc-c3 63.318 126.520
ca-cc-cd 69.802 113.510
ca-ca-ca 68.767 120.020
ca-ca-na 87.167 118.340
ca-ca-ha 48.680 119.880
ca-na-hn 46.979 125.540
ca-na-cd 69.377 113.150
ca-ca-oh 87.211 119.900
ca-oh-ho 50.712 108.580
na-cd-ca 83.626 123.450
hn-na-cd 47.101 125.500
c3-cc-cd 66.802 119.450
c3-c3-h1 46.868 109.560
c3-c3-n8 83.305 111.040 same as c3-
c3-n3
hc-c3-hc 38.960 107.580
hc-c3-c3 46.816 109.800
c3-n8-hn 47.782 109.290 same as c3-
n3-hn
h1-c3-h1 38.802 108.460
h1-c3-n8 61.163 109.880 same as h1-
c3-n3
hn-n8-hn 40.828 106.400 same as hn-
n3-hn
cd-ca-ca 67.122 120.790
ca-ca-h4 48.561 120.340
ca-ca-nb 86.849 122.940
ca-nb-ca 70.356 117.220
h4-ca-nb 64.066 116.030

DIHE
cc-ca-ca-ca 1 3.625 180.000
2.000
cc-ca-ca-na 1 3.625 180.000
2.000
cc-ca-ca-ha 1 3.625 180.000
2.000
cc-c3-c3-h1 1 0.156 0.000
3.000
cc-c3-c3-n8 1 0.156 0.000
3.000
cc-cd-na-ca 1 1.700 180.000
2.000
cc-cd-na-hn 1 1.700 180.000
2.000
cc-cd-ca-ca 1 0.700 180.000
2.000 same as X -c2-ca-X
ca-cc-c3-hc 1 0.000 0.000
3.000
ca-cc-c3-c3 1 0.082 0.000
3.000
ca-cc-cd-na 1 4.000 180.000
2.000
ca-cc-cd-ca 1 4.000 180.000
2.000
ca-ca-ca-ca 1 3.625 180.000
2.000
ca-ca-ca-ha 1 3.625 180.000
2.000
ca-ca-na-hn 1 0.300 180.000
2.000
ca-ca-na-cd 1 0.300 180.000
2.000
ca-ca-ca-oh 1 3.625 180.000
2.000
ca-ca-cc-c3 1 0.540 180.000
2.000 same as ca-ca-ce-c3

ca-ca-cc-cd 1 0.700 180.000
2.000 same as X -c2-ca-X
ca-na-cd-ca 1 1.700 180.000
2.000
ca-ca-ca-na 1 3.625 180.000
2.000
ca-ca-oh-ho 1 0.835 180.000
2.000
ha-ca-ca-oh 1 3.625 180.000
2.000
ha-ca-ca-ha 1 3.625 180.000
2.000
ha-ca-ca-na 1 3.625 180.000
2.000
na-cd-cc-c3 1 4.000 180.000
2.000
na-cd-ca-ca 1 0.700 180.000
2.000 same as X -c2-ca-X
hn-na-cd-ca 1 1.700 180.000
2.000
c3-cc-cd-ca 1 4.000 180.000
2.000
c3-c3-n8-hn 1 0.217 0.000
3.000 same as hn-n3-c3-c3
hc-c3-cc-cd 1 0.000 0.000
3.000
hc-c3-c3-h1 1 0.156 0.000
3.000
hc-c3-c3-n8 1 0.156 0.000
3.000
c3-c3-cc-cd 1 0.157 180.000
3.000
h1-c3-n8-hn 1 0.300 0.000
3.000 same as X -c3-n3-X
cd-ca-ca-h4 1 3.625 180.000
2.000
cd-ca-ca-nb 1 3.625 180.000
2.000
cd-ca-ca-ha 1 3.625 180.000
2.000
cd-ca-ca-ca 1 3.625 180.000
2.000
ca-ca-nb-ca 1 4.800 180.000
2.000
ca-nb-ca-h4 1 4.800 180.000
2.000
h4-ca-ca-ca 1 3.625 180.000
2.000
ca-ca-ca-nb 1 3.625 180.000
2.000
ha-ca-ca-h4 1 3.625 180.000
2.000
ha-ca-ca-nb 1 3.625 180.000
2.000

IMPROPER
c3-ca-cc-cd 1.1 180.0 2.0
Using default value
ca-ca-ca-cc 1.1 180.0 2.0
Using default value
ca-ca-ca-na 1.1 180.0 2.0
Using default value
ca-ca-ca-ha 1.1 180.0 2.0
General improper torsional angle (2 general atom types)
ca-ca-ca-oh 1.1 180.0 2.0
Using default value
ca-cd-na-hn 1.1 180.0 2.0
General improper torsional angle (2 general atom types)
ca-cc-cd-na 1.1 180.0 2.0
Using default value
ca-ca-ca-cd 1.1 180.0 2.0
Using default value
ca-h4-ca-nb 1.1 180.0 2.0
Using default value

NONBON
cc 1.8606 0.0988
ca 1.8606 0.0988
ha 1.4735 0.0161
oh 1.8200 0.0930
ho 0.3019 0.0047
na 1.7992 0.2042
hn 0.6210 0.0100
c3 1.9069 0.1078
hc 1.4593 0.0208
h1 1.3593 0.0208
n8 2.0486 0.0323
cd 1.8606 0.0988
h4 1.4235 0.0161
nb 1.8993 0.0941

38.prepc

0 0 2

This is a remark line
molecule.res
MOL XYZ 0
CHANGE OMIT DU BEG
0.0000
1 DUMM DU M 999.000 999.0
-999.0 .00000
2 DUMM DU M 999.000 -999.0
999.0 .00000
3 DUMM DU M -999.000 999.0
999.0 .00000
4 C7 cc M 0.142000 0.623000
0.305000 -0.016375
5 C1 ca S -1.240000 0.202000
0.200000 -0.146450
6 C2 ca B -1.234000 -1.168000
-0.068000 0.309553
7 C3 ca B -2.413000 -1.897000
-0.208000 -0.460921
8 C4 ca B -3.576000 -1.195000
-0.073000 0.246151
9 C5 ca B -3.628000 0.179000
0.188000 0.246515
10 C6 ca S -2.458000 0.882000
0.323000 -0.298042
11 H2 ha E -2.505000 1.935000
0.529000 0.253094
12 O1 oh S -4.820000 0.810000
0.308000 -0.621546
13 H14 ho E -5.526000 0.189000
0.190000 0.454117
14 F1 f E -4.754000 -1.827000 -
0.193000 -0.207828
15 H1 ha E -2.437000 -2.951000
-0.413000 0.239743
16 N2 na S 0.073000 -1.571000
-0.160000 -0.468263
17 H13 hn E 0.380000 -2.515000
-0.113000 0.357948
18 C14 c3 3 0.606000 2.042000
0.495000 -0.142864
19 H7 hc E 1.646000 2.056000
0.803000 0.070449
20 H8 hc E 0.043000 2.516000
1.293000 0.070449
21 C15 c3 3 0.460000 2.894000
-0.769000 0.455314
22 H9 h1 E 1.021000 2.424000
-1.579000 -0.040695
23 H10 h1 E -0.579000 2.911000
-1.078000 -0.040695
24 N1 n8 B 0.869000 4.264000
-0.497000 -1.089594
25 H11 hn E 1.858000 4.313000
-0.342000 0.387843
26 H12 hn E 0.669000 4.855000
-1.281000 0.387843
27 C8 cd M 0.893000 -0.489000
0.106000 -0.004556
28 C9 ca M 2.359000 -0.682000
0.102000 -0.146819
29 C11 ca S 2.991000 -1.295000
-0.977000 0.412726
30 H4 h4 E 2.411000 -1.610000
-1.827000 0.037462
31 C10 ca M 3.162000 -0.290000
1.166000 0.155290
32 H3 ha E 2.722000 0.166000
2.034000 0.101239
33 C12 ca M 4.525000 -0.510000
1.100000 -0.516706
34 H5 ha E 5.174000 -0.221000
1.905000 0.201862
35 C13 ca M 5.037000 -1.130000
-0.027000 0.426869
36 H6 h4 E 6.092000 -1.324000
-0.112000 0.052316
37 N3 nb M 4.289000 -1.518000
-1.044000 -0.665430

LOOP
C6 C1
C8 N2
N3 C11

IMPROPER
C14 C1 C7 C8
C2 C6 C1 C7
C1 C3 C2 N2

C2 C4 C3 H1
C3 C5 C4 F1
C4 C6 C5 O1
C1 C5 C6 H2
C2 C8 N2 H13
C9 C7 C8 N2
C11 C10 C9 C8
C9 H4 C11 N3
C9 C12 C10 H3
C10 C13 C12 H5
C12 H6 C13 N3

DONE
STOP

38.frcmod

remark goes here
MASS
cc 12.010 0.360
ca 12.010 0.360
ca 12.010 0.360
ca 12.010 0.360
ca 12.010 0.360
ca 12.010 0.360
ca 12.010 0.360
ha 1.008 0.135
oh 16.000 0.465
ho 1.008 0.135
f 19.000 0.320
ha 1.008 0.135
na 14.010 0.530
hn 1.008 0.161
c3 12.010 0.878
hc 1.008 0.135
hc 1.008 0.135
c3 12.010 0.878
h1 1.008 0.135
h1 1.008 0.135
n8 14.010 0.530
hn 1.008 0.161
hn 1.008 0.161
cd 12.010 0.360
ca 12.010 0.360
ca 12.010 0.360
h4 1.008 0.135
ca 12.010 0.360
ha 1.008 0.135
ca 12.010 0.360
ha 1.008 0.135
ca 12.010 0.360
h4 1.008 0.135
nb 14.010 0.530

BOND
cc-ca 308.19 1.456
cc-c3 262.64 1.502
cc-cd 416.13 1.373
ca-ca 378.57 1.398
ca-ca 378.57 1.398
ca-ca 378.57 1.398
ca-na 349.52 1.384
ca-ca 378.57 1.398
ca-ha 395.72 1.086
ca-ca 378.57 1.398
ca-f 353.59 1.349
ca-ca 378.57 1.398
ca-oh 365.55 1.364
ca-ha 395.72 1.086
oh-ho 563.51 0.973
na-hn 535.14 1.010
na-cd 354.49 1.380
c3-hc 375.92 1.097
c3-hc 375.92 1.097
c3-c3 232.52 1.538
c3-h1 375.92 1.097
c3-h1 375.92 1.097
c3-n8 261.19 1.465 same as c3-n3
n8-hn 511.28 1.019 same as hn-n3
n8-hn 511.28 1.019
cd-ca 308.19 1.456
ca-ca 378.57 1.398
ca-ca 378.57 1.398
ca-h4 390.15 1.089
ca-nb 414.24 1.339
ca-ha 395.72 1.086
ca-ca 378.57 1.398
ca-ha 395.72 1.086
ca-ca 378.57 1.398
ca-h4 390.15 1.089
ca-nb 414.24 1.339

ANGLE
cc-ca-ca 67.122 120.790
cc-ca-ca 67.122 120.790
cc-ca-ca 67.122 120.790
cc-ca-ca 67.122 120.790
cc-c3-hc 47.671 110.490
cc-c3-hc 47.671 110.490
cc-c3-hc 47.671 110.490
cc-c3-hc 47.671 110.490
cc-c3-c3 65.515 111.930
cc-c3-c3 65.515 111.930
cc-cd-na 92.653 106.990
cc-cd-na 92.653 106.990
cc-cd-ca 69.802 113.510
cc-cd-ca 69.802 113.510
ca-cc-c3 63.318 126.520
ca-cc-c3 63.318 126.520
ca-cc-cd 69.802 113.510
ca-cc-cd 69.802 113.510
ca-ca-ca 68.767 120.020
ca-ca-ca 68.767 120.020
ca-ca-na 87.167 118.340
ca-ca-na 87.167 118.340
ca-ca-ca 68.767 120.020
ca-ca-ca 68.767 120.020
ca-ca-ha 48.680 119.880
ca-ca-ha 48.680 119.880
ca-ca-cc 67.122 120.790
ca-ca-cc 67.122 120.790
ca-ca-ca 68.767 120.020
ca-ca-ca 68.767 120.020
ca-ca-ca 68.767 120.020
ca-ca-ca 68.767 120.020
ca-ca-ha 48.680 119.880
ca-ca-ha 48.680 119.880
ca-na-hn 46.979 125.540
ca-na-hn 46.979 125.540
ca-na-cd 69.377 113.150
ca-na-cd 69.377 113.150
ca-ca-ca 68.767 120.020
ca-ca-ca 68.767 120.020
ca-ca-na 87.167 118.340
ca-ca-na 87.167 118.340
ca-ca-ca 68.767 120.020
ca-ca-ca 68.767 120.020
ca-ca-f 89.261 118.960
ca-ca-f 89.261 118.960
ca-ca-ca 68.767 120.020
ca-ca-ca 68.767 120.020
ca-ca-ha 48.680 119.880
ca-ca-ha 48.680 119.880
ca-ca-ca 68.767 120.020
ca-ca-ca 68.767 120.020
ca-ca-oh 87.211 119.900
ca-ca-oh 87.211 119.900
ca-ca-ca 68.767 120.020
ca-ca-ca 68.767 120.020
ca-ca-f 89.261 118.960
ca-ca-f 89.261 118.960
ca-ca-ca 68.767 120.020
ca-ca-ca 68.767 120.020
ca-ca-ha 48.680 119.880
ca-ca-ha 48.680 119.880
ca-oh-ho 50.712 108.580
ca-oh-ho 50.712 108.580
ca-ca-cc 67.122 120.790
ca-ca-cc 67.122 120.790
ca-ca-ca 68.767 120.020
ca-ca-ca 68.767 120.020
ca-ca-ca 68.767 120.020
ca-ca-ca 68.767 120.020
ca-ca-oh 87.211 119.900
ca-ca-oh 87.211 119.900
ha-ca-ca 48.680 119.880
ha-ca-ca 48.680 119.880
ha-ca-ca 48.680 119.880
ha-ca-ca 48.680 119.880
oh-ca-ca 87.211 119.900
oh-ca-ca 87.211 119.900
oh-ca-ca 87.211 119.900
oh-ca-ca 87.211 119.900
ho-oh-ca 50.712 108.580
ho-oh-ca 50.712 108.580
f -ca-ca 89.261 118.960
f -ca-ca 89.261 118.960
f -ca-ca 89.261 118.960
f -ca-ca 89.261 118.960
ha-ca-ca 48.680 119.880
ha-ca-ca 48.680 119.880
ha-ca-ca 48.680 119.880
ha-ca-ca 48.680 119.880
na-ca-ca 87.167 118.340
na-ca-ca 87.167 118.340
na-ca-ca 87.167 118.340
na-ca-ca 87.167 118.340
na-cd-cc 92.653 106.990
na-cd-cc 92.653 106.990

na-cd-ca 83.626 123.450
na-cd-ca 83.626 123.450
hn-na-ca 46.979 125.540
hn-na-ca 46.979 125.540
hn-na-cd 47.101 125.500
hn-na-cd 47.101 125.500
c3-cc-ca 63.318 126.520
c3-cc-ca 63.318 126.520
c3-cc-cd 66.802 119.450
c3-cc-cd 66.802 119.450
c3-c3-h1 46.868 109.560
c3-c3-h1 46.868 109.560
c3-c3-h1 46.868 109.560
c3-c3-h1 46.868 109.560
c3-c3-n8 83.305 111.040 same as c3-c3-n3
hc-c3-cc 47.671 110.490
hc-c3-cc 47.671 110.490
hc-c3-hc 38.960 107.580
hc-c3-hc 38.960 107.580
hc-c3-c3 46.816 109.800
hc-c3-c3 46.816 109.800
hc-c3-cc 47.671 110.490
hc-c3-cc 47.671 110.490
hc-c3-hc 38.960 107.580
hc-c3-hc 38.960 107.580
hc-c3-c3 46.816 109.800
hc-c3-c3 46.816 109.800
c3-c3-cc 65.515 111.930
c3-c3-cc 65.515 111.930
c3-c3-hc 46.816 109.800
c3-c3-hc 46.816 109.800
c3-c3-hc 46.816 109.800
c3-c3-hc 46.816 109.800
c3-n8-hn 47.782 109.290 same as c3-n3-hn
c3-n8-hn 47.782 109.290
c3-n8-hn 47.782 109.290
h1-c3-c3 46.868 109.560
h1-c3-c3 46.868 109.560
h1-c3-h1 38.802 108.460
h1-c3-h1 38.802 108.460
h1-c3-n8 61.163 109.880 same as h1-c3-n3
h1-c3-c3 46.868 109.560
h1-c3-c3 46.868 109.560
h1-c3-h1 38.802 108.460
h1-c3-h1 38.802 108.460
h1-c3-n8 61.163 109.880
h1-c3-n8 61.163 109.880
n8-c3-c3 83.305 111.040
n8-c3-c3 83.305 111.040
n8-c3-h1 61.163 109.880
n8-c3-h1 61.163 109.880
n8-c3-h1 61.163 109.880
n8-c3-h1 61.163 109.880
hn-n8-c3 47.782 109.290
hn-n8-c3 47.782 109.290
hn-n8-hn 40.828 106.400 same as hn-n3-hn
hn-n8-c3 47.782 109.290
hn-n8-c3 47.782 109.290
hn-n8-hn 40.828 106.400
hn-n8-hn 40.828 106.400
cd-cc-ca 69.802 113.510
cd-cc-ca 69.802 113.510
cd-cc-c3 66.802 119.450
cd-cc-c3 66.802 119.450
cd-na-ca 69.377 113.150
cd-na-ca 69.377 113.150
cd-na-hn 47.101 125.500
cd-na-hn 47.101 125.500
cd-ca-ca 67.122 120.790
cd-ca-ca 67.122 120.790
cd-ca-ca 67.122 120.790
cd-ca-ca 67.122 120.790
ca-cd-cc 69.802 113.510
ca-cd-cc 69.802 113.510
ca-cd-na 83.626 123.450
ca-cd-na 83.626 123.450
ca-ca-h4 48.561 120.340
ca-ca-h4 48.561 120.340
ca-ca-nb 86.849 122.940
ca-ca-nb 86.849 122.940
ca-ca-ha 48.680 119.880
ca-ca-ha 48.680 119.880
ca-ca-ca 68.767 120.020
ca-ca-ca 68.767 120.020
ca-ca-cd 67.122 120.790
ca-ca-cd 67.122 120.790
ca-ca-ca 68.767 120.020
ca-ca-ca 68.767 120.020
ca-nb-ca 70.356 117.220
ca-nb-ca 70.356 117.220
h4-ca-ca 48.561 120.340
h4-ca-ca 48.561 120.340
h4-ca-nb 64.066 116.030
h4-ca-nb 64.066 116.030
ca-ca-cd 67.122 120.790
ca-ca-cd 67.122 120.790
ca-ca-ca 68.767 120.020
ca-ca-ca 68.767 120.020
ca-ca-ha 48.680 119.880
ca-ca-ha 48.680 119.880
ca-ca-ca 68.767 120.020
ca-ca-ca 68.767 120.020
ha-ca-ca 48.680 119.880
ha-ca-ca 48.680 119.880
ha-ca-ca 48.680 119.880
ha-ca-ca 48.680 119.880
ca-ca-ca 68.767 120.020
ca-ca-ca 68.767 120.020
ca-ca-ha 48.680 119.880
ca-ca-ha 48.680 119.880
ca-ca-h4 48.561 120.340
ca-ca-h4 48.561 120.340
ca-ca-nb 86.849 122.940
ca-ca-nb 86.849 122.940
ha-ca-ca 48.680 119.880
ha-ca-ca 48.680 119.880
ha-ca-ca 48.680 119.880
ha-ca-ca 48.680 119.880
ca-ca-ca 68.767 120.020
ca-ca-ca 68.767 120.020
ca-ca-ha 48.680 119.880
ca-ca-ha 48.680 119.880
ca-nb-ca 70.356 117.220
ca-nb-ca 70.356 117.220
h4-ca-ca 48.561 120.340
h4-ca-ca 48.561 120.340
h4-ca-nb 64.066 116.030
h4-ca-nb 64.066 116.030
nb-ca-ca 86.849 122.940
nb-ca-ca 86.849 122.940
nb-ca-h4 64.066 116.030
nb-ca-h4 64.066 116.030
nb-ca-ca 86.849 122.940
nb-ca-ca 86.849 122.940
nb-ca-h4 64.066 116.030
nb-ca-h4 64.066 116.030

DIHE
cc-ca-ca-ca 1 3.625 180.000 2.000
cc-ca-ca-ca 1 3.625 180.000 2.000
cc-ca-ca-na 1 3.625 180.000 2.000
cc-ca-ca-na 1 3.625 180.000 2.000
cc-ca-ca-ca 1 3.625 180.000 2.000
cc-ca-ca-ca 1 3.625 180.000 2.000
cc-ca-ca-ha 1 3.625 180.000 2.000
cc-ca-ca-ha 1 3.625 180.000 2.000
cc-c3-c3-h1 1 0.156 0.000 3.000
cc-c3-c3-h1 1 0.156 0.000 3.000
cc-c3-c3-h1 1 0.156 0.000 3.000
cc-c3-c3-h1 1 0.156 0.000 3.000
cc-c3-c3-n8 1 0.156 0.000 3.000
cc-c3-c3-n8 1 0.156 0.000 3.000
cc-cd-na-ca 1 1.700 180.000 2.000
cc-cd-na-ca 1 1.700 180.000 2.000
cc-cd-na-hn 1 1.700 180.000 2.000
cc-cd-na-hn 1 1.700 180.000 2.000
cc-cd-ca-ca 1 0.700 180.000 2.000 same as X -c2-ca-X
cc-cd-ca-ca 1 0.700 180.000 2.000
cc-cd-ca-ca 1 0.700 180.000 2.000
ca-cc-c3-hc 1 0.000 0.000 3.000
ca-cc-c3-hc 1 0.000 0.000 3.000

ca-cc-c3-hc 1 0.000 0.000 3.000
ca-cc-c3-hc 1 0.000 0.000 3.000
ca-cc-c3-c3 1 0.082 0.000 3.000
ca-cc-c3-c3 1 0.082 0.000 3.000
ca-cc-cd-na 1 4.000 180.000 2.000
ca-cc-cd-na 1 4.000 180.000 2.000
ca-cc-cd-ca 1 4.000 180.000 2.000
ca-cc-cd-ca 1 4.000 180.000 2.000
ca-ca-ca-ca 1 3.625 180.000 2.000
ca-ca-ca-ca 1 3.625 180.000 2.000
ca-ca-ca-ha 1 3.625 180.000 2.000
ca-ca-ca-ha 1 3.625 180.000 2.000
ca-ca-na-hn 1 0.300 180.000 2.000
ca-ca-na-hn 1 0.300 180.000 2.000
ca-ca-na-cd 1 0.300 180.000 2.000
ca-ca-na-cd 1 0.300 180.000 2.000
ca-ca-ca-ca 1 3.625 180.000 2.000
ca-ca-ca-ca 1 3.625 180.000 2.000
ca-ca-ca-oh 1 3.625 180.000 2.000
ca-ca-ca-oh 1 3.625 180.000 2.000
ca-ca-cc-c3 1 0.540 180.000 2.000 same as ca-ca-ce-c3
ca-ca-cc-c3 1 0.540 180.000 2.000
ca-ca-cc-cd 1 0.700 180.000 2.000 same as X -c2-ca-X
ca-ca-ca-ca 1 3.625 180.000 2.000
ca-ca-ca-ca 1 3.625 180.000 2.000
ca-ca-ca-ha 1 3.625 180.000 2.000
ca-ca-ca-ha 1 3.625 180.000 2.000
ca-ca-ca-ca 1 3.625 180.000 2.000
ca-ca-ca-ca 1 3.625 180.000 2.000
ca-ca-ca-f 1 3.625 180.000 2.000
ca-ca-ca-f 1 3.625 180.000 2.000

ca-na-cd-cc 1 1.700 180.000 2.000
ca-na-cd-cc 1 1.700 180.000 2.000
ca-na-cd-ca 1 1.700 180.000 2.000
ca-na-cd-ca 1 1.700 180.000 2.000
ca-ca-ca-cc 1 3.625 180.000 2.000
ca-ca-ca-cc 1 3.625 180.000 2.000
ca-ca-ca-ca 1 3.625 180.000 2.000
ca-ca-ca-ca 1 3.625 180.000 2.000
ca-ca-na-hn 1 0.300 180.000 2.000
ca-ca-na-hn 1 0.300 180.000 2.000
ca-ca-na-cd 1 0.300 180.000 2.000
ca-ca-na-cd 1 0.300 180.000 2.000
ca-ca-ca-ca 1 3.625 180.000 2.000
ca-ca-ca-ca 1 3.625 180.000 2.000
ca-ca-ca-oh 1 3.625 180.000 2.000
ca-ca-ca-oh 1 3.625 180.000 2.000
ca-ca-ca-ca 1 3.625 180.000 2.000
ca-ca-ca-ca 1 3.625 180.000 2.000
ca-ca-ca-na 1 3.625 180.000 2.000
ca-ca-ca-na 1 3.625 180.000 2.000
ca-ca-ca-ca 1 3.625 180.000 2.000
ca-ca-ca-ca 1 3.625 180.000 2.000
ca-ca-ca-ha 1 3.625 180.000 2.000
ca-ca-ca-ha 1 3.625 180.000 2.000
ca-ca-oh-ho 1 0.835 180.000 2.000
ca-ca-oh-ho 1 0.835 180.000 2.000
ca-ca-ca-ca 1 3.625 180.000 2.000
ca-ca-ca-ca 1 3.625 180.000 2.000
ca-ca-ca-ha 1 3.625 180.000 2.000
ca-ca-ca-ha 1 3.625 180.000 2.000
ca-ca-ca-cc 1 3.625 180.000 2.000

ca-ca-ca-cc 1 3.625 180.000 2.000
ca-ca-ca-ca 1 3.625 180.000 2.000
ca-ca-ca-ca 1 3.625 180.000 2.000
ca-ca-cc-c3 1 0.540 180.000 2.000
ca-ca-cc-c3 1 0.540 180.000 2.000
ca-ca-cc-cd 1 0.700 180.000 2.000
ca-ca-cc-cd 1 0.700 180.000 2.000
ca-ca-ca-ca 1 3.625 180.000 2.000
ca-ca-ca-ca 1 3.625 180.000 2.000
ca-ca-ca-na 1 3.625 180.000 2.000
ca-ca-ca-na 1 3.625 180.000 2.000
ca-ca-ca-ca 1 3.625 180.000 2.000
ca-ca-ca-ca 1 3.625 180.000 2.000
ca-ca-ca-f 1 3.625 180.000 2.000
ca-ca-ca-f 1 3.625 180.000 2.000
ca-ca-oh-ho 1 0.835 180.000 2.000
ca-ca-oh-ho 1 0.835 180.000 2.000
ha-ca-ca-cc 1 3.625 180.000 2.000
ha-ca-ca-cc 1 3.625 180.000 2.000
ha-ca-ca-ca 1 3.625 180.000 2.000
ha-ca-ca-ca 1 3.625 180.000 2.000
ha-ca-ca-ca 1 3.625 180.000 2.000
ha-ca-ca-ca 1 3.625 180.000 2.000
ha-ca-ca-oh 1 3.625 180.000 2.000
ha-ca-ca-oh 1 3.625 180.000 2.000
oh-ca-ca-ca 1 3.625 180.000 2.000
oh-ca-ca-ca 1 3.625 180.000 2.000
oh-ca-ca-f 1 3.625 180.000 2.000
oh-ca-ca-f 1 3.625 180.000 2.000
oh-ca-ca-ca 1 3.625 180.000 2.000
oh-ca-ca-ca 1 3.625 180.000 2.000

oh-ca-ca-ha 1 3.625 180.000
2.000
oh-ca-ca-ha 1 3.625 180.000
2.000
ho-oh-ca-ca 1 0.835 180.000
2.000
ho-oh-ca-ca 1 0.835 180.000
2.000
ho-oh-ca-ca 1 0.835 180.000
2.000
ho-oh-ca-ca 1 0.835 180.000
2.000
f -ca-ca-ca 1 3.625 180.000
2.000
f -ca-ca-ca 1 3.625 180.000
2.000
f -ca-ca-ha 1 3.625 180.000
2.000
f -ca-ca-ha 1 3.625 180.000
2.000
f -ca-ca-ca 1 3.625 180.000
2.000
f -ca-ca-ca 1 3.625 180.000
2.000
f -ca-ca-oh 1 3.625 180.000
2.000
f -ca-ca-oh 1 3.625 180.000
2.000
ha-ca-ca-ca 1 3.625 180.000
2.000
ha-ca-ca-ca 1 3.625 180.000
2.000
ha-ca-ca-na 1 3.625 180.000
2.000
ha-ca-ca-na 1 3.625 180.000
2.000
ha-ca-ca-ca 1 3.625 180.000
2.000
ha-ca-ca-ca 1 3.625 180.000
2.000
ha-ca-ca-f 1 3.625 180.000
2.000
ha-ca-ca-f 1 3.625 180.000
2.000
na-ca-ca-cc 1 3.625 180.000
2.000
na-ca-ca-cc 1 3.625 180.000
2.000
na-ca-ca-ca 1 3.625 180.000
2.000
na-ca-ca-ca 1 3.625 180.000
2.000
na-ca-ca-ca 1 3.625 180.000
2.000
na-ca-ca-ca 1 3.625 180.000
2.000
na-ca-ca-ha 1 3.625 180.000
2.000
na-ca-ca-ha 1 3.625 180.000
2.000
na-cd-cc-ca 1 4.000 180.000
2.000

na-cd-cc-ca 1 4.000 180.000
2.000
na-cd-cc-c3 1 4.000 180.000
2.000
na-cd-cc-c3 1 4.000 180.000
2.000
na-cd-ca-ca 1 0.700 180.000
2.000 same as X -c2-ca-X
na-cd-ca-ca 1 0.700 180.000
2.000
na-cd-ca-ca 1 0.700 180.000
2.000
hn-na-ca-ca 1 0.300 180.000
2.000
hn-na-ca-ca 1 0.300 180.000
2.000
hn-na-ca-ca 1 0.300 180.000
2.000
hn-na-ca-ca 1 0.300 180.000
2.000
hn-na-cd-cc 1 1.700 180.000
2.000
hn-na-cd-cc 1 1.700 180.000
2.000
hn-na-cd-ca 1 1.700 180.000
2.000
hn-na-cd-ca 1 1.700 180.000
2.000
c3-cc-ca-ca 1 0.540 180.000
2.000
c3-cc-ca-ca 1 0.540 180.000
2.000
c3-cc-ca-ca 1 0.540 180.000
2.000
c3-cc-ca-ca 1 0.540 180.000
2.000
c3-cc-cd-na 1 4.000 180.000
2.000
c3-cc-cd-na 1 4.000 180.000
2.000
c3-cc-cd-ca 1 4.000 180.000
2.000
c3-cc-cd-ca 1 4.000 180.000
2.000
c3-c3-n8-hn 1 0.217 0.000
3.000 same as hn-n3-c3-c3
c3-c3-n8-hn 1 0.217 0.000
3.000
c3-c3-n8-hn 1 0.217 0.000
3.000
hc-c3-cc-ca 1 0.000 0.000
3.000
hc-c3-cc-ca 1 0.000 0.000
3.000
hc-c3-cc-cd 1 0.000 0.000
3.000
hc-c3-cc-cd 1 0.000 0.000
3.000
hc-c3-c3-h1 1 0.156 0.000
3.000
hc-c3-c3-h1 1 0.156 0.000
3.000

hc-c3-c3-h1 1 0.156 0.000
3.000
hc-c3-c3-h1 1 0.156 0.000
3.000
hc-c3-c3-n8 1 0.156 0.000
3.000
hc-c3-c3-n8 1 0.156 0.000
3.000
hc-c3-cc-ca 1 0.000 0.000
3.000
hc-c3-cc-ca 1 0.000 0.000
3.000
hc-c3-cc-cd 1 0.000 0.000
3.000
hc-c3-cc-cd 1 0.000 0.000
3.000
hc-c3-c3-h1 1 0.156 0.000
3.000
hc-c3-c3-h1 1 0.156 0.000
3.000
hc-c3-c3-h1 1 0.156 0.000
3.000
hc-c3-c3-h1 1 0.156 0.000
3.000
hc-c3-c3-n8 1 0.156 0.000
3.000
hc-c3-c3-n8 1 0.156 0.000
3.000
c3-c3-cc-ca 1 0.082 0.000
3.000
c3-c3-cc-ca 1 0.082 0.000
3.000
c3-c3-cc-cd 1 0.157 180.000
3.000
c3-c3-cc-cd 1 0.157 180.000
3.000
h1-c3-c3-cc 1 0.156 0.000
3.000
h1-c3-c3-cc 1 0.156 0.000
3.000
h1-c3-c3-hc 1 0.156 0.000
3.000
h1-c3-c3-hc 1 0.156 0.000
3.000
h1-c3-c3-hc 1 0.156 0.000
3.000
h1-c3-c3-hc 1 0.156 0.000
3.000
h1-c3-n8-hn 1 0.300 0.000
3.000 same as X -c3-n3-X
h1-c3-n8-hn 1 0.300 0.000
3.000
h1-c3-n8-hn 1 0.300 0.000
3.000
h1-c3-c3-cc 1 0.156 0.000
3.000
h1-c3-c3-cc 1 0.156 0.000
3.000
h1-c3-c3-hc 1 0.156 0.000
3.000
h1-c3-c3-hc 1 0.156 0.000
3.000

h1-c3-c3-hc 1 0.156 0.000
3.000
h1-c3-c3-hc 1 0.156 0.000
3.000
h1-c3-n8-hn 1 0.300 0.000
3.000
h1-c3-n8-hn 1 0.300 0.000
3.000
h1-c3-n8-hn 1 0.300 0.000
3.000
h1-c3-n8-hn 1 0.300 0.000
3.000
n8-c3-c3-cc 1 0.156 0.000
3.000
n8-c3-c3-cc 1 0.156 0.000
3.000
n8-c3-c3-hc 1 0.156 0.000
3.000
n8-c3-c3-hc 1 0.156 0.000
3.000
n8-c3-c3-hc 1 0.156 0.000
3.000
n8-c3-c3-hc 1 0.156 0.000
3.000
hn-n8-c3-c3 1 0.217 0.000
3.000
hn-n8-c3-c3 1 0.217 0.000
3.000
hn-n8-c3-h1 1 0.300 0.000
3.000
hn-n8-c3-h1 1 0.300 0.000
3.000
hn-n8-c3-h1 1 0.300 0.000
3.000
hn-n8-c3-h1 1 0.300 0.000
3.000
hn-n8-c3-c3 1 0.217 0.000
3.000
hn-n8-c3-c3 1 0.217 0.000
3.000
hn-n8-c3-h1 1 0.300 0.000
3.000
hn-n8-c3-h1 1 0.300 0.000
3.000
hn-n8-c3-h1 1 0.300 0.000
3.000
hn-n8-c3-h1 1 0.300 0.000
3.000
cd-cc-ca-ca 1 0.700 180.000
2.000
cd-cc-ca-ca 1 0.700 180.000
2.000
cd-cc-ca-ca 1 0.700 180.000
2.000
cd-cc-ca-ca 1 0.700 180.000
2.000
cd-cc-c3-hc 1 0.000 0.000
3.000
cd-cc-c3-hc 1 0.000 0.000
3.000
cd-cc-c3-hc 1 0.000 0.000
3.000

cd-cc-c3-hc 1 0.000 0.000
3.000
cd-cc-c3-c3 1 0.157 180.000
3.000
cd-cc-c3-c3 1 0.157 180.000
3.000
cd-na-ca-ca 1 0.300 180.000
2.000
cd-na-ca-ca 1 0.300 180.000
2.000
cd-na-ca-ca 1 0.300 180.000
2.000
cd-na-ca-ca 1 0.300 180.000
2.000
cd-ca-ca-h4 1 3.625 180.000
2.000
cd-ca-ca-h4 1 3.625 180.000
2.000
cd-ca-ca-nb 1 3.625 180.000
2.000
cd-ca-ca-nb 1 3.625 180.000
2.000
cd-ca-ca-ha 1 3.625 180.000
2.000
cd-ca-ca-ha 1 3.625 180.000
2.000
cd-ca-ca-ca 1 3.625 180.000
2.000
cd-ca-ca-ca 1 3.625 180.000
2.000
ca-cd-cc-ca 1 4.000 180.000
2.000
ca-cd-cc-ca 1 4.000 180.000
2.000
ca-cd-cc-c3 1 4.000 180.000
2.000
ca-cd-cc-c3 1 4.000 180.000
2.000
ca-cd-na-ca 1 1.700 180.000
2.000
ca-cd-na-ca 1 1.700 180.000
2.000
ca-cd-na-hn 1 1.700 180.000
2.000
ca-cd-na-hn 1 1.700 180.000
2.000
ca-ca-nb-ca 1 4.800 180.000
2.000
ca-ca-nb-ca 1 4.800 180.000
2.000
ca-ca-ca-ha 1 3.625 180.000
2.000
ca-ca-ca-ha 1 3.625 180.000
2.000
ca-ca-ca-ca 1 3.625 180.000
2.000
ca-ca-ca-ca 1 3.625 180.000
2.000
ca-ca-cd-cc 1 0.700 180.000
2.000
ca-ca-cd-cc 1 0.700 180.000
2.000

ca-ca-cd-na 1 0.700 180.000
2.000
ca-ca-cd-na 1 0.700 180.000
2.000
ca-ca-ca-ha 1 3.625 180.000
2.000
ca-ca-ca-ha 1 3.625 180.000
2.000
ca-ca-ca-ca 1 3.625 180.000
2.000
ca-ca-ca-ca 1 3.625 180.000
2.000
ca-nb-ca-ca 1 4.800 180.000
2.000
ca-nb-ca-ca 1 4.800 180.000
2.000
ca-nb-ca-h4 1 4.800 180.000
2.000
ca-nb-ca-h4 1 4.800 180.000
2.000
h4-ca-ca-cd 1 3.625 180.000
2.000
h4-ca-ca-cd 1 3.625 180.000
2.000
h4-ca-ca-ca 1 3.625 180.000
2.000
h4-ca-ca-ca 1 3.625 180.000
2.000
h4-ca-nb-ca 1 4.800 180.000
2.000
h4-ca-nb-ca 1 4.800 180.000
2.000
ca-ca-cd-cc 1 0.700 180.000
2.000
ca-ca-cd-cc 1 0.700 180.000
2.000
ca-ca-cd-na 1 0.700 180.000
2.000
ca-ca-cd-na 1 0.700 180.000
2.000
ca-ca-ca-h4 1 3.625 180.000
2.000
ca-ca-ca-h4 1 3.625 180.000
2.000
ca-ca-ca-nb 1 3.625 180.000
2.000
ca-ca-ca-nb 1 3.625 180.000
2.000
ca-ca-ca-h4 1 3.625 180.000
2.000
ca-ca-ca-h4 1 3.625 180.000
2.000
ca-ca-ca-nb 1 3.625 180.000
2.000
ca-ca-ca-nb 1 3.625 180.000
2.000
ha-ca-ca-cd 1 3.625 180.000
2.000
ha-ca-ca-cd 1 3.625 180.000
2.000
ha-ca-ca-ca 1 3.625 180.000
2.000

ha-ca-ca-ca 1 3.625 180.000 2.000
ha-ca-ca-ha 1 3.625 180.000 2.000
ha-ca-ca-ha 1 3.625 180.000 2.000
ha-ca-ca-ca 1 3.625 180.000 2.000
ha-ca-ca-ca 1 3.625 180.000 2.000
ca-ca-ca-cd 1 3.625 180.000 2.000
ca-ca-ca-cd 1 3.625 180.000 2.000
ca-ca-ca-ca 1 3.625 180.000 2.000
ca-ca-ca-ca 1 3.625 180.000 2.000
ca-ca-nb-ca 1 4.800 180.000 2.000
ca-ca-nb-ca 1 4.800 180.000 2.000
ha-ca-ca-ca 1 3.625 180.000 2.000
ha-ca-ca-ca 1 3.625 180.000 2.000
ha-ca-ca-ha 1 3.625 180.000 2.000
ha-ca-ca-ha 1 3.625 180.000 2.000
ha-ca-ca-h4 1 3.625 180.000 2.000
ha-ca-ca-h4 1 3.625 180.000 2.000
ha-ca-ca-nb 1 3.625 180.000 2.000
ha-ca-ca-nb 1 3.625 180.000 2.000
ca-ca-ca-ca 1 3.625 180.000 2.000
ca-ca-ca-ca 1 3.625 180.000 2.000
ca-ca-ca-ha 1 3.625 180.000 2.000
ca-ca-ca-ha 1 3.625 180.000 2.000
ca-nb-ca-ca 1 4.800 180.000 2.000
ca-nb-ca-ca 1 4.800 180.000 2.000
ca-nb-ca-h4 1 4.800 180.000 2.000
ca-nb-ca-h4 1 4.800 180.000 2.000
h4-ca-ca-ca 1 3.625 180.000 2.000
h4-ca-ca-ca 1 3.625 180.000 2.000
h4-ca-ca-ha 1 3.625 180.000 2.000
h4-ca-ca-ha 1 3.625 180.000 2.000
h4-ca-nb-ca 1 4.800 180.000 2.000
h4-ca-nb-ca 1 4.800 180.000 2.000
nb-ca-ca-cd 1 3.625 180.000 2.000
nb-ca-ca-cd 1 3.625 180.000 2.000
nb-ca-ca-ca 1 3.625 180.000 2.000
nb-ca-ca-ca 1 3.625 180.000 2.000
nb-ca-ca-ca 1 3.625 180.000 2.000
nb-ca-ca-ca 1 3.625 180.000 2.000
nb-ca-ca-ha 1 3.625 180.000 2.000
nb-ca-ca-ha 1 3.625 180.000 2.000

IMPROPER
c3-ca-cc-cd 1.1 180.0 2.0
Using default value
ca-ca-ca-cc 1.1 180.0 2.0
Using default value
ca-ca-ca-na 1.1 180.0 2.0
Using default value
ca-ca-ca-ha 1.1 180.0 2.0
General improper torsional angle (2 general atom types)
ca-ca-ca-f 1.1 180.0 2.0
ca-ca-ca-oh 1.1 180.0 2.0
Using default value
ca-ca-ca-ha 1.1 180.0 2.0
ca-cd-na-hn 1.1 180.0 2.0
General improper torsional angle (2 general atom types)
ca-cc-cd-na 1.1 180.0 2.0
Using default value
ca-ca-ca-cd 1.1 180.0 2.0
Using default value
ca-h4-ca-nb 1.1 180.0 2.0
Using default value
ca-ca-ca-ha 1.1 180.0 2.0
ca-ca-ca-ha 1.1 180.0 2.0
ca-h4-ca-nb 1.1 180.0 2.0

NONBON
cc 1.8606 0.0988
ca 1.8606 0.0988
ca 1.8606 0.0988
ca 1.8606 0.0988
ca 1.8606 0.0988
ca 1.8606 0.0988
ca 1.8606 0.0988
ha 1.4735 0.0161
oh 1.8200 0.0930
ho 0.3019 0.0047
f 1.7029 0.0832
ha 1.4735 0.0161
na 1.7992 0.2042
hn 0.6210 0.0100
c3 1.9069 0.1078
hc 1.4593 0.0208
hc 1.4593 0.0208
c3 1.9069 0.1078
h1 1.3593 0.0208
h1 1.3593 0.0208
n8 2.0486 0.0323
hn 0.6210 0.0100
hn 0.6210 0.0100
cd 1.8606 0.0988
ca 1.8606 0.0988
ca 1.8606 0.0988
h4 1.4235 0.0161
ca 1.8606 0.0988
ha 1.4735 0.0161
ca 1.8606 0.0988
ha 1.4735 0.0161
ca 1.8606 0.0988
h4 1.4235 0.0161
nb 1.8993 0.0941
